# Brexpiprazole inhibits EMT and migration of colorectal cancer cells by downregulating the SREBP1/SNAI1 signaling pathway

**DOI:** 10.3389/fonc.2025.1734678

**Published:** 2026-01-15

**Authors:** Xiaojie Liu, Jingyi He, Ao Ma, Wenjun Xia, Zhiyang Xia, Lu Liu, Zhuoze Wu, Wei Chen

**Affiliations:** 1 Institute of Basic Medicine,North Sichuan Medical College, Nanchong, Sichuan, China; 2 School of Medical Laboratory,North Henan Medical College, Xinxiang, Henan, China; 3 School of Mental Health, North Sichuan Medical College, Nanchong, China

**Keywords:** brexpiprazole, colorectal cancer, EMT, metastasis, SREBP1

## Abstract

**Objective:**

To investigate the role and mechanism of the SREBP1/SNAI1 signalling pathway in the effect of brexpiprazole on the EMT and metastasis of CRC.

**Methods:**

The effects of different concentrations of brexpiprazole on cell migration, cell invasion and protein expression *in vitro* were examined by cell scratch assays, Transwell assays, Western blotting, ELISA, immunofluorescence, and transmission electron microscopy. A dual-luciferase reporter gene assay was used to assess the interactions between SREBP1 and SNAI1. A model of CRC metastasis in nude mice was established, and Western blotting, HE staining, and PET/CT were utilised to explore the effects of brexpiprazole on CRC lung metastasis.

**Results:**

Brexpiprazole significantly inhibited the migration and invasion of CRC cells; downregulated the expression of SREBP1(m), SNAI1 and MMP9; upregulated the expression of E-Cad and ZO1; and decreased the levels of secreted ICAM-1 and VEGF in the supernatant of CRC cells. Western blotting and dual-luciferase assays revealed that SREBP1 could directly regulate the expression of SANI1. On the other hand, *in vivo* experiments revealed that brexpiprazole significantly inhibited the formation of CRC lung metastases, suppressed the expression of SREBP1(m), SNAI1and MMP9, and upregulated the expression of E-Cad and ZO1.

**Conclusion:**

Brexpiprazole inhibits the migration, invasion and metastasis of CRC cells by inhibiting the SREBP1/SNAI1 signalling pathway and downregulating the expression of EMT-related factors.

## 1 Introduction

Colorectal cancer (CRC) is the third most common cancer and the second leading cause of cancer-related death in the world, accounting for approximately 10% of global cancer cases and related deaths each year (1, 2). Despite major advances in CRC treatment in recent years, the recurrence and metastasis of CRC are still the leading causes of patient mortality (2, 3). One study revealed that approximately 86% of patients with advanced CRC develop metastasis and die within 5 years (4). Therefore, it is important to explore new therapies that can prevent and treat CRC metastasis.

The activation of epithelial-mesenchymal transition (EMT) is considered a critical process in the development of tumor metastasis and invasion. Extensive research has demonstrated its pathological role in facilitating the progression of various tumors, including colorectal cancer (CRC), hepatocellular carcinoma, and pancreatic cancer (5–8). Downregulation of the expression of the EMT marker E-cadherin as well as elevation of the expression of vimentin predict the presence of lymph node metastasis, poor tumour differentiation, and poor prognosis in patients with CRC, and the prevention or reversal of EMT in CRC may suppress metastasis, recurrence, and drug resistance (5, 7, 9).

SNAI1 is considered to be a key factor in tumor invasive expression, as it plays a key role in the EMT pathway associated with tumor metastasis (10, 11).Studies showed that SNAI1 expression in endothelial cells controlled growth,angiogenesis and differentiation of breast tumors (12).Moreover, in normal tissues SNAI1 gene was silent, but in tumor tissues its expression was up-regulated, and exerted a functional role by controlling the expression of related proteins (13).Therefore, it is still worth exploring the detailed molecular mechanism of SNAI1 in CRC.

Research has demonstrated that the reprogramming of lipid metabolism can modulate the phenotypic characteristics of tumor cells, with a particular emphasis on EMT (14). Enhanced lipid synthesis and uptake contribute to tumour formation and progression, and the dysregulation of sterol regulatory element binding proteins (SREBPs) plays a central role in these processes and is an important feature of metabolic reprogramming in cancer (15–17).Sterol regulatory element-binding protein 1 (SREBP1), also known as sterol regulatory element-binding transcription factor 1 (SREBF1), is a major transcription factor that regulates lipid metabolism and has become a biomarker for prognosis and drug efficacy monitoring in cancer patients (18–20).Multiple studies have consistently demonstrated that SREBP1 can facilitate tumor invasion and metastasis by modulating EMT of tumor cells, for instance SREBP1 overexpression significantly promotes thyroid cancer cell oxygen consumption, filopodia formation, migration and invasion; SREBP1 silencing significantly increases the expression level of E-cadherin in oesophageal squamous cell carcinoma and decreases the expression levels of N-cadherin, Vimentin, SNAI1, MMP9, and VEGF-c, thereby inhibiting the proliferation, migration and invasion of oesophageal squamous cell carcinoma (ESCC) (15, 16, 21).Recent studies showed that SREBP1 expression not only promoted lipid metabolism and thus promoted the proliferation of tumour cells,but also activated the NF-κB pathway, increased MMP7 expression and promoted CRC cell invasion and metastasis (22).All the aforementioned evidence indicates a potential link between SREBP1 and SNAI1. However, to date, there has been no definitive report elucidating their connection in the context of tumors. This study investigates whether SREBP1 influences the metastasis and invasion of colorectal cancer by modulating the expression of SNAI1.

Recently, antipsychotic drugs have been reported to have potential therapeutic effects for various cancers.Brexpiprazole is a second-generation antipsychotic drug for the treatment of major depressive disorder and schizophrenia with an enhanced medication safety profile (23–25).It was first approved for clinical use in the United States in July 2015 (26–28).It has recently been reported that it has antitumour stem cell (CSC) activity, significantly inhibits the growth of a wide range of tumour cells (lung cancer, pancreatic cancer, and glioblastoma), decreases the expression of survivin and subsequently reverses resistance to EGFR tyrosine kinase inhibitors, thus exerting anticancer effects (29–31). Bioinformatics studies conducted by our group have shown that brexpiprazole can significantly inhibit the lipid metabolic pathway in CRC cells, and it has been demonstrated that brexpiprazole can inhibit the proliferation of tumour cells by inducing the expression of AMPK, downregulating the expression of SREBP1 and regulating the synthesis of lipids in CRC cells (31).

As mentioned above, SREBP1 may be strongly associated with SNAI1 and affect its expression, thereby influencing tumour metastasis. However, whether brexpiprazole can also affect CRC EMT and inhibit invasion and metastasis, as well as the role of SREBP1 and the SNAI1 protein family in its mechanism, are not clearly understood.

In this study, we explored the role of SREBP1/SNAI1 in the effects of brexpiprazole on the EMT and metastasis of CRC by *in vivo* and *in vitro* experimental methods to provide a theoretical and experimental basis for the use of brexpiprazole in antitumour therapy.

## 2 Materials and methods

### 2.1 Cell lines and reagents

HCT116 and SW620 cells were obtained from Procell Life Science & Technology Co., Ltd. (Wuhan, China). Brexpiprazole was purchased from Bide Pharmatech Ltd. (Shanghai, China). Preparation of brexpiprazole storage solution (20 mM): Two milligrams of brexpiprazole was dissolved in 230 μl of DMSO for *in vitro* experiments. Brexpiprazole was dissolved in 5% gum arabic solvent and formulated to a concentration of 5 mg/kg for *in vivo* experiments. The following antibodies were used: E-cadherin (CST, 3195S, Shanghai, China), SREBP1 (Proteintech, 14088-1-AP, Wuhan, China), ZO1 (Affinity, AF5145, Jiangsu, China), SNAI1 (CST, 3879T, Shanghai, China), MMP9 (Huabio, ET1704-69, Hangzhou, China), and SNAI1 (Proteintech, 13099-1-AP, Wuhan, China). The Dual Luciferase Reporter Gene Assay Kit (RG027) was obtained from Beyotime Biotechnology (Shanghai, China). The hSREBP1 expression vector was obtained from VectorBuilder, Inc. (Guangzhou, China). Four SNAI1 siRNAs were designed with the assistance of Shanghai Gene Chemistry Co., Ltd. for use in subsequent experiments. The sequences were as follows: sequence 1, 5′-CCCACUCAGAUGUCAAGAATT-3′; sequence 2, 5′-CAGGACUCUAAUCCAGAGUTT-3′;sequence 3, 5′-CUCCUCUACUUCAGUCUCUTT-3′; and sequence 4, 5′-AUGCUCAUCUGGGACUCUGTT-3′.

### 2.2 Cell culture

The human CRC cell lines HCT116 and SW620 were cultured in McCoy’s 5A medium and high-sugar DMEM, respectively, supplemented with 10% foetal bovine serum and 1% double antibody solution, incubated and grown in an incubator at 37°C with 5% CO2.

### 2.3 Scratch wound healing experiment

A 10 μl pipette tip was used to gently scratch a straight line perpendicular to the horizontal line at the bottom of the well plate, and the cells were observed and imaged under an inverted microscope. Forty-eight hours later, the cells were observed, pictures were taken under an inverted microscope again; three independent experiments were performed.

### 2.4 Transwell invasion experiment

The cell density was adjusted to 2.5×105/ml, 200 μl of cell suspension was added to both the upper chamber of the well plate, and 600 μl of culture medium containing 20% FBS was added to the lower chamber. Forty-eight hours later, the chambers were stained and fixed, and the cells were observed and photographed under an inverted microscope.

### 2.5 Western blotting

The cells in each group were collected, the cell concentration was adjusted to 1×106/ml, the cells were centrifuged, the supernatant was discarded, and lysis solution was added. After gel electrophoresis, the proteins were transferred to PVDF membranes, which were incubated with 5% skim milk powder for 60 min at room temperature. The corresponding primary antibodies were added, and the membranes were incubated at 4°C overnight. The membranes were incubated with the secondary antibodies at room temperature for 1 h, after which they were developed and photographed in a chemiluminescence imaging system. The grayscale values of the protein bands were analysed by ImageJ software.

### 2.6 Cell transfection

The cells were spread in 6-well plates and transfected when the cell density reached 80%. Two hundred microlitres of serum-free medium was diluted with 3 μg of DNA. Two hundred microlitres of serum-free medium was diluted with 6 μl of GP-transfect-Mate transfection reagent. The sample was mixed well and incubated at room temperature for 5 min. The GP-transfect-Mate medium mixture was added dropwise to the DNA medium mixture, mixed well, allowed to stand at room temperature for 15 min, and then transfection was performed immediately. Then, 400 μl of transfection mixture was added to the wells, and the final volume of the mixture was 2 ml. Protein expression was detected after 48 h.

### 2.7 ELISA

The cell culture supernatant was collected and centrifuged at 4 °C for 20 min. Blank wells, standard wells, and sample wells were established, each with 3 duplicate wells. The samples were added to the bottom of the enzyme-labelled well plate, the plate was sealed with a sealing mould, and the plate was placed in an oven at 37 °C for 30 min. The solution was washed 5 times. Fifty microlitres of enzyme reagent was added to each well, and the plates were incubated for 30 min. Fifty microlitres of colour developer A and B were added successively, and the colour was developed at 37 °C for 10 min. Then, termination solution was added to terminate colour development. The OD value of each well was read at 450 nm. The OD value of each well was read at 450 nm, and the corresponding concentration was calculated according to the standard curve of the OD value of the samples.

### 2.8 Immunofluorescence

After fixation, permeabilisation, sealing, primary antibody binding, secondary antibody binding, and DAPI nuclear staining for 5 min, the cells were inoculated into confocal dishes, and 3 compound wells were established for each of the control and experimental groups. The cells were observed and photographed under a fluorescence microscope.

### 2.9 Dual-luciferase promoter assay

The promoter region of SNAI1 was inserted into a luciferase reporter gene vector to construct a reporter gene plasmid. After the cells were cotransfected with the SREBP1 plasmid and reporter gene plasmid, the fluorescence was measured, and the data were analysed.

### 2.10 Animal experiments

The animal husbandry and experimental procedures were reviewed and approved by the Ethics Committee of North Sichuan Medical College (permit number: 2023095) in compliance with the Guide for the Care and Use of Laboratory Animals published by the US National Institutes of Health (NIH publication no. 85-23, revised 1996).

### 2.10.1 Construction of an animal model of CRC cell metastasis

**Figure 1:**
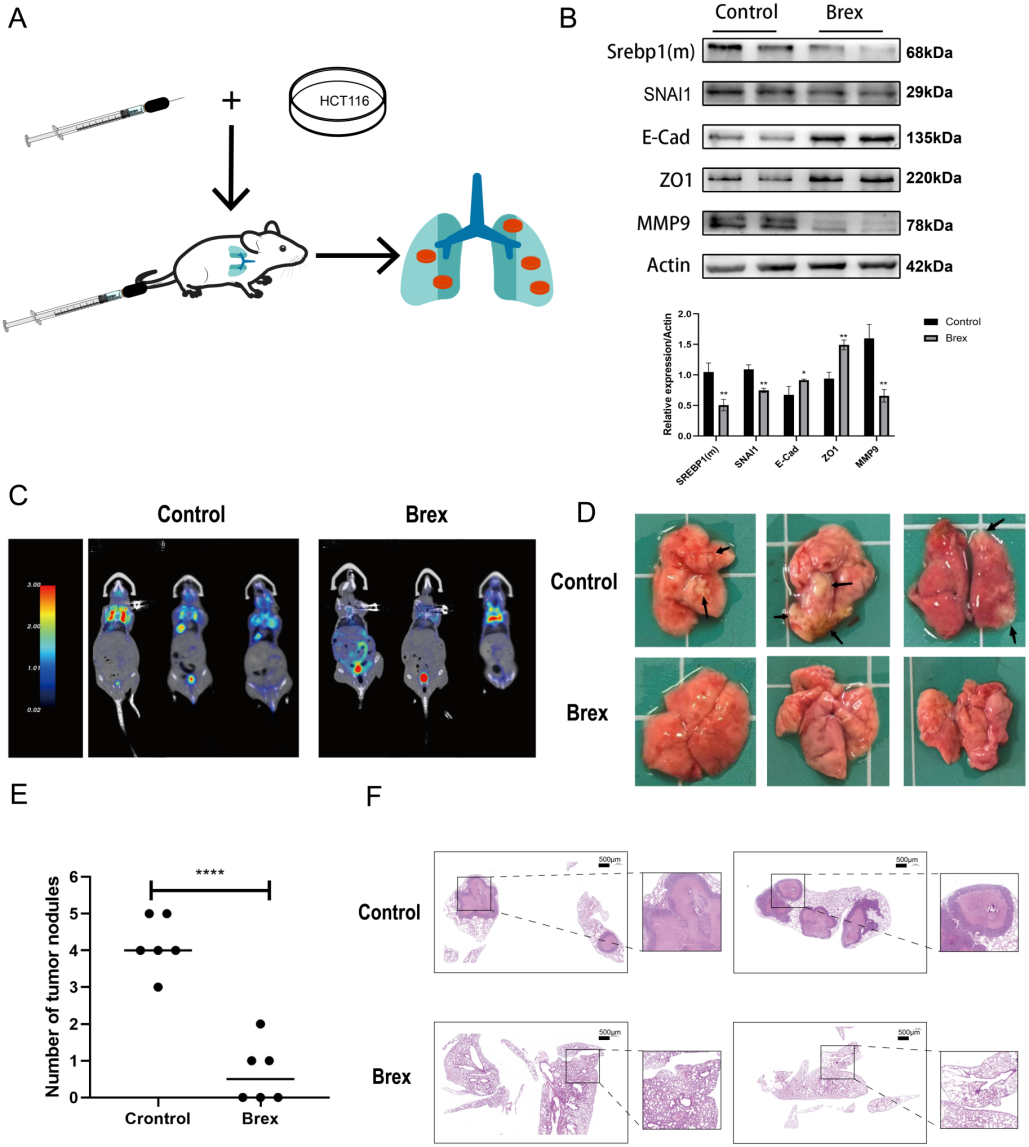
Brexpiprazole reduces the metastatic ability of CRC cells *in vivo* (n=6). **(A)** .Construction of a lung metastasis model in nude mice(n=6 mice per group). **(B)** Effects of brexpiprazole on the protein expression levels of SREBP1 (m), SNAI1, E-Cad, ZO1, and MMP9 in CRC *in vivo*. β-Actin was used as a loading control. Blots are representative of three independent experiments.Data were analyzed by one-way ANOVA. *p<0.05, **p<0.01, ***p<0.001. **(C)** PET/CT scanning for detecting metastasis in the lungs of nude mice. **(D)** Arrows indicate CRC metastatic foci; there were basically no metastatic foci in the experimental group. **(E)** Statistical analysis of the number of metastatic lung nodules.Data are presented as mean ± SD. Statistical significance was determined by Student’s t-test. ****p<0.0001. **(F)** HE staining of lung tissue. ****p<0.0001.

Twelve male BALB/c nude mice 4–6 weeks old, SPF grade and weighing 20 ± 2 g were selected and acclimatised to a specific-pathogen-free (SPF)-grade environment for one week. HCT116 cells in the logarithmic growth phase were collected, the cell density was adjusted to 1×10^6^/ml, and 100 µl of cell suspension was injected into the tail vein of nude mice (**Figure 1A**). The nude mice were randomly divided into a control group and an experimental group (n = 6 per group). The control group received 5% gum arabic (100 µl) by oral gavage once daily, while the experimental group was administered the same volume of brexpiprazole suspension via oral gavage at a dose of 5 mg/kg, once daily.Animal health and potential signs of toxicity were monitored daily throughout the experiment.Eight weeks later, the nude mice were anaesthetised with 1% pentobarbital and then killed by cervical dislocation, after which the lungs were harvested; part of each lung was preserved at –80°C, and the other part was placed in 4% paraformaldehyde solution for subsequent HE staining.

### 2.10.2 HE staining

Tumour tissue was fixed in 4% paraformaldehyde at room temperature for 24 h, embedded in a wax block and cut into 4 μm thick slices. The sections were stained with haematoxylin, rinsed in water, stained with eosin for several seconds and rinsed in running water. The sections were sequentially dewaxed in ethanol solutions of varying concentrations, sealed, observed under a microscope, and imaged.

### 2.10.3 PET/CT scanning

Nude mice were anaesthetised by placing them in an anaesthesia box with a concentration of 2.5% isoflurane for 10 min; each mouse was injected with 400 µci of active 18F-FDG and placed into the PET/CT scanning unit in the prone position for detection and imaging.

### 2.11 Statistical analysis

All the data were analysed with GraphPad Prism software version 9.5.0, and the results are expressed as the mean ± standard deviation (mean ± SD). Multigroup data were assessed by one-way ANOVA, and two-group data were assessed via t tests. All experiments were repeated three times. The level of significance was set at p<0.05, *p<0.05, **p<0.01, ***p<0.001, and ****p<0.0001.

## 3 Results

### 3.1 Brexpiprazole inhibits the migration and invasion of CRC cells

**Figure 2:**
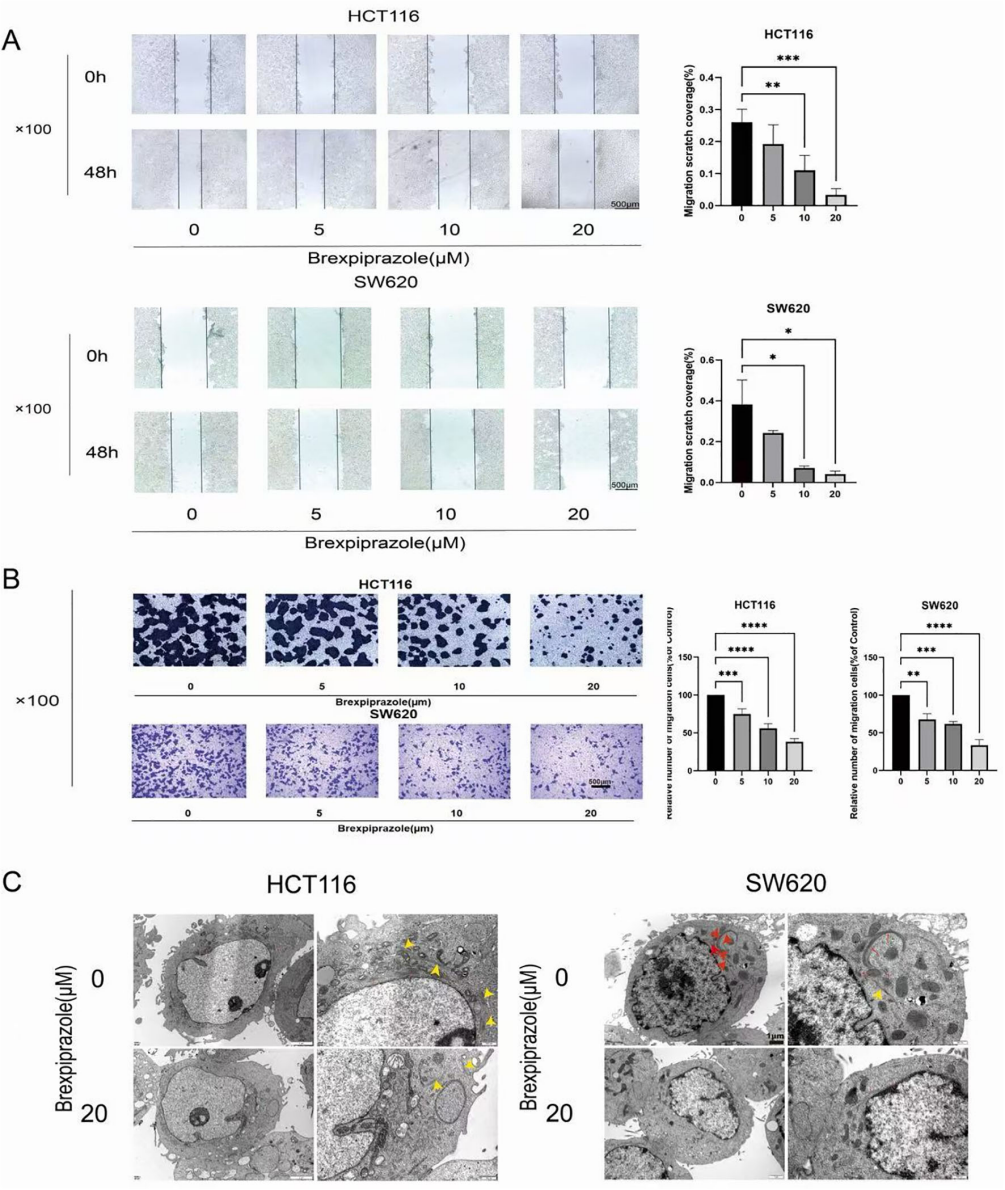
Brexpiprazole inhibits the *in vitro* migration and invasion of CRC cells (n=3). **(A, B)** Scratch assays and Transwell assays revealed that brexpiprazole inhibited the *in vitro* migration and invasion ability of the CRC cell lines HCT116 and SW620.Data are from three independent experiments (biological replicates) and were analyzed by Student’s t-test. Data are presented as mean ± SD. *p<0.05, **p<0.01, ***p<0.001. **(C)** Transmission electron microscopy was used to observe the effect of 20 µM brexpiprazole on cellular microfilaments.

We treated CRC cell lines (HCT116 and SW620) with different concentrations of brexpiprazole (0, 5, 10, or 20 µM) for 48 h. Scratch experiments revealed that brexpiprazole significantly reduced the scratch area (indicating cell migration) in a dose-dependent manner (**Figure 2A**). On the other hand, Transwell experiments revealed that the number of CRC cells from the upper chamber that invaded the lower chamber gradually decreased compared to that in the control group (**Figure 2B**). We also observed the ultrastructure of CRC cells treated with brexpiprazole (20 µM) by transmission electron microscopy and found that the number of microfilaments in the cells was significantly reduced (**Figure 2C**, yellow arrows indicate the enlarged area of the image). These results clearly demonstrated that brexpiprazole inhibits the migration and invasion of CRC cells.

### 3.2 Brexpiprazole inhibits EMT in CRC cells

**Figure 3:**
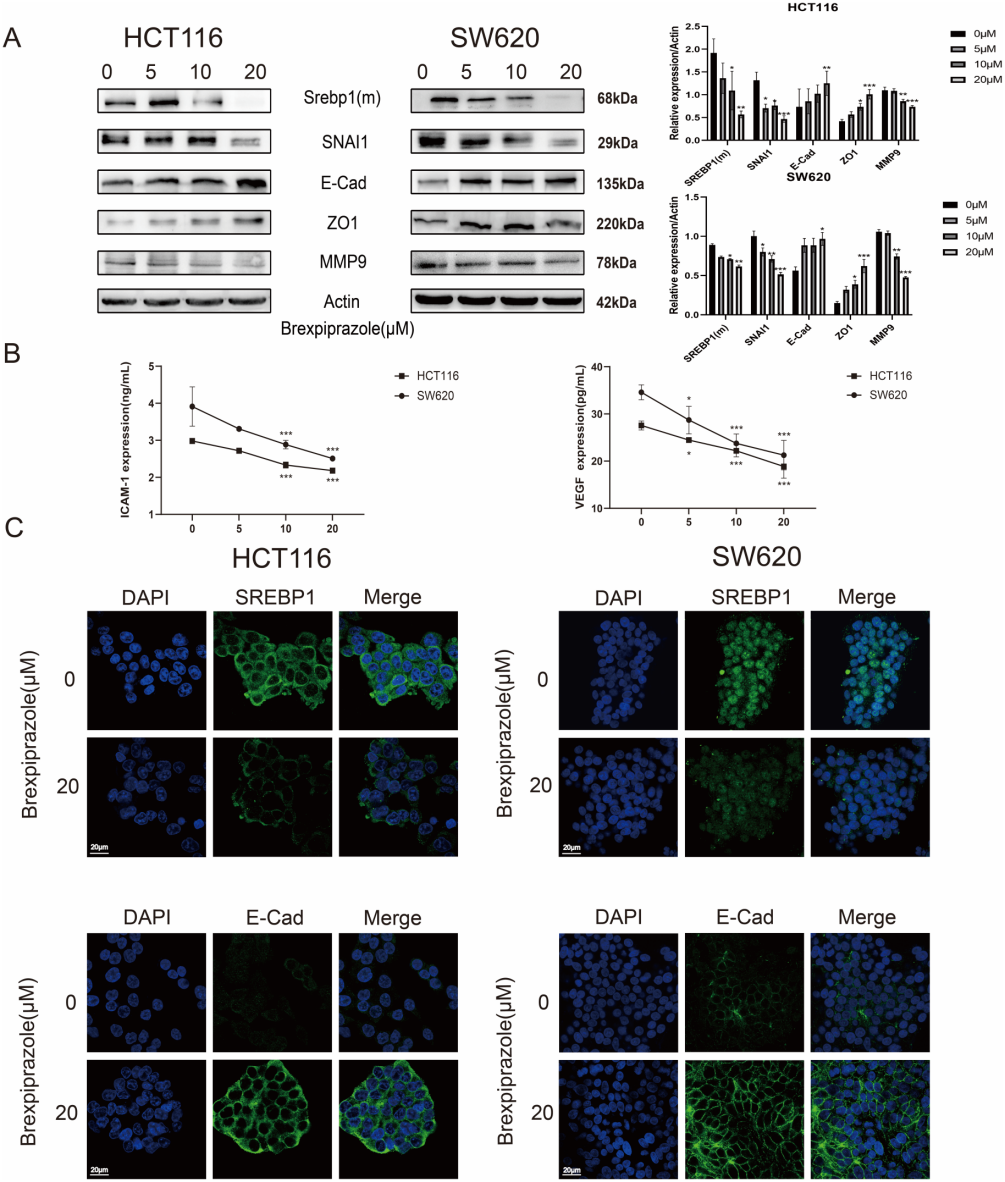
Brexpiprazole inhibits the protein expression of EMT-related markers as well as SREBP1 and E-Cad in CRC cells (n=3). **(A)**. Western blotting analysis of the effects of different concentrations of brexpiprazole on the protein expression levels of SREBP1 (m), SNAI1, E-Cad, ZO1, and MMP9 in HCT116 and SW620 CRC cells. β-Actin was used as a loading control. Blots are representative of three independent experiments.Data were analyzed by one-way ANOVA. *p<0.05, **p<0.01, ***p<0.001. **(B)**. ELISA was used to detect the expression levels of ICAM-1 and VEGF in the supernatants of the cells.Data are from three independent experiments and were analyzed by one-way ANOVA. Data are presented as mean ± SD. *p<0.05, **p<0.01, ***p<0.001. **(C)**. Immunofluorescence was used to detect the localisation and expression of SREBP1 and E-Cad in cells after treatment with 20 µM brexpiprazole.Representative images are shown. Scale bar, 20 µm.

The expression of EMT-related proteins and related factors in HCT116 and SW620 cells was detected by Western blotting and ELISA. The results showed that brexpiprazole downregulated the expression of the mesenchymal-associated proteins SNAI1 and MMP9 and upregulated the expression of the epithelial-associated proteins ZO1 and E-Cad in a dose-dependent manner (**Figure 3A**). The expression of ICAM-1 and VEGF gradually decreased with increasing brexpiprazole concentrations (**Figure 3B**).

### 3.3 Expression localisation assay of SREBP1 and the key EMT protein E-Cad

To further assess the localisation and distribution of SREBP1 and the key EMT protein E-Cad in CRC cells after treatment with brexpiprazole, we used immunofluorescence to detect their expression in the cells. Compared to that in the control group, SREBP1 expression decreased in both cell lines after treatment with 20 µM brexpiprazole; SREBP1 was mainly concentrated in the cytoplasm of HCT116 cells but was mainly distributed in the nucleus of SW620 cells. These findings suggested that for the HCT116 cell line of *in situ* cancer origin, brexpiprazole exerts antitumour effects by downregulating SREBP1 expression, while brexpiprazole exerts antitumour effects on the SW620 cell line of lymphatic metastasis origin by affecting the transcriptional function of SREBP1. Compared with that in the control group, the expression of E-Cad was increased in the brexpiprazole group, and it was mainly distributed in the cytomembrane (**Figure 3C**). The above results indicated that brexpiprazole inhibits the expression of SREBP1, affects its intracellular localisation, and promotes the localisation of E-Cad to the cell membrane.

### 3.4 Brexpiprazole regulates EMT through SREBP1/SNAI1 and inhibits CRC metastasis

**Figure 4:**
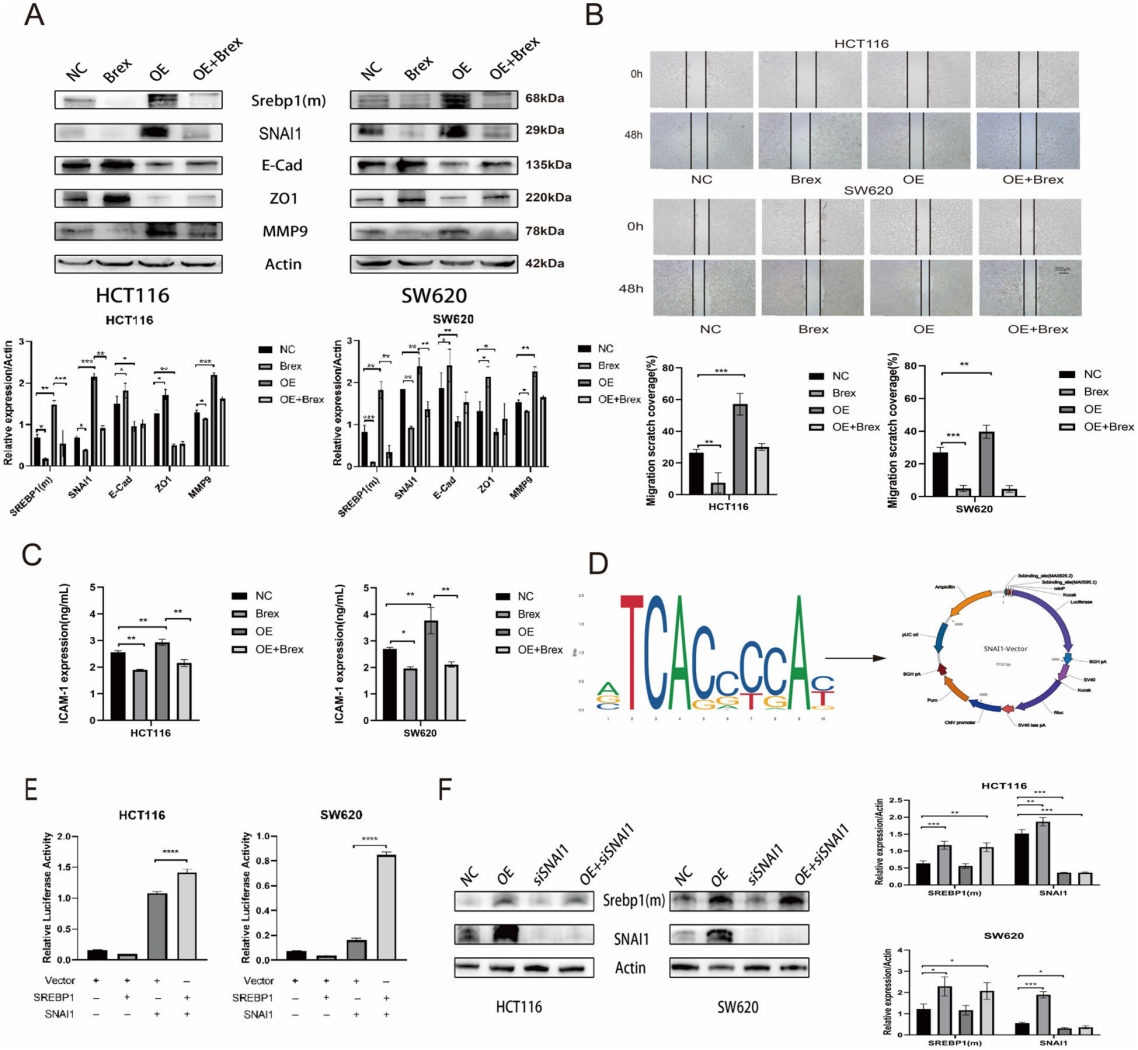
Brexpiprazole regulates EMT-related proteins through SREBP1/SNAI1, thereby affecting CRC metastasis (n=3). **(A, B)**. After SREBP1 was overexpressed, Western blotting was used to detect the protein expression levels of SREBP1 (m), SNAI1, E-Cad, ZO1, and MMP9 in the CRC cell lines HCT116 and SW620, and a scratch assay was used to detect the *in vitro* migratory ability of the cells. **(C)**. For each group, the volume of the control corresponded to the volume used in the experimental group. For the NC group, DMSO and control plasmid for SREBP1 were added; for the Brex group, 20 μM brexpiprazole formulated with DMSO and the control plasmid were added; for the OE group, DMSO and the SREBP1 plasmid were added; for the OE+Brex group, 20 μM brexpiprazole formulated with DMSO and the SREBP1 plasmid were added. ELISA was performed to examine the expression levels of ICAM-1 and VEGF in the cell supernatants. **(D)**. The JASPAR database was used to predict the binding sites for SREBP1 and SNAI1, and a dual-luciferase reporter gene plasmid for SNAI1. **(E)**. Dual-luciferase promoter assays demonstrated that SREBP1 directly regulates SNAI1. There were 4 groups: Group 1, which was transfected with equal amounts of SREBP1 and SNAI1 control plasmids; Group 2, which was transfected with equal amounts of SREBP1 plasmid and SNAI1 control plasmid; Group 3, which was transfected with equal amounts of SREBP1 control plasmid and SNAI1 dual-luciferase reporter gene plasmid; and Group 4, which was transfected with equal amounts of SREBP1 plasmid and SNAI1 dual-luciferase reporter gene plasmid. **(F)**. The Western blotting results indicated that SREBP1 could increase SNAI1 expression. NC indicates transfection with equal amounts of SREBP1 control plasmid and siSNAI1 control RNA; OE indicates transfection of SREBP1 plasmid and siSNAI1 control RNA; siSNAI1 indicates transfection of SREBP1 control plasmid and siSNAI1; and OE+siSNAI1 indicates transfection of SREBP1 plasmid and siSNAI1. Data in **(A-C, E, F)** are from three independent experiments. Data are presented as mean ± SD and were analyzed by one-way ANOVA. *p<0.05, **p<0.01, ***p<0.001, ****p<0.0001.

We generated a CRC cell line with SREBP1 overexpression and a CRC cell line with low SNAI1 expression to further clarify the role of SREBP1/SNAI1 in the metastatic process of CRC cells. Western blotting was used to detect the expression of SREBP1 and EMT-related proteins. After overexpression of SREBP1, the levels of the mesenchymal phenotype-associated proteins SNAI1 and MMP9 were elevated, the levels of the epithelial phenotype-associated proteins ZO1 and E-Cad were reduced (**Figure 4A**); the expression of ICAM-1 and VEGF was elevated (**Figure 4C**). We also assessed the migration ability of the cells, and the scratch assay showed that overexpression of SREBP1 increased the *in vitro* migration ability of the cells (**Figure 4B**). These results all illustrated that SREBP1 promotes the migration of CRC cells. To clarify the relationship between SREBP1 and SNAI1, we predicted the potential binding sites of SREBP1 and SNAI1 through the JASPAR database and designed and constructed a dual-luciferase reporter gene plasmid containing the SNAI1 promoter region (**Figure 4D**). A dual-luciferase promoter assay showed that SREBP1 directly regulated SNAI1, which in turn affected the metastasis of CRC cells (**Figure 4E**). Western blotting analysis revealed that SREBP1 overexpression increased SNAI1 expression, and SNAI1 knockdown did not significantly affect SREBP1 expression, suggesting that SREBP1 can directly regulate the metastasis of SNAI1, thus affecting the metastasis of CRC cells (**Figure 4F**).

### 3.5 Brexpiprazole inhibits CRC metastasis *in vivo*

To investigate the effect of brexpiprazole on *in vivo* metastasis in animals, we constructed an animal model of CRC metastasis (**Figure 1A**). Western blotting analysis of the expression of SREBP1 (m) and EMT-related factors in the fresh lung tissues of nude mice revealed that compared with that in the control group, the expression of SREBP1 (m), SNAI1, and MMP9 in the lung tissues of the experimental group was significantly decreased, and the expression of E-Cad and ZO1 was increased (**Figure 1B**). PTE/CT revealed significant 18F-FDG aggregation in the lungs of control mice, which was greatly reduced by brexpiprazole (**Figure 1C**). Pathological examination of the lung tissue revealed a significant reduction in the number of metastatic foci in the lungs of the brexpiprazole group compared to that in the lungs of the control group. (**Figures 1D, E**). HE staining revealed that the lung metastases of nude mouse cancer cells were significantly reduced in the experimental group compared with the control group (**Figure 1F**).

### 3.6 Diagram of the mechanism by which brexpiprazole exercises its function

**Figure 5:**
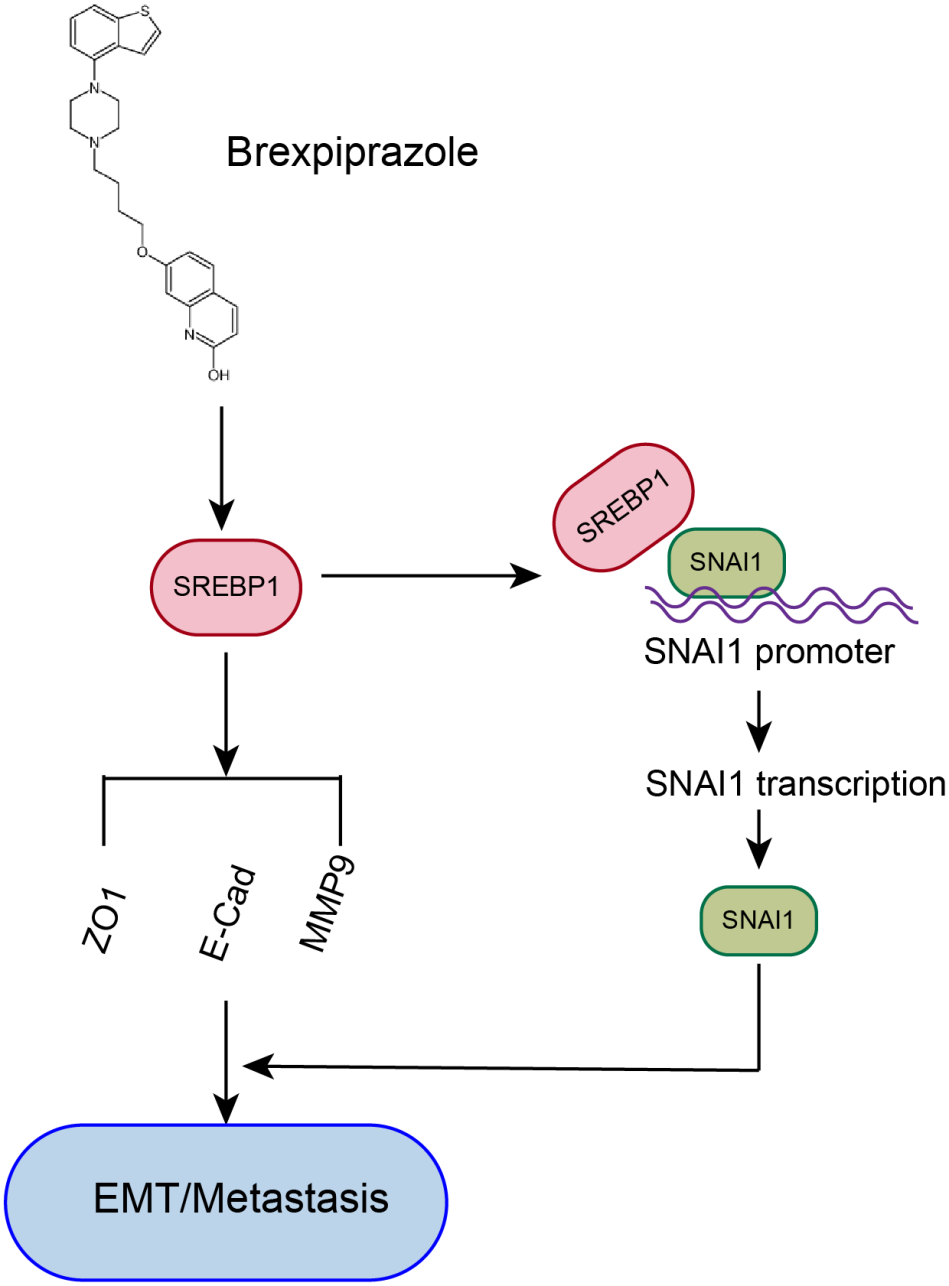
Diagram of the mechanism by which brexpiprazole exercises its function.

Brexpiprazole downregulates the expression of SREBP1, which can bind to and promote the transcription and expression of SNAI1 subsequently modulates the expression of E-Cadherin, MMP9 and ZO-1. Therefore, Brexpiprazole inhibits the EMT process by downregulating both SREBP1 and SNAI1 expression, ultimately suppressing the migration of colorectal cancer cells (**Figure 5**).

## 4 Discussion

The occult progression and metastasis of colorectal cancer are the primary contributors to its mortality. Numerous studies have demonstrated that the invasion and metastasis of colorectal cancer are closely associated with the reprogramming of lipid metabolism (32, 33).For instance, elevated expression of SREBP1, a key regulatory factor for the synthesis and metabolism of cholesterol and fatty acids, is associated with metastasis, invasion, and a poor prognosis in solid tumours such as endometrial, renal, hepatocellular, and breast cancers; furthermore, the inhibition of SREBP1 may inhibit the growth, migration, and invasion of cancer cells (21, 22).The research conducted by our group demonstrates that brexpiprazole can effectively suppress the metastasis and invasion of CRC cells by down-regulating the expression of SREBP1. Furthermore, pronounced dose-dependent effects have been consistently observed in both *in vitro* and *in vivo* experiments. Consequently, targeting SREBP1 could be an effective way to inhibit tumour cell proliferation and malignant progression (15, 34).

The inhibitory effects of brexpiprazole on cell proliferation have been demonstrated in various tumour types, including non-small cell lung cancer, pancreatic cancer, and glioblastoma, but the mechanism of action is not fully understood (26, 34, 35).Recent studies revealed that brexpiprazole can remarkably synergize with cetuximab to significantly inhibit xenograft tumor growth in CRC.This finding overcomes the problem of tumor drug resistance and is important in cancer treatment (36).In our preliminary studies, the research group explored the complex relationship between the anti-tumor effects of brexpiprazole and lipid metabolism in CRC. Our findings confirmed that brexpiprazole effectively inhibited CRC cells proliferation through the AMPK/SREBP1 signaling pathway (31). In this study, we further demonstrated using scratch assay and transwell migration assay that brexpiprazole could significantly inhibit the invasion and metastasis abilities of HCT116 and SW620 human CRC cell lines. Based on these observations, we hypothesize that brexpiprazole may also regulate SREBP1 to induce EMT in CRC cells, thereby affecting their invasion and metastasis capacity. However, the underlying mechanism remains to be elucidated.

The snail1 protein, encoded by the SNAI1 gene, is a zinc-finger transcription factor that plays a crucial role in embryonic development and tumor metastasis. Literature reports that SREBP1 can regulate the stability of SNAI1 to promote cancer cell migration and invasion (18, 19).Our research group observed that overexpression of SREBP1 significantly increased migratory and invasive abilities of colon cancer cells, accompanied by an increase in SNAI1 expression levels. However, knocking down SNAI1 did not significantly alter the expression of SREBP1. These findings suggest that SREBP1 is an upstream regulatory factor for SNAI1. Brexpiprazole may potentially act through modulation of the SREBP1/SNAI1 signaling pathway to exert its effects in cancer biology.

Clinical analyses have confirmed that elevated SNAI1 expression is correlated with tumour size, lymph node metastasis, distant metastasis, clinical stage/grade, and a poor prognosis in CRC patients (37–39).SNAI1 significantly inhibits E-Cad, regulates the expression of the matrix metalloproteinases MMP2 and MMP9, and promotes the invasion ability of CRC cells, which represents the acquisition of cancer stem cell-like characteristics and promotes drug resistance, tumour recurrence and metastasis (39–41). In our study, the expression of SREBP1, MMP9 and SNAI1 in CRC cells treated with brexpiprazole significantly decreased concentration-dependently. Meanwhile, the expressions of E-Cad and ZO-1 increased. To further demonstrate that brexpiprazole inhibits CRC cell invasion and metastasis through downregulating SREBP1, we established a colon cancer cell line overexpressing SREBP1 following transfection. In this study, cells with overexpression exhibited significantly enhanced migratory and invasive capacities, accompanied by increased SNAI1 expression and decreased E-Cad and ZO-1 expressions. The expression decreases of E-cad and ZO-1,as the tight junction proteins,indicated a weakened the barrier function between cells, allowing tumor cells to more easily invade into the vasculature and undergo metastasis.These findings suggest that brexpiprazole functions through the SREBP1/SNAI1 pathway in regulating the EMT process and promoting CRC cell metastasis(**Figure 5**). Furthermore, transmission electron microscopy analysis of SREBP1 expression localization and distribution within CRC cells treated with brexpiprazole revealed an interesting observation: despite a significant decrease in intracellular expressions of both SREBP1, HCT116 human colon cancer cells exhibited the majority of their SREBP1 localized in the cytoplasm, whereas SW620 human colon cancer cells showed increased nuclear localization. These results indicate that brexpiprazole downregulating SREBP1 expression exerts anti-tumor effects through different mechanisms: for locally originating HCT116 cells, it functions by reducing SREBP1 expression; while for lymphtransferase originating SW620 cells, it inhibits the transcriptional activity of SREBP1. This could be one of the possible reasons for the differential antitumor activities observed with brexpiprazole in these two cell lines.

To further clarify whether SREBP1 can directly act on SNAI1, we used the JASPAR database to predict the potential binding sites of the transcription factor SREBP1 to SNAI1, constructed a dual-luciferase reporter gene plasmid for SNAI1, and performed a dual-luciferase promoter assay, which revealed that SREBP1 can directly bind to the promoter of SNAI1 at the gene level (**Figure 5**).

Unfortunately, we only verified the regulatory relationship between SREBP1 and SNAI1 at the gene level through dual-luciferase assays, and evidence of their interaction at the protein level is still insufficient. The relationship between SREBP1 and SNAI1 will be further validated in subsequent studies through the application of coimmunoprecipitation or mass spectrometry techniques.

## 5 Conclusion

In summary, brexpiprazole regulates the expression of EMT-related factors in CRC cells by inhibiting the SREBP1/SNAI1 signalling pathway, thus suppressing their invasion and metastasis. These findings suggest that SREBP1 could be a new target for the prevention and treatment of invasion and metastasis in CRC patients and provided a theoretical and experimental basis for the use of brexpiprazole for preventing and controlling CRC.

## Data Availability

The raw data supporting the conclusions of this article will be made available by the authors, without undue reservation.
