# Supplementary figures and images for: Brexpiprazole inhibits EMT and migration of colorectal cancer cells by downregulating the SREBP1/SNAI1 signaling pathway (part 1 of 4)
Source: Front Oncol. 2026 Jan 15;15:1734678. doi: 10.3389/fonc.2025.1734678 (PMC12852020; doi:10.3389/fonc.2025.1734678)

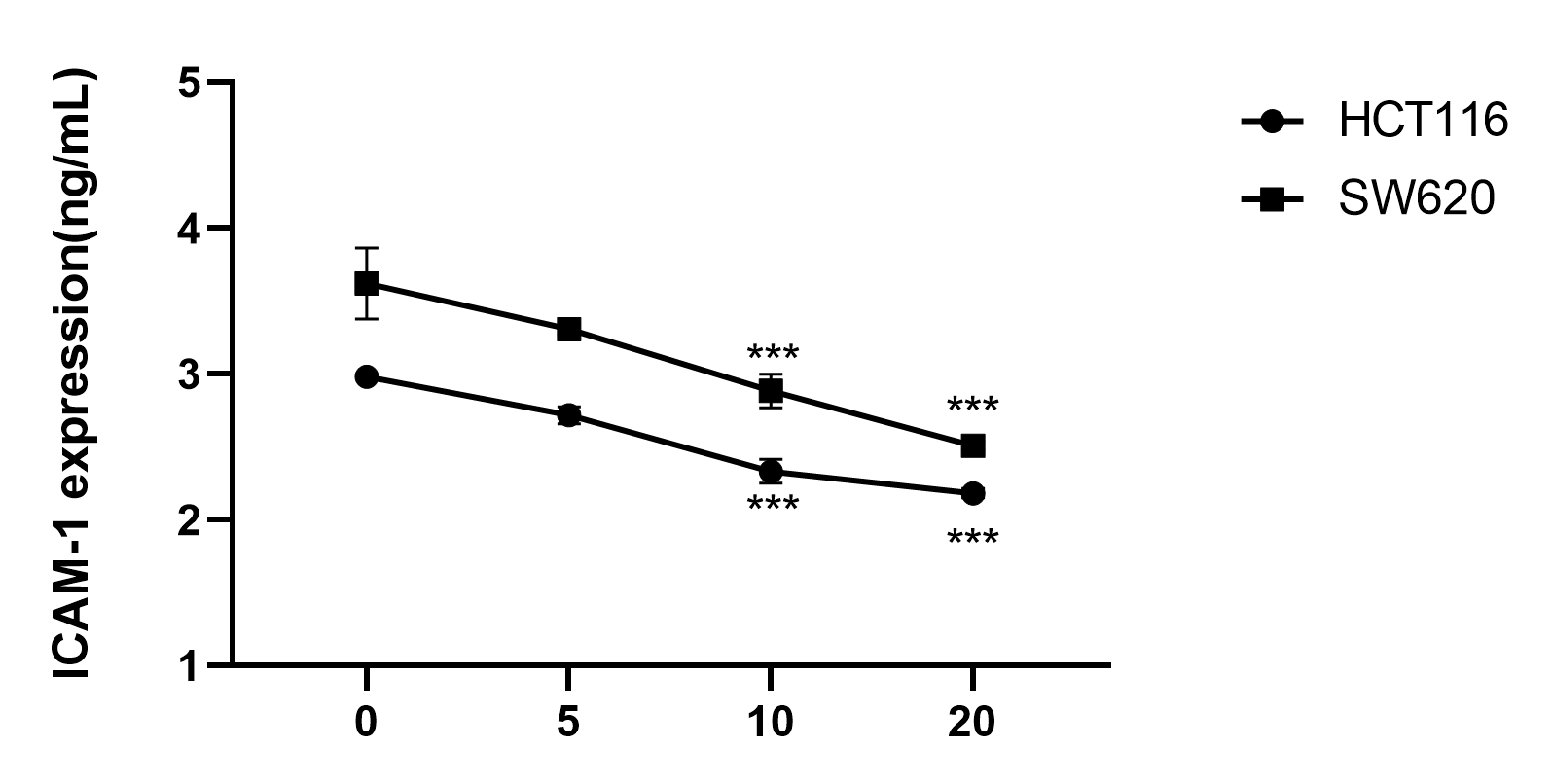

Supplement: Supplementary file 1 [file SupplementaryFile1.zip › ELISA数据/ICAM-1/ICAM1.tif]

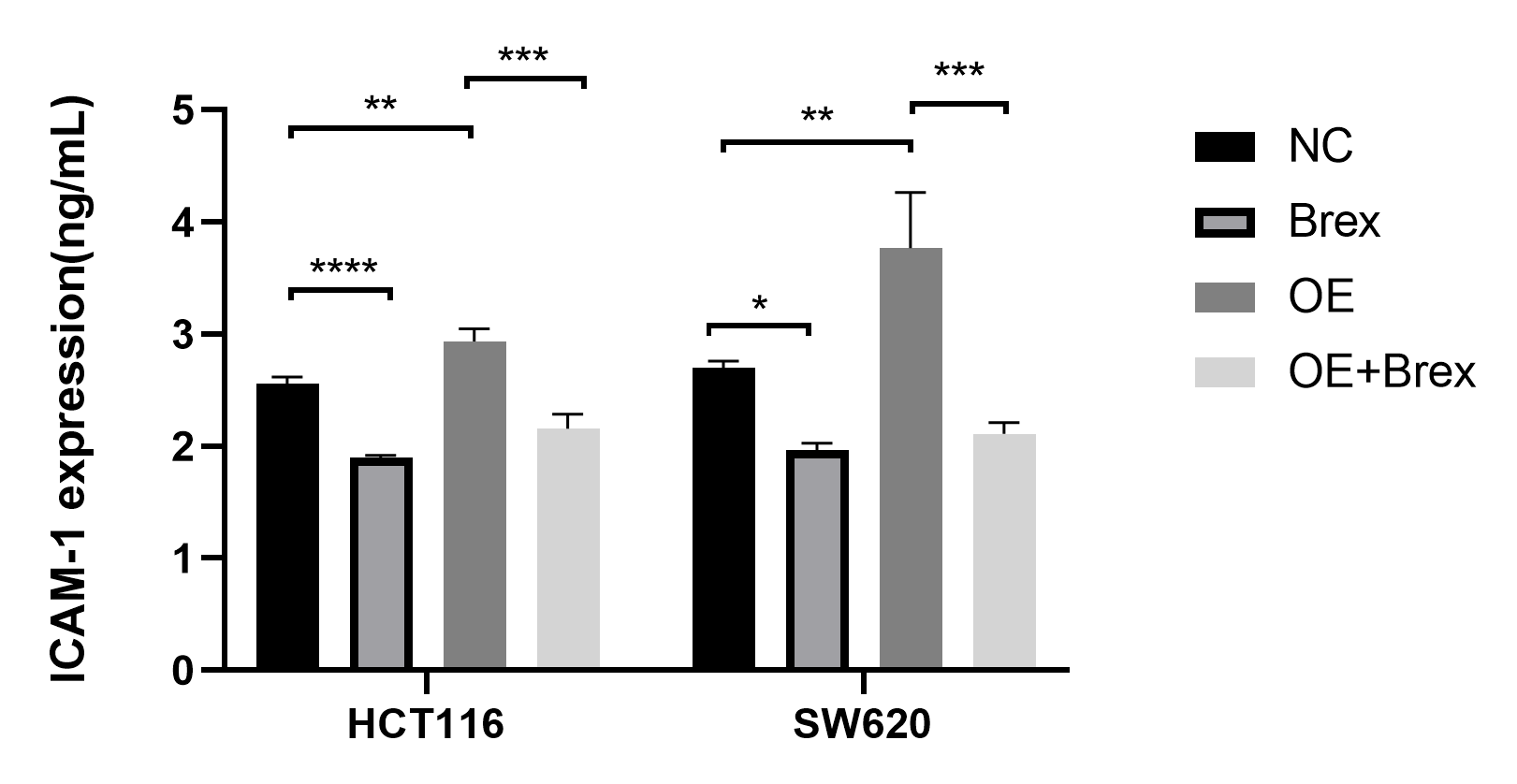

Supplement: Supplementary file 1 [file SupplementaryFile1.zip › ELISA数据/ICAM-1/ICAM1`.tif]

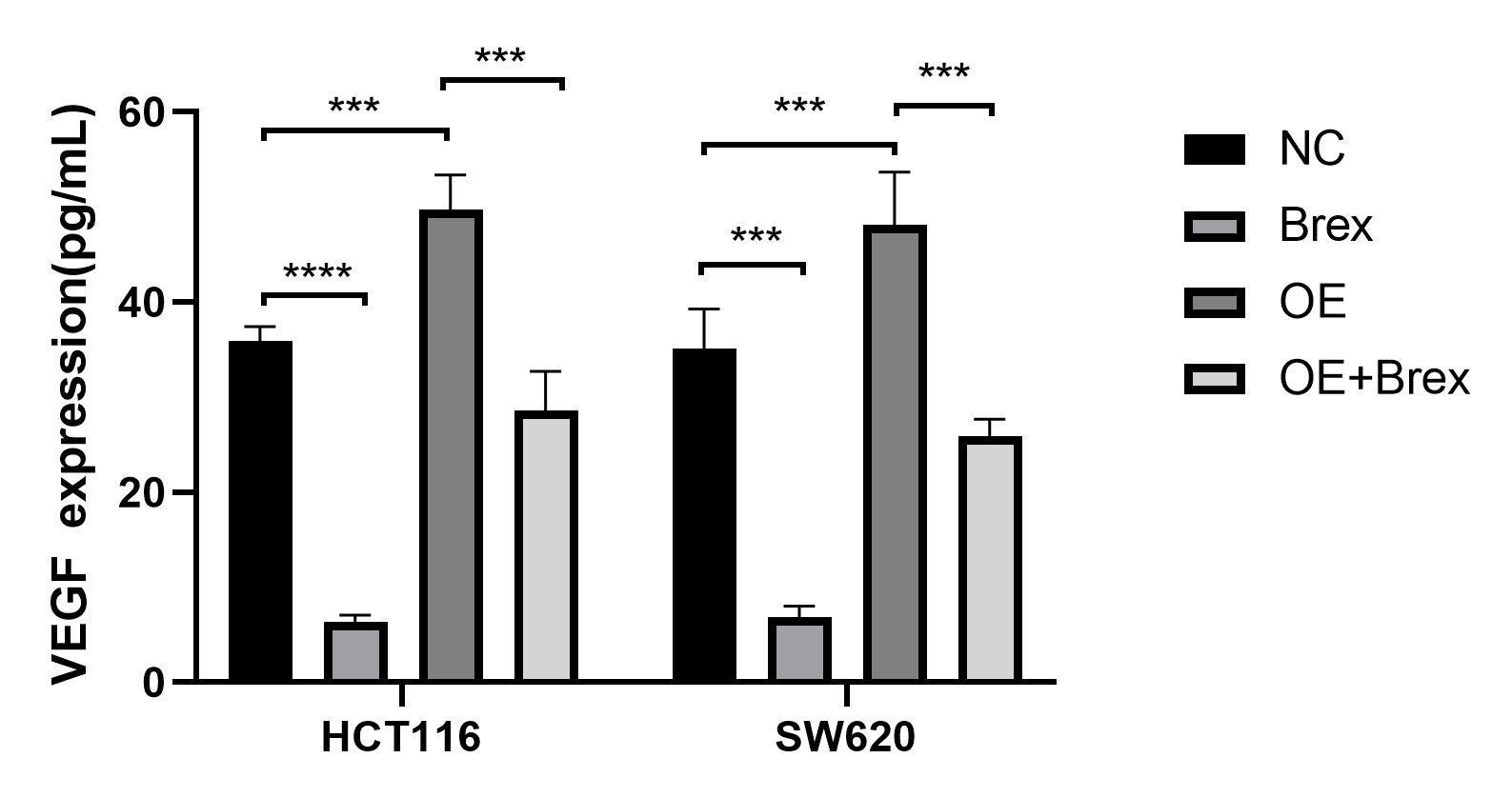

Supplement: Supplementary file 1 [file SupplementaryFile1.zip › ELISA数据/VEGF/VEGF--.tif]

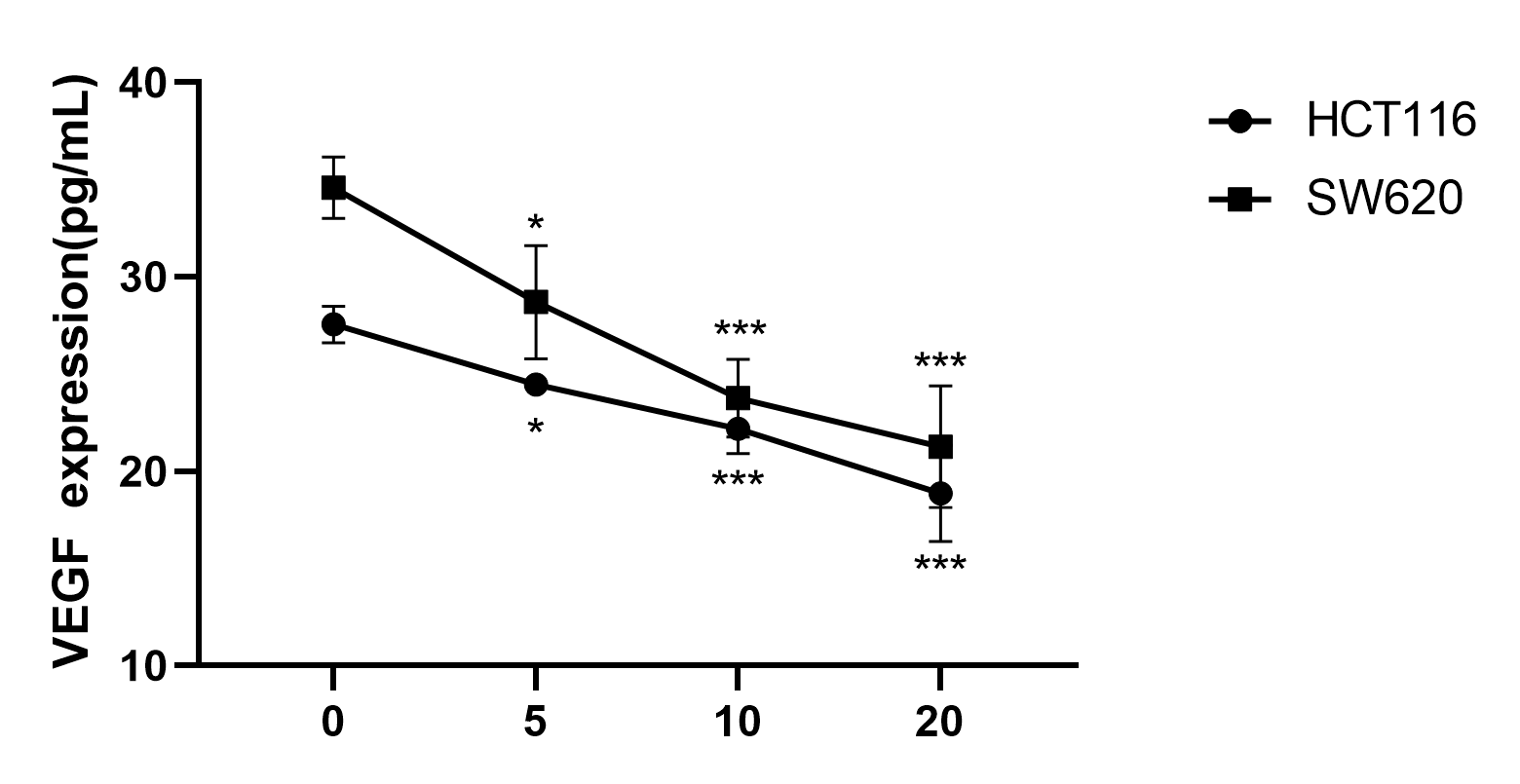

Supplement: Supplementary file 1 [file SupplementaryFile1.zip › ELISA数据/VEGF/VEGF5.tif]

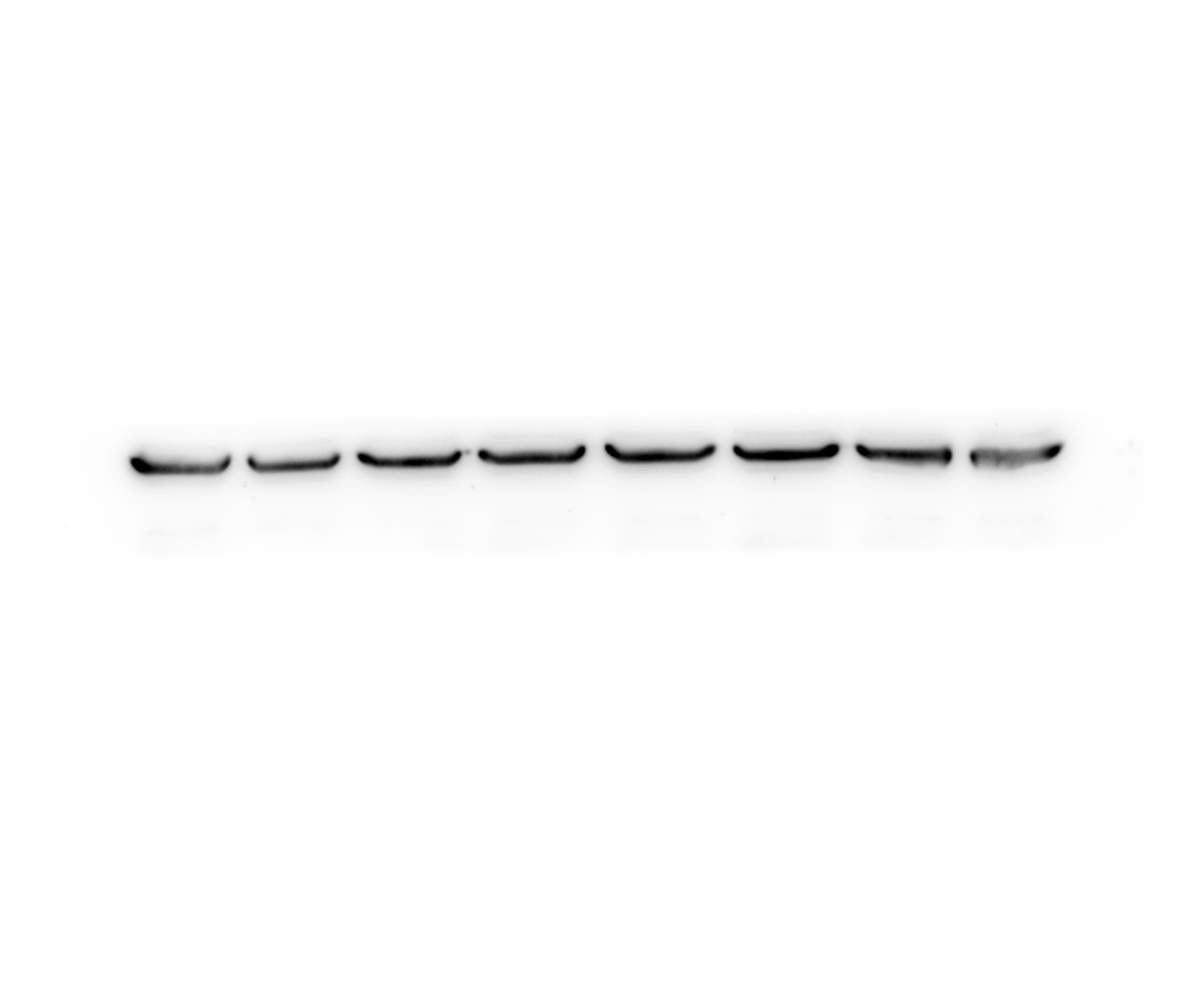

Supplement: Supplementary file 2 [file SupplementaryFile2.zip › WB数据/116/116-wb/actin/116-actin--.tif]

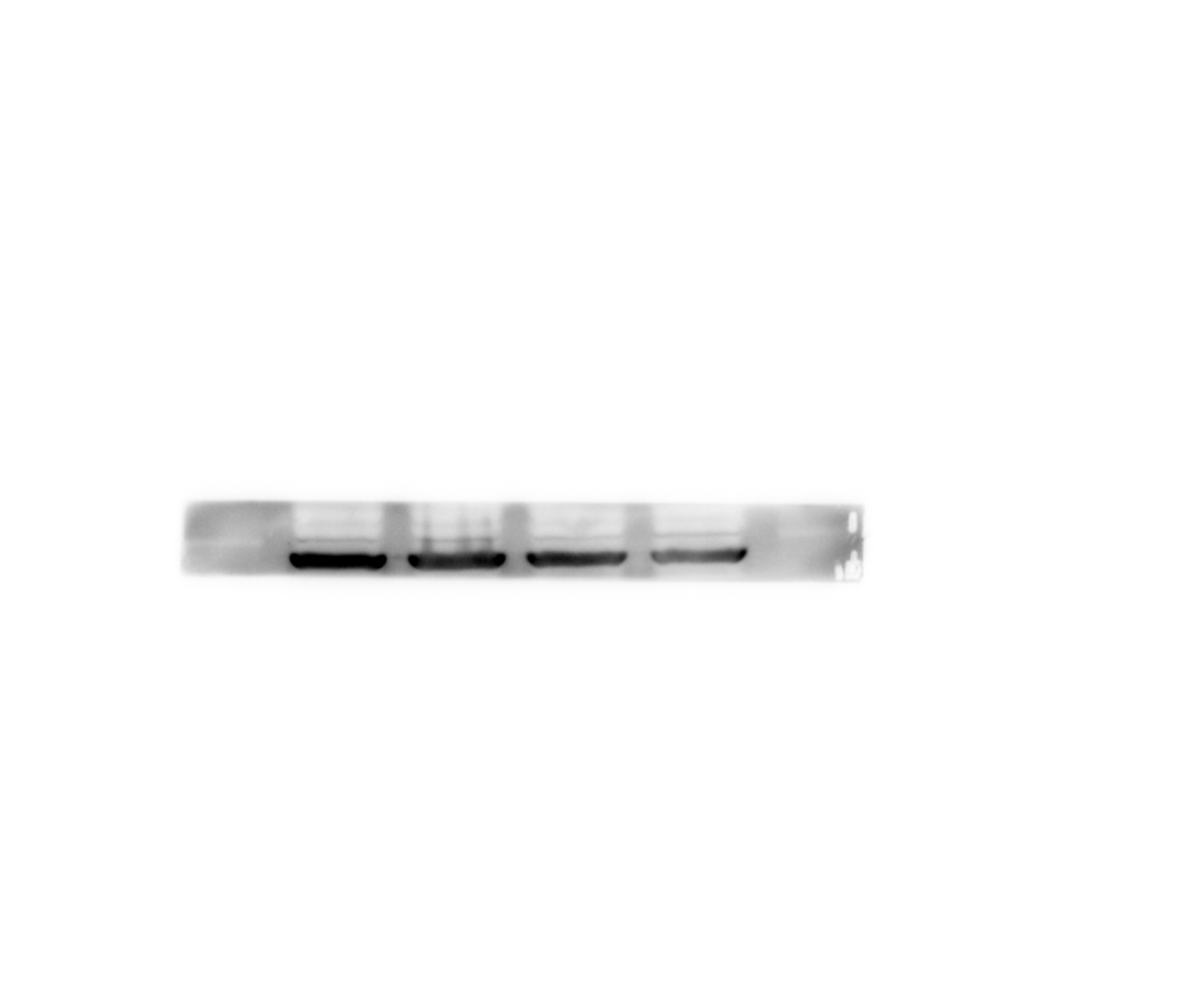

Supplement: Supplementary file 2 [file SupplementaryFile2.zip › WB数据/116/116-wb/actin/116-actin-.tif]

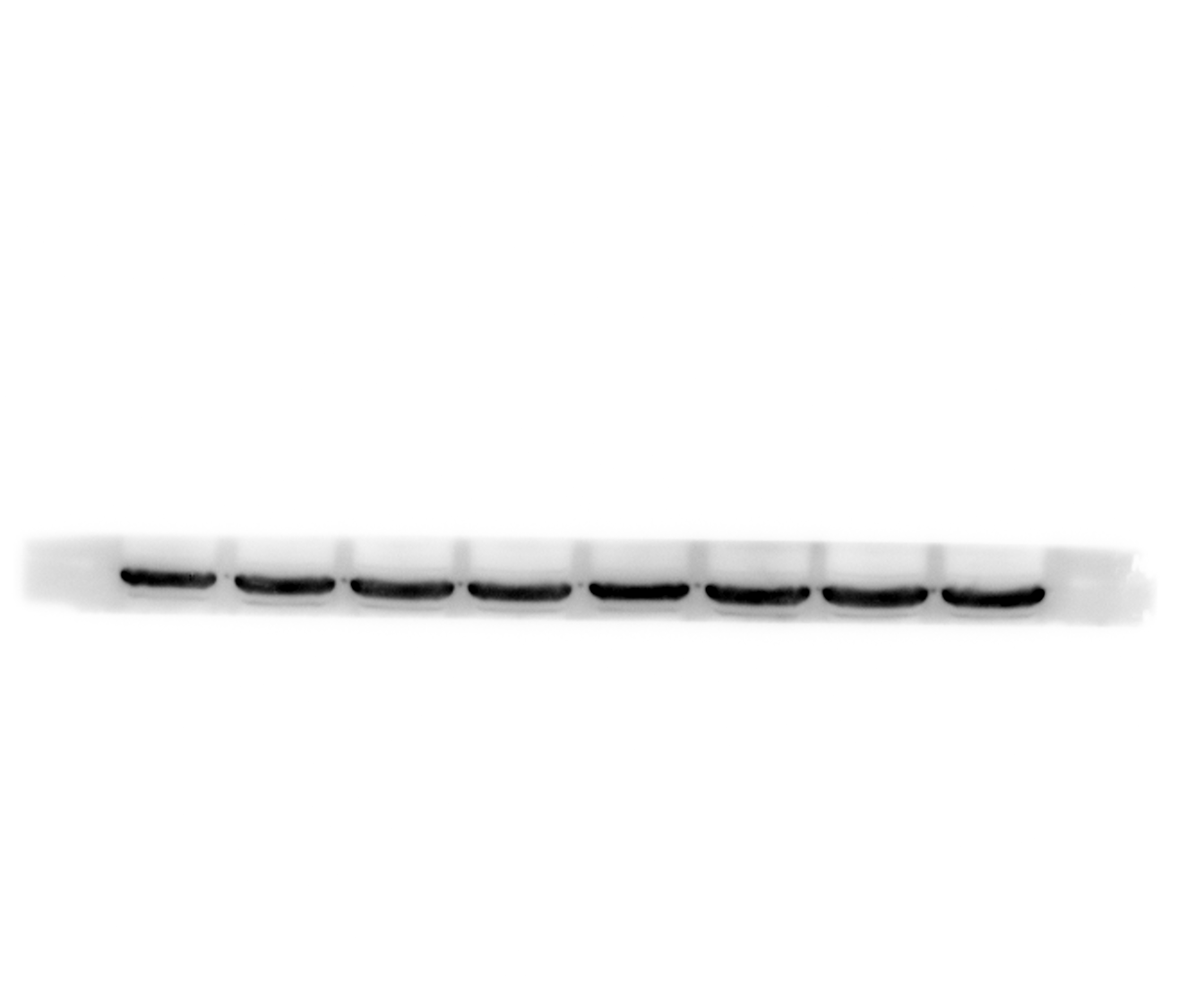

Supplement: Supplementary file 2 [file SupplementaryFile2.zip › WB数据/116/116-wb/actin/116-actin.tif]

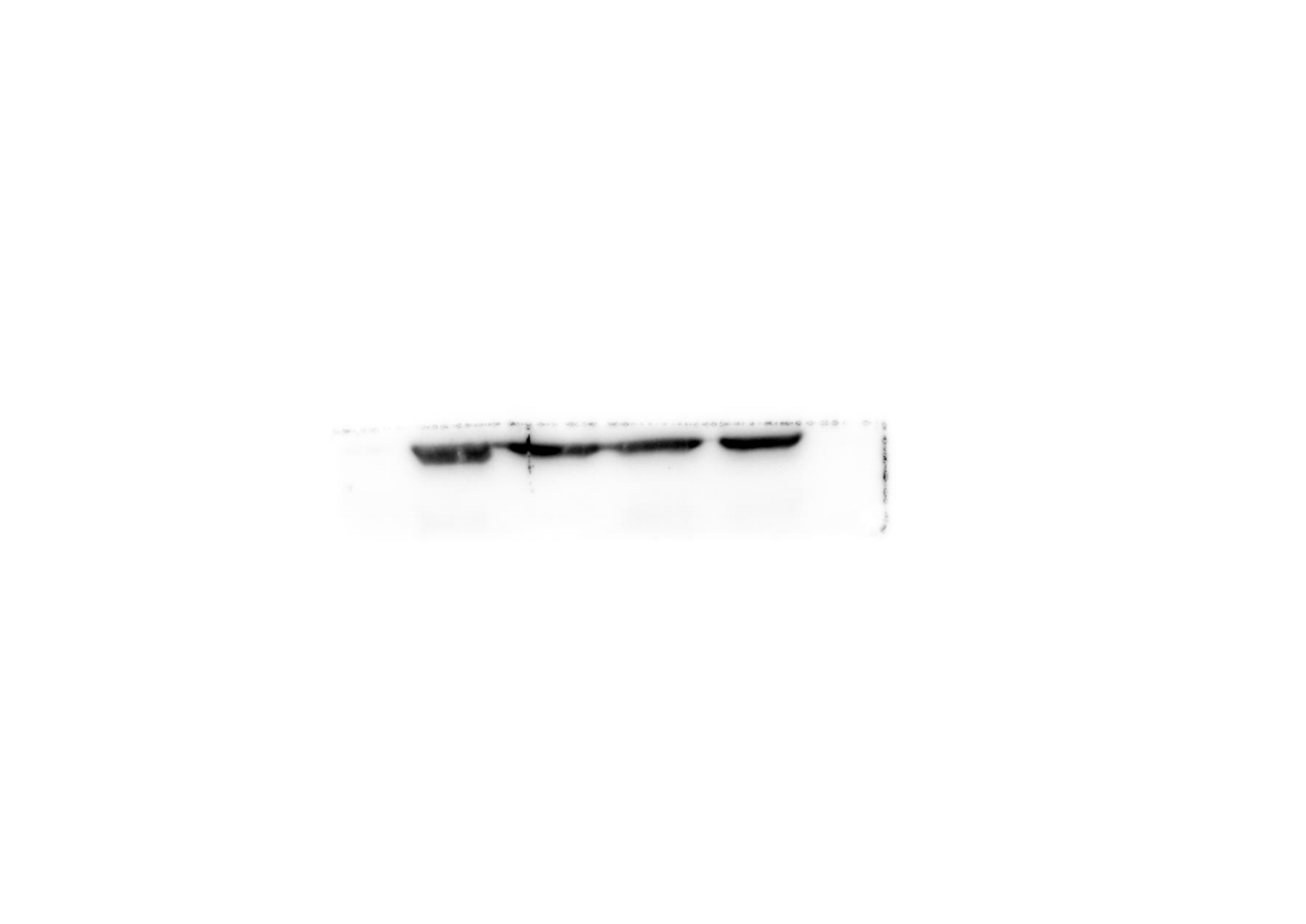

Supplement: Supplementary file 2 [file SupplementaryFile2.zip › WB数据/116/116-wb/actin/116-actin``_00.tif]

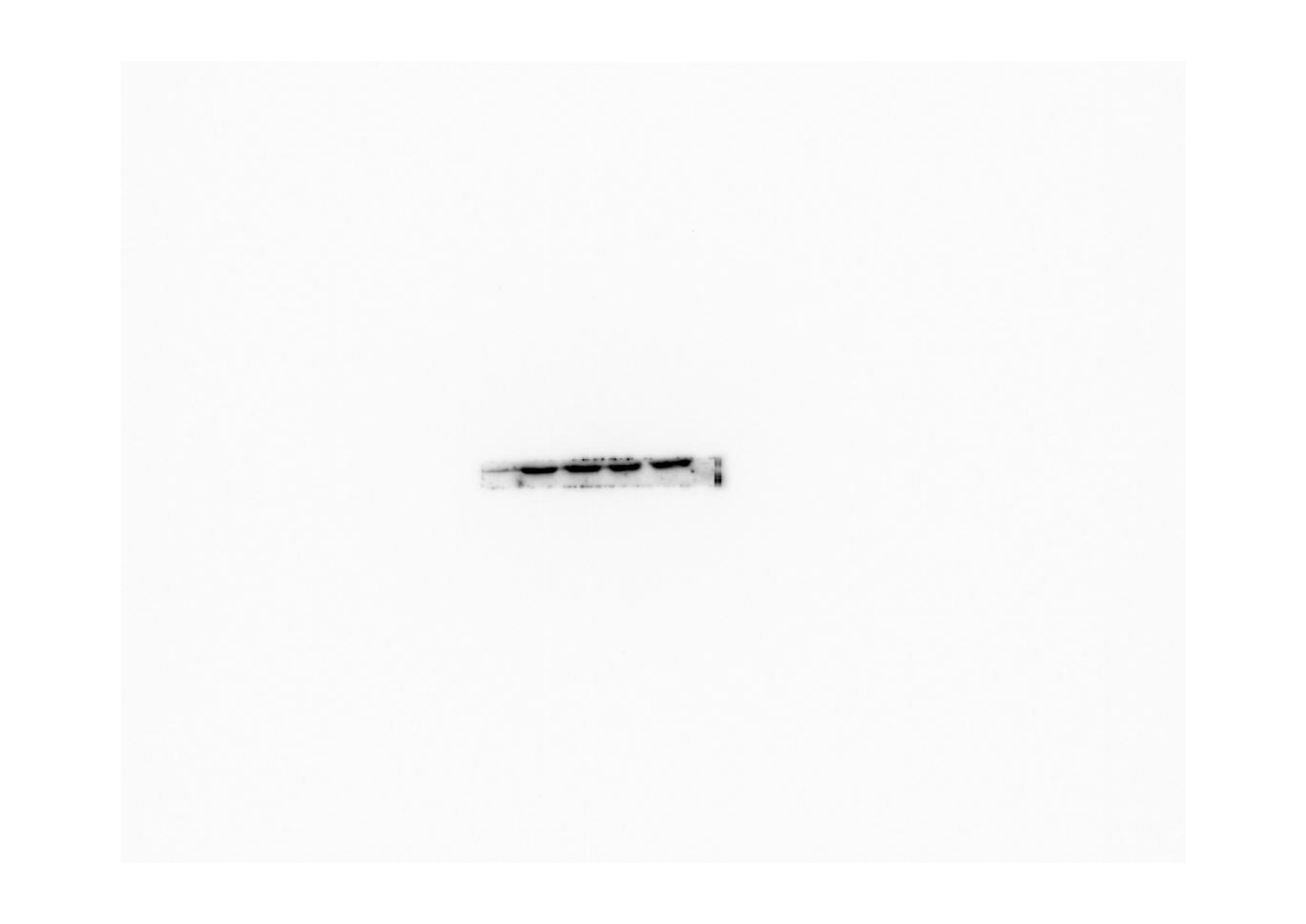

Supplement: Supplementary file 2 [file SupplementaryFile2.zip › WB数据/116/116-wb/actin/actin-----_00.tif]

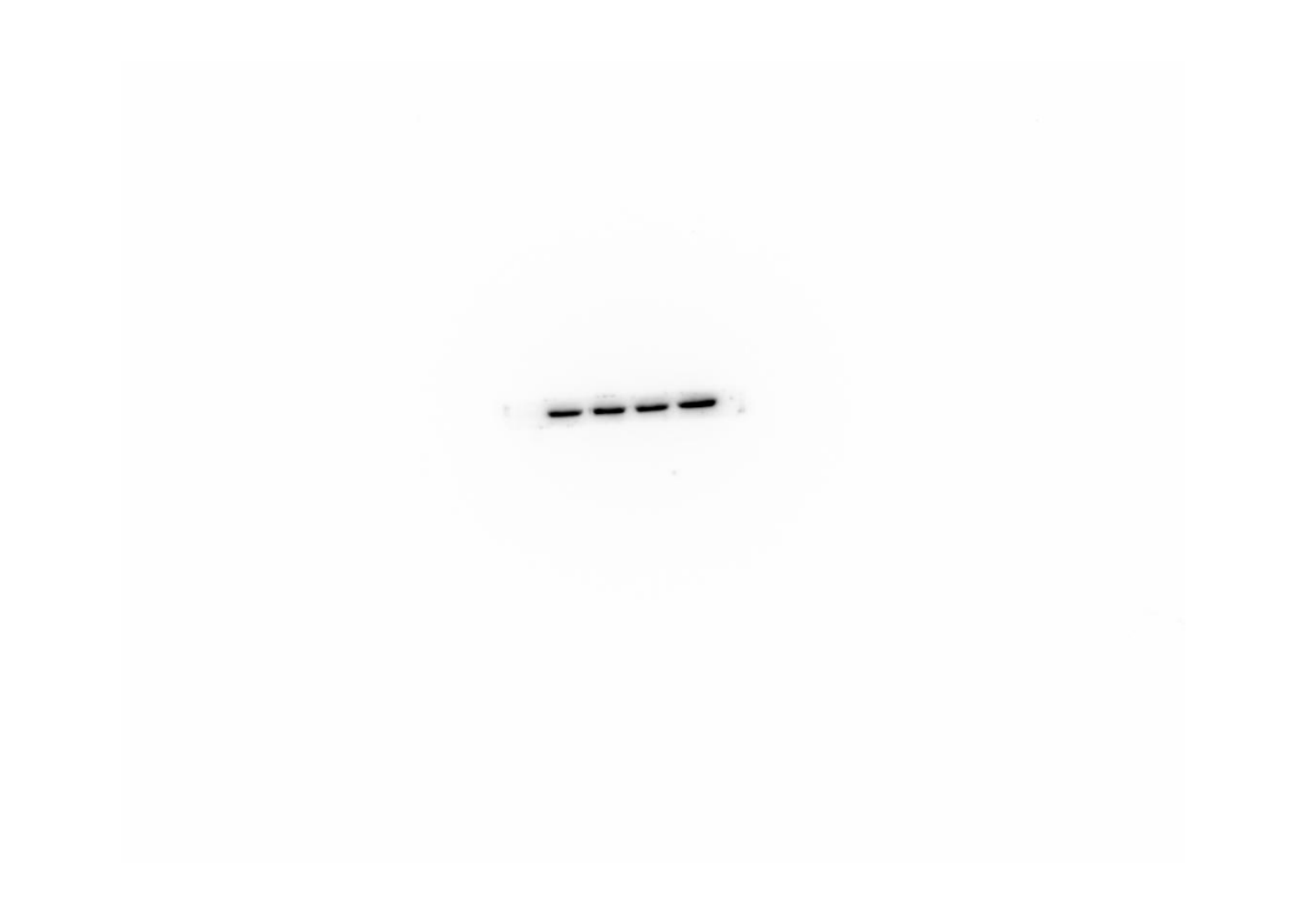

Supplement: Supplementary file 2 [file SupplementaryFile2.zip › WB数据/116/116-wb/actin/actin-_00.tif]

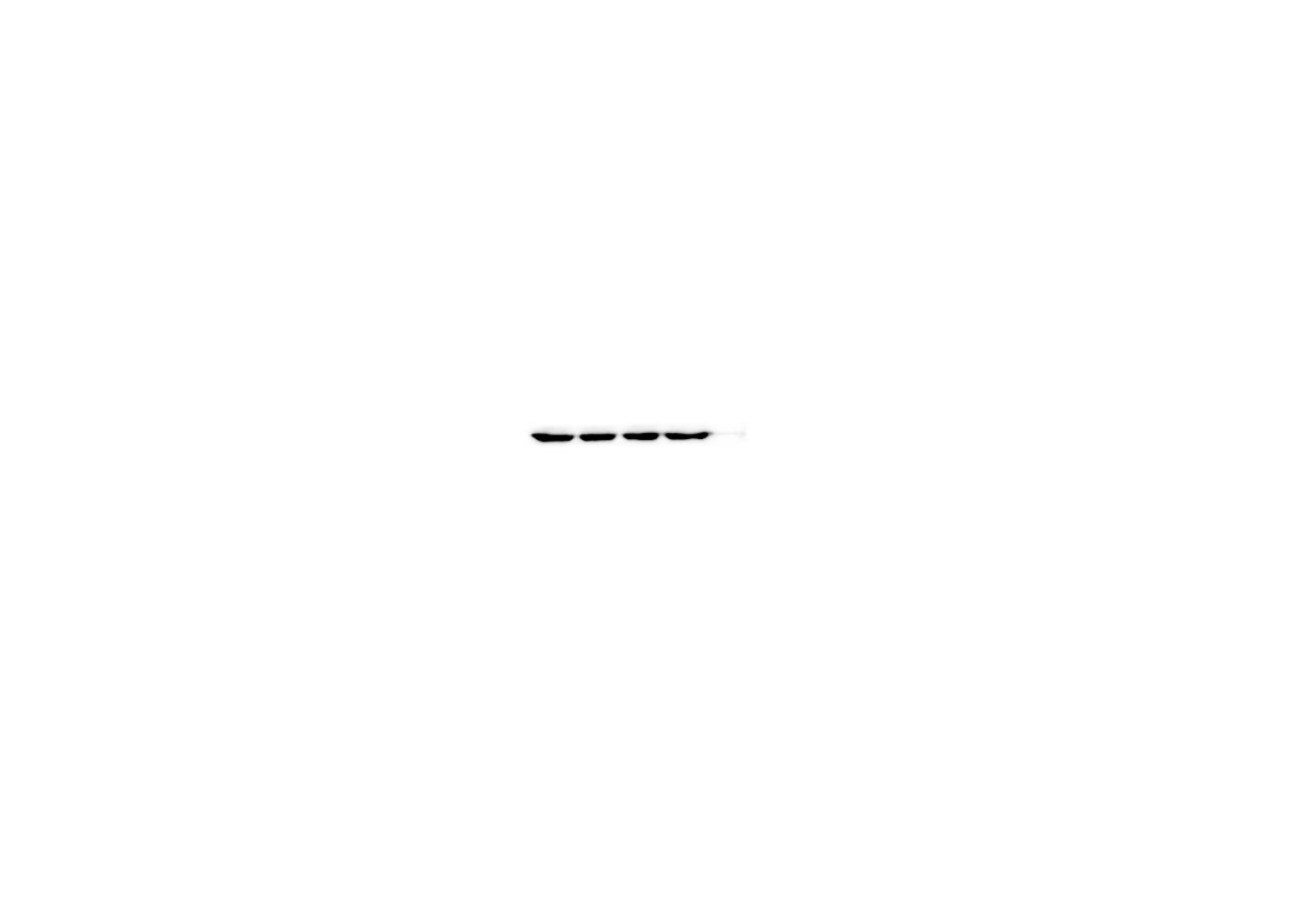

Supplement: Supplementary file 2 [file SupplementaryFile2.zip › WB数据/116/116-wb/actin/actin-`_00.tif]

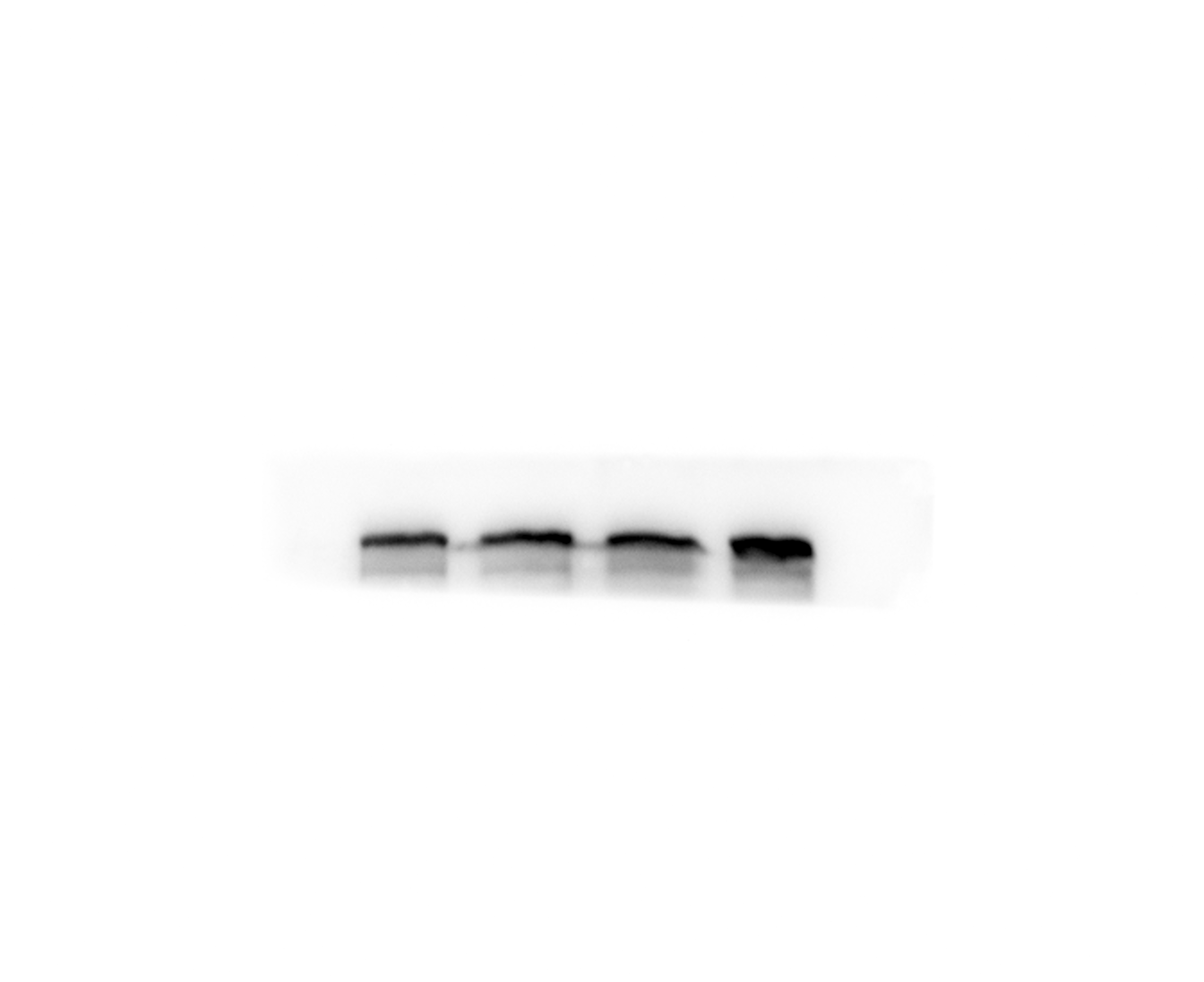

Supplement: Supplementary file 2 [file SupplementaryFile2.zip › WB数据/116/116-wb/e/116-e.tif]

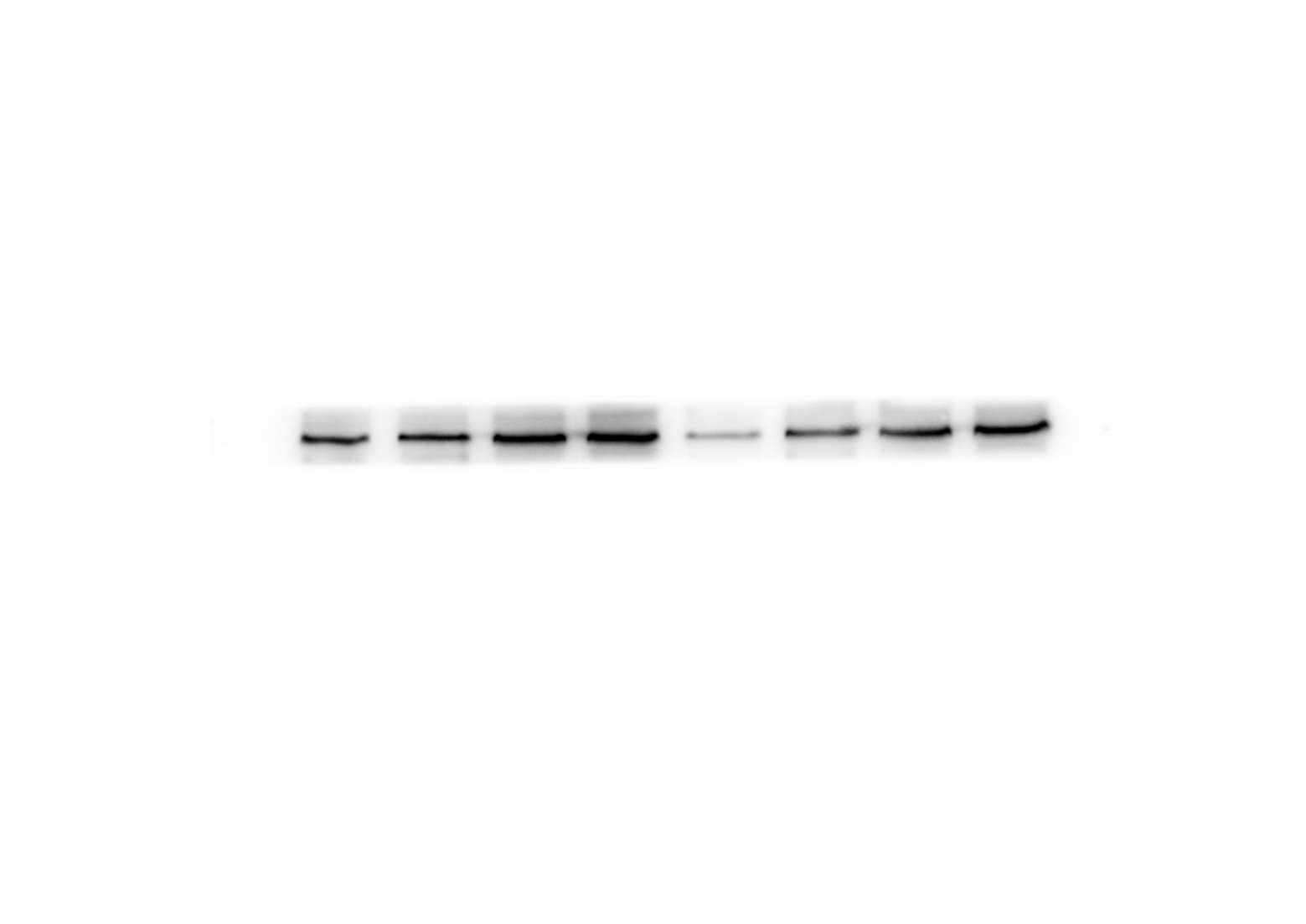

Supplement: Supplementary file 2 [file SupplementaryFile2.zip › WB数据/116/116-wb/e/116-e_00.tif]

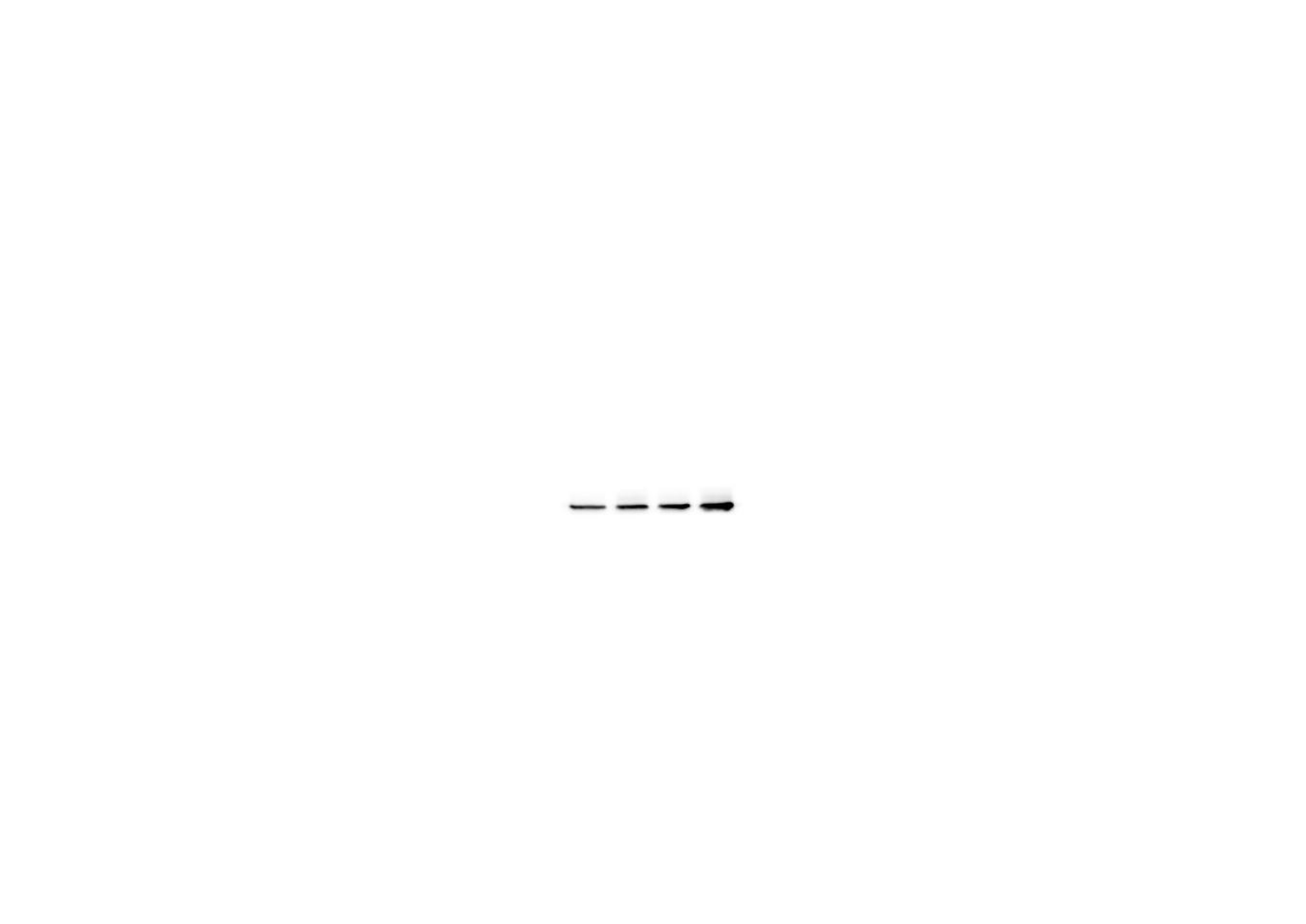

Supplement: Supplementary file 2 [file SupplementaryFile2.zip › WB数据/116/116-wb/e/e``_00.tif]

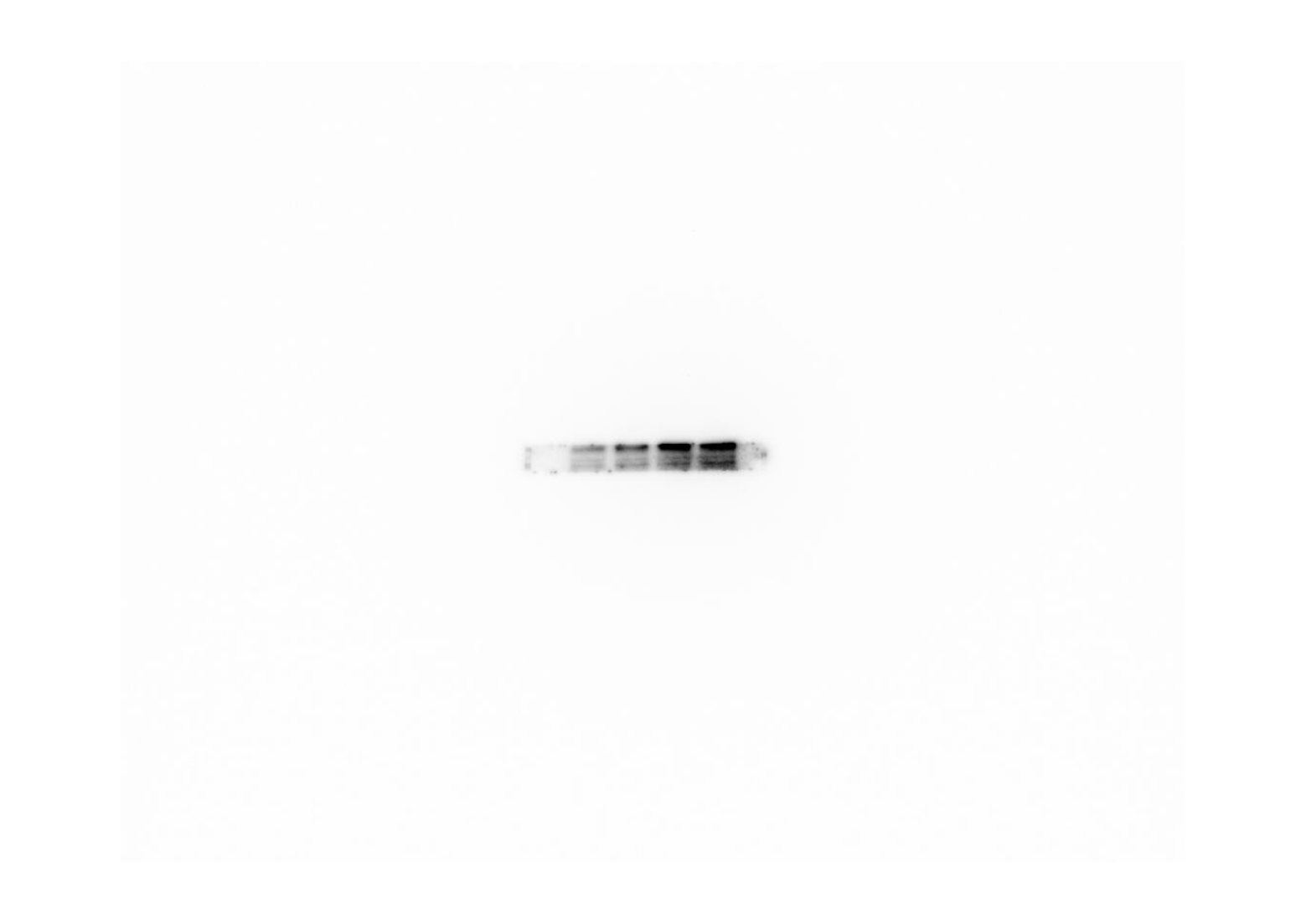

Supplement: Supplementary file 2 [file SupplementaryFile2.zip › WB数据/116/116-wb/e/e````_00.tif]

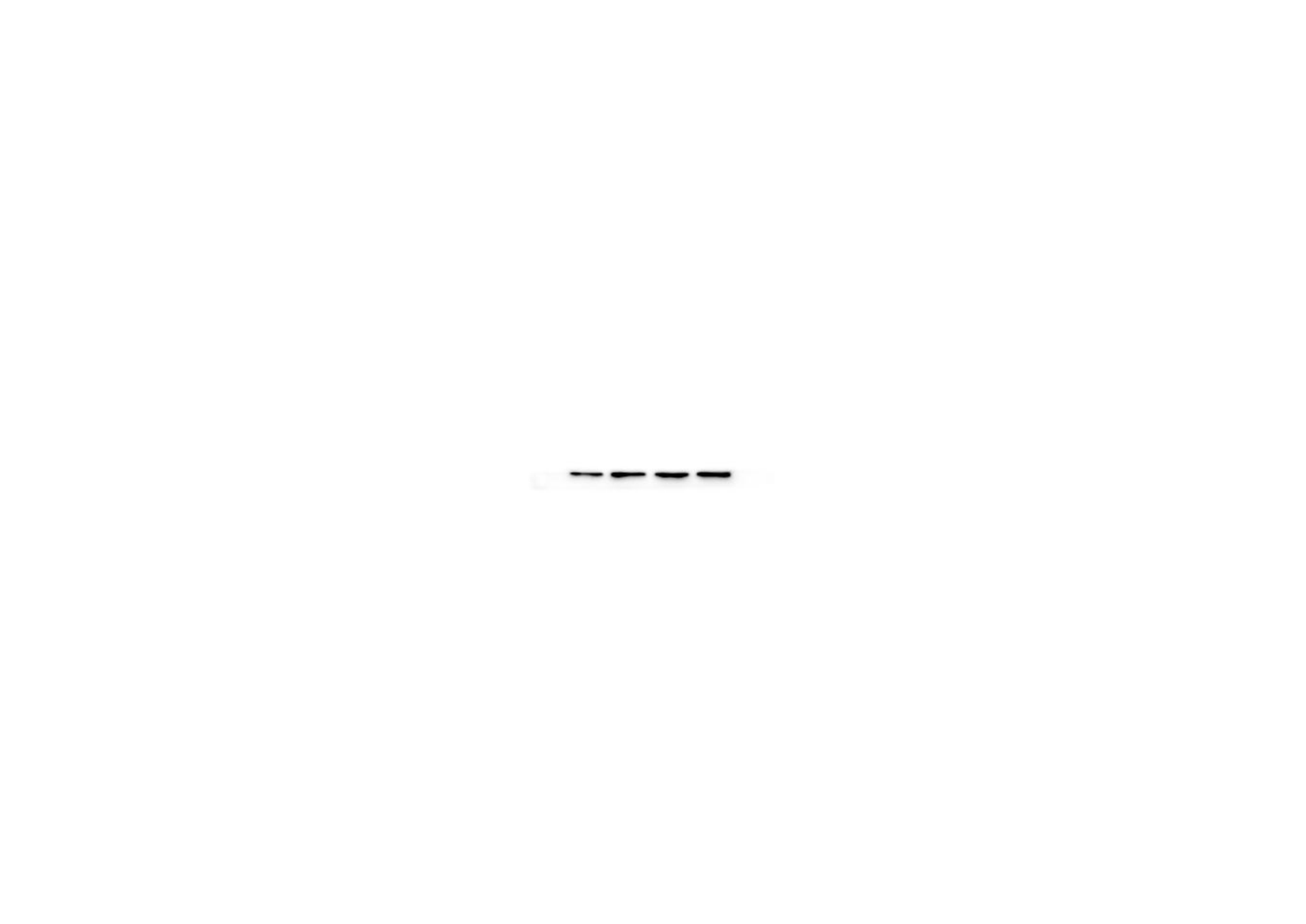

Supplement: Supplementary file 2 [file SupplementaryFile2.zip › WB数据/116/116-wb/e/e钙粘_00.tif]

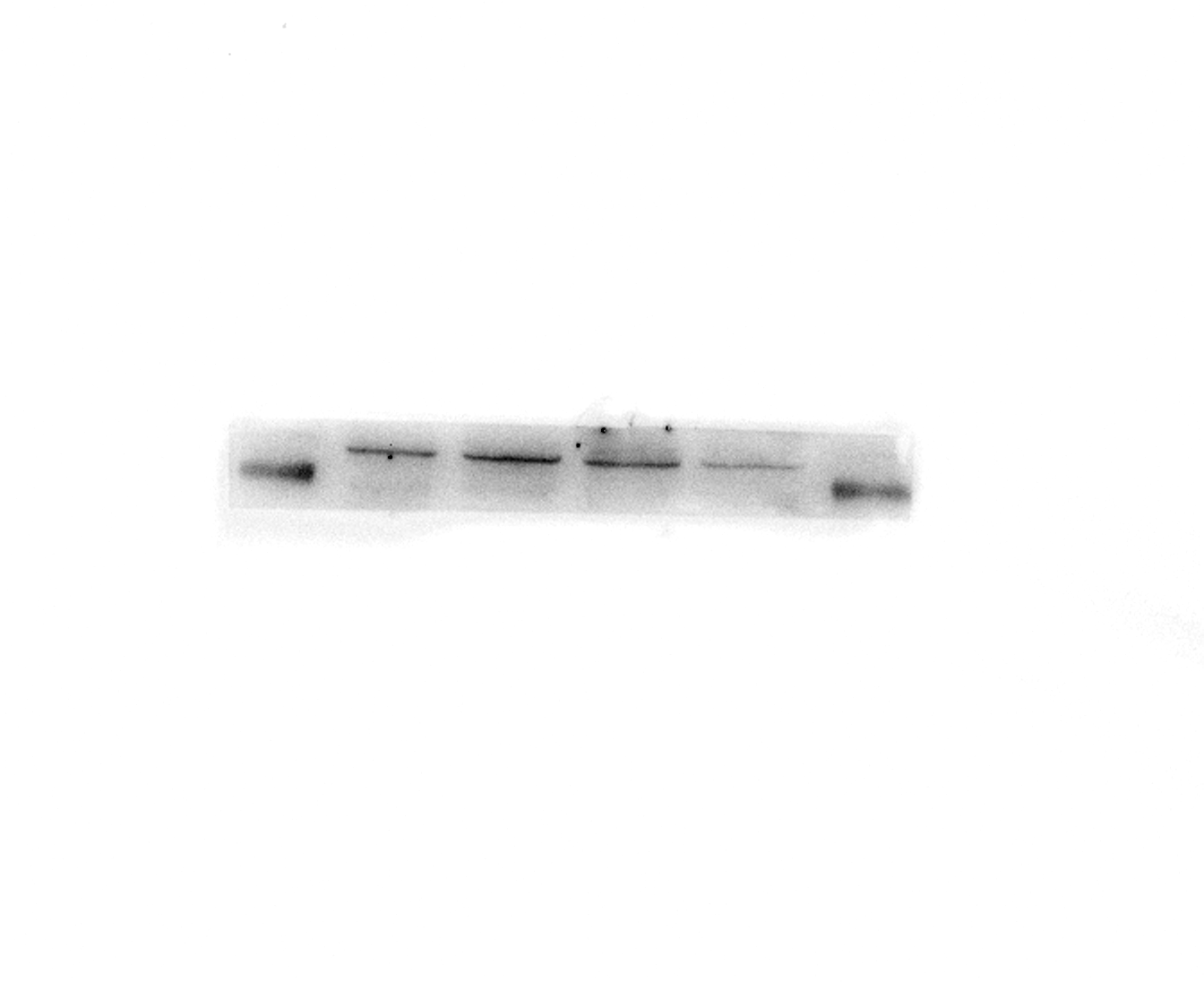

Supplement: Supplementary file 2 [file SupplementaryFile2.zip › WB数据/116/116-wb/mmp9/116-MMP9.tif]

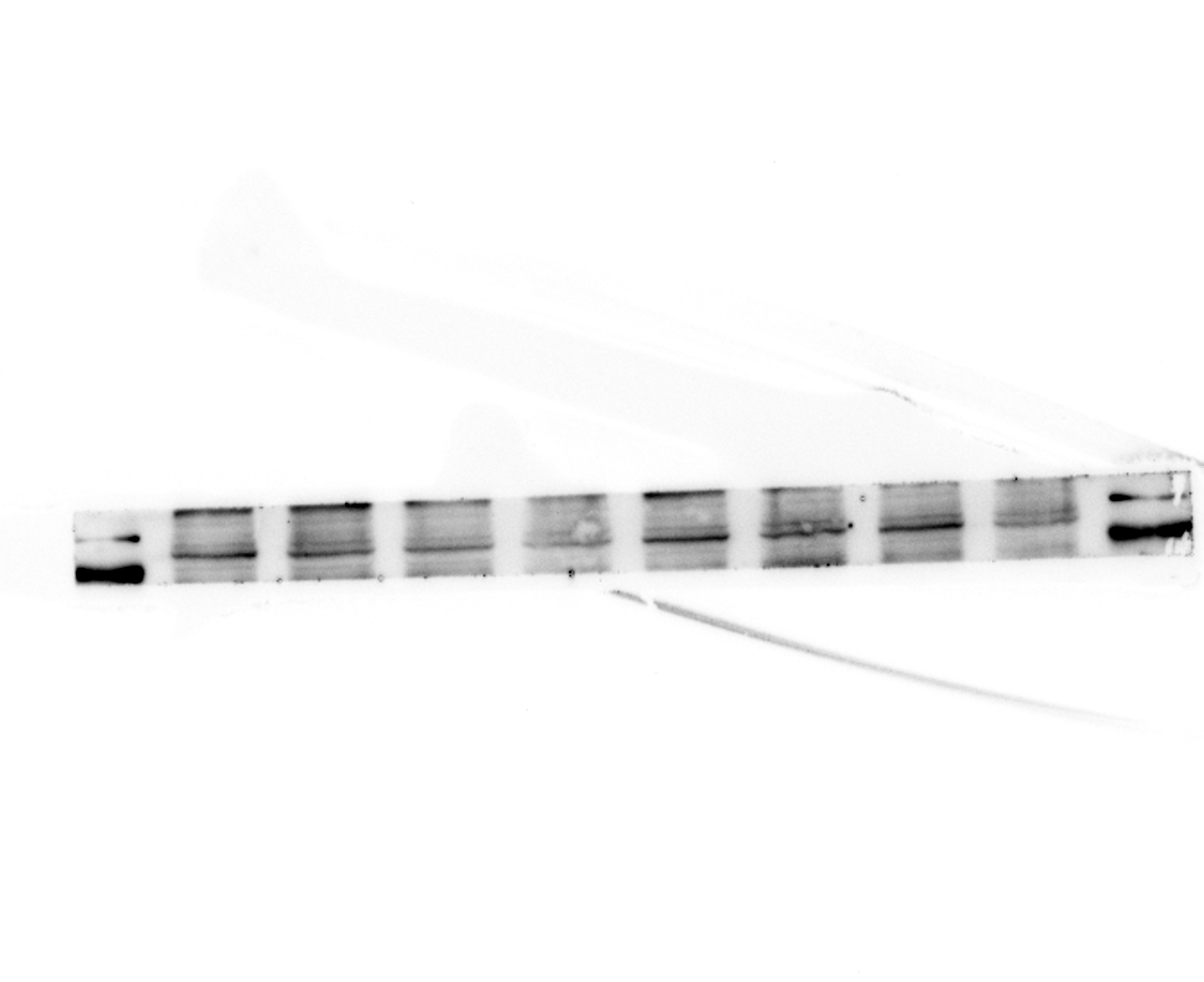

Supplement: Supplementary file 2 [file SupplementaryFile2.zip › WB数据/116/116-wb/mmp9/116-mmp9``.tif]

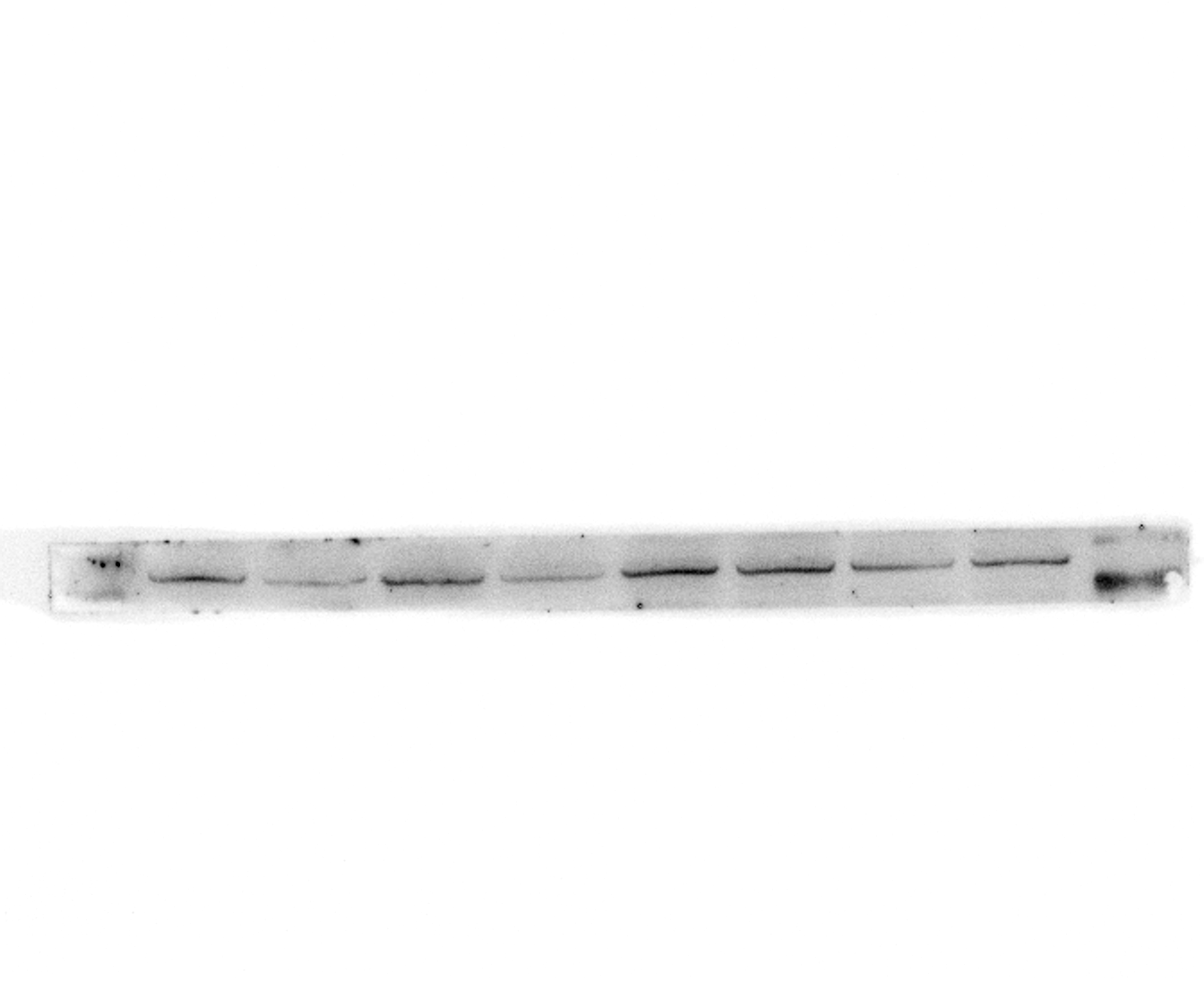

Supplement: Supplementary file 2 [file SupplementaryFile2.zip › WB数据/116/116-wb/mmp9/mmp9-.tif]

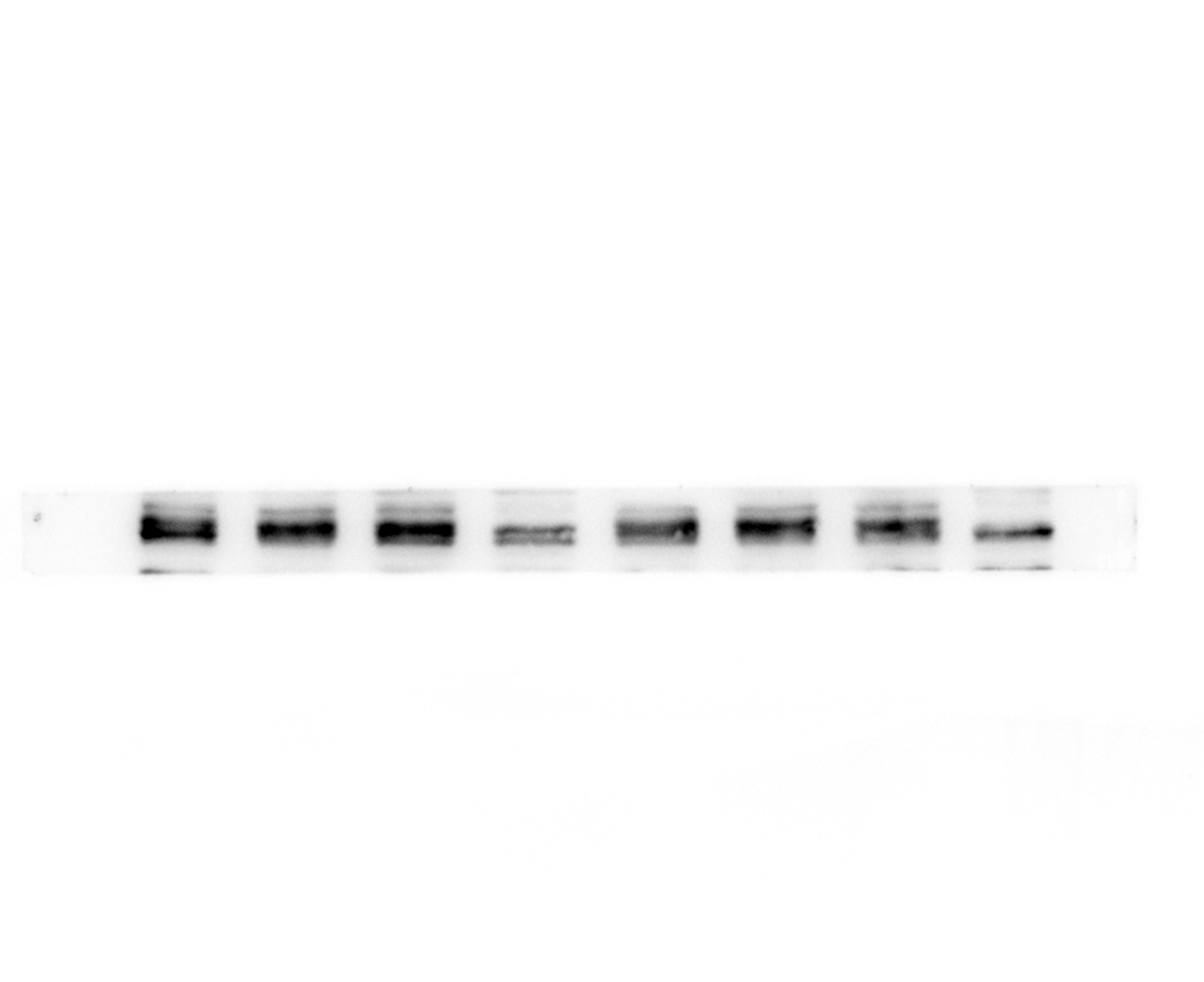

Supplement: Supplementary file 2 [file SupplementaryFile2.zip › WB数据/116/116-wb/snail/116-snail---.tif]

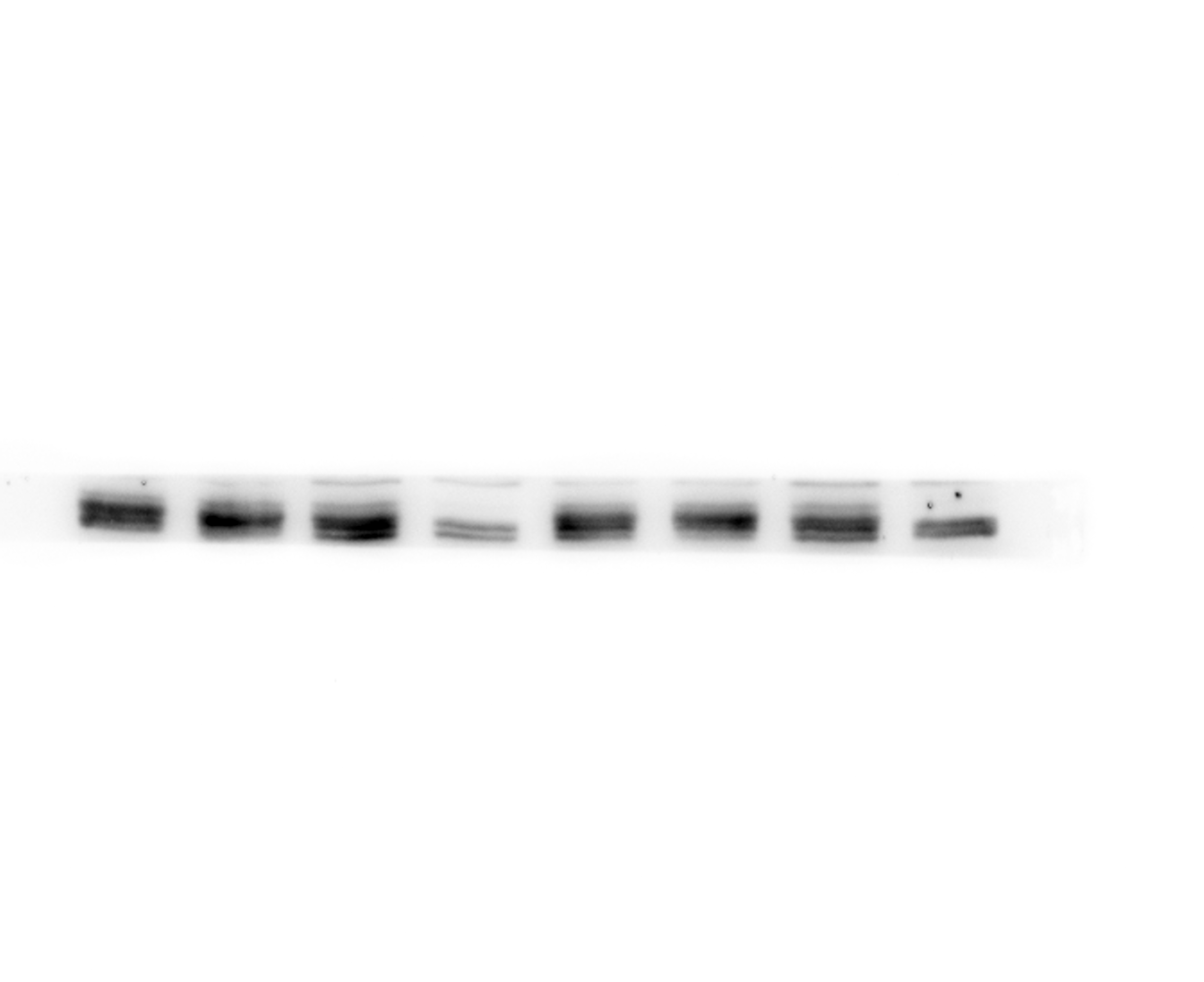

Supplement: Supplementary file 2 [file SupplementaryFile2.zip › WB数据/116/116-wb/snail/116-snail.tif]

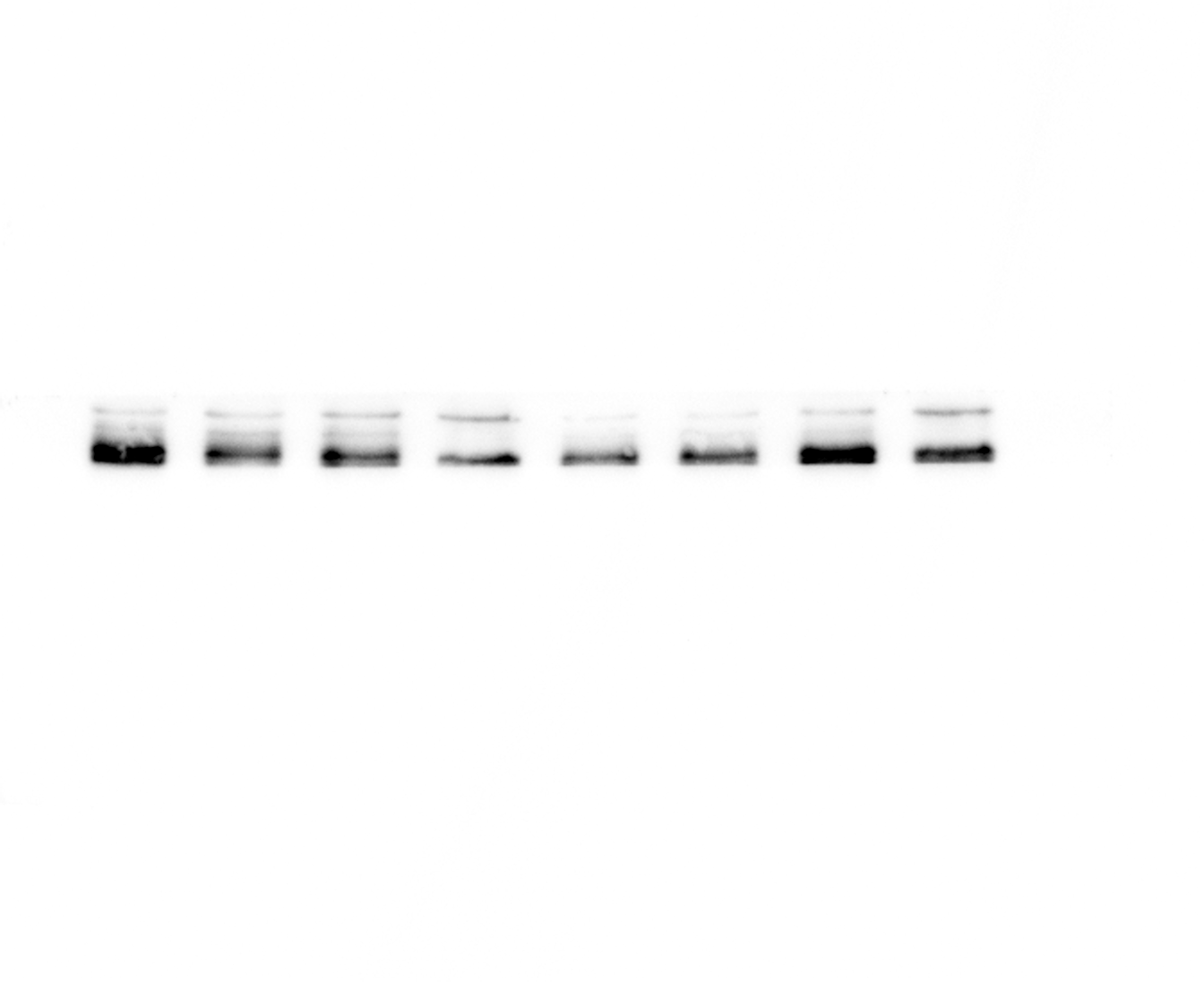

Supplement: Supplementary file 2 [file SupplementaryFile2.zip › WB数据/116/116-wb/snail/116-snail``.tif]

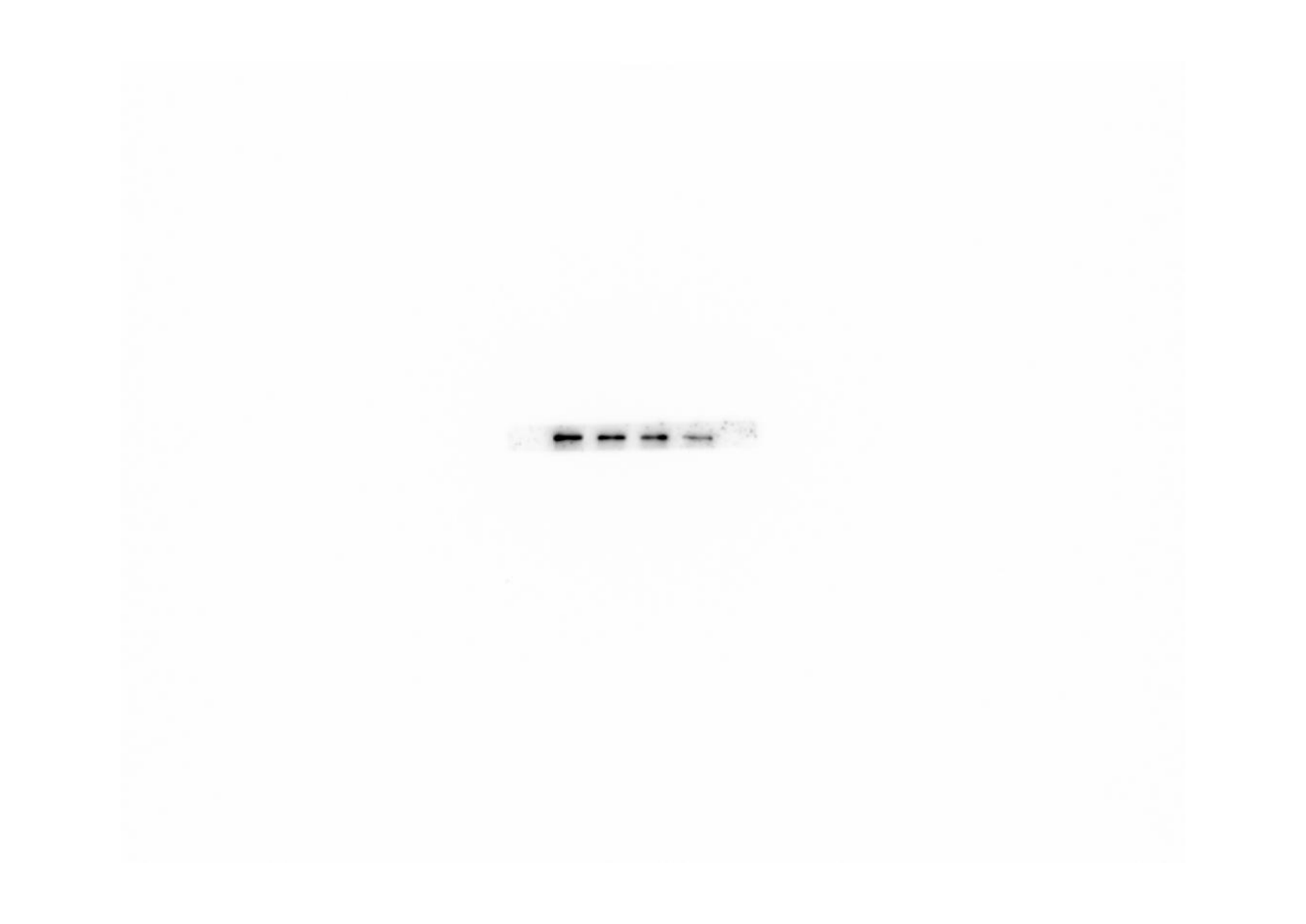

Supplement: Supplementary file 2 [file SupplementaryFile2.zip › WB数据/116/116-wb/snail/snail-_00.tif]

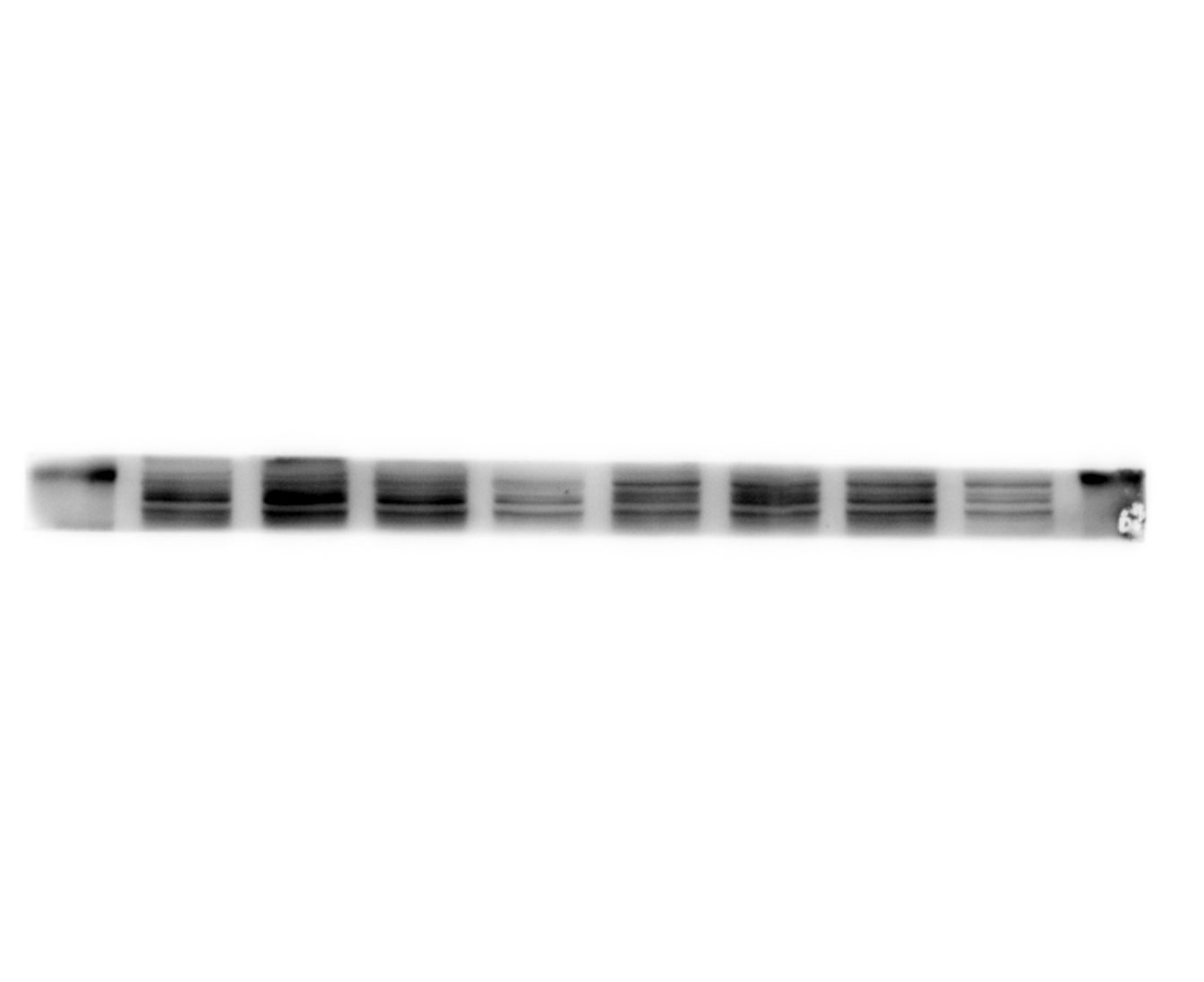

Supplement: Supplementary file 2 [file SupplementaryFile2.zip › WB数据/116/116-wb/srebp1/116--srebp1-68-.tif]

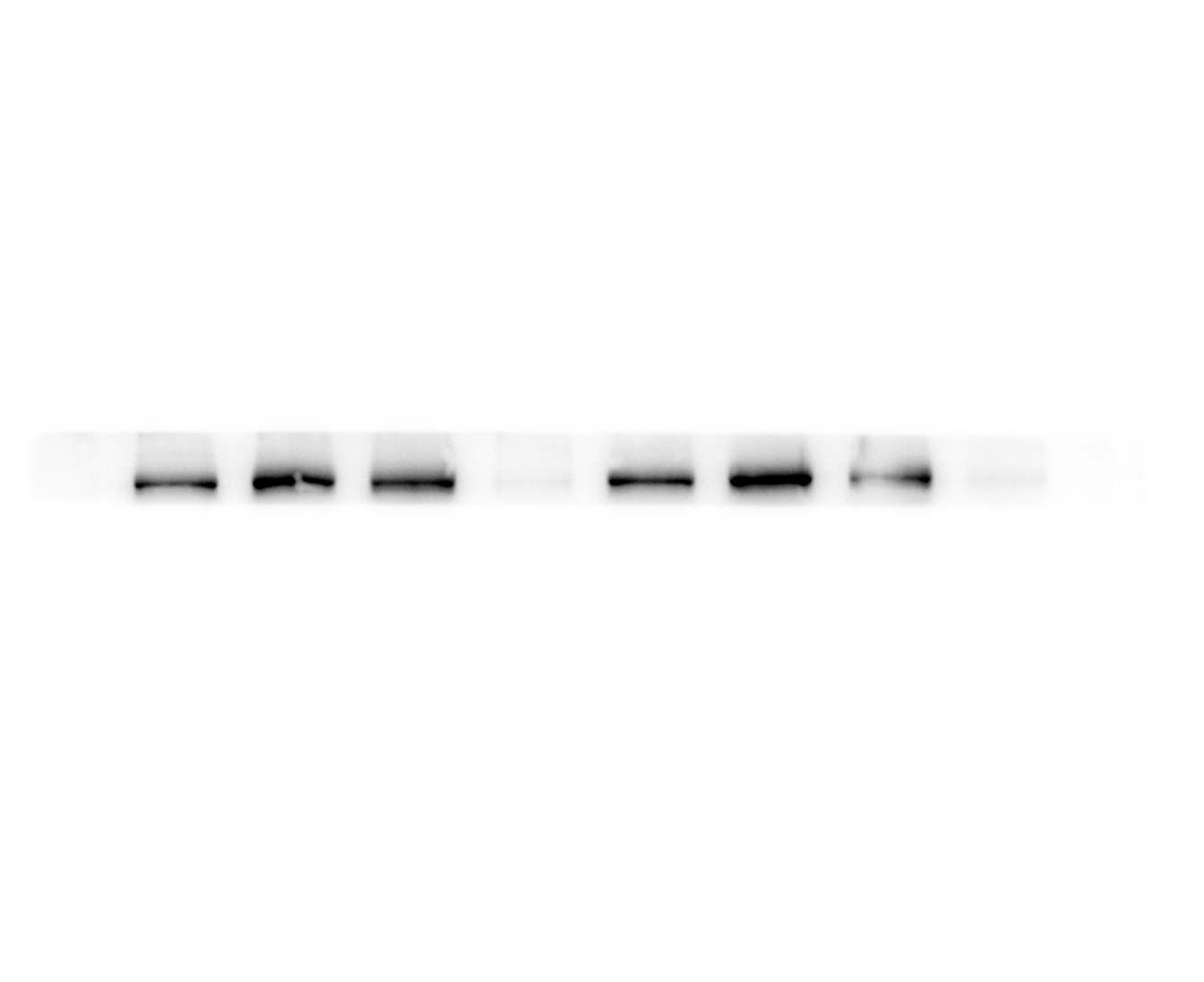

Supplement: Supplementary file 2 [file SupplementaryFile2.zip › WB数据/116/116-wb/srebp1/116-srebp1-68.tif]

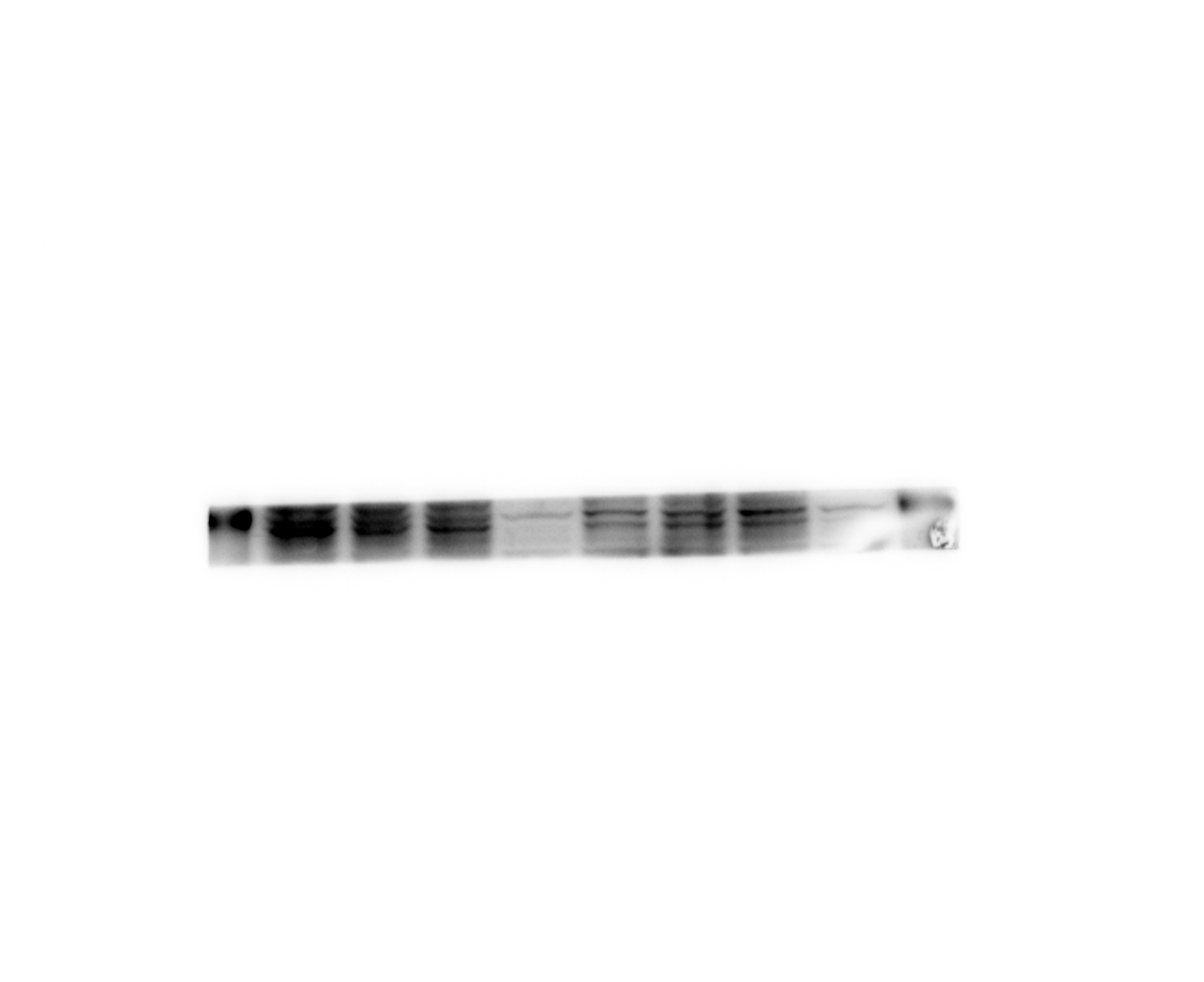

Supplement: Supplementary file 2 [file SupplementaryFile2.zip › WB数据/116/116-wb/srebp1/srebp1-68.tif]

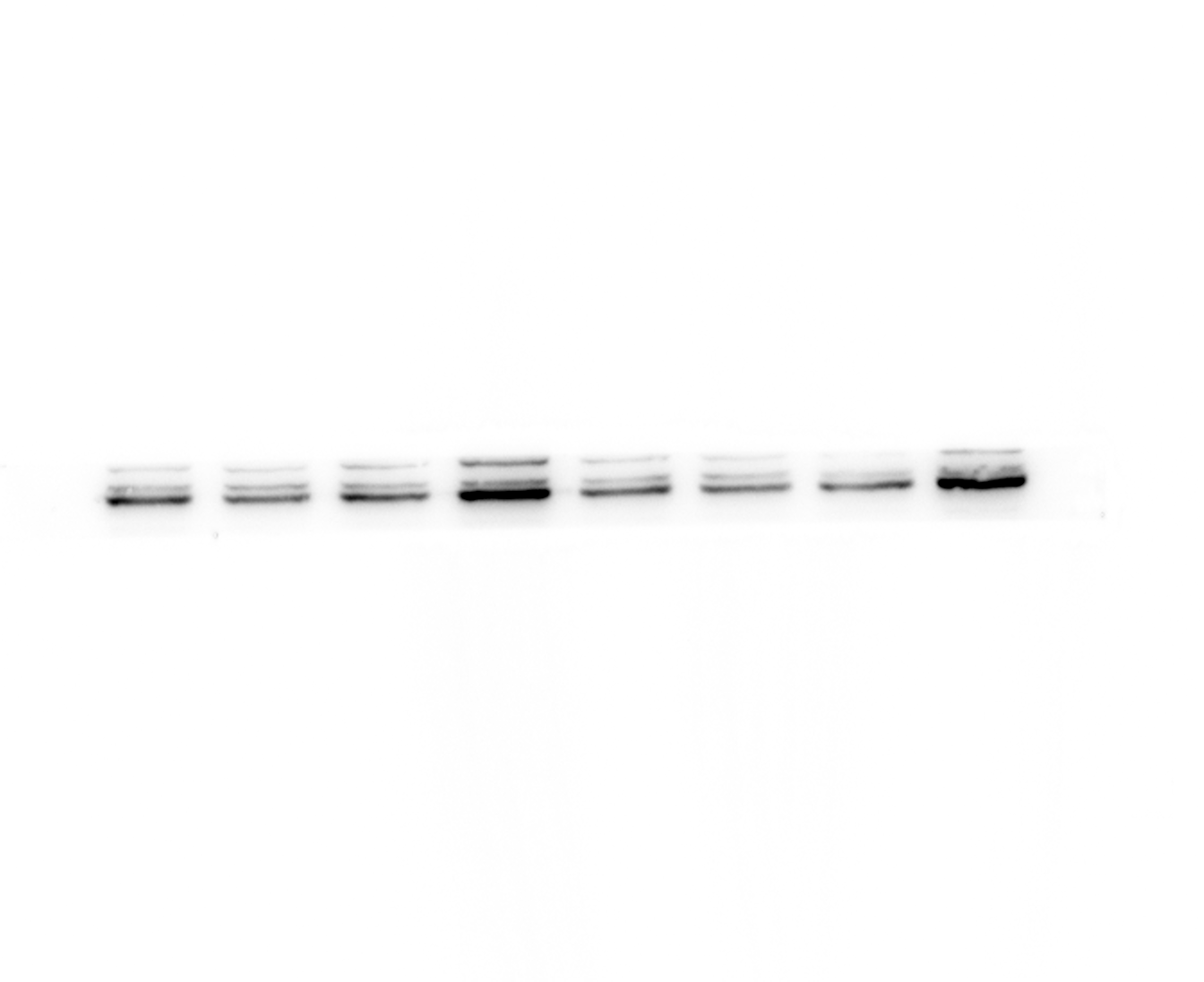

Supplement: Supplementary file 2 [file SupplementaryFile2.zip › WB数据/116/116-wb/zo1/116-zo1--.tif]

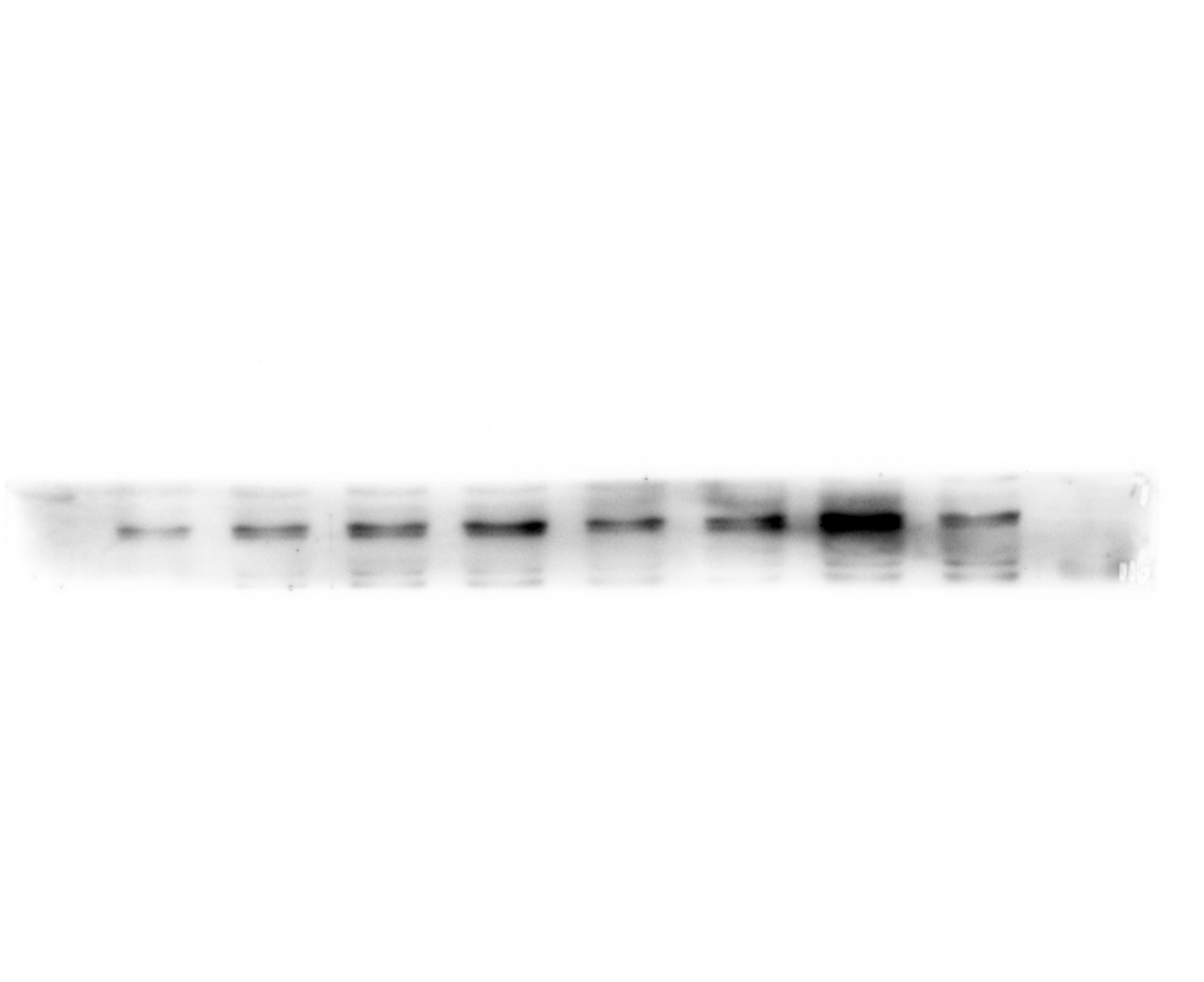

Supplement: Supplementary file 2 [file SupplementaryFile2.zip › WB数据/116/116-wb/zo1/116-zo1.tif]

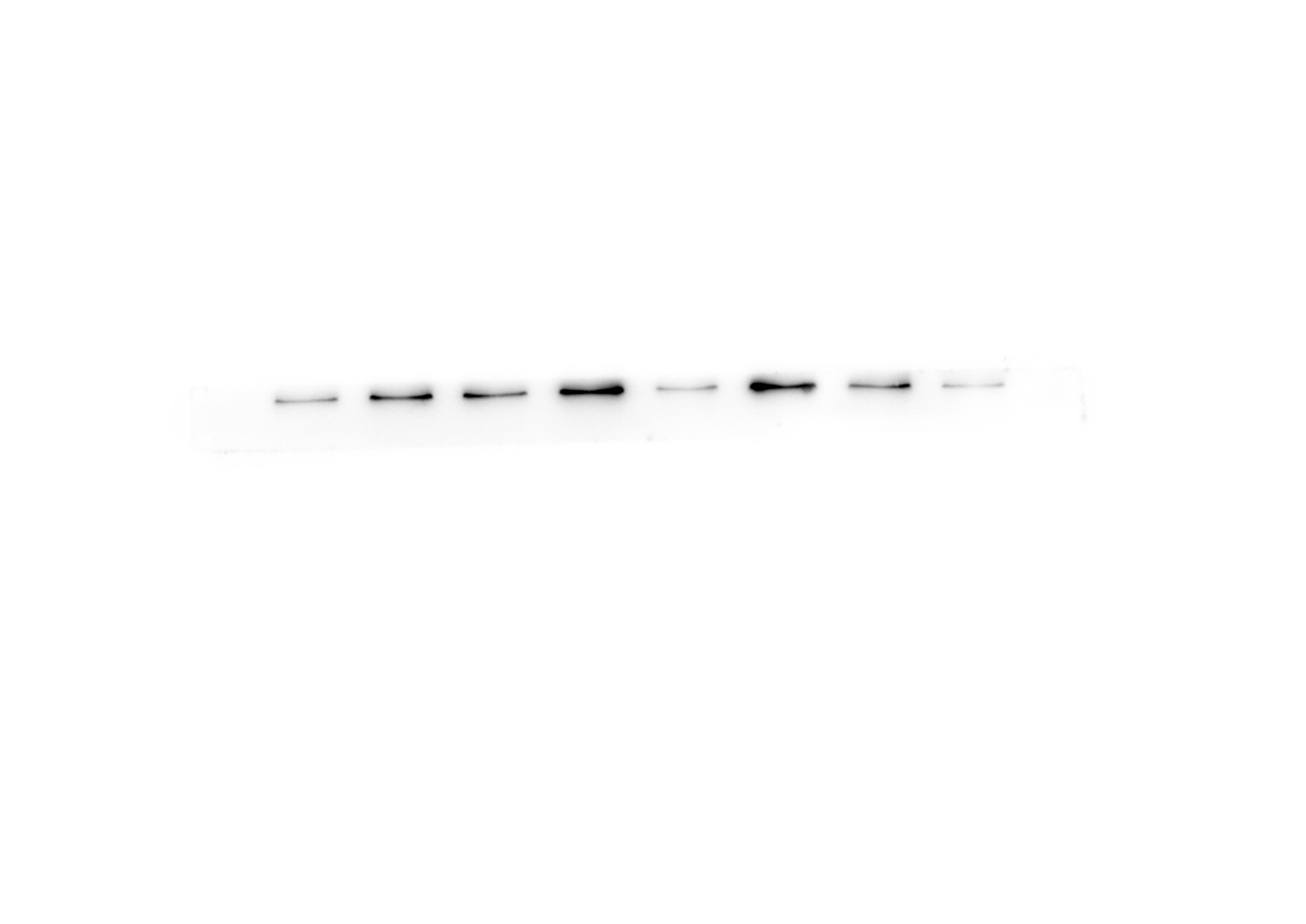

Supplement: Supplementary file 2 [file SupplementaryFile2.zip › WB数据/116/116-wb/zo1/zo1_00.tif]

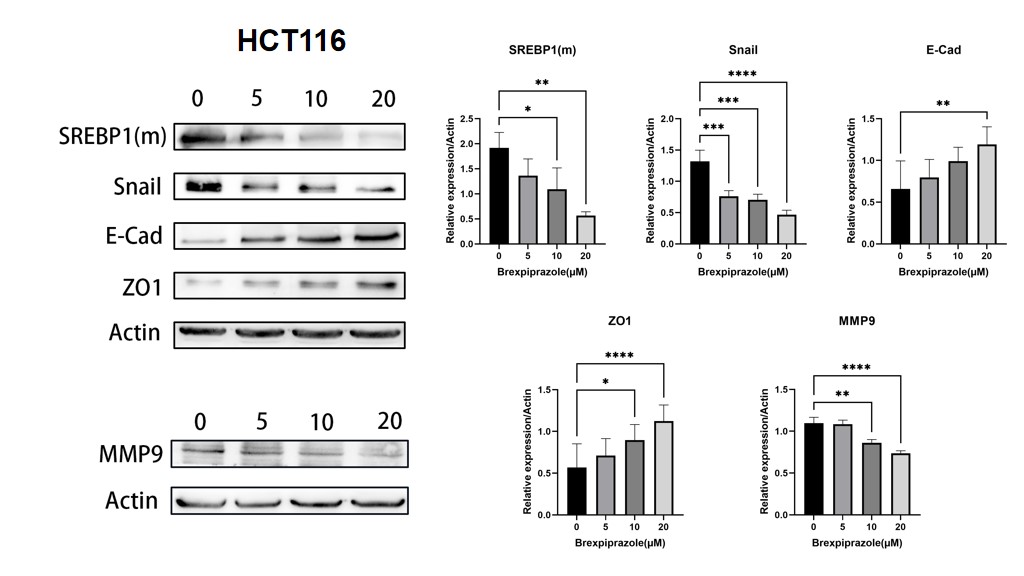

Supplement: Supplementary file 2 [file SupplementaryFile2.zip › WB数据/116/数据图/1.jpg]

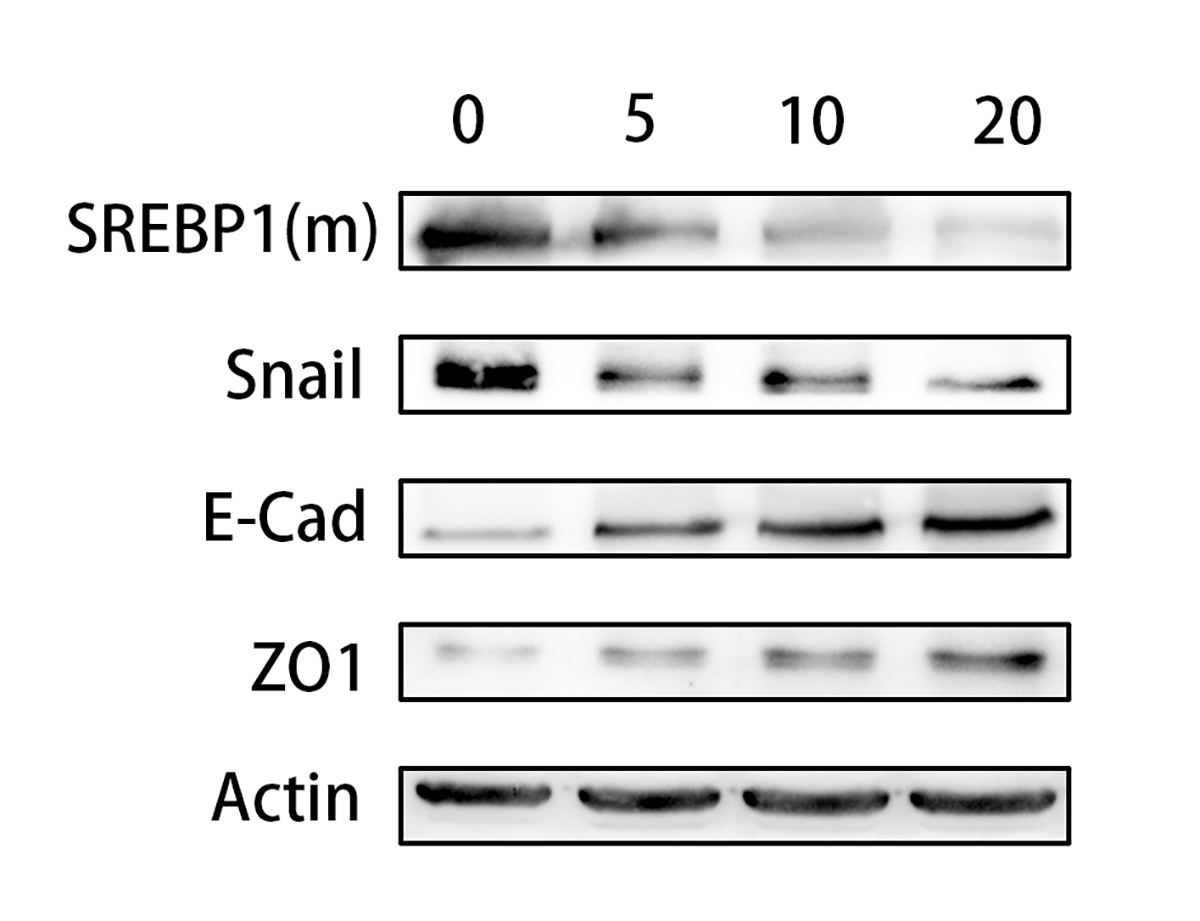

Supplement: Supplementary file 2 [file SupplementaryFile2.zip › WB数据/116/数据图/116-.png]

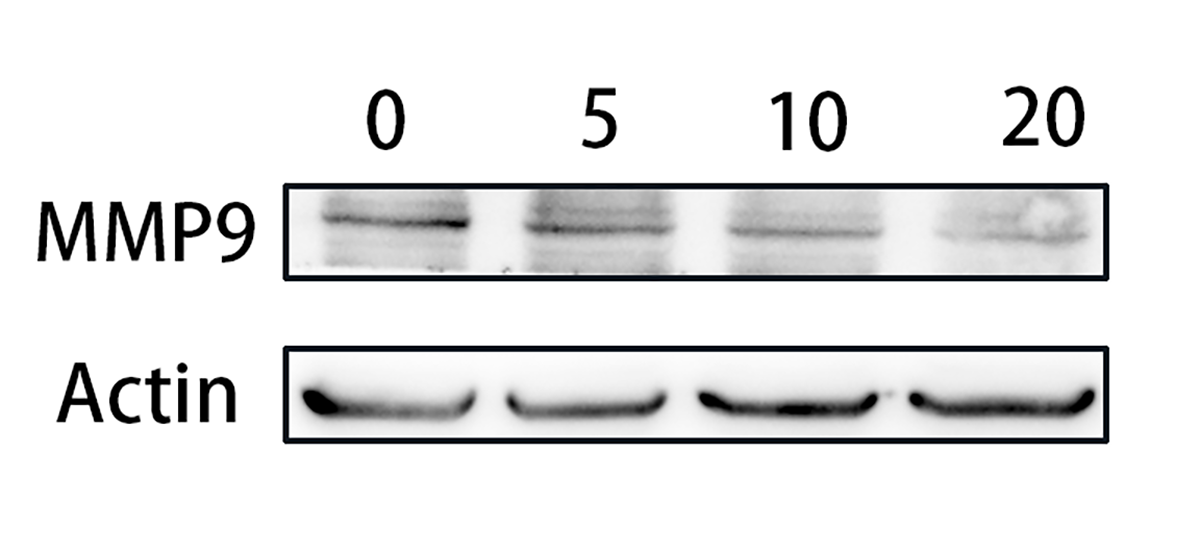

Supplement: Supplementary file 2 [file SupplementaryFile2.zip › WB数据/116/数据图/116-MMP9-.png]

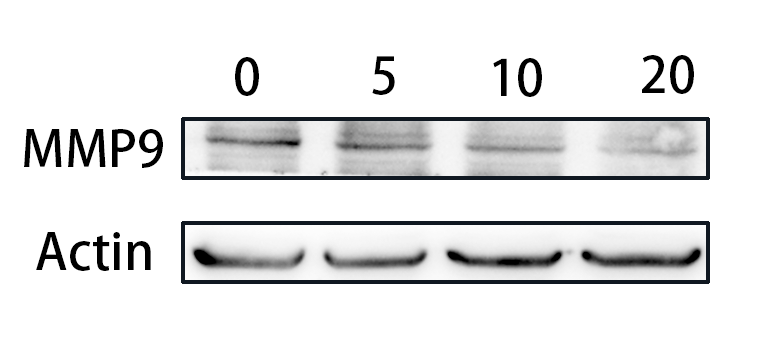

Supplement: Supplementary file 2 [file SupplementaryFile2.zip › WB数据/116/数据图/116-MMP9.png]

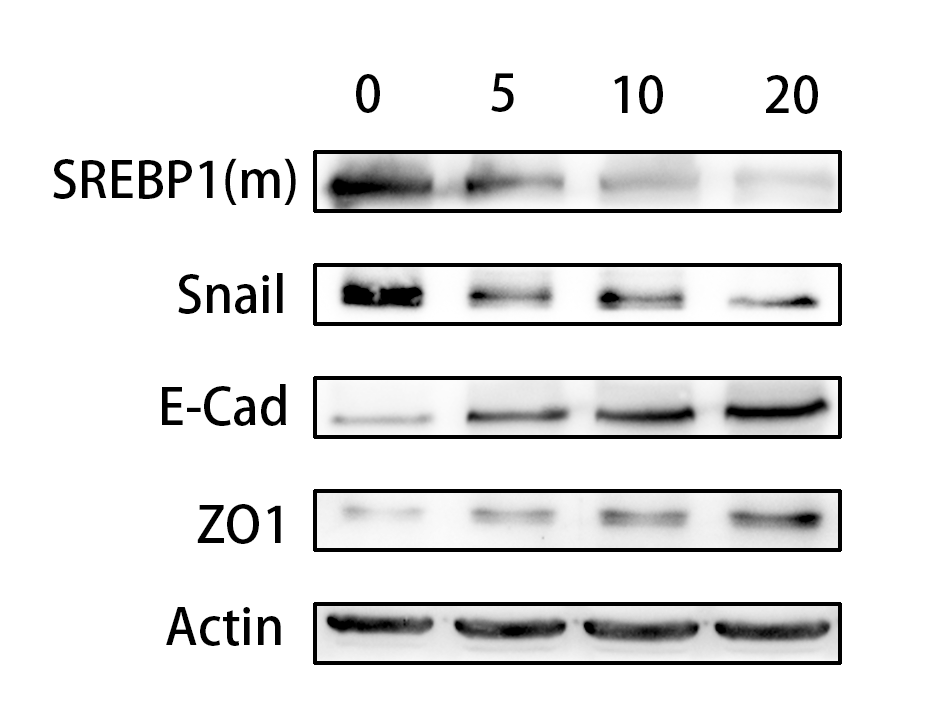

Supplement: Supplementary file 2 [file SupplementaryFile2.zip › WB数据/116/数据图/116.png]

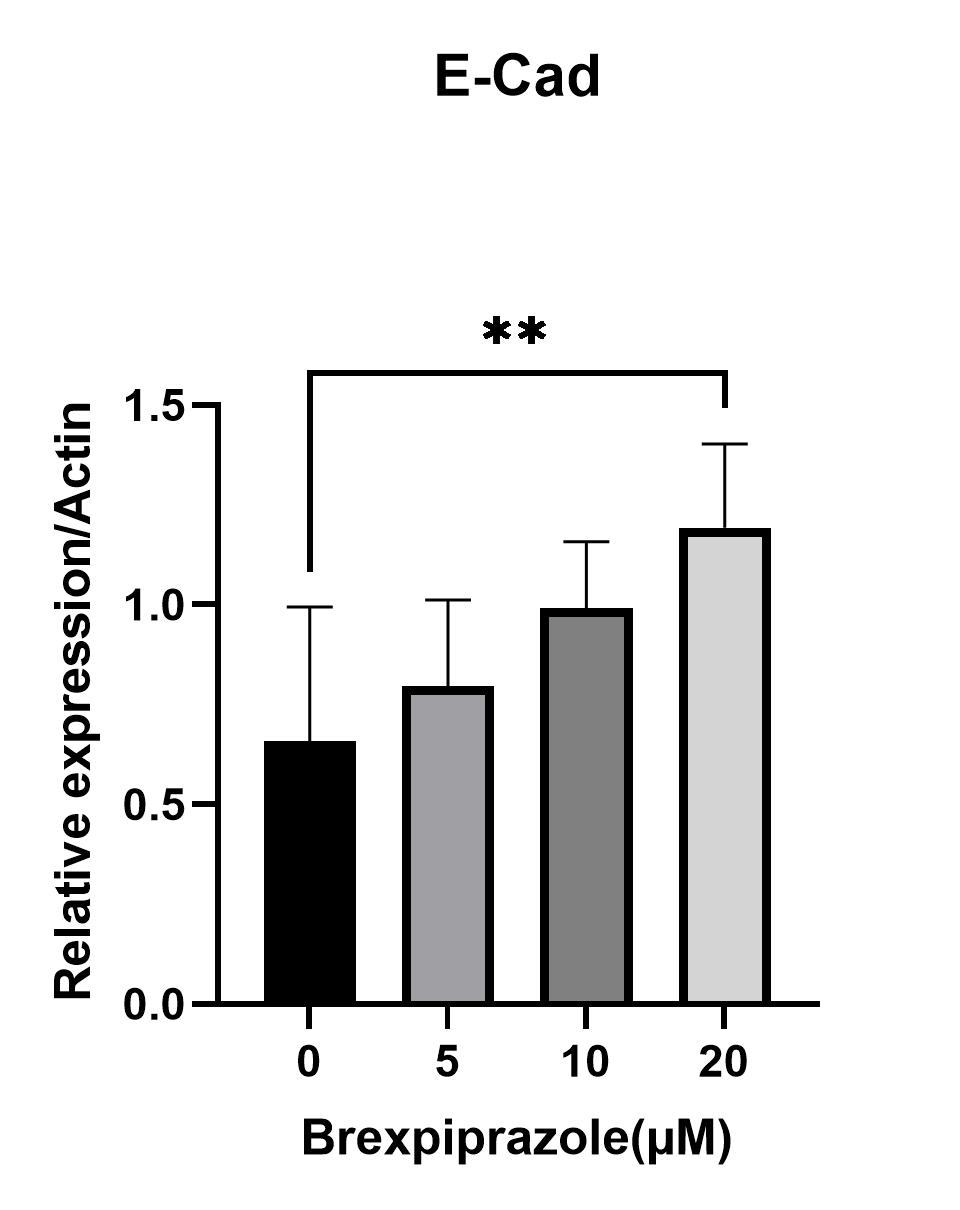

Supplement: Supplementary file 2 [file SupplementaryFile2.zip › WB数据/116/数据图/E.tif]

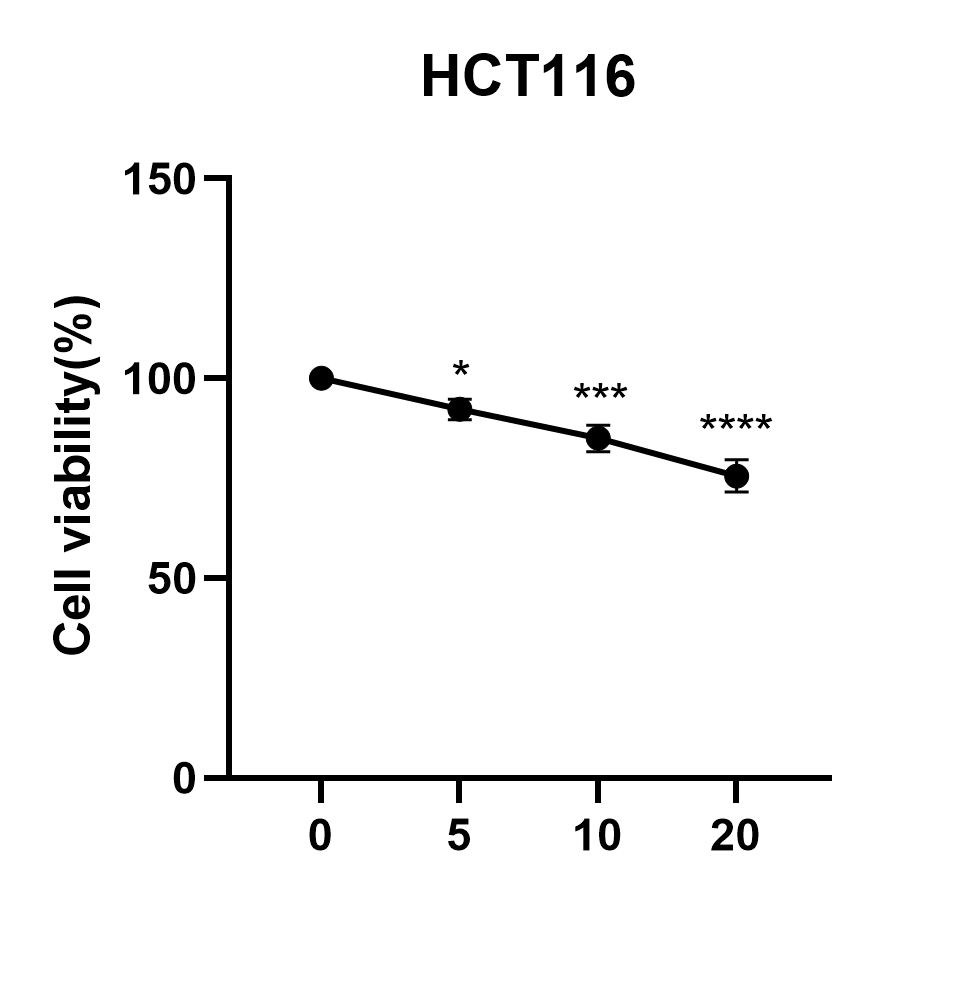

Supplement: Supplementary file 2 [file SupplementaryFile2.zip › WB数据/116/数据图/HCT116-CCK8.tif]

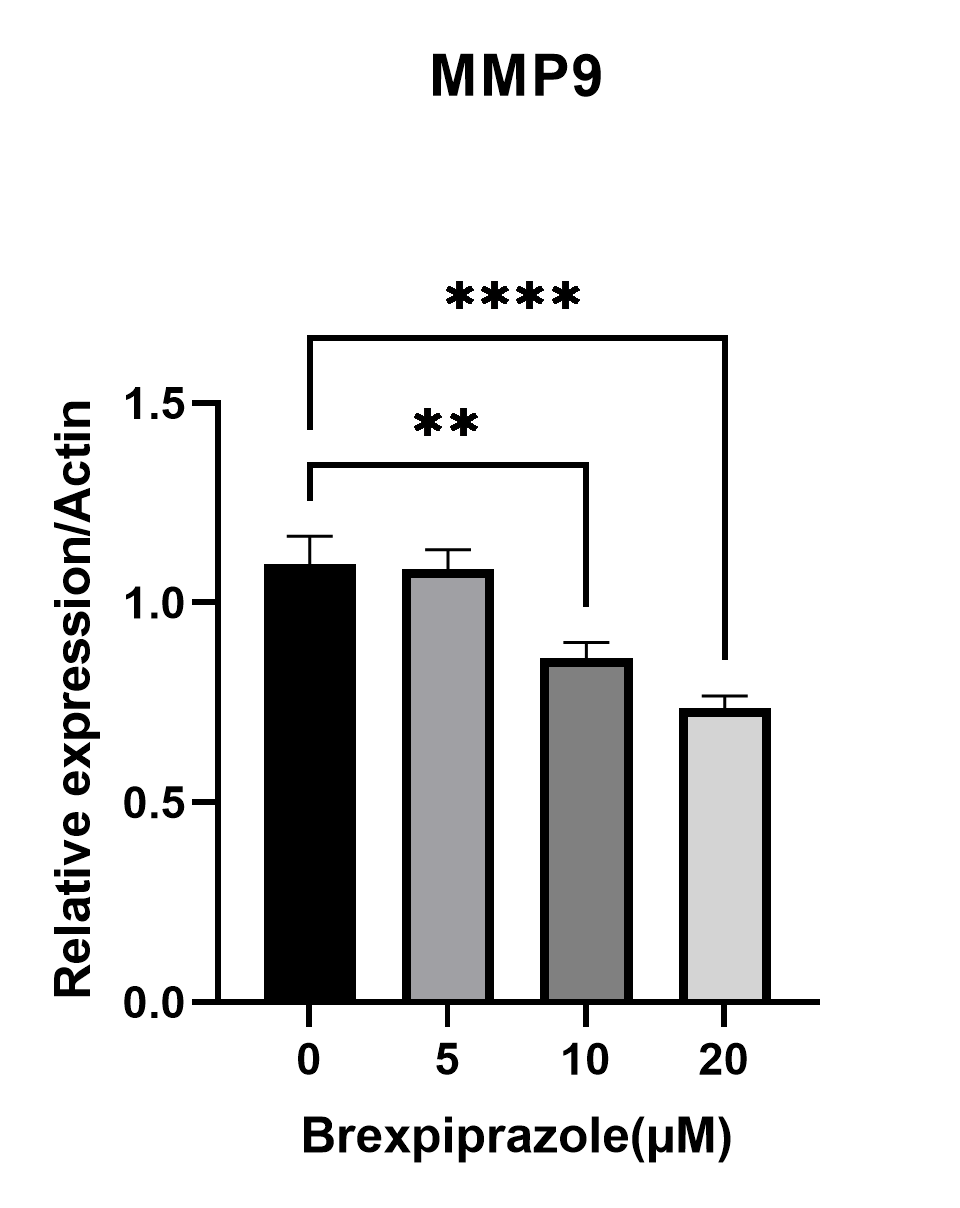

Supplement: Supplementary file 2 [file SupplementaryFile2.zip › WB数据/116/数据图/MMP9.tif]

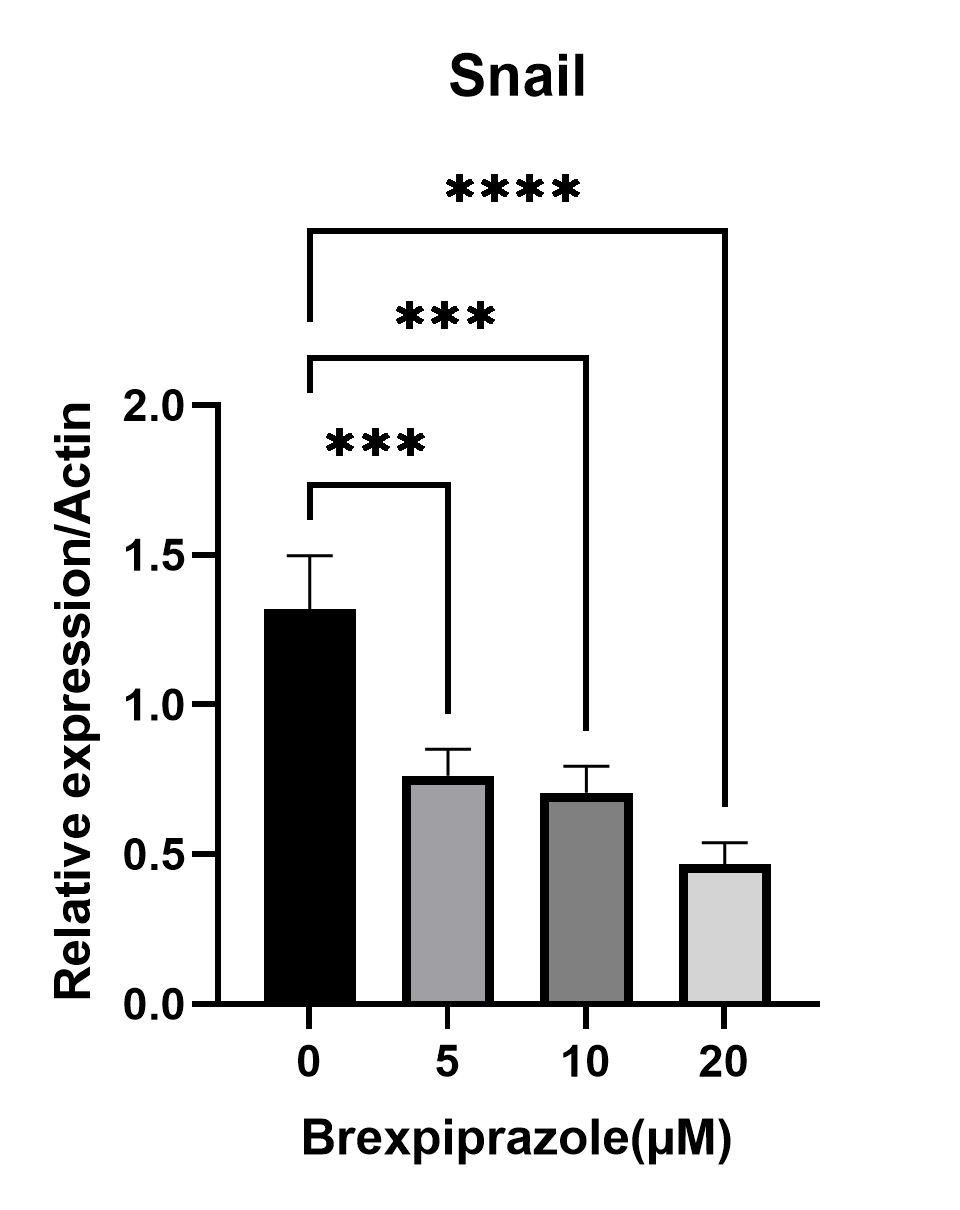

Supplement: Supplementary file 2 [file SupplementaryFile2.zip › WB数据/116/数据图/SNAIL.tif]

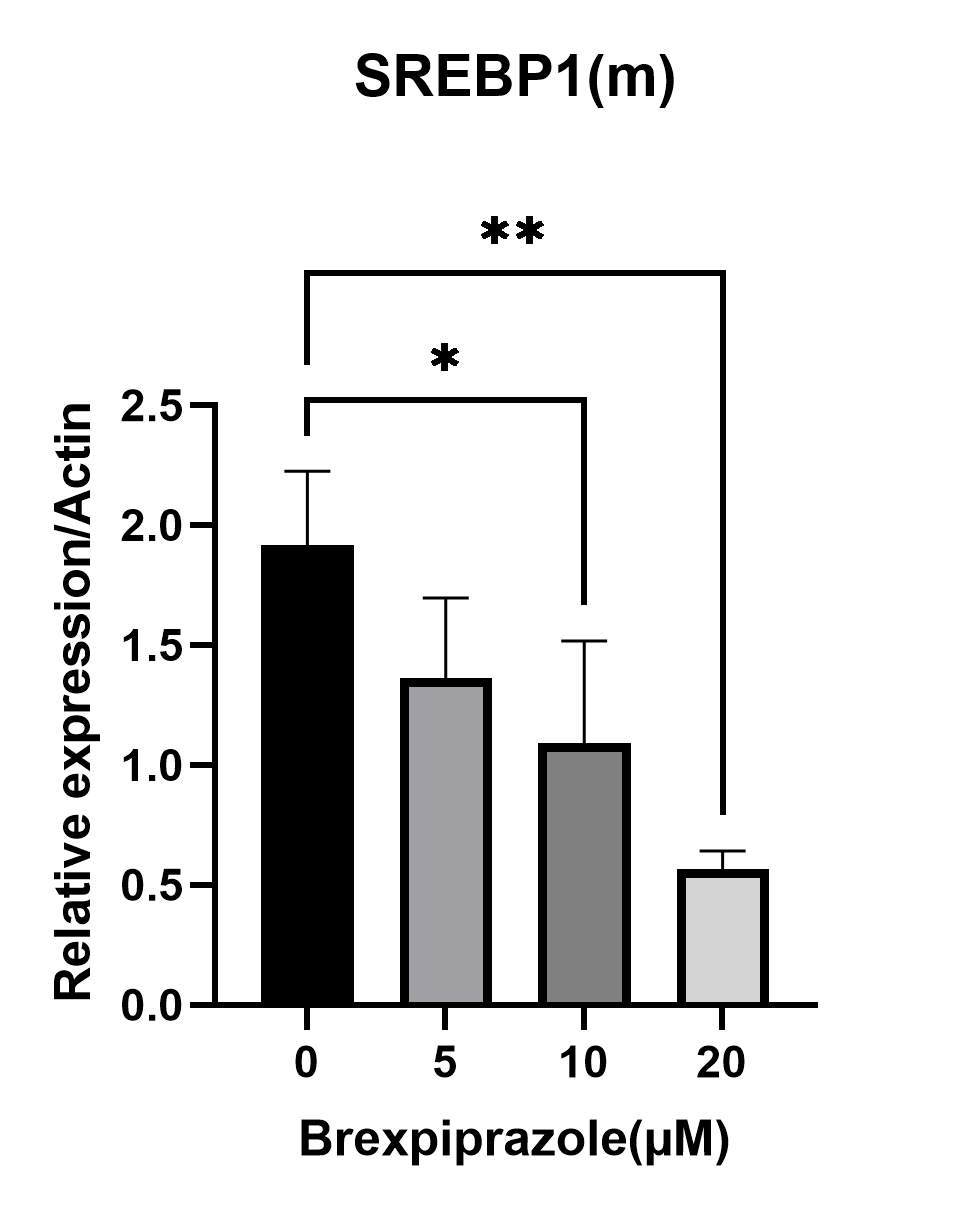

Supplement: Supplementary file 2 [file SupplementaryFile2.zip › WB数据/116/数据图/srebp1.tif]

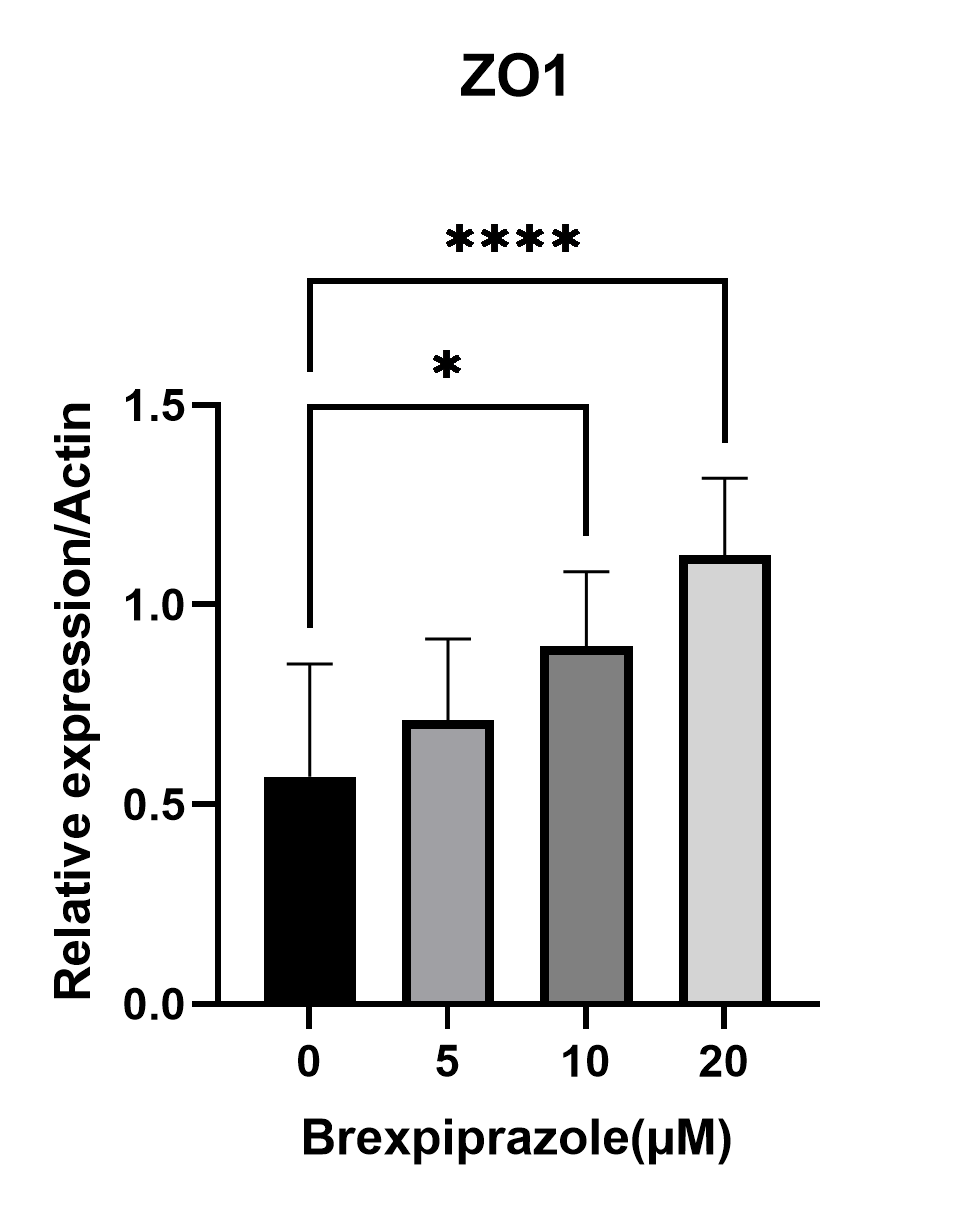

Supplement: Supplementary file 2 [file SupplementaryFile2.zip › WB数据/116/数据图/ZO1.tif]

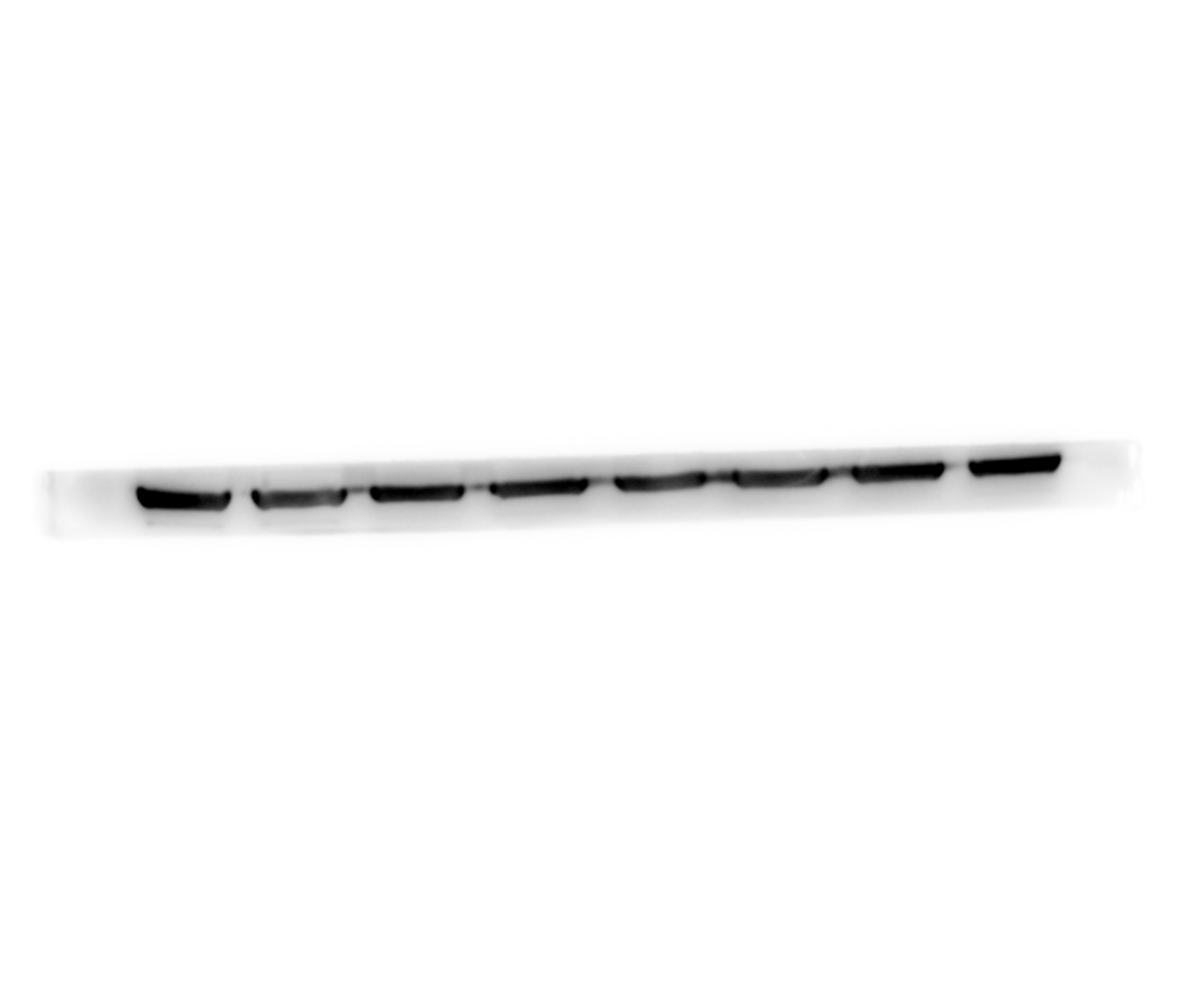

Supplement: Supplementary file 2 [file SupplementaryFile2.zip › WB数据/116-oe/116-wb/actin/116-actin.tif]

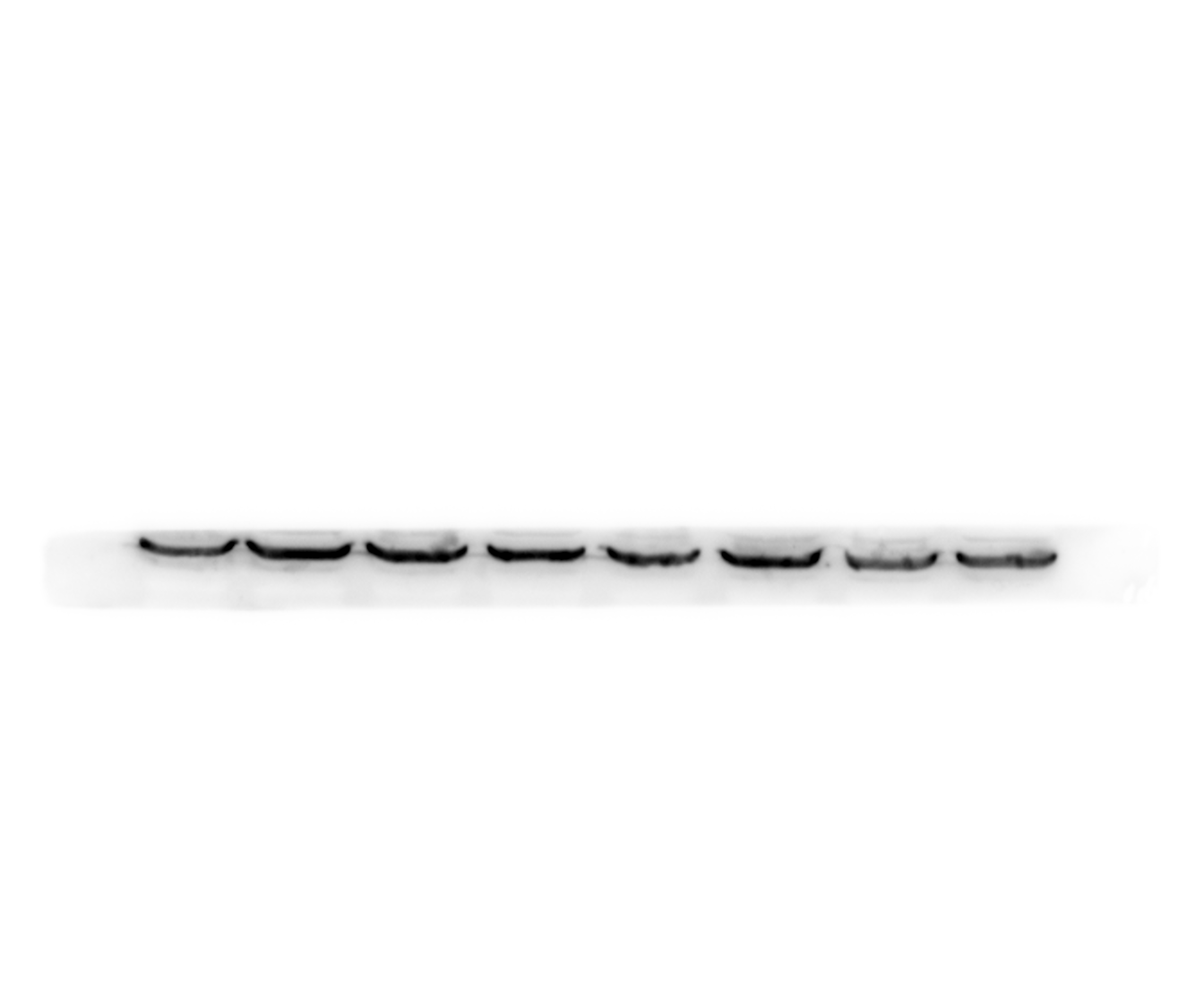

Supplement: Supplementary file 2 [file SupplementaryFile2.zip › WB数据/116-oe/116-wb/actin/116-actin`.tif]

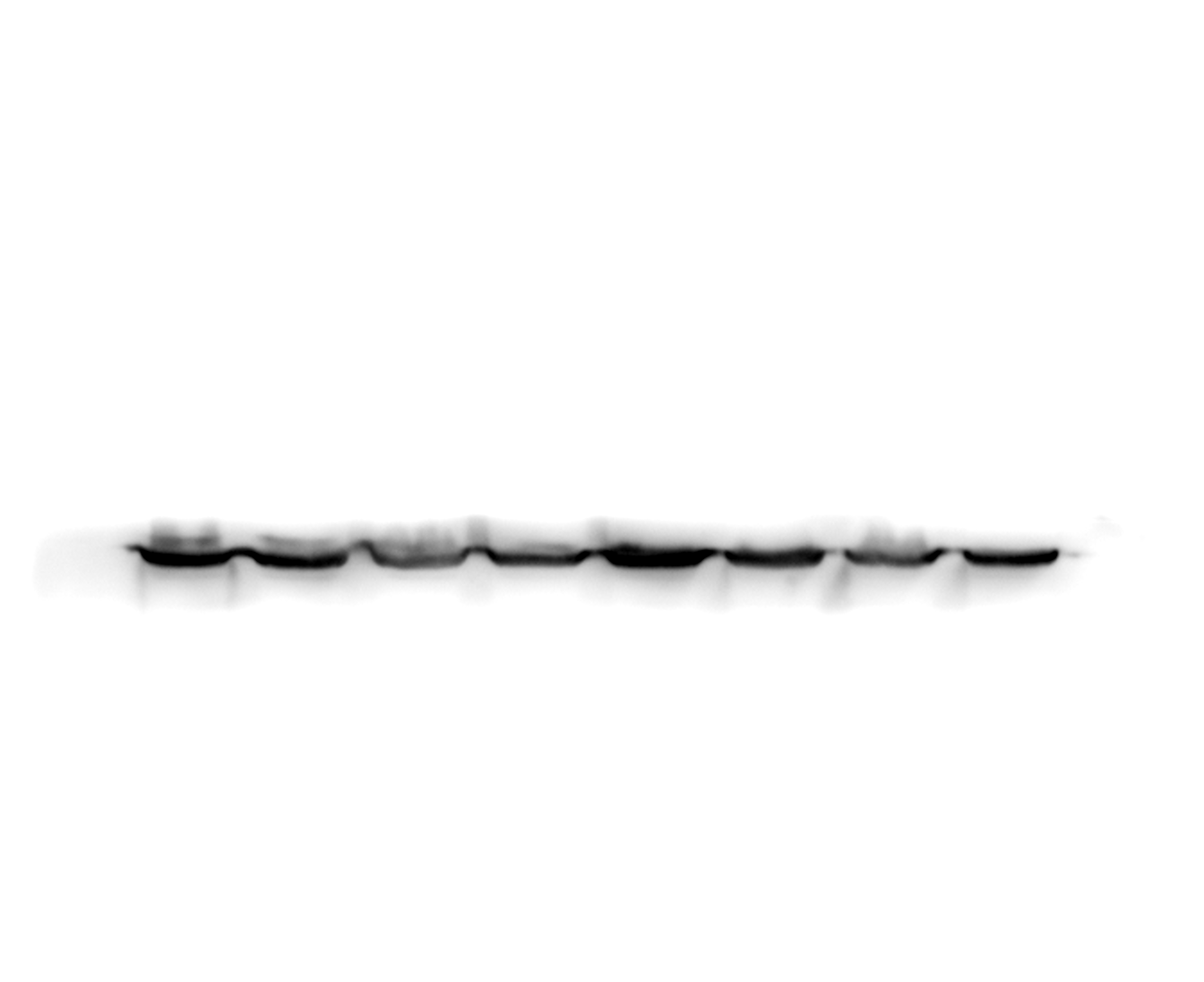

Supplement: Supplementary file 2 [file SupplementaryFile2.zip › WB数据/116-oe/116-wb/actin/actin.tif]

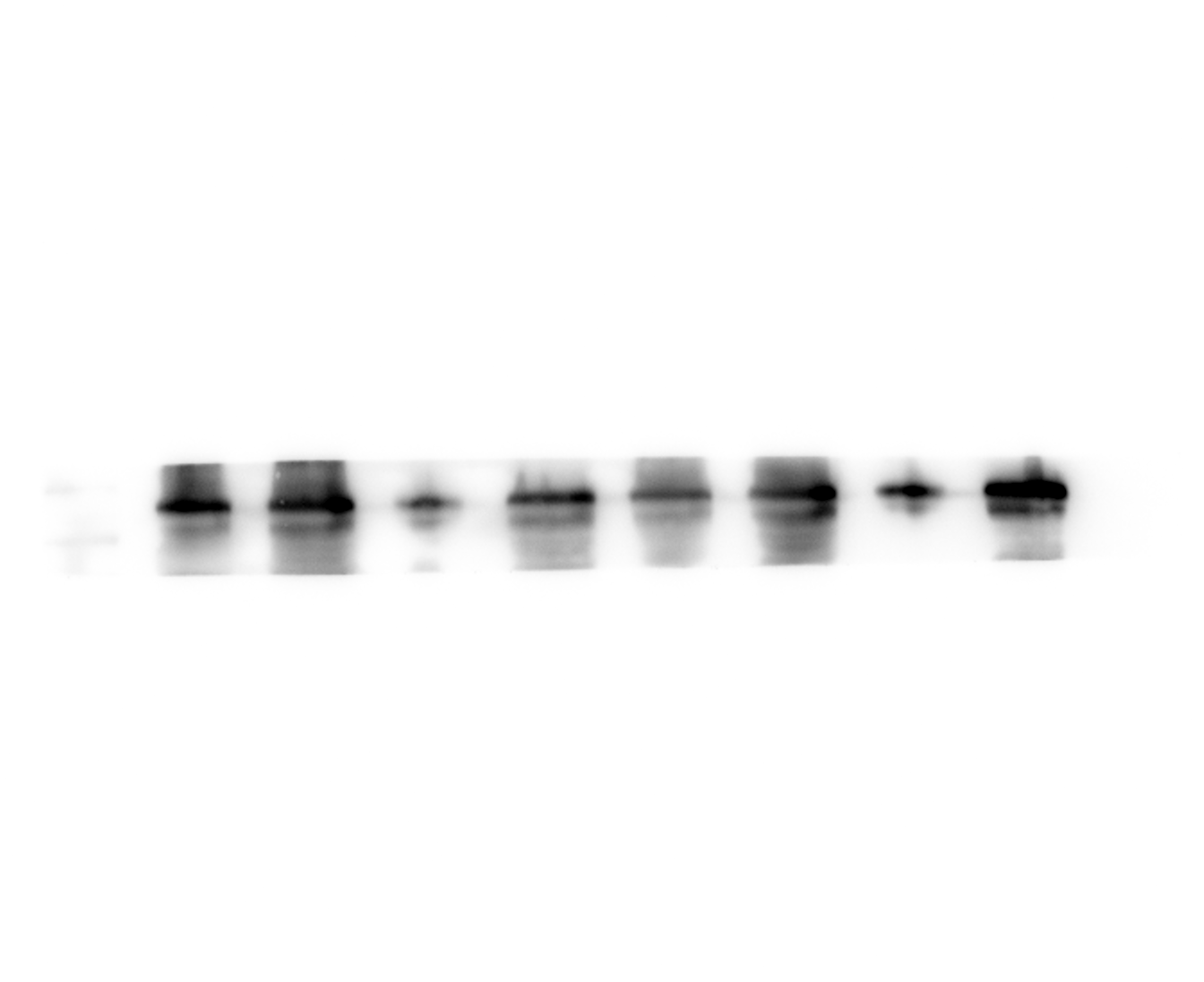

Supplement: Supplementary file 2 [file SupplementaryFile2.zip › WB数据/116-oe/116-wb/e/116-e----.tif]

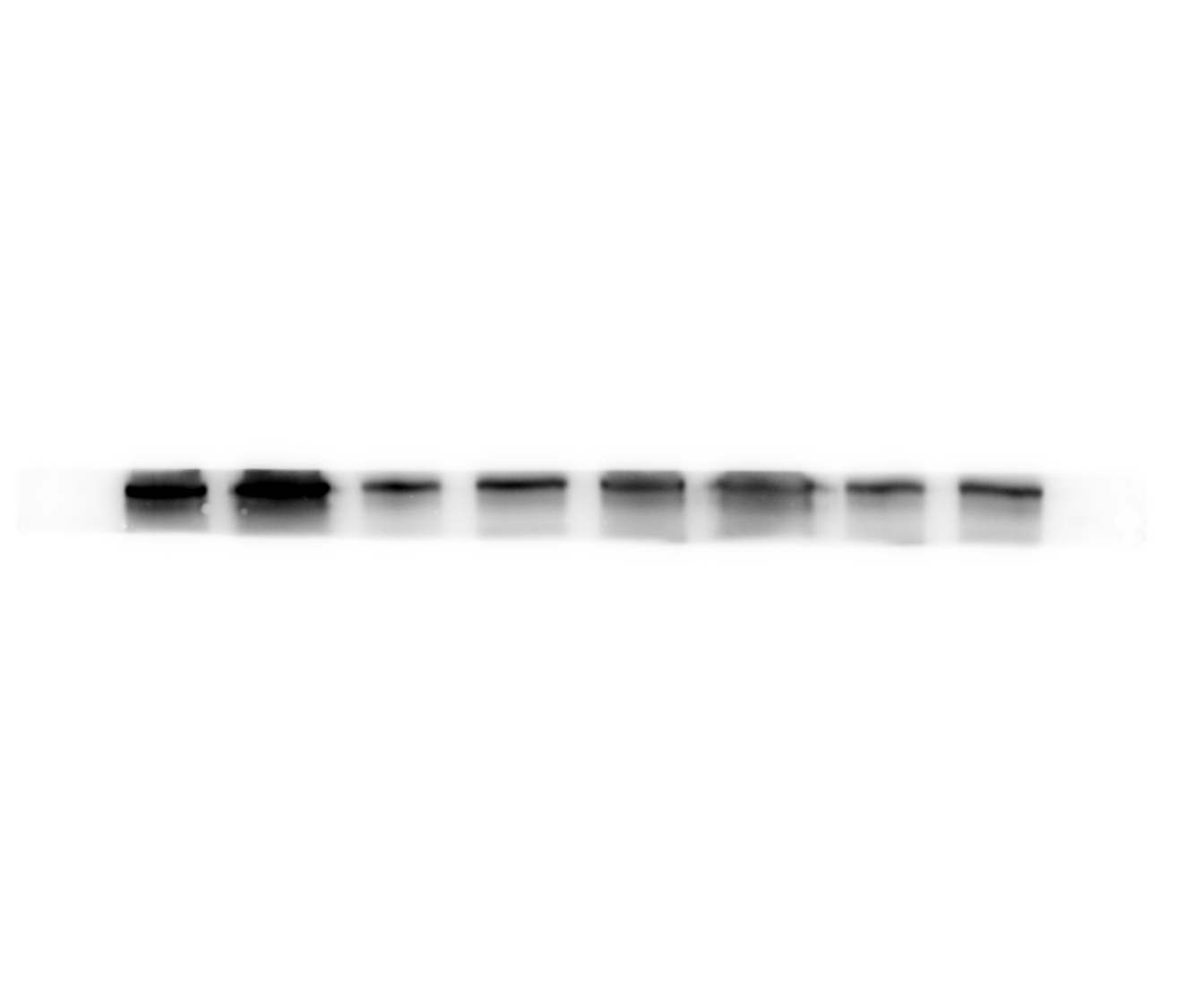

Supplement: Supplementary file 2 [file SupplementaryFile2.zip › WB数据/116-oe/116-wb/e/116-e.tif]

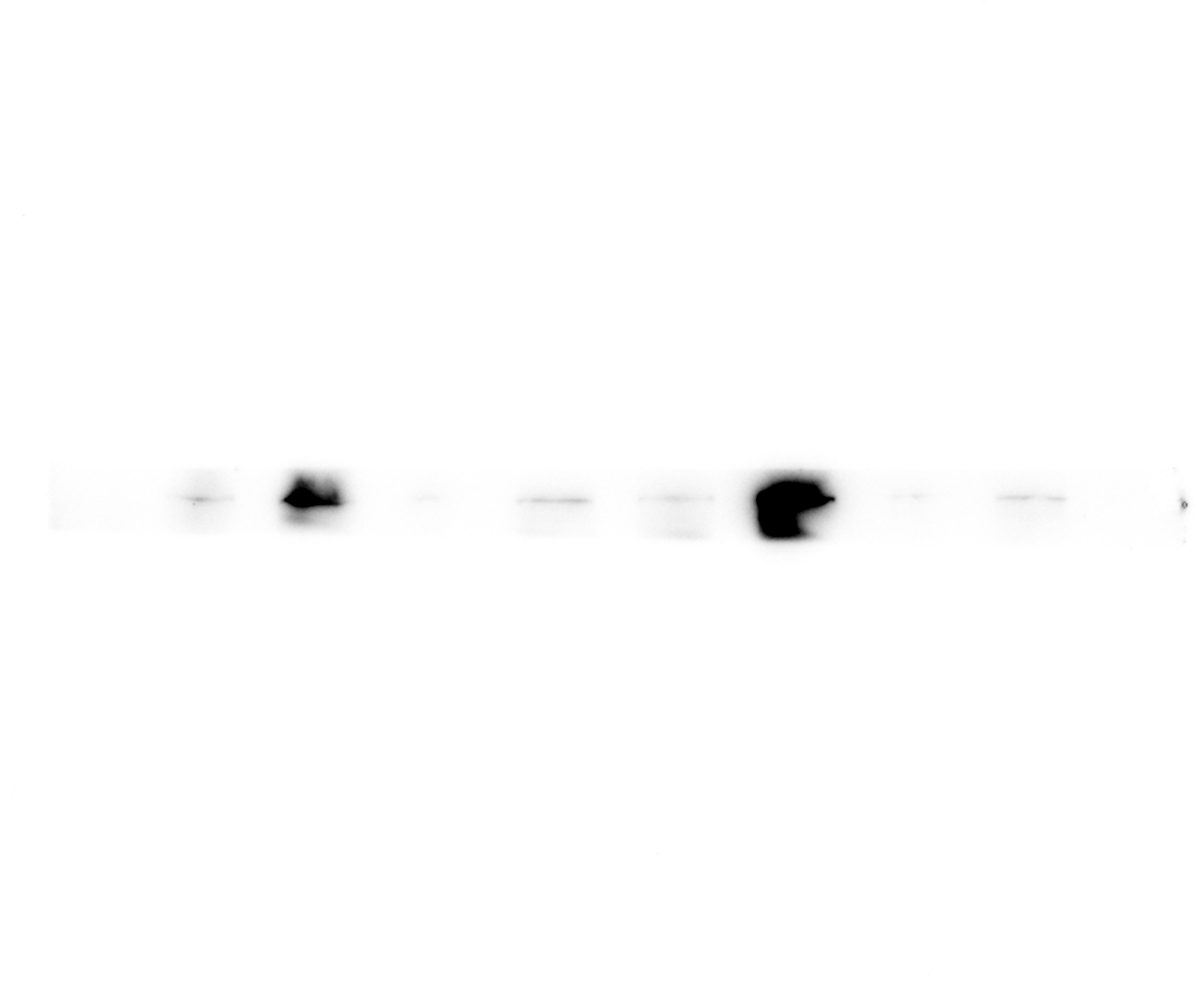

Supplement: Supplementary file 2 [file SupplementaryFile2.zip › WB数据/116-oe/116-wb/e/e----.tif]

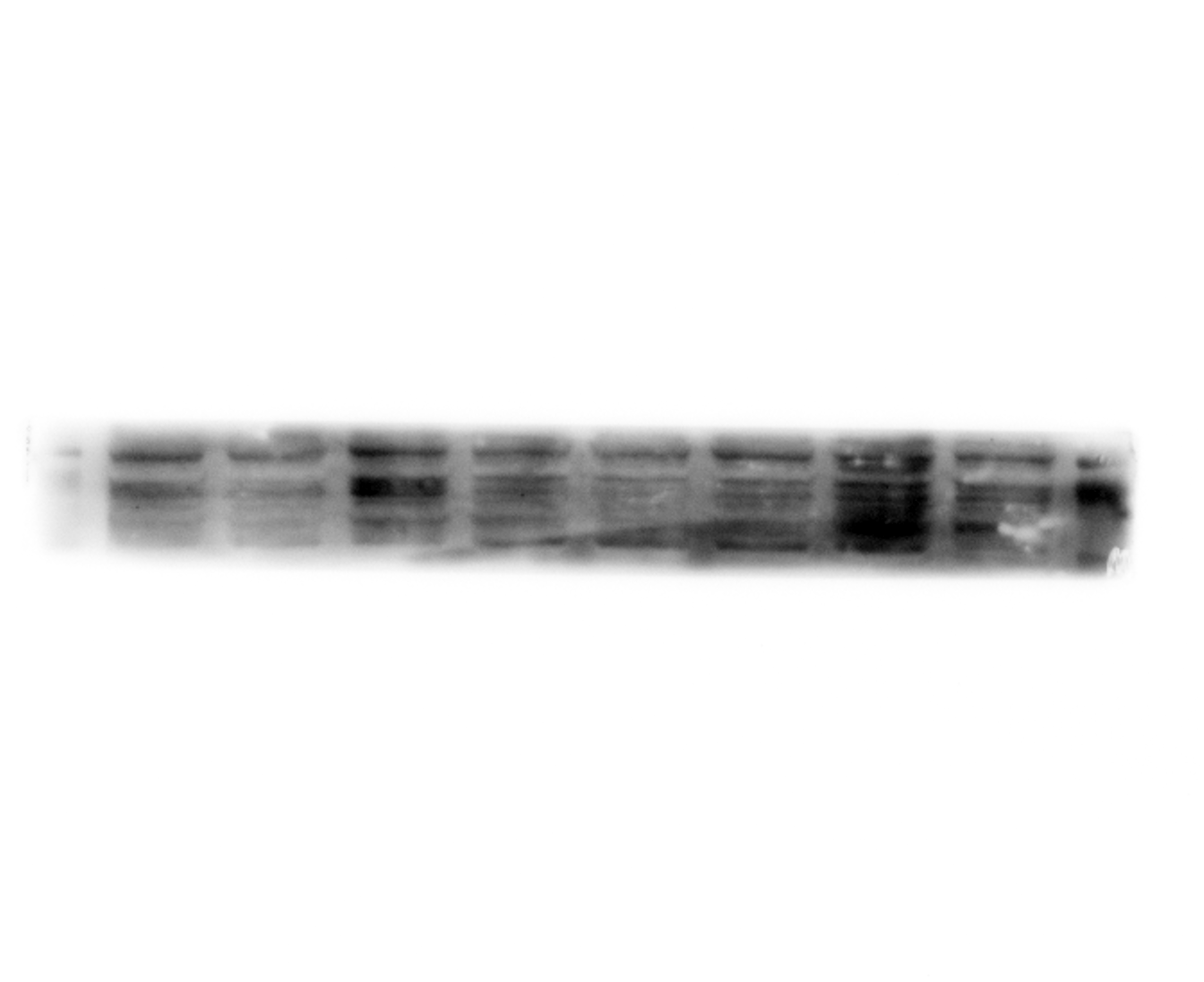

Supplement: Supplementary file 2 [file SupplementaryFile2.zip › WB数据/116-oe/116-wb/mmp9/116--mmp9.tif]

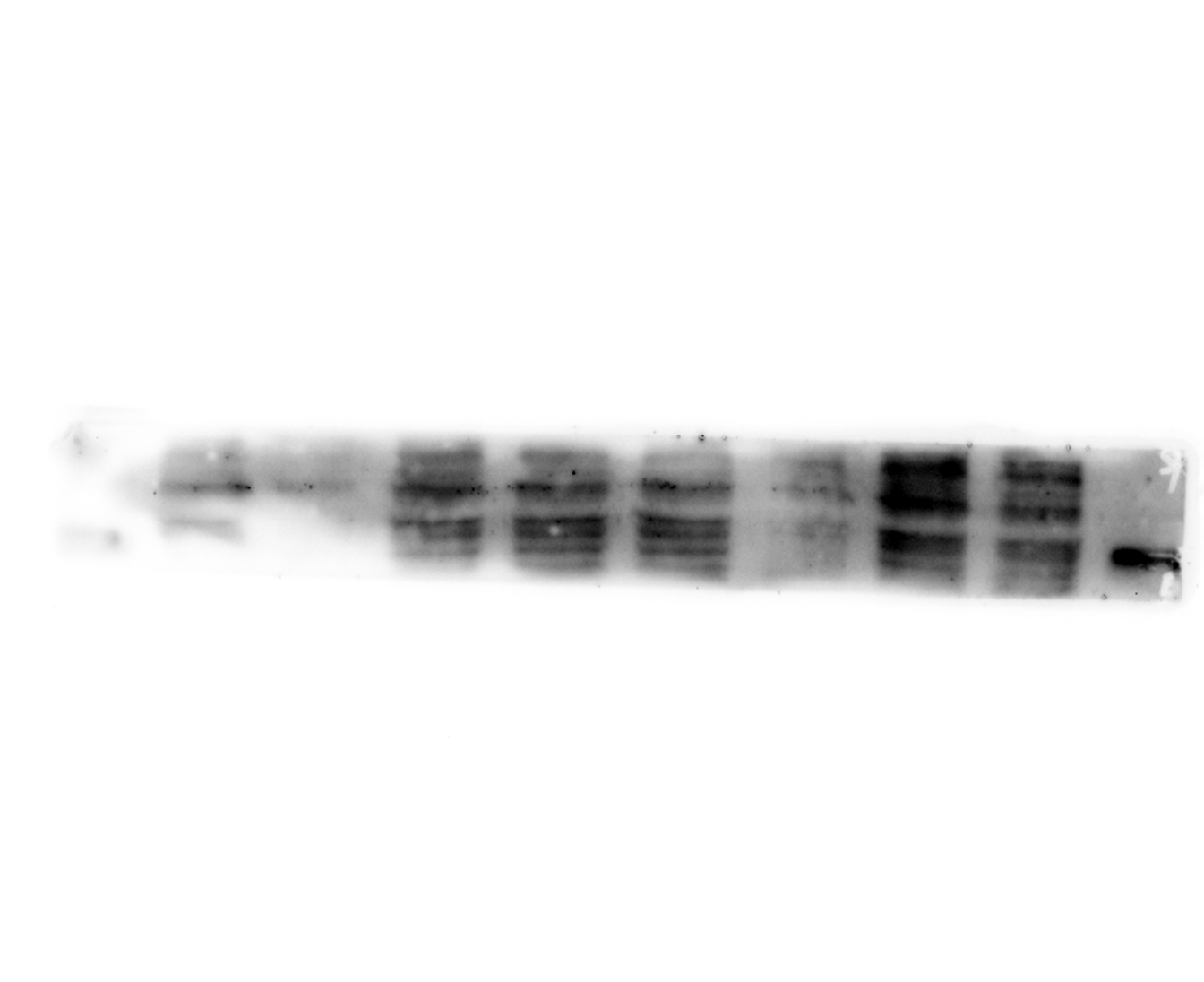

Supplement: Supplementary file 2 [file SupplementaryFile2.zip › WB数据/116-oe/116-wb/mmp9/116-mmp9-.tif]

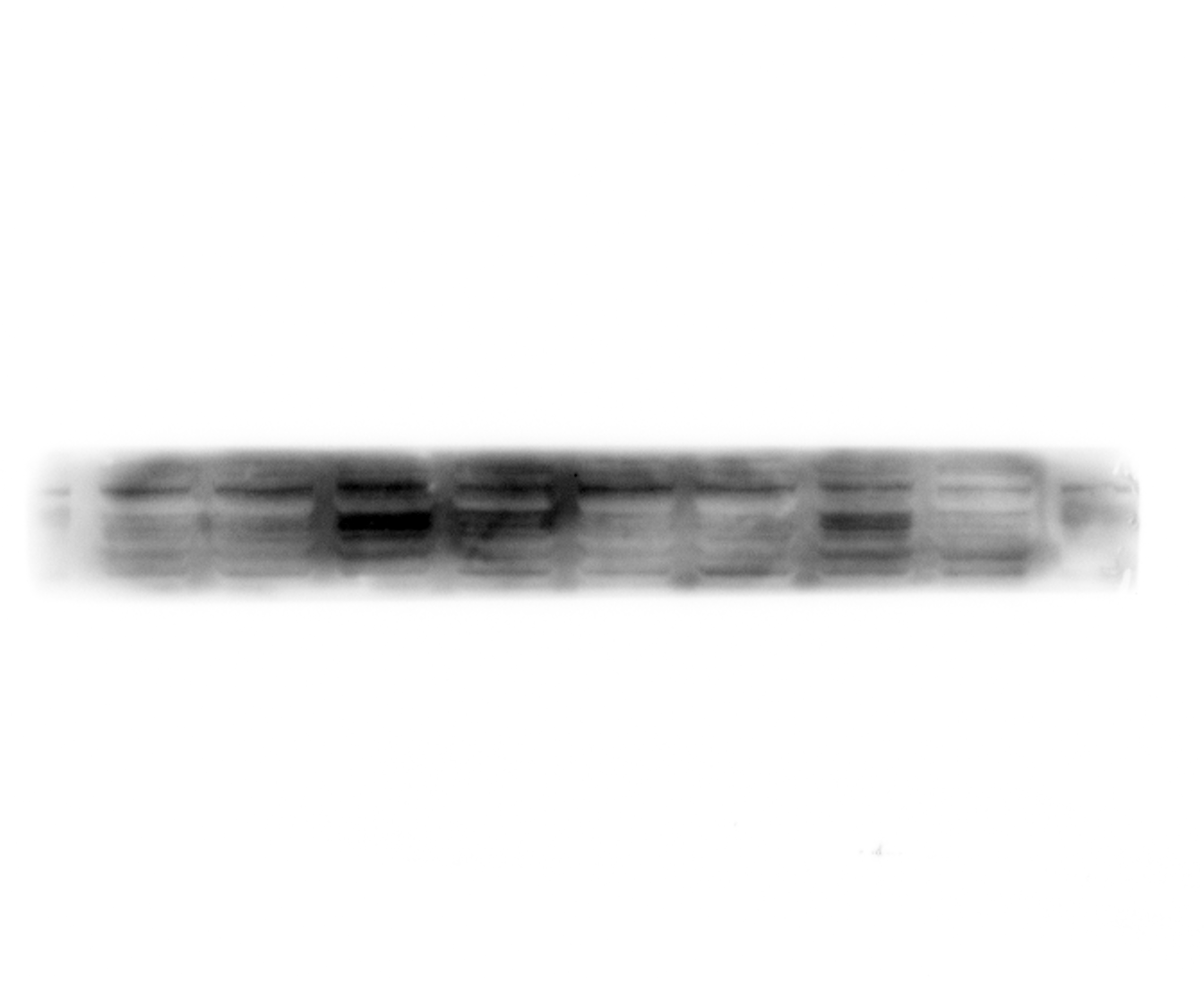

Supplement: Supplementary file 2 [file SupplementaryFile2.zip › WB数据/116-oe/116-wb/mmp9/116-mmp9.tif]

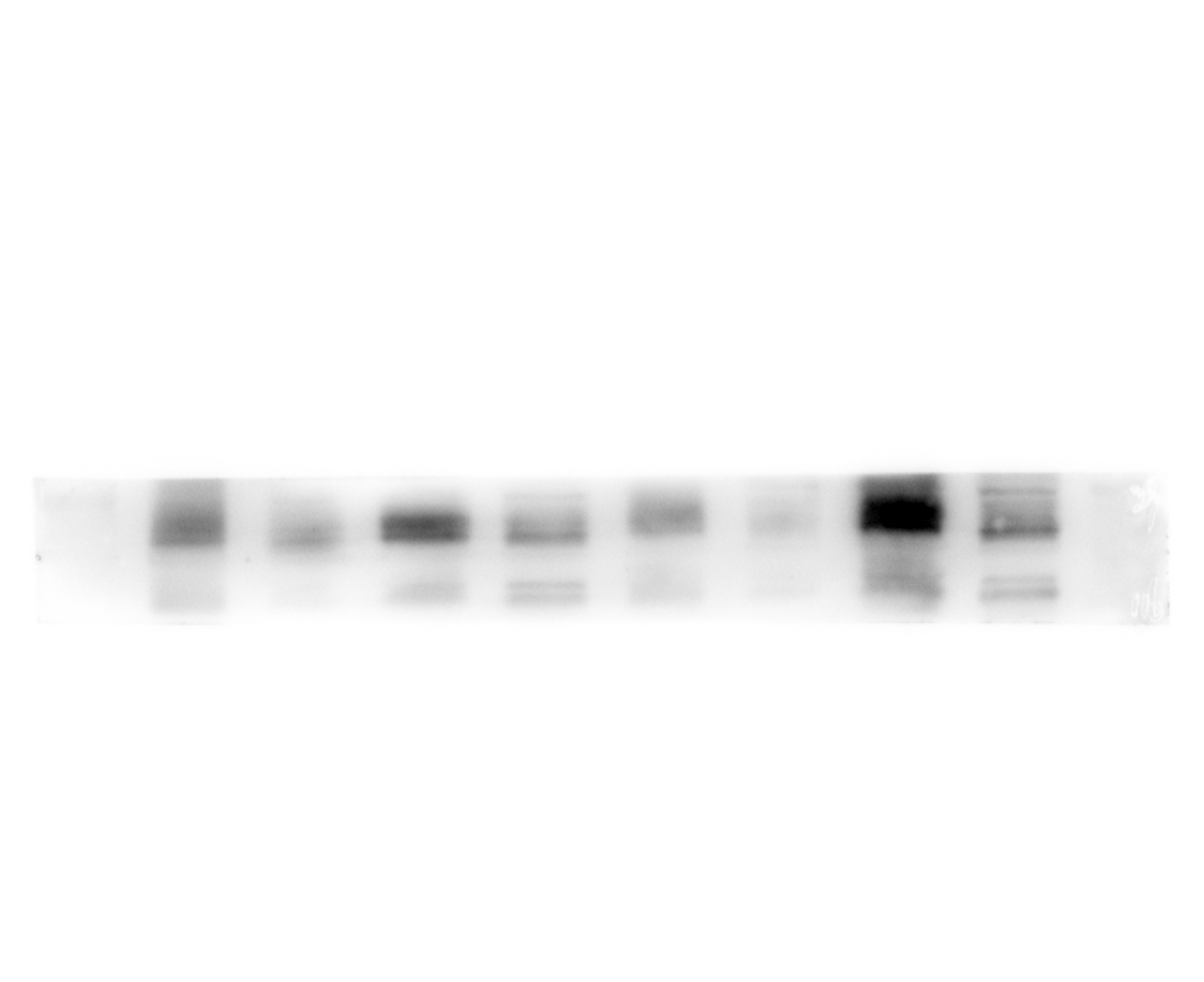

Supplement: Supplementary file 2 [file SupplementaryFile2.zip › WB数据/116-oe/116-wb/snail/116-snail--.tif]

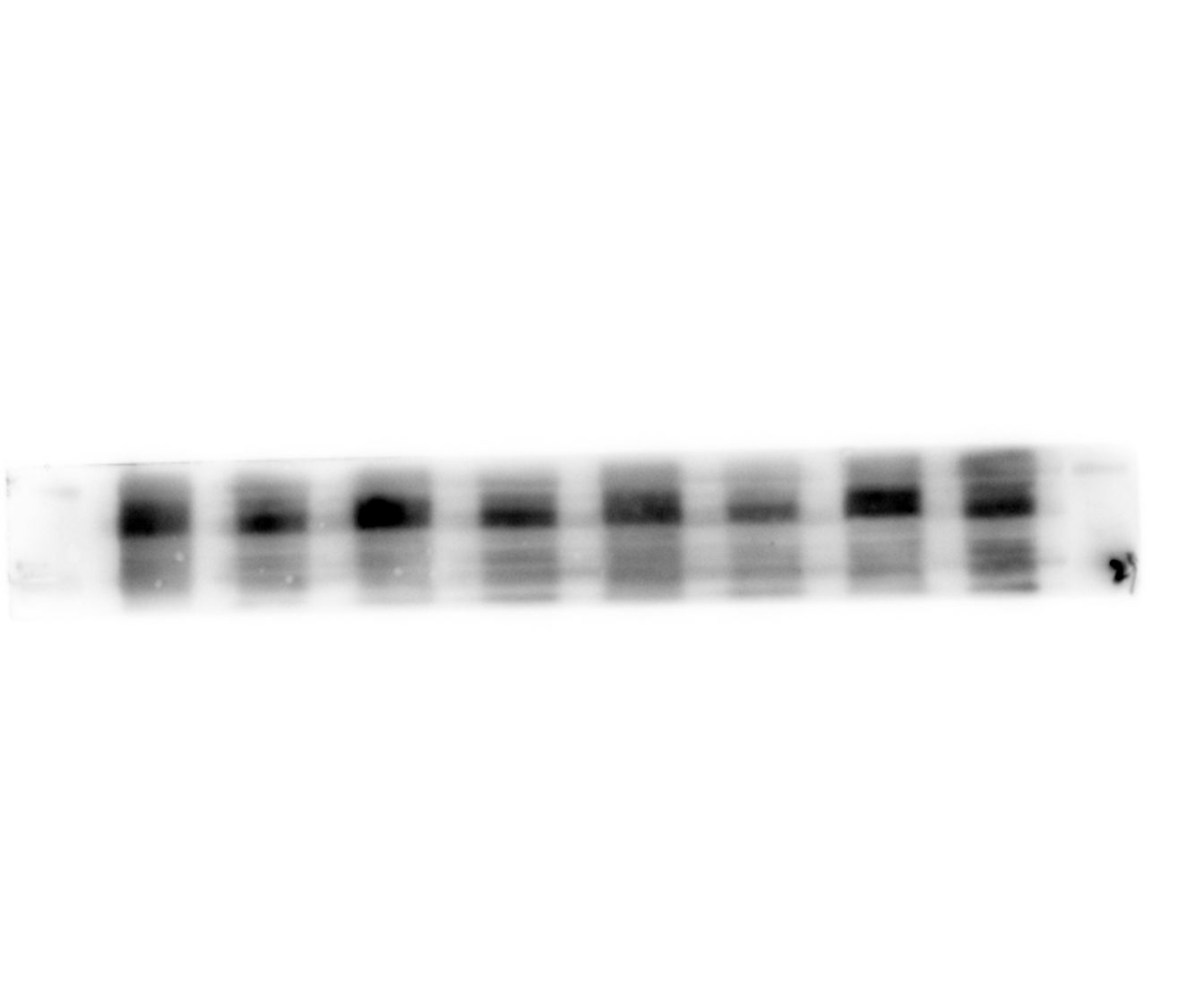

Supplement: Supplementary file 2 [file SupplementaryFile2.zip › WB数据/116-oe/116-wb/snail/116-snail-.tif]

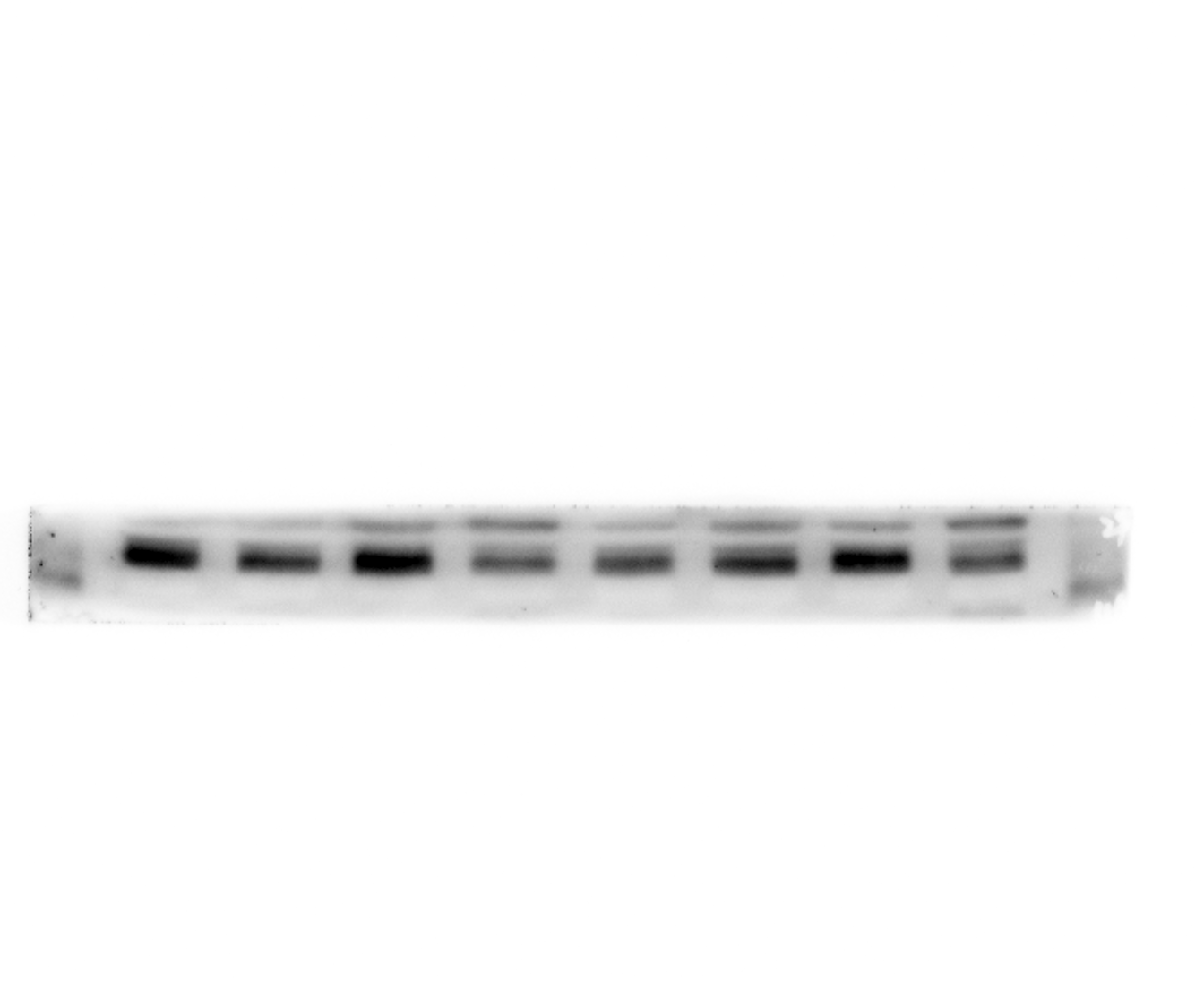

Supplement: Supplementary file 2 [file SupplementaryFile2.zip › WB数据/116-oe/116-wb/snail/116-snail.tif]

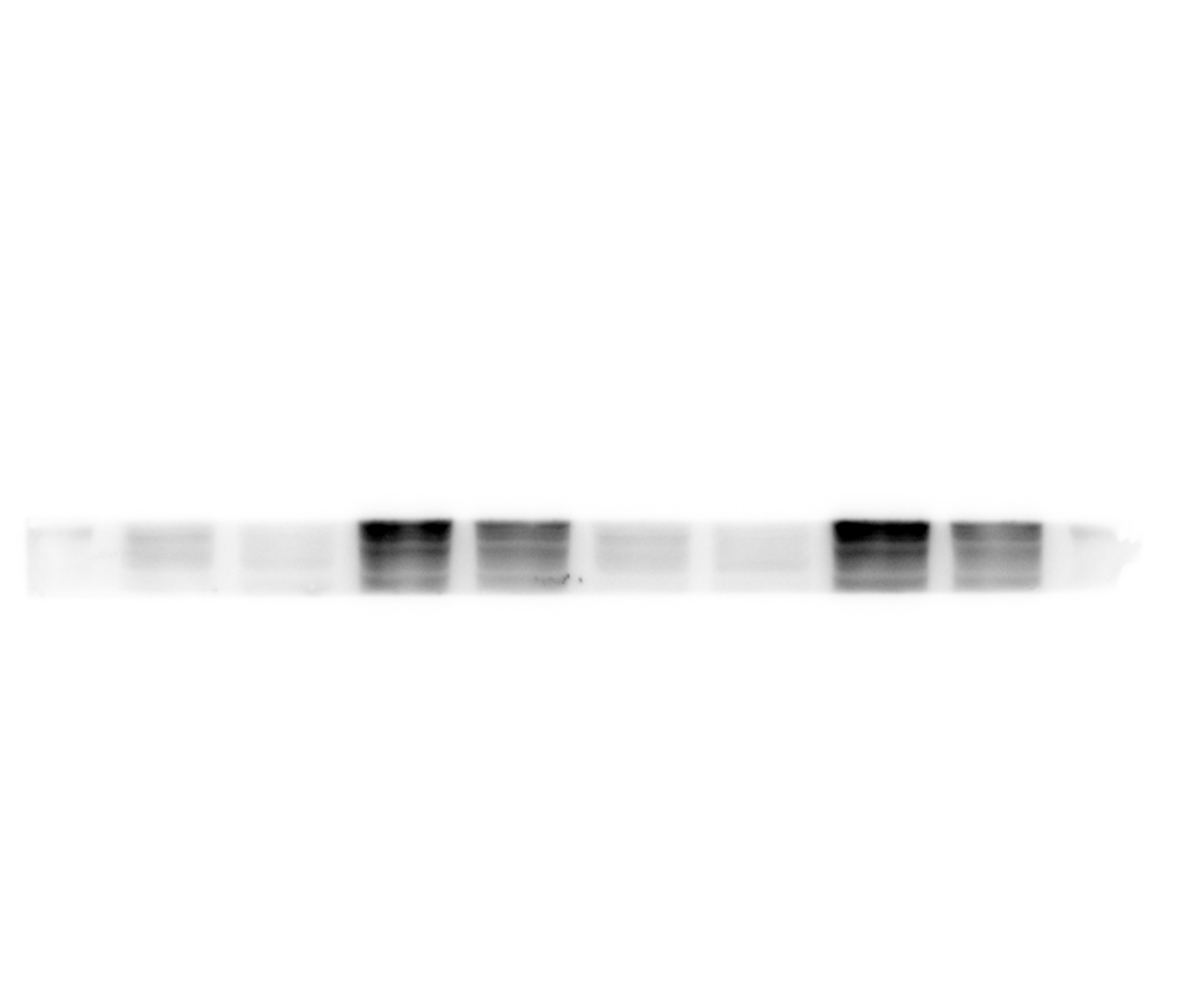

Supplement: Supplementary file 2 [file SupplementaryFile2.zip › WB数据/116-oe/116-wb/srebp1/116-srebp1-68-.tif]

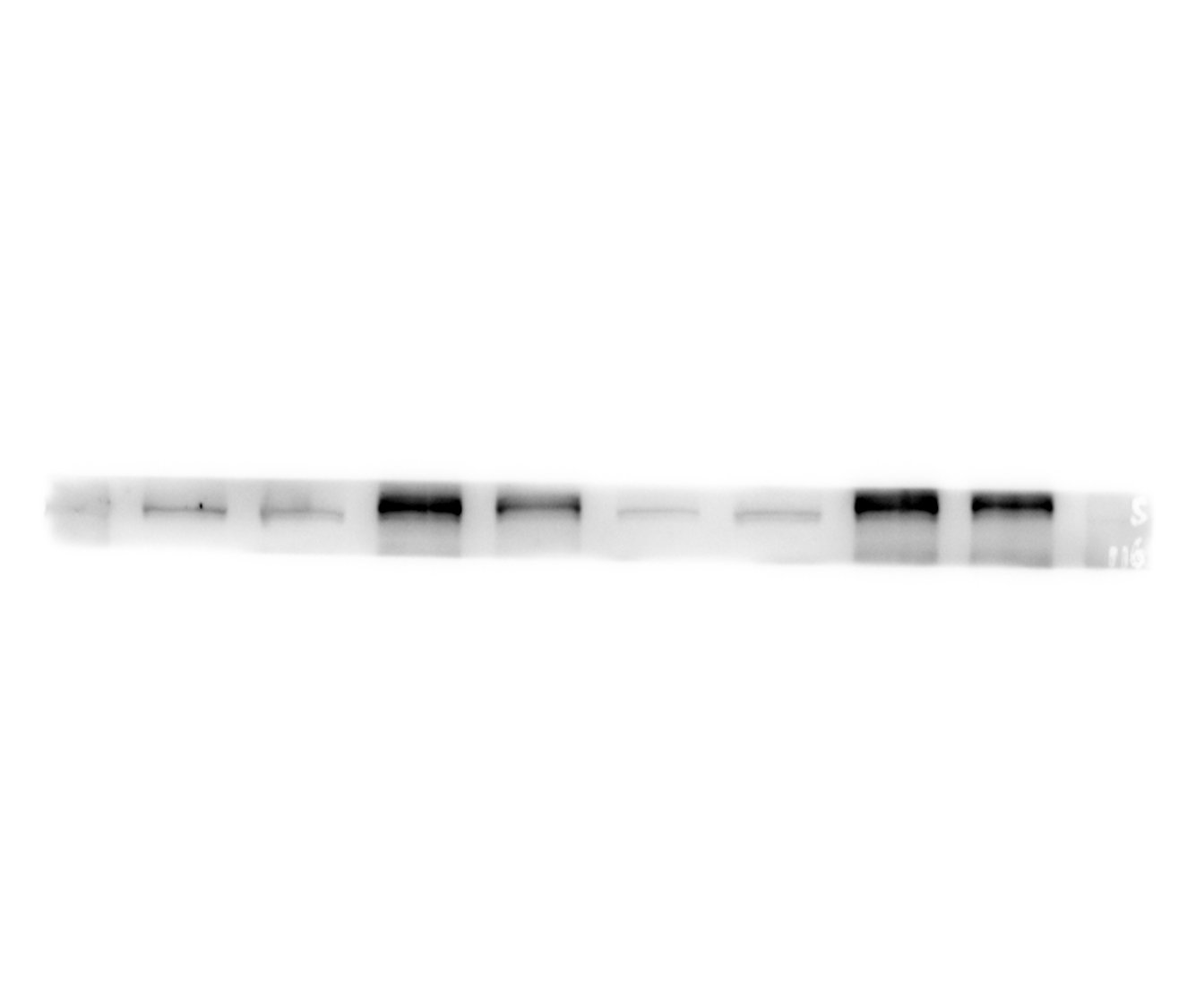

Supplement: Supplementary file 2 [file SupplementaryFile2.zip › WB数据/116-oe/116-wb/srebp1/116-srebp1-68.tif]

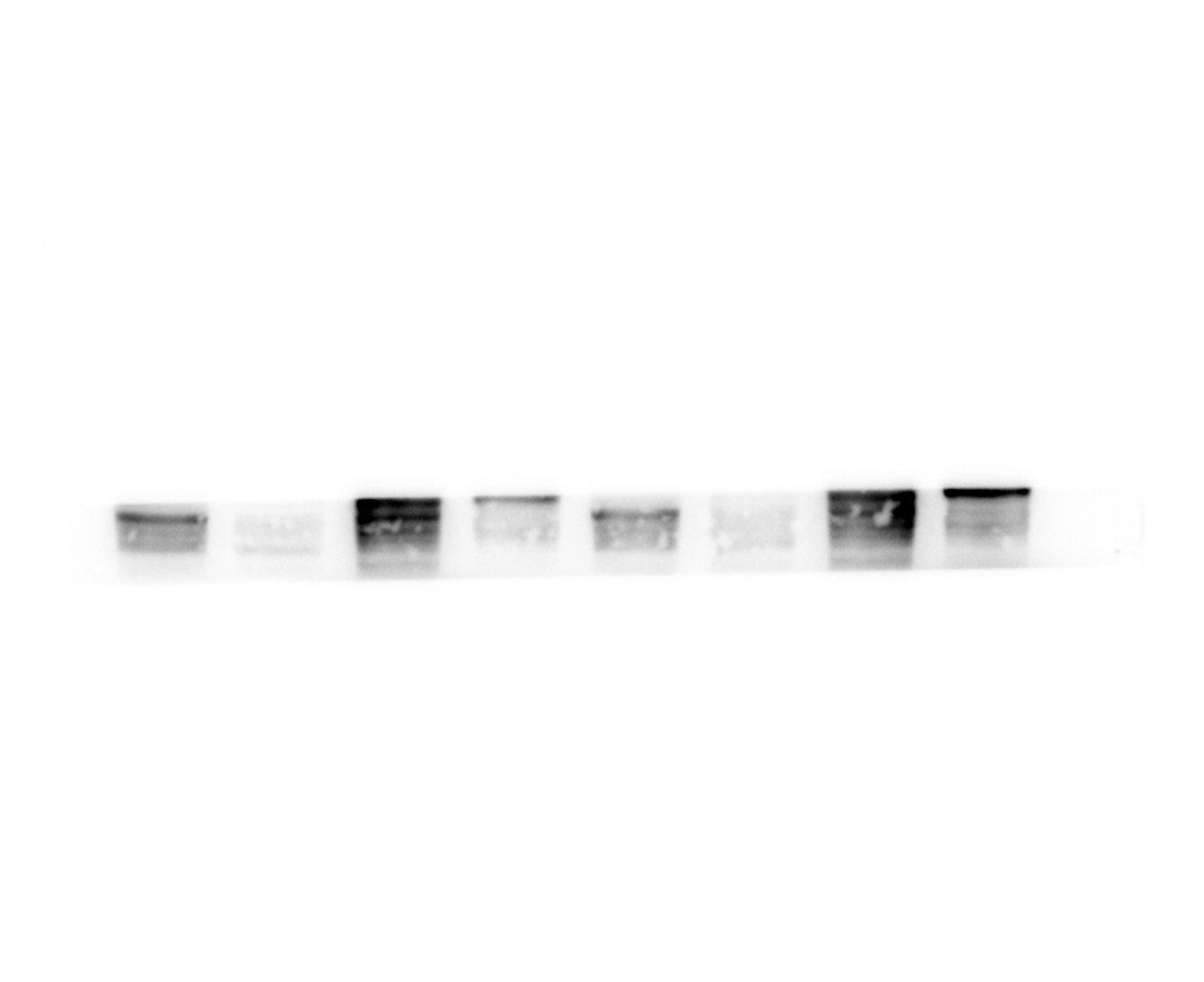

Supplement: Supplementary file 2 [file SupplementaryFile2.zip › WB数据/116-oe/116-wb/srebp1/srebp1-68.tif]

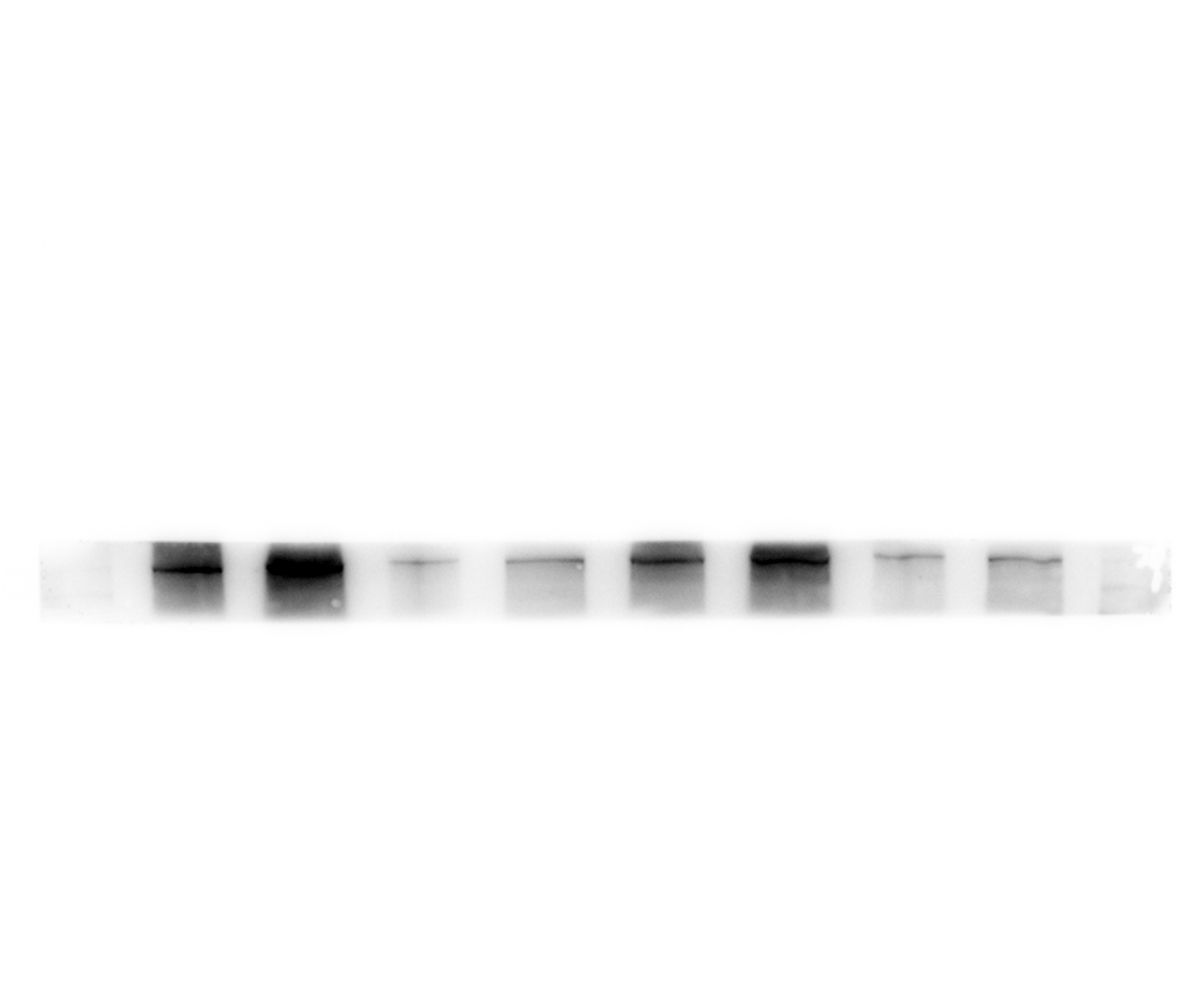

Supplement: Supplementary file 2 [file SupplementaryFile2.zip › WB数据/116-oe/116-wb/zo1/116-zo1--.tif]

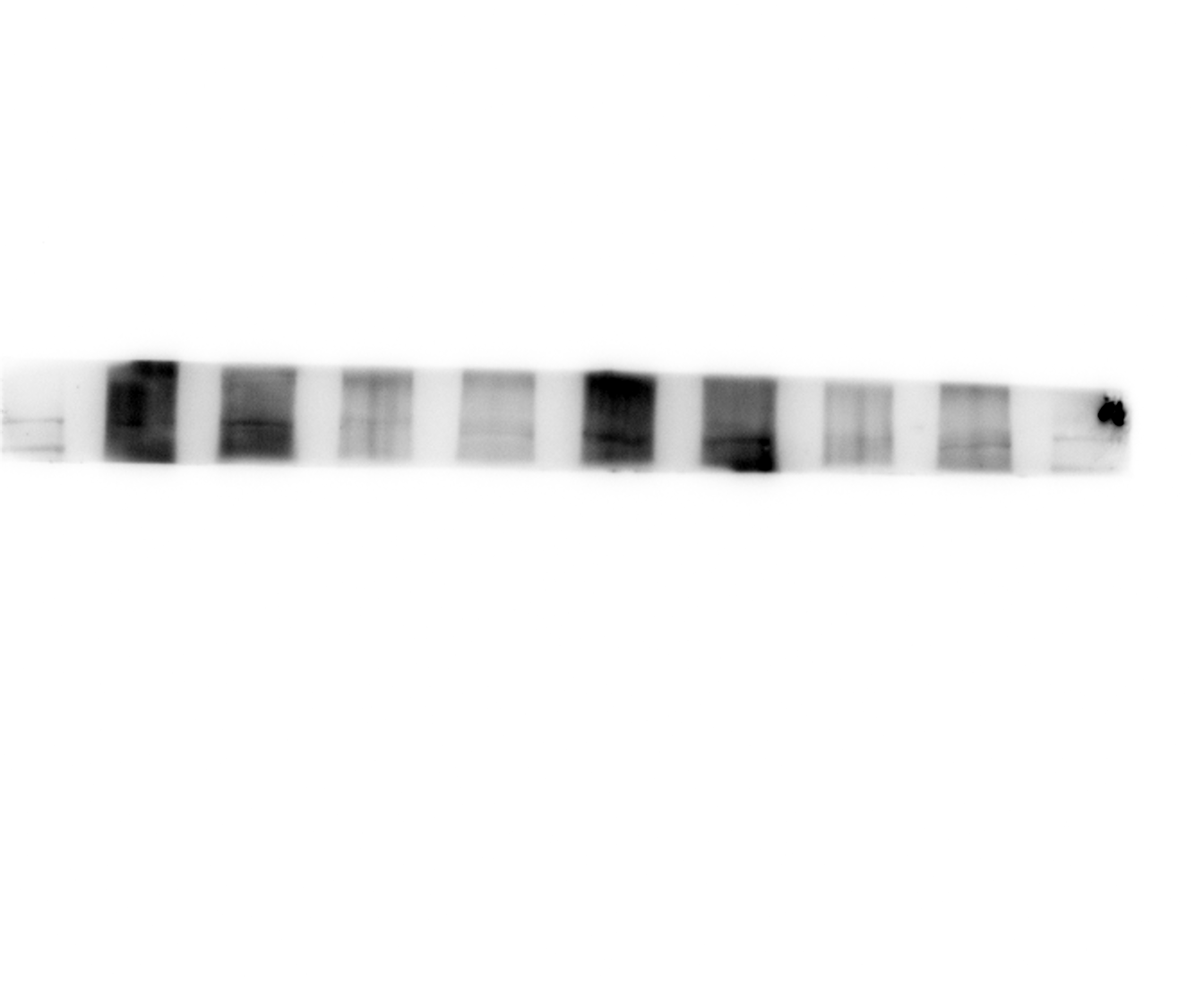

Supplement: Supplementary file 2 [file SupplementaryFile2.zip › WB数据/116-oe/116-wb/zo1/116-zo1.tif]

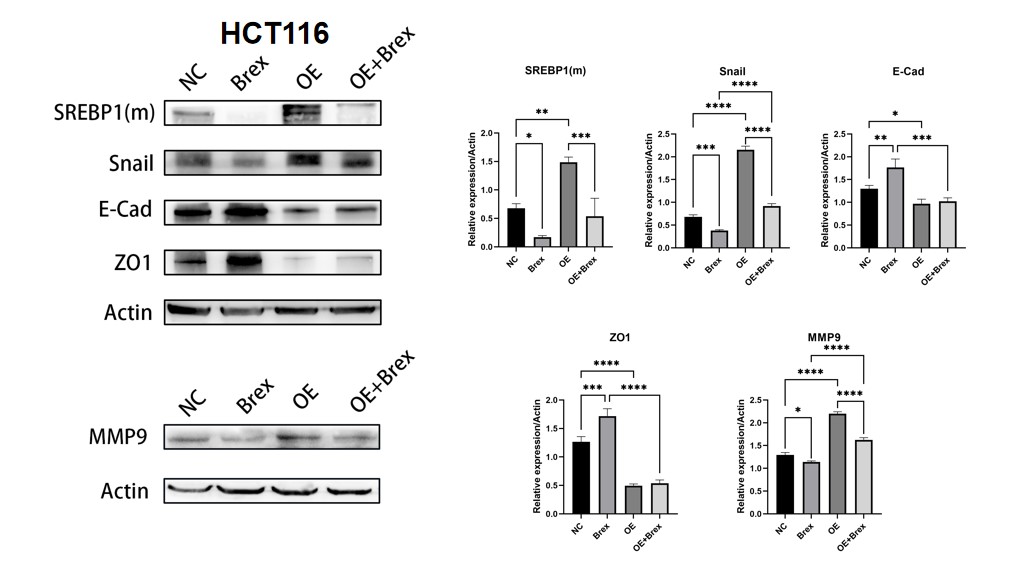

Supplement: Supplementary file 2 [file SupplementaryFile2.zip › WB数据/116-oe/数据图/1.jpg]

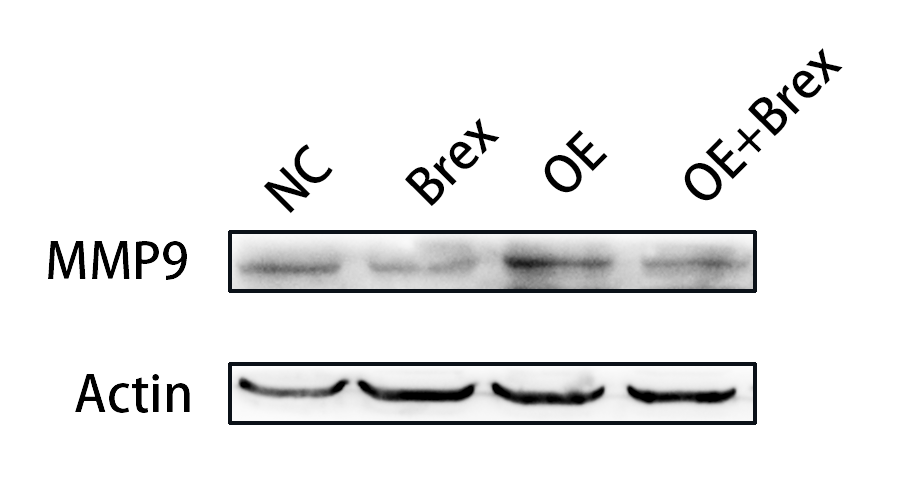

Supplement: Supplementary file 2 [file SupplementaryFile2.zip › WB数据/116-oe/数据图/116-OE-MMP9.png]

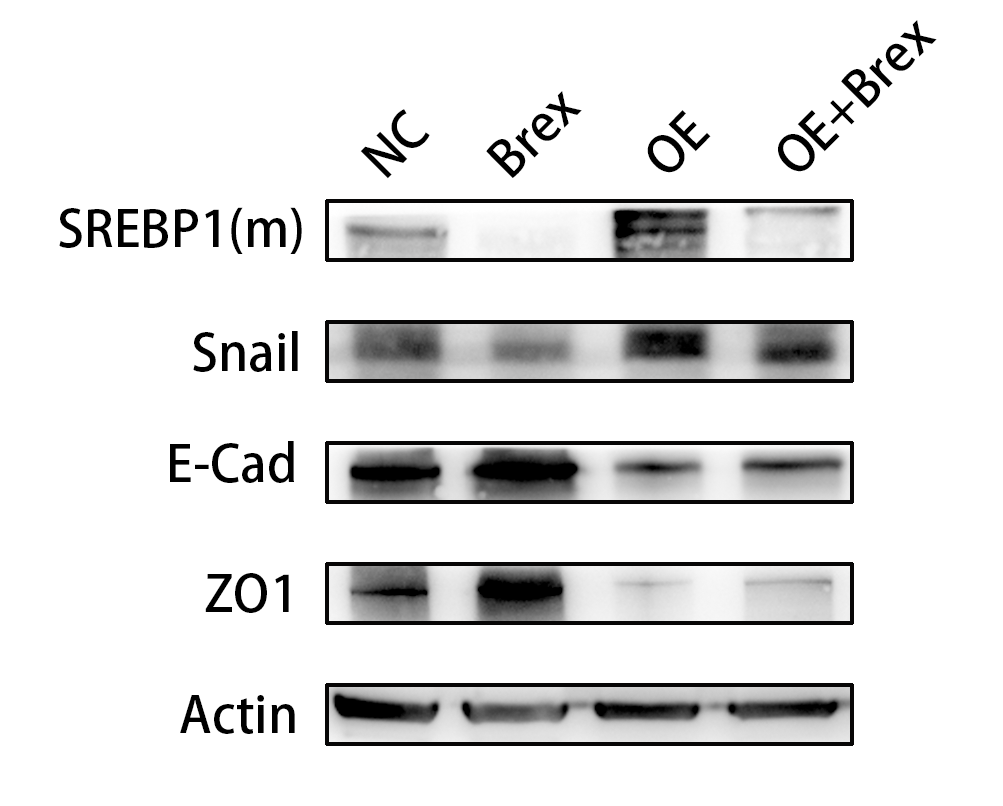

Supplement: Supplementary file 2 [file SupplementaryFile2.zip › WB数据/116-oe/数据图/116-OE.png]

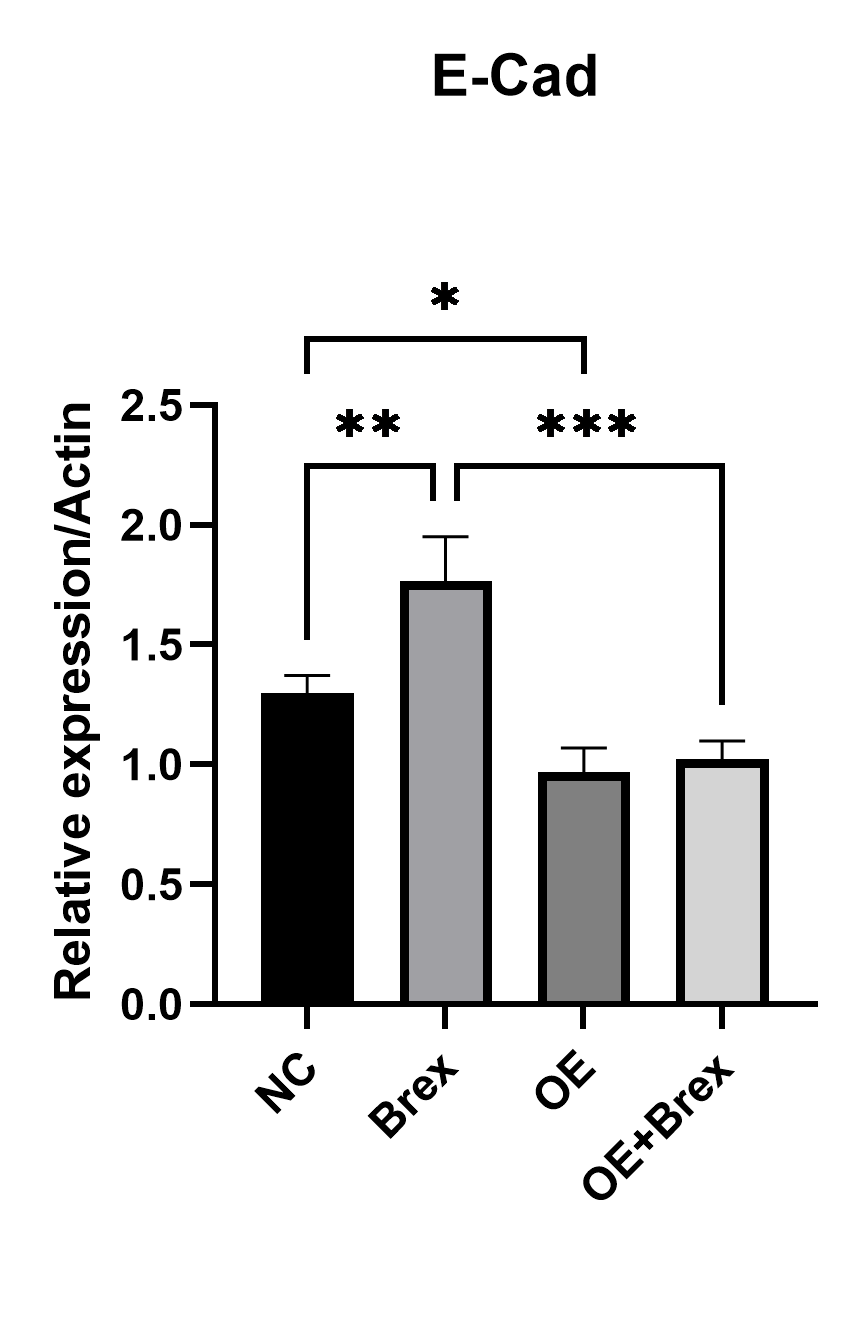

Supplement: Supplementary file 2 [file SupplementaryFile2.zip › WB数据/116-oe/数据图/E.tif]

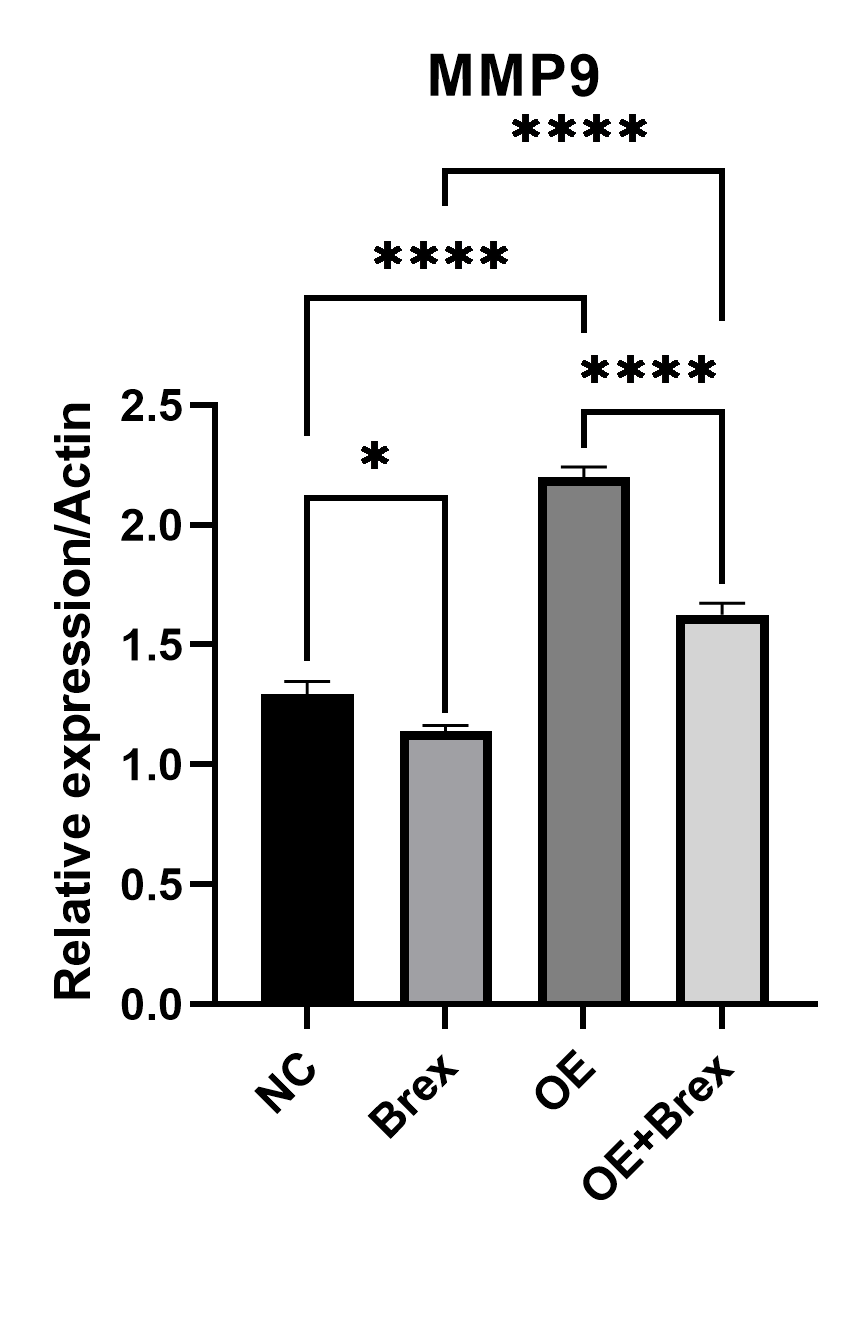

Supplement: Supplementary file 2 [file SupplementaryFile2.zip › WB数据/116-oe/数据图/MMP9.tif]

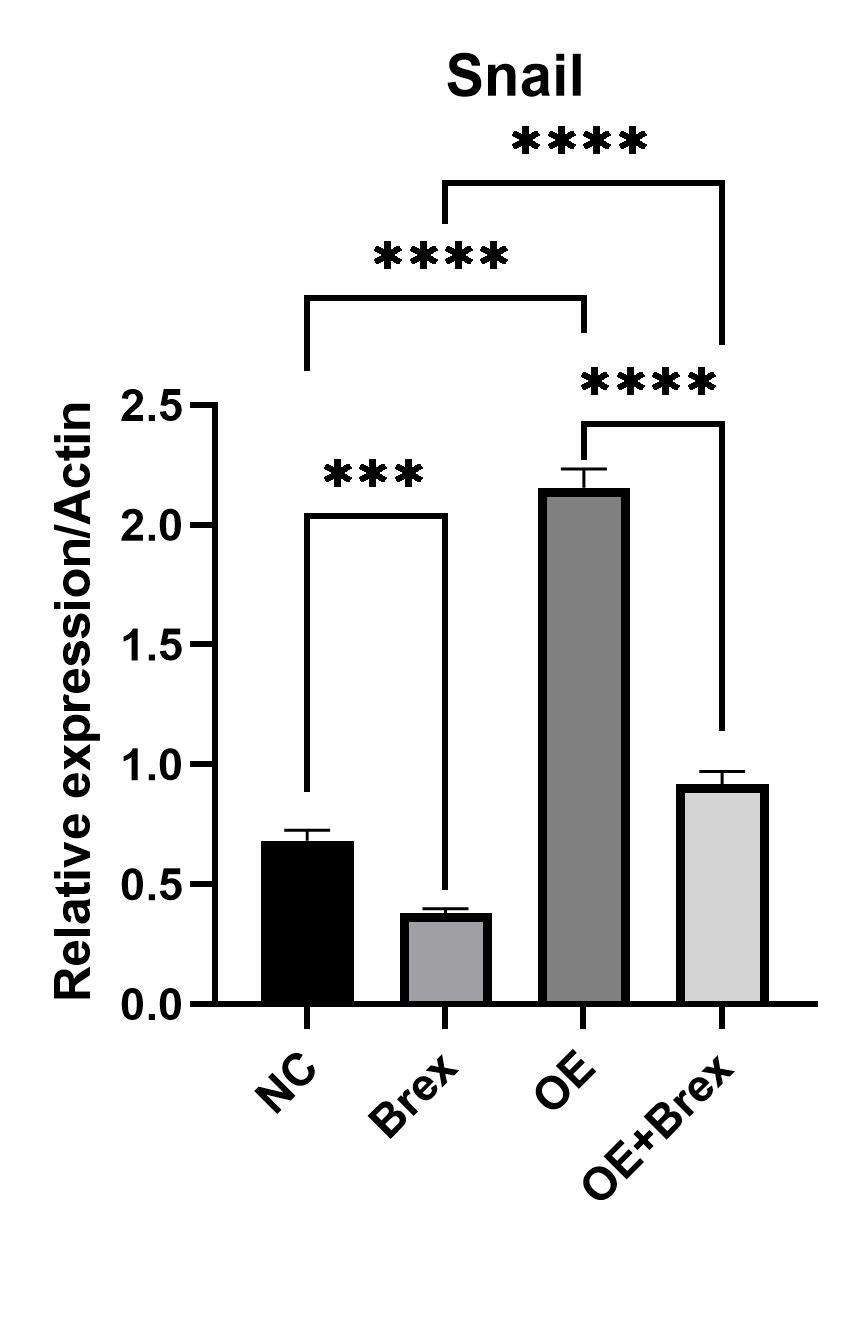

Supplement: Supplementary file 2 [file SupplementaryFile2.zip › WB数据/116-oe/数据图/SNAIL.tif]

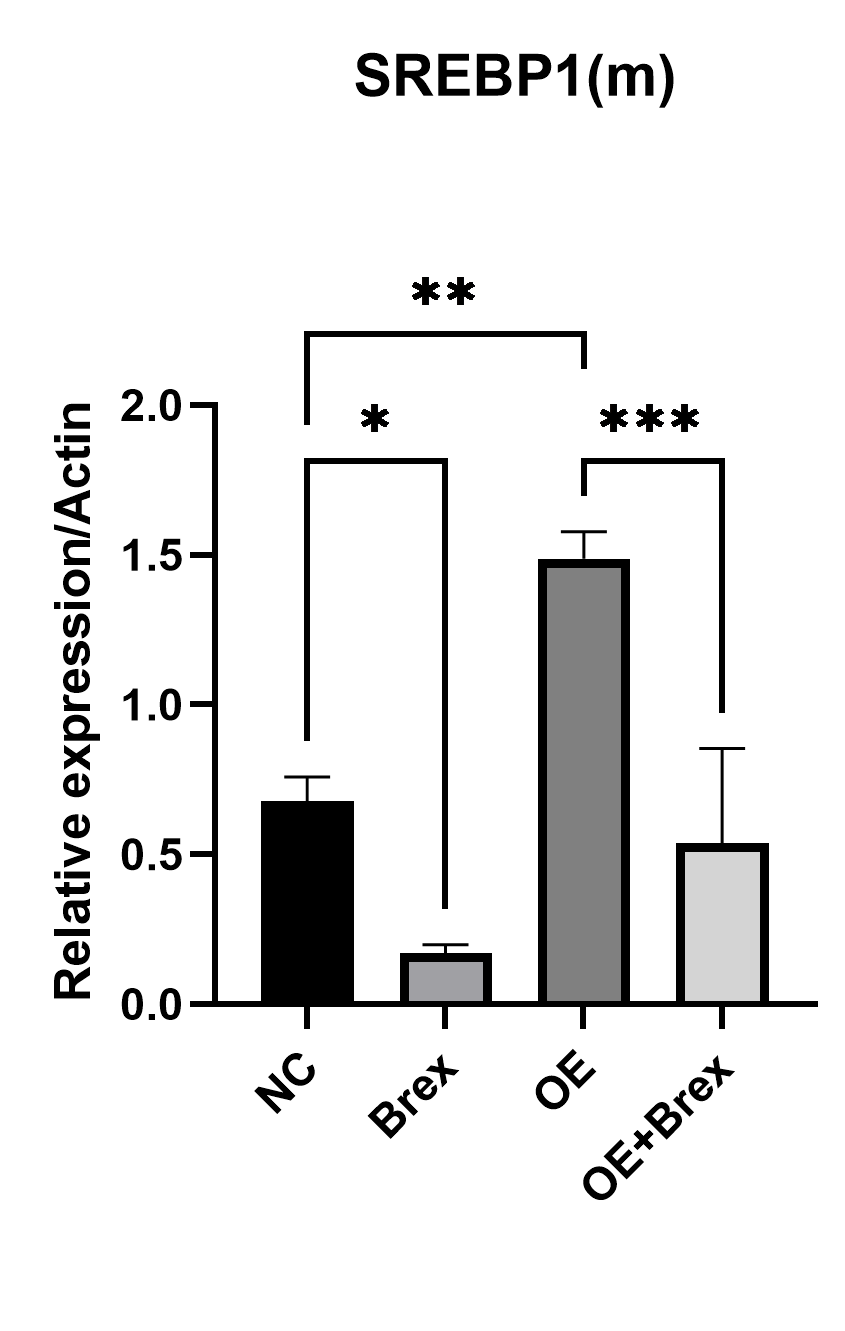

Supplement: Supplementary file 2 [file SupplementaryFile2.zip › WB数据/116-oe/数据图/SREBP1.tif]

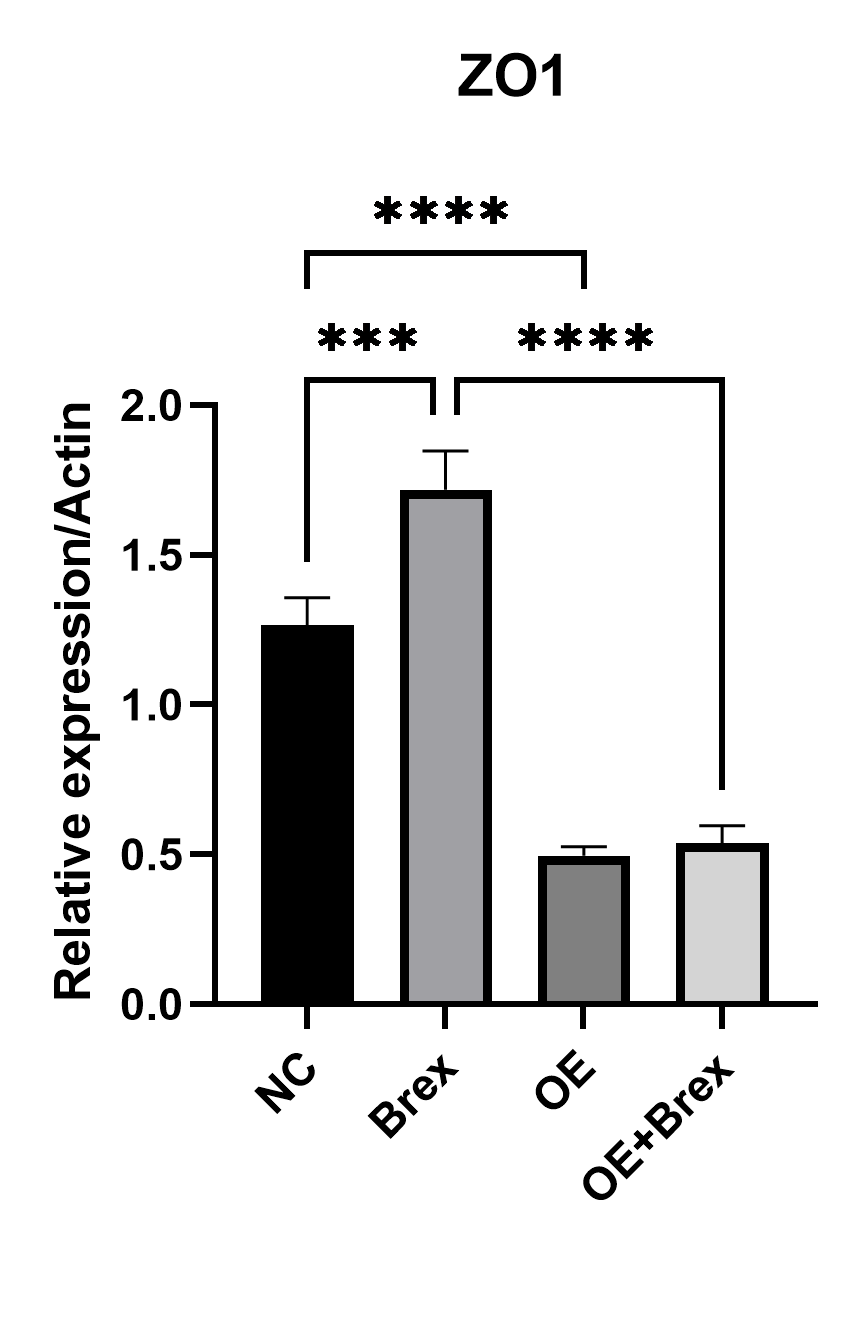

Supplement: Supplementary file 2 [file SupplementaryFile2.zip › WB数据/116-oe/数据图/ZO1.tif]

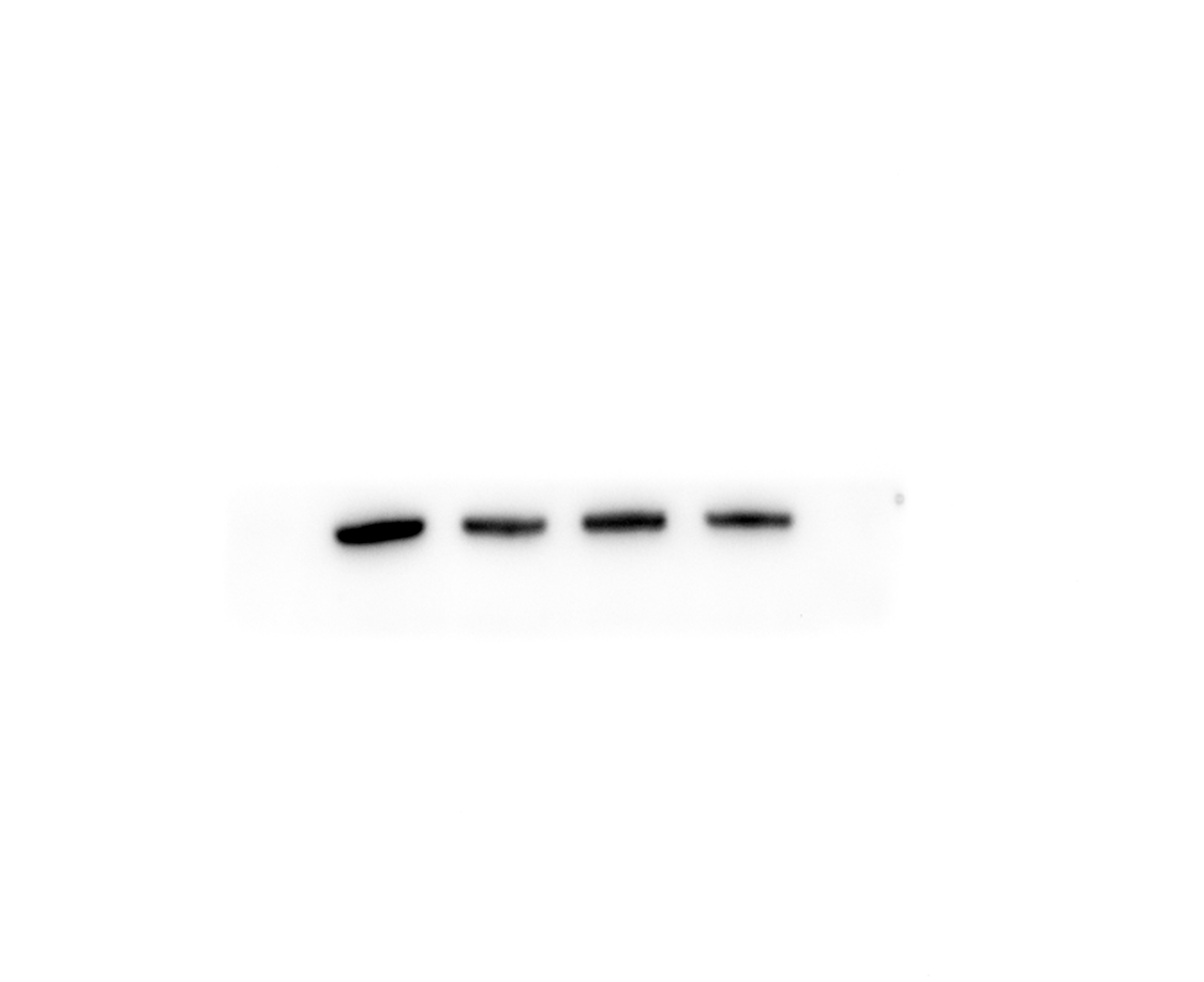

Supplement: Supplementary file 2 [file SupplementaryFile2.zip › WB数据/116-si/actin/116-actin-.tif]

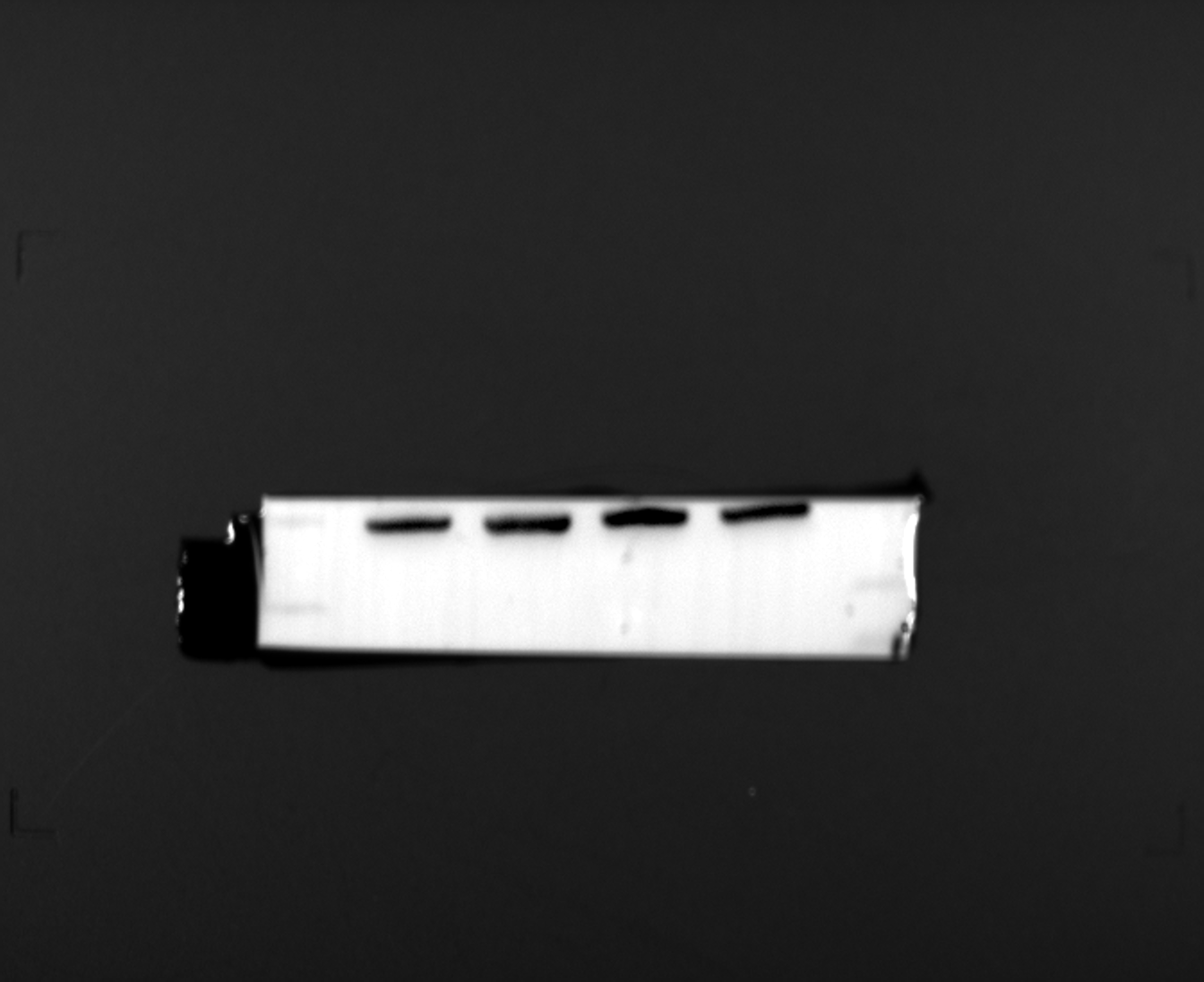

Supplement: Supplementary file 2 [file SupplementaryFile2.zip › WB数据/116-si/actin/116-actin-merge`.tif]

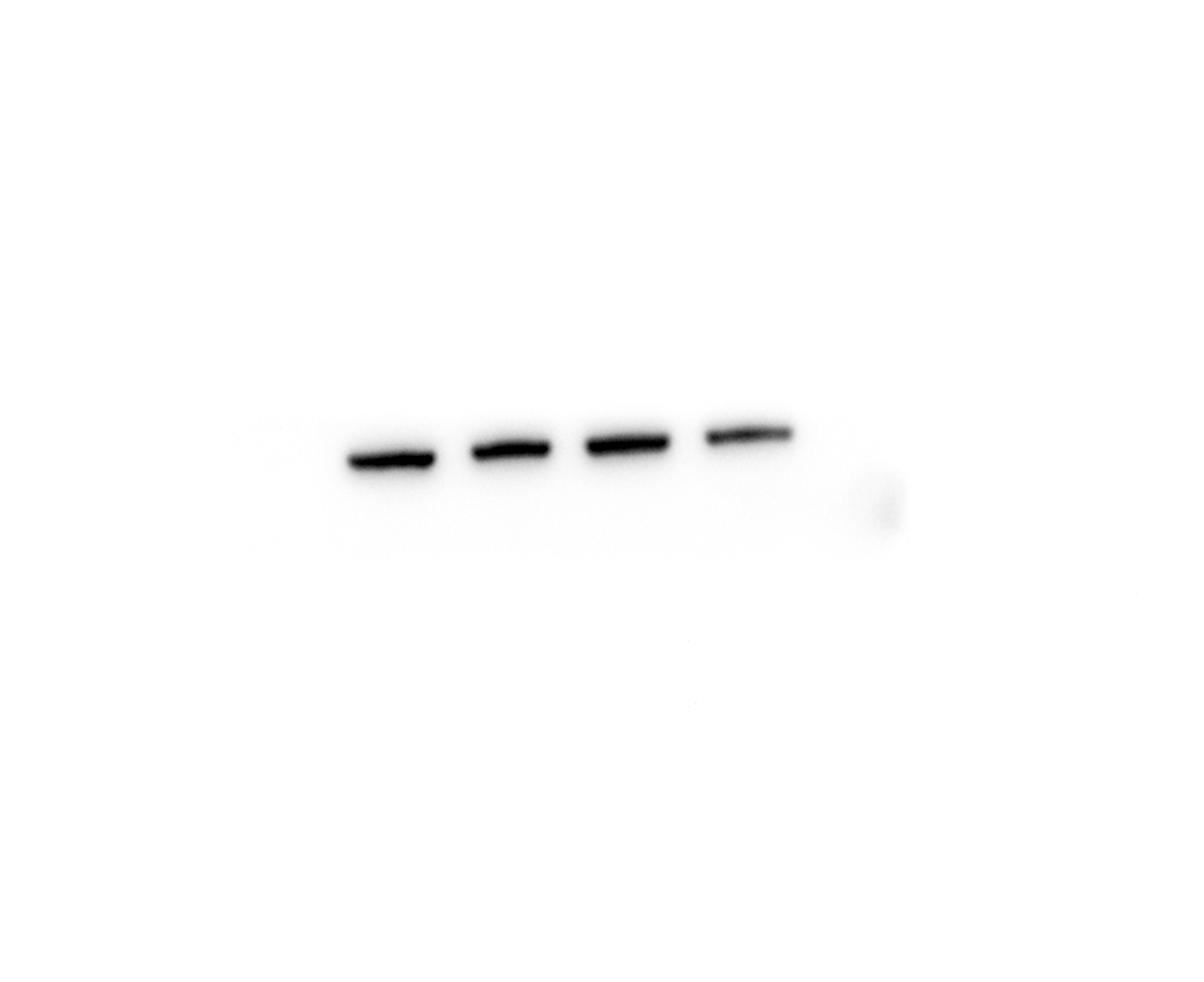

Supplement: Supplementary file 2 [file SupplementaryFile2.zip › WB数据/116-si/actin/116-actin.tif]

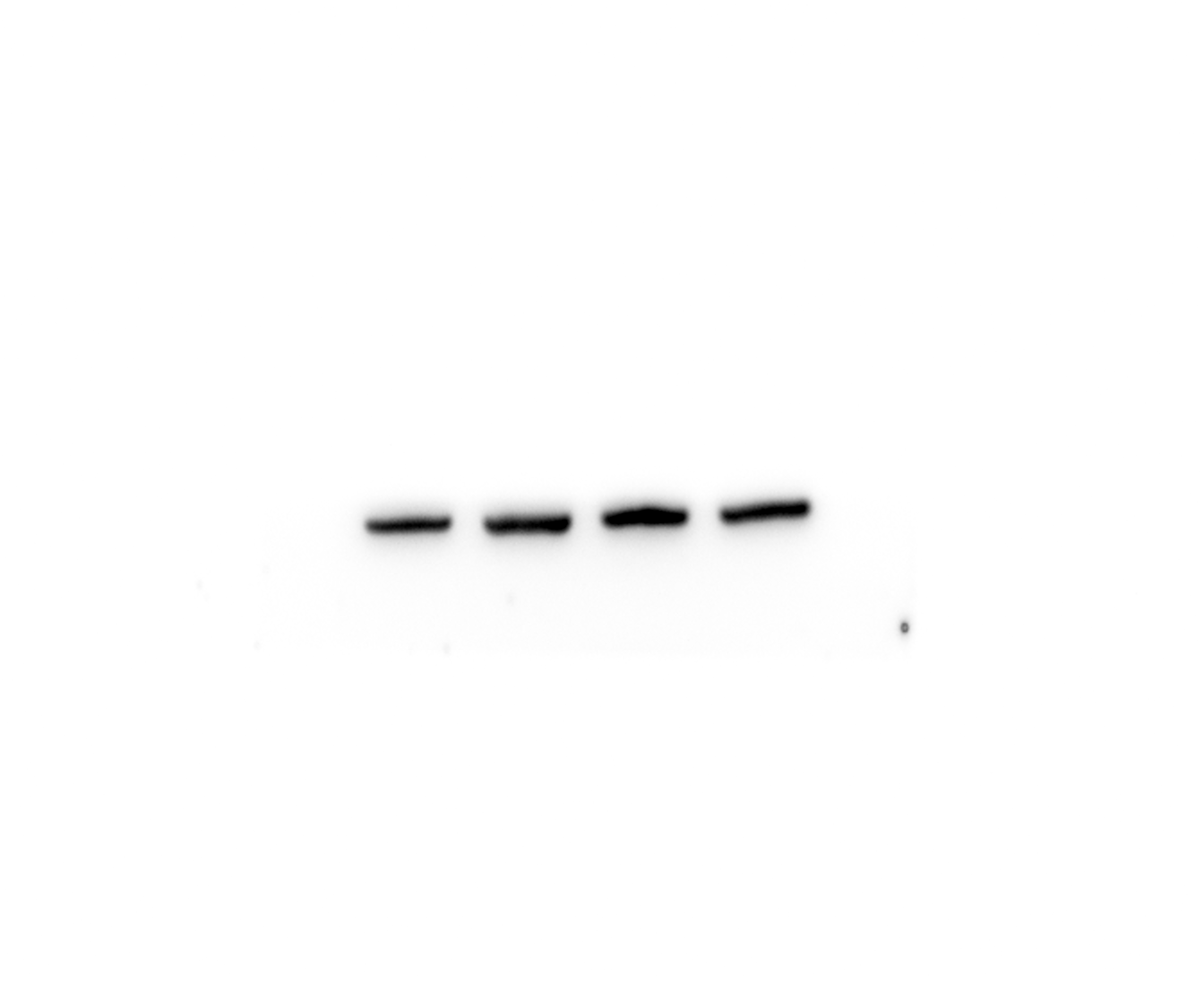

Supplement: Supplementary file 2 [file SupplementaryFile2.zip › WB数据/116-si/actin/116-actin`.tif]

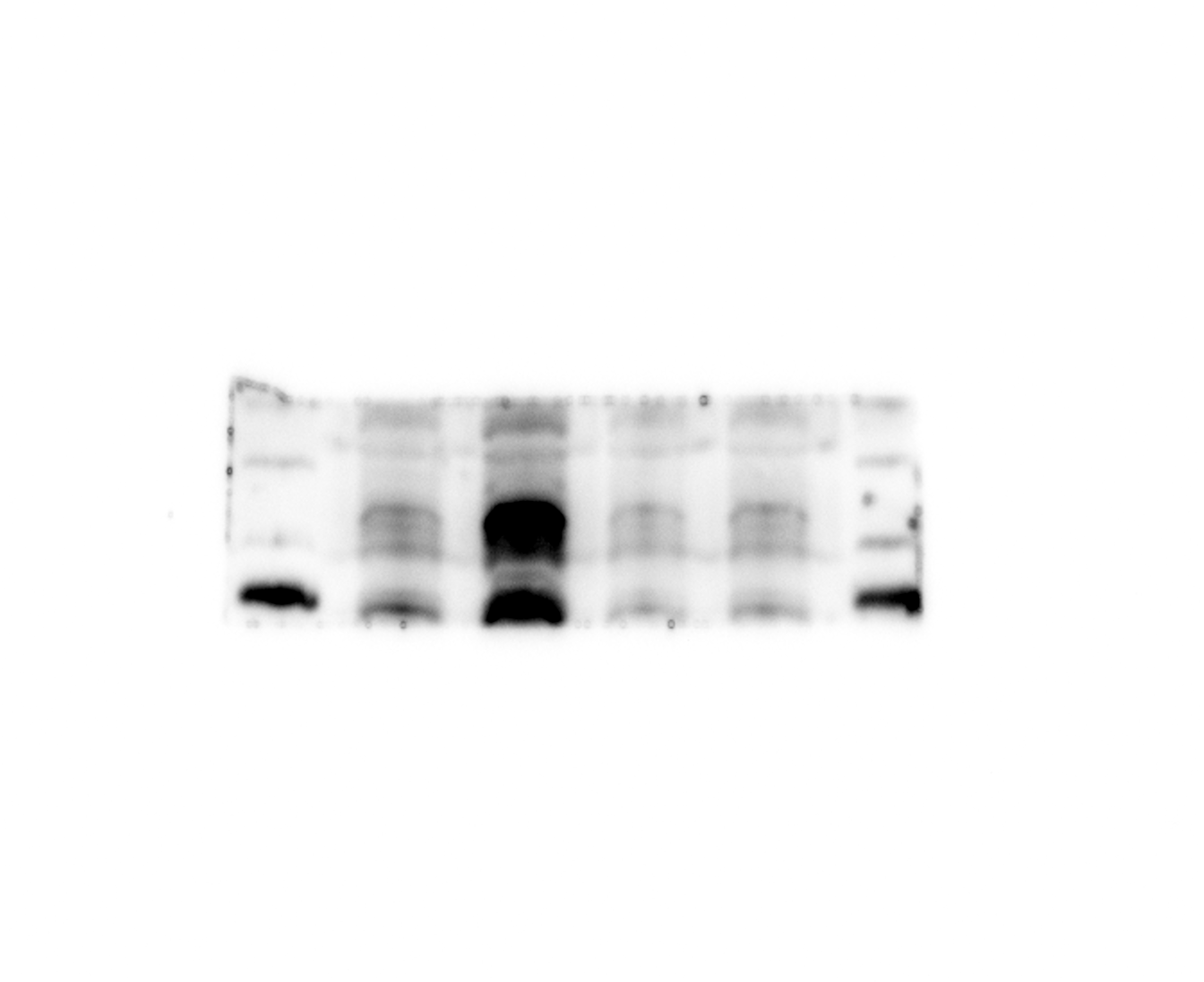

Supplement: Supplementary file 2 [file SupplementaryFile2.zip › WB数据/116-si/snail1/116-snai1--.tif]

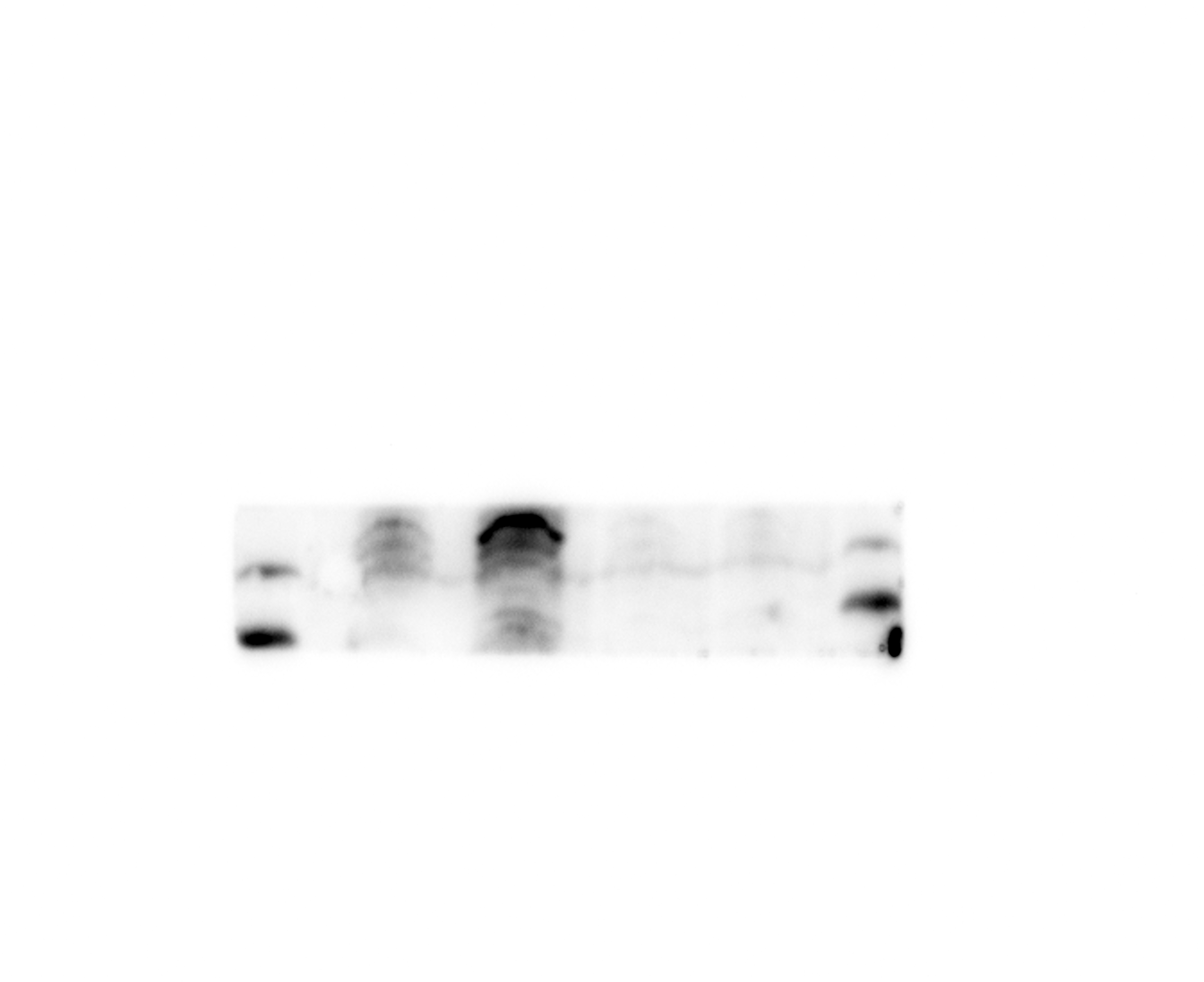

Supplement: Supplementary file 2 [file SupplementaryFile2.zip › WB数据/116-si/snail1/116-snai1-`.tif]

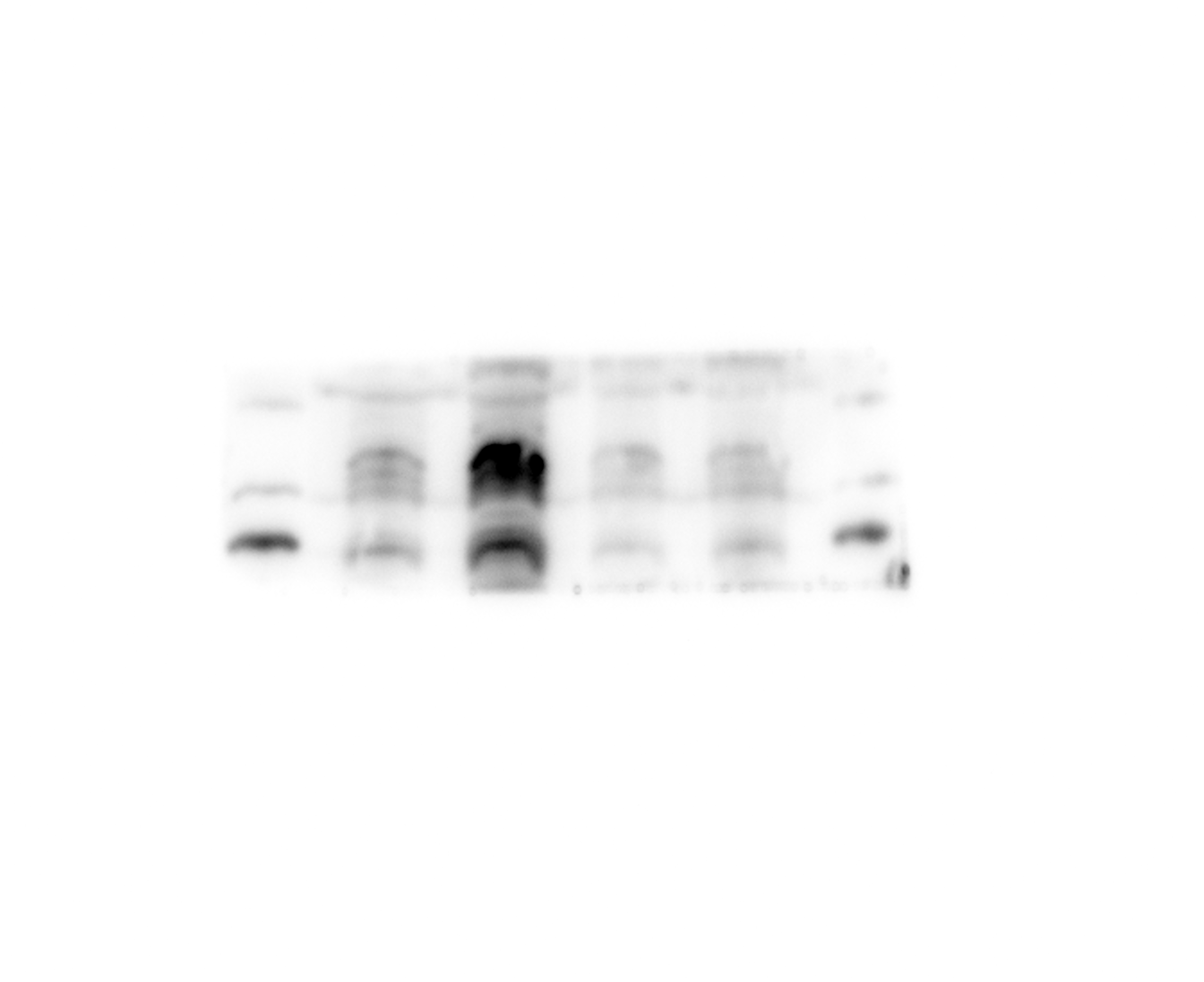

Supplement: Supplementary file 2 [file SupplementaryFile2.zip › WB数据/116-si/snail1/116-snai1.tif]

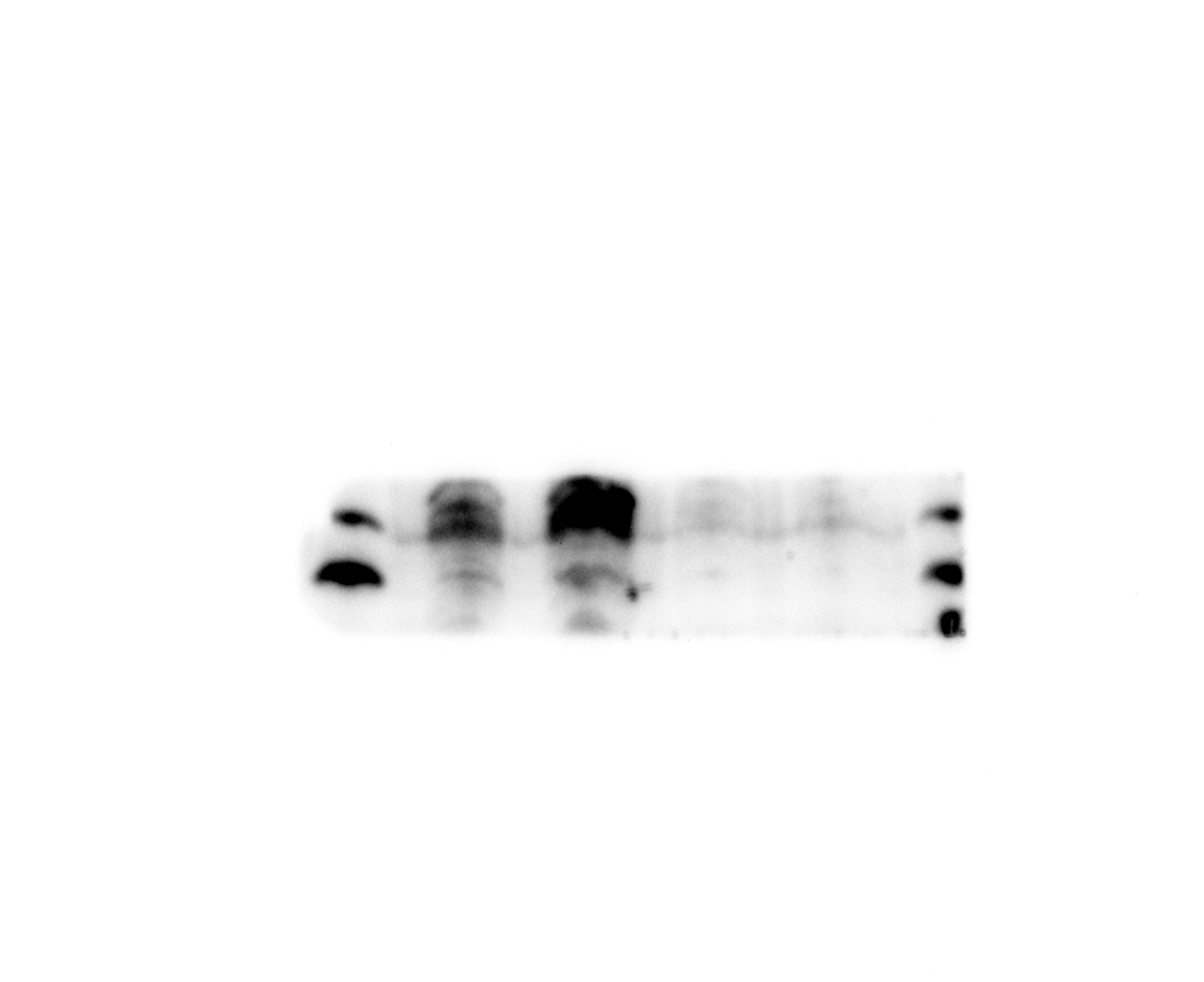

Supplement: Supplementary file 2 [file SupplementaryFile2.zip › WB数据/116-si/snail1/116-snai1`.tif]

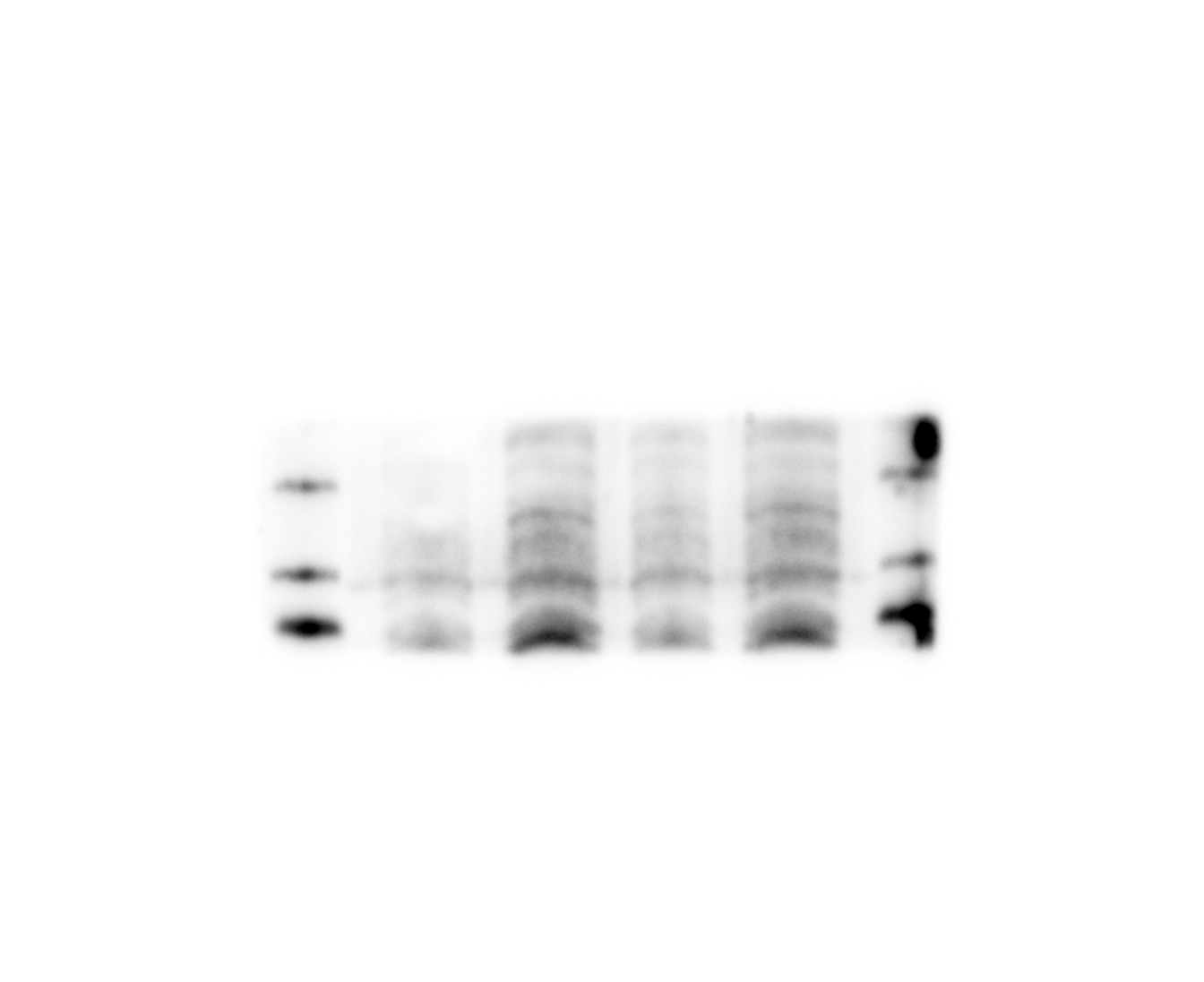

Supplement: Supplementary file 2 [file SupplementaryFile2.zip › WB数据/116-si/srebp1/116-sr1----.tif]

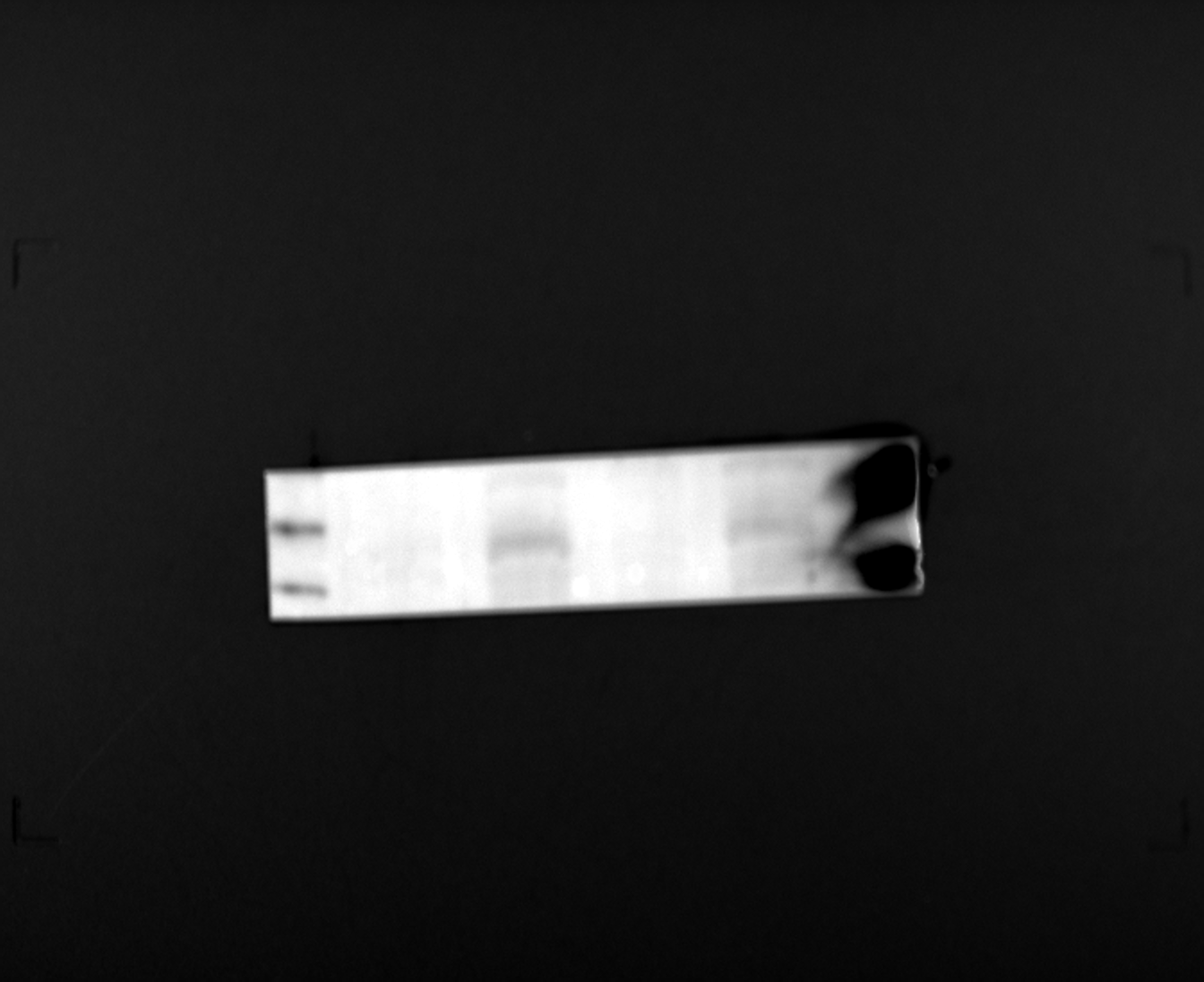

Supplement: Supplementary file 2 [file SupplementaryFile2.zip › WB数据/116-si/srebp1/116-sr1-merge`.tif]

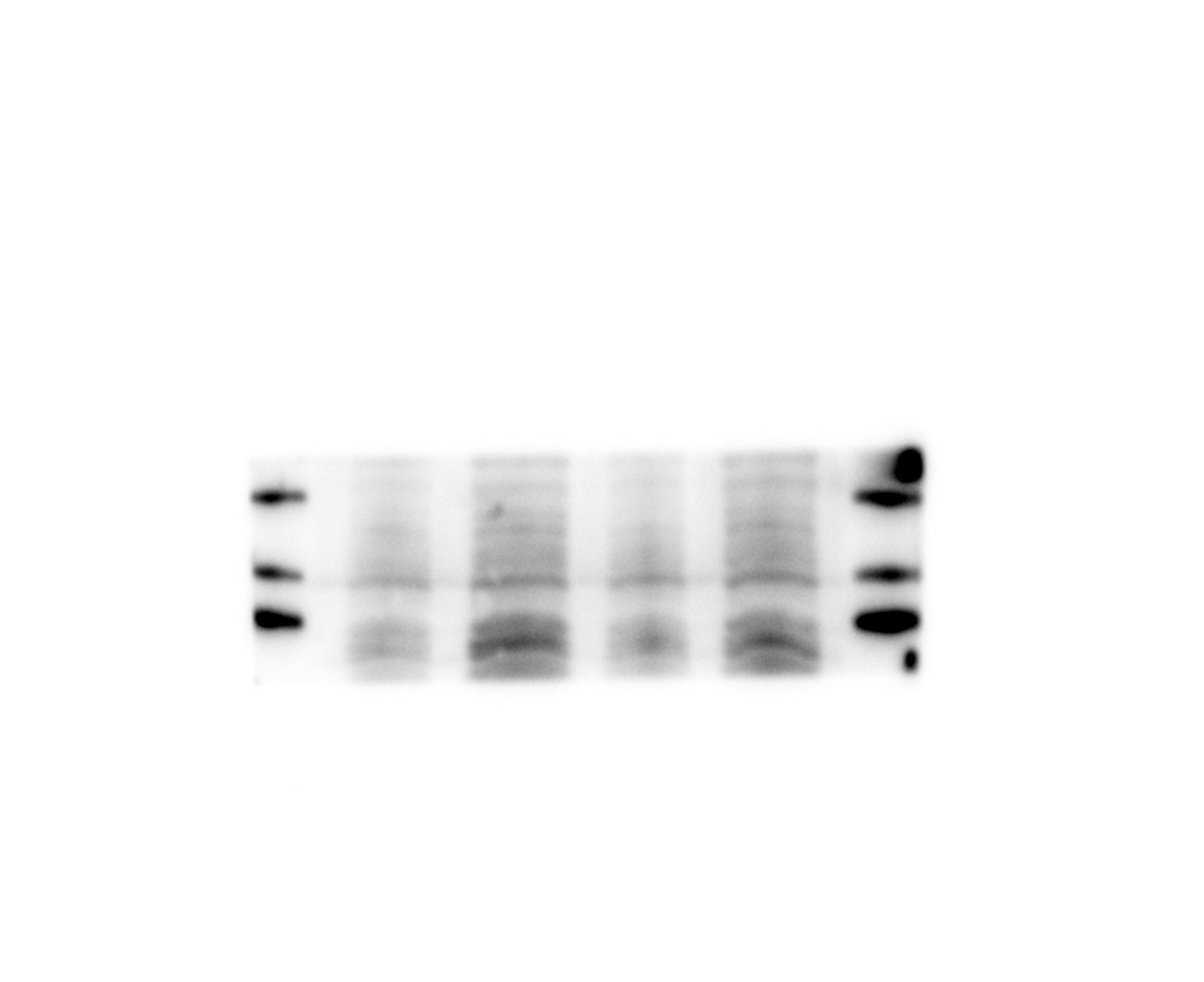

Supplement: Supplementary file 2 [file SupplementaryFile2.zip › WB数据/116-si/srebp1/116-sr1.tif]

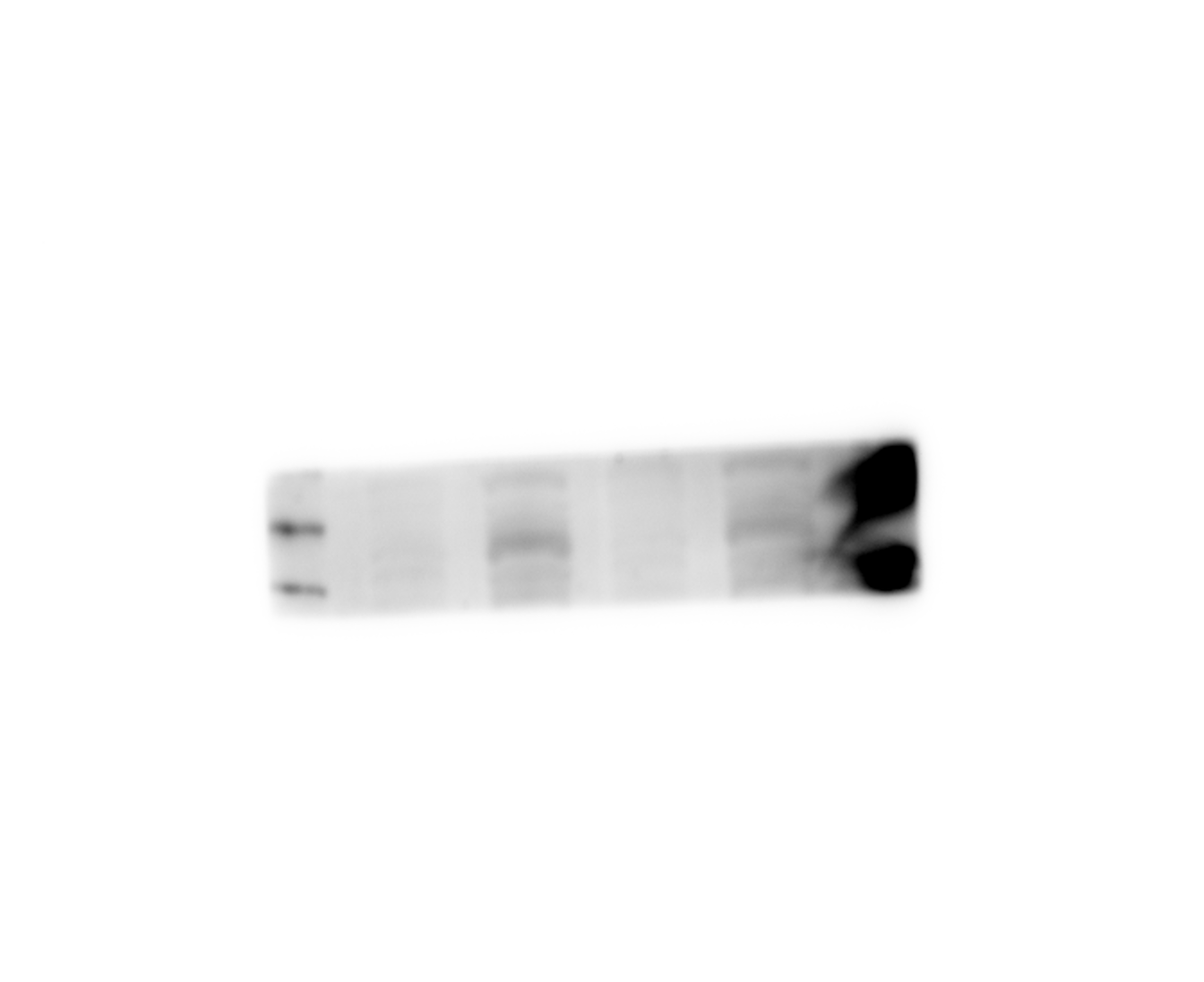

Supplement: Supplementary file 2 [file SupplementaryFile2.zip › WB数据/116-si/srebp1/116-sr1`.tif]

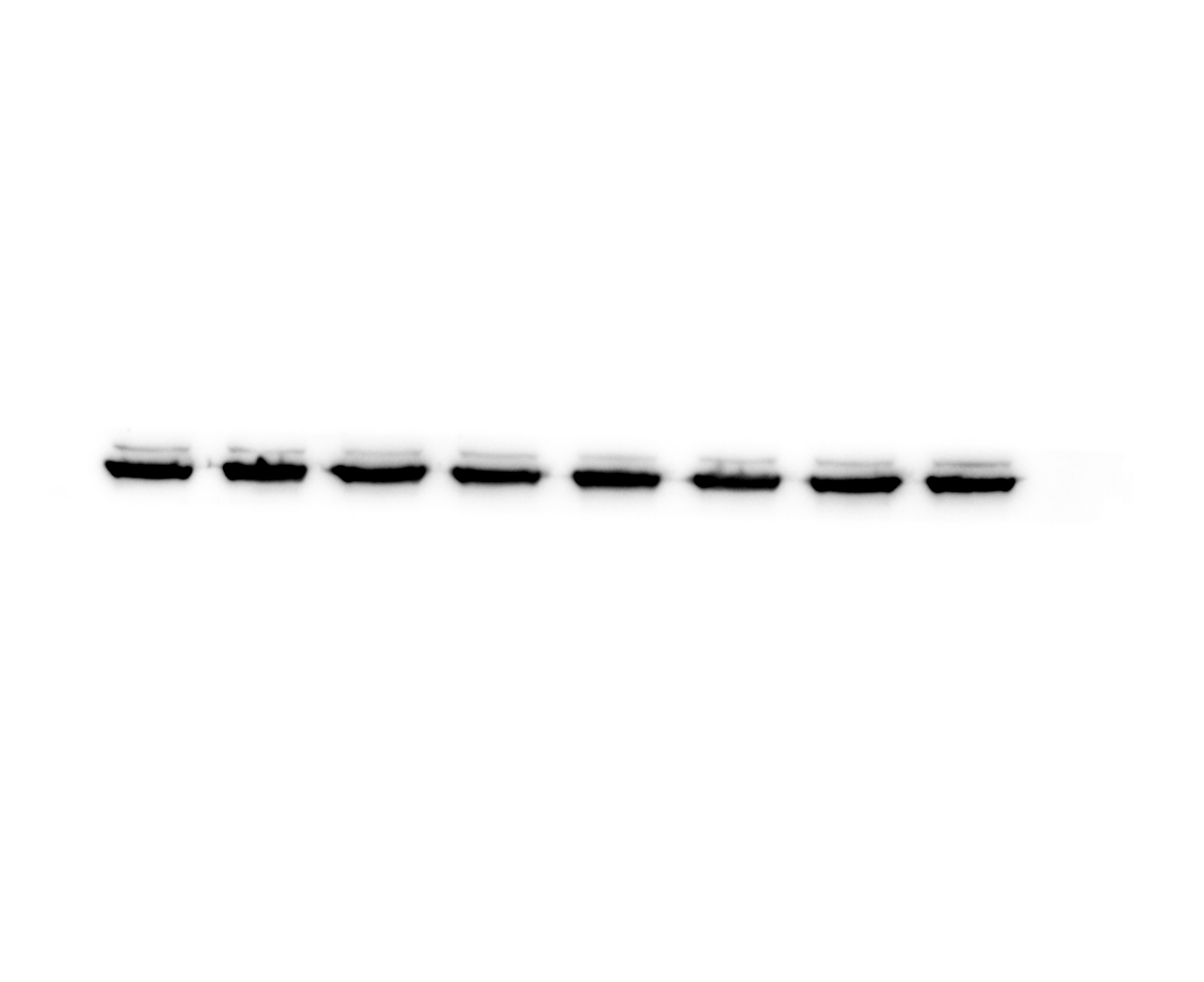

Supplement: Supplementary file 2 [file SupplementaryFile2.zip › WB数据/620/620-wb/actin/620-actin---.tif]

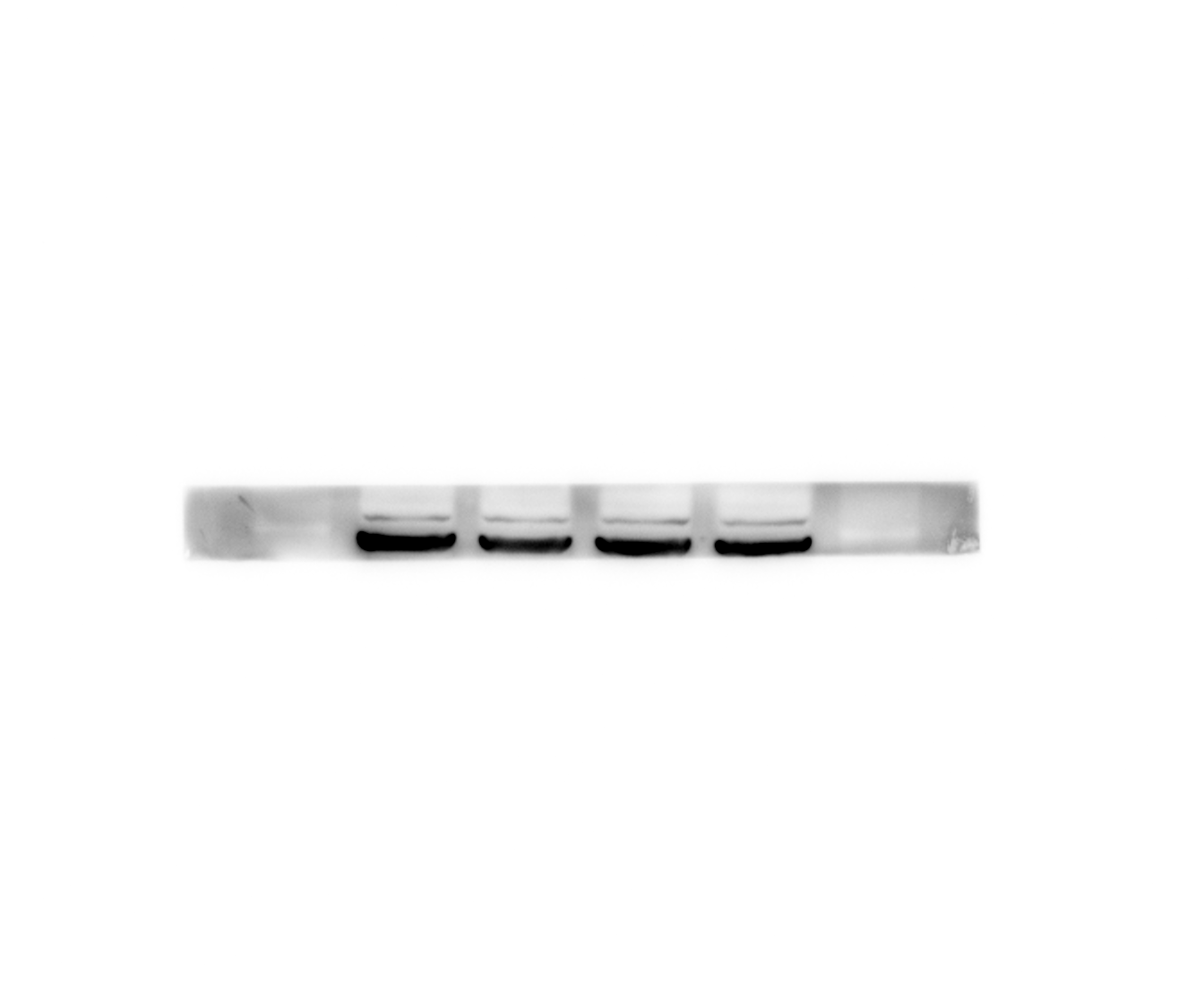

Supplement: Supplementary file 2 [file SupplementaryFile2.zip › WB数据/620/620-wb/actin/620-actin-.tif]

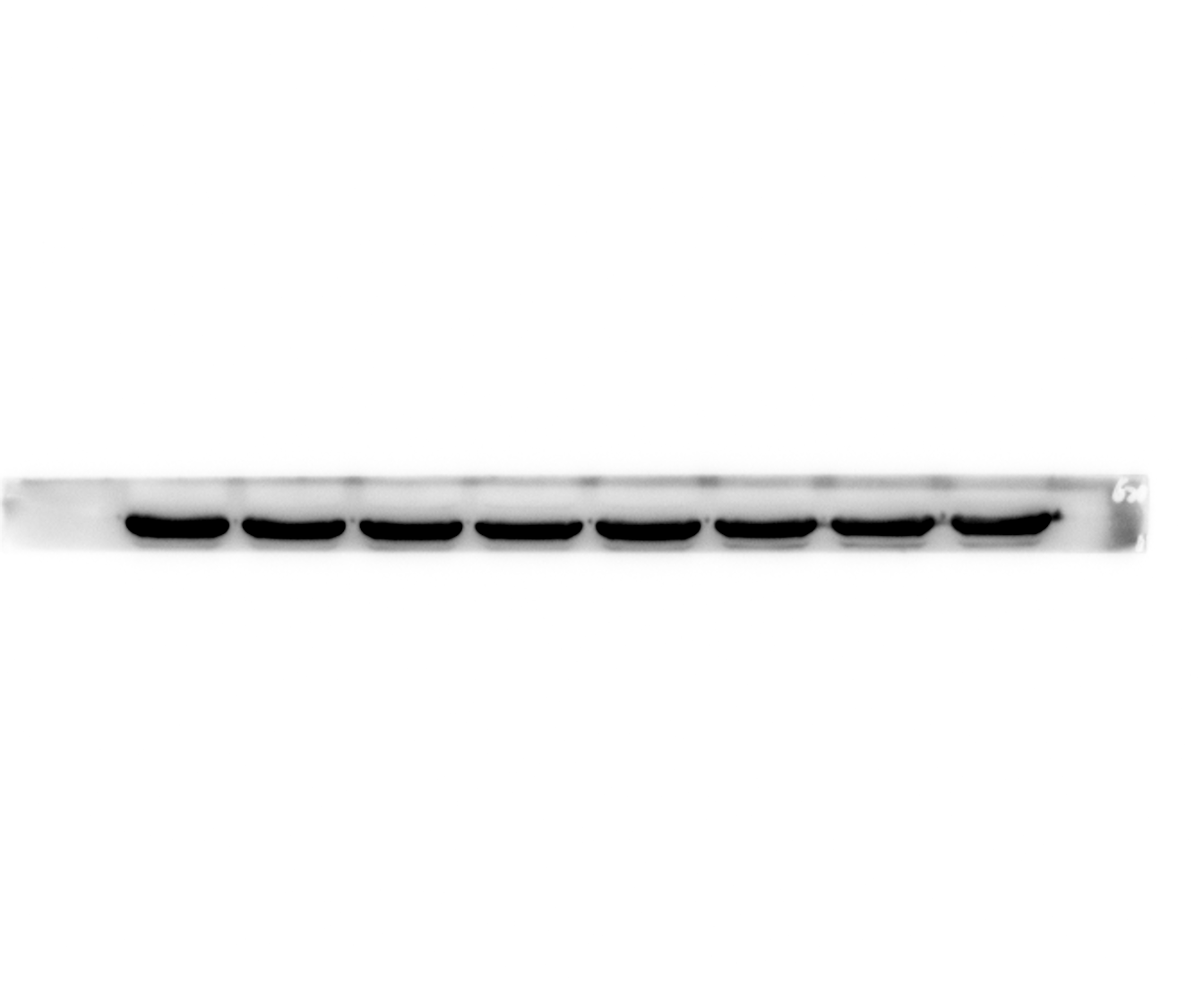

Supplement: Supplementary file 2 [file SupplementaryFile2.zip › WB数据/620/620-wb/actin/620-actin.tif]

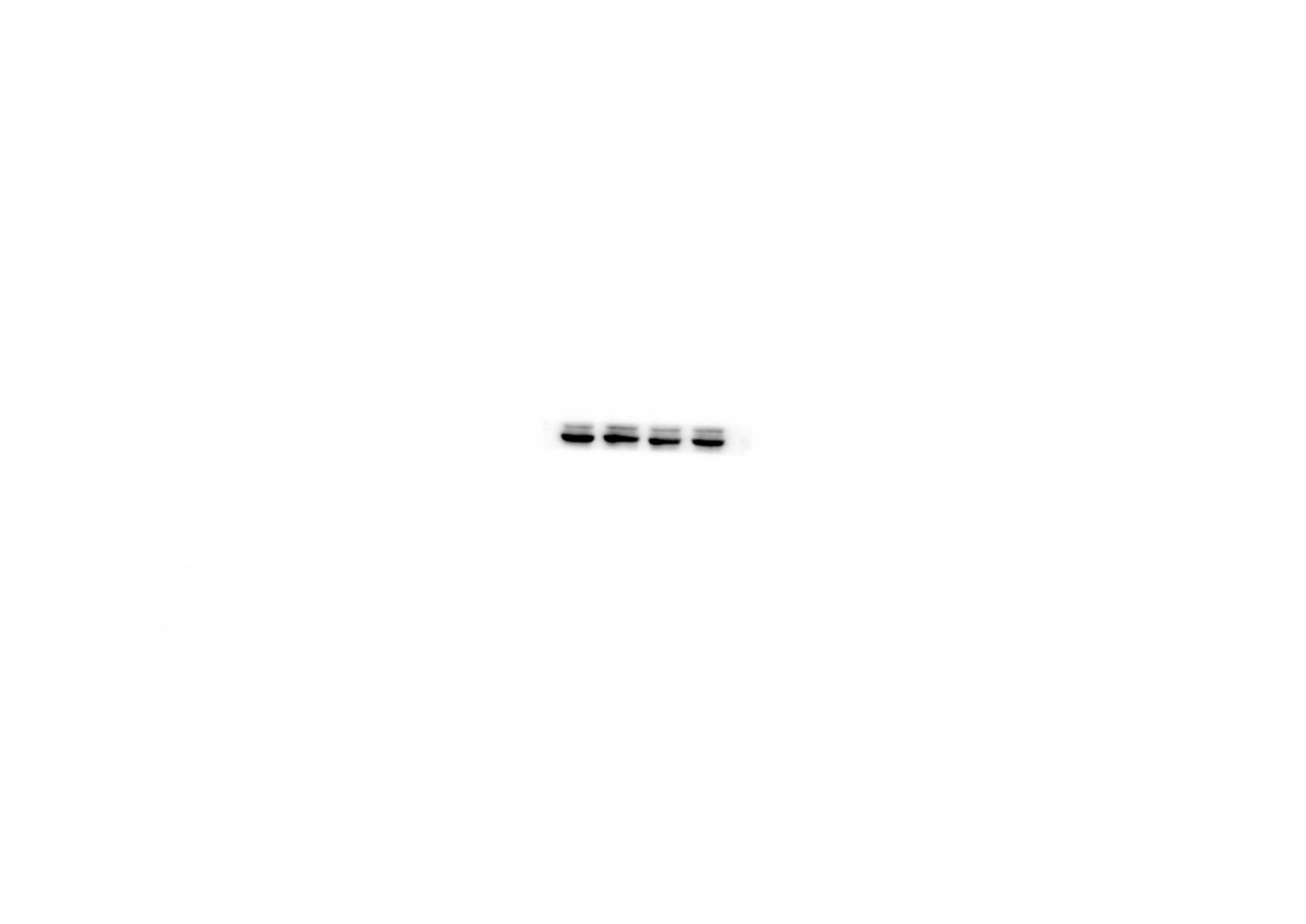

Supplement: Supplementary file 2 [file SupplementaryFile2.zip › WB数据/620/620-wb/actin/actin-_00.tif]

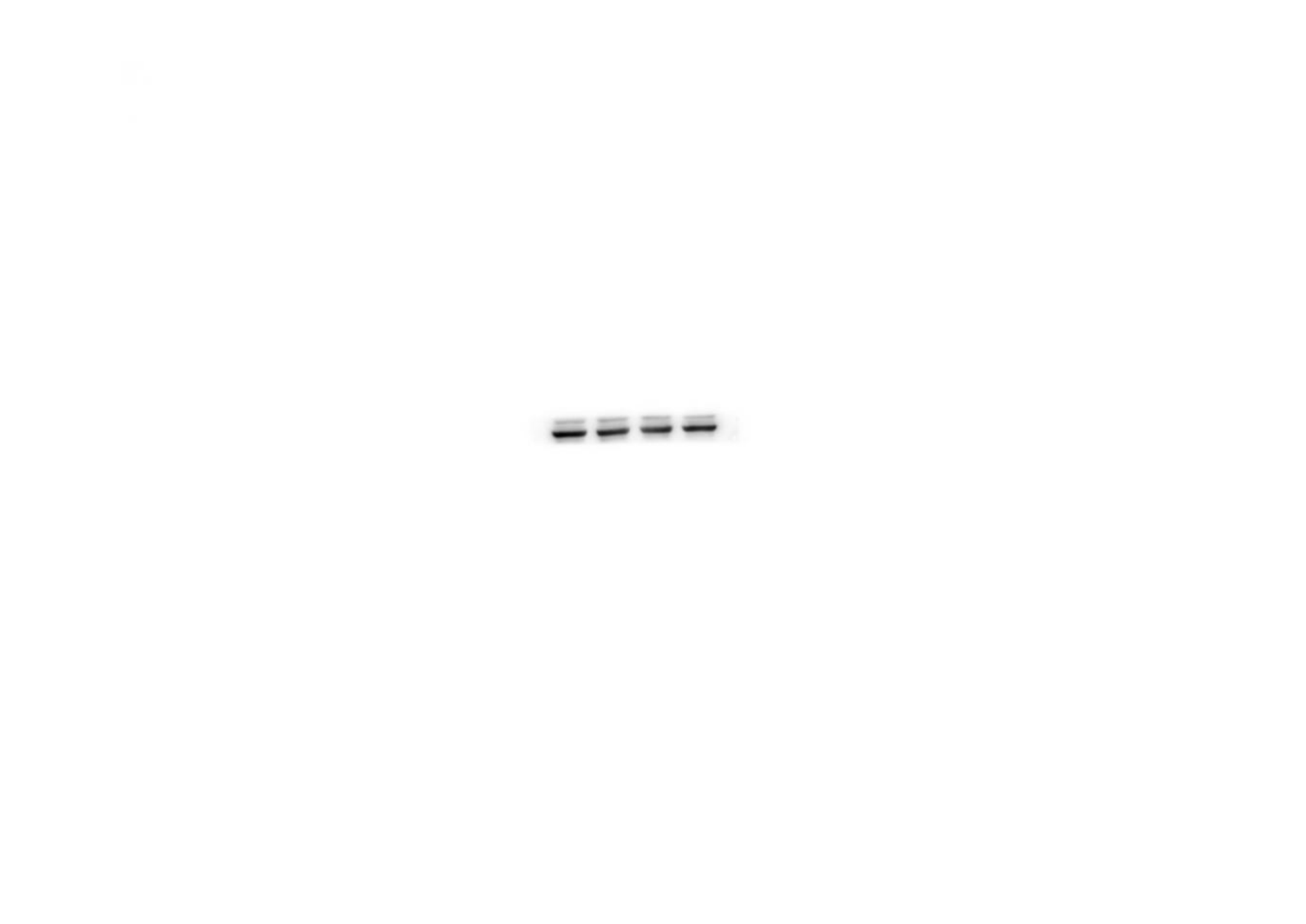

Supplement: Supplementary file 2 [file SupplementaryFile2.zip › WB数据/620/620-wb/actin/actin_00.tif]

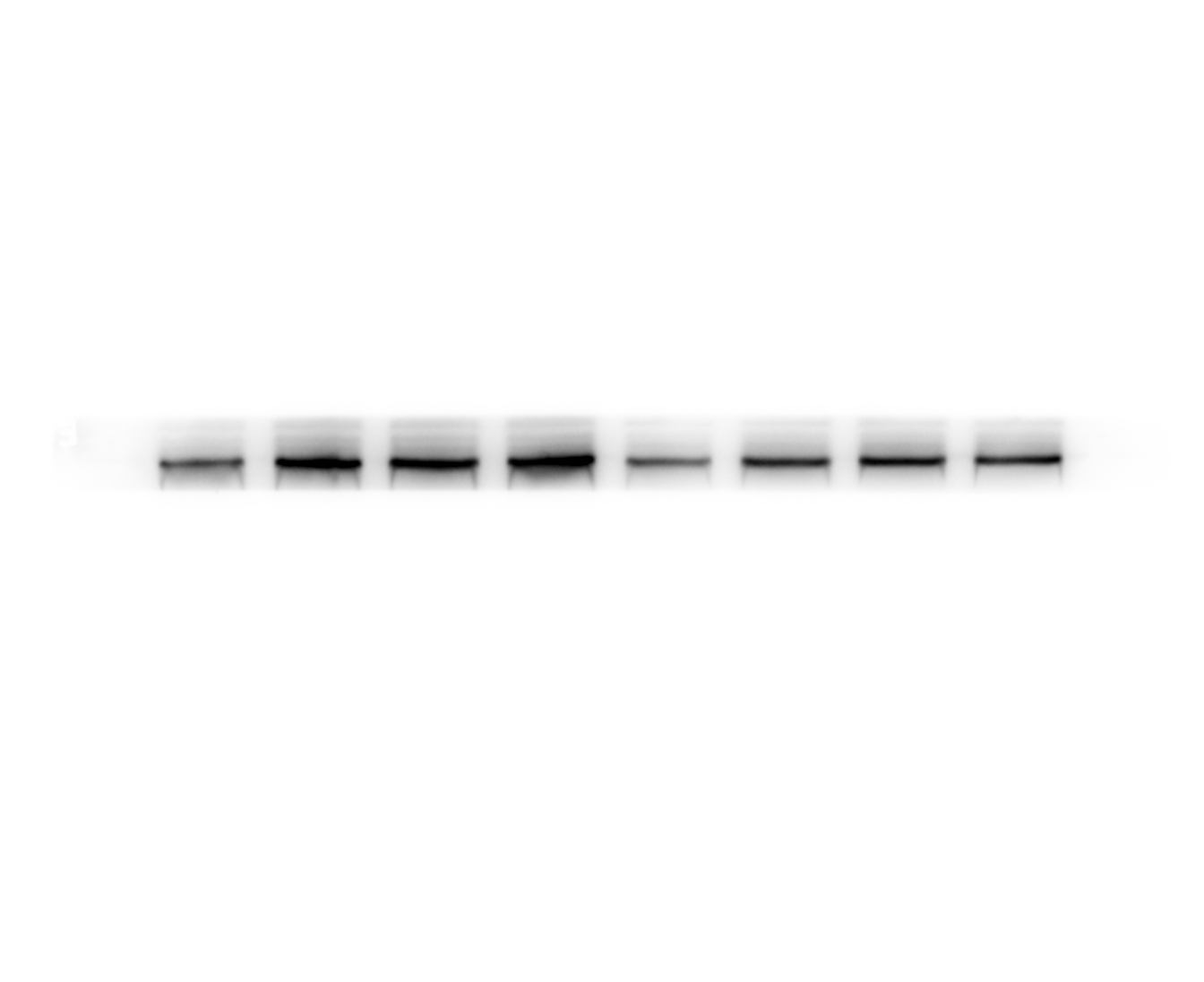

Supplement: Supplementary file 2 [file SupplementaryFile2.zip › WB数据/620/620-wb/e/620e-.tif]

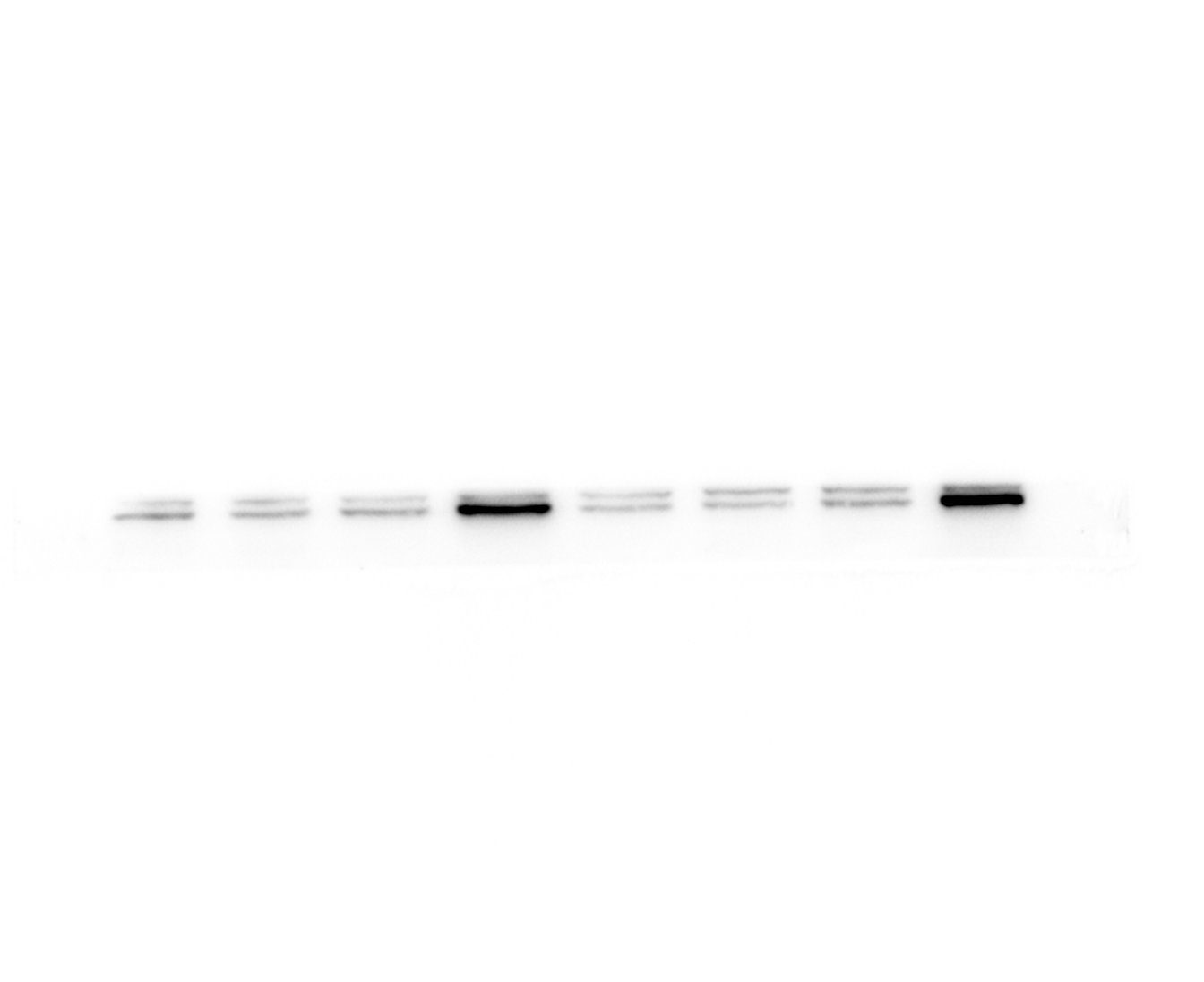

Supplement: Supplementary file 2 [file SupplementaryFile2.zip › WB数据/620/620-wb/e/e.tif]

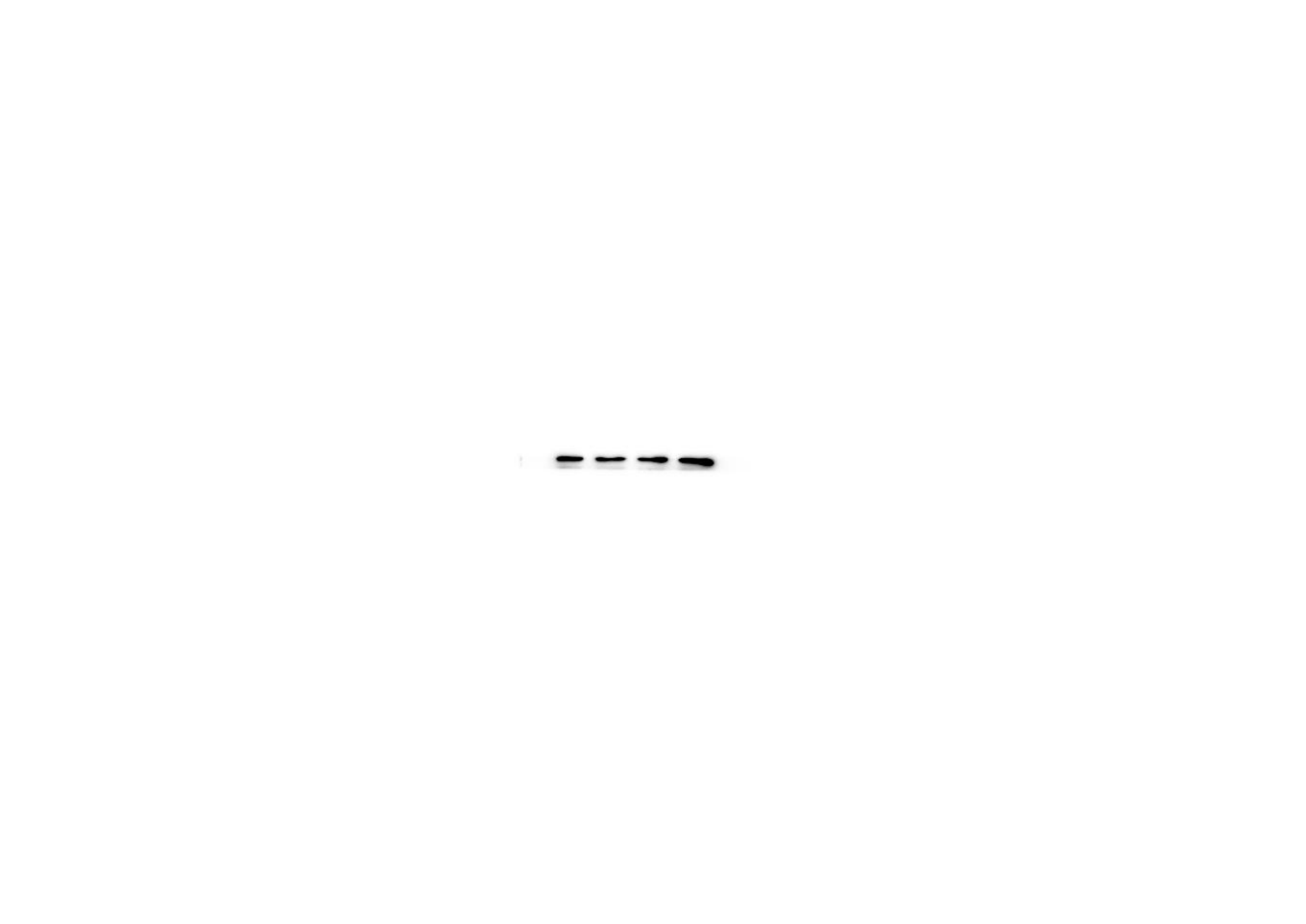

Supplement: Supplementary file 2 [file SupplementaryFile2.zip › WB数据/620/620-wb/e/e````_00.tif]

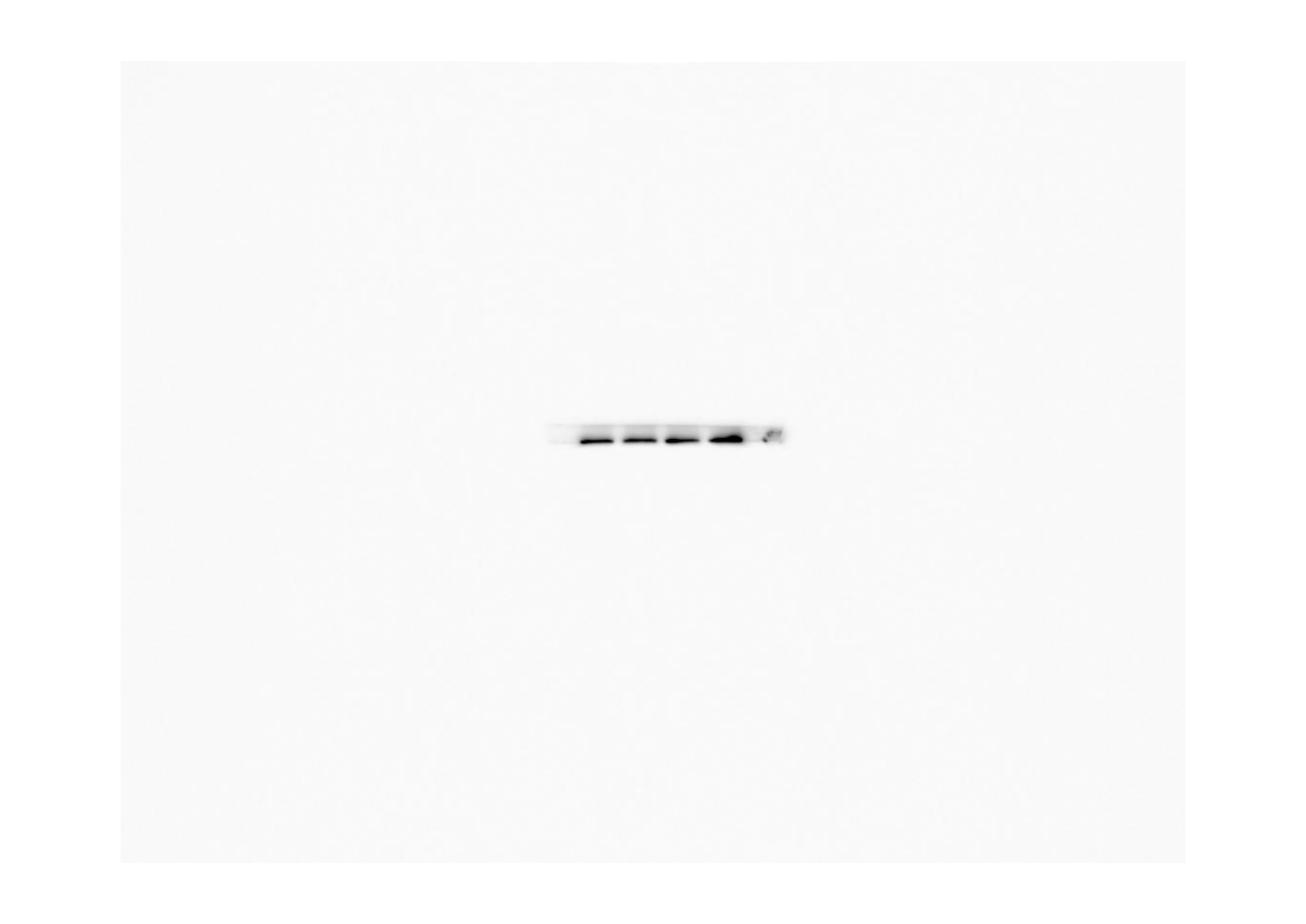

Supplement: Supplementary file 2 [file SupplementaryFile2.zip › WB数据/620/620-wb/e/e钙粘_00.tif]

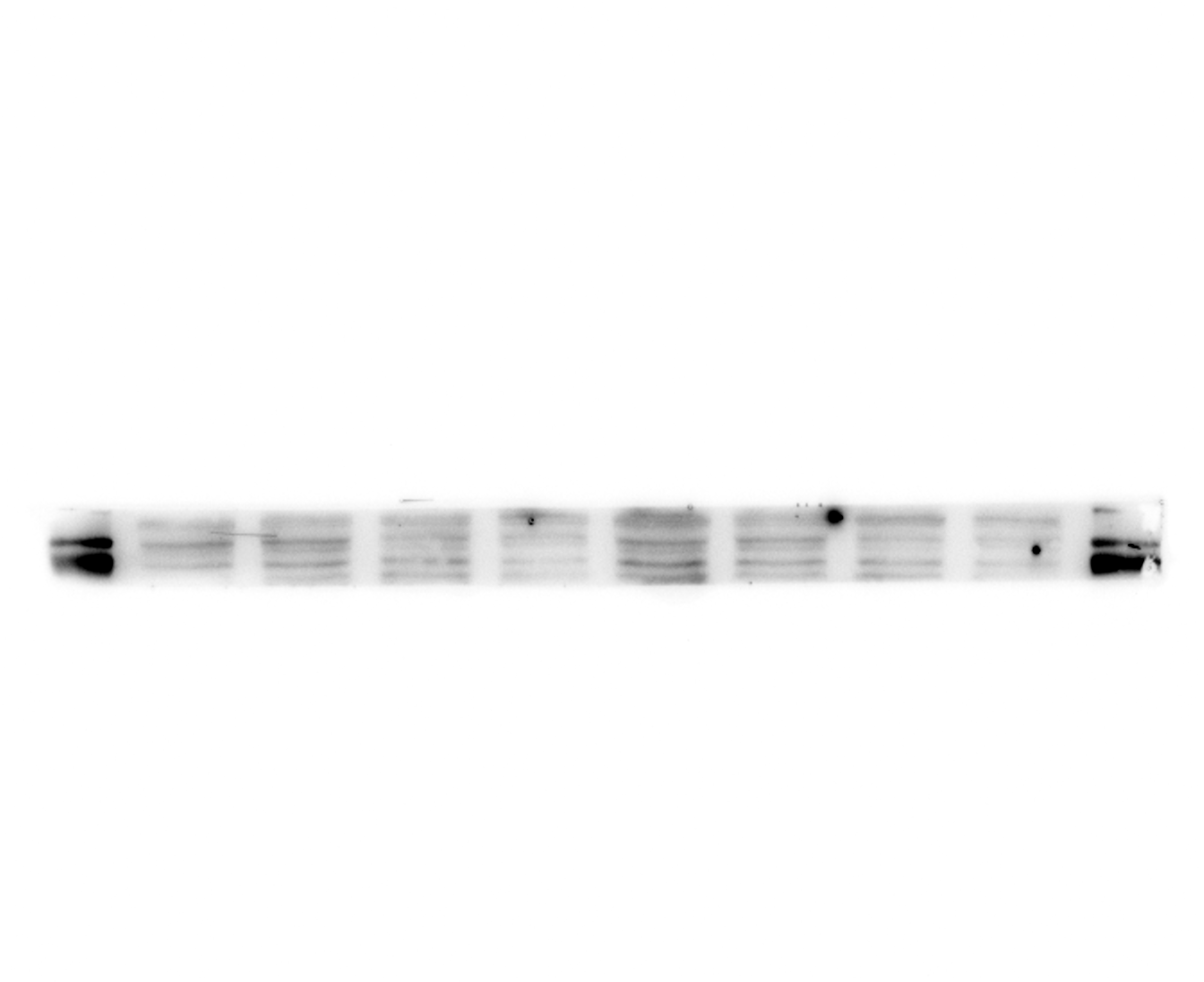

Supplement: Supplementary file 2 [file SupplementaryFile2.zip › WB数据/620/620-wb/mmp9/620-mmp9---------.tif]

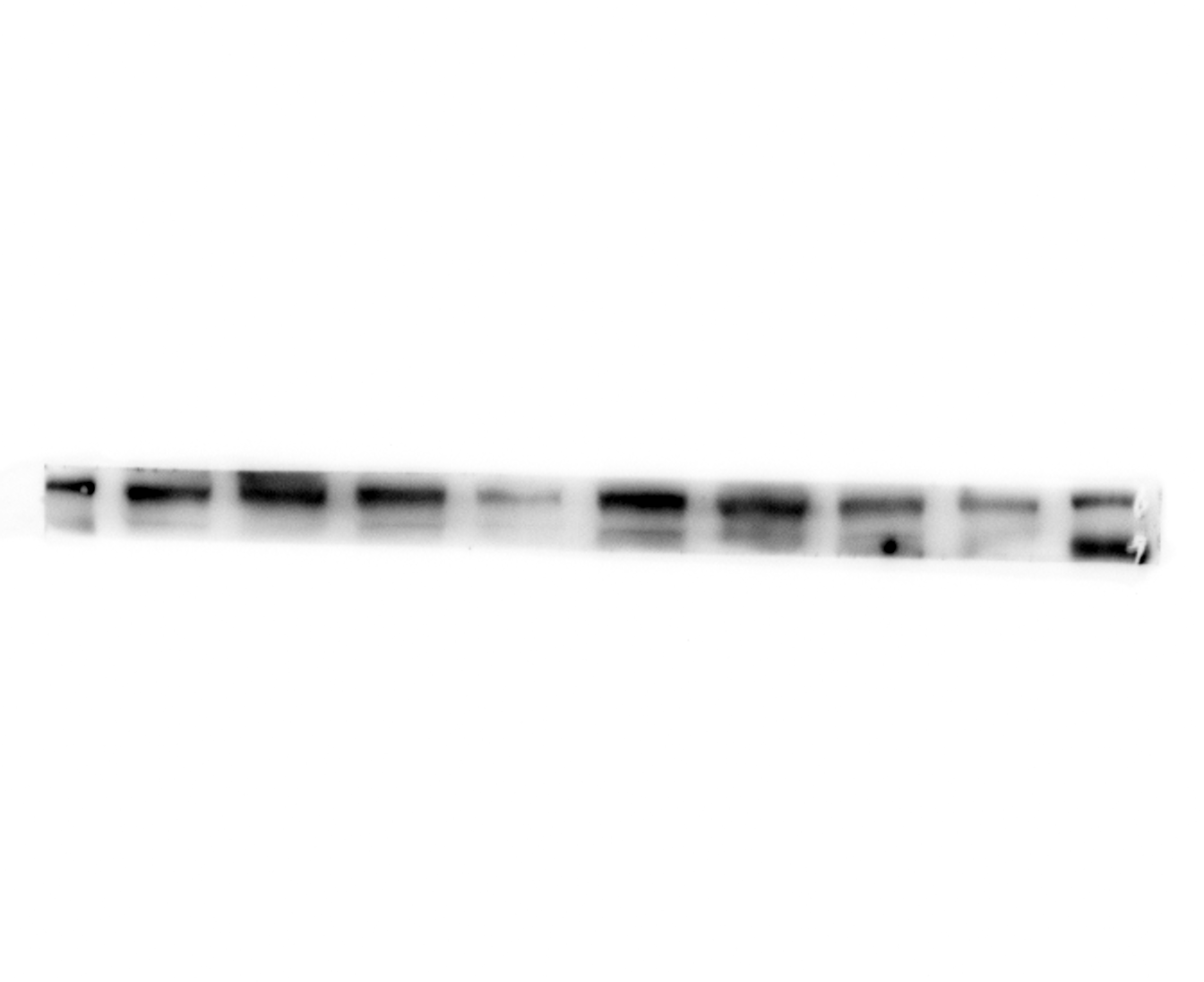

Supplement: Supplementary file 2 [file SupplementaryFile2.zip › WB数据/620/620-wb/mmp9/620-mmp9---.tif]

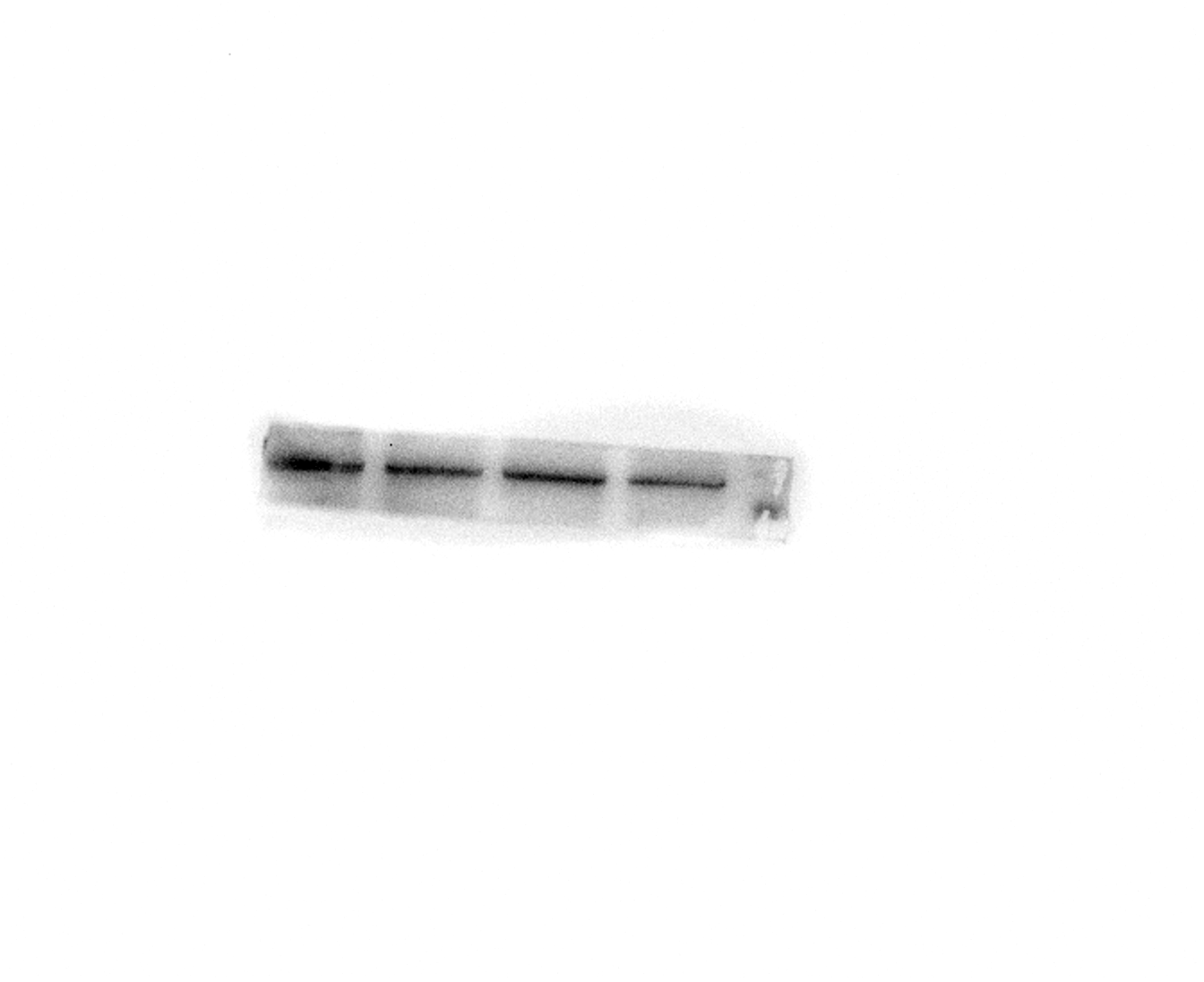

Supplement: Supplementary file 2 [file SupplementaryFile2.zip › WB数据/620/620-wb/mmp9/620-MMP9-.tif]

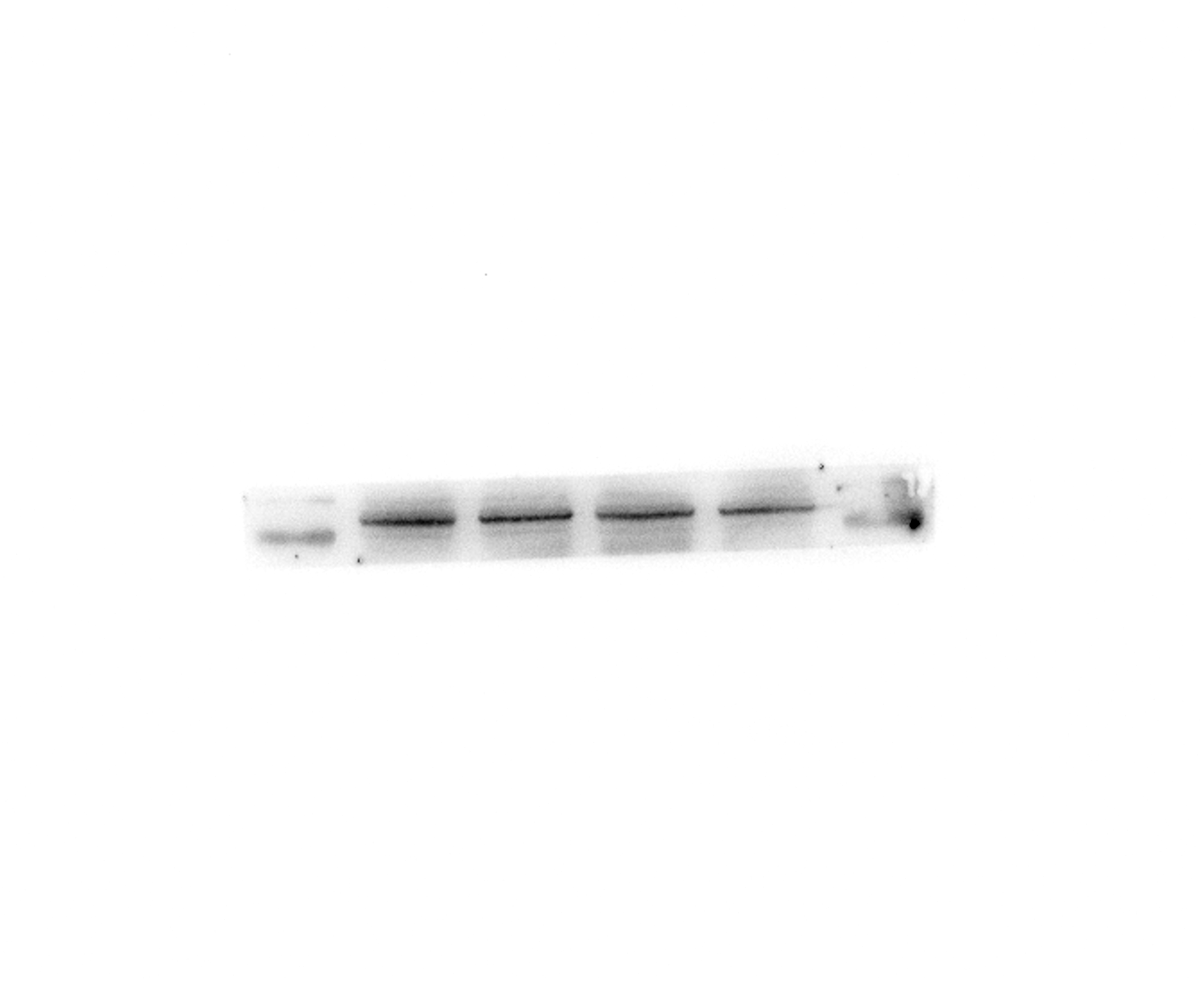

Supplement: Supplementary file 2 [file SupplementaryFile2.zip › WB数据/620/620-wb/mmp9/620-MMP9.tif]

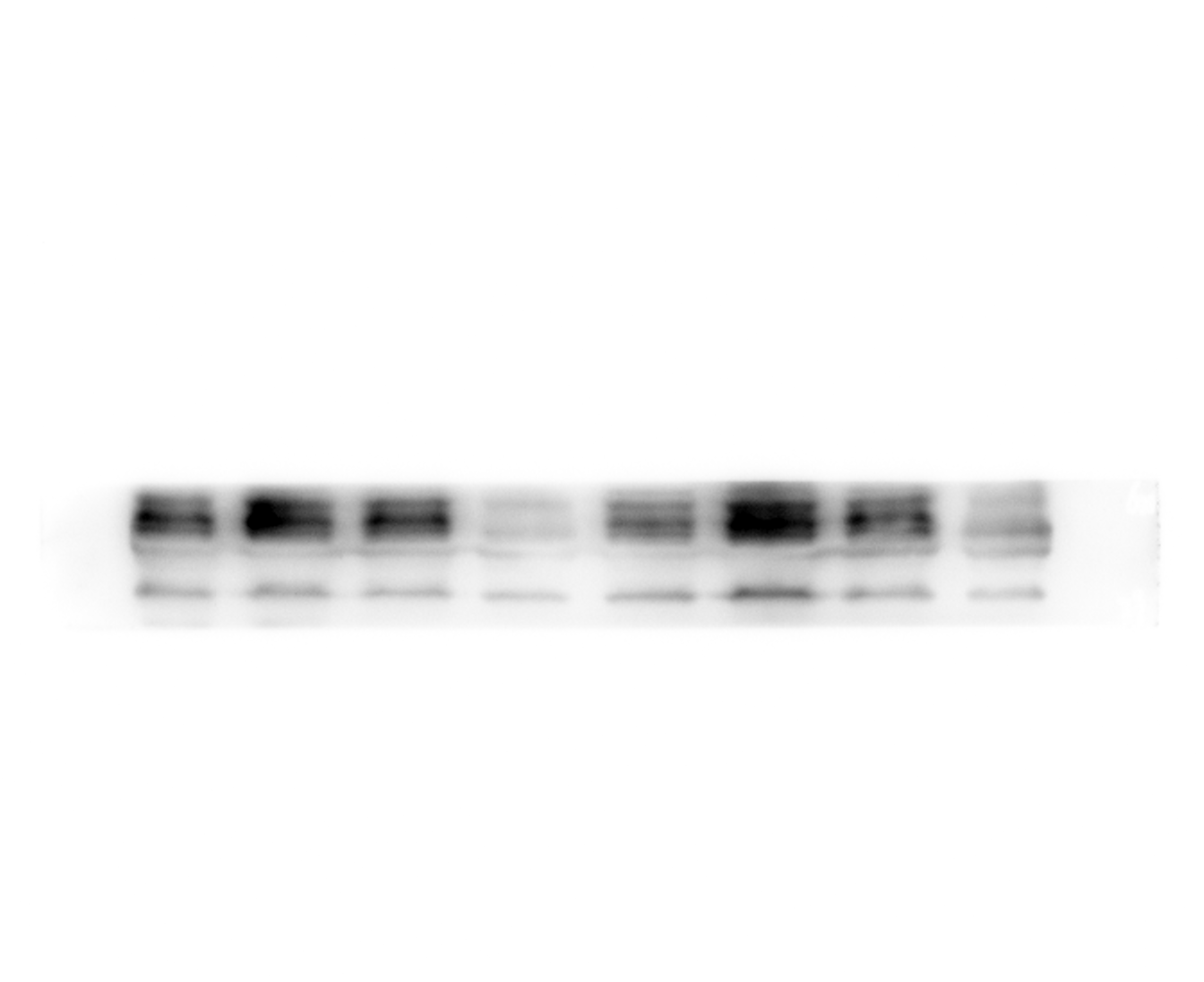

Supplement: Supplementary file 2 [file SupplementaryFile2.zip › WB数据/620/620-wb/snail/620-snail--.tif]

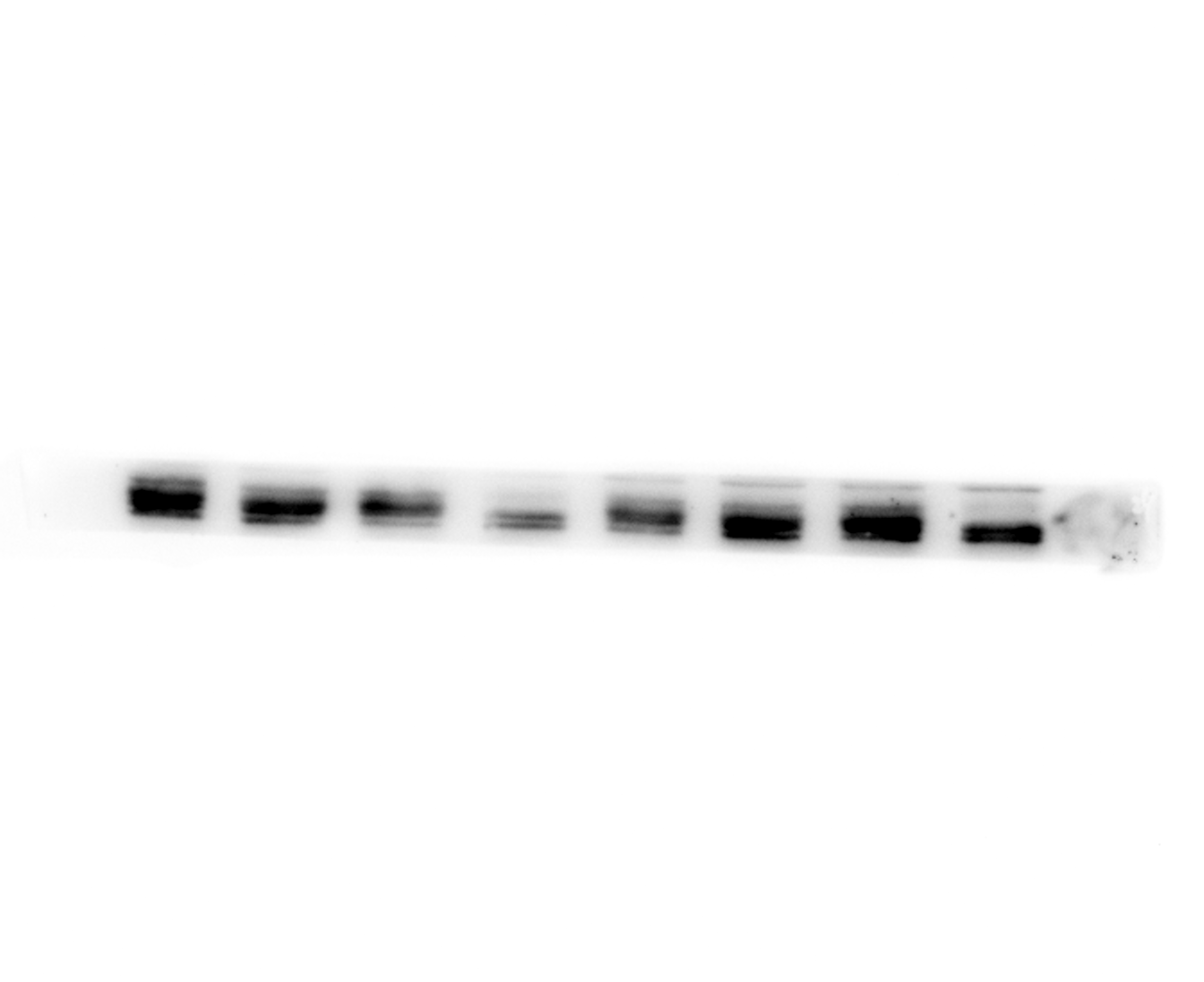

Supplement: Supplementary file 2 [file SupplementaryFile2.zip › WB数据/620/620-wb/snail/620-snail.tif]

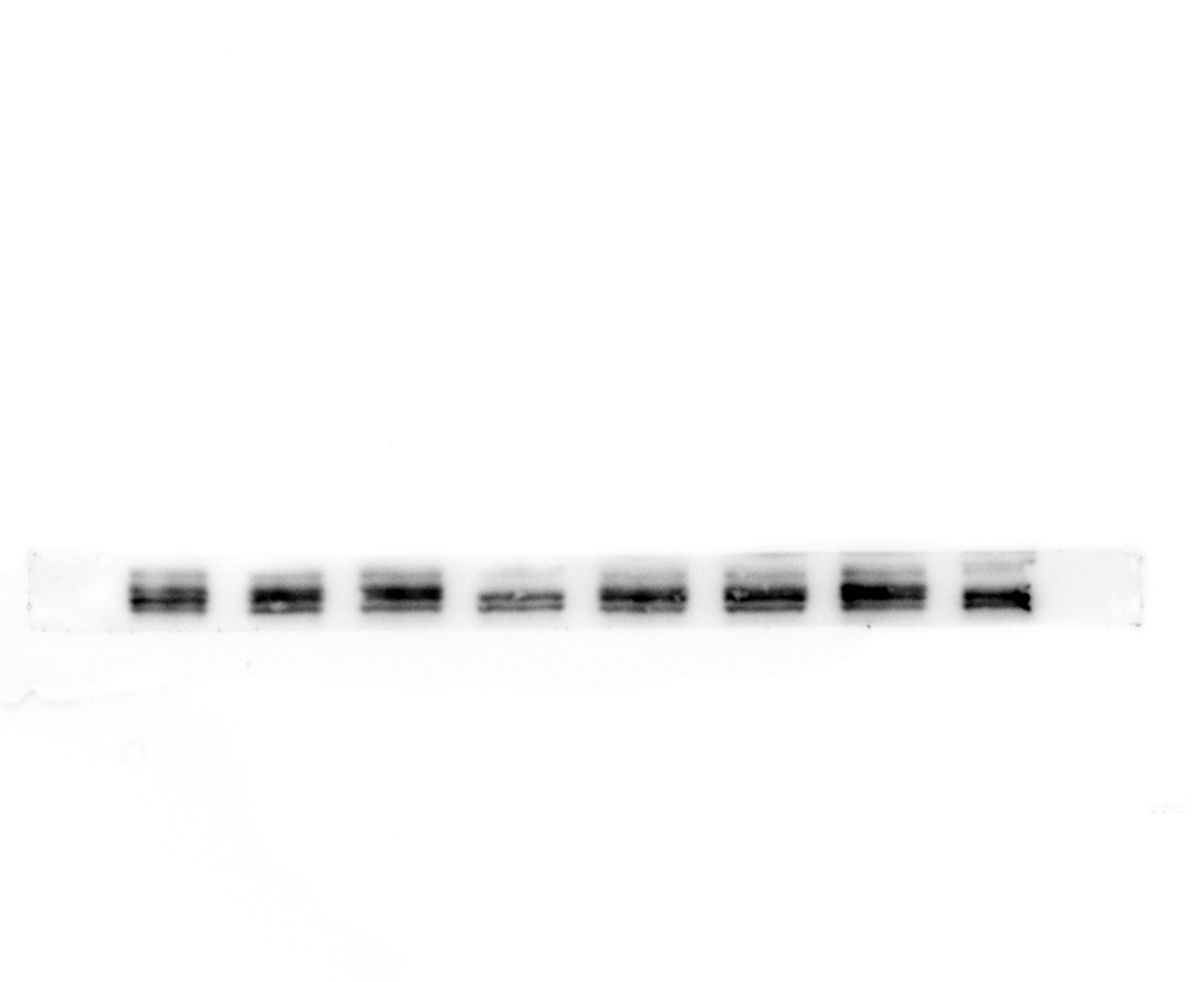

Supplement: Supplementary file 2 [file SupplementaryFile2.zip › WB数据/620/620-wb/snail/620-snail`.tif]

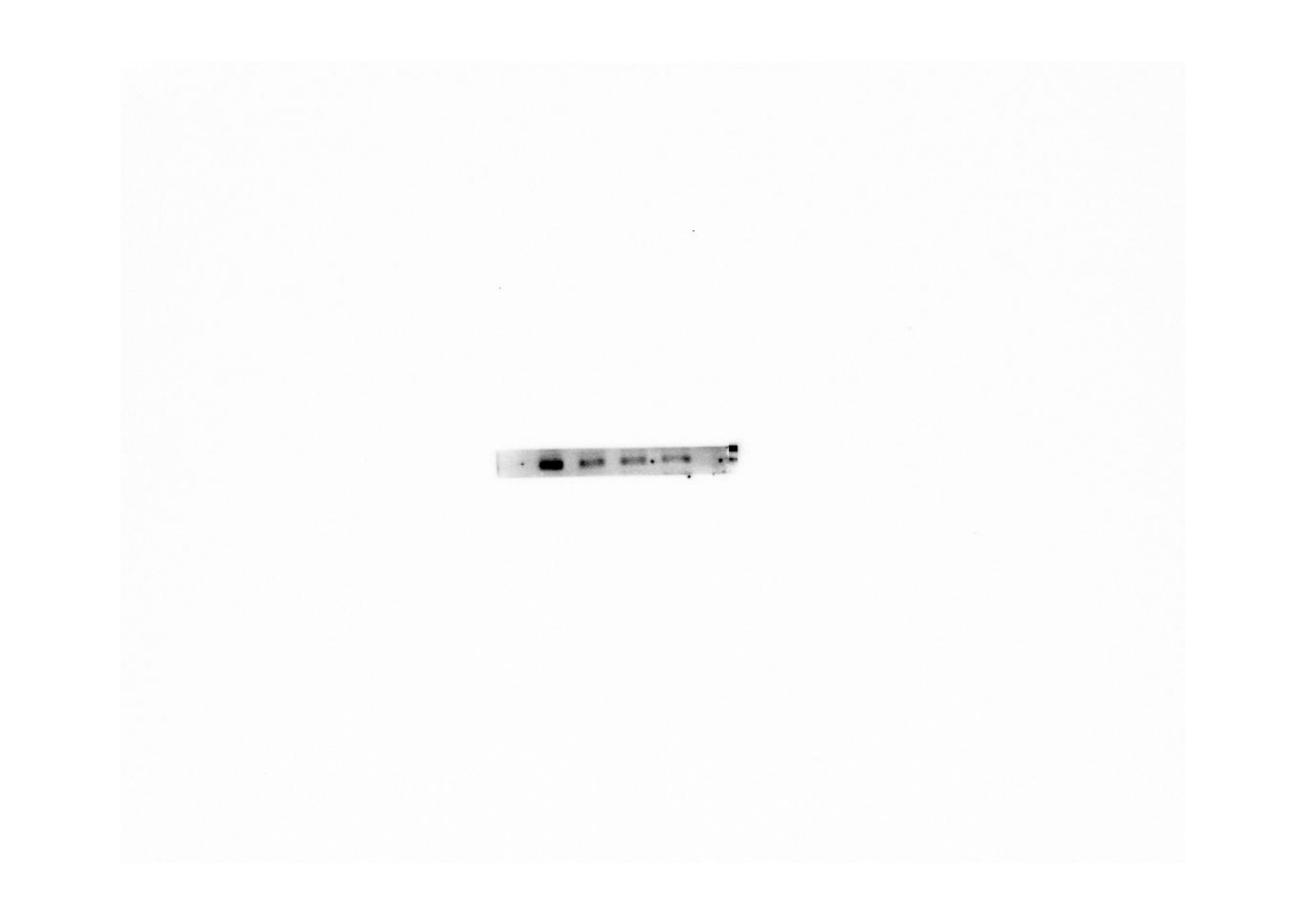

Supplement: Supplementary file 2 [file SupplementaryFile2.zip › WB数据/620/620-wb/snail/s-snail_00.tif]

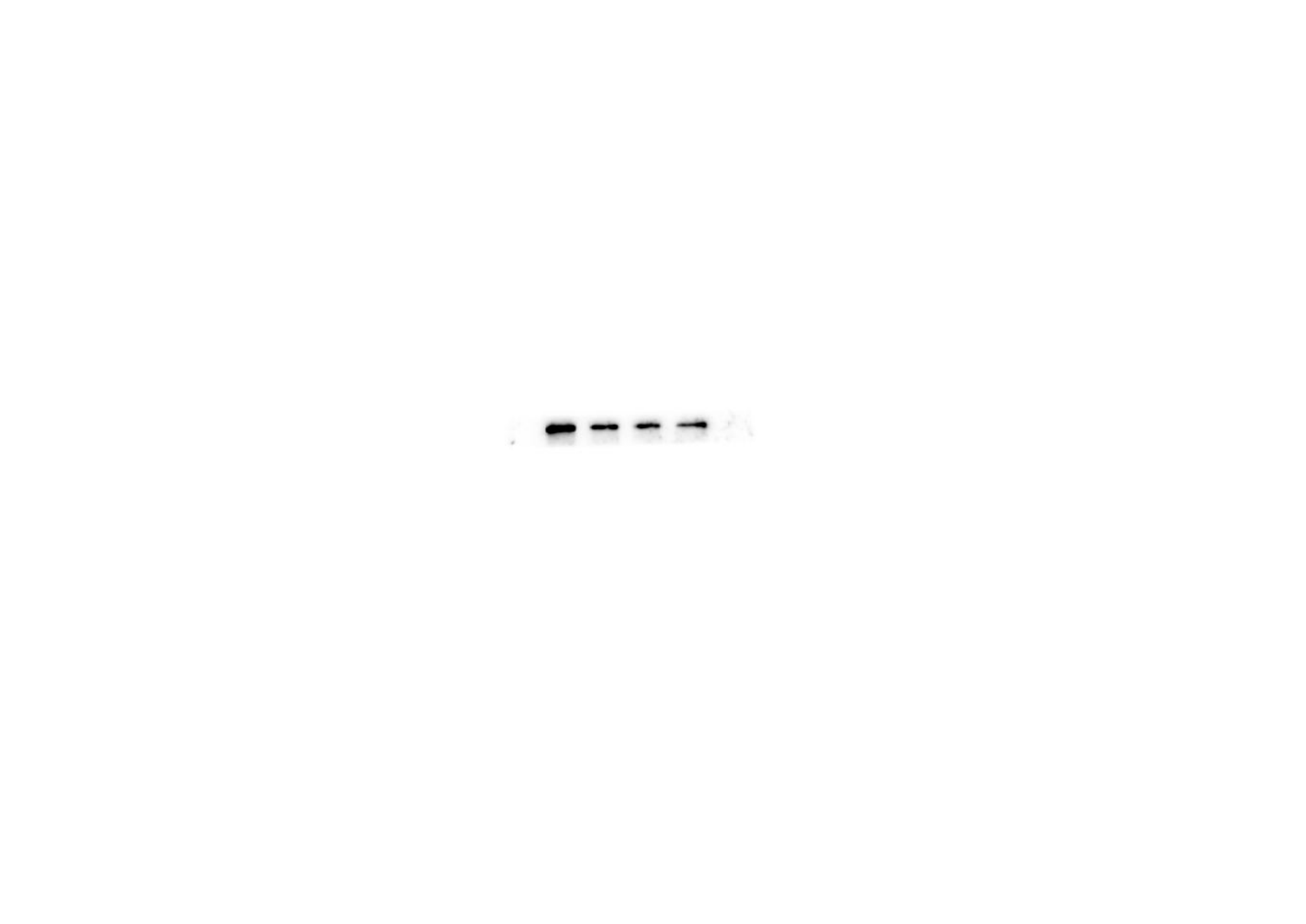

Supplement: Supplementary file 2 [file SupplementaryFile2.zip › WB数据/620/620-wb/snail/snail_00.tif]

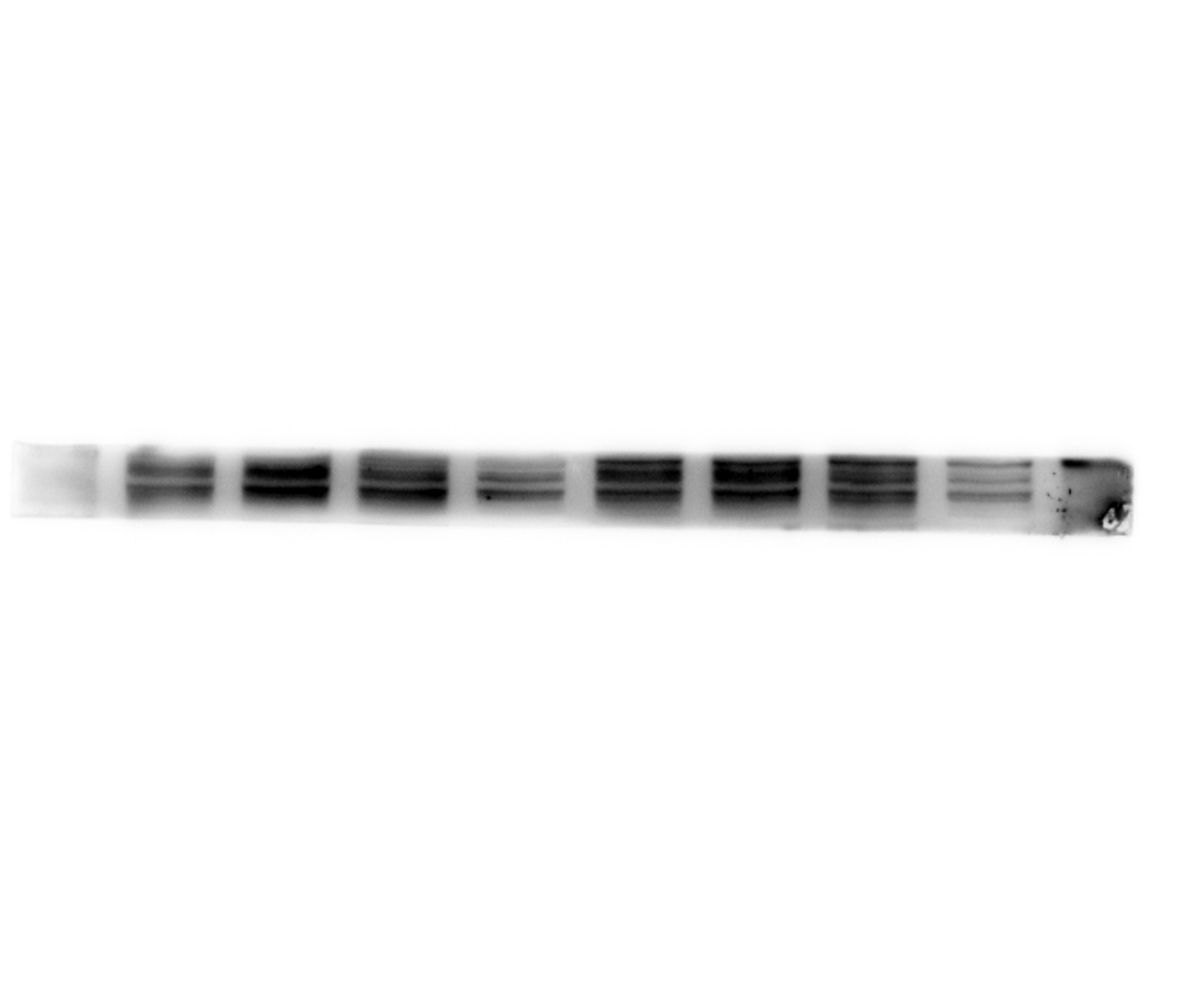

Supplement: Supplementary file 2 [file SupplementaryFile2.zip › WB数据/620/620-wb/srebp1/620-srebp1-68.tif]

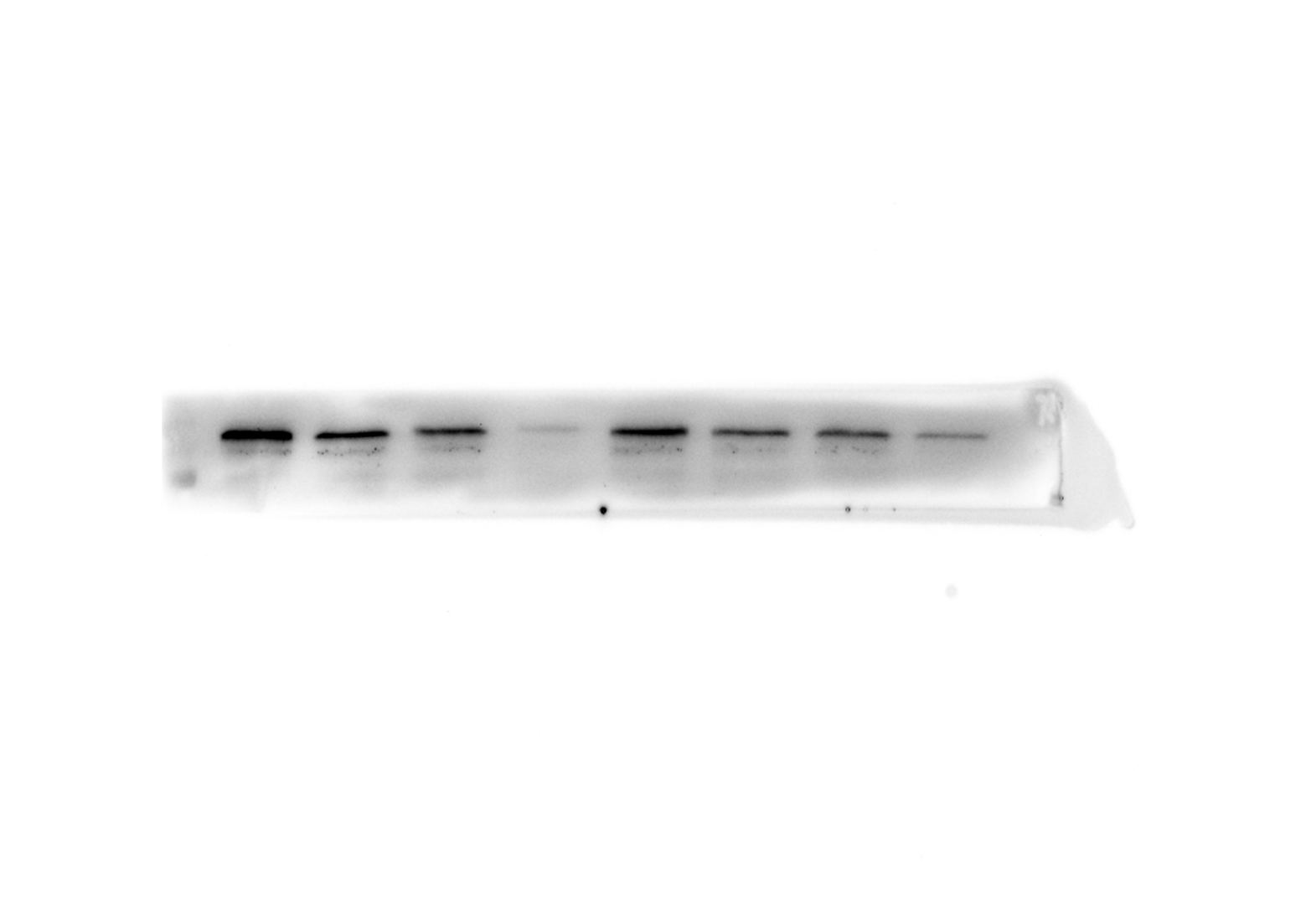

Supplement: Supplementary file 2 [file SupplementaryFile2.zip › WB数据/620/620-wb/srebp1/620-srebp1_00.tif]

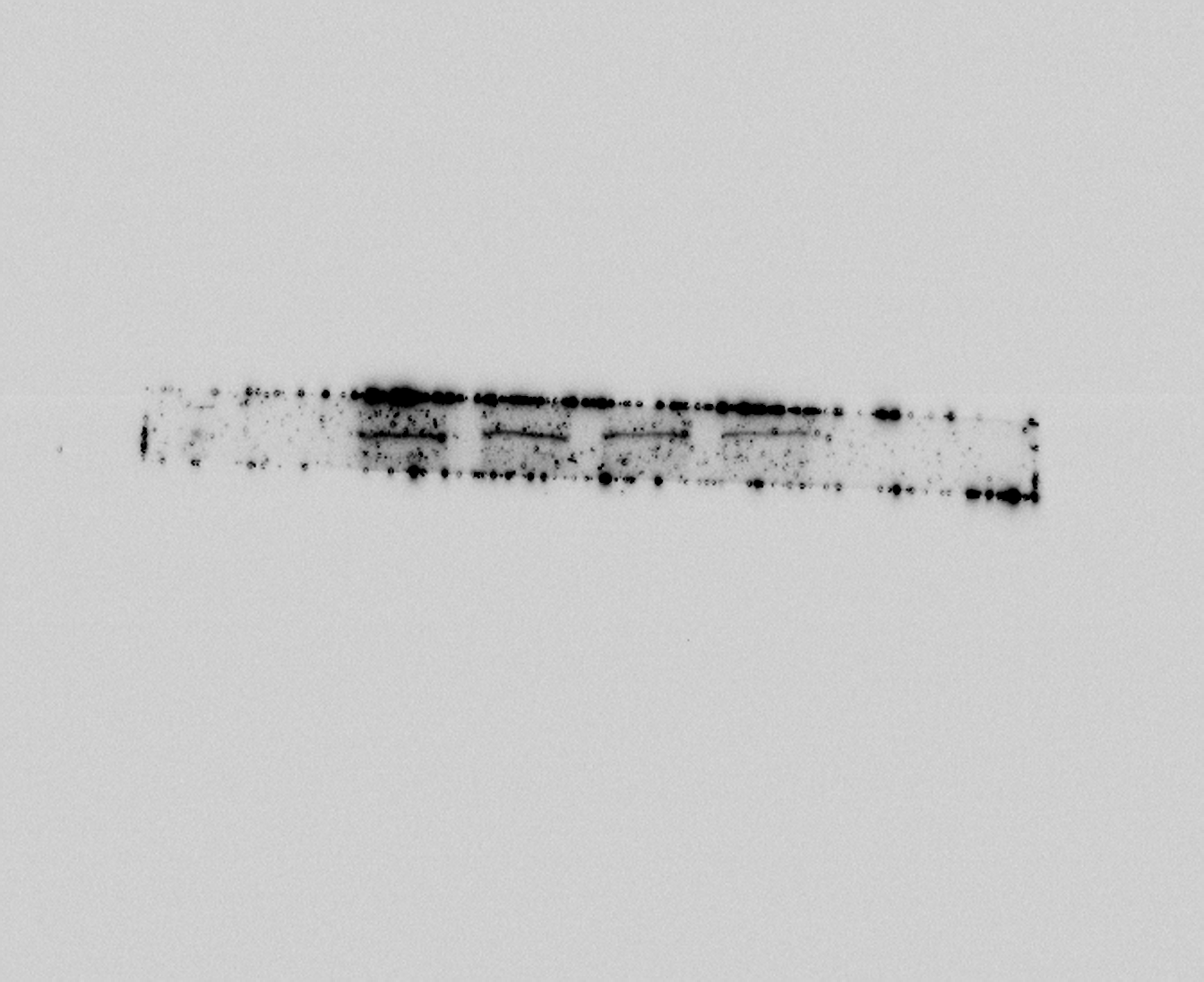

Supplement: Supplementary file 2 [file SupplementaryFile2.zip › WB数据/620/620-wb/srebp1/srebp1-68-.tif]

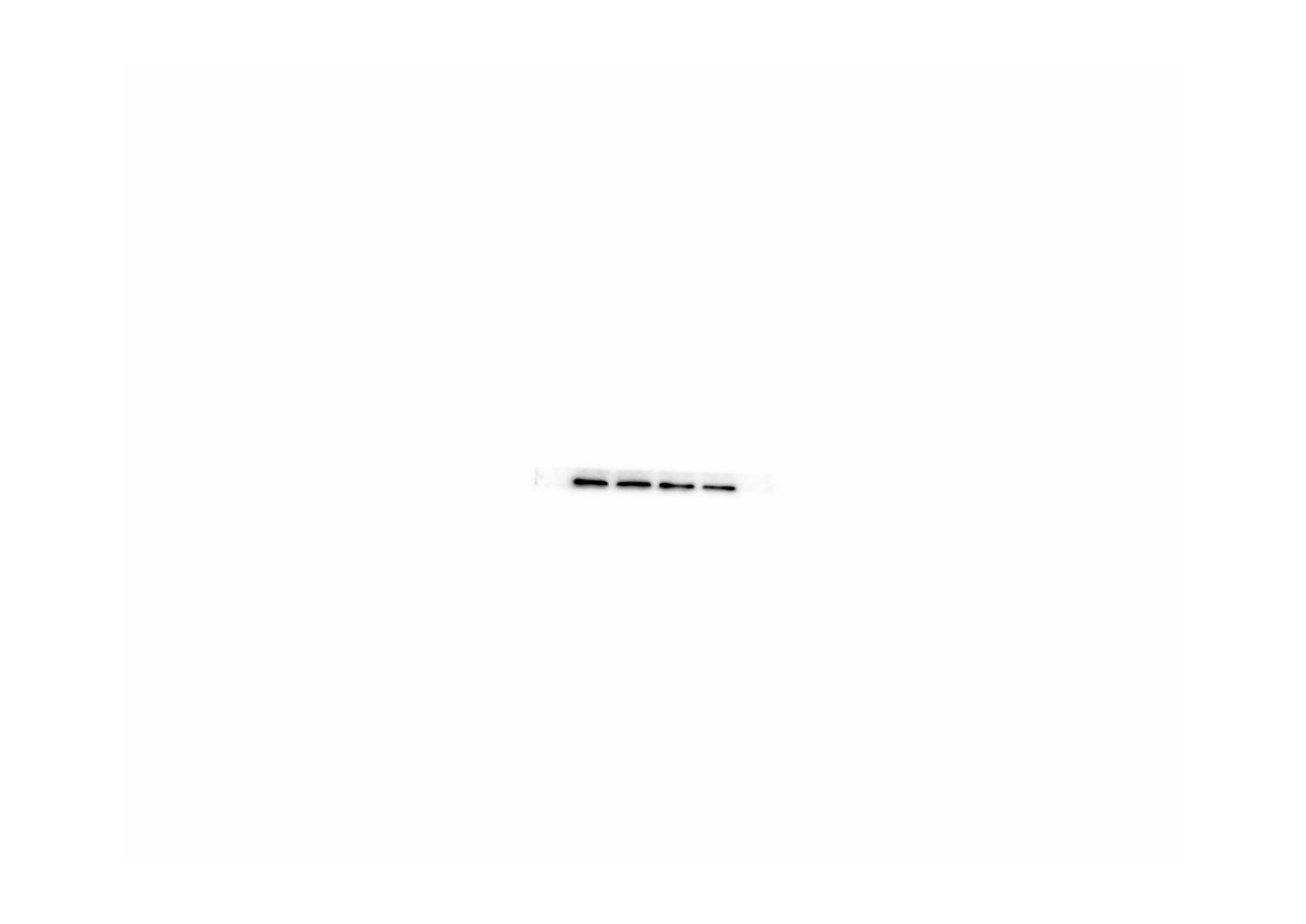

Supplement: Supplementary file 2 [file SupplementaryFile2.zip › WB数据/620/620-wb/srebp1/srebp1-68_00.tif]

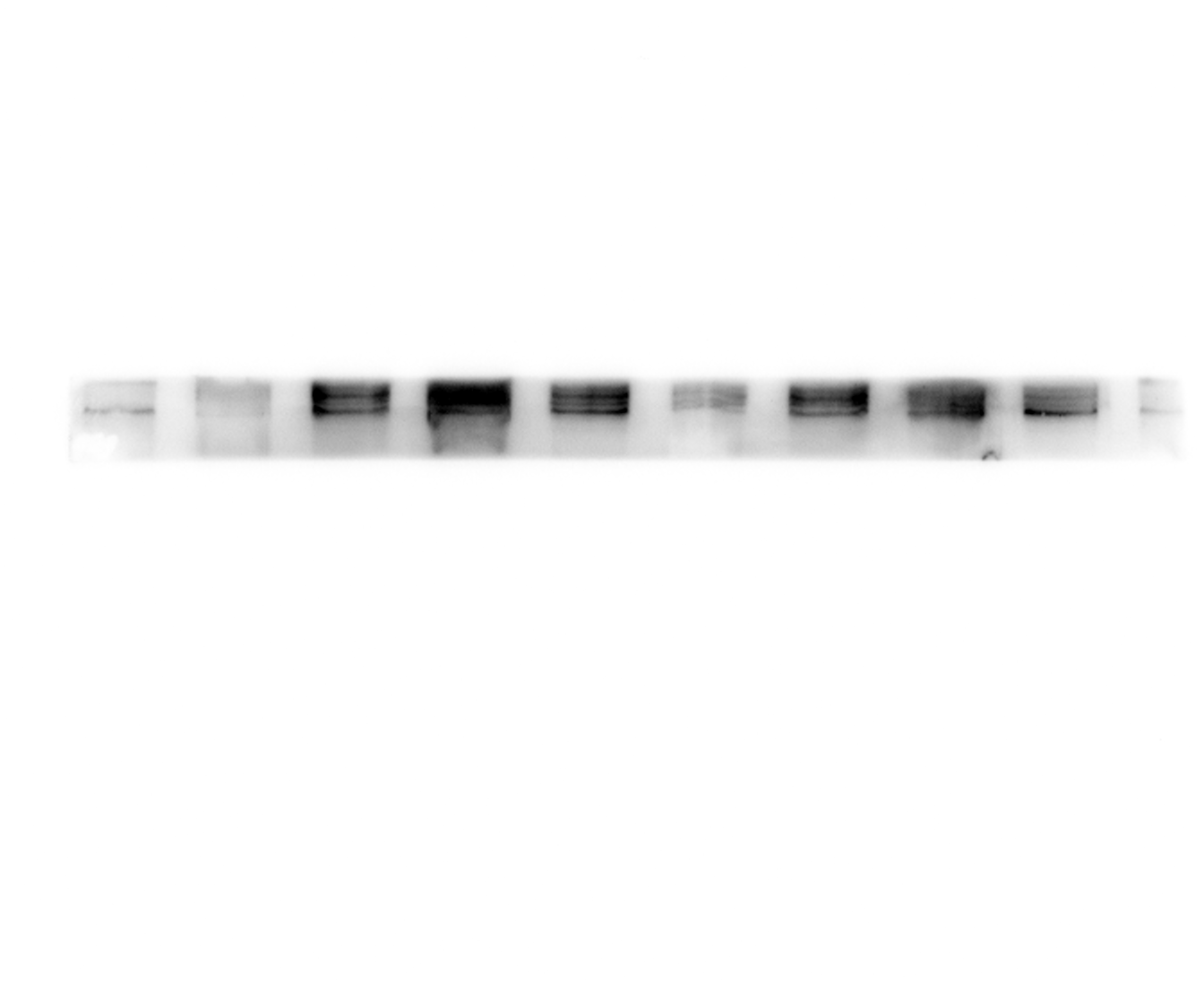

Supplement: Supplementary file 2 [file SupplementaryFile2.zip › WB数据/620/620-wb/zo1/620-zo1.tif]
