# Supplementary figures and images for: Brexpiprazole inhibits EMT and migration of colorectal cancer cells by downregulating the SREBP1/SNAI1 signaling pathway (part 2 of 4)
Source: Front Oncol. 2026 Jan 15;15:1734678. doi: 10.3389/fonc.2025.1734678 (PMC12852020; doi:10.3389/fonc.2025.1734678)

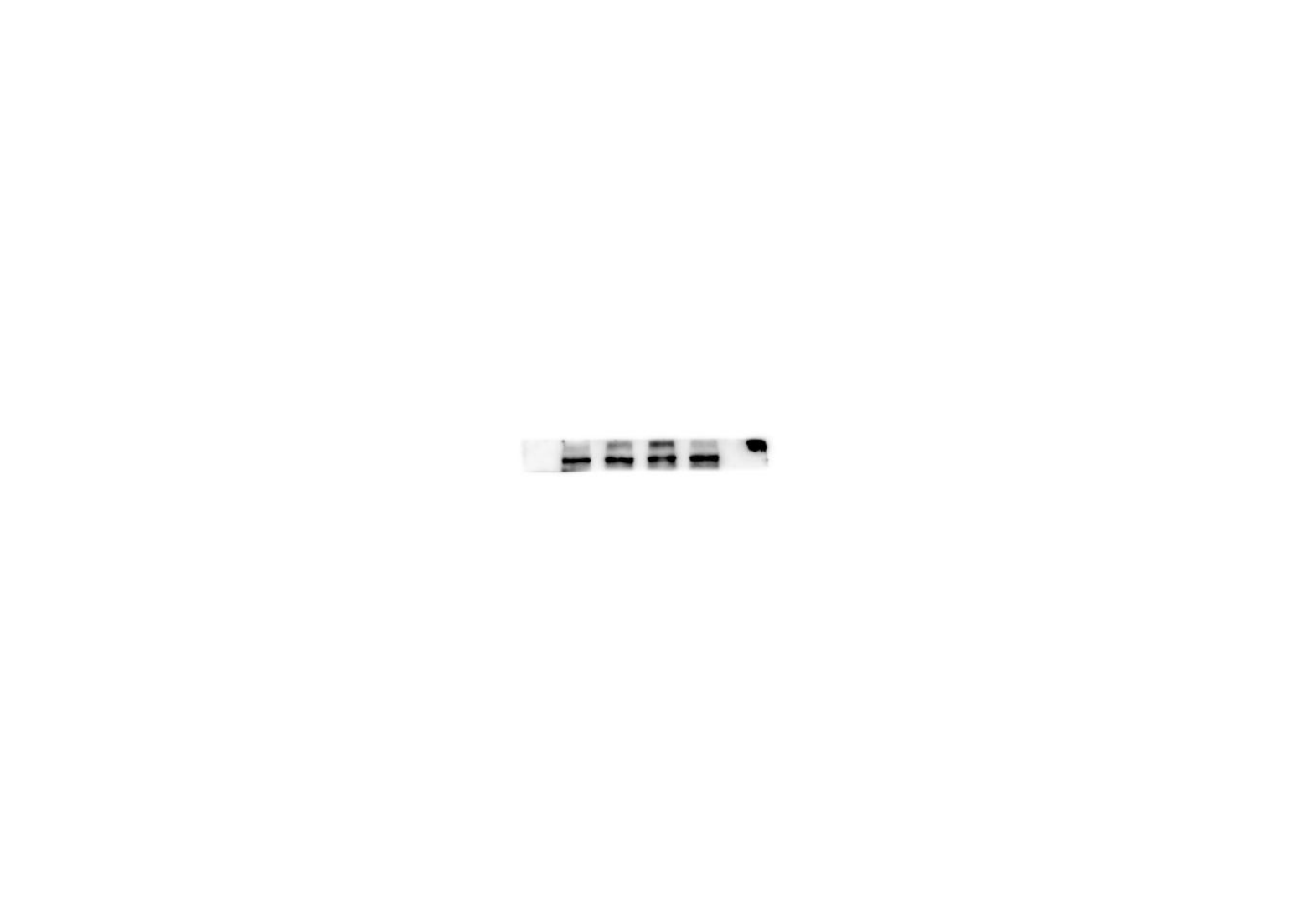

Supplement: Supplementary file 2 [file SupplementaryFile2.zip › WB数据/620/620-wb/zo1/zo-1-_00.tif]

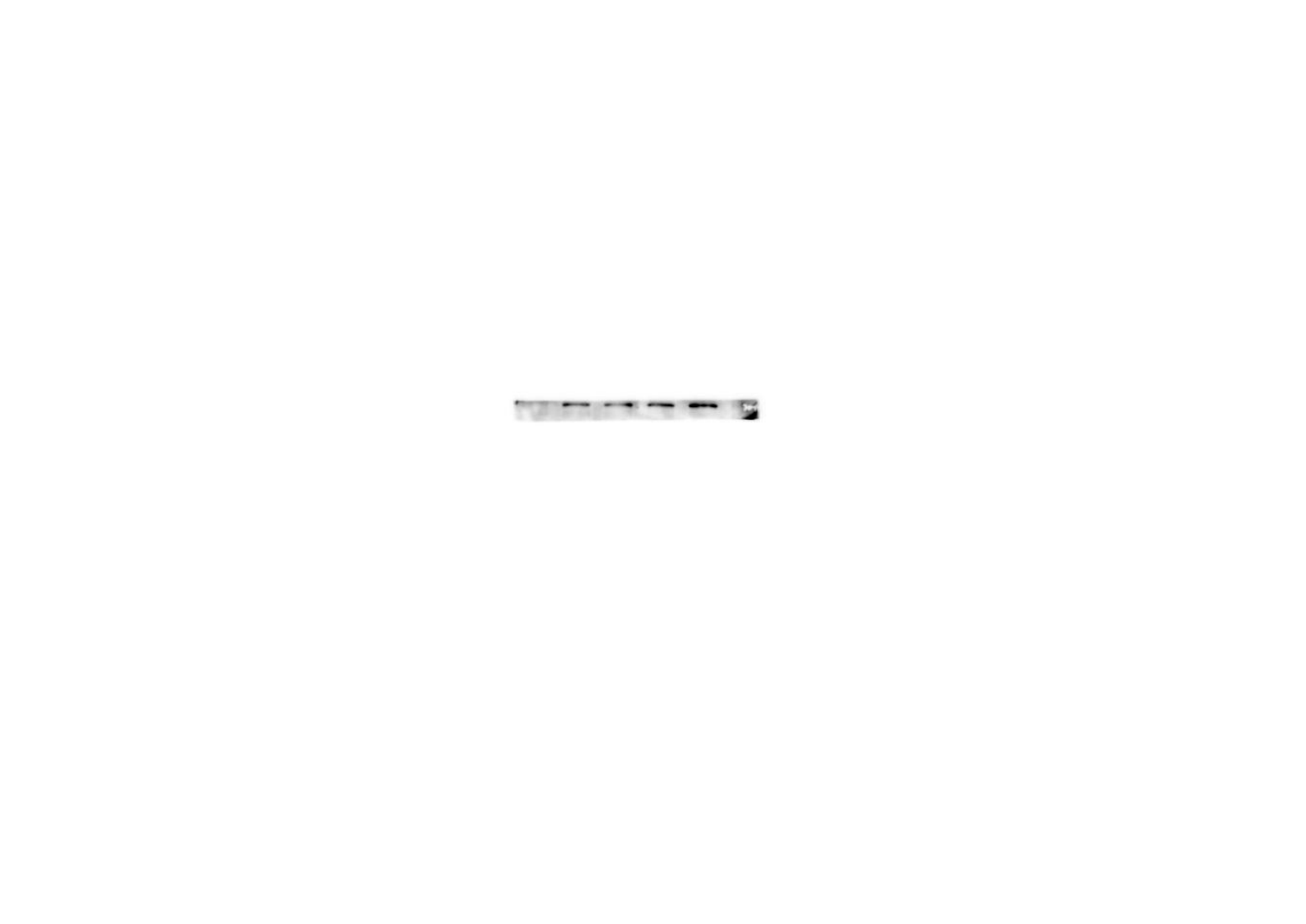

Supplement: Supplementary file 2 [file SupplementaryFile2.zip › WB数据/620/620-wb/zo1/zo1-_00.tif]

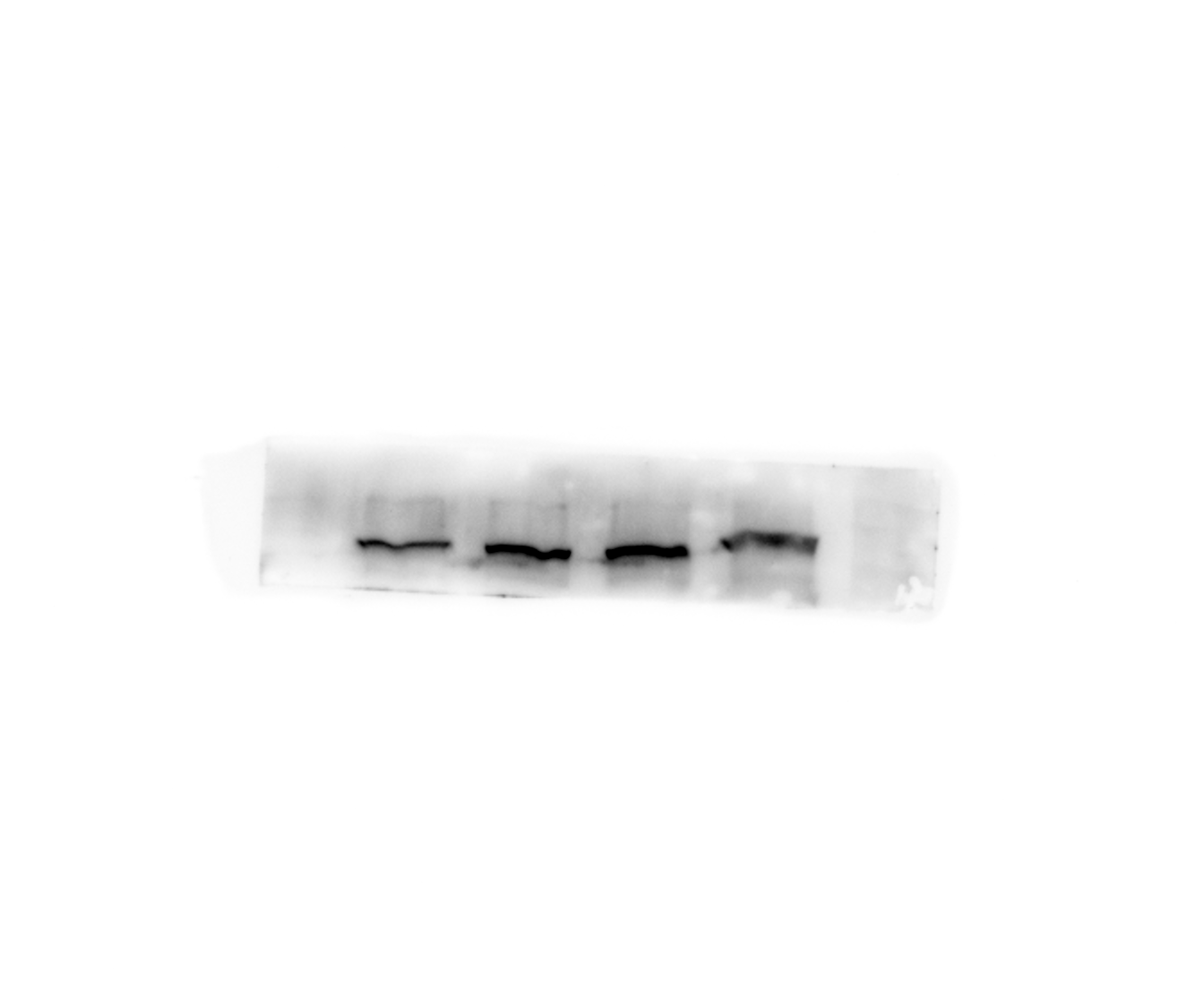

Supplement: Supplementary file 2 [file SupplementaryFile2.zip › WB数据/620/620-wb/zo1/zo1·.tif]

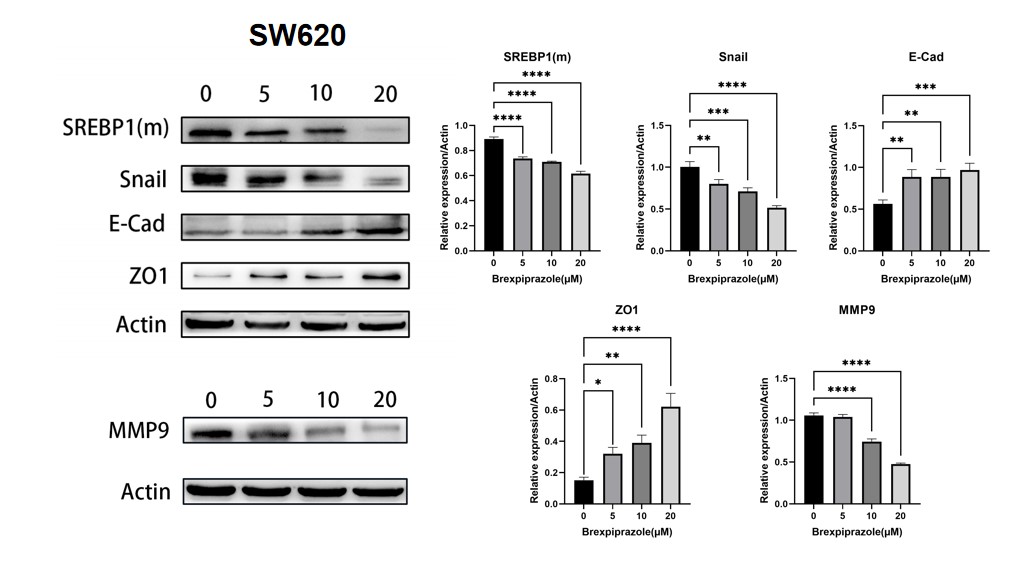

Supplement: Supplementary file 2 [file SupplementaryFile2.zip › WB数据/620/数据图/1.jpg]

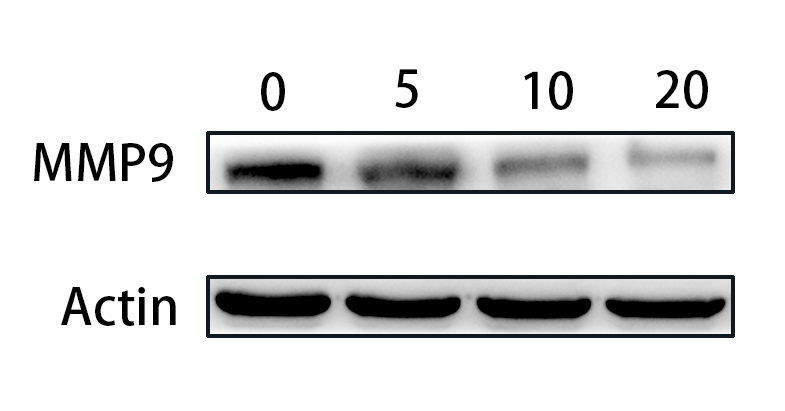

Supplement: Supplementary file 2 [file SupplementaryFile2.zip › WB数据/620/数据图/620-MMP9.png]

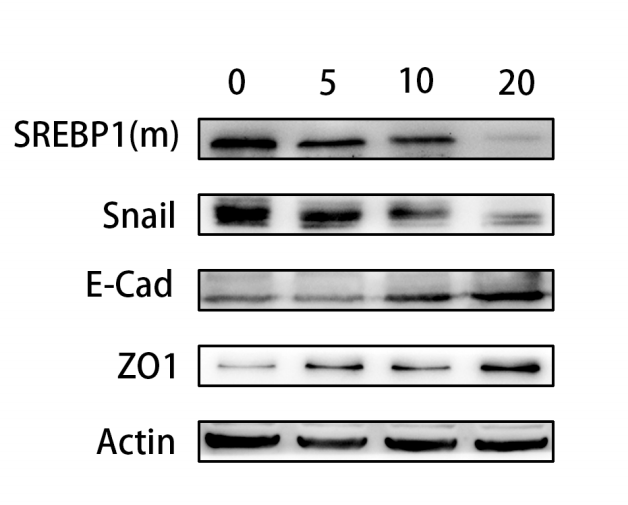

Supplement: Supplementary file 2 [file SupplementaryFile2.zip › WB数据/620/数据图/620.png]

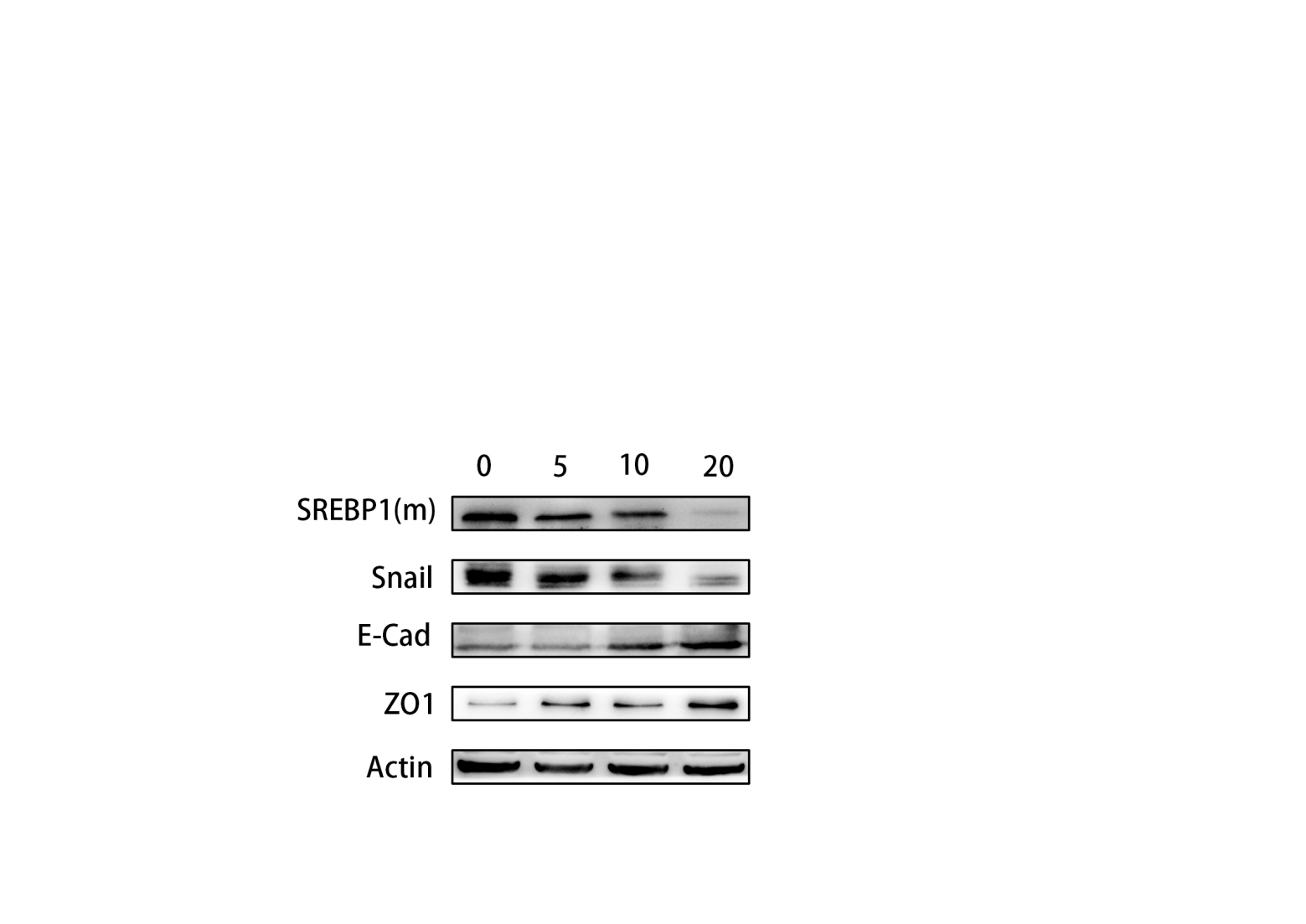

Supplement: Supplementary file 2 [file SupplementaryFile2.zip › WB数据/620/数据图/620.tif]

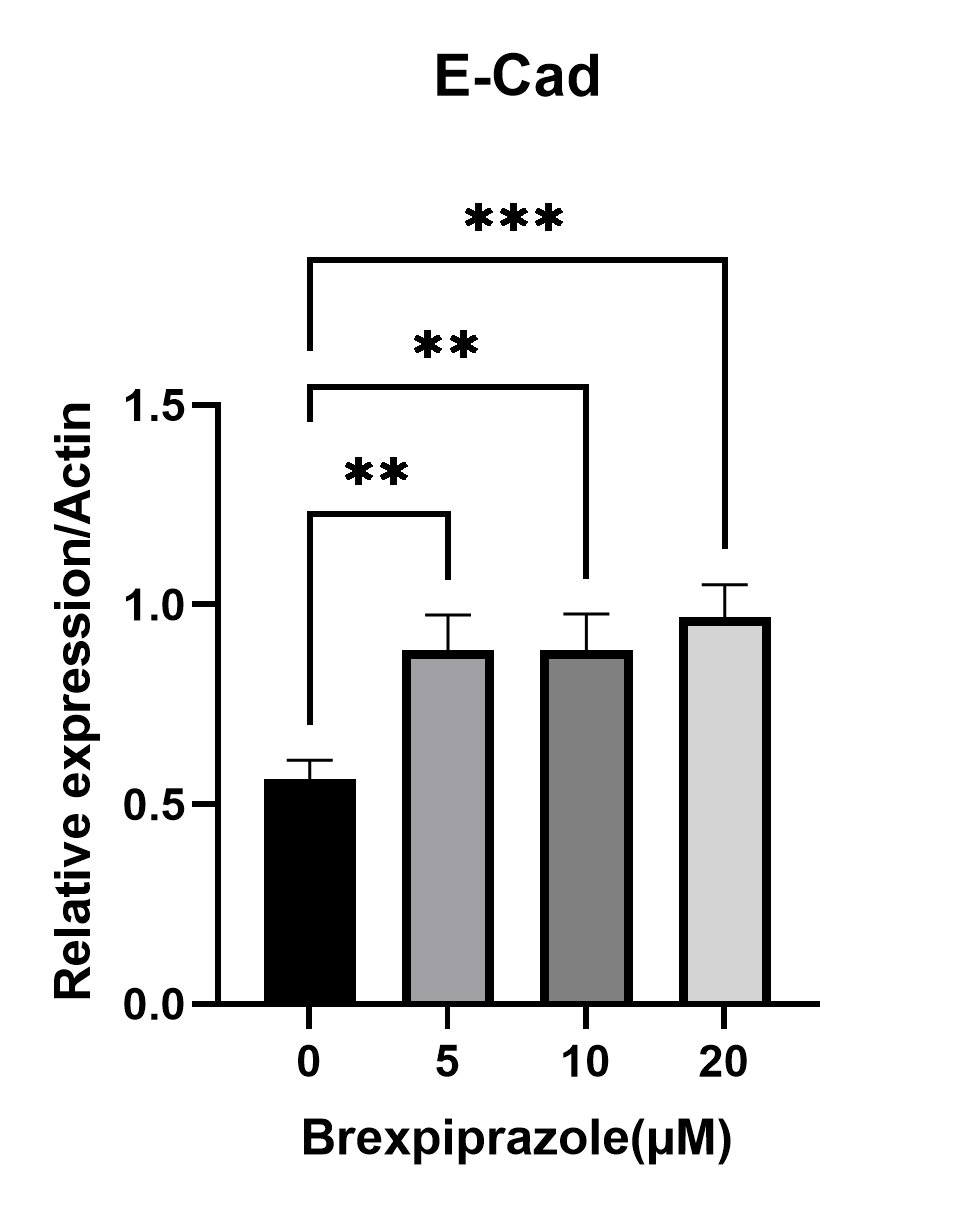

Supplement: Supplementary file 2 [file SupplementaryFile2.zip › WB数据/620/数据图/E.tif]

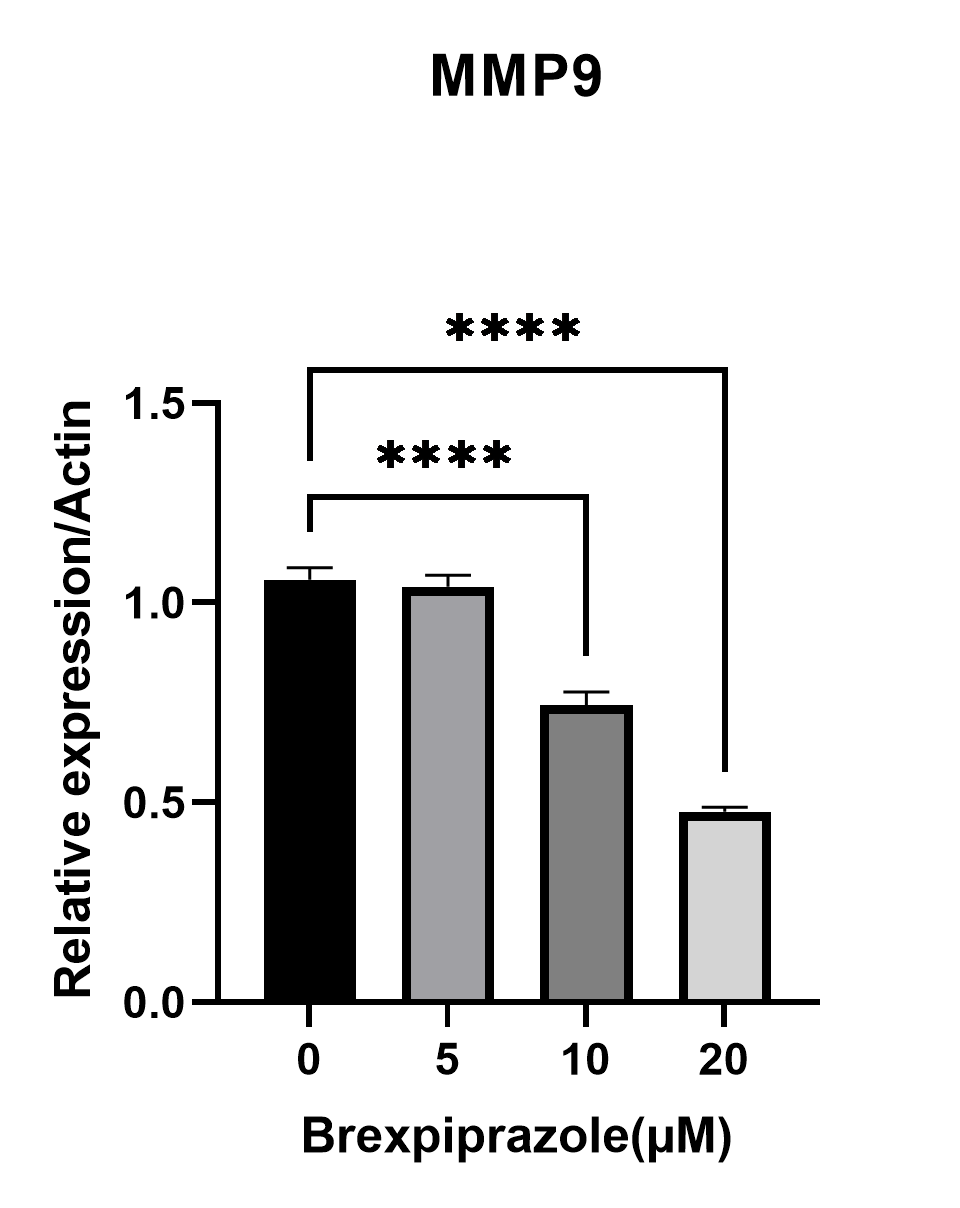

Supplement: Supplementary file 2 [file SupplementaryFile2.zip › WB数据/620/数据图/MMP9.tif]

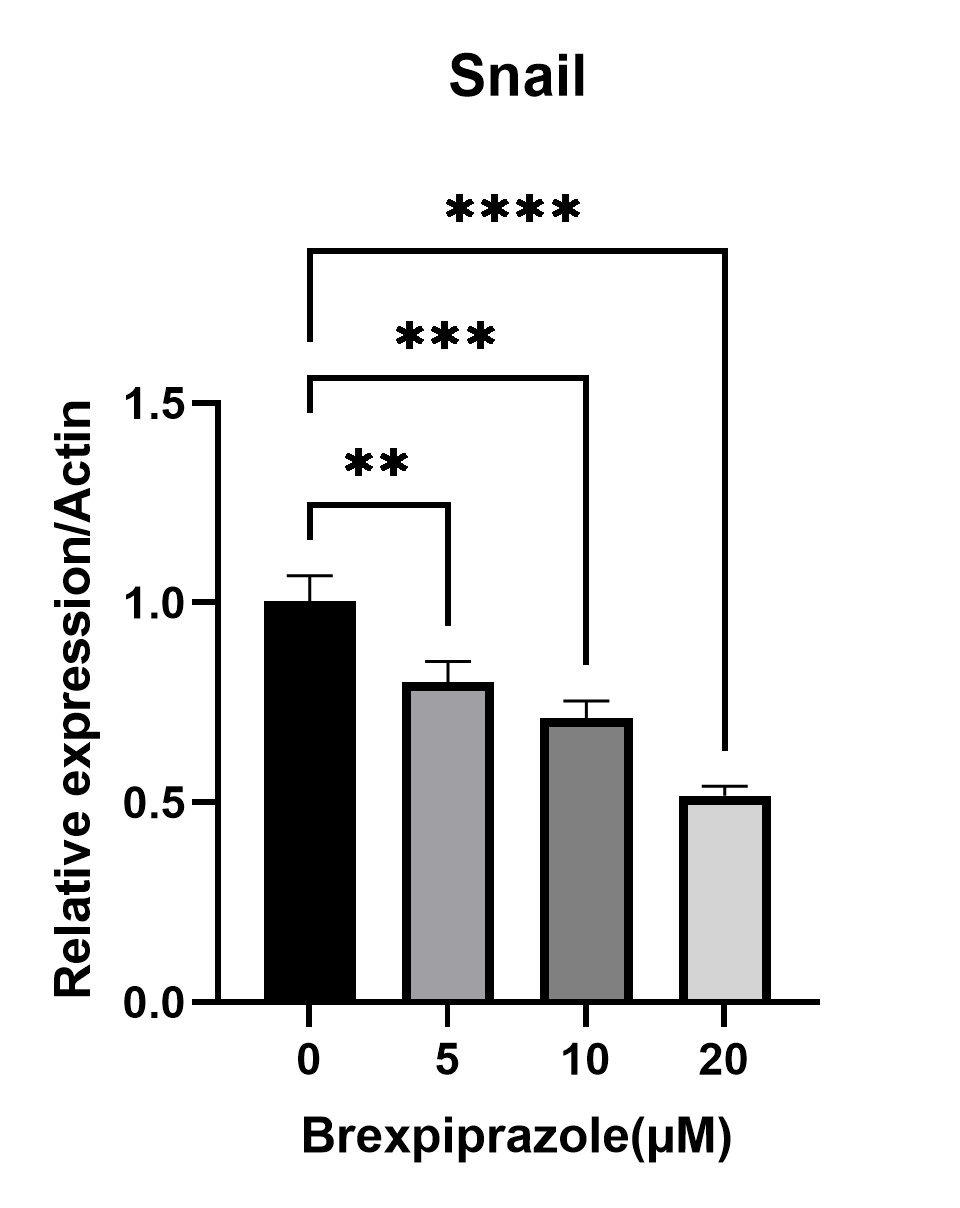

Supplement: Supplementary file 2 [file SupplementaryFile2.zip › WB数据/620/数据图/SNAIL.tif]

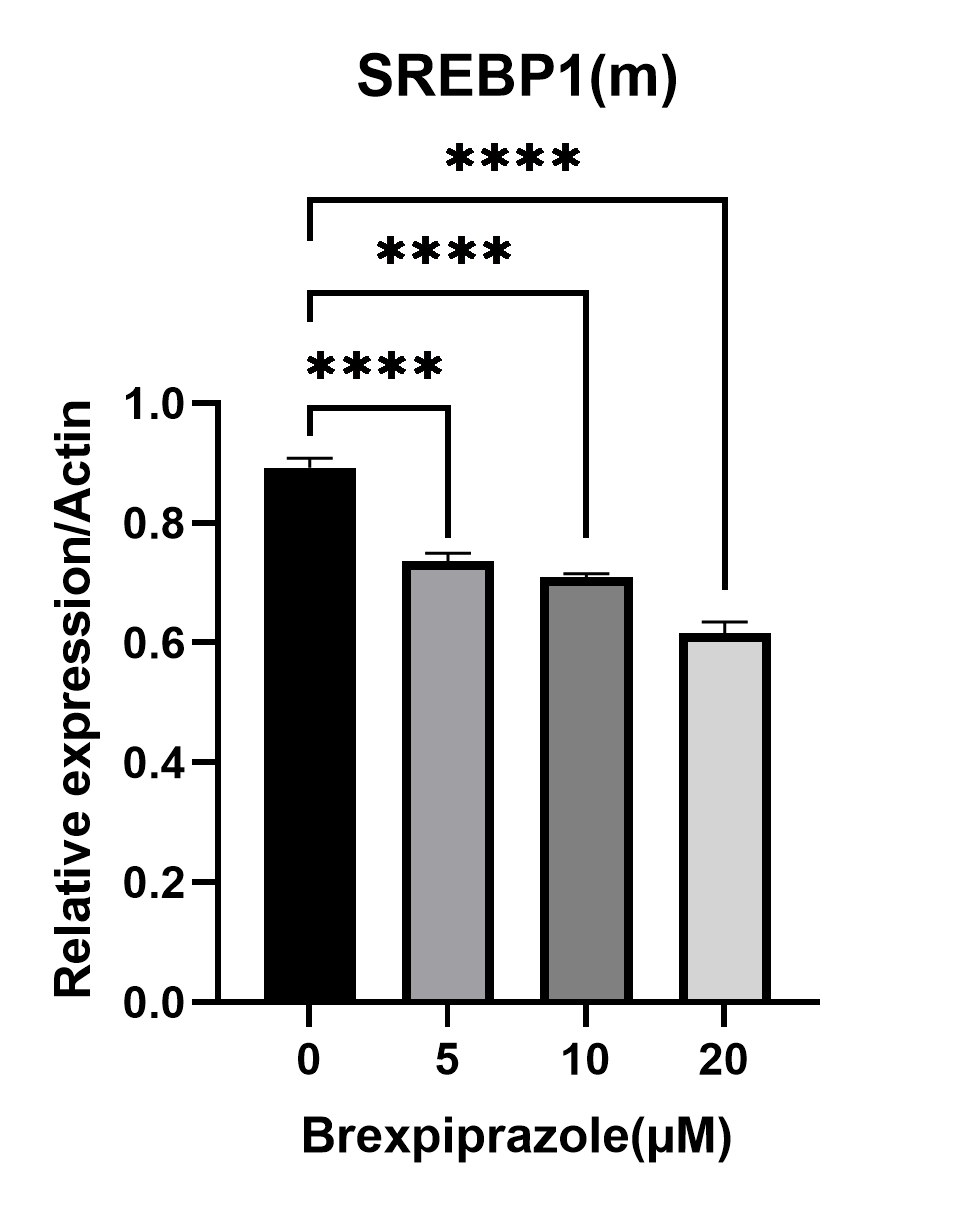

Supplement: Supplementary file 2 [file SupplementaryFile2.zip › WB数据/620/数据图/SREBP1.tif]

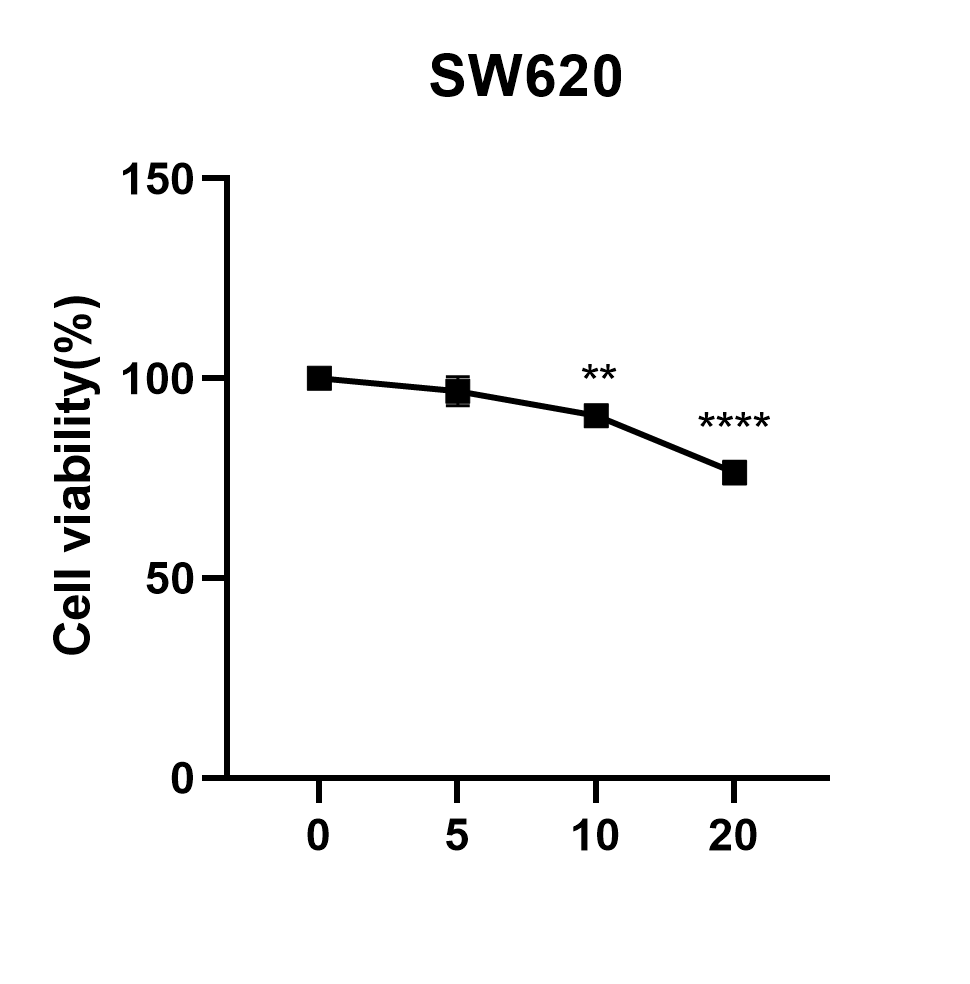

Supplement: Supplementary file 2 [file SupplementaryFile2.zip › WB数据/620/数据图/SW620-CCK8.tif]

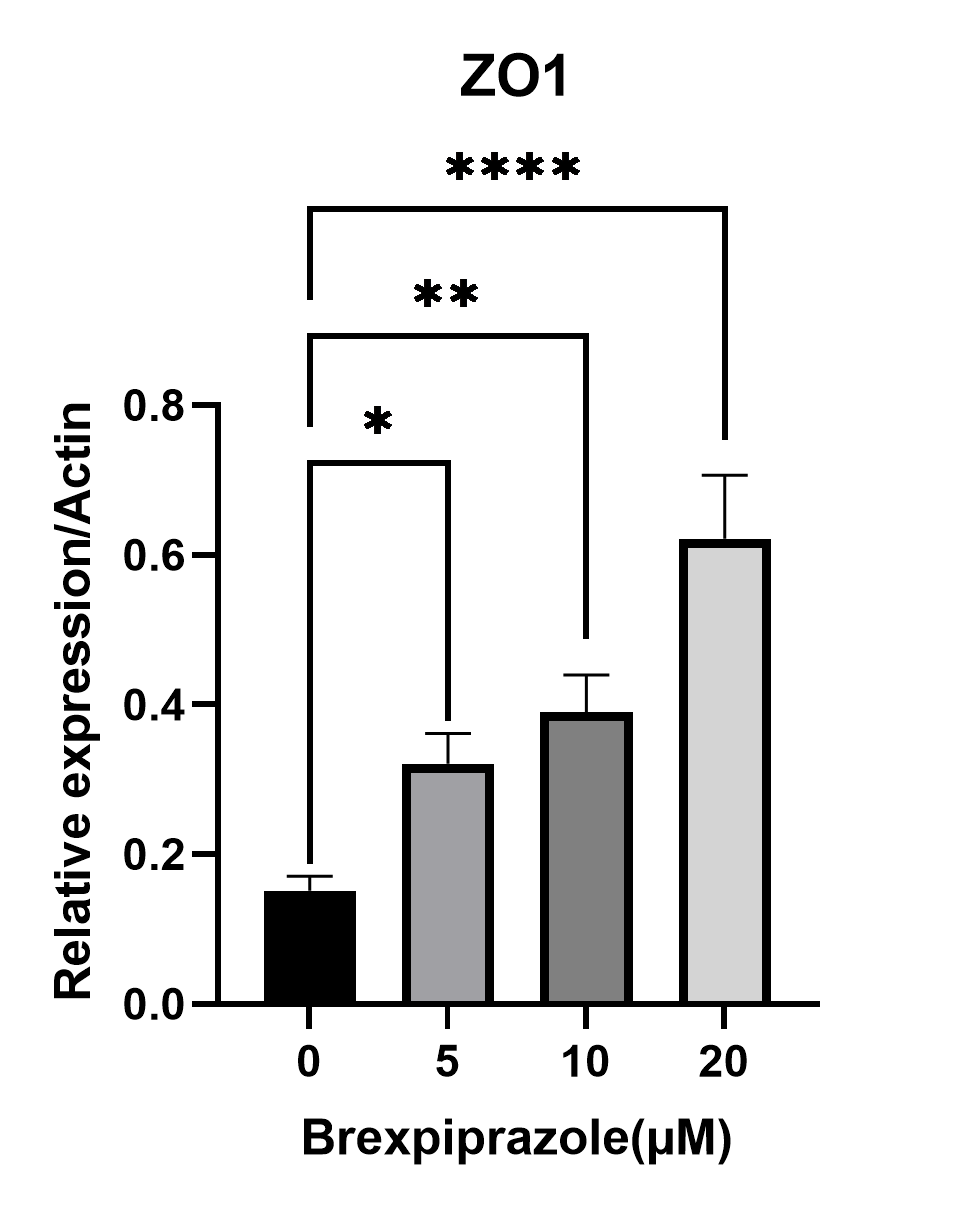

Supplement: Supplementary file 2 [file SupplementaryFile2.zip › WB数据/620/数据图/ZO1.tif]

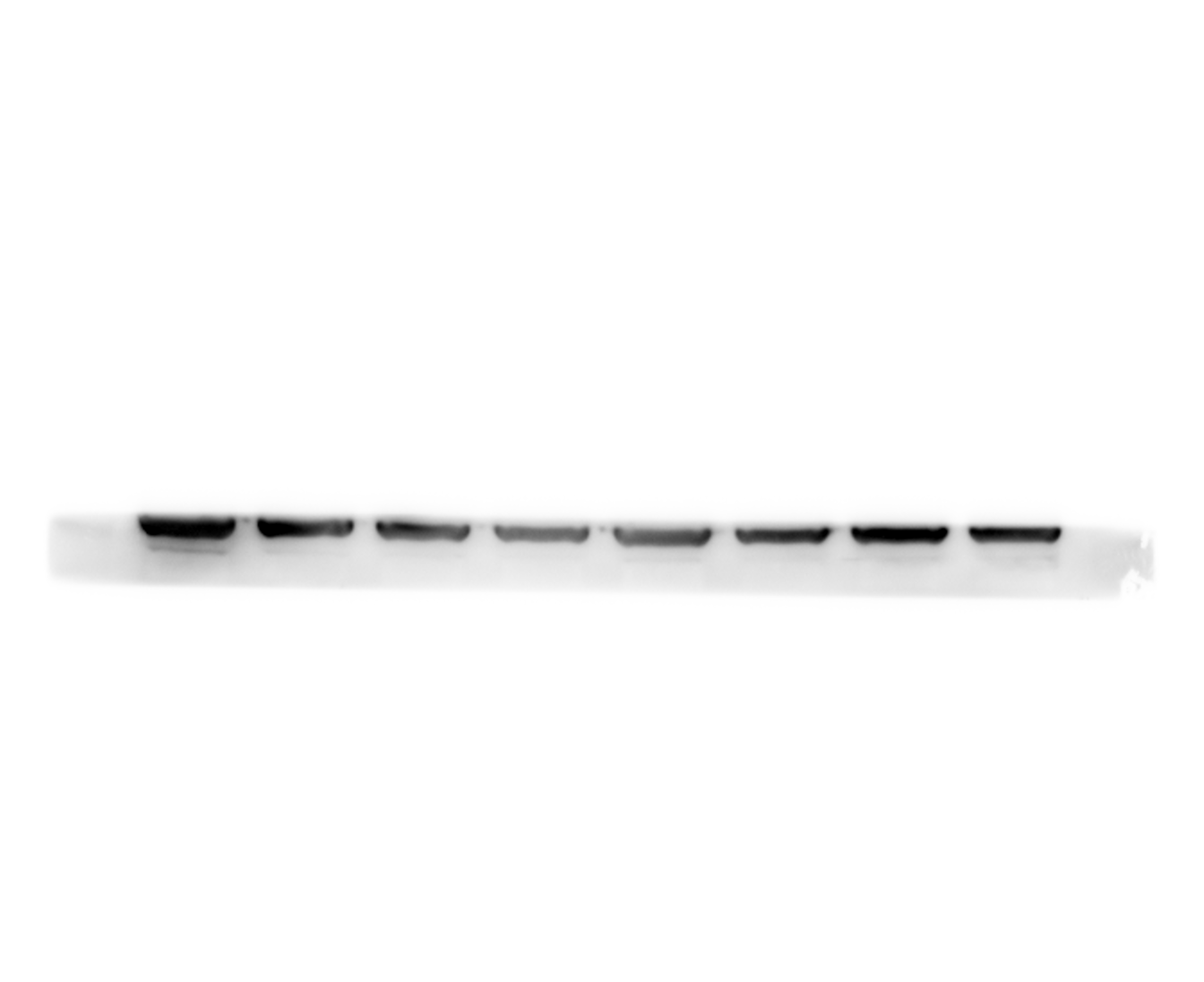

Supplement: Supplementary file 2 [file SupplementaryFile2.zip › WB数据/620-oe/620-wb/actin/620-actin.tif]

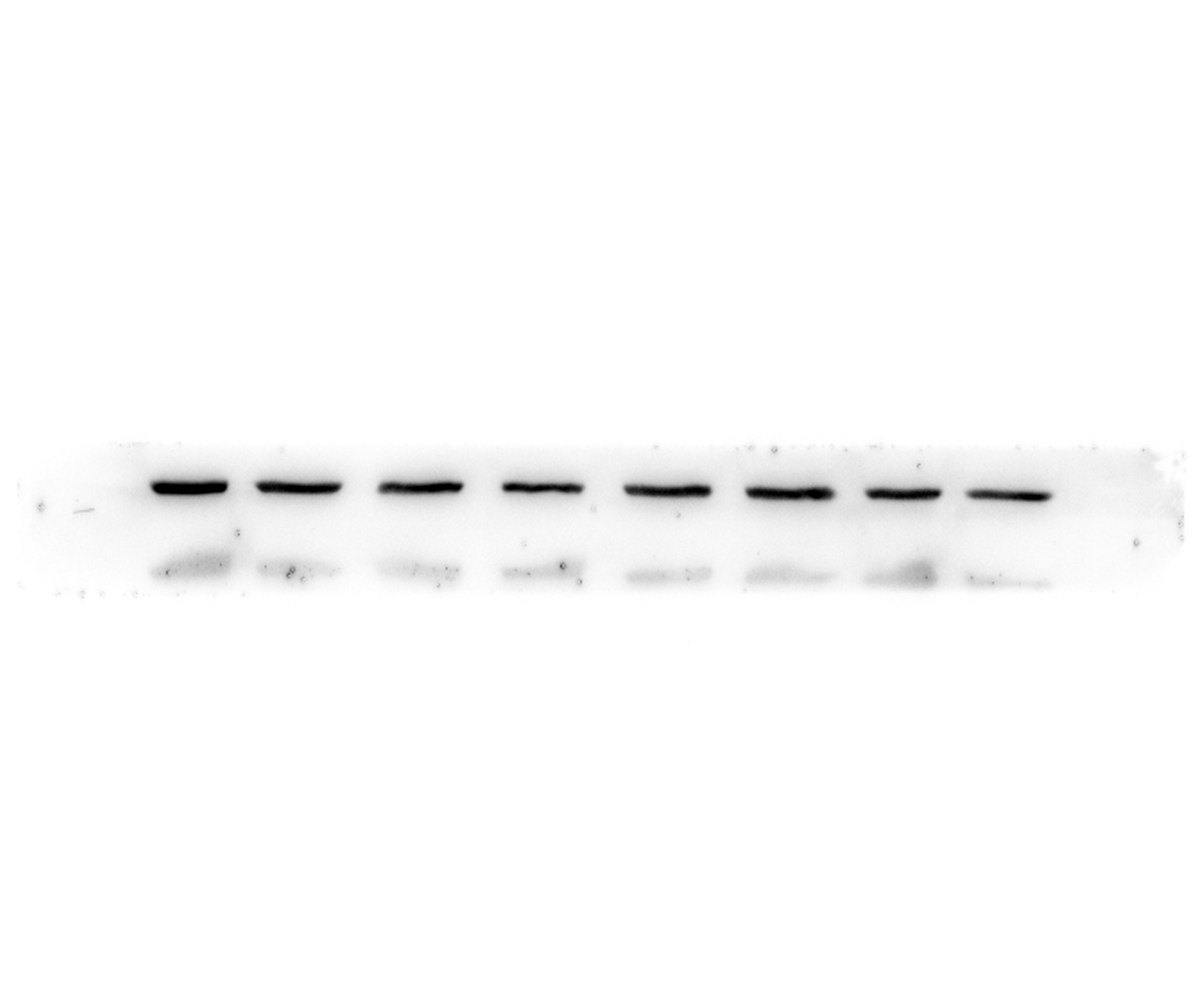

Supplement: Supplementary file 2 [file SupplementaryFile2.zip › WB数据/620-oe/620-wb/actin/620-actin`.tif]

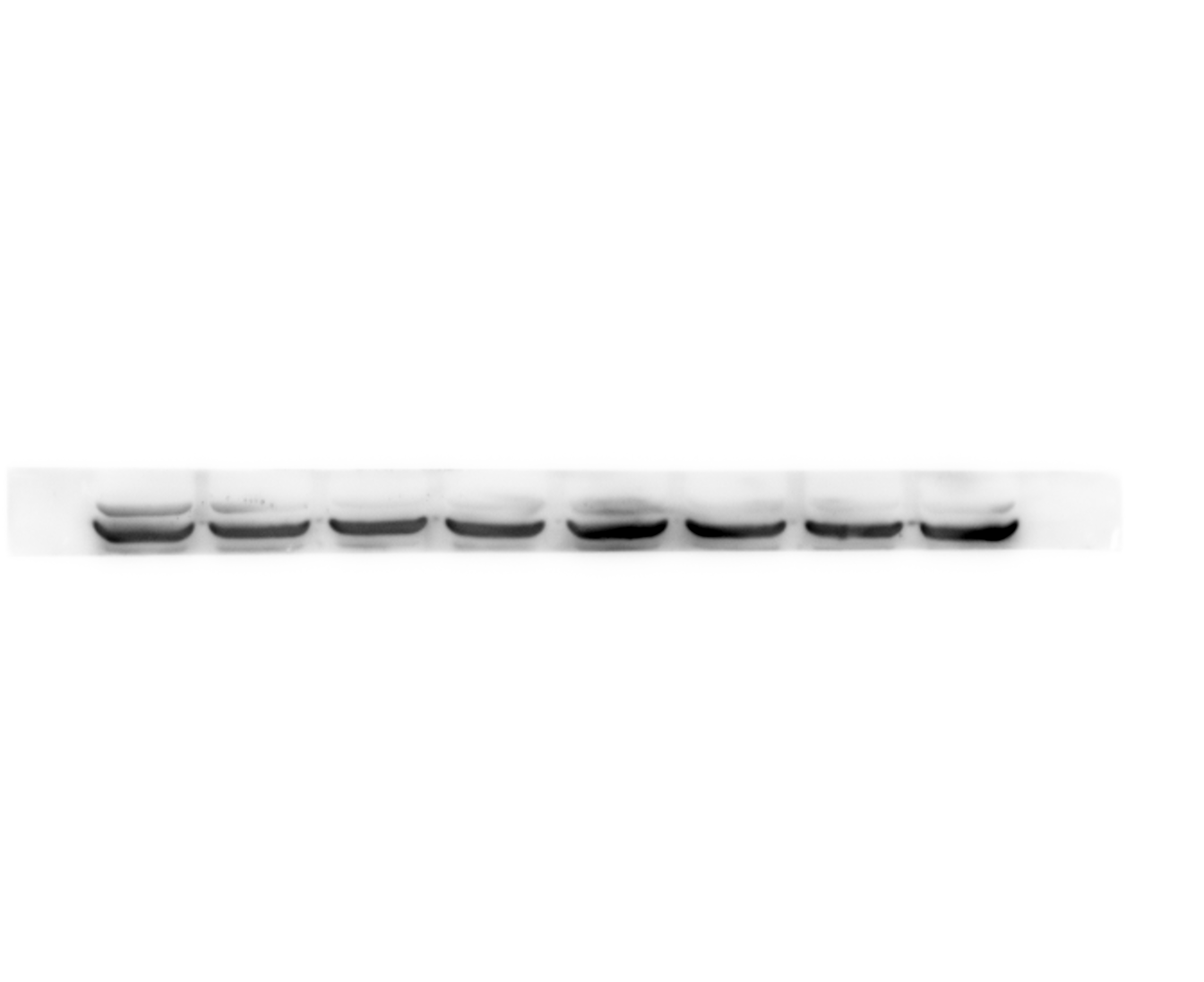

Supplement: Supplementary file 2 [file SupplementaryFile2.zip › WB数据/620-oe/620-wb/actin/ACTIN-1.tif]

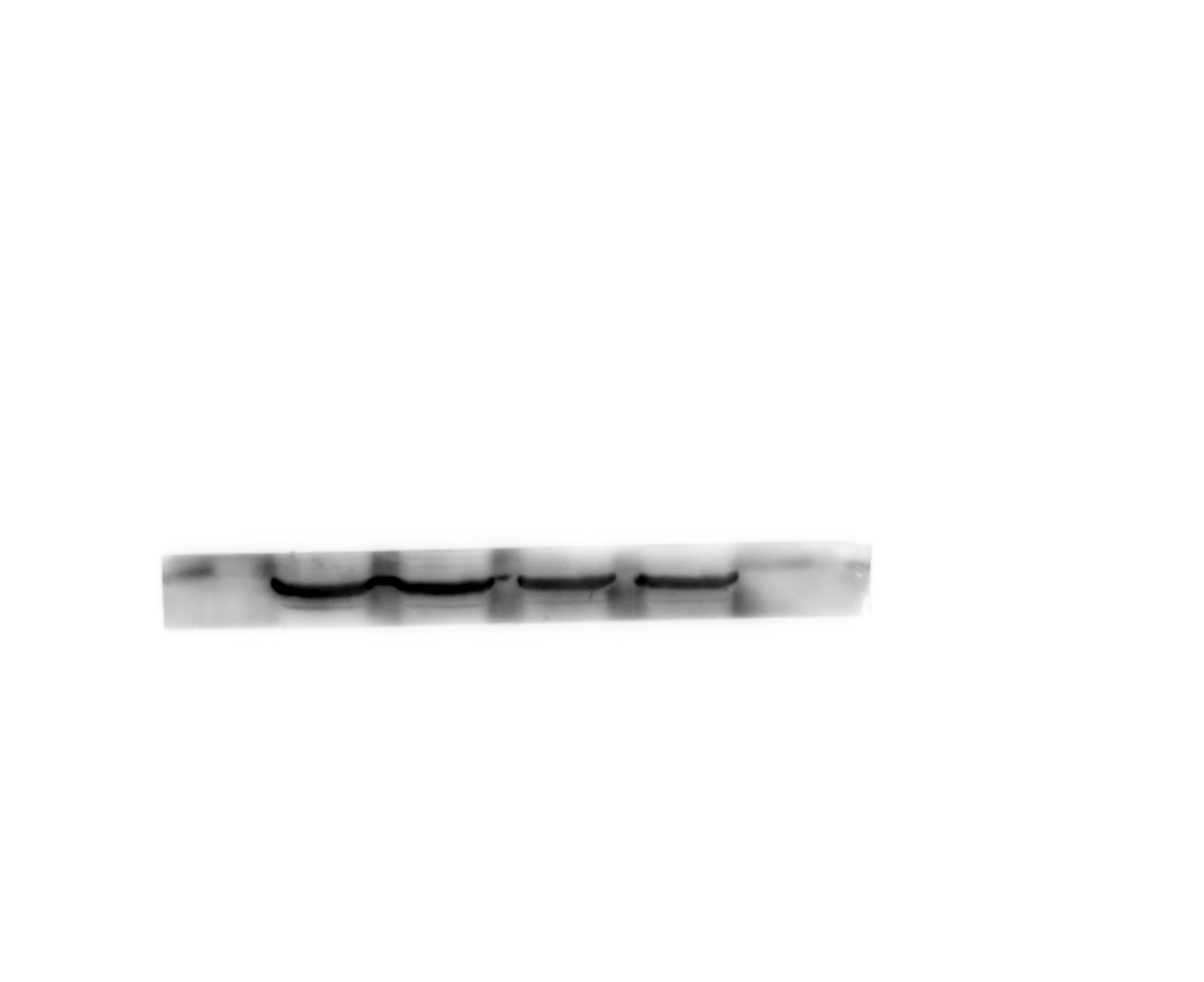

Supplement: Supplementary file 2 [file SupplementaryFile2.zip › WB数据/620-oe/620-wb/actin/ACTIN.tif]

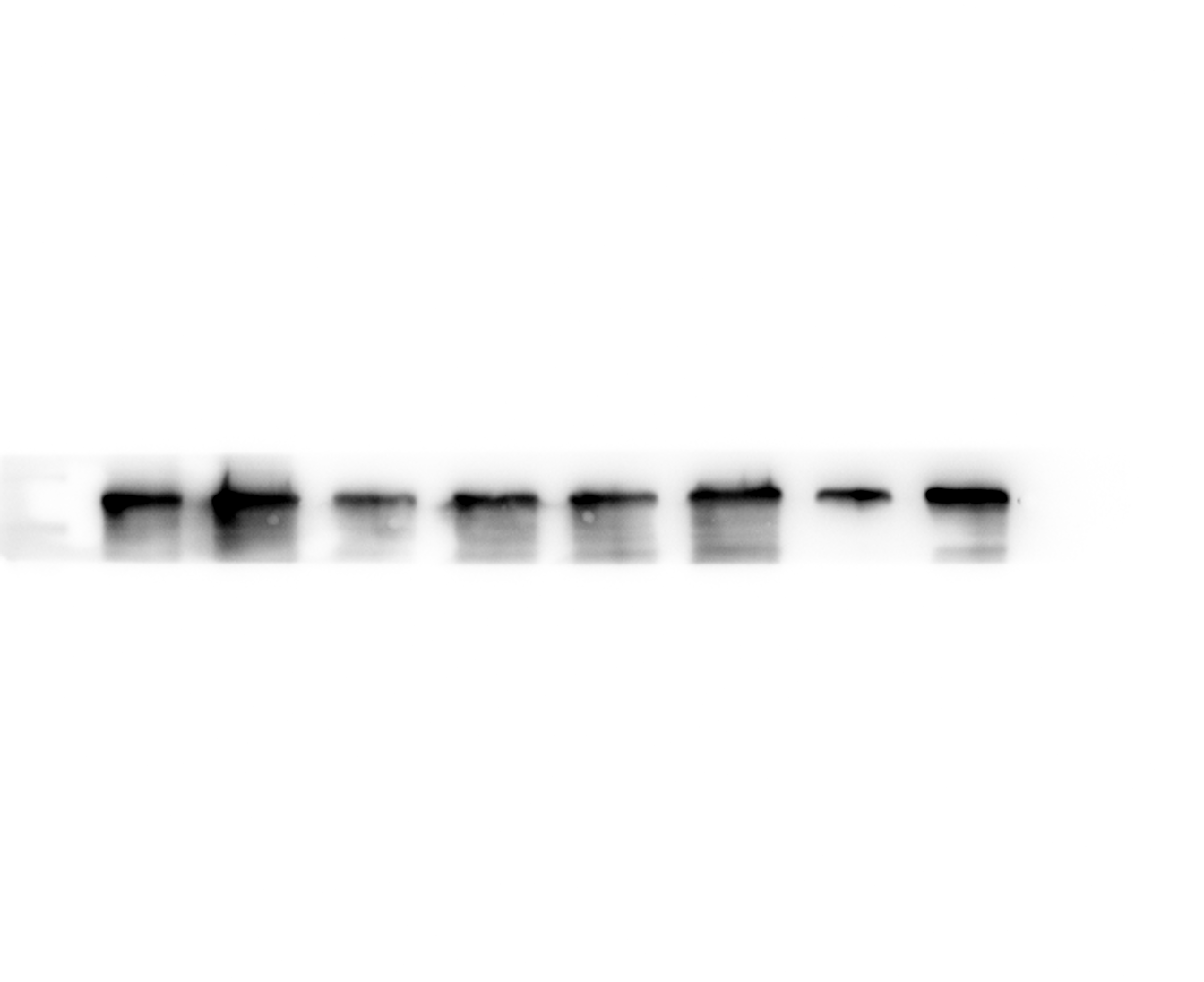

Supplement: Supplementary file 2 [file SupplementaryFile2.zip › WB数据/620-oe/620-wb/e/620-e---.tif]

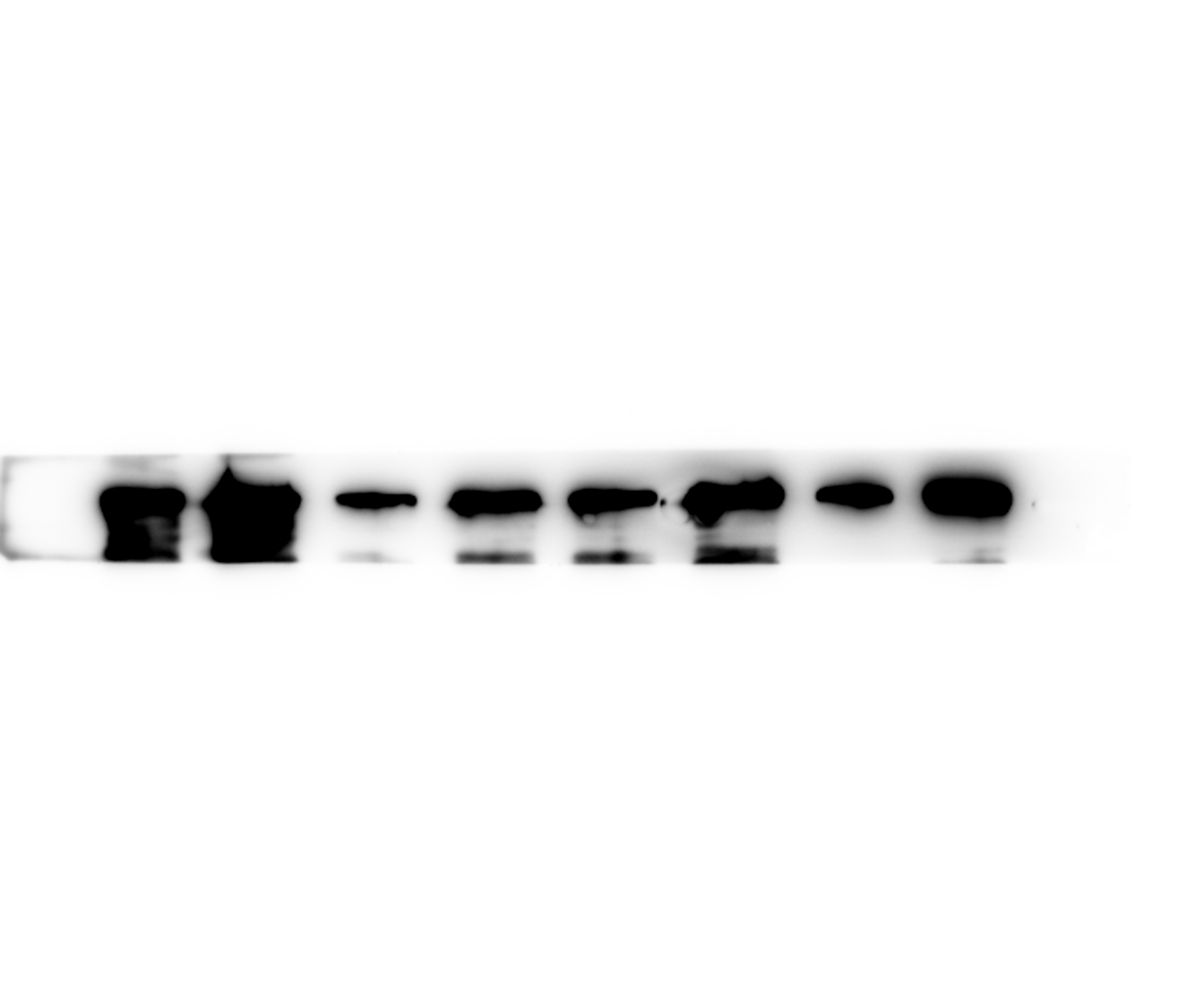

Supplement: Supplementary file 2 [file SupplementaryFile2.zip › WB数据/620-oe/620-wb/e/620-e-.tif]

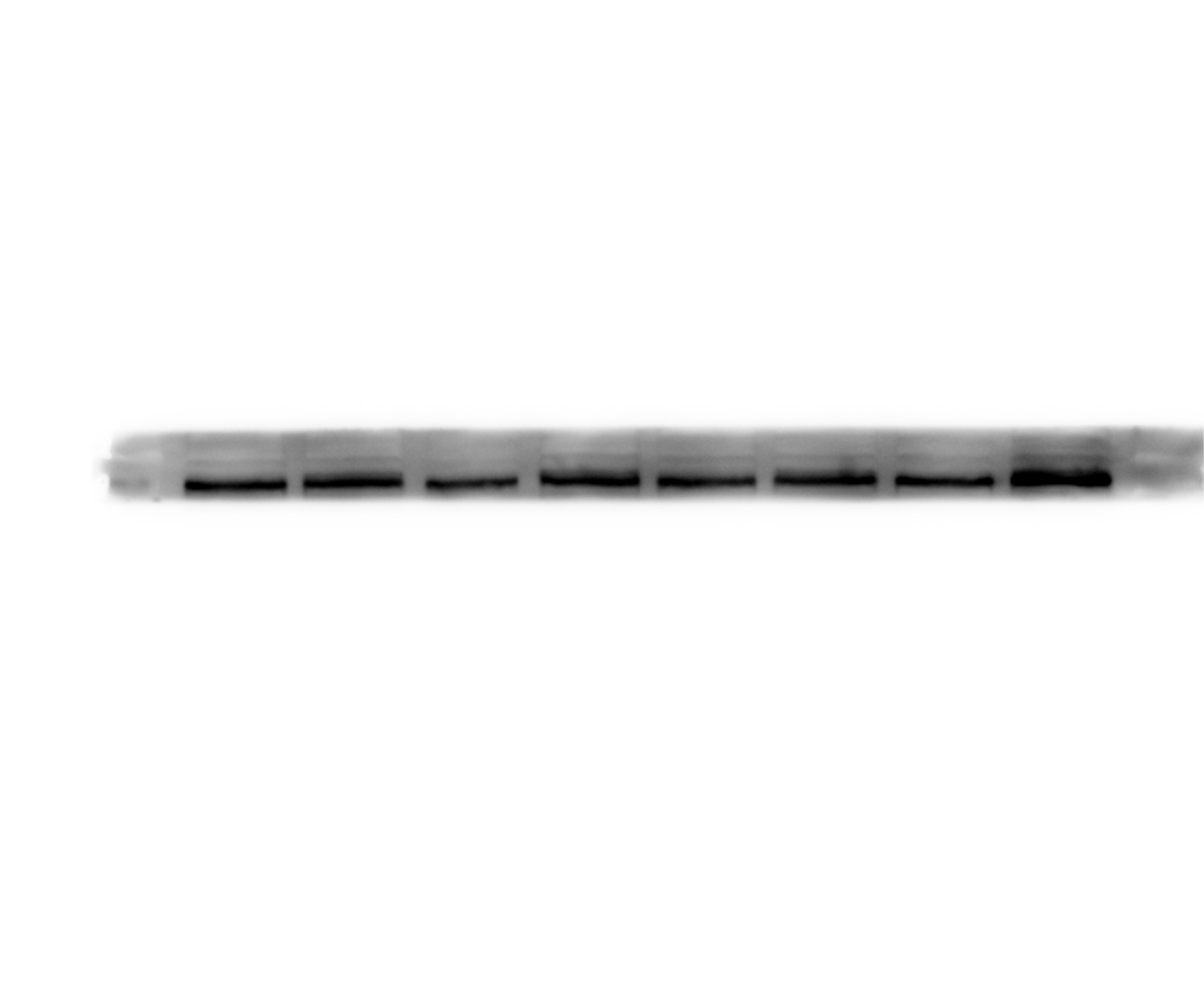

Supplement: Supplementary file 2 [file SupplementaryFile2.zip › WB数据/620-oe/620-wb/e/620-e.png]

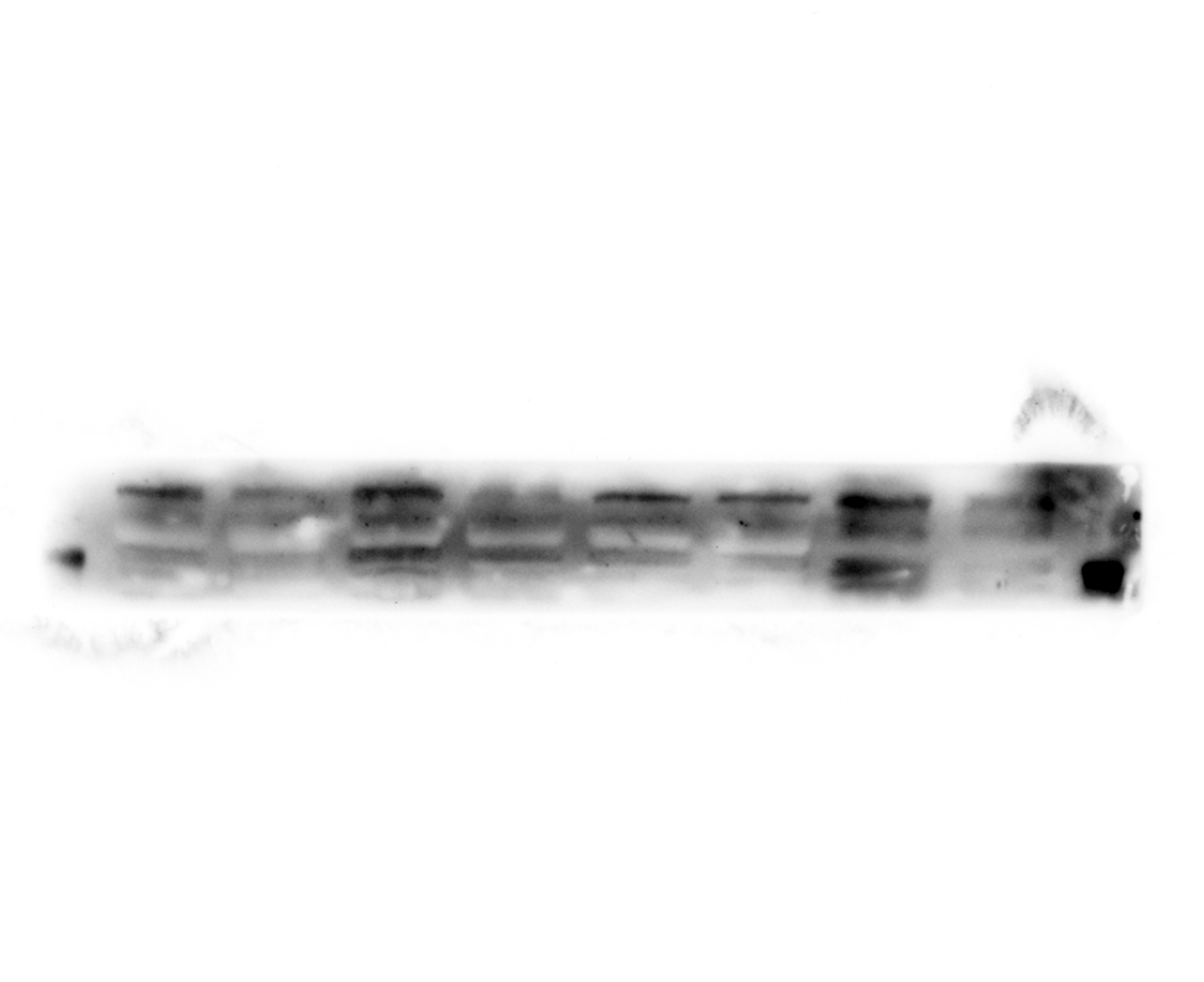

Supplement: Supplementary file 2 [file SupplementaryFile2.zip › WB数据/620-oe/620-wb/mmp9/620-mmp9---.tif]

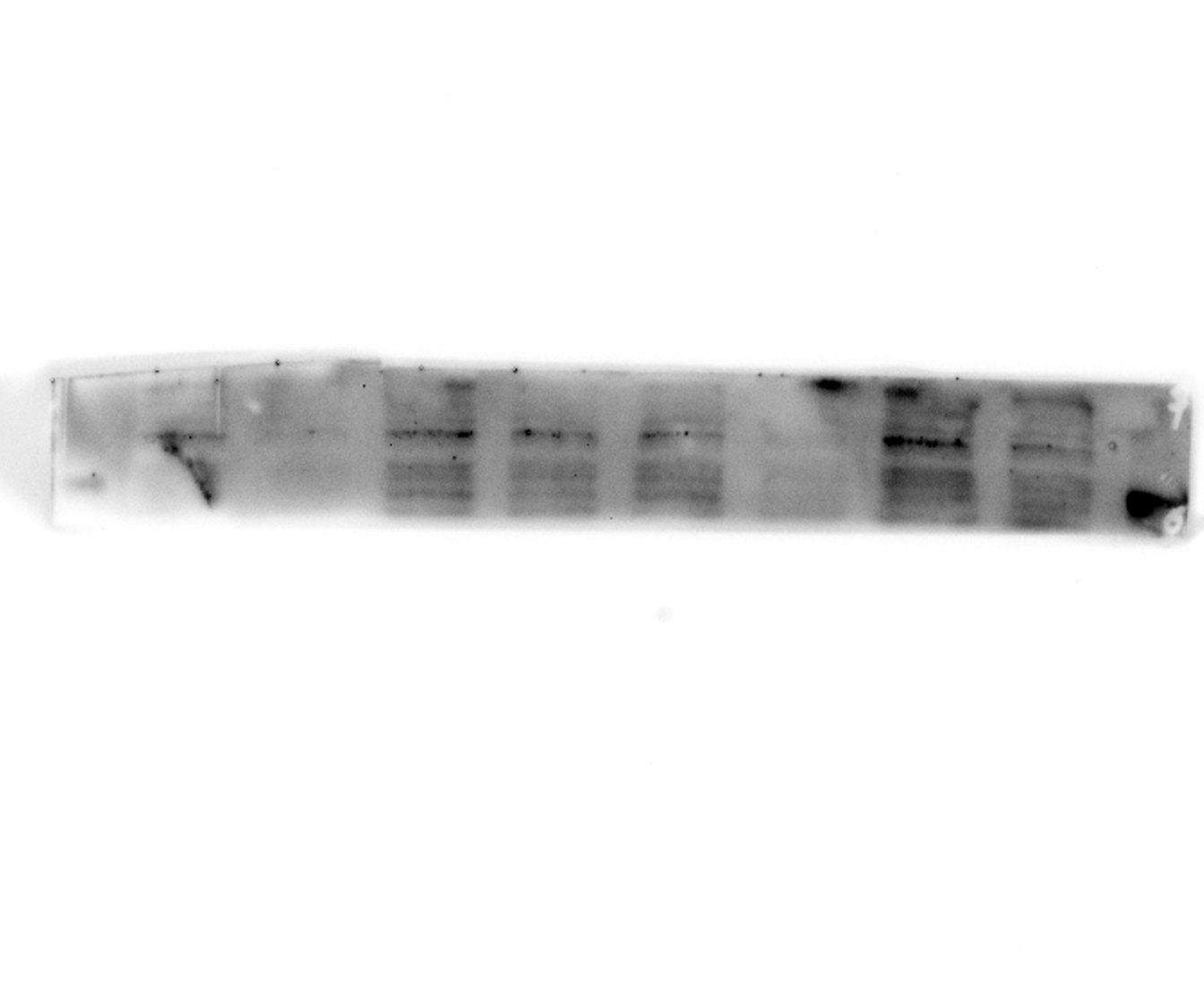

Supplement: Supplementary file 2 [file SupplementaryFile2.zip › WB数据/620-oe/620-wb/mmp9/620-mmp9-.tif]

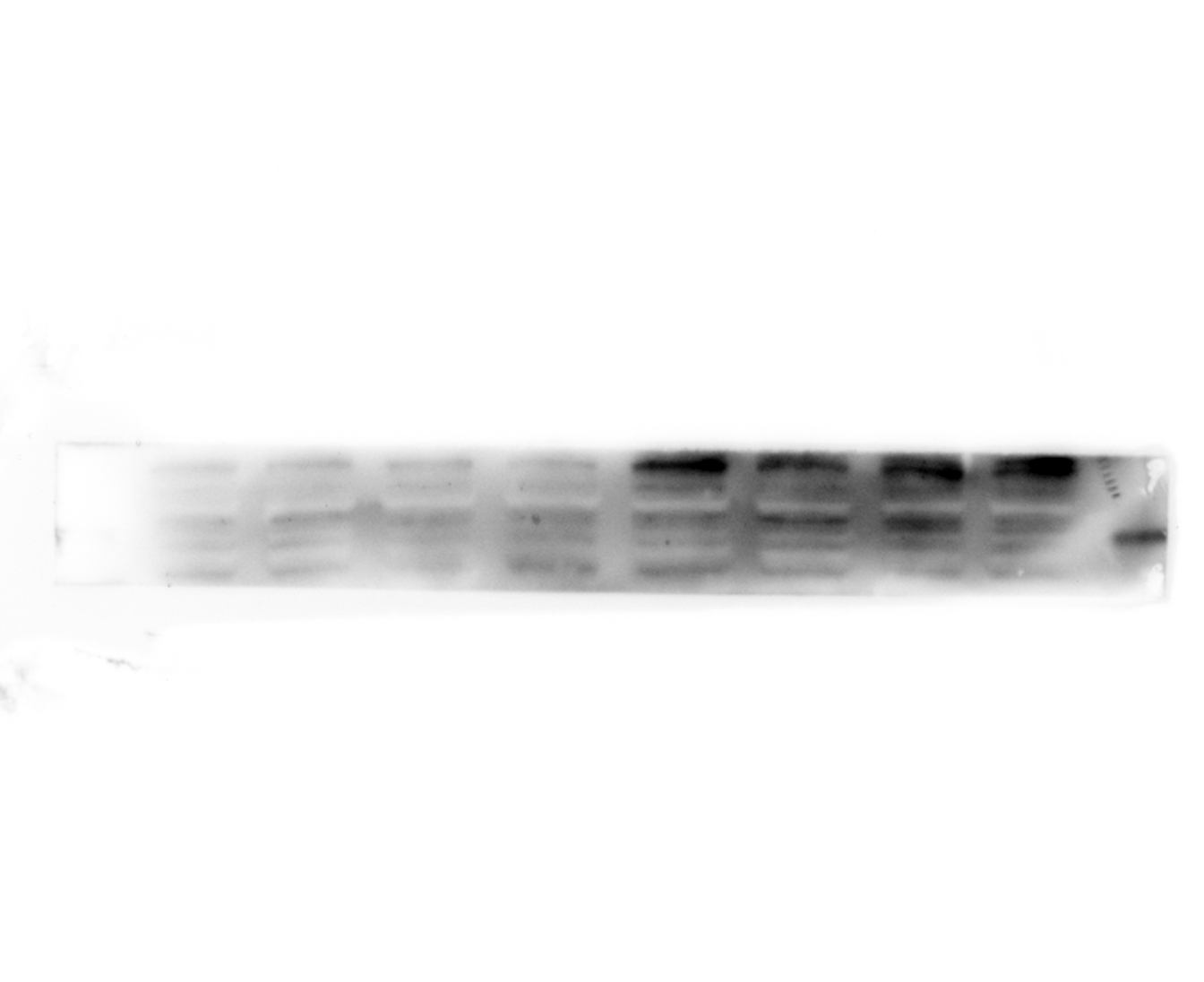

Supplement: Supplementary file 2 [file SupplementaryFile2.zip › WB数据/620-oe/620-wb/mmp9/620-mmp9``.tif]

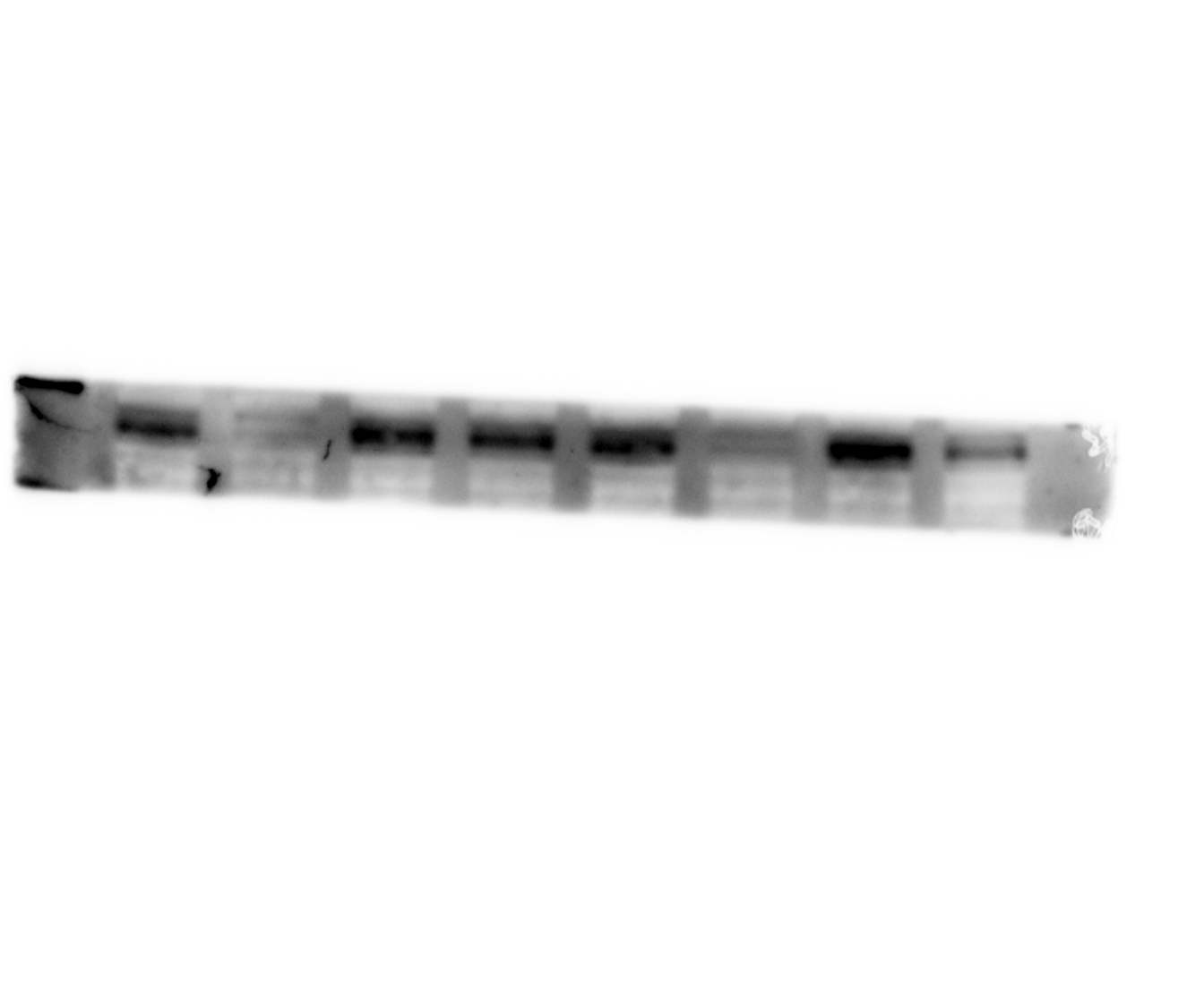

Supplement: Supplementary file 2 [file SupplementaryFile2.zip › WB数据/620-oe/620-wb/snail/620-snail.tif]

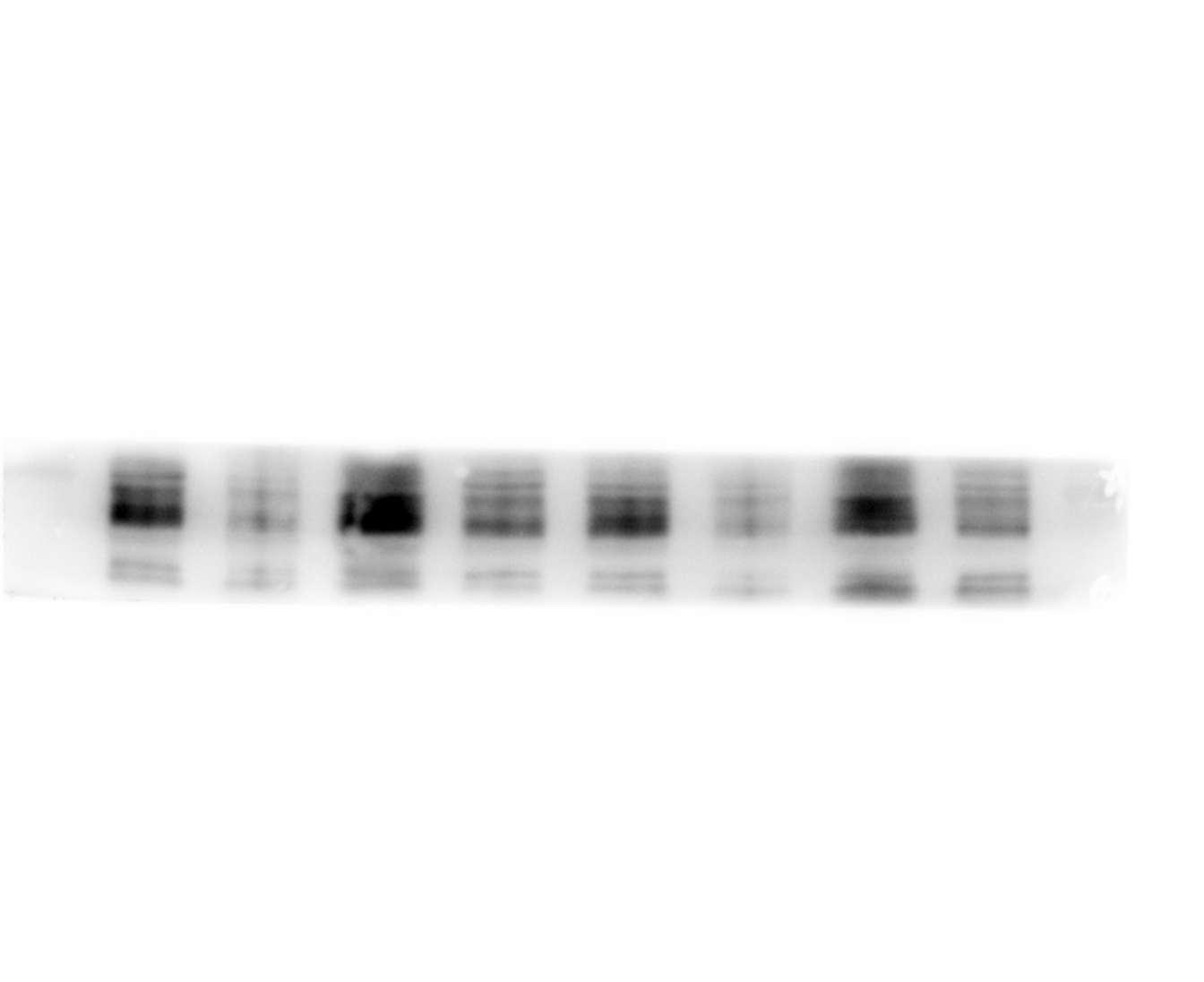

Supplement: Supplementary file 2 [file SupplementaryFile2.zip › WB数据/620-oe/620-wb/snail/620-snail`.tif]

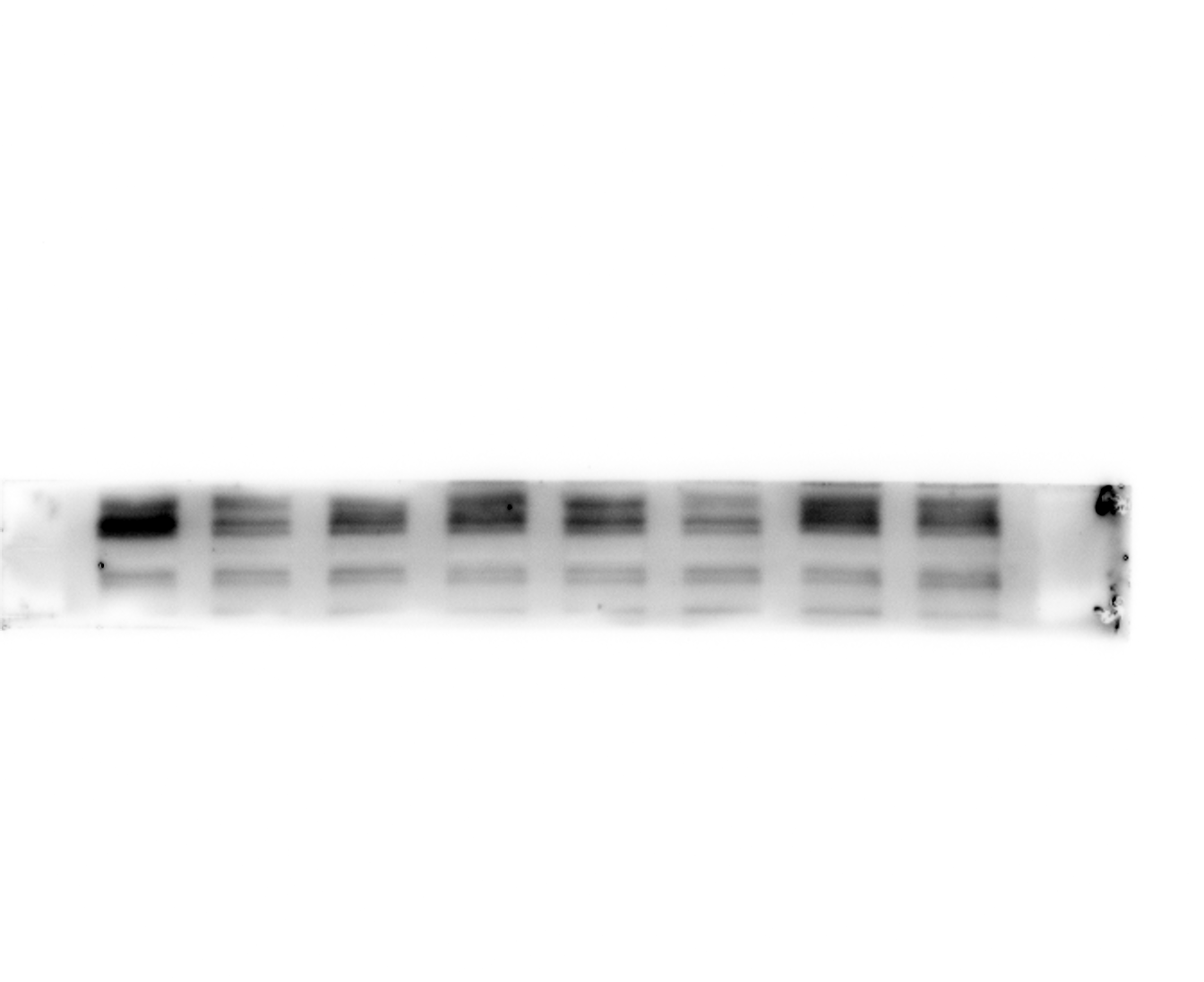

Supplement: Supplementary file 2 [file SupplementaryFile2.zip › WB数据/620-oe/620-wb/snail/620-snail可以用.tif]

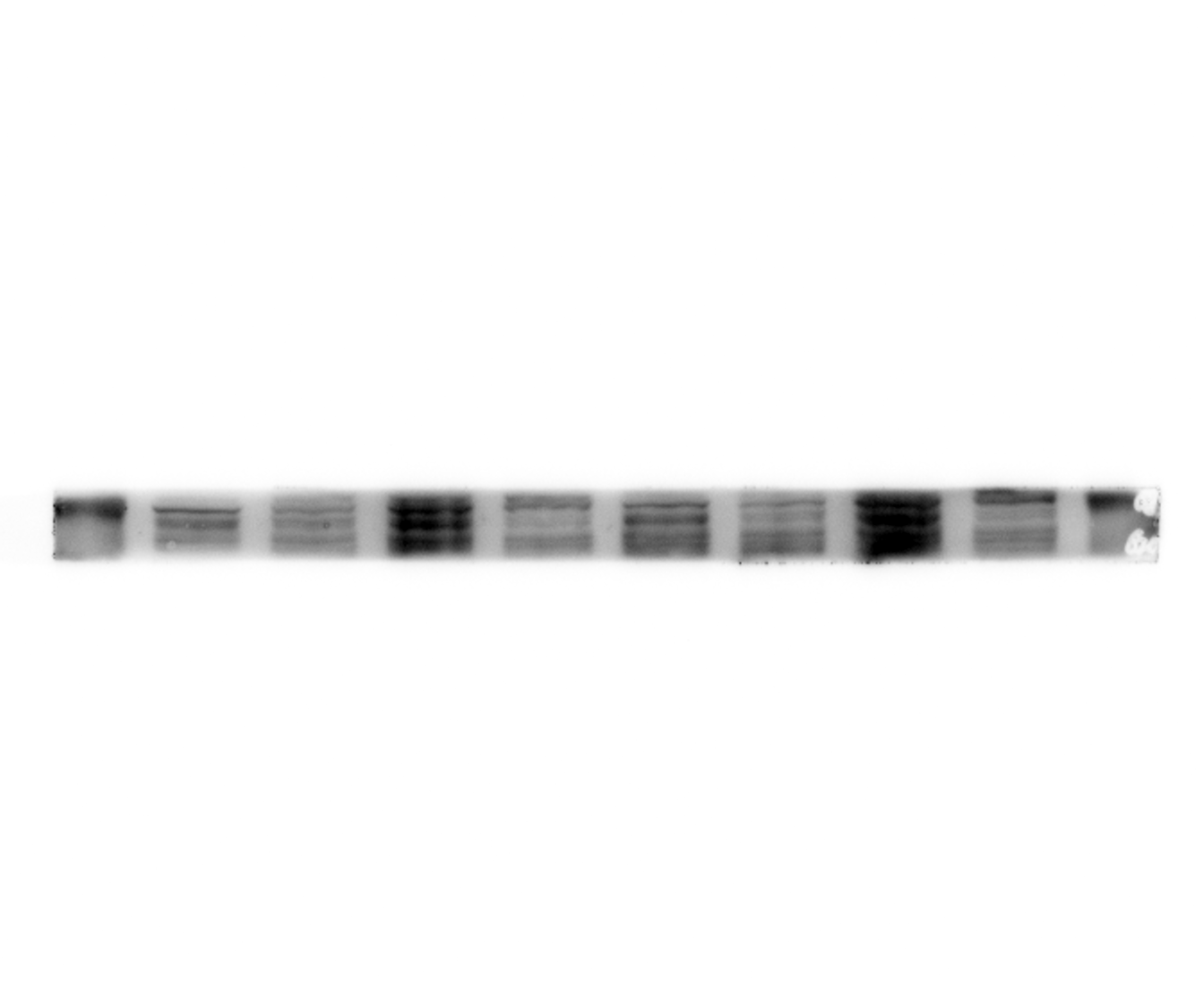

Supplement: Supplementary file 2 [file SupplementaryFile2.zip › WB数据/620-oe/620-wb/srebp1/620-srebp1-68-.tif]

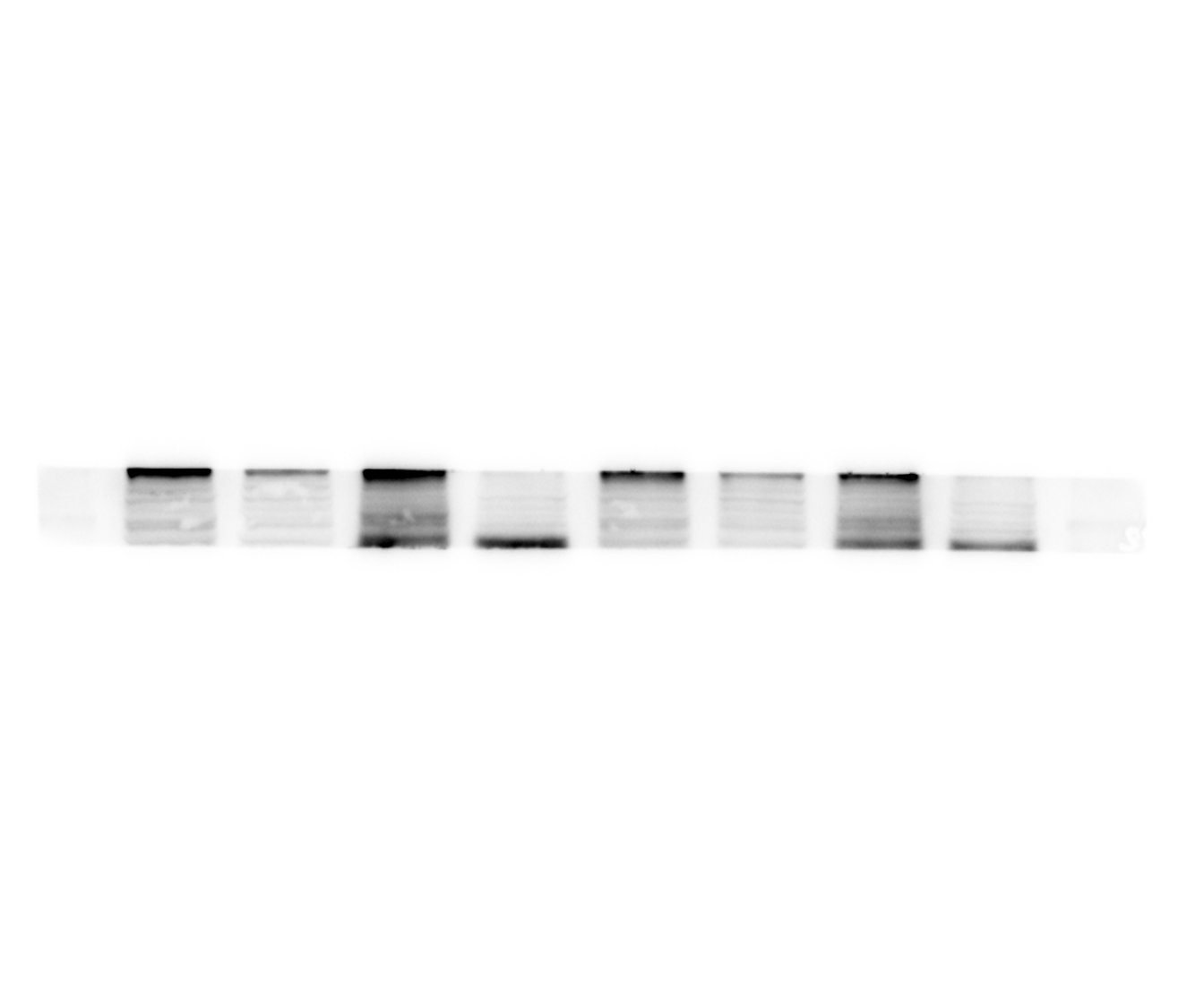

Supplement: Supplementary file 2 [file SupplementaryFile2.zip › WB数据/620-oe/620-wb/srebp1/SREBP1-.tif]

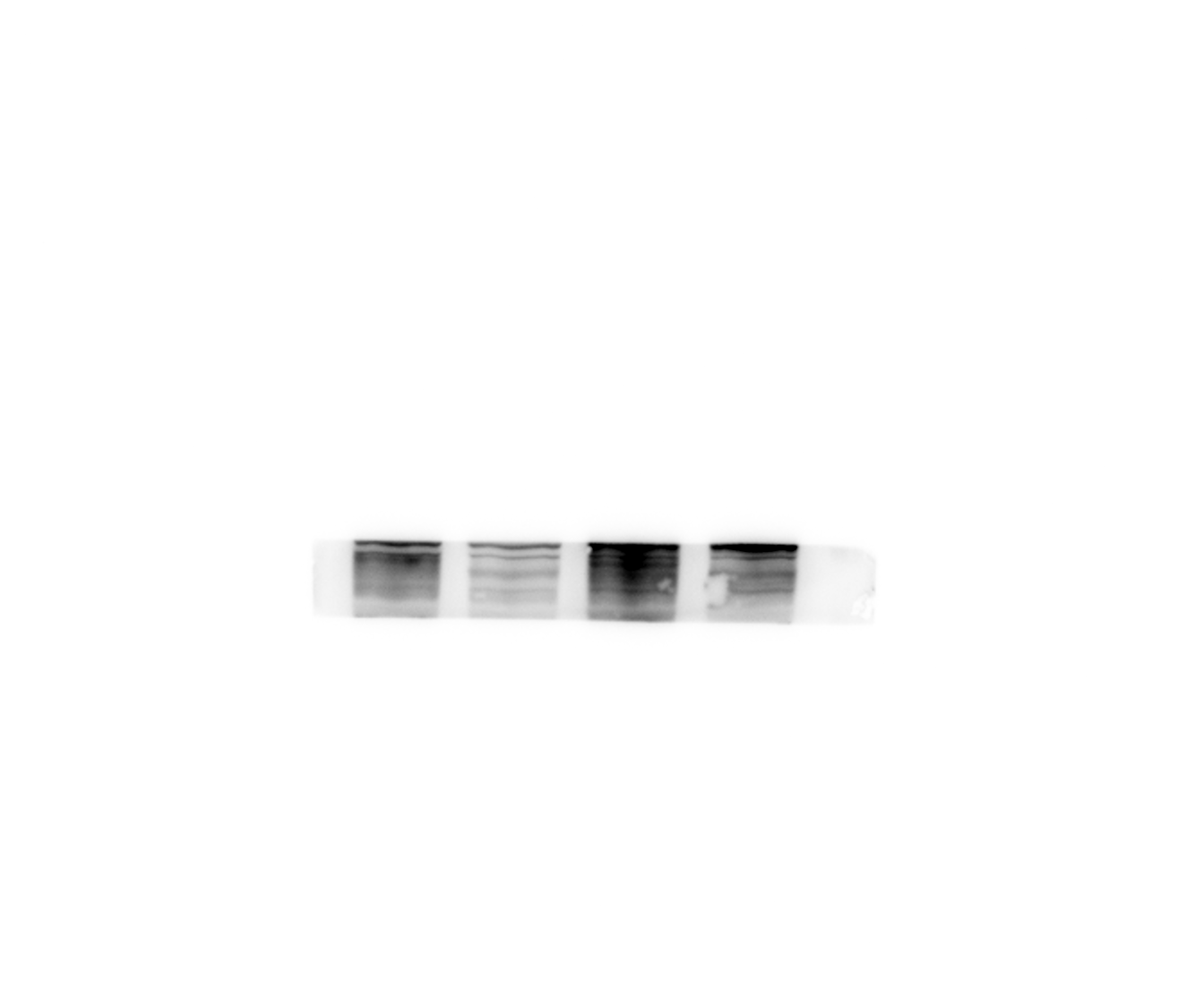

Supplement: Supplementary file 2 [file SupplementaryFile2.zip › WB数据/620-oe/620-wb/srebp1/SREBP1-68-.tif]

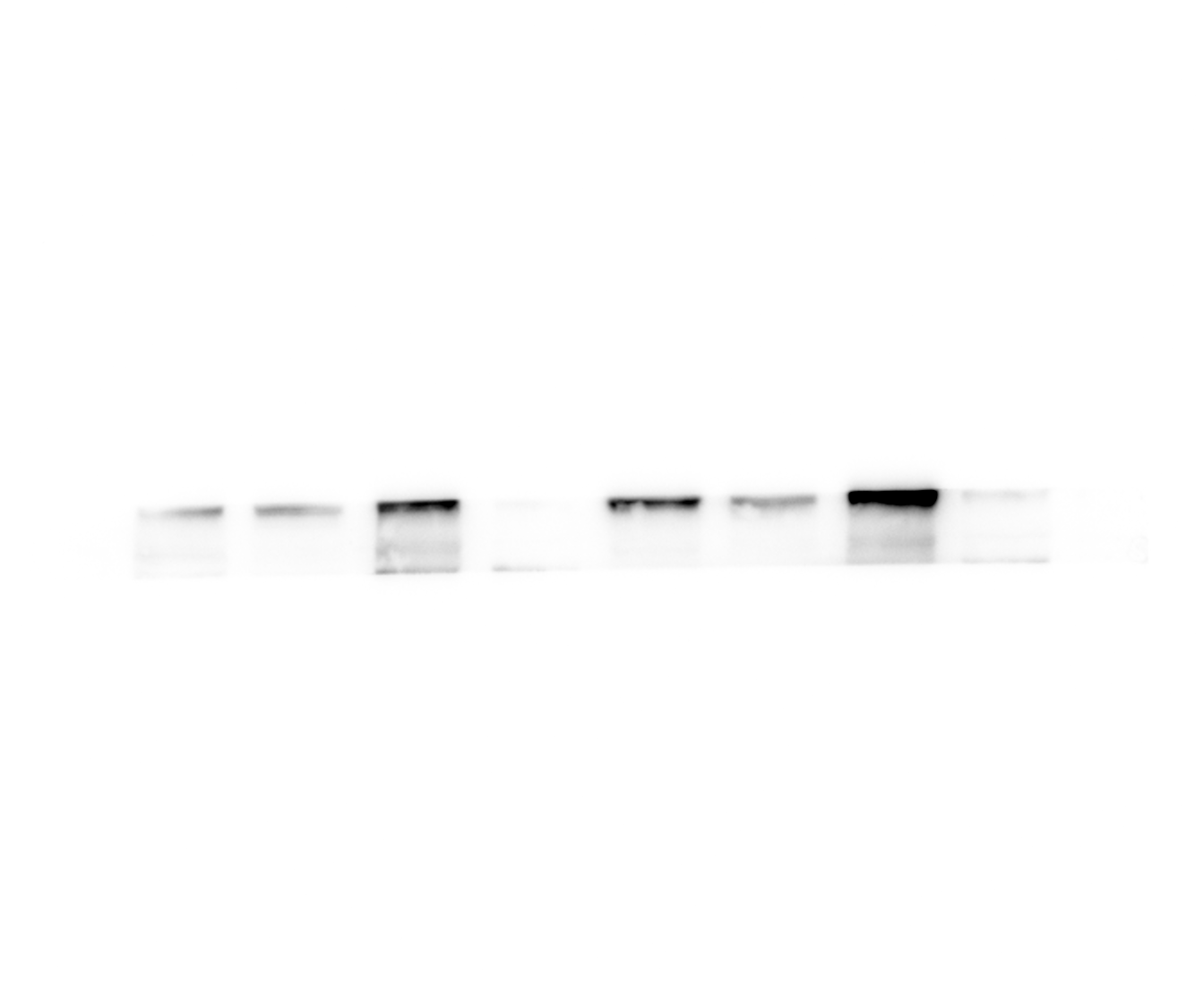

Supplement: Supplementary file 2 [file SupplementaryFile2.zip › WB数据/620-oe/620-wb/srebp1/srebp1.tif]

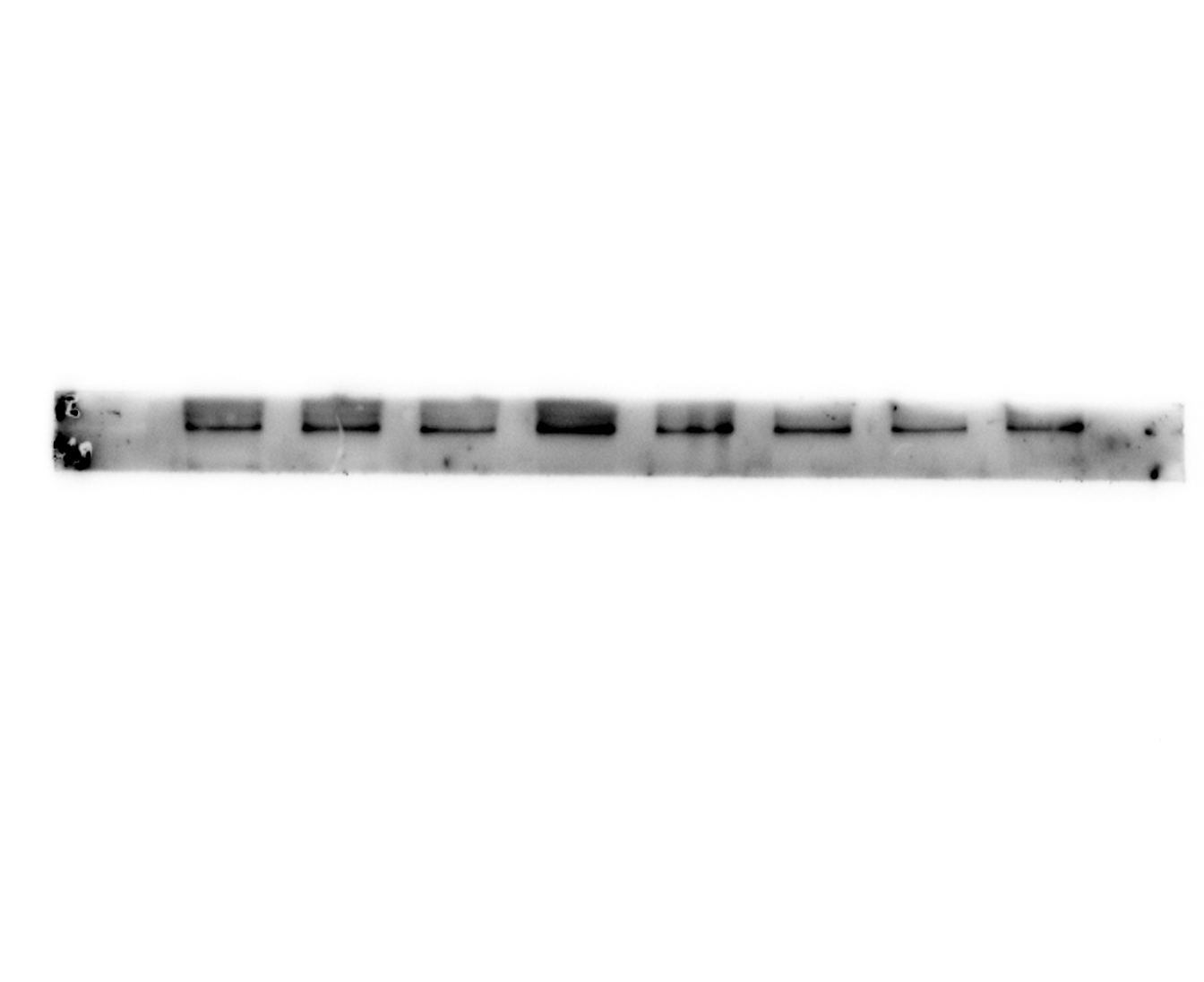

Supplement: Supplementary file 2 [file SupplementaryFile2.zip › WB数据/620-oe/620-wb/zo1/620-zo1--.png]

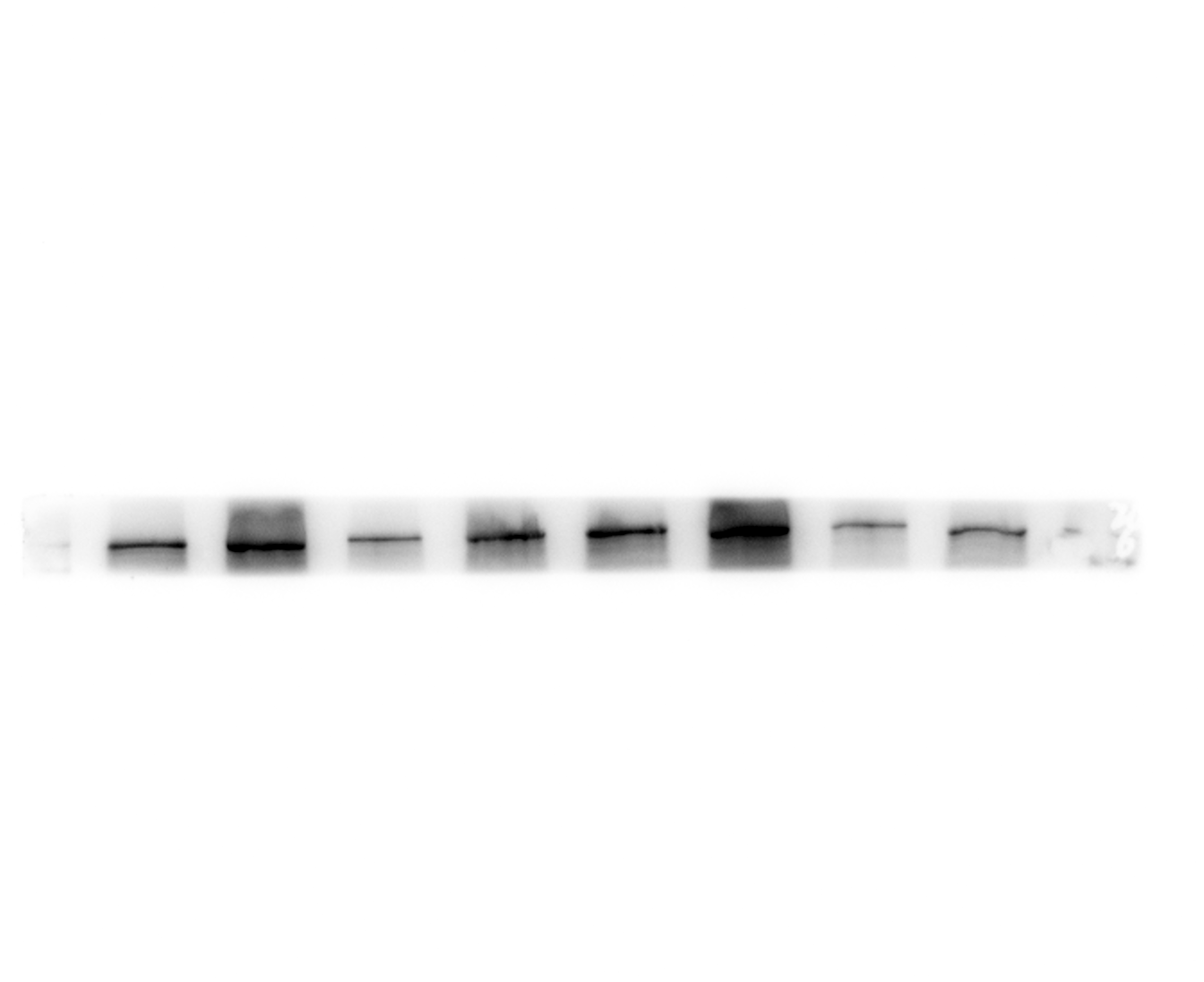

Supplement: Supplementary file 2 [file SupplementaryFile2.zip › WB数据/620-oe/620-wb/zo1/620-zo1.tif]

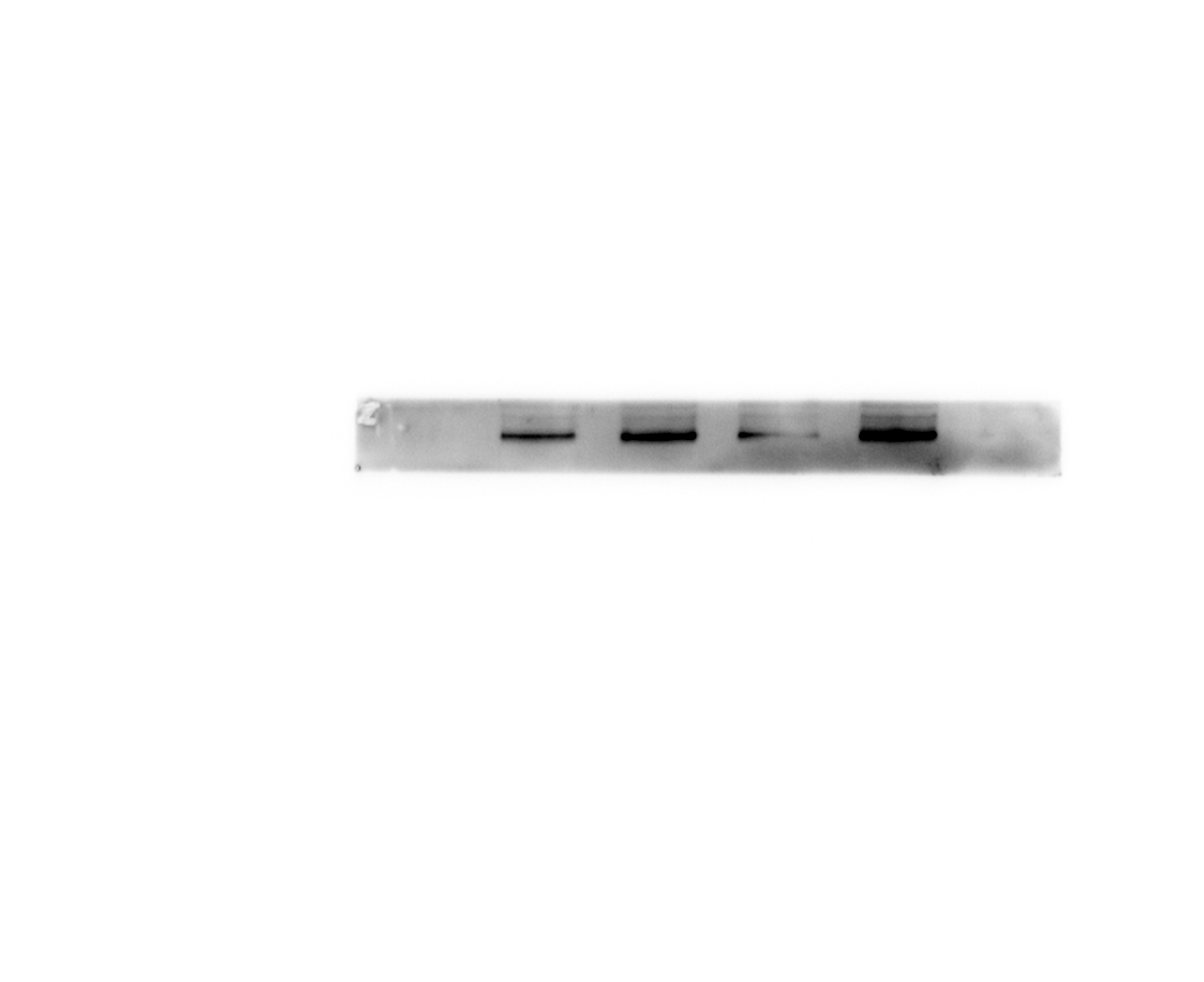

Supplement: Supplementary file 2 [file SupplementaryFile2.zip › WB数据/620-oe/620-wb/zo1/zo1-.png]

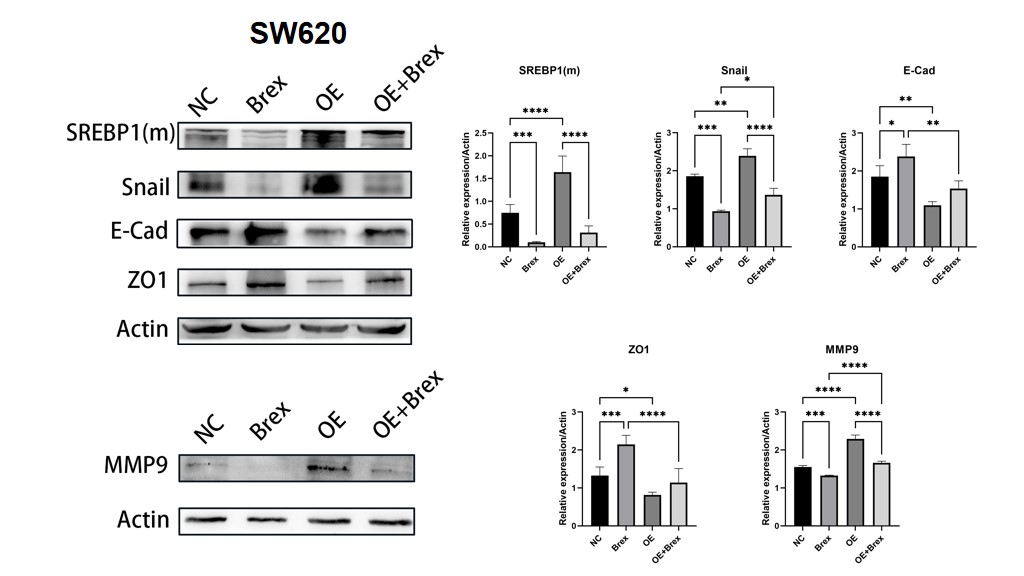

Supplement: Supplementary file 2 [file SupplementaryFile2.zip › WB数据/620-oe/数据图/1.jpg]

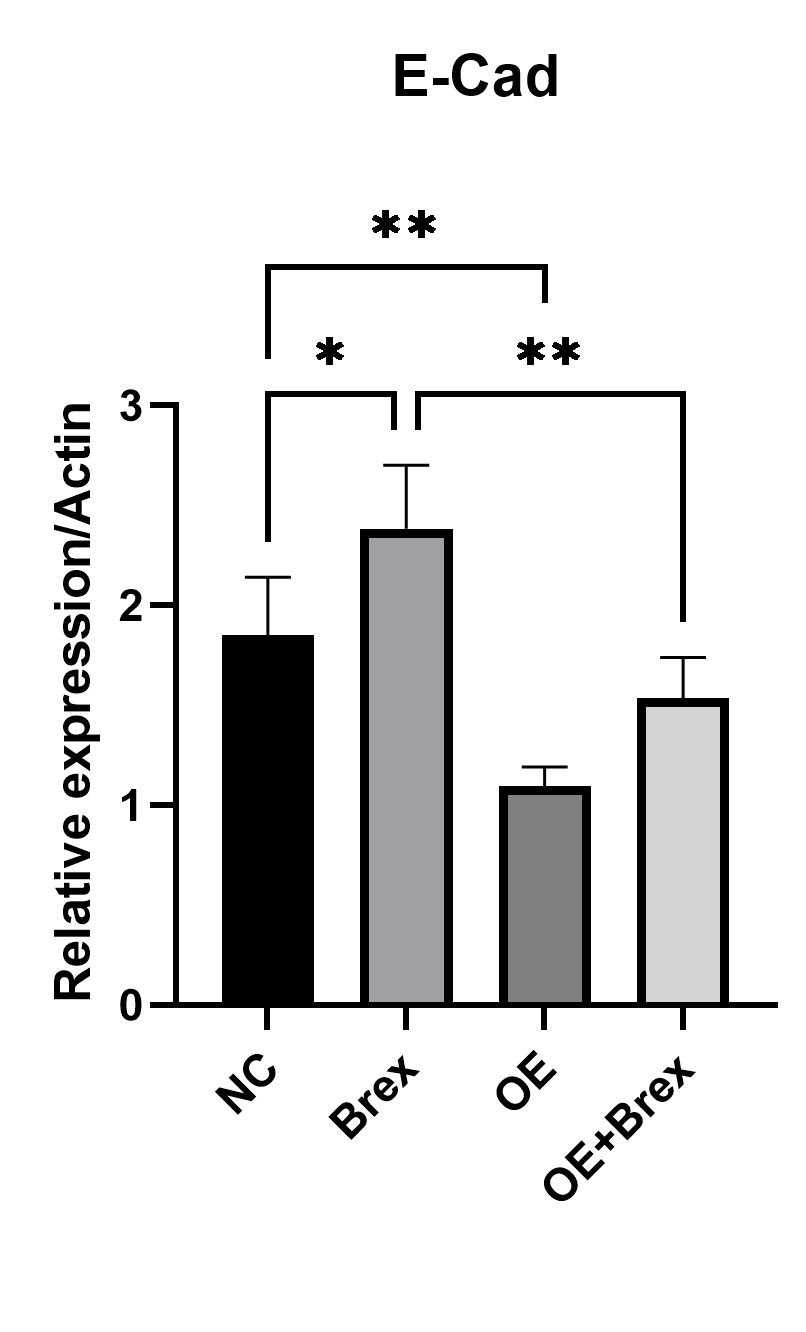

Supplement: Supplementary file 2 [file SupplementaryFile2.zip › WB数据/620-oe/数据图/E.tif]

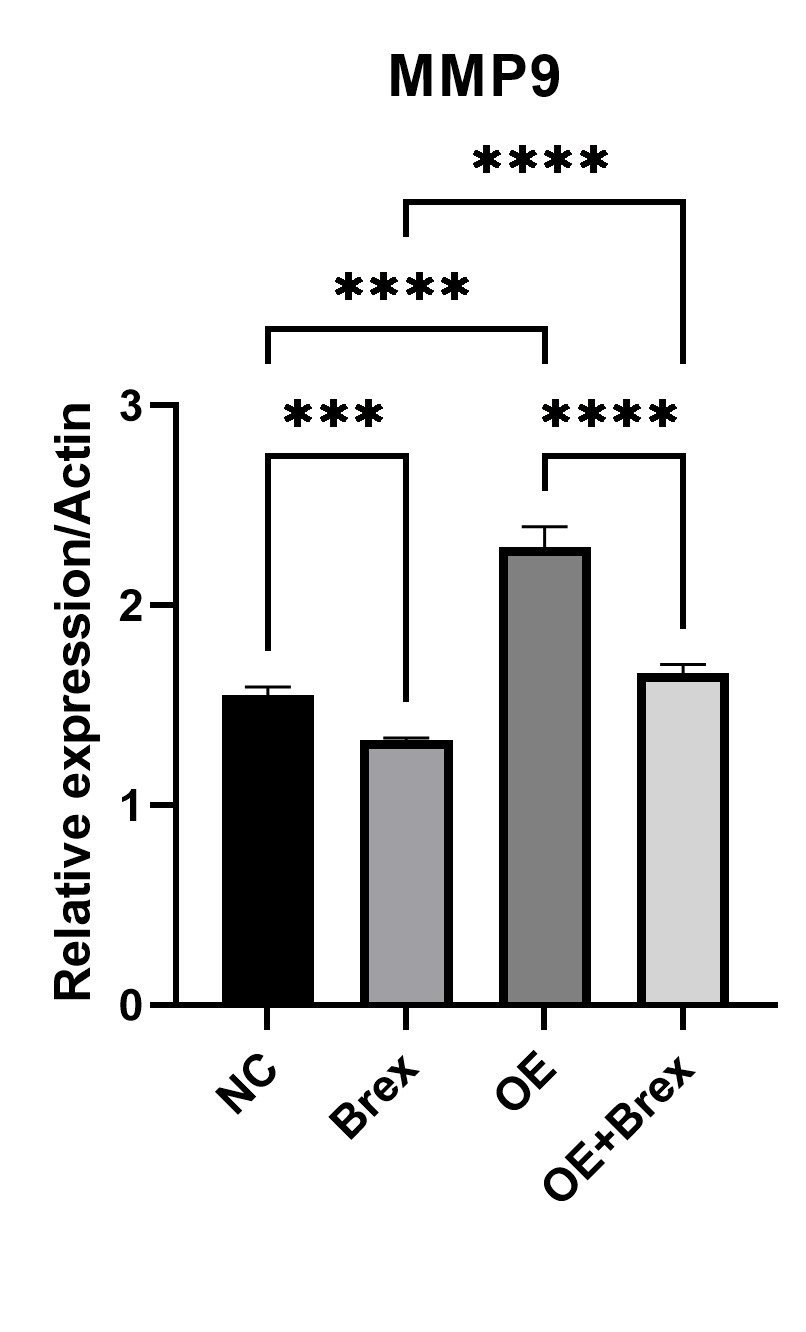

Supplement: Supplementary file 2 [file SupplementaryFile2.zip › WB数据/620-oe/数据图/MMP9.tif]

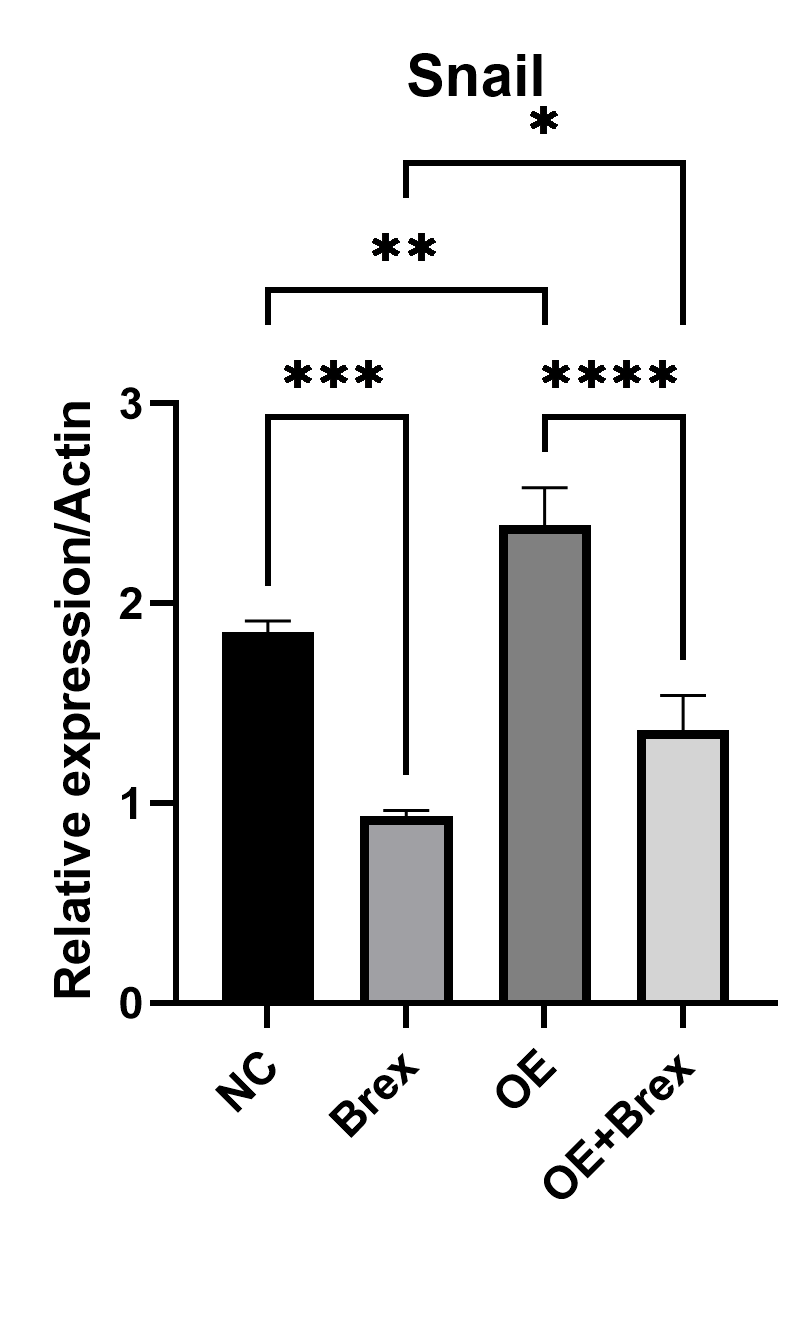

Supplement: Supplementary file 2 [file SupplementaryFile2.zip › WB数据/620-oe/数据图/SNAIL.tif]

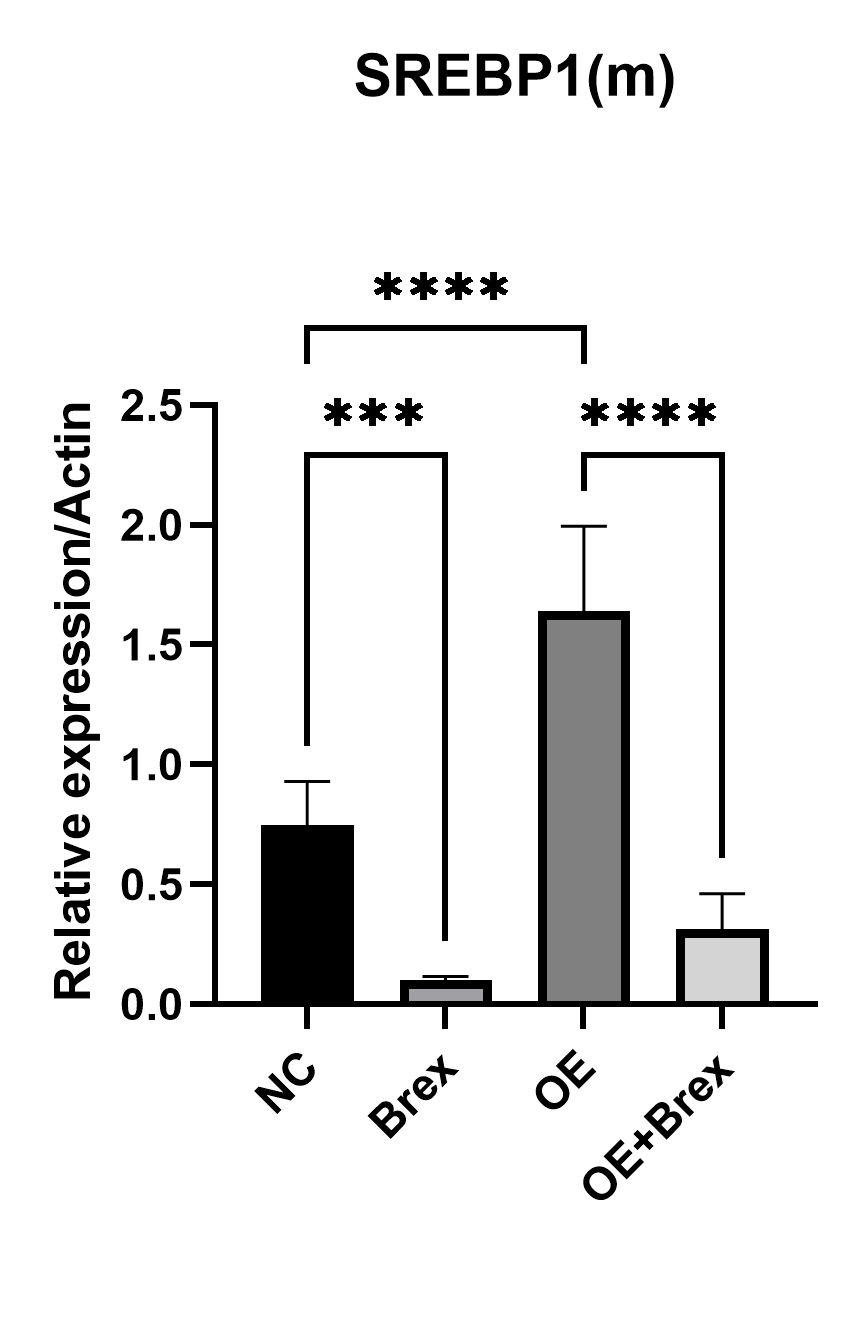

Supplement: Supplementary file 2 [file SupplementaryFile2.zip › WB数据/620-oe/数据图/SREBP1.tif]

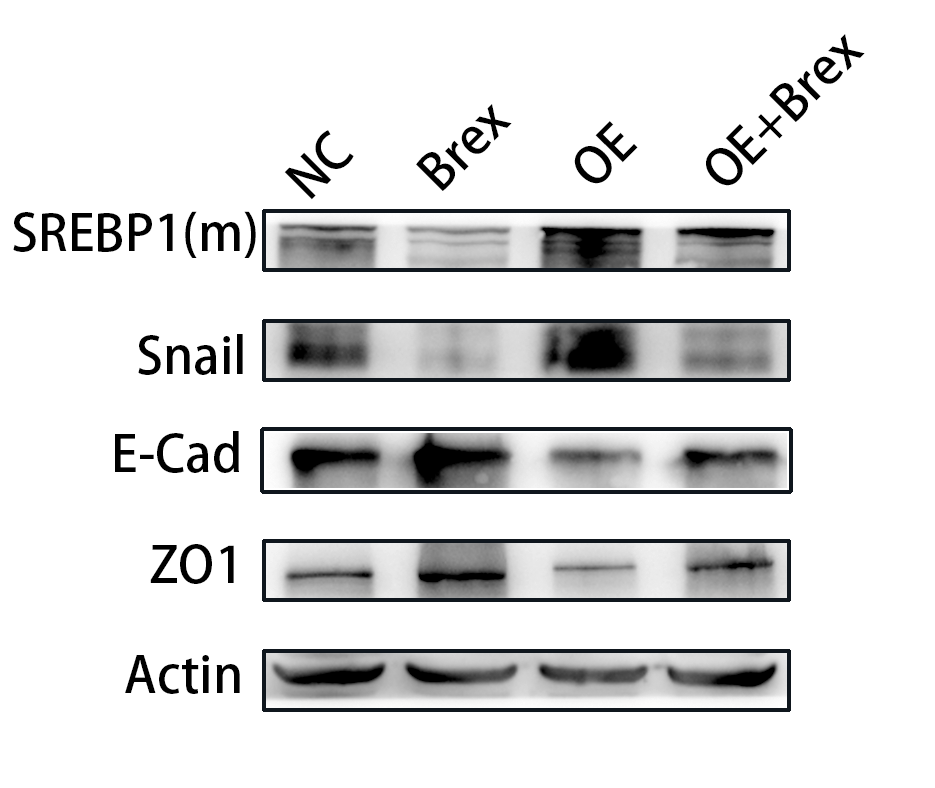

Supplement: Supplementary file 2 [file SupplementaryFile2.zip › WB数据/620-oe/数据图/SW620-OE--.png]

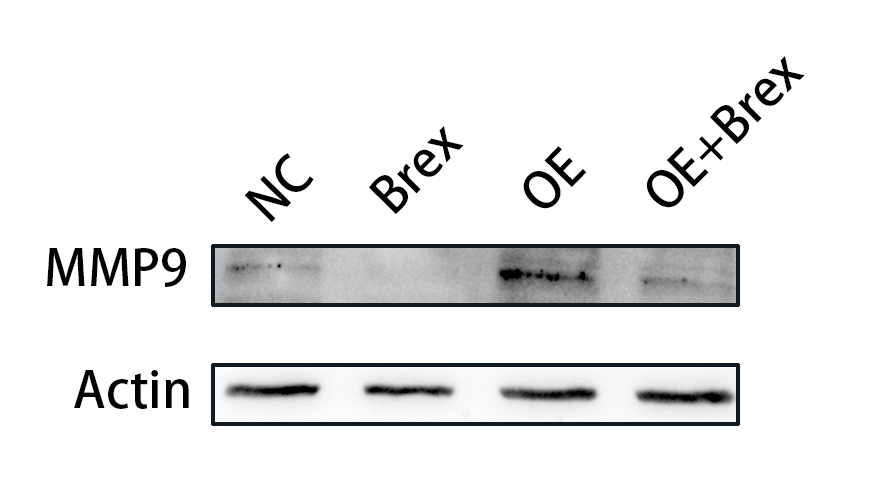

Supplement: Supplementary file 2 [file SupplementaryFile2.zip › WB数据/620-oe/数据图/sw620-OE-MMP9.png]

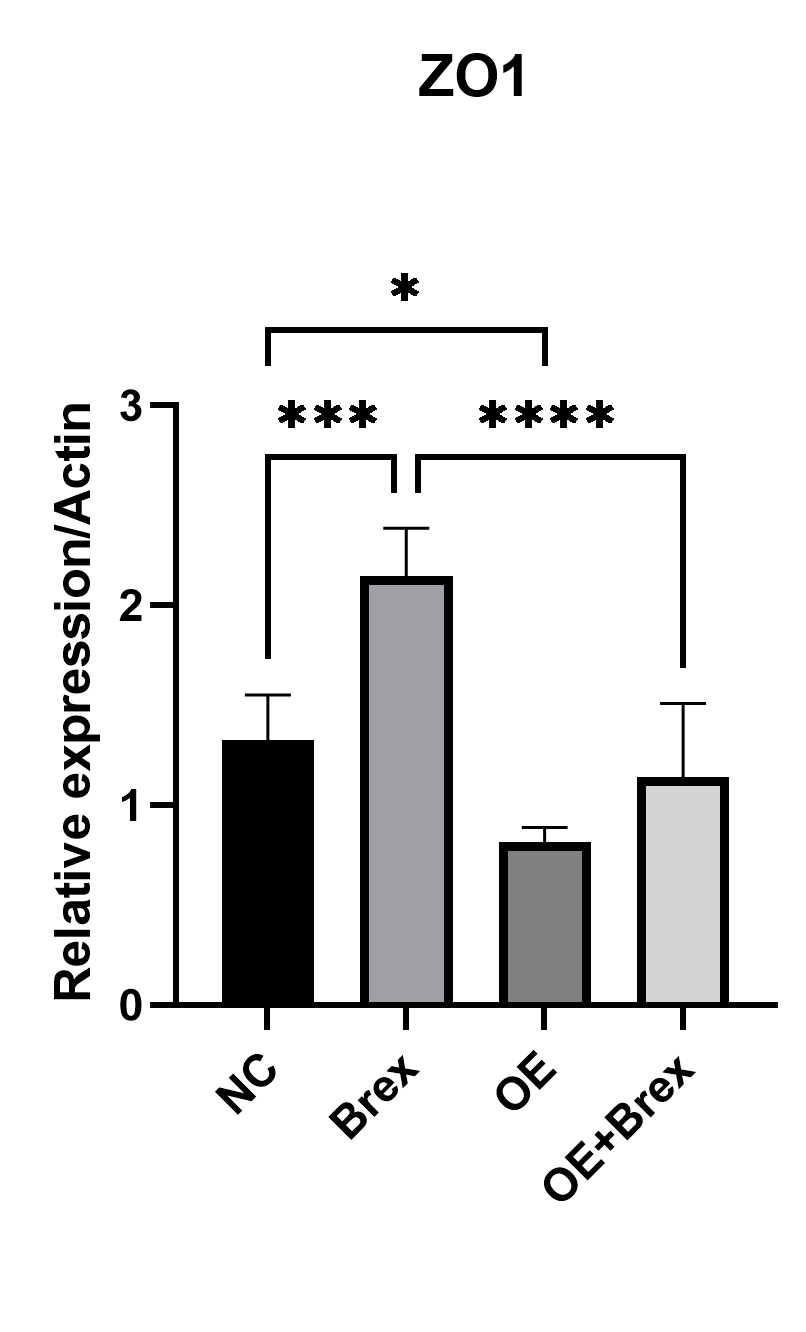

Supplement: Supplementary file 2 [file SupplementaryFile2.zip › WB数据/620-oe/数据图/ZO1.tif]

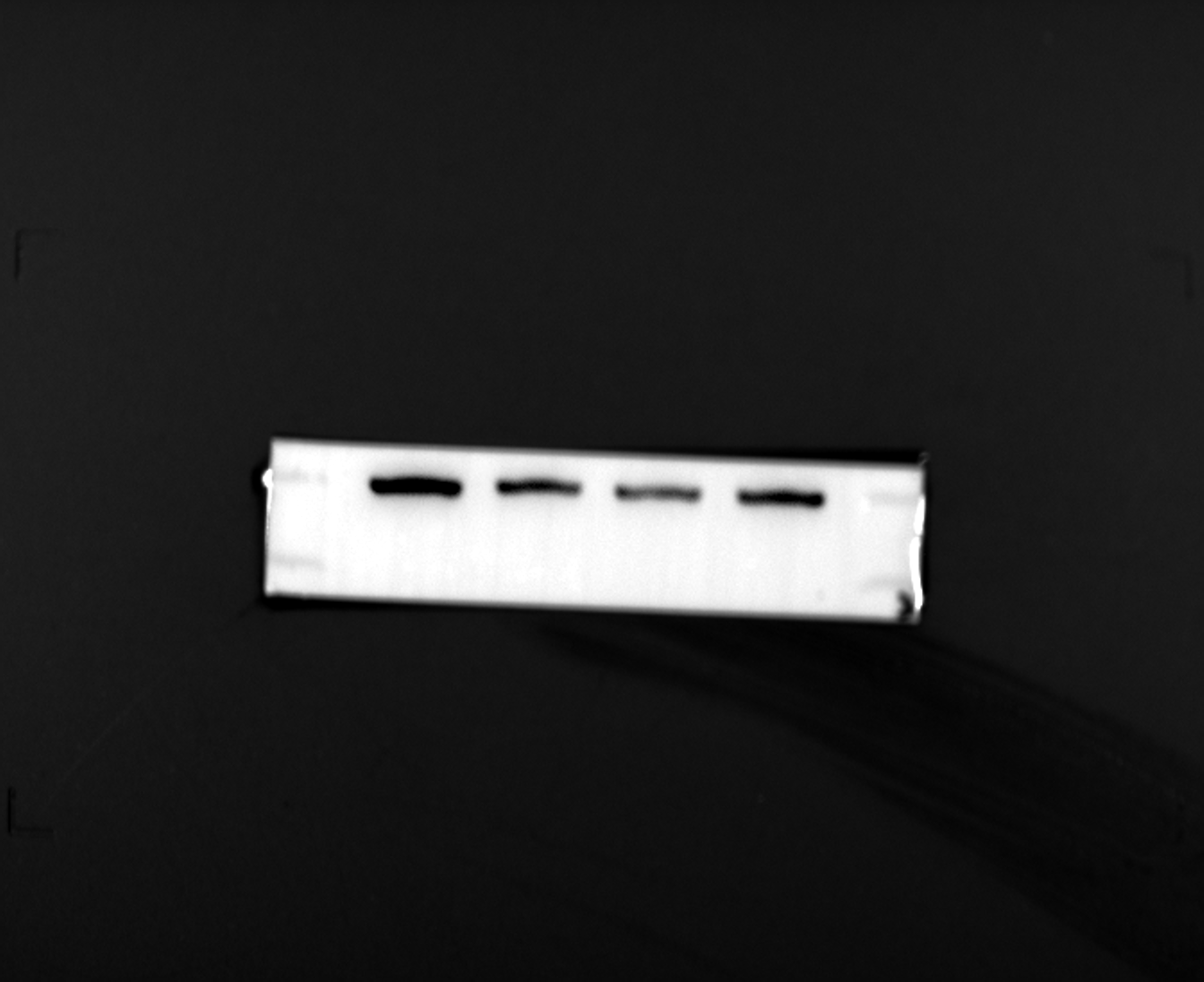

Supplement: Supplementary file 2 [file SupplementaryFile2.zip › WB数据/620-si/actin/620-actin-merge-.tif]

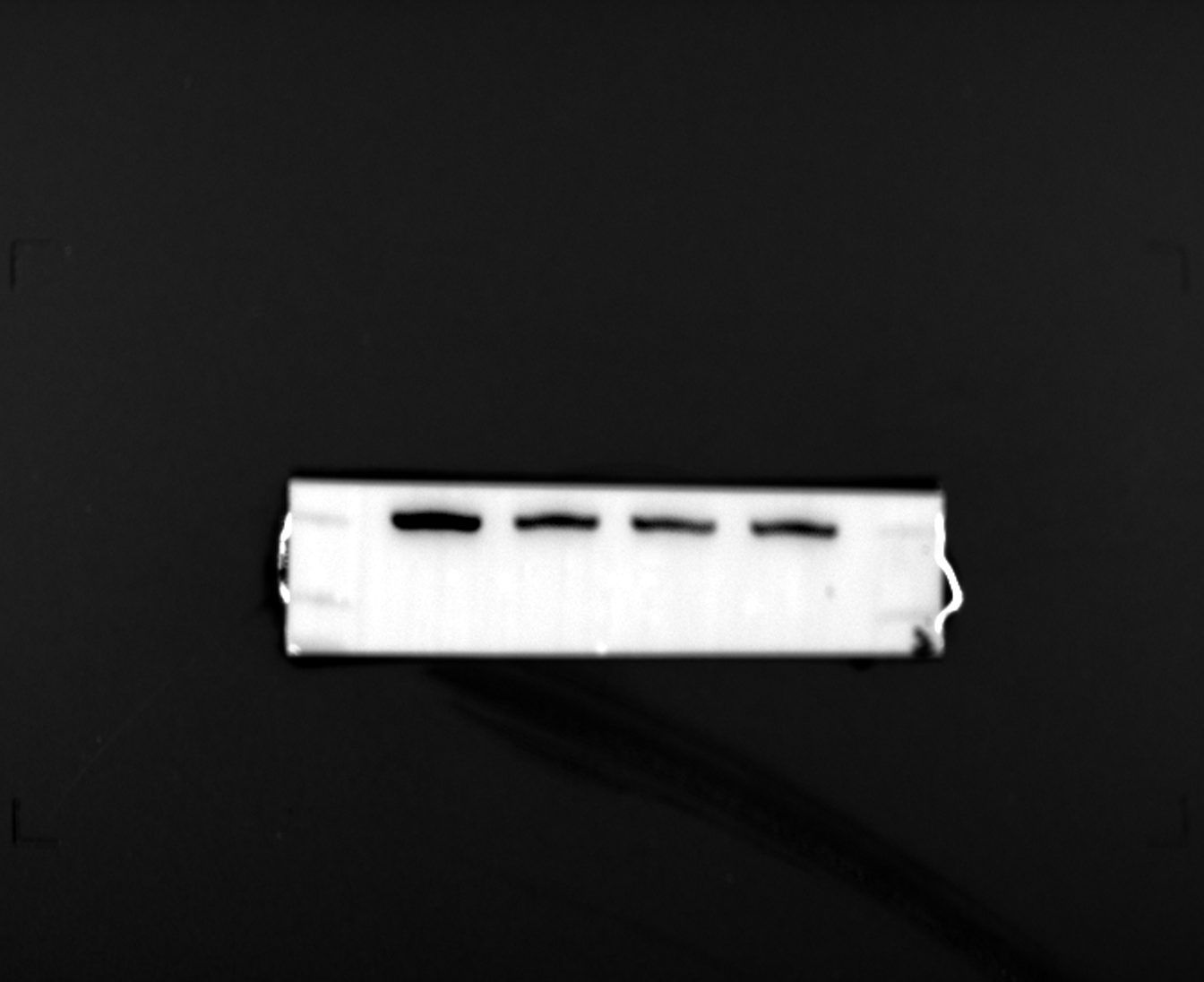

Supplement: Supplementary file 2 [file SupplementaryFile2.zip › WB数据/620-si/actin/620-actin-merge.tif]

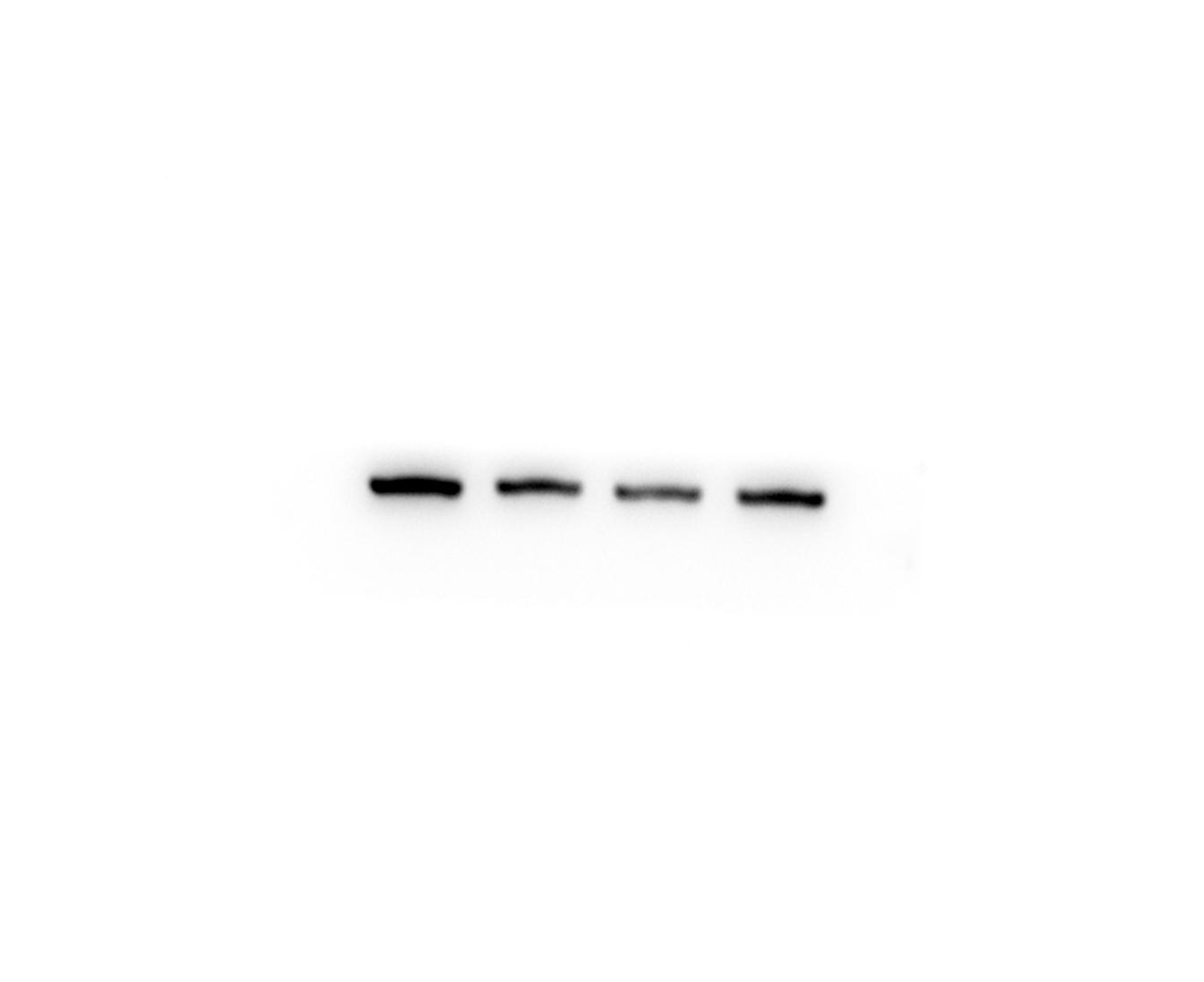

Supplement: Supplementary file 2 [file SupplementaryFile2.zip › WB数据/620-si/actin/620-actin-`.tif]

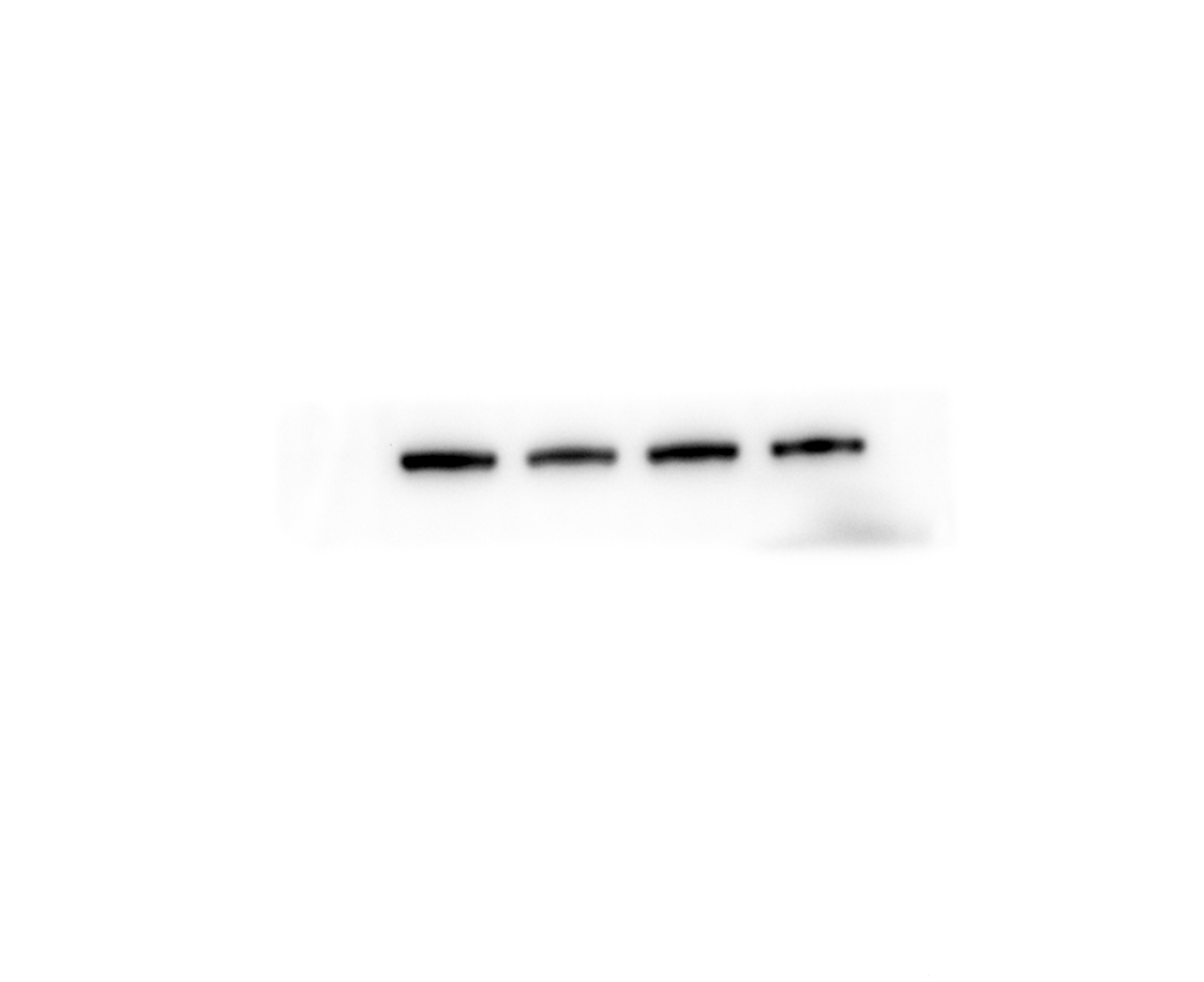

Supplement: Supplementary file 2 [file SupplementaryFile2.zip › WB数据/620-si/actin/620-actin.tif]

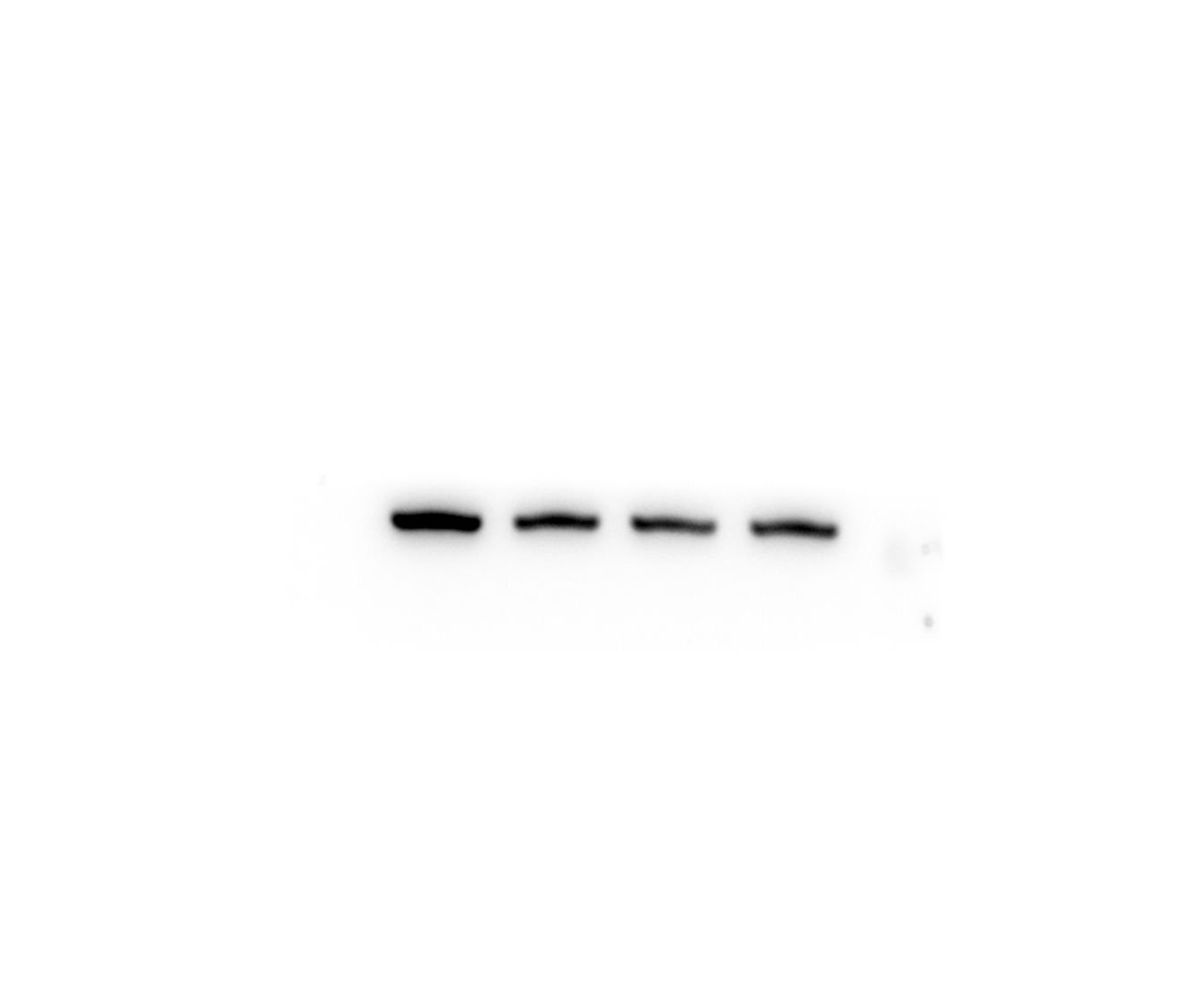

Supplement: Supplementary file 2 [file SupplementaryFile2.zip › WB数据/620-si/actin/620-actin`.tif]

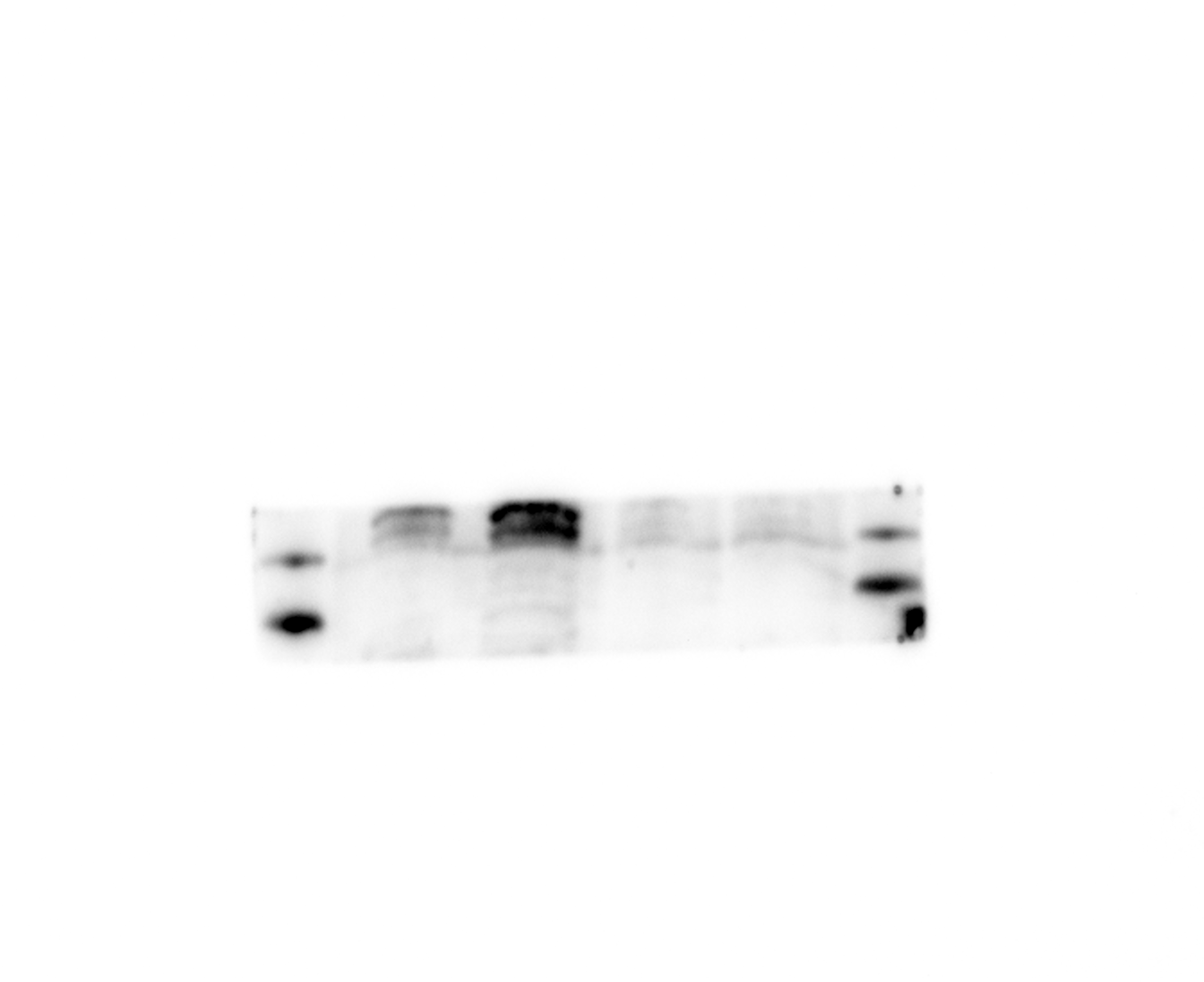

Supplement: Supplementary file 2 [file SupplementaryFile2.zip › WB数据/620-si/snail1/620-snai1-.tif]

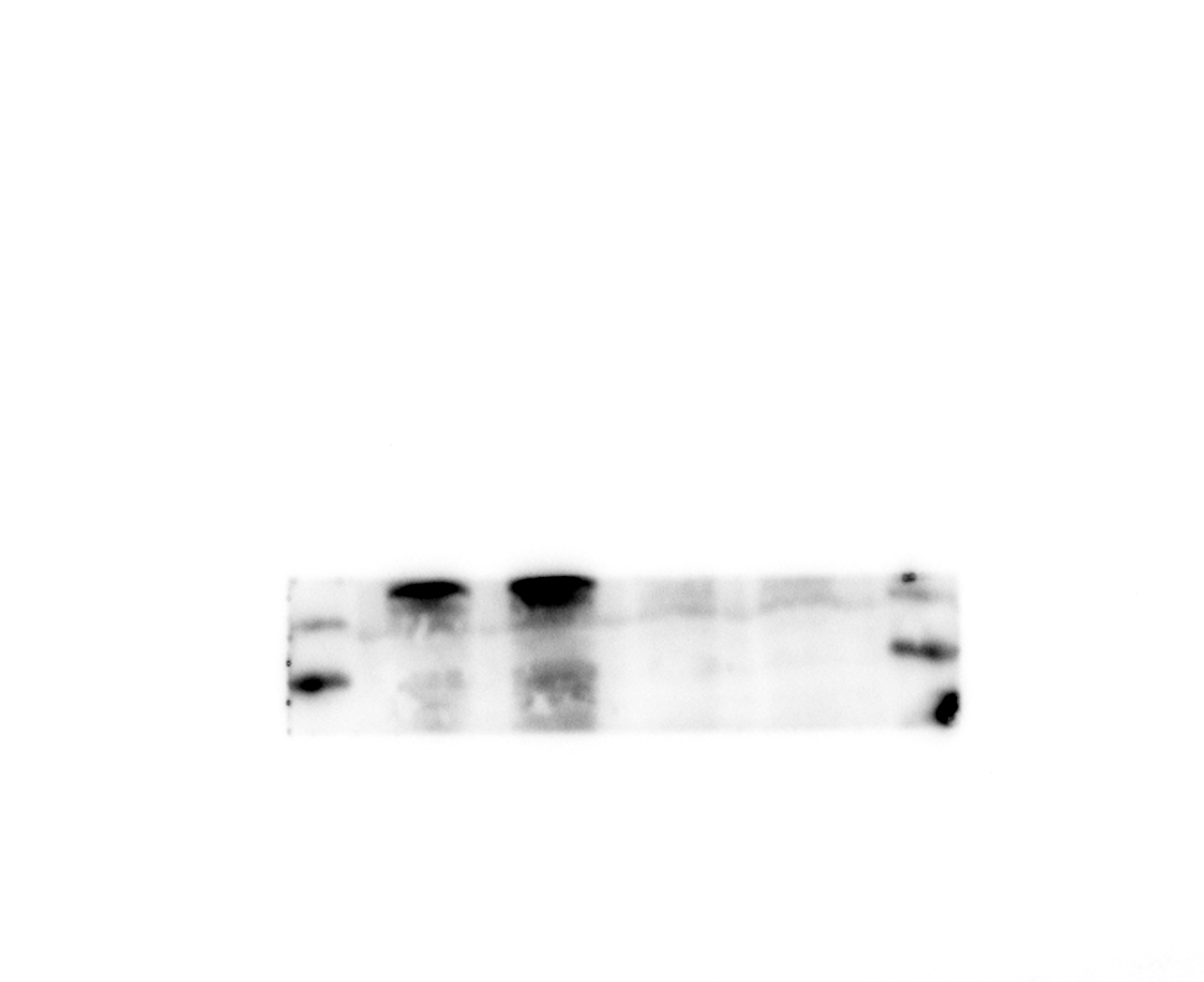

Supplement: Supplementary file 2 [file SupplementaryFile2.zip › WB数据/620-si/snail1/620-snai1.tif]

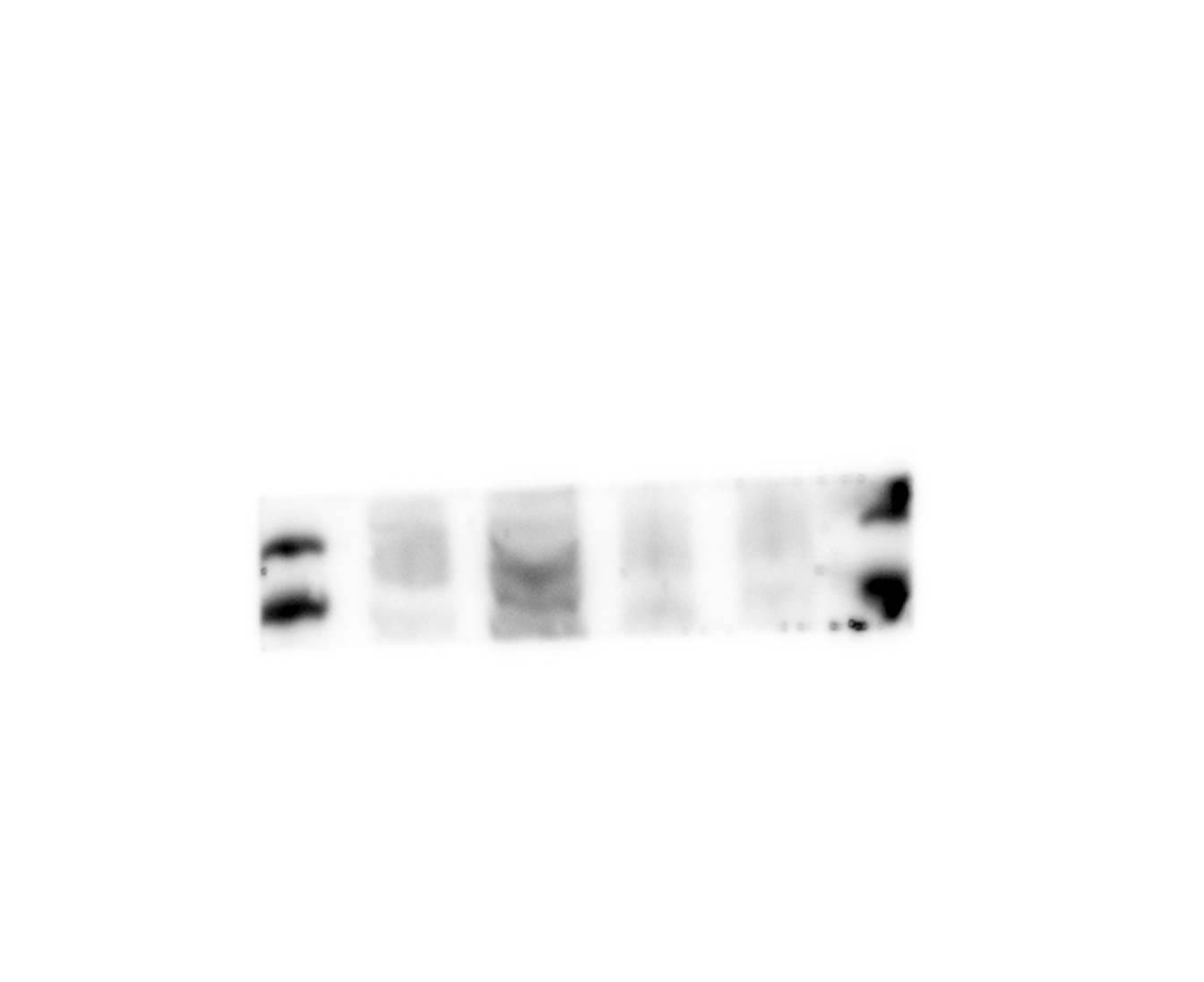

Supplement: Supplementary file 2 [file SupplementaryFile2.zip › WB数据/620-si/snail1/620-snai1`.tif]

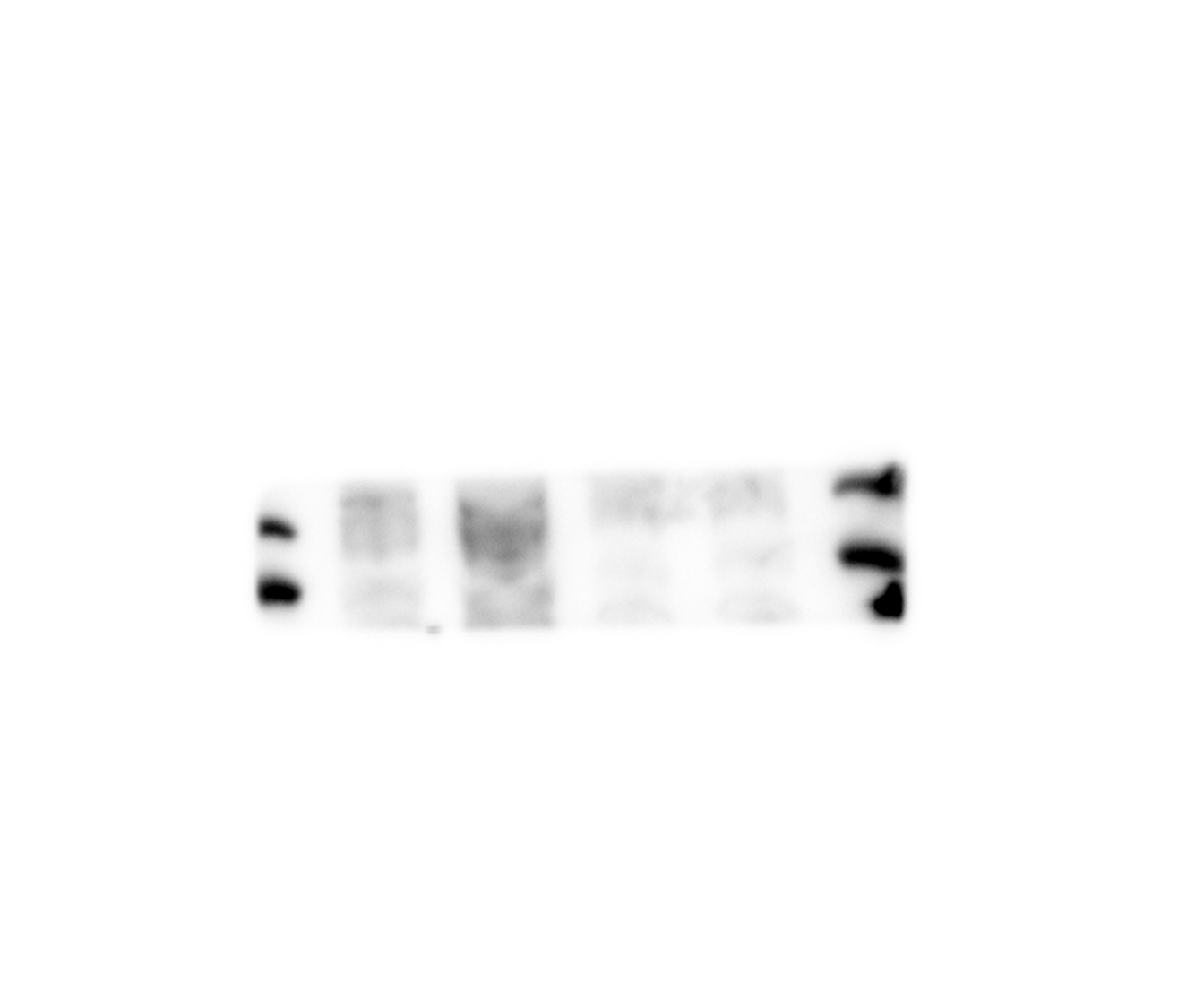

Supplement: Supplementary file 2 [file SupplementaryFile2.zip › WB数据/620-si/snail1/620-snai``.tif]

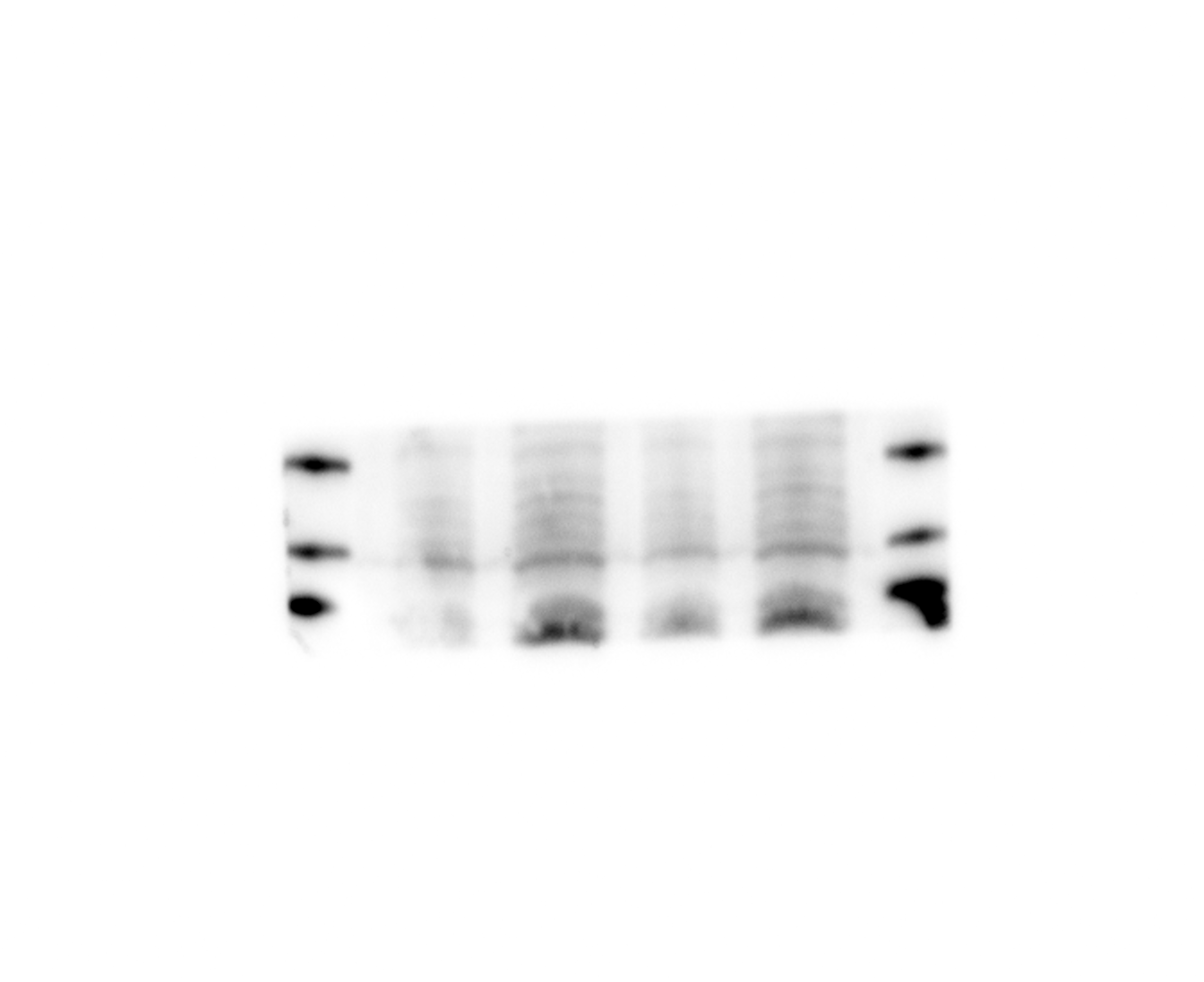

Supplement: Supplementary file 2 [file SupplementaryFile2.zip › WB数据/620-si/srebp1/620-sr1---.tif]

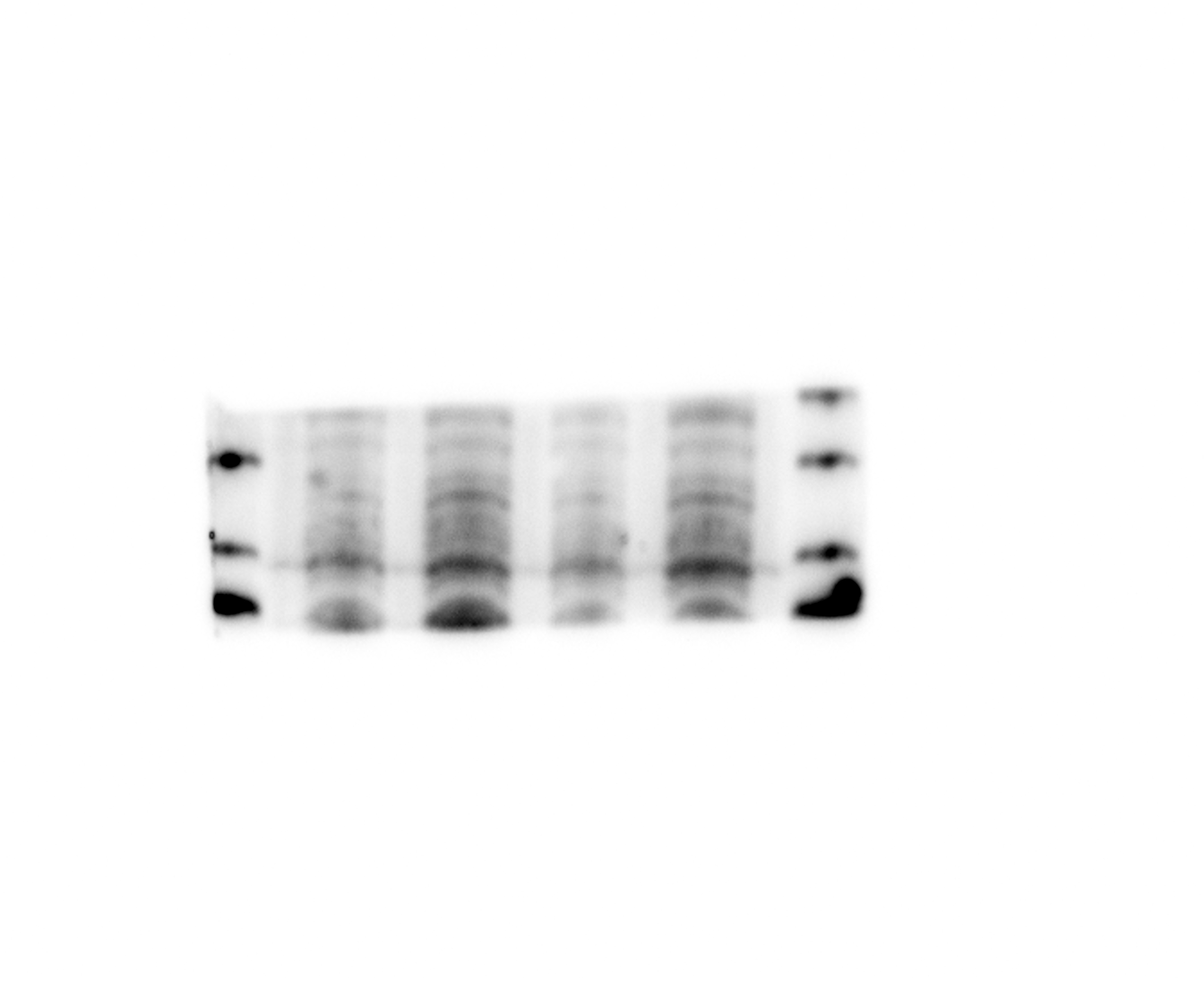

Supplement: Supplementary file 2 [file SupplementaryFile2.zip › WB数据/620-si/srebp1/620-sr1-.tif]

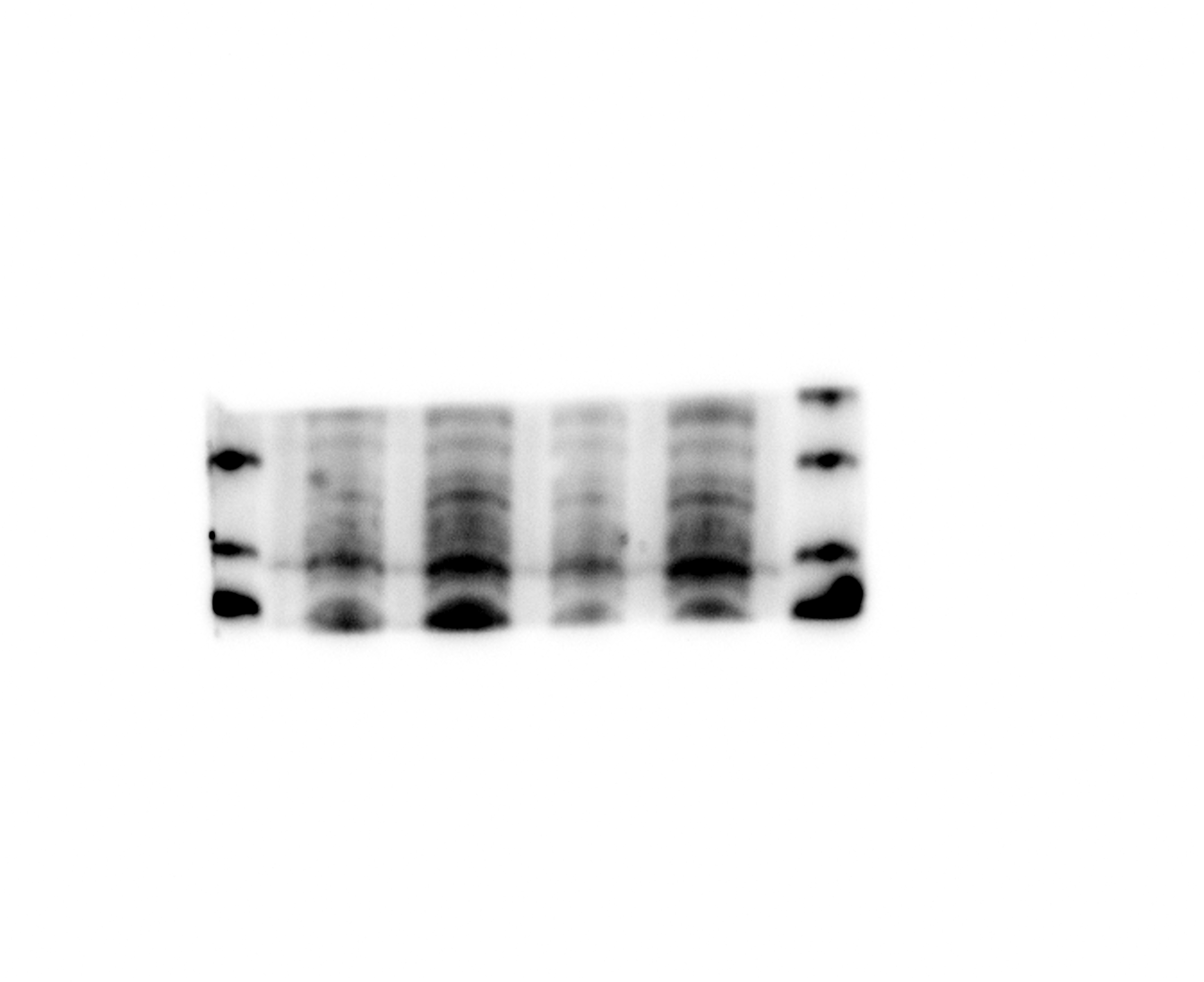

Supplement: Supplementary file 2 [file SupplementaryFile2.zip › WB数据/620-si/srebp1/620-sr1.tif]

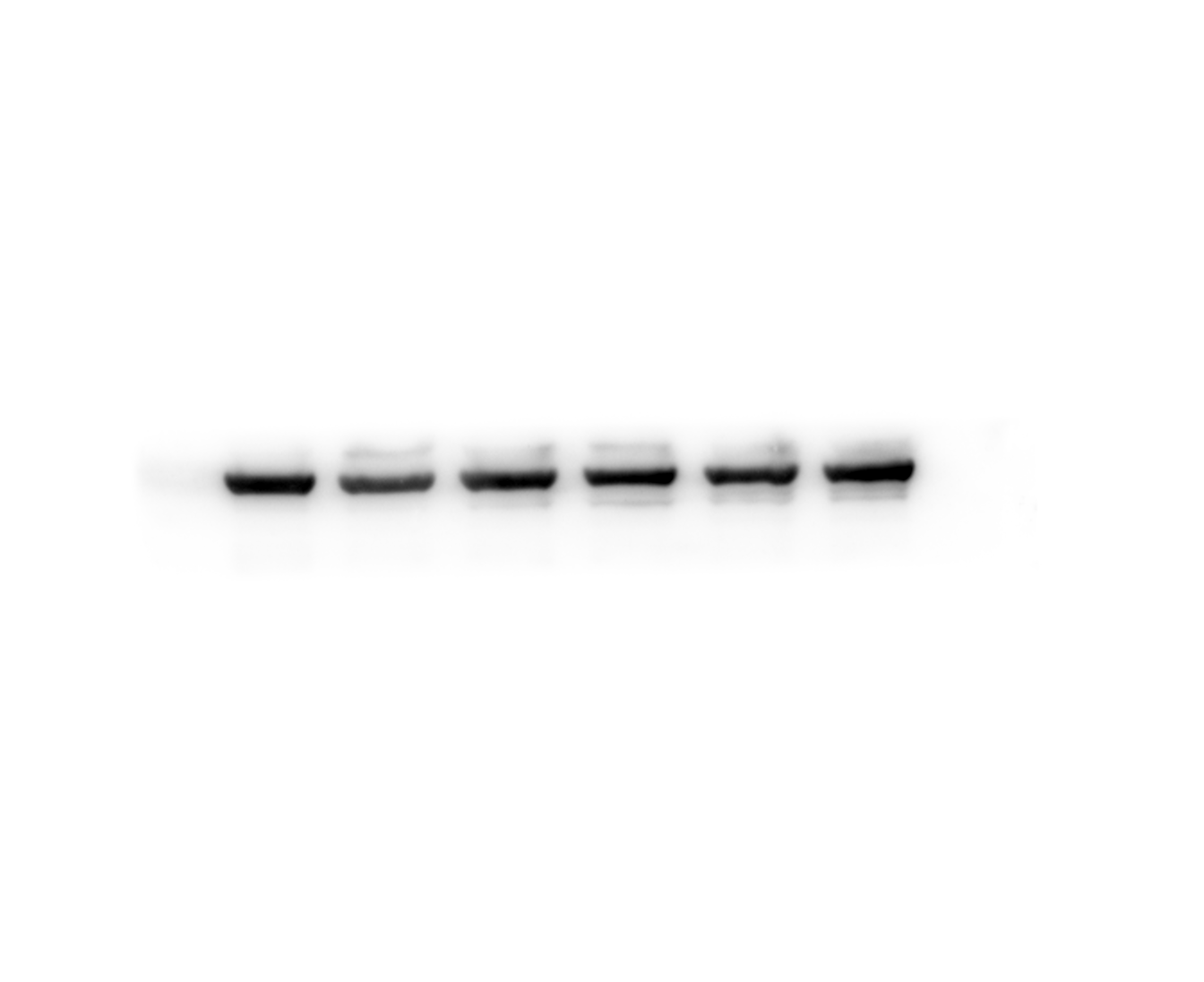

Supplement: Supplementary file 2 [file SupplementaryFile2.zip › WB数据/动物/actin/-actin.tif]

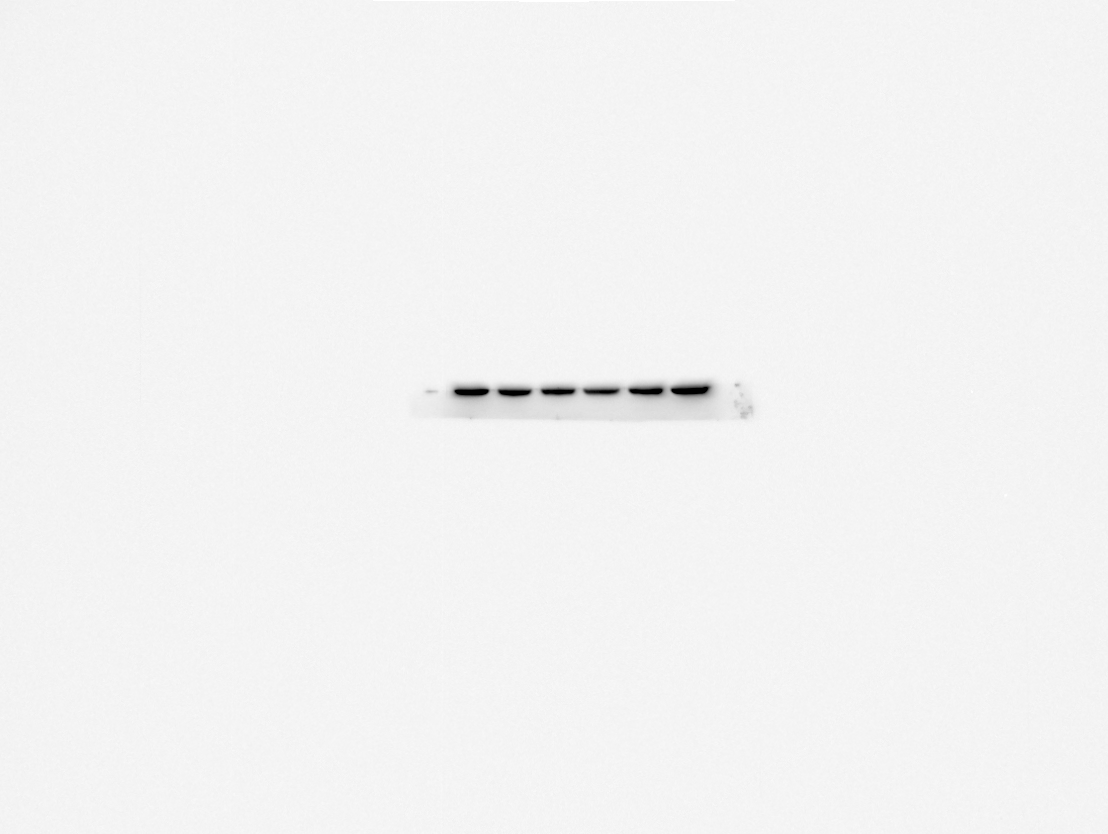

Supplement: Supplementary file 2 [file SupplementaryFile2.zip › WB数据/动物/actin/actin-.jpg]

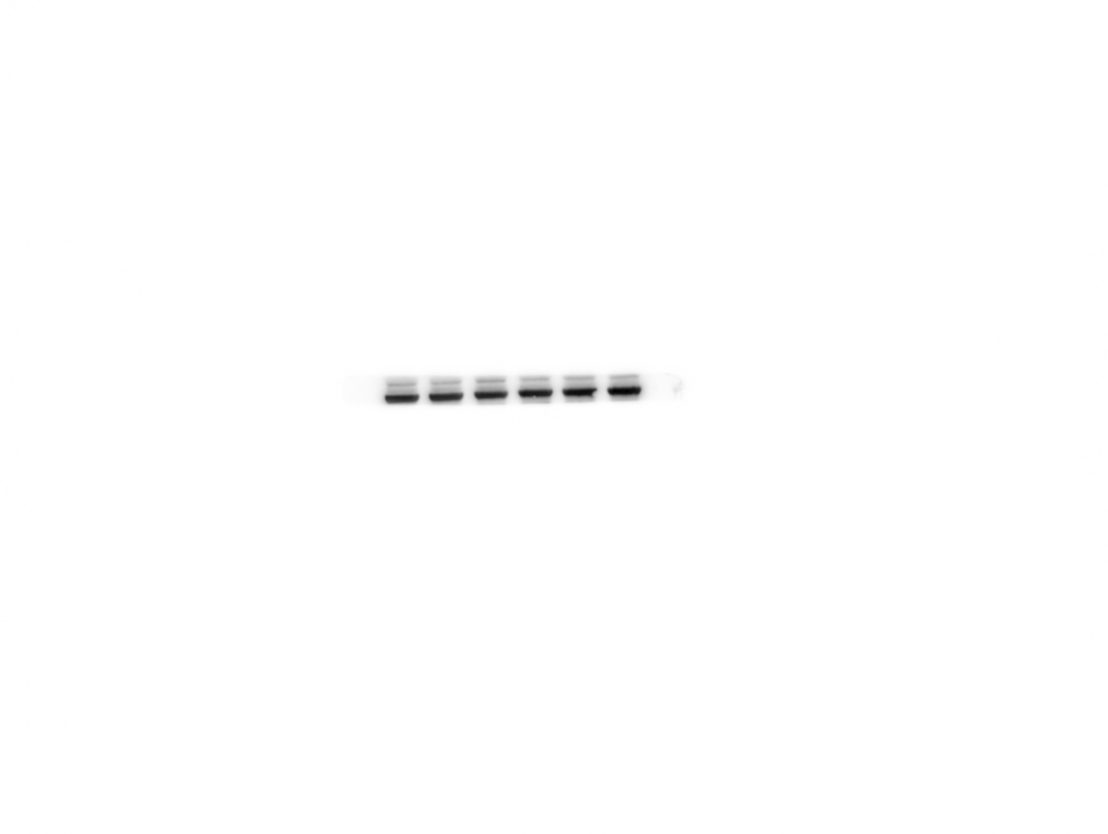

Supplement: Supplementary file 2 [file SupplementaryFile2.zip › WB数据/动物/actin/actin.jpg]

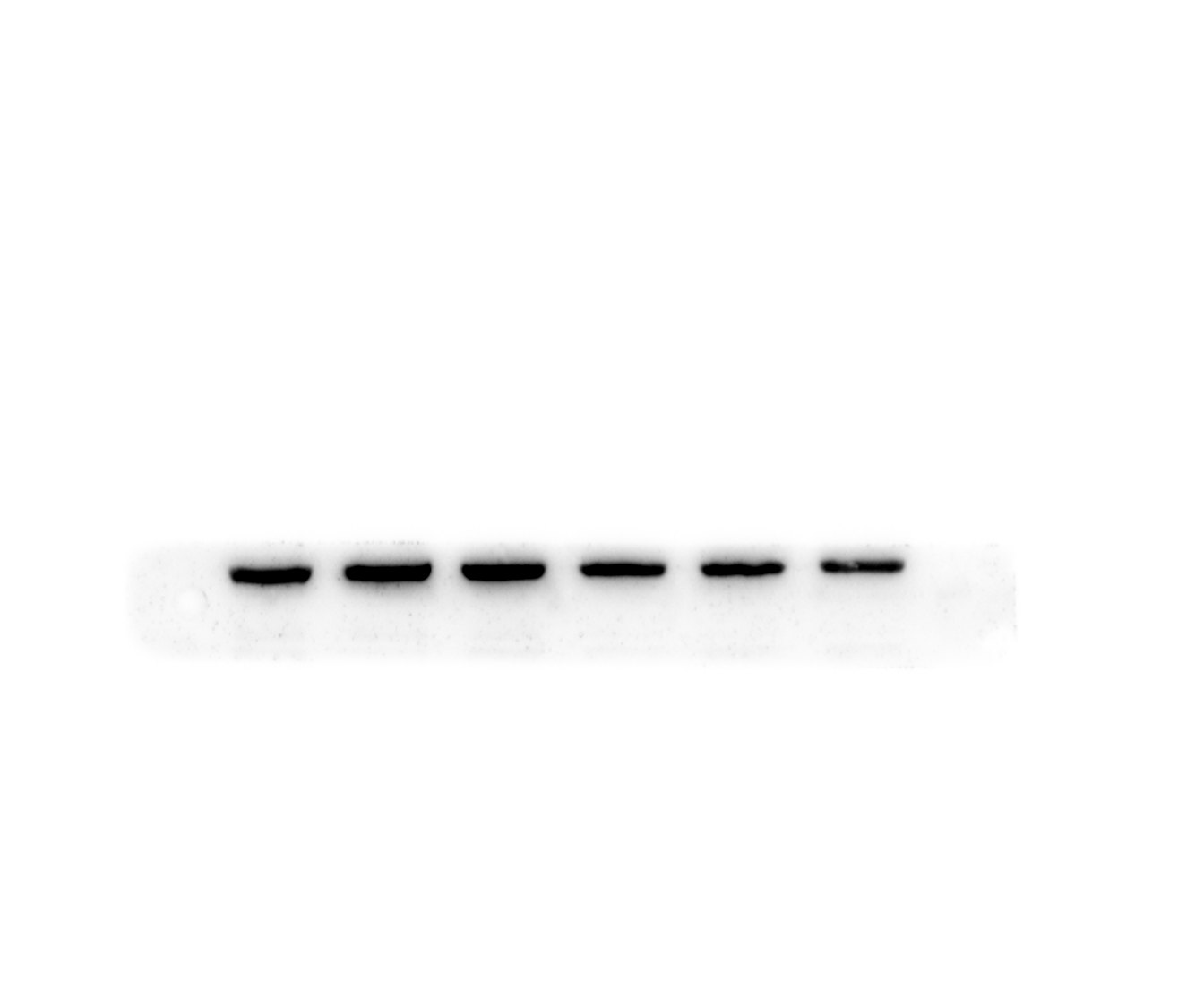

Supplement: Supplementary file 2 [file SupplementaryFile2.zip › WB数据/动物/actin/组织actin.tif]

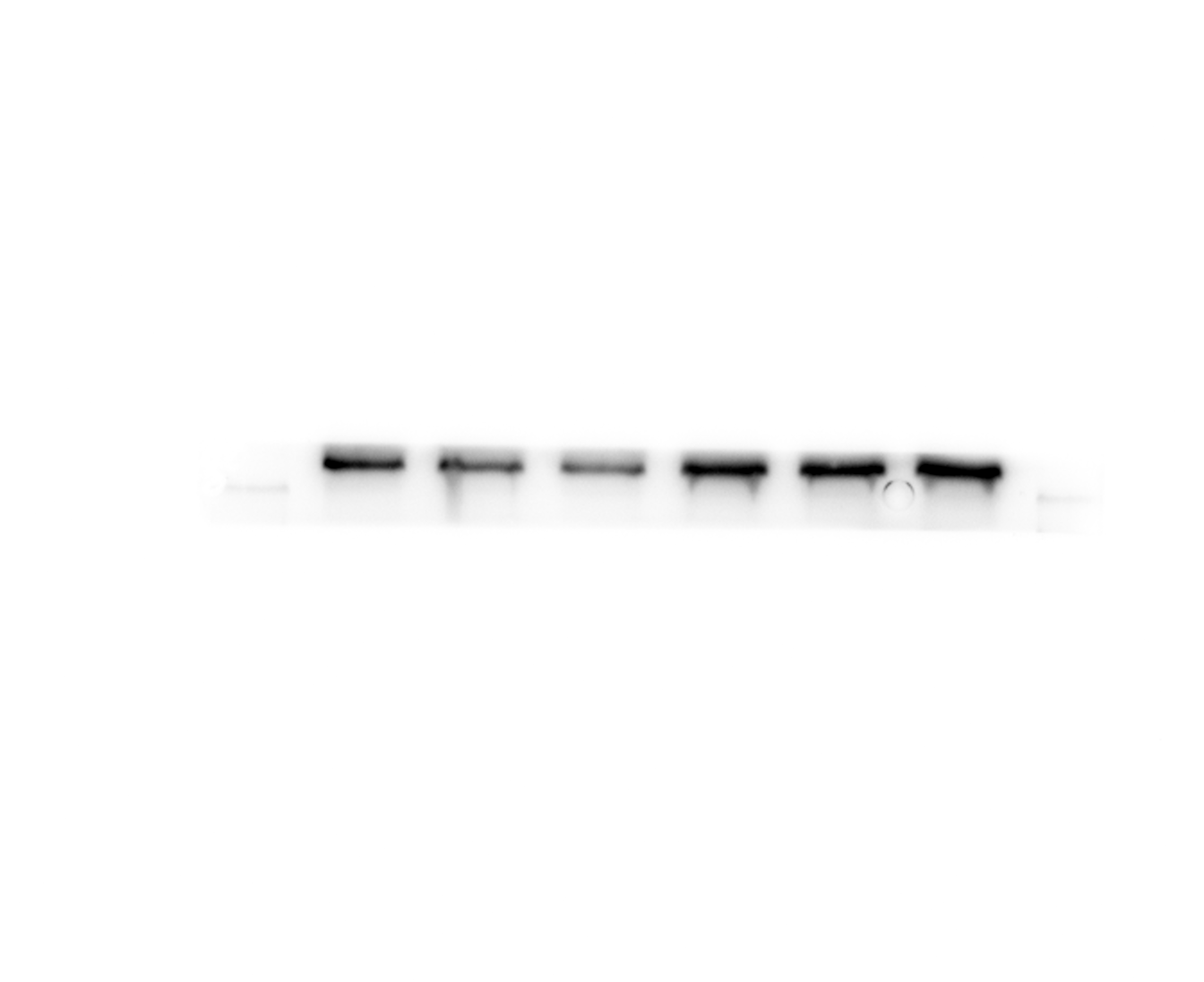

Supplement: Supplementary file 2 [file SupplementaryFile2.zip › WB数据/动物/e/e.tif]

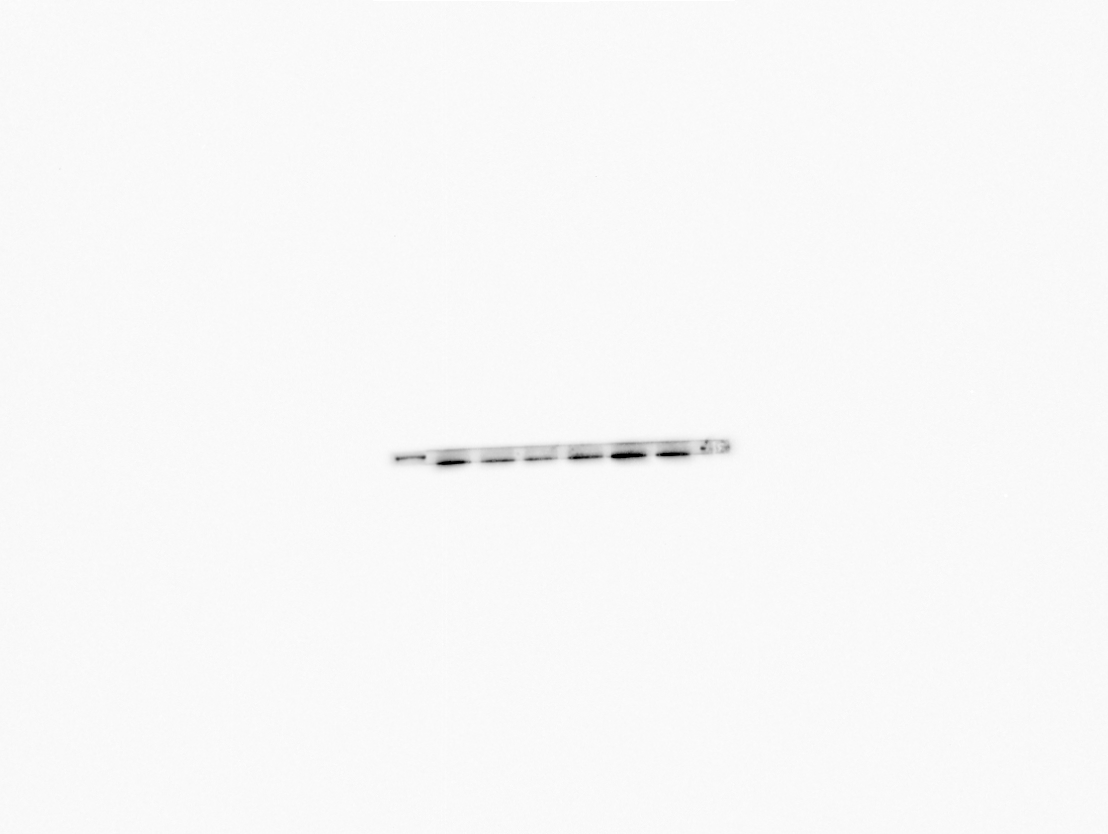

Supplement: Supplementary file 2 [file SupplementaryFile2.zip › WB数据/动物/e/E钙粘蛋白-.jpg]

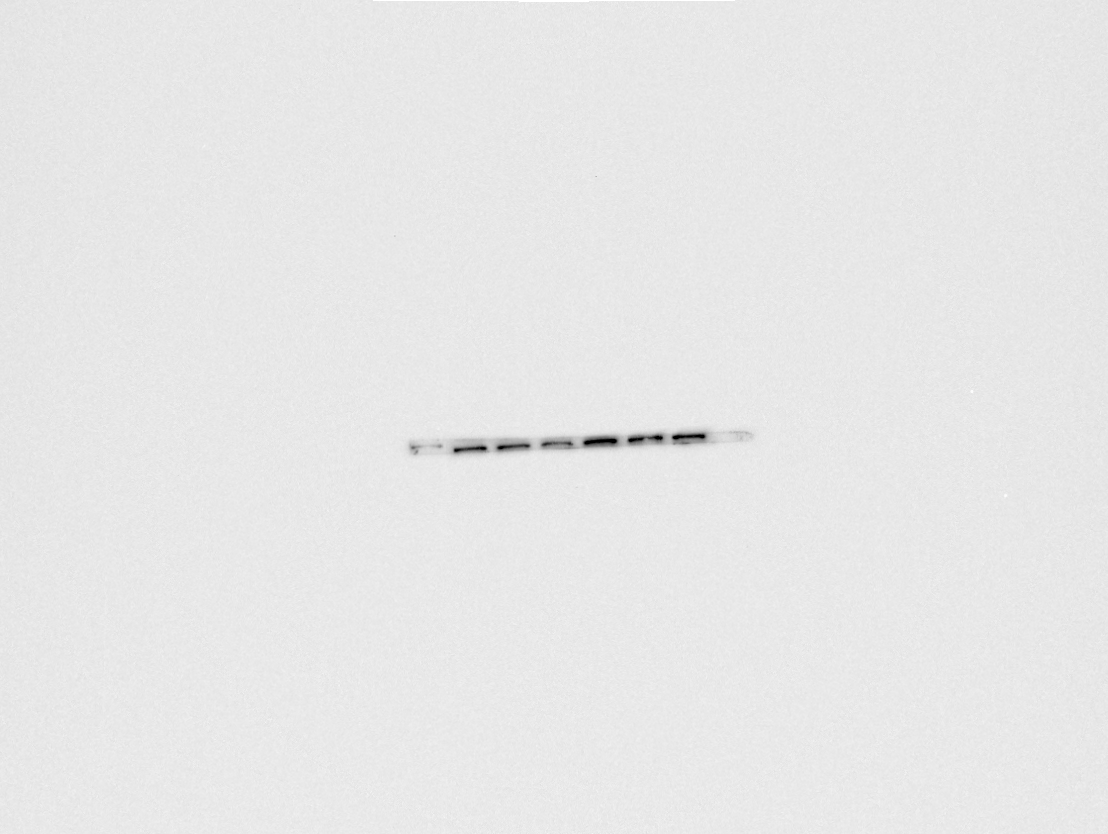

Supplement: Supplementary file 2 [file SupplementaryFile2.zip › WB数据/动物/e/E钙粘蛋白.jpg]

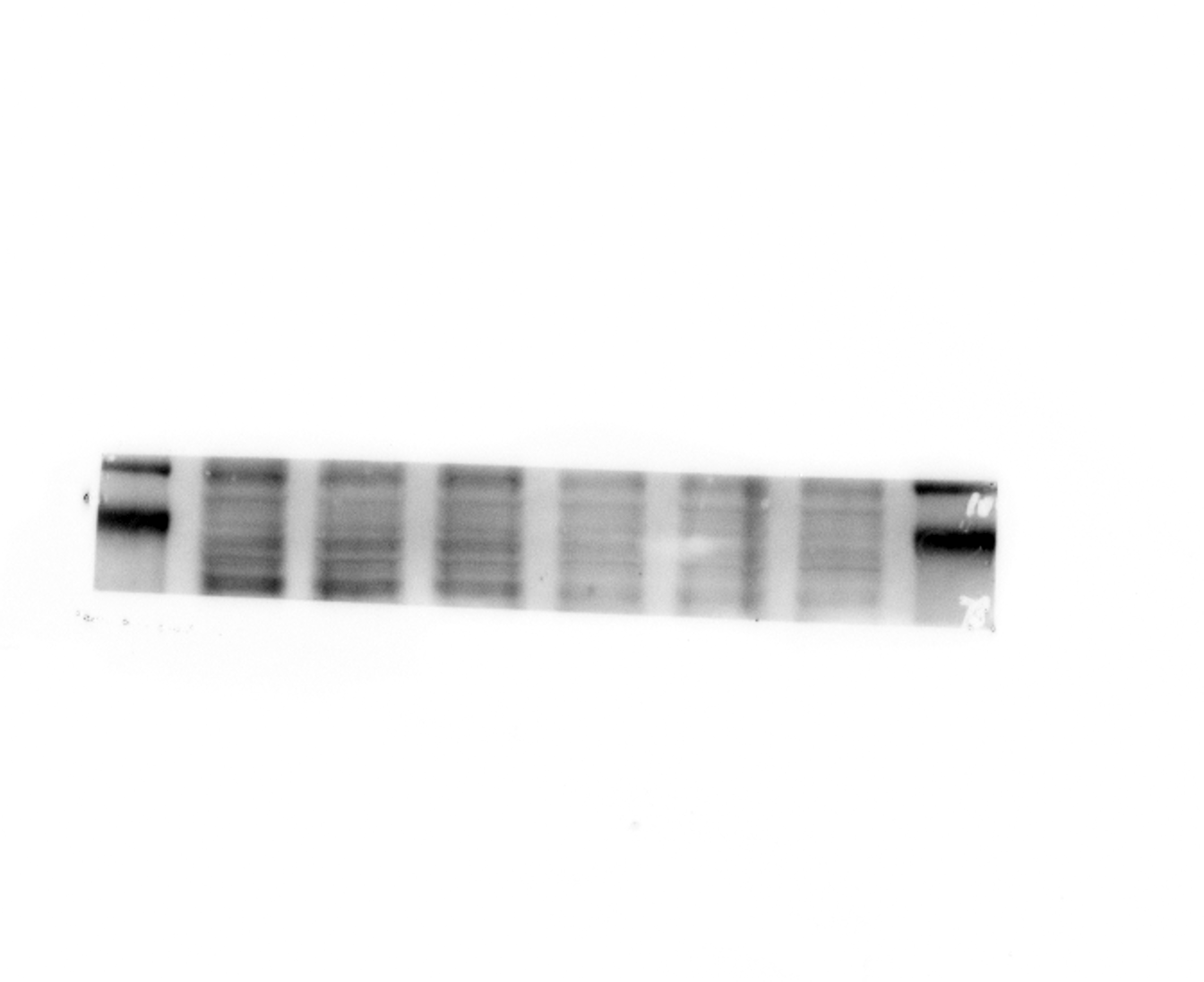

Supplement: Supplementary file 2 [file SupplementaryFile2.zip › WB数据/动物/mmp9/mmp9--.tif]

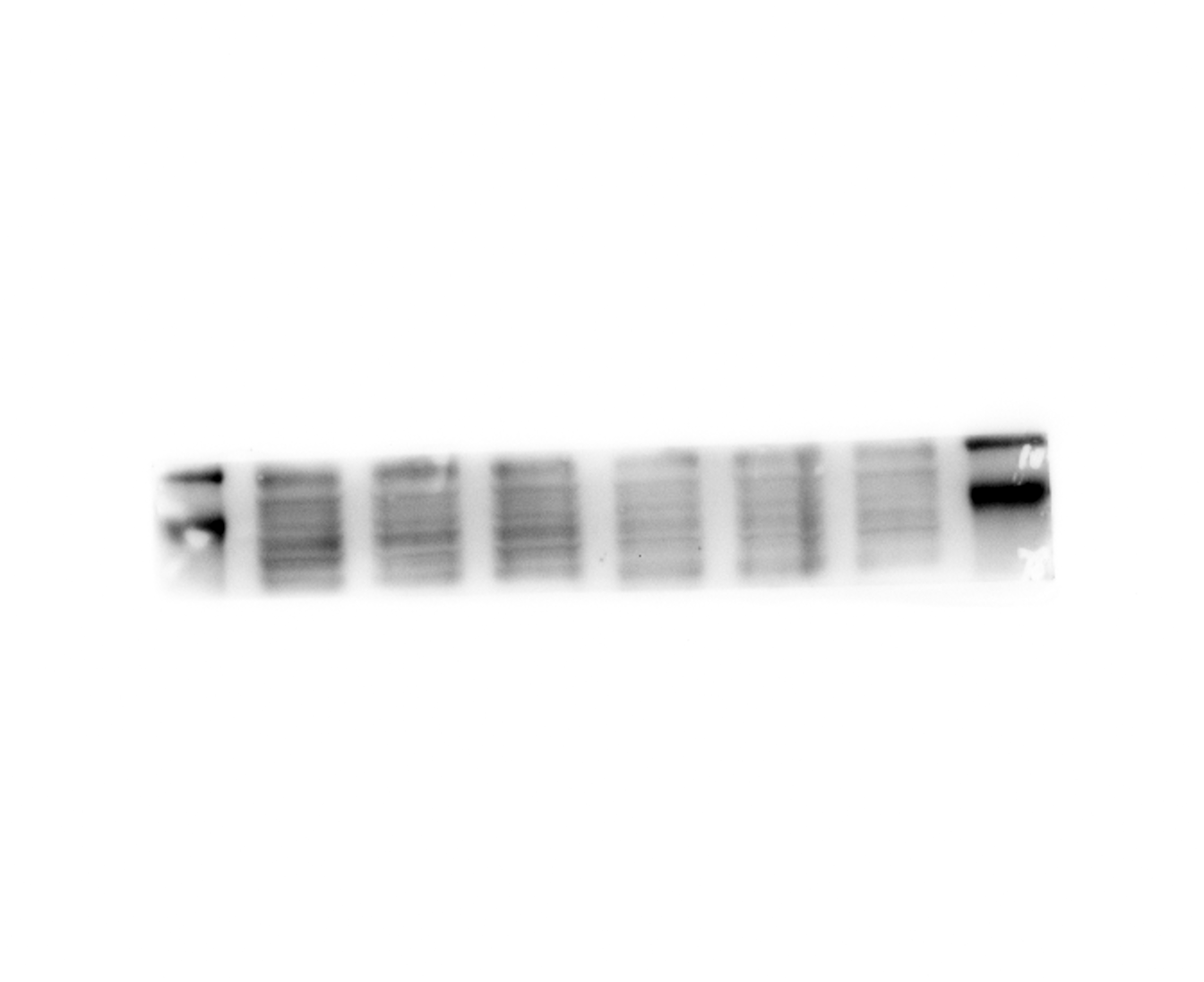

Supplement: Supplementary file 2 [file SupplementaryFile2.zip › WB数据/动物/mmp9/mmp9-.tif]

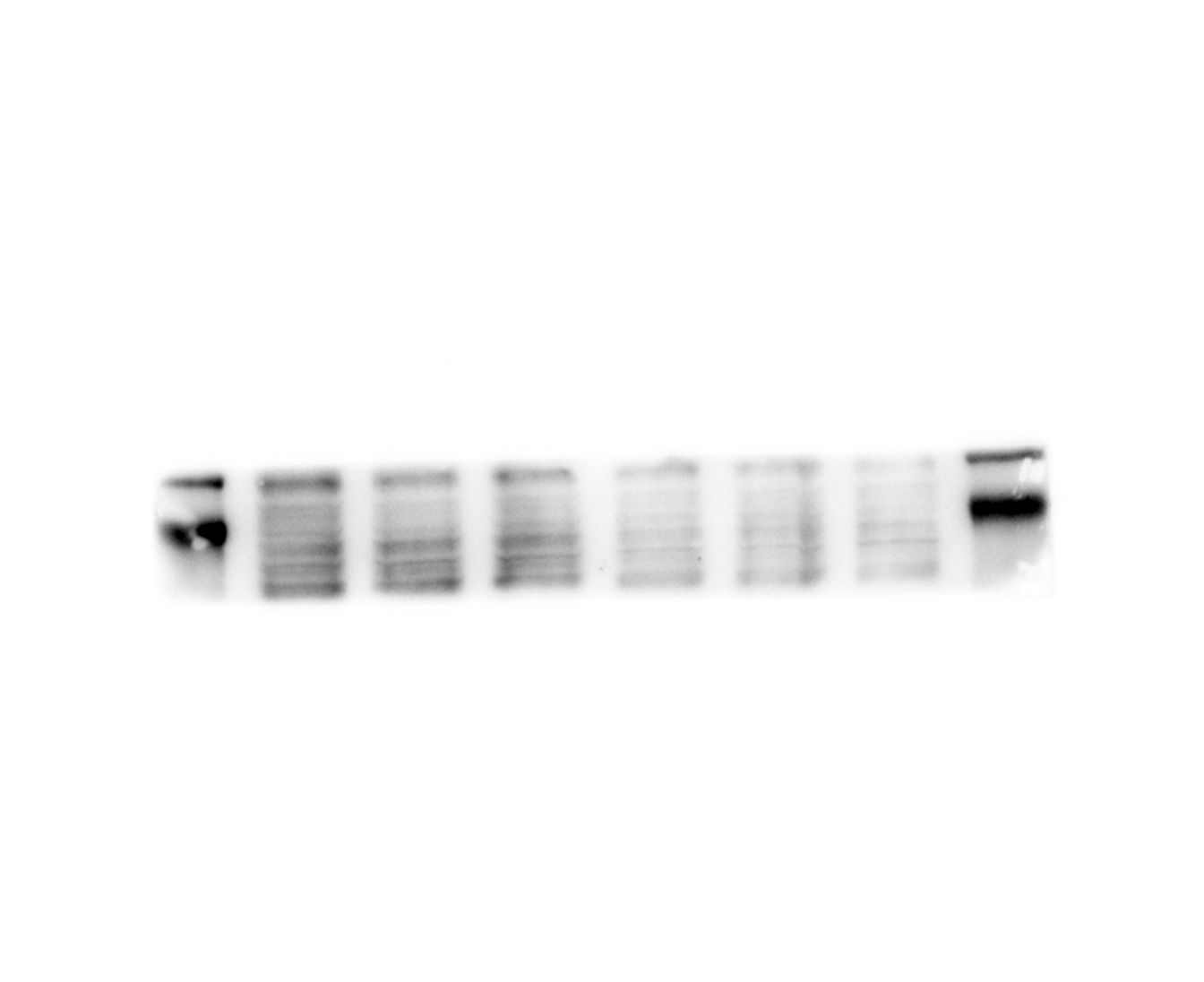

Supplement: Supplementary file 2 [file SupplementaryFile2.zip › WB数据/动物/mmp9/mmp9.tif]

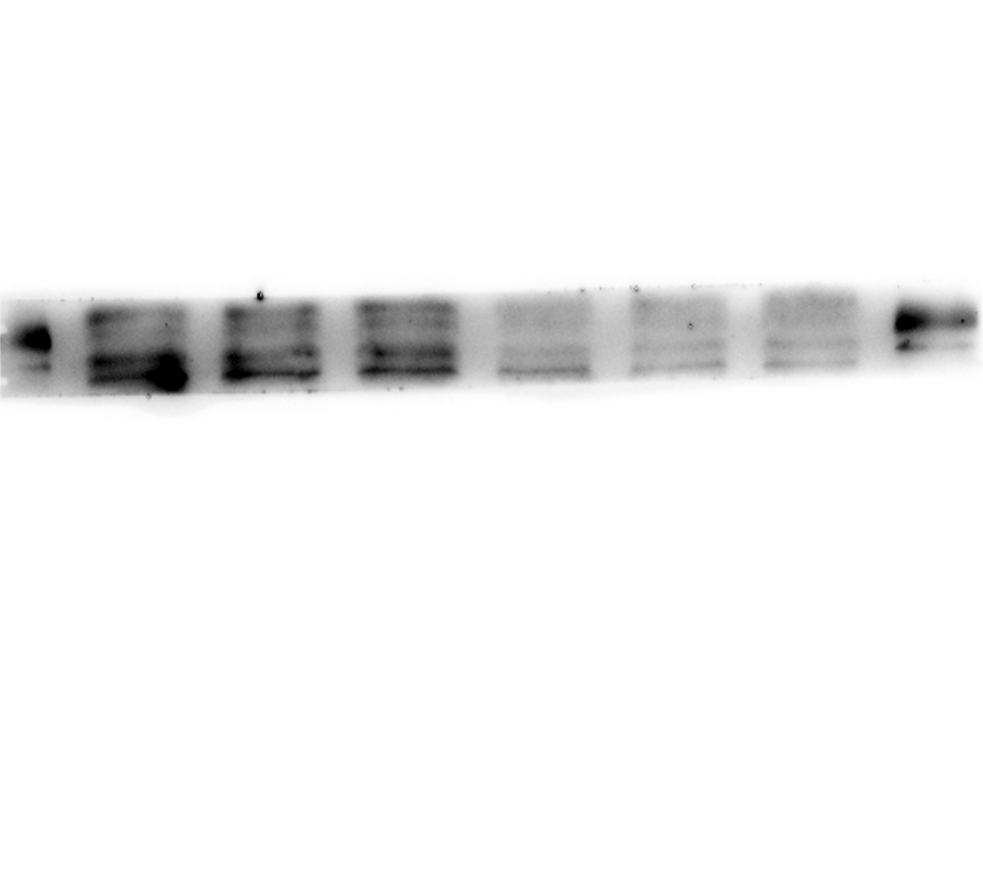

Supplement: Supplementary file 2 [file SupplementaryFile2.zip › WB数据/动物/mmp9/t-mmp9.png]

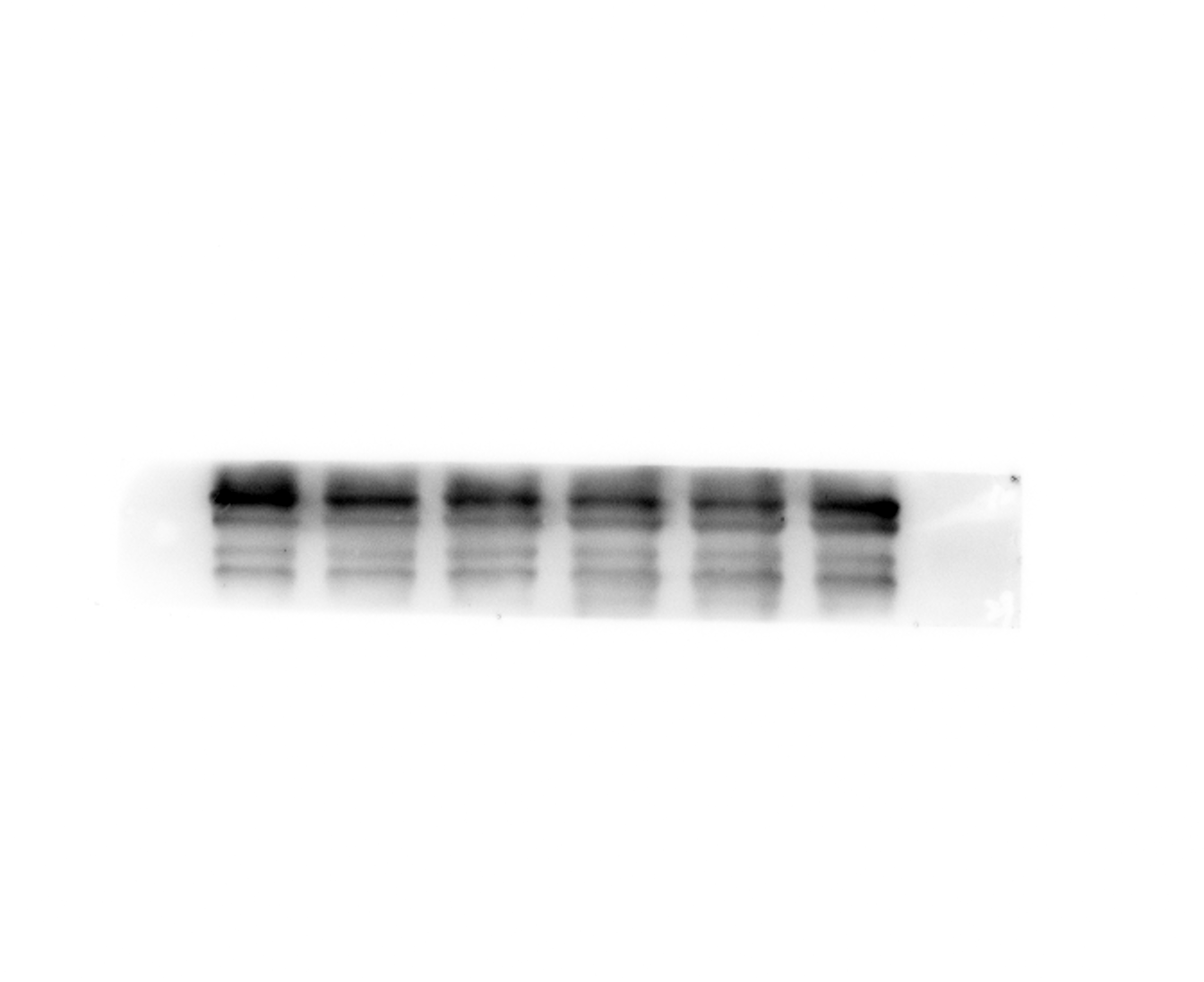

Supplement: Supplementary file 2 [file SupplementaryFile2.zip › WB数据/动物/snail/snail--.tif]

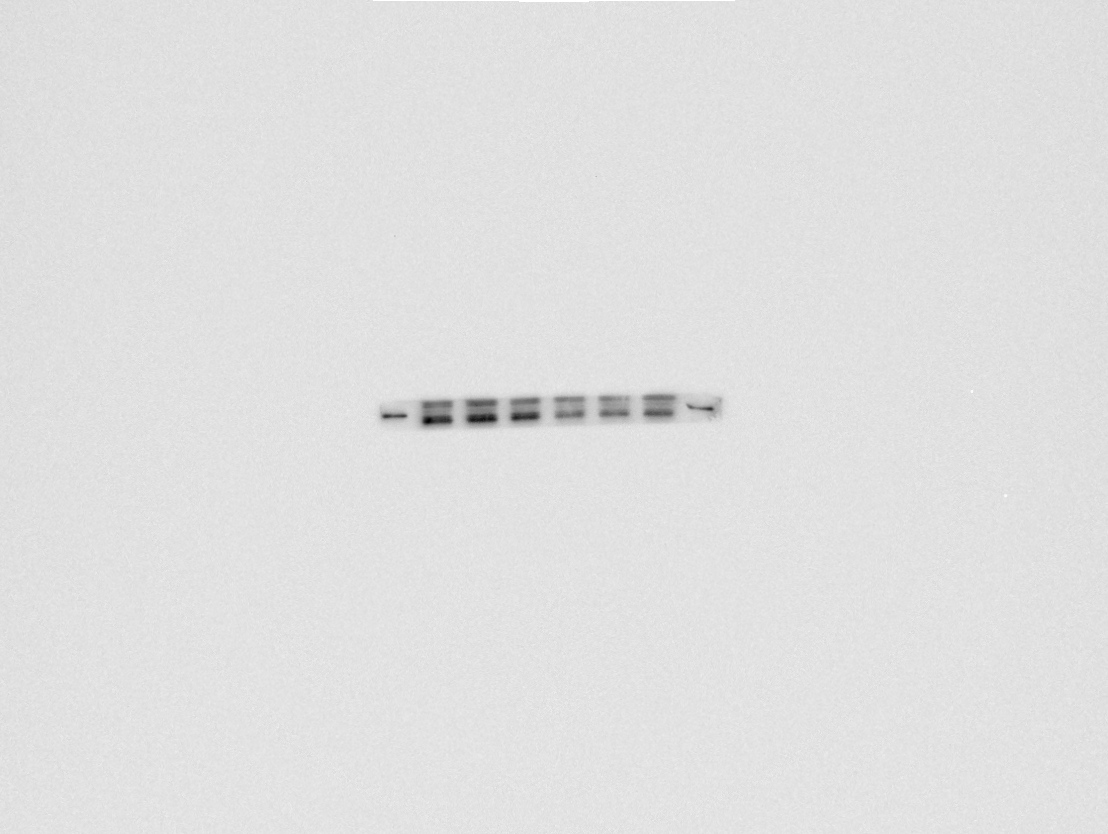

Supplement: Supplementary file 2 [file SupplementaryFile2.zip › WB数据/动物/snail/snail-.jpg]

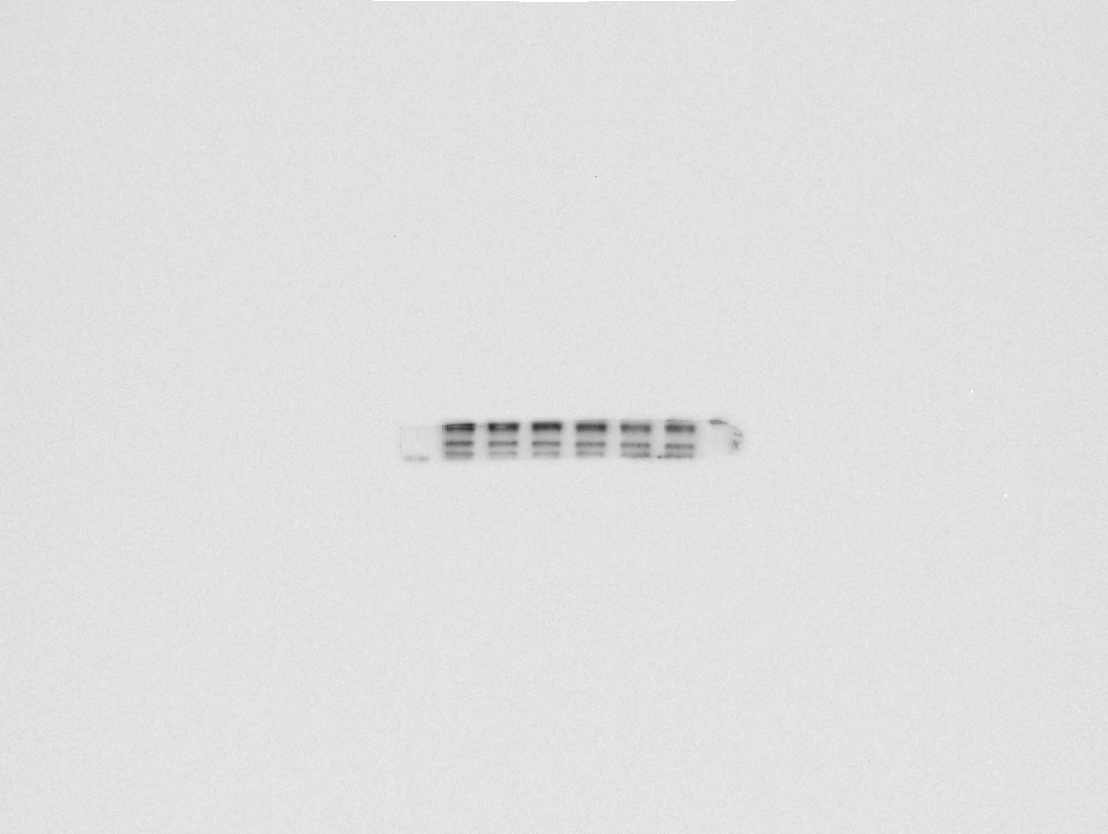

Supplement: Supplementary file 2 [file SupplementaryFile2.zip › WB数据/动物/snail/snail.jpg]

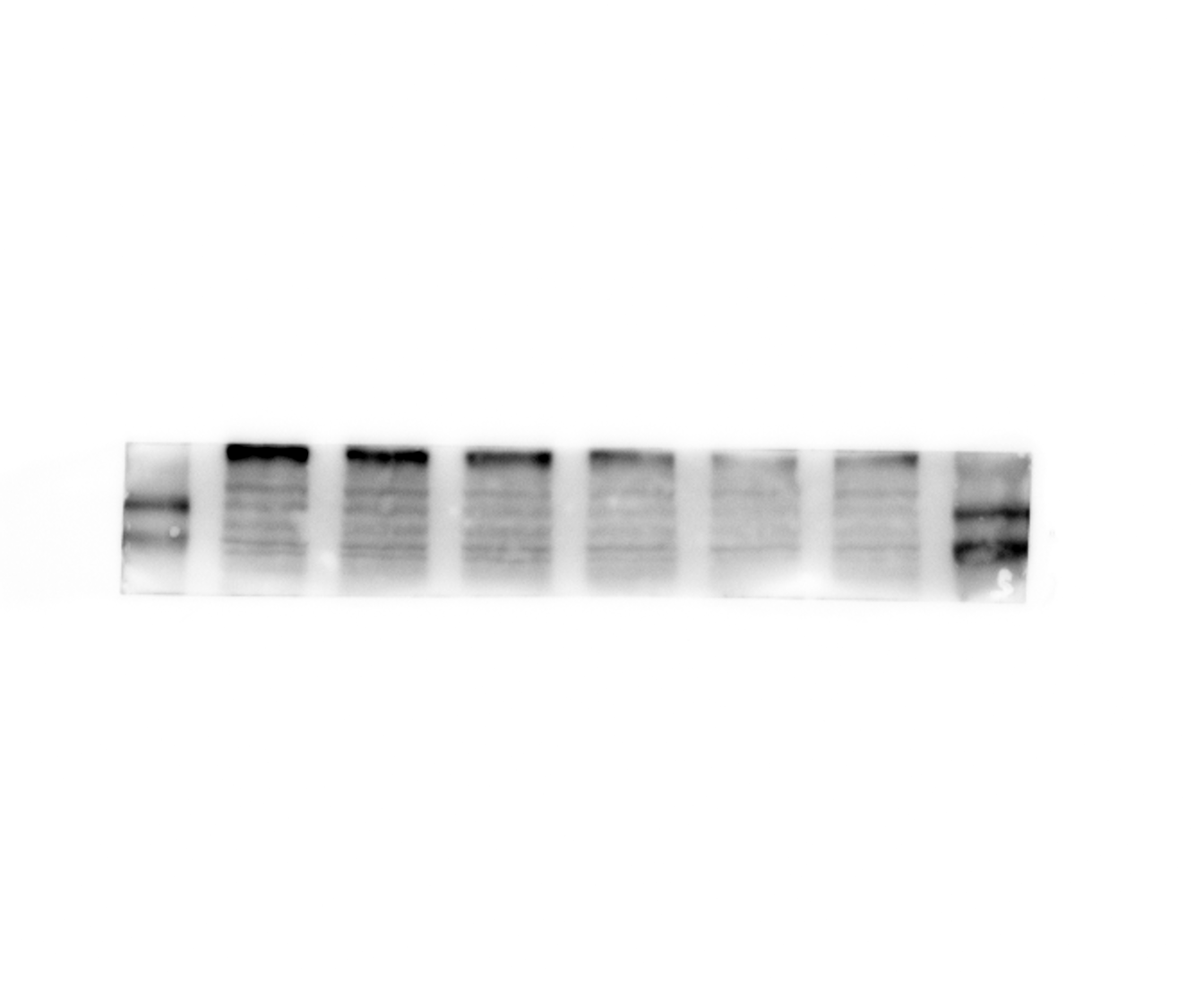

Supplement: Supplementary file 2 [file SupplementaryFile2.zip › WB数据/动物/srebp1/srebp1--.tif]

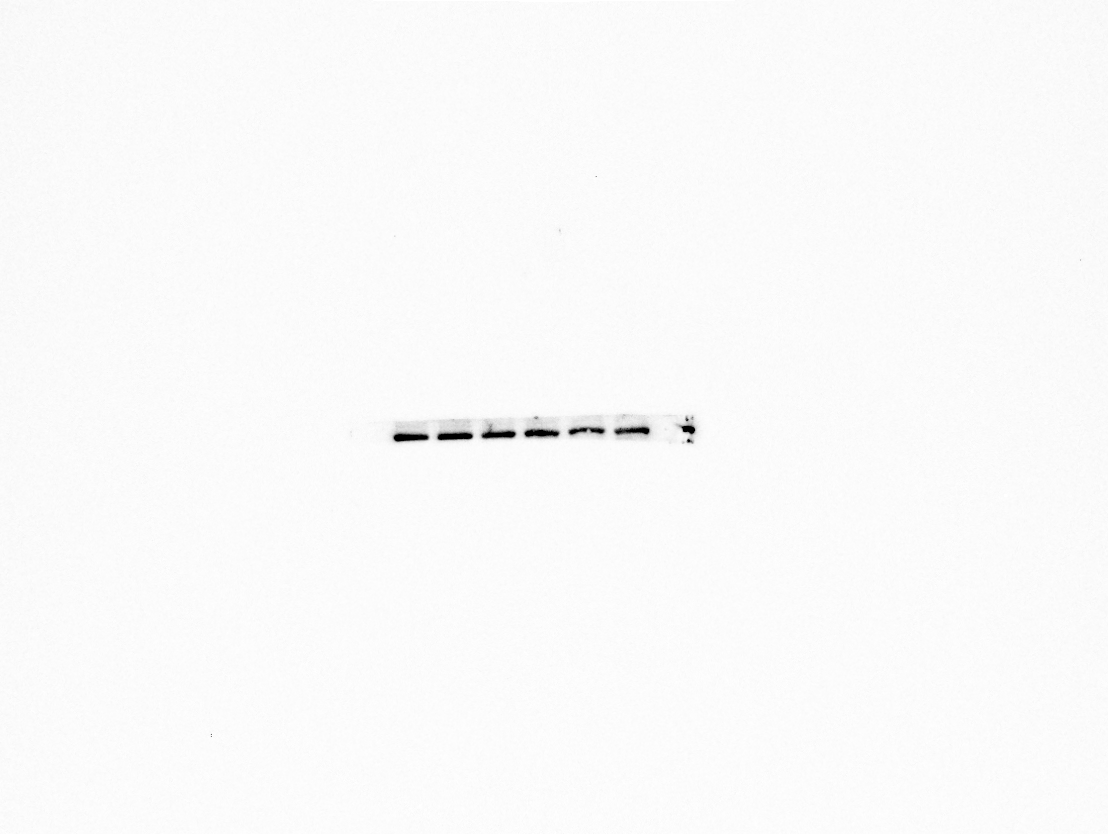

Supplement: Supplementary file 2 [file SupplementaryFile2.zip › WB数据/动物/srebp1/srebp1-68.jpg]

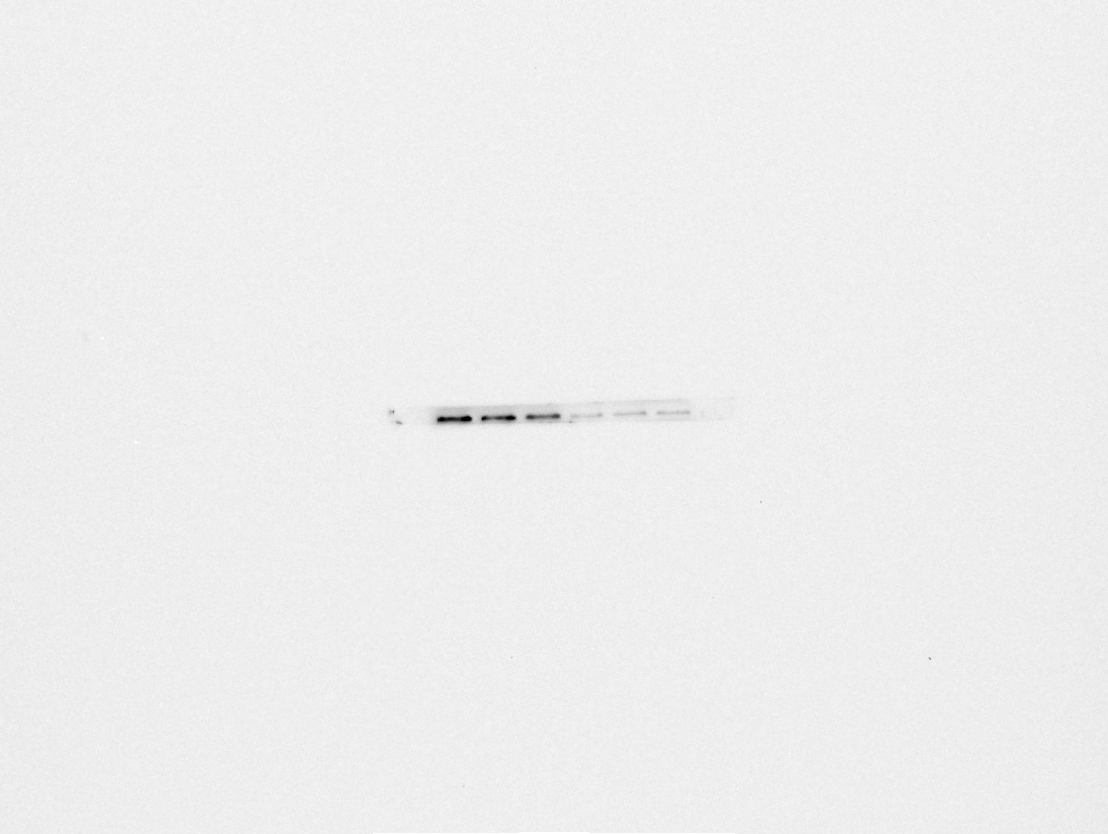

Supplement: Supplementary file 2 [file SupplementaryFile2.zip › WB数据/动物/srebp1/srebp1.jpg]

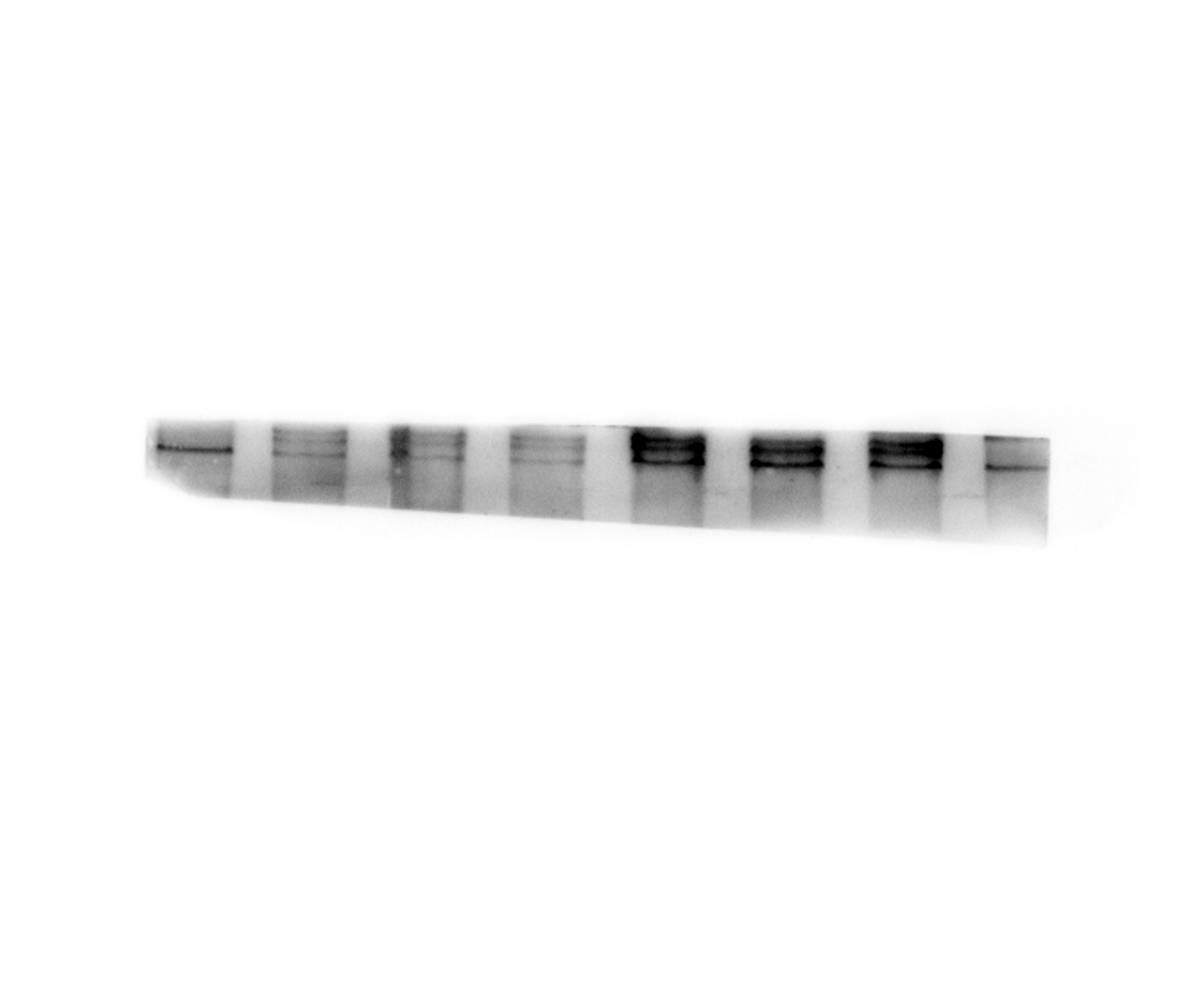

Supplement: Supplementary file 2 [file SupplementaryFile2.zip › WB数据/动物/zo1/zo1----.tif]

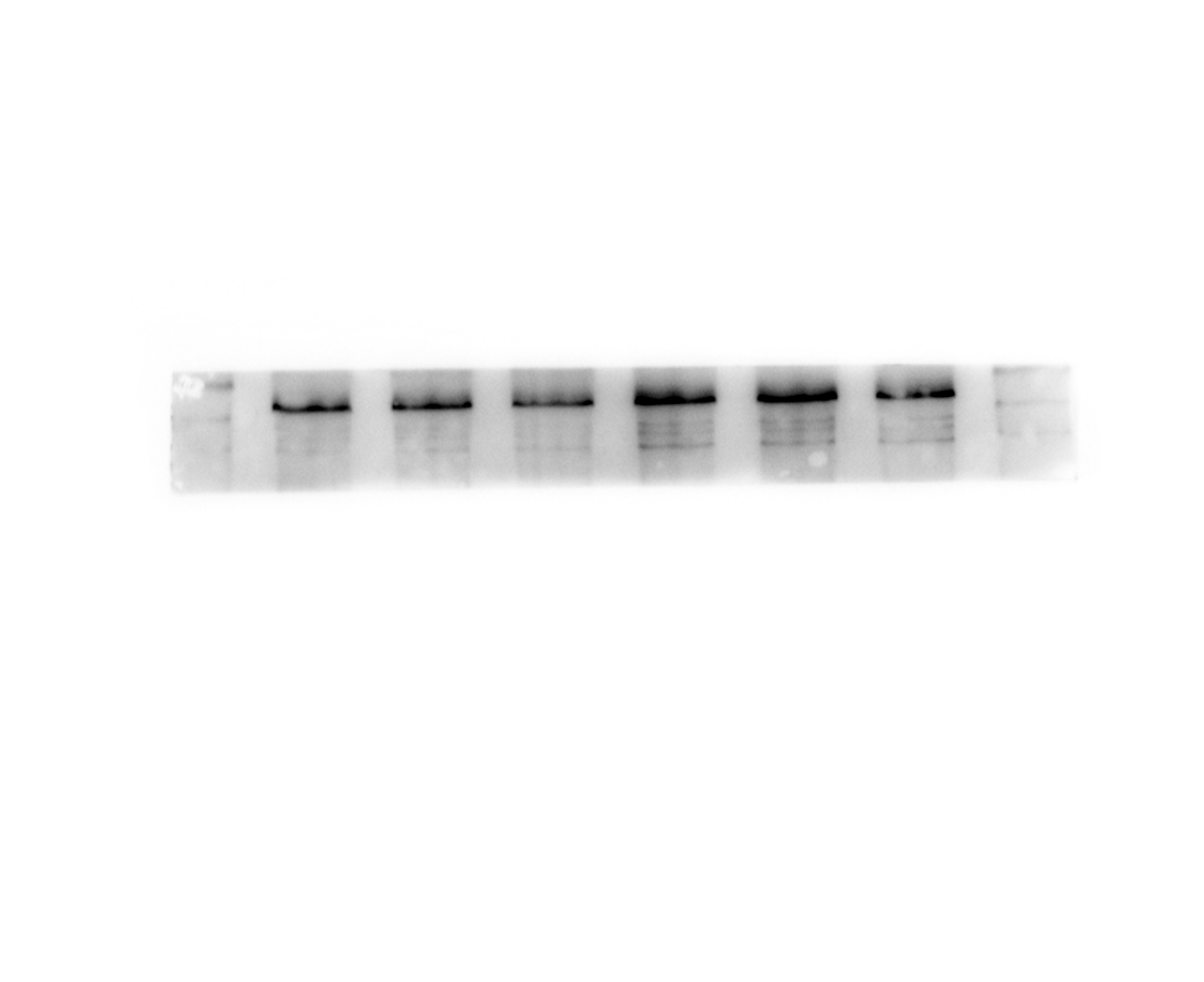

Supplement: Supplementary file 2 [file SupplementaryFile2.zip › WB数据/动物/zo1/zo1--.tif]

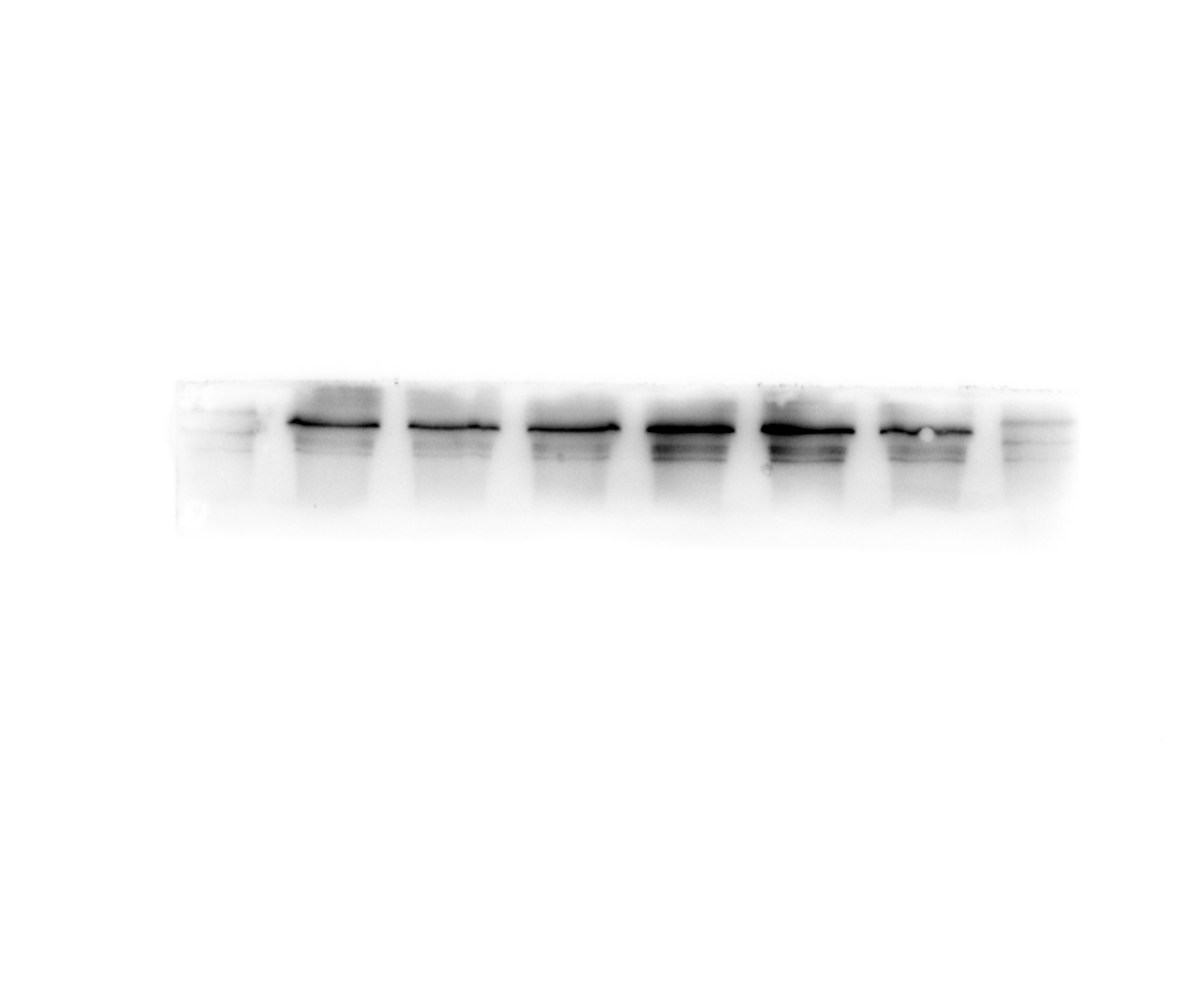

Supplement: Supplementary file 2 [file SupplementaryFile2.zip › WB数据/动物/zo1/zo1.tif]

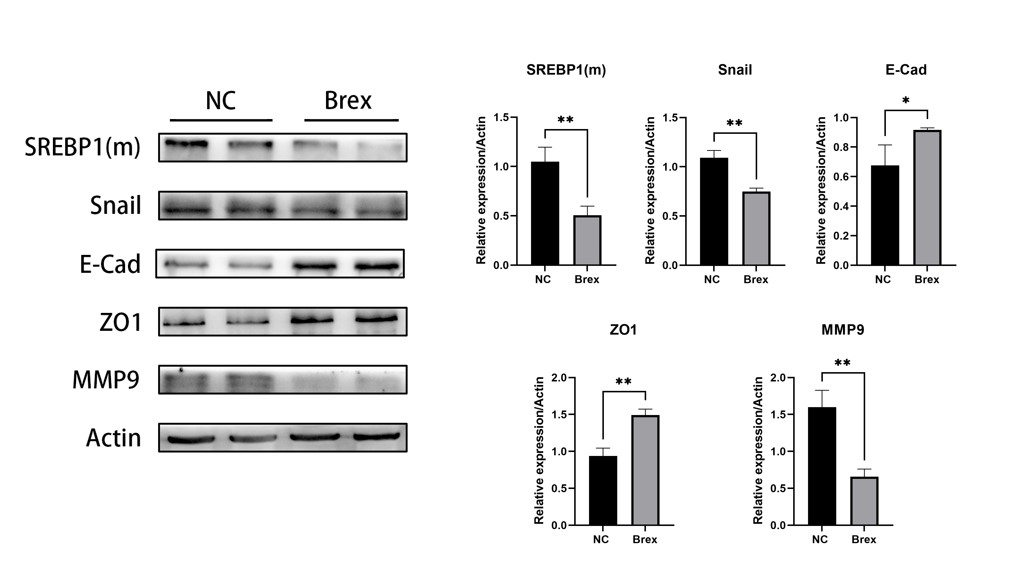

Supplement: Supplementary file 2 [file SupplementaryFile2.zip › WB数据/动物/数据分析/1.jpg]

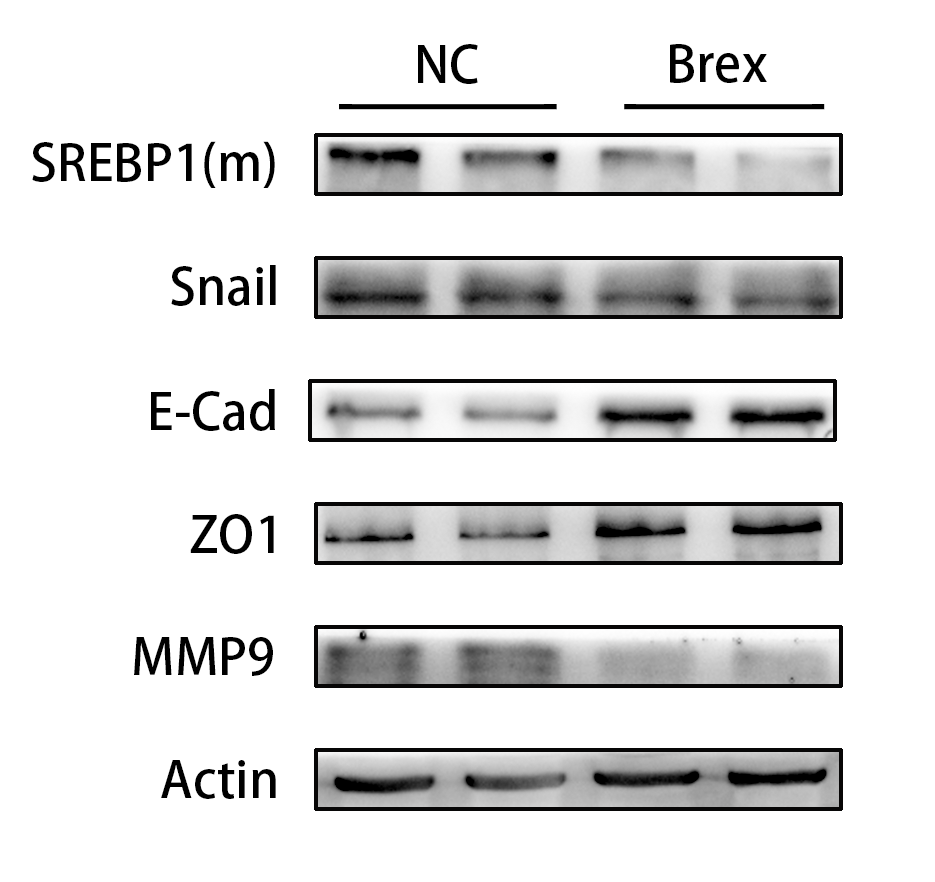

Supplement: Supplementary file 2 [file SupplementaryFile2.zip › WB数据/动物/数据分析/1.png]

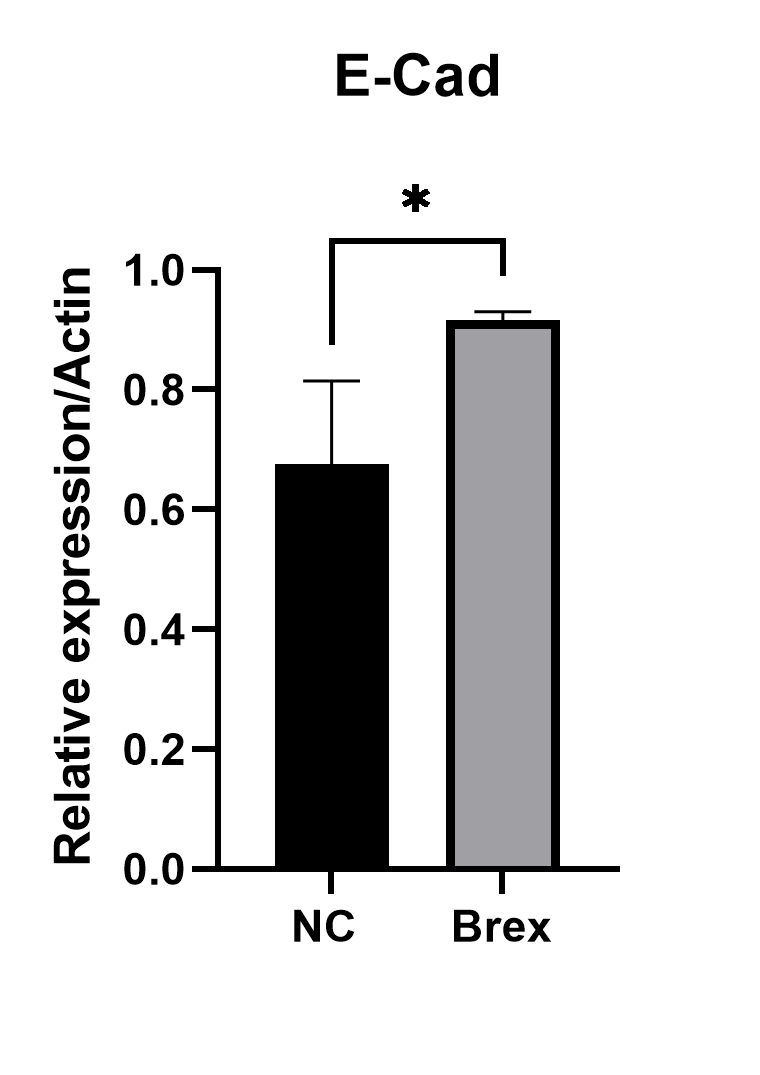

Supplement: Supplementary file 2 [file SupplementaryFile2.zip › WB数据/动物/数据分析/E.tif]

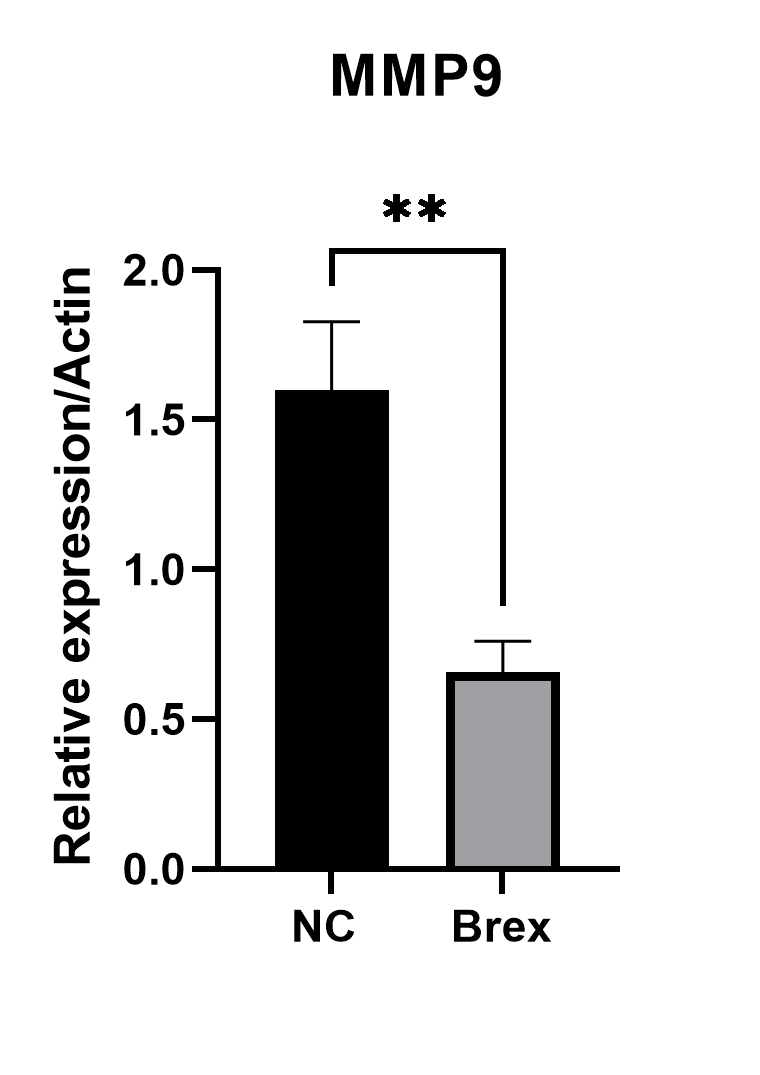

Supplement: Supplementary file 2 [file SupplementaryFile2.zip › WB数据/动物/数据分析/MMP9.tif]

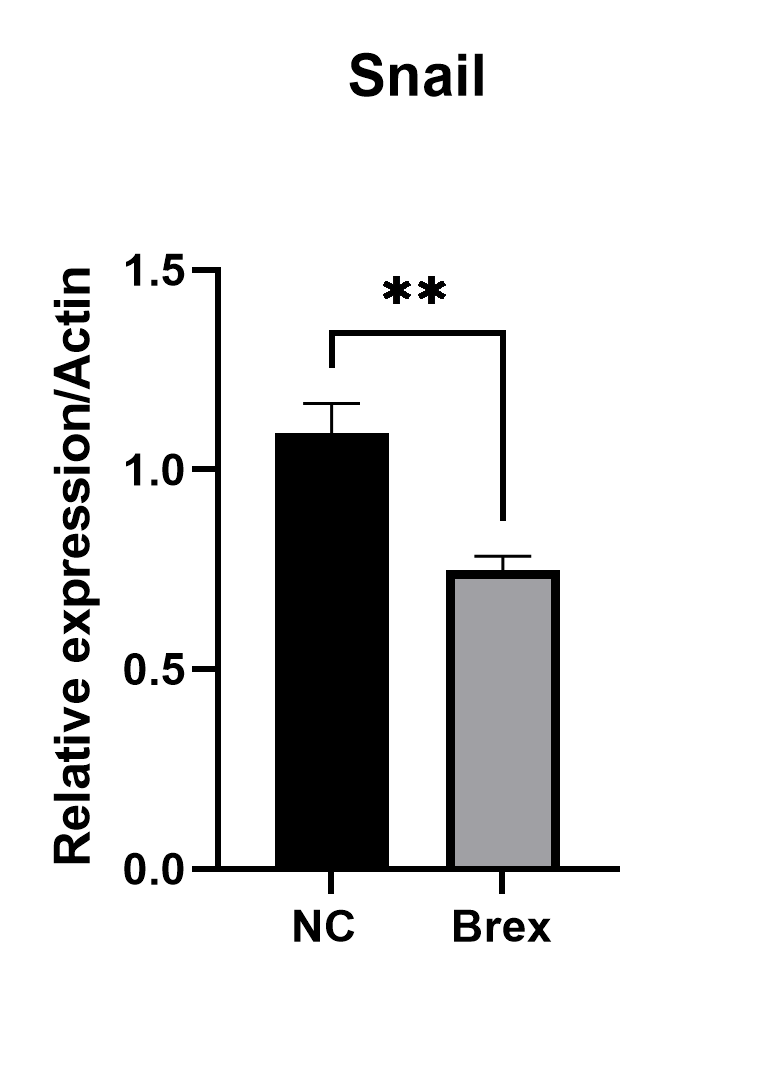

Supplement: Supplementary file 2 [file SupplementaryFile2.zip › WB数据/动物/数据分析/SNAIL.tif]

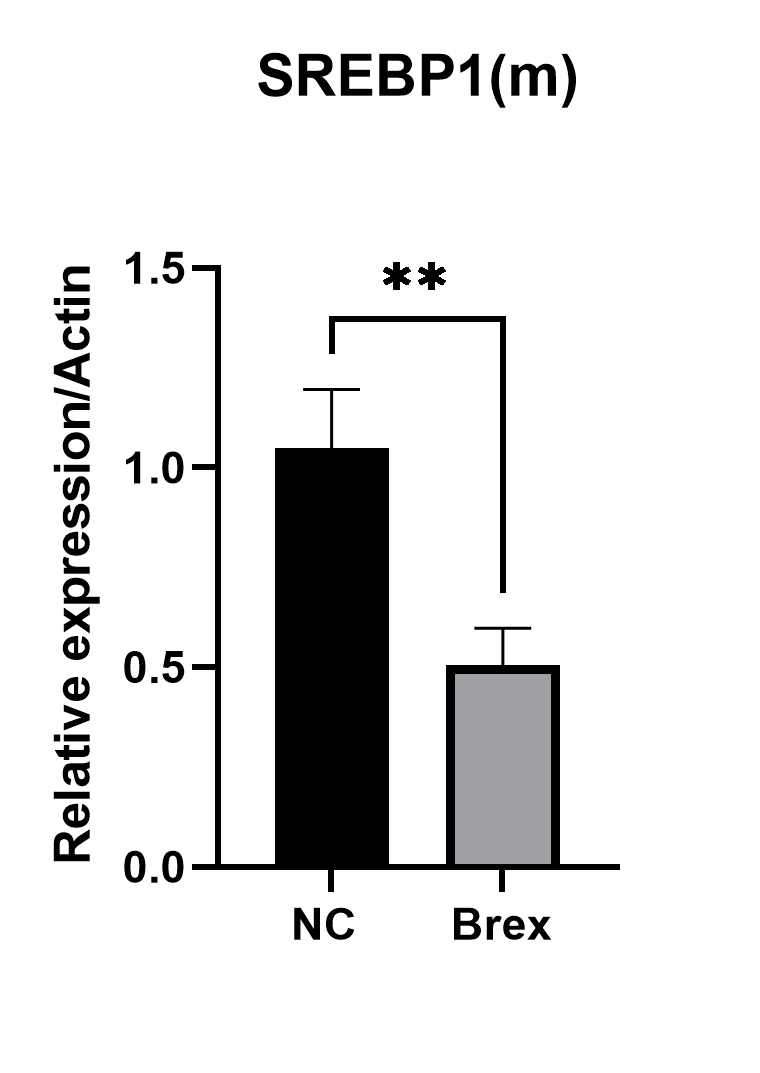

Supplement: Supplementary file 2 [file SupplementaryFile2.zip › WB数据/动物/数据分析/SREBP1.tif]

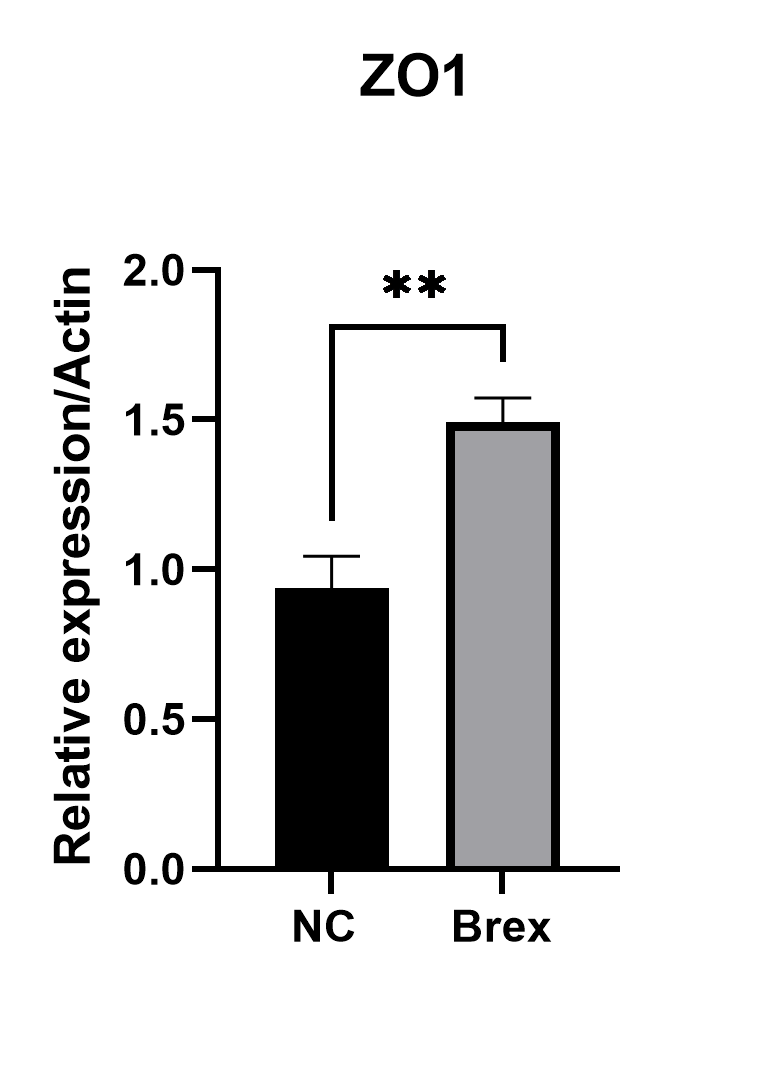

Supplement: Supplementary file 2 [file SupplementaryFile2.zip › WB数据/动物/数据分析/ZO1.tif]

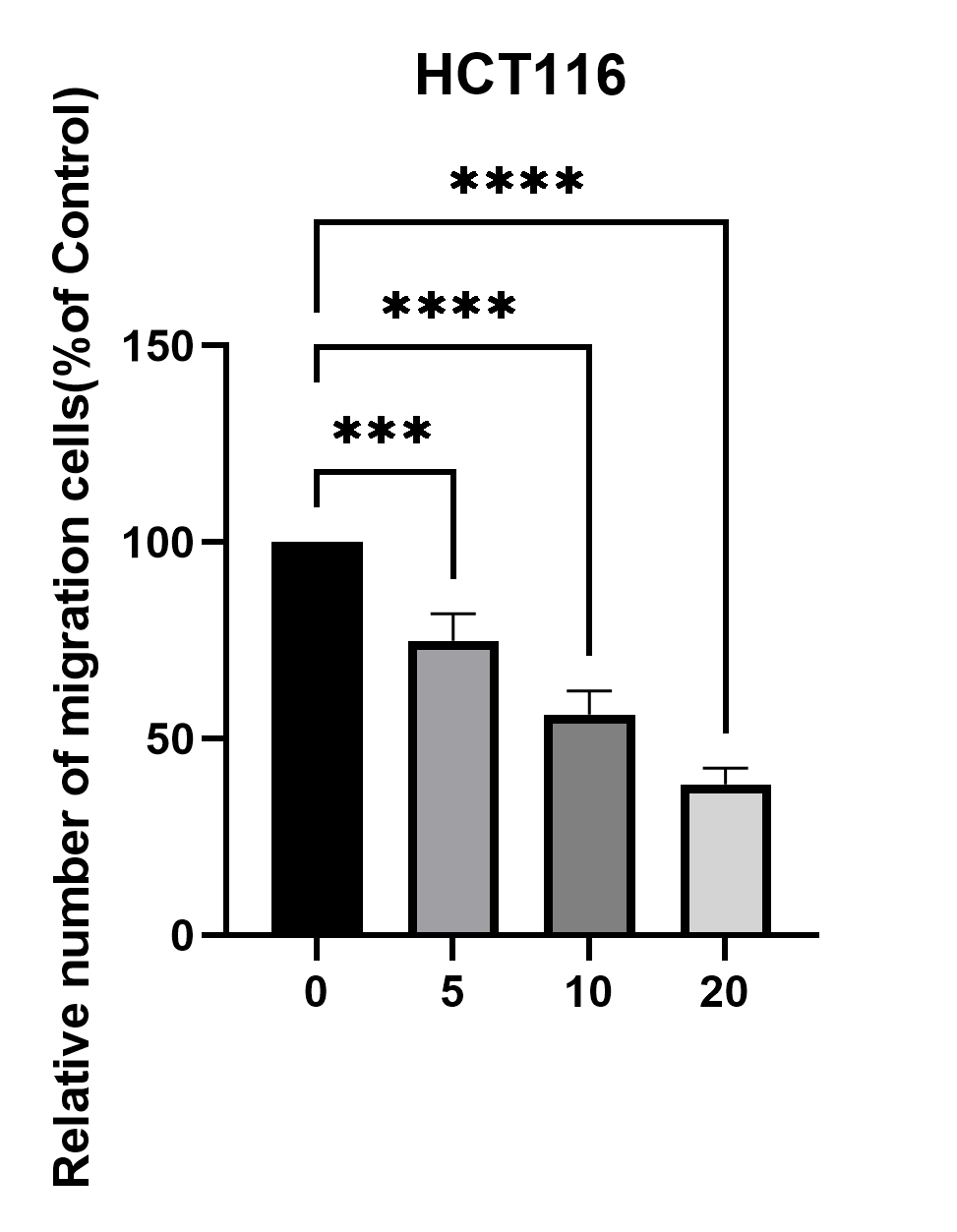

Supplement: Supplementary file 4 [file SupplementaryFile4.zip › 划痕侵袭分析/侵袭分析图/HCT116.tif]

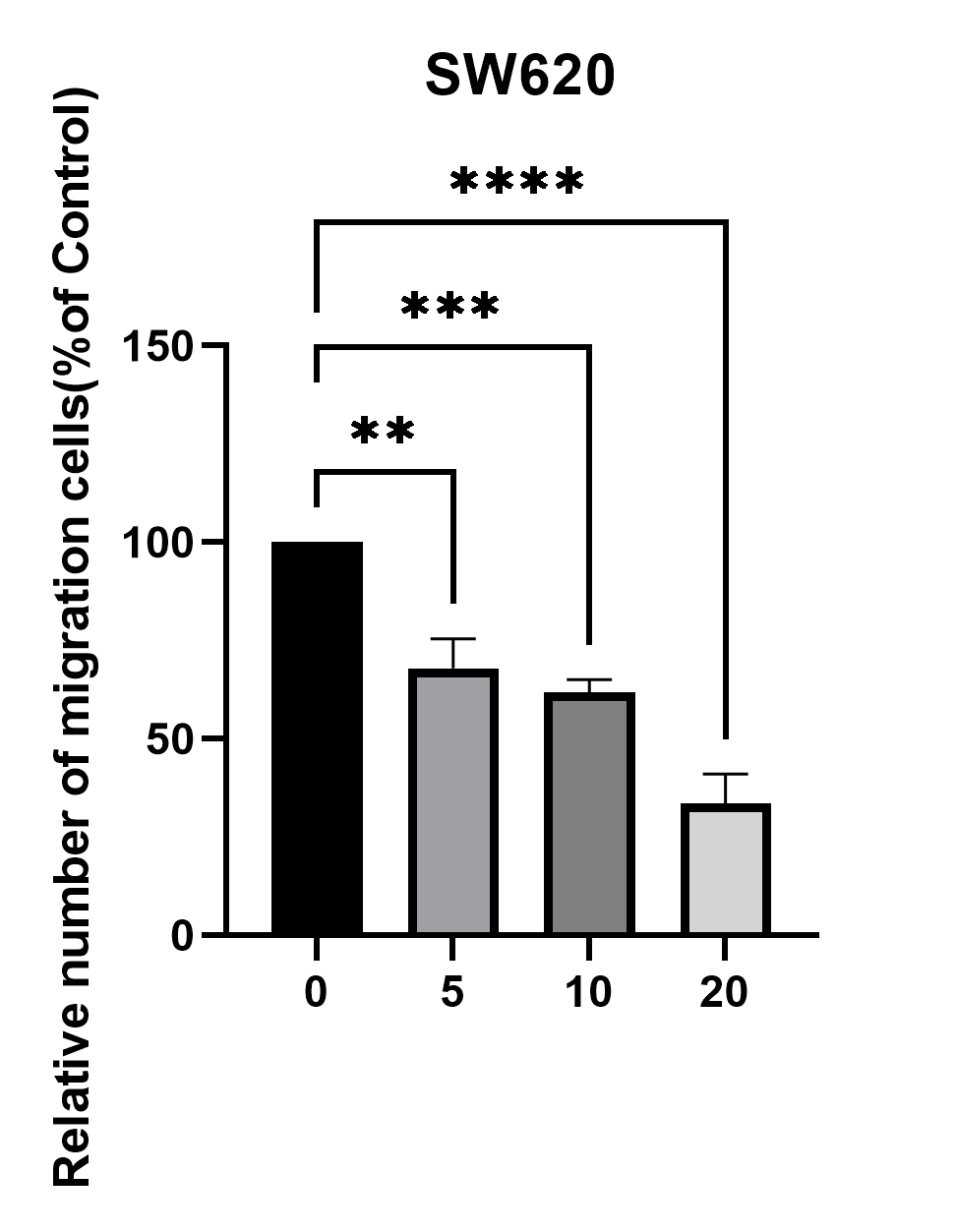

Supplement: Supplementary file 4 [file SupplementaryFile4.zip › 划痕侵袭分析/侵袭分析图/sw620.tif]

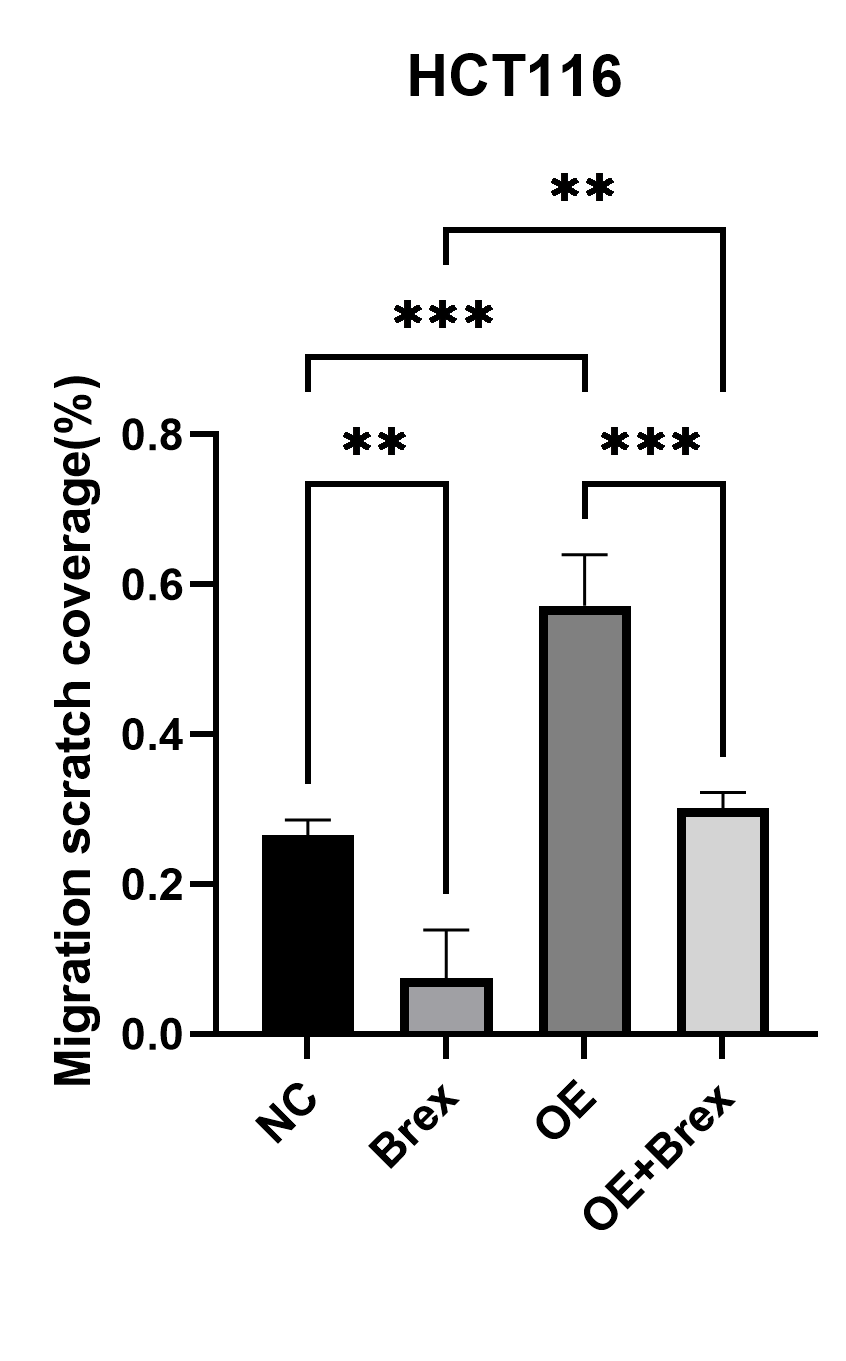

Supplement: Supplementary file 4 [file SupplementaryFile4.zip › 划痕侵袭分析/划痕分析图/HCT116-OE.tif]

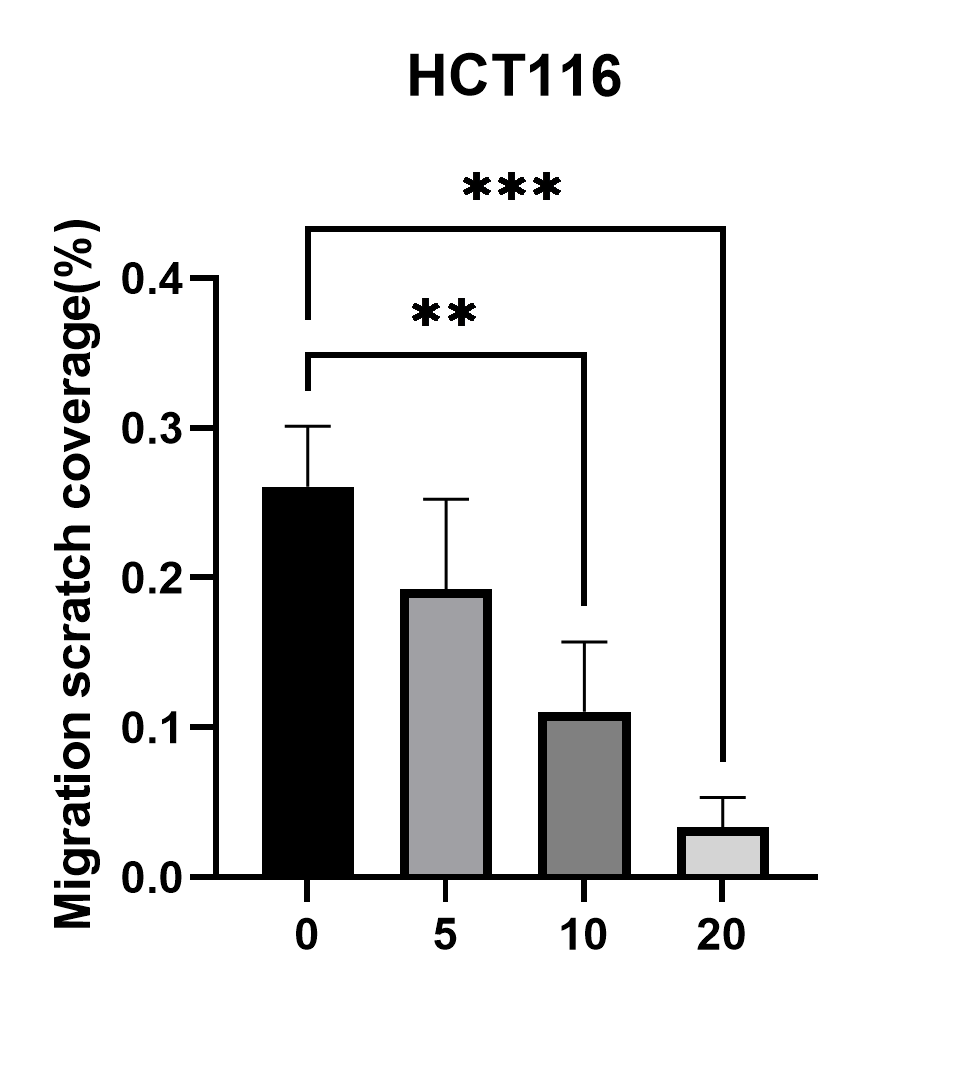

Supplement: Supplementary file 4 [file SupplementaryFile4.zip › 划痕侵袭分析/划痕分析图/HCT116.tif]

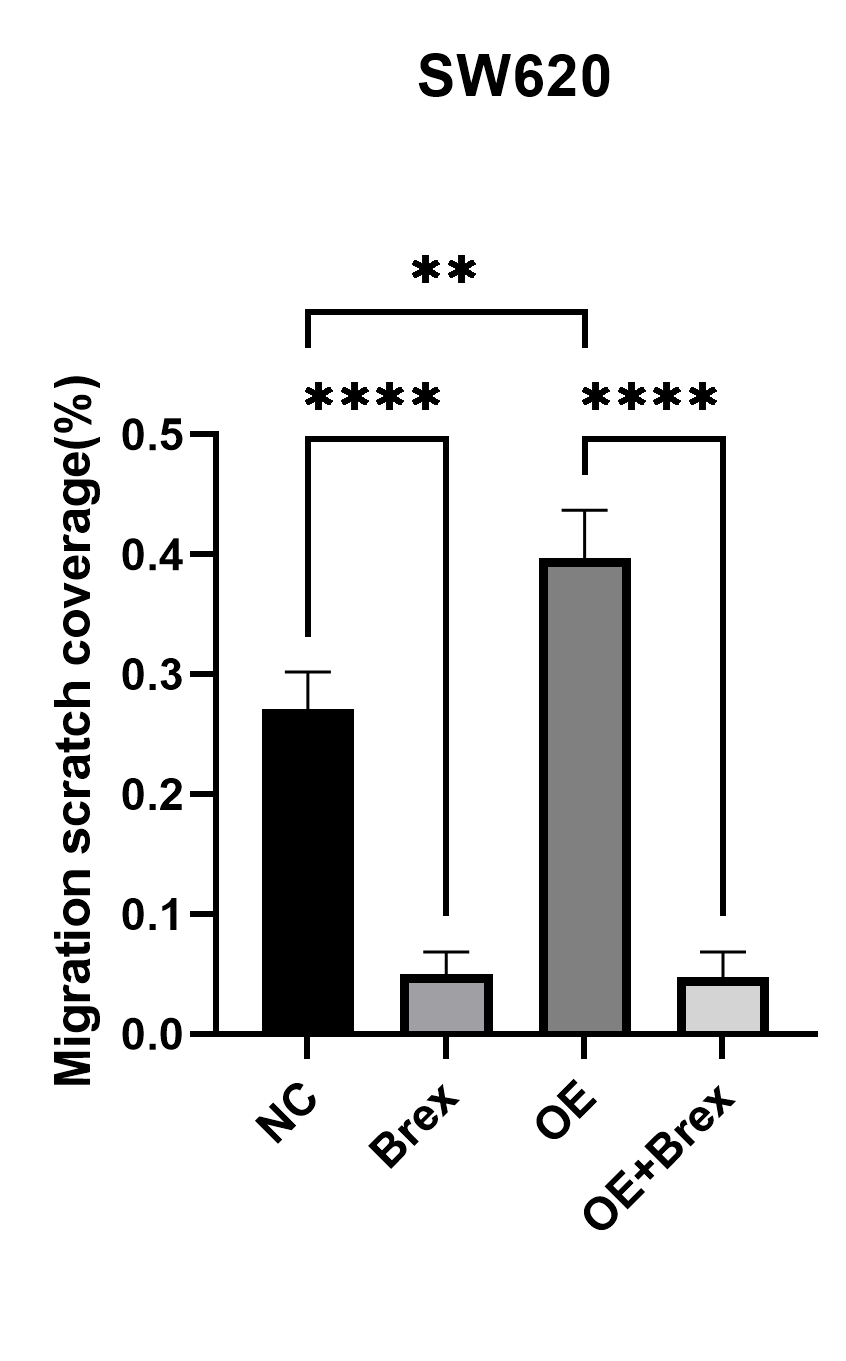

Supplement: Supplementary file 4 [file SupplementaryFile4.zip › 划痕侵袭分析/划痕分析图/SW620-OE.tif]

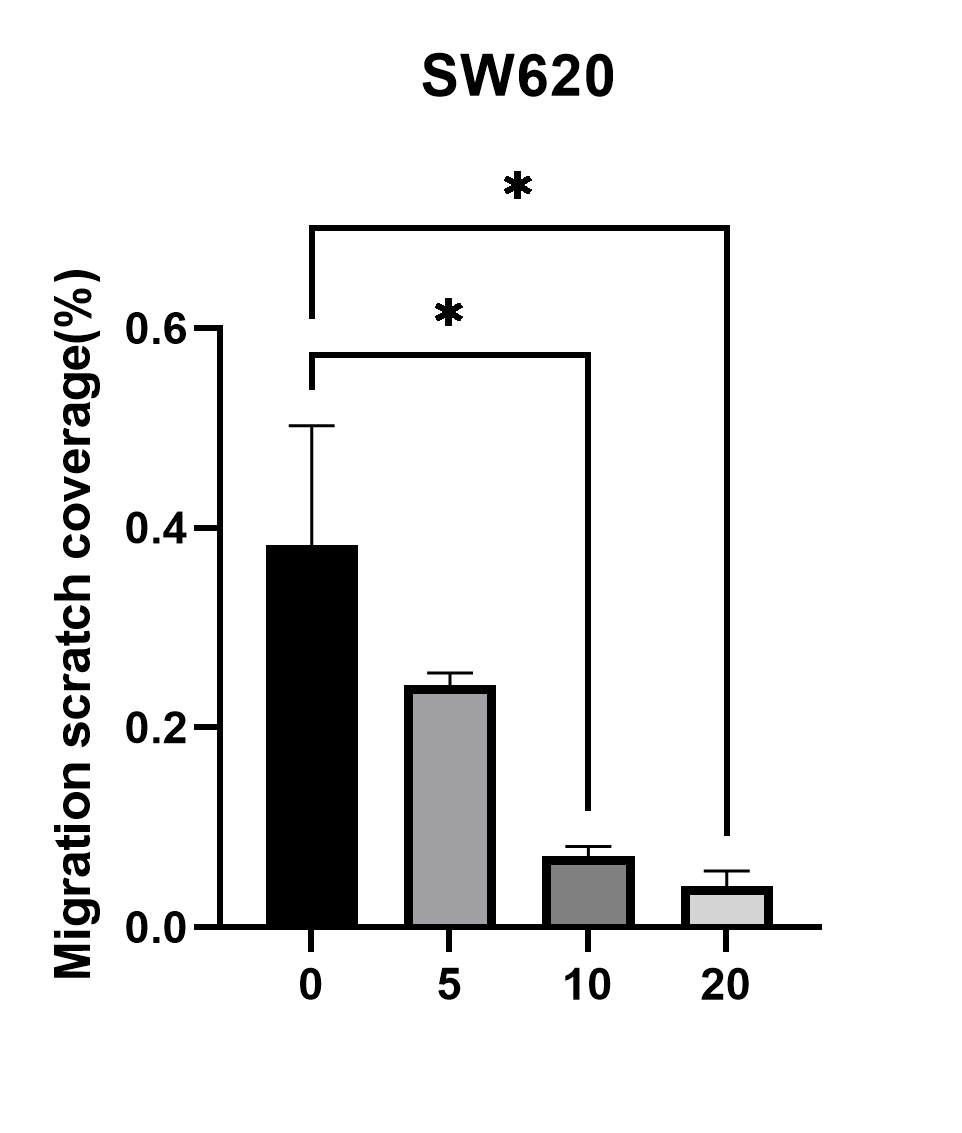

Supplement: Supplementary file 4 [file SupplementaryFile4.zip › 划痕侵袭分析/划痕分析图/SW620.tif]

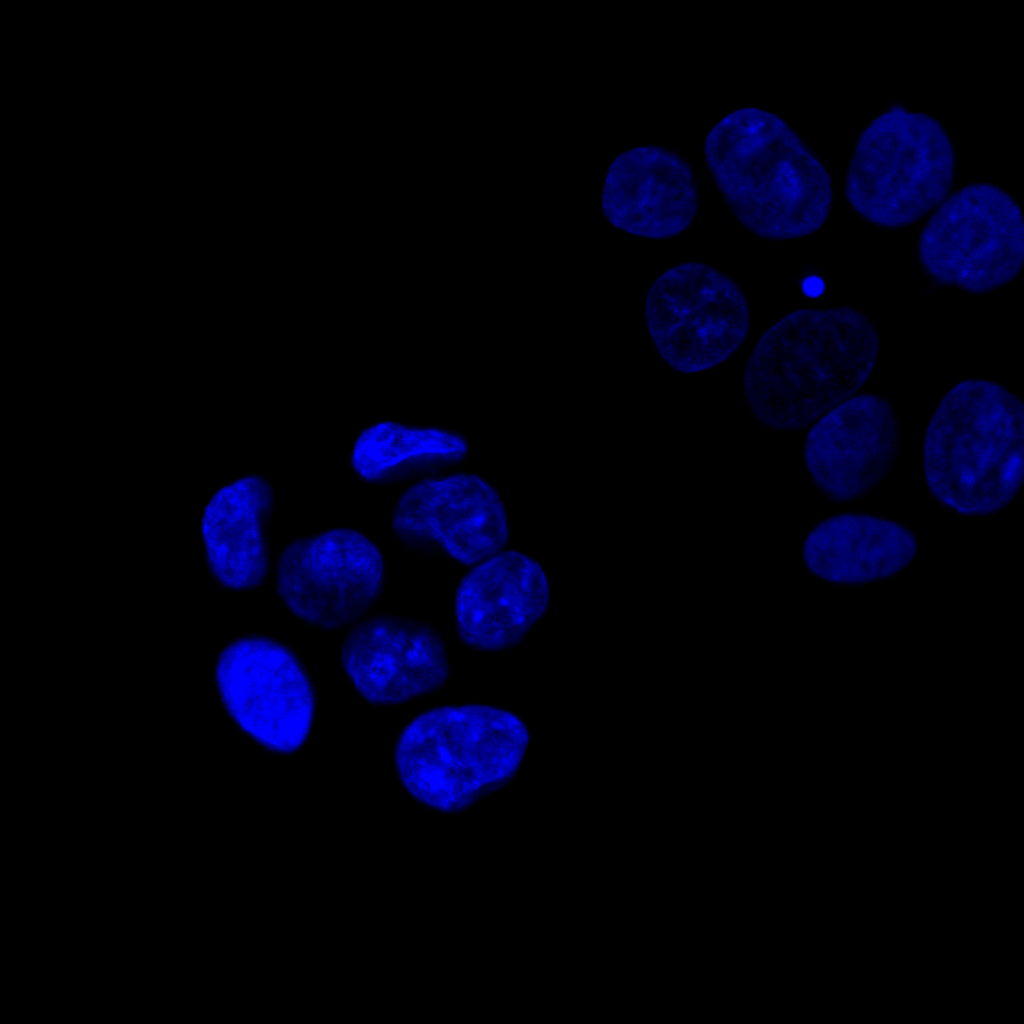

Supplement: Supplementary file 5 [file SupplementaryFile5.zip › 免疫荧光/6.13/E-Cad/lxj-BRE-E.tif.frames/lxj-BRE-E_C001T001.tif]

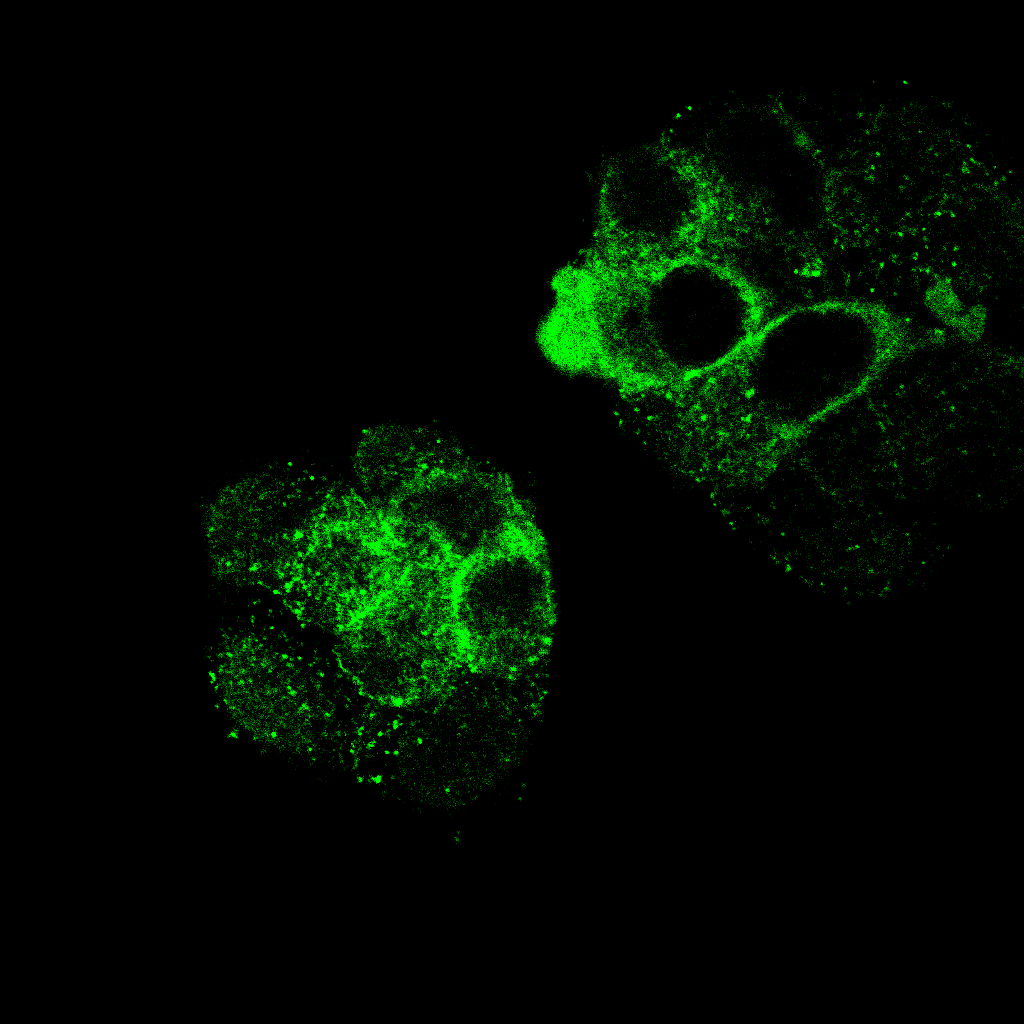

Supplement: Supplementary file 5 [file SupplementaryFile5.zip › 免疫荧光/6.13/E-Cad/lxj-BRE-E.tif.frames/lxj-BRE-E_C002T001.tif]

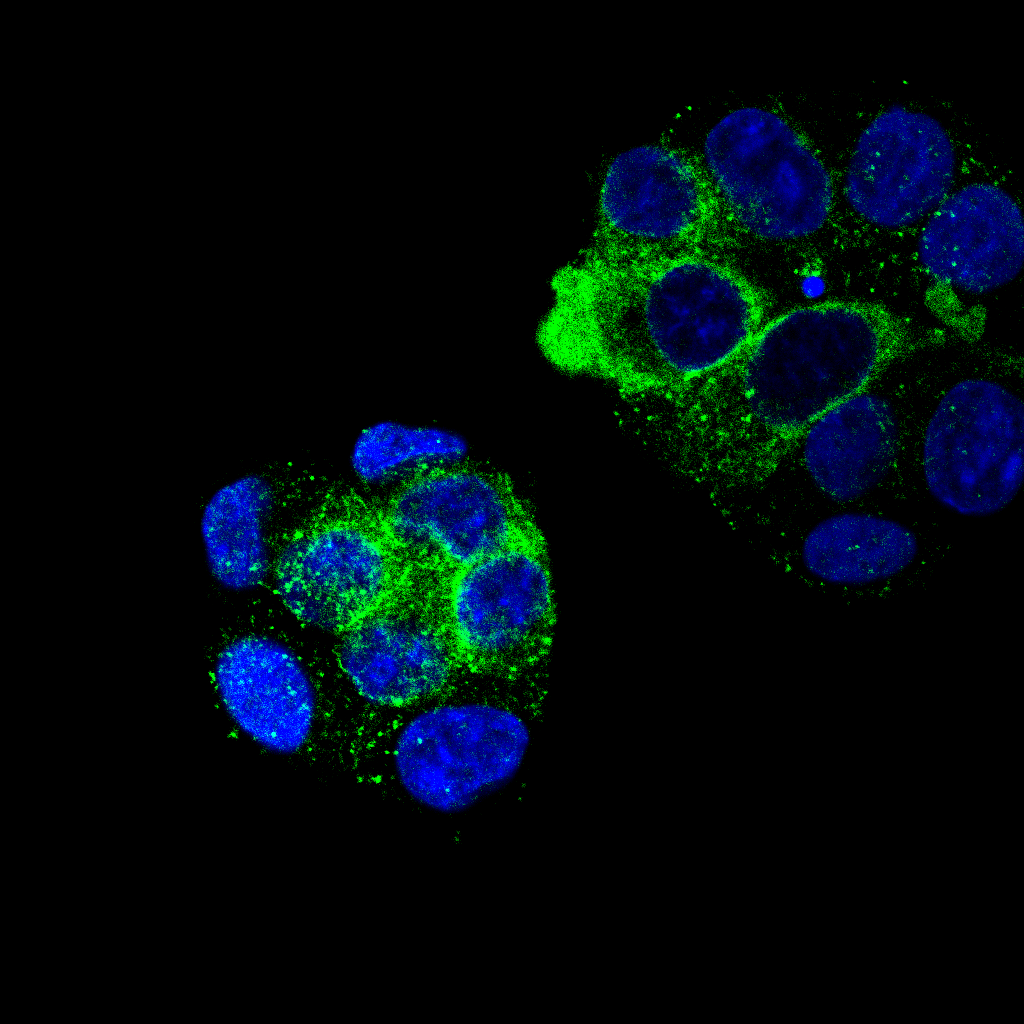

Supplement: Supplementary file 5 [file SupplementaryFile5.zip › 免疫荧光/6.13/E-Cad/lxj-BRE-E.tif.frames/lxj-BRE-E_T001.tif]

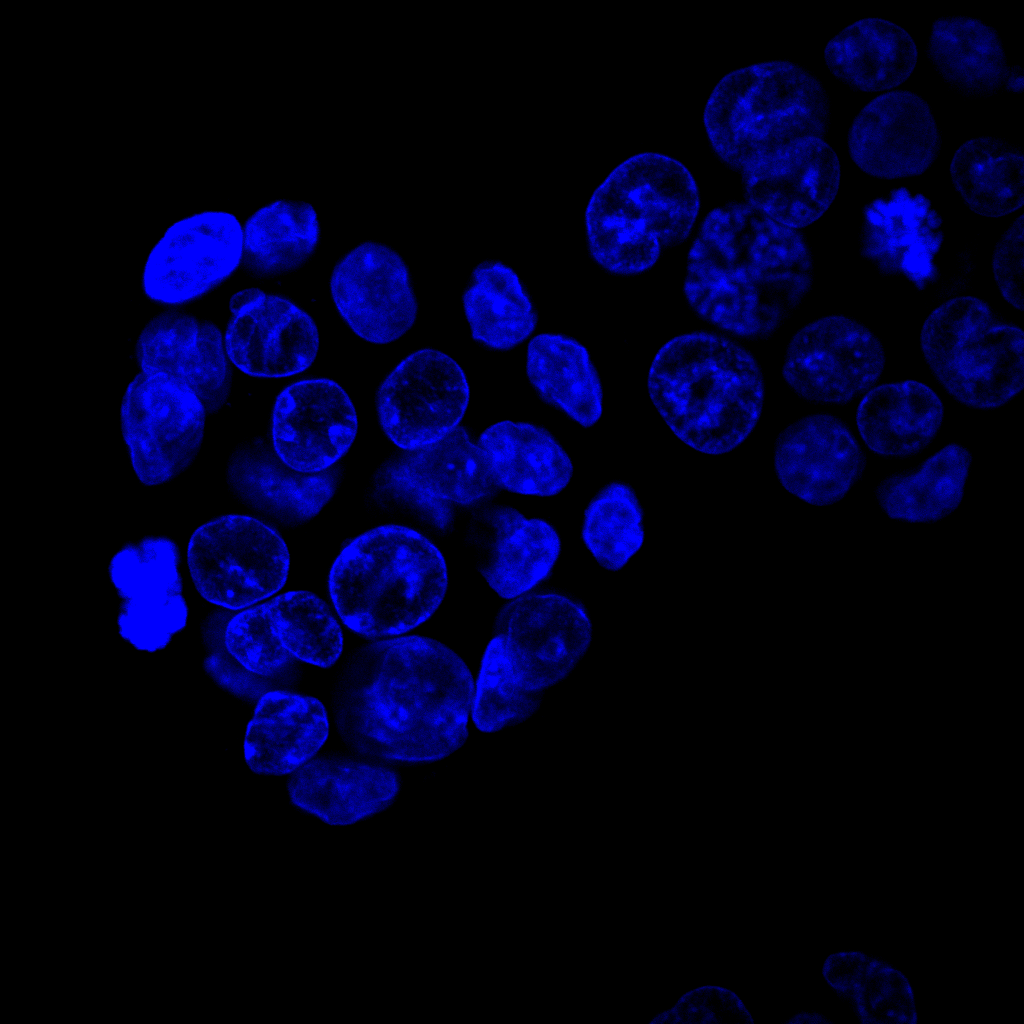

Supplement: Supplementary file 5 [file SupplementaryFile5.zip › 免疫荧光/6.13/E-Cad/lxj-BRE-E_0001.tif.frames/lxj-BRE-E_0001_C001T001.tif]

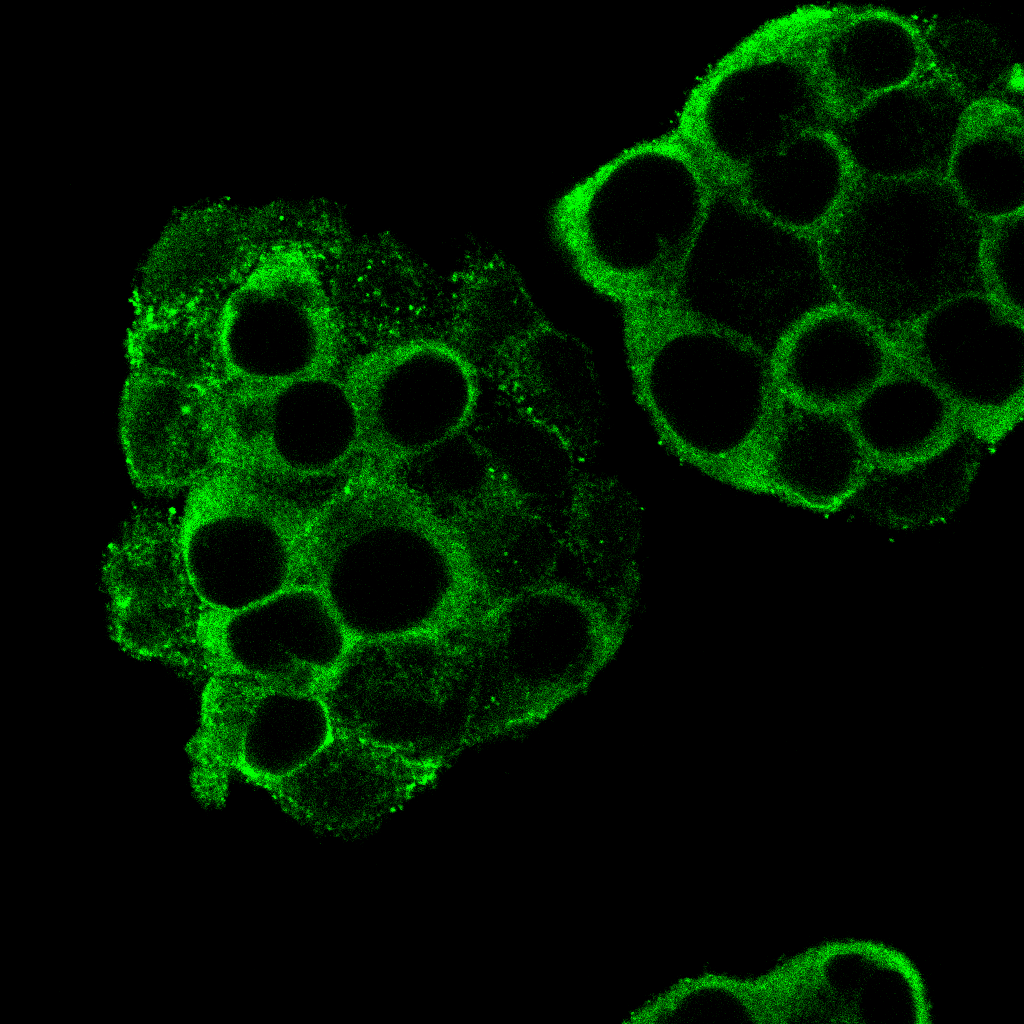

Supplement: Supplementary file 5 [file SupplementaryFile5.zip › 免疫荧光/6.13/E-Cad/lxj-BRE-E_0001.tif.frames/lxj-BRE-E_0001_C002T001.tif]

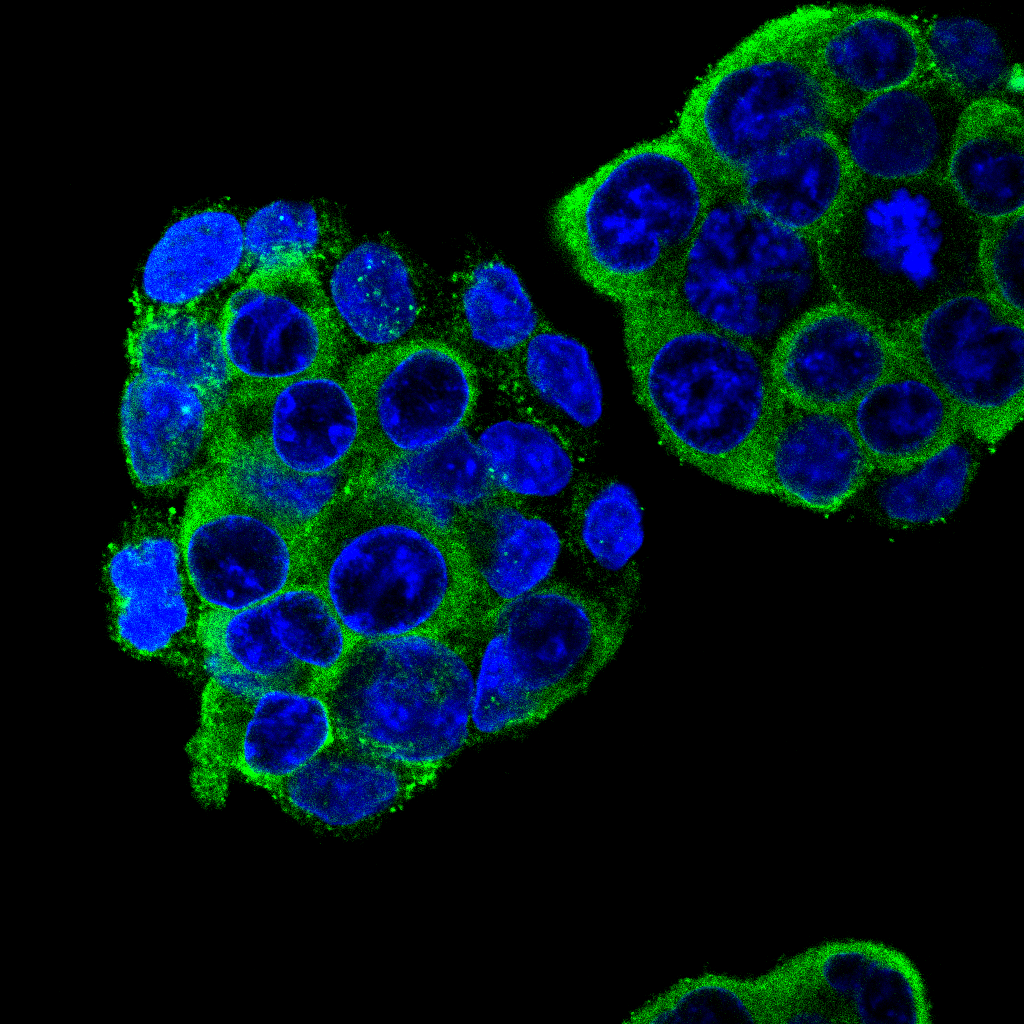

Supplement: Supplementary file 5 [file SupplementaryFile5.zip › 免疫荧光/6.13/E-Cad/lxj-BRE-E_0001.tif.frames/lxj-BRE-E_0001_T001.tif]

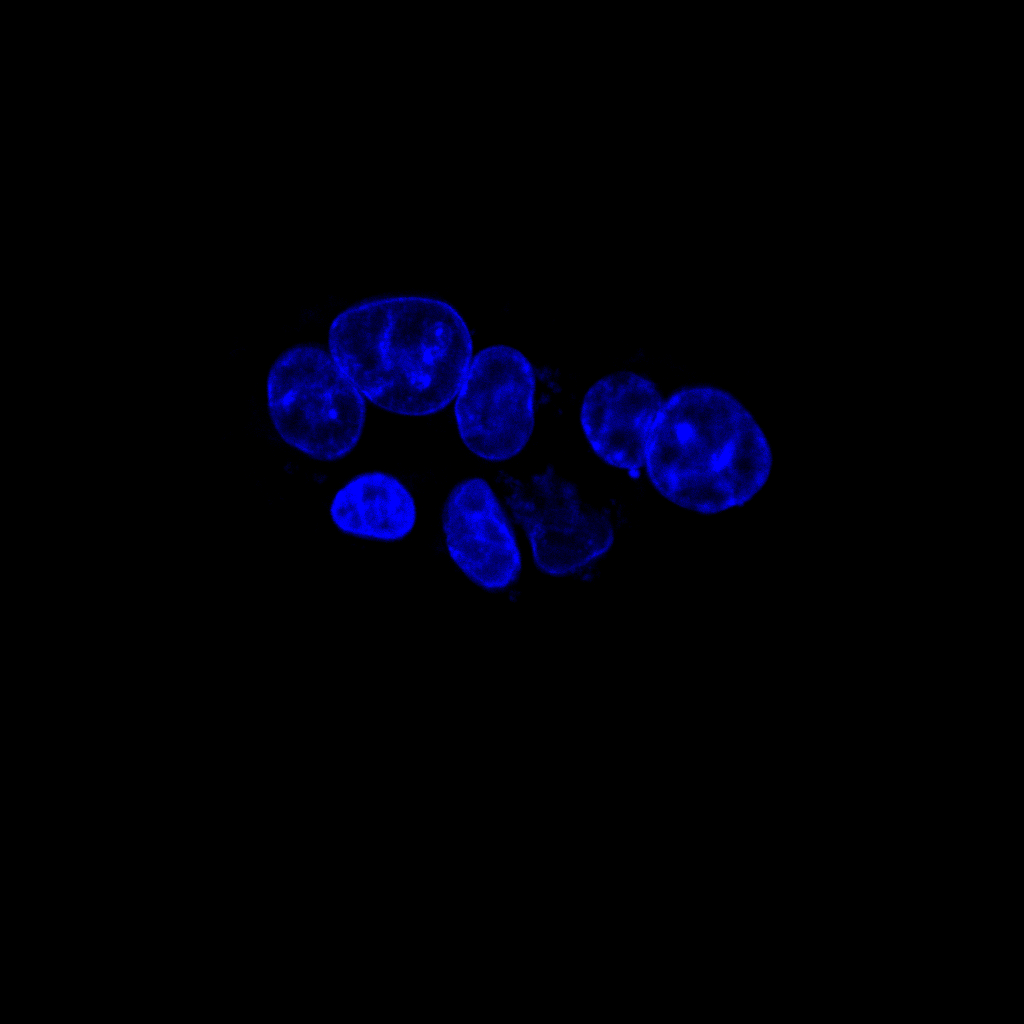

Supplement: Supplementary file 5 [file SupplementaryFile5.zip › 免疫荧光/6.13/E-Cad/lxj-BRE-E_0002.tif.frames/lxj-BRE-E_0002_C001T001.tif]

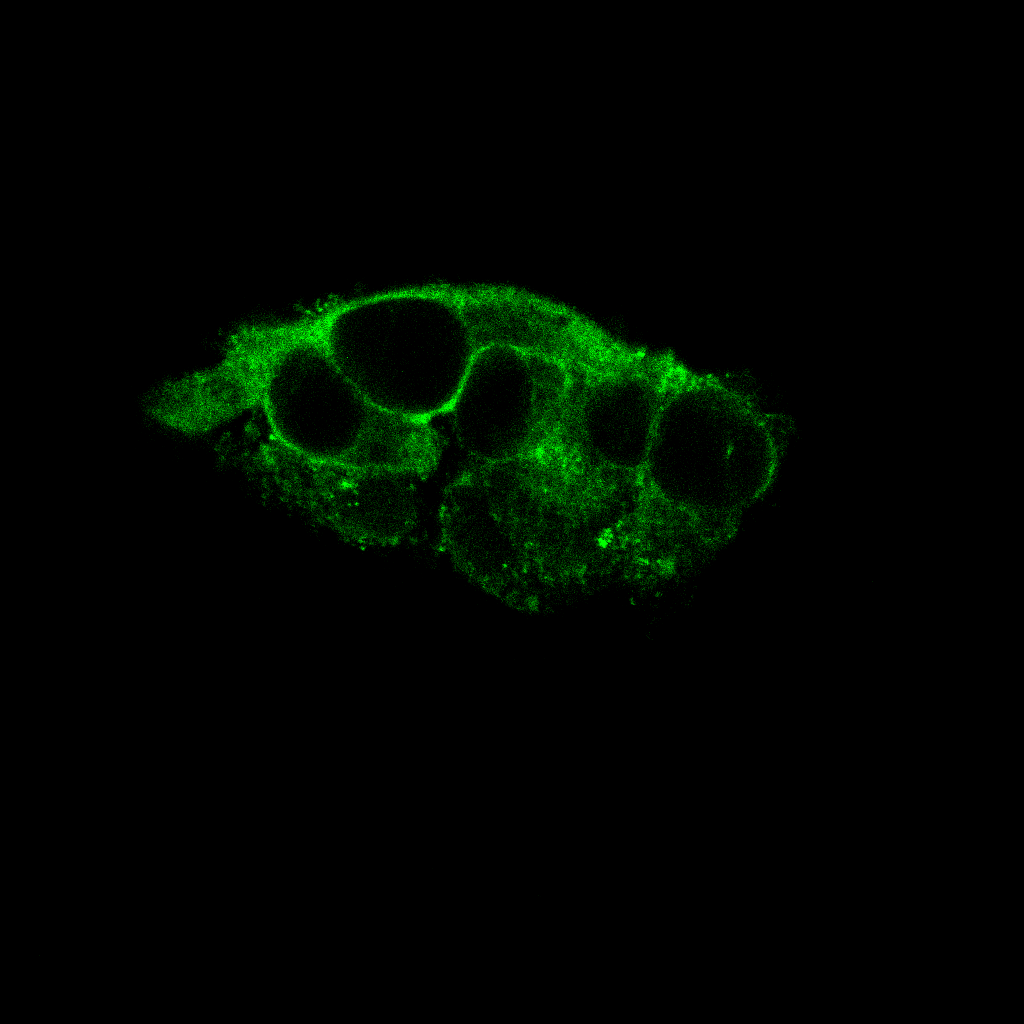

Supplement: Supplementary file 5 [file SupplementaryFile5.zip › 免疫荧光/6.13/E-Cad/lxj-BRE-E_0002.tif.frames/lxj-BRE-E_0002_C002T001.tif]

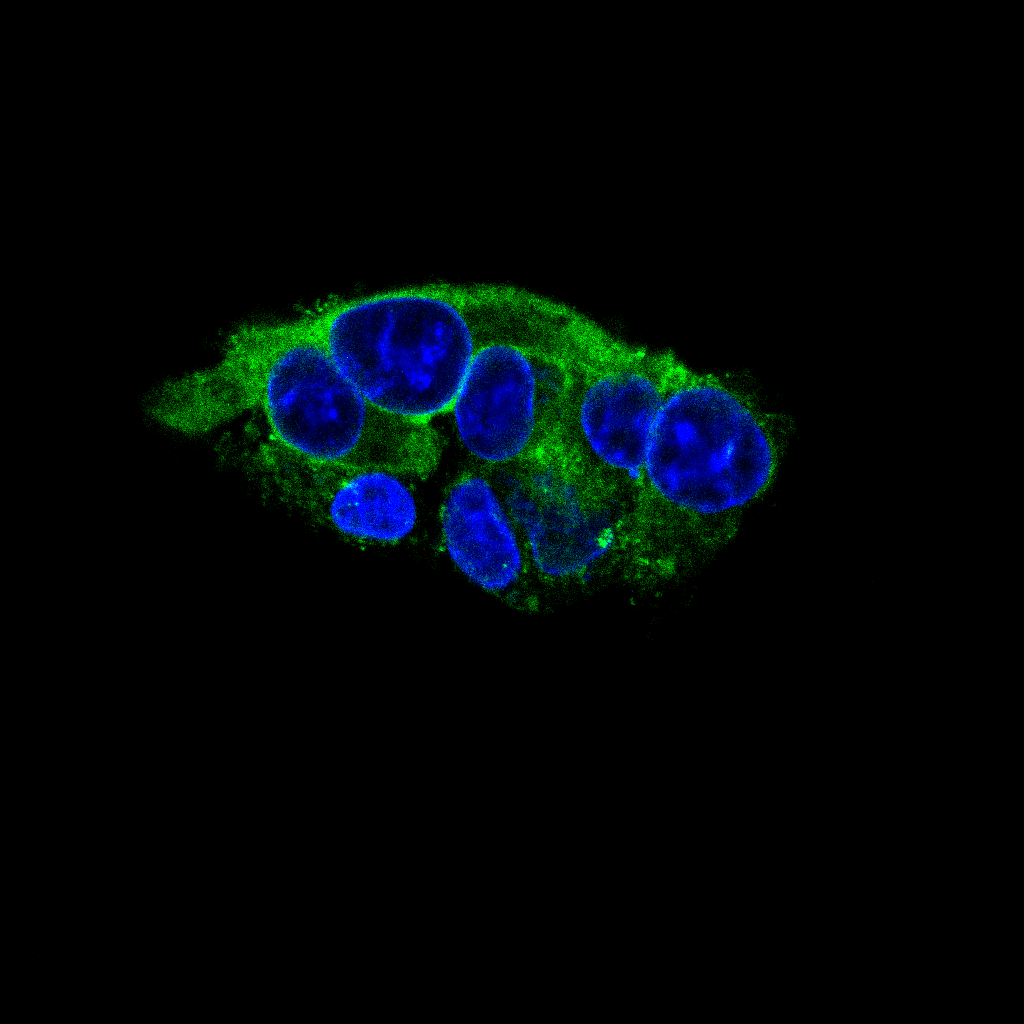

Supplement: Supplementary file 5 [file SupplementaryFile5.zip › 免疫荧光/6.13/E-Cad/lxj-BRE-E_0002.tif.frames/lxj-BRE-E_0002_T001.tif]

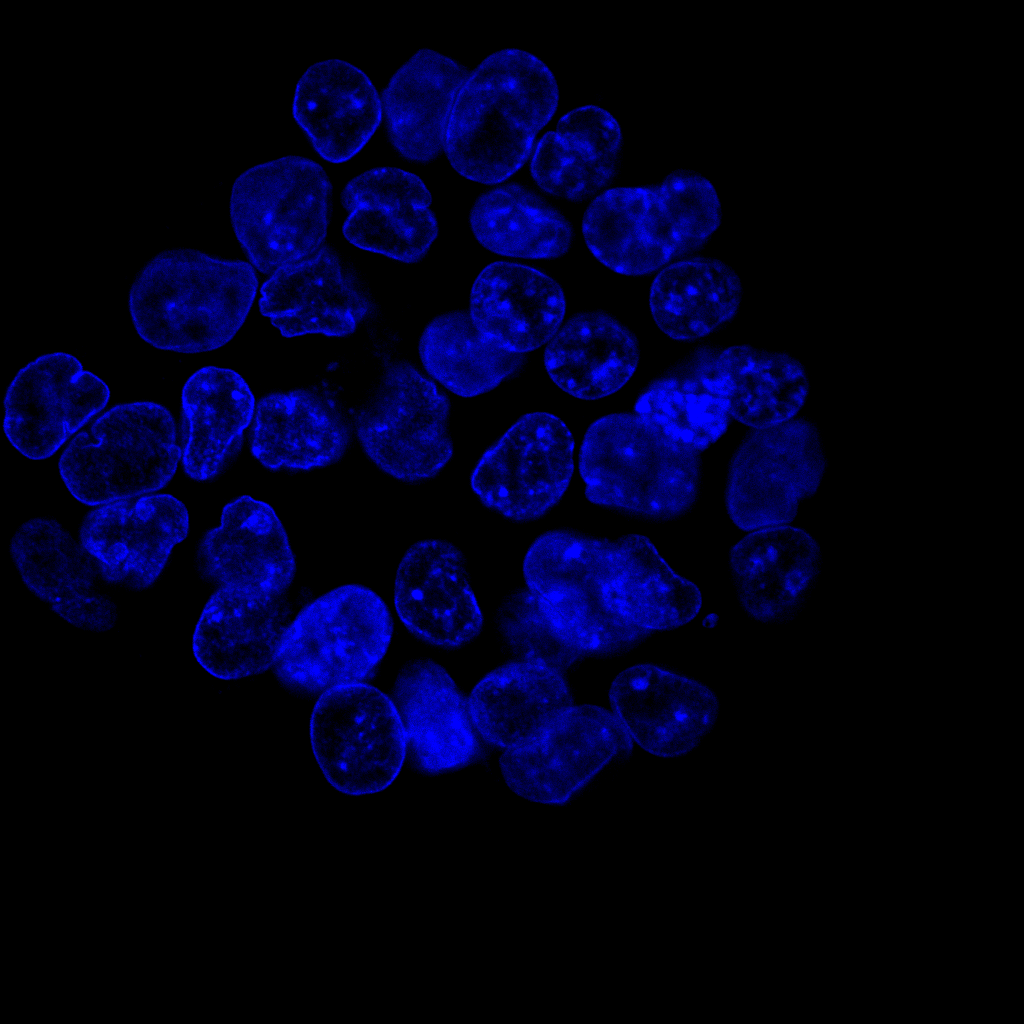

Supplement: Supplementary file 5 [file SupplementaryFile5.zip › 免疫荧光/6.13/E-Cad/lxj-BRE-E_0003.tif.frames/lxj-BRE-E_0003_C001T001.tif]

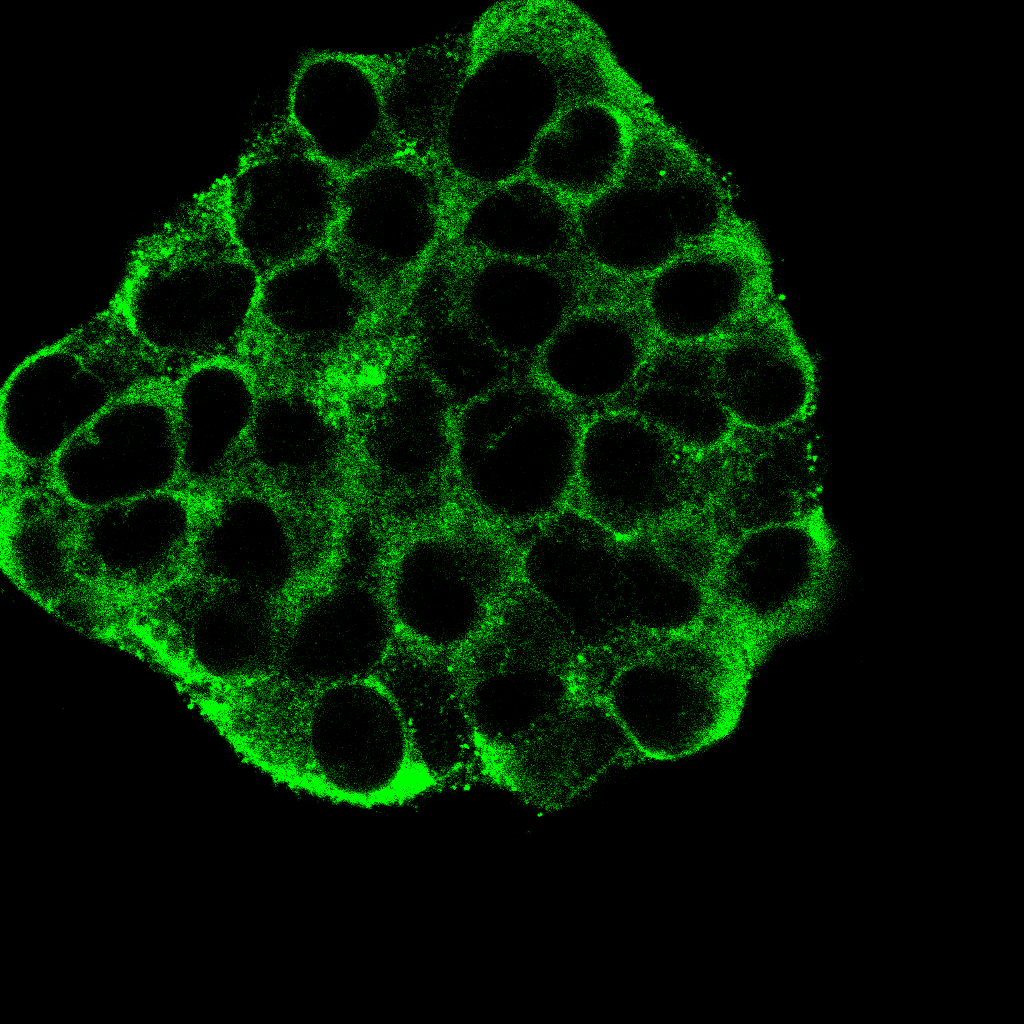

Supplement: Supplementary file 5 [file SupplementaryFile5.zip › 免疫荧光/6.13/E-Cad/lxj-BRE-E_0003.tif.frames/lxj-BRE-E_0003_C002T001.tif]

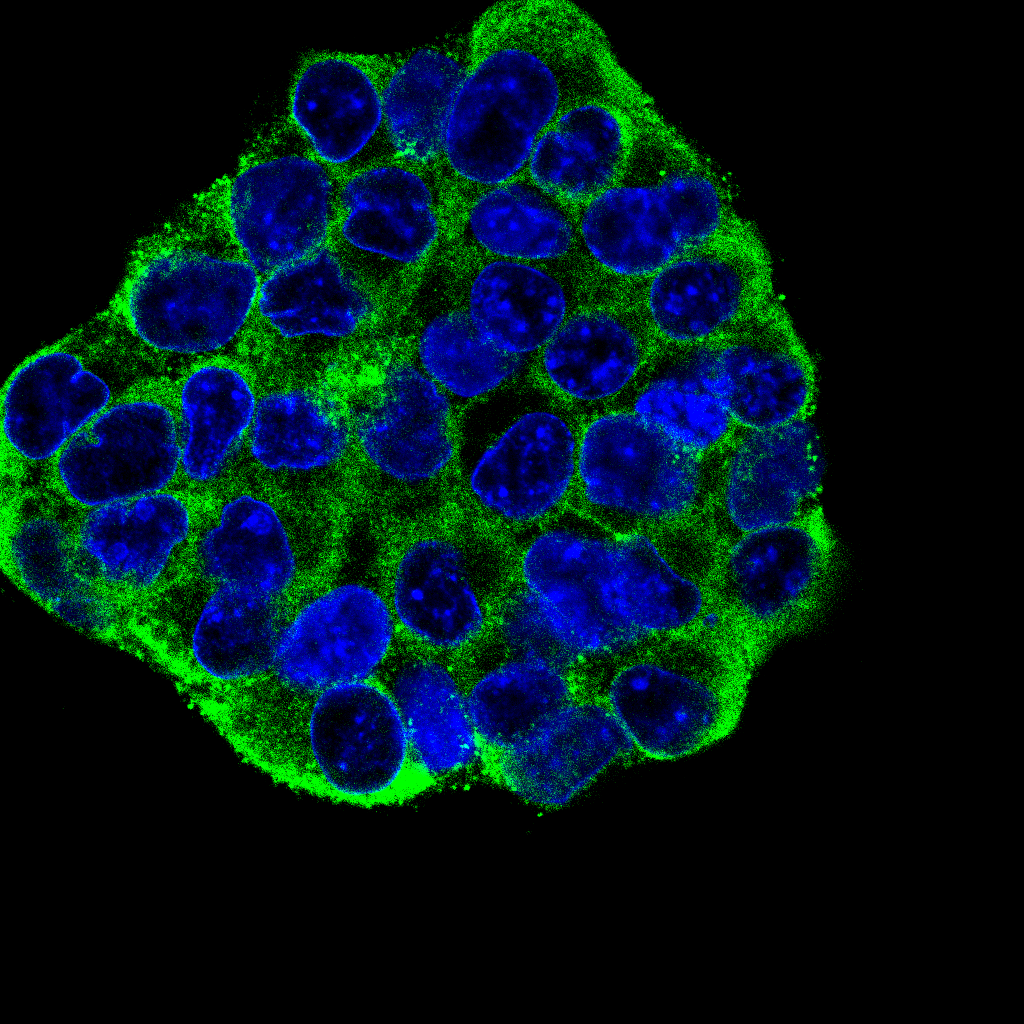

Supplement: Supplementary file 5 [file SupplementaryFile5.zip › 免疫荧光/6.13/E-Cad/lxj-BRE-E_0003.tif.frames/lxj-BRE-E_0003_T001.tif]

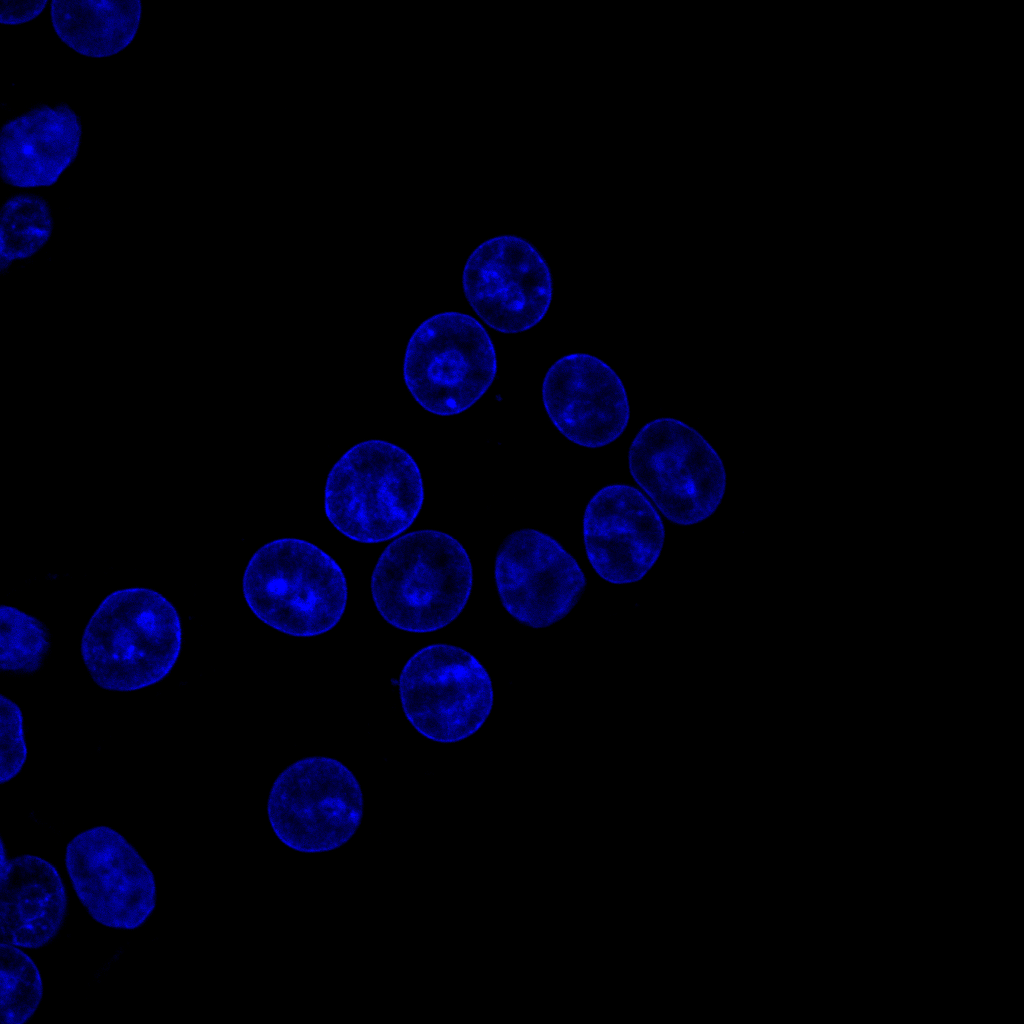

Supplement: Supplementary file 5 [file SupplementaryFile5.zip › 免疫荧光/6.13/E-Cad/lxj-BRE-E_0004.tif.frames/lxj-BRE-E_0004_C001T001.tif]

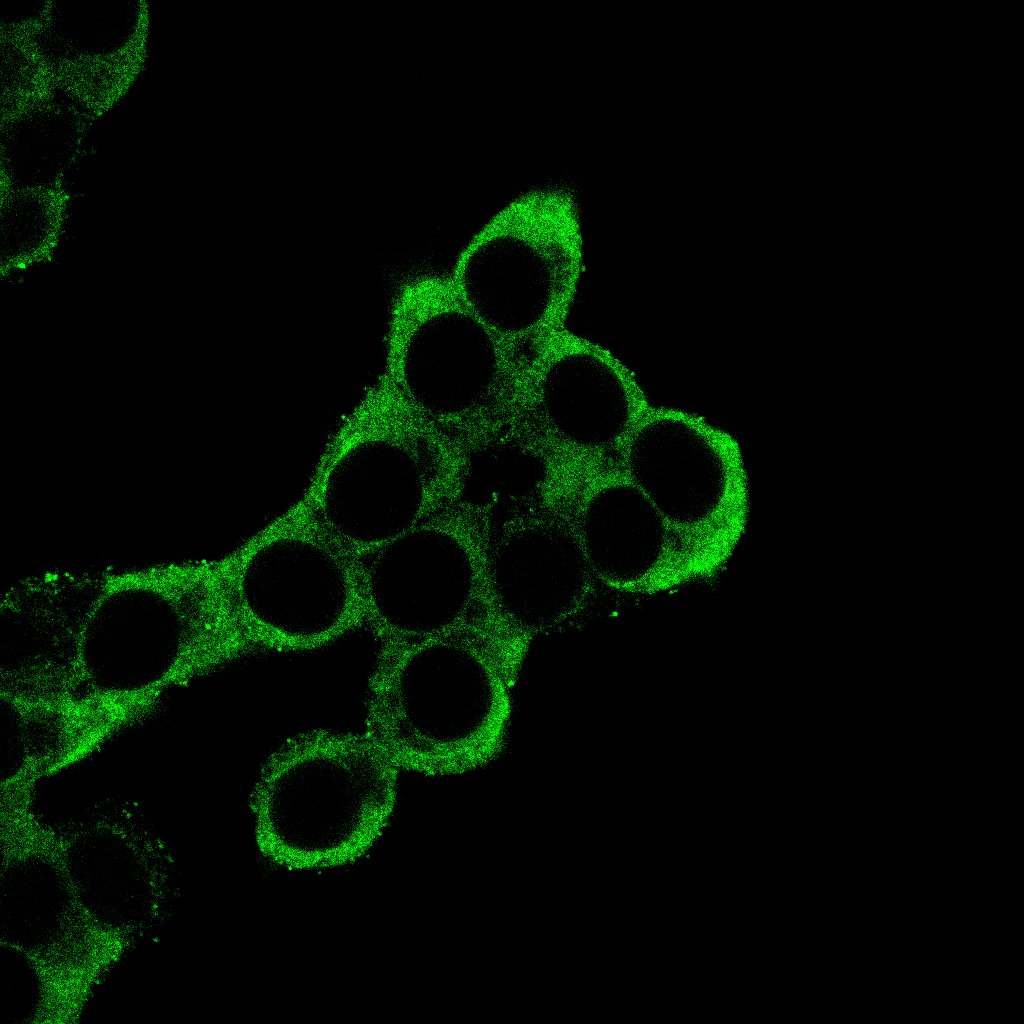

Supplement: Supplementary file 5 [file SupplementaryFile5.zip › 免疫荧光/6.13/E-Cad/lxj-BRE-E_0004.tif.frames/lxj-BRE-E_0004_C002T001.tif]
