# Supplementary figures and images for: Brexpiprazole inhibits EMT and migration of colorectal cancer cells by downregulating the SREBP1/SNAI1 signaling pathway (part 4 of 4)
Source: Front Oncol. 2026 Jan 15;15:1734678. doi: 10.3389/fonc.2025.1734678 (PMC12852020; doi:10.3389/fonc.2025.1734678)

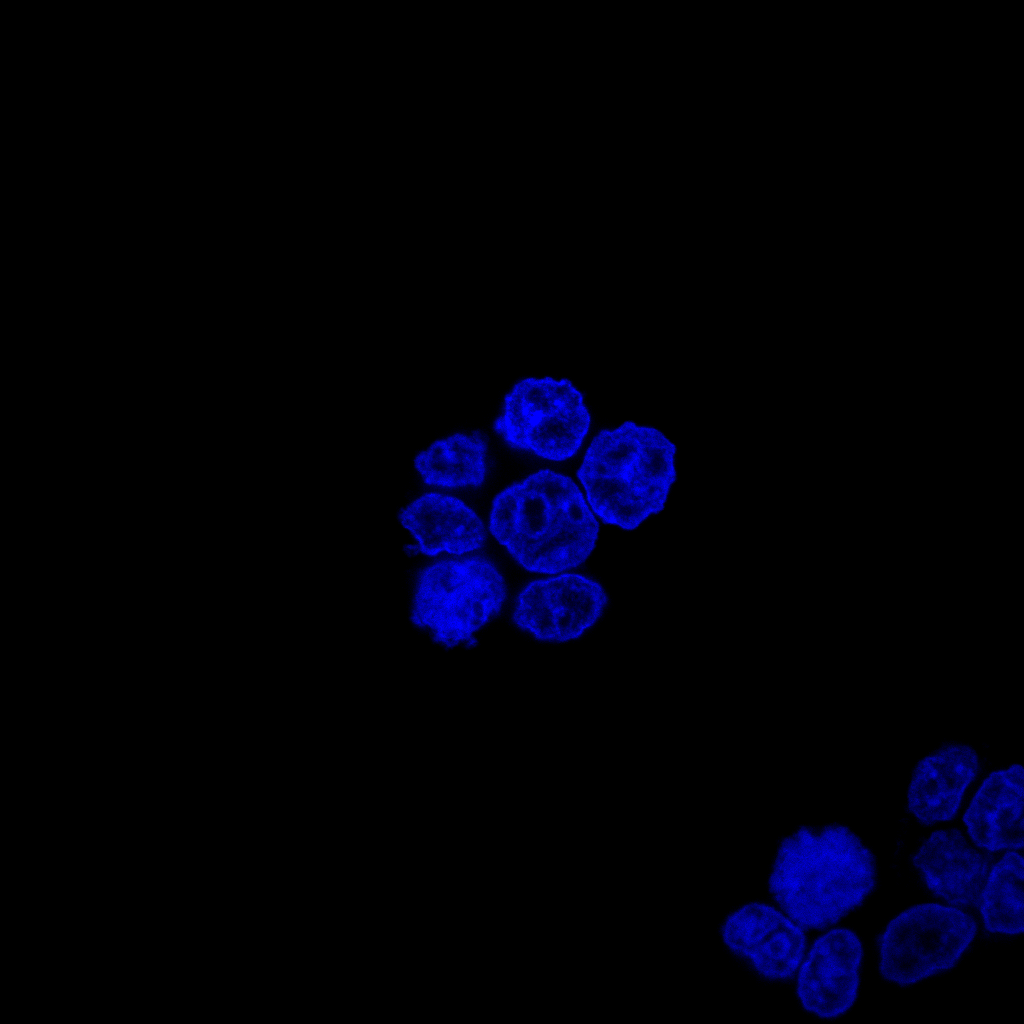

Supplement: Supplementary file 5 [file SupplementaryFile5.zip › 免疫荧光/6.18/BR1-Y_0003.tif.frames/BR1-Y_0003_C001T001.tif]

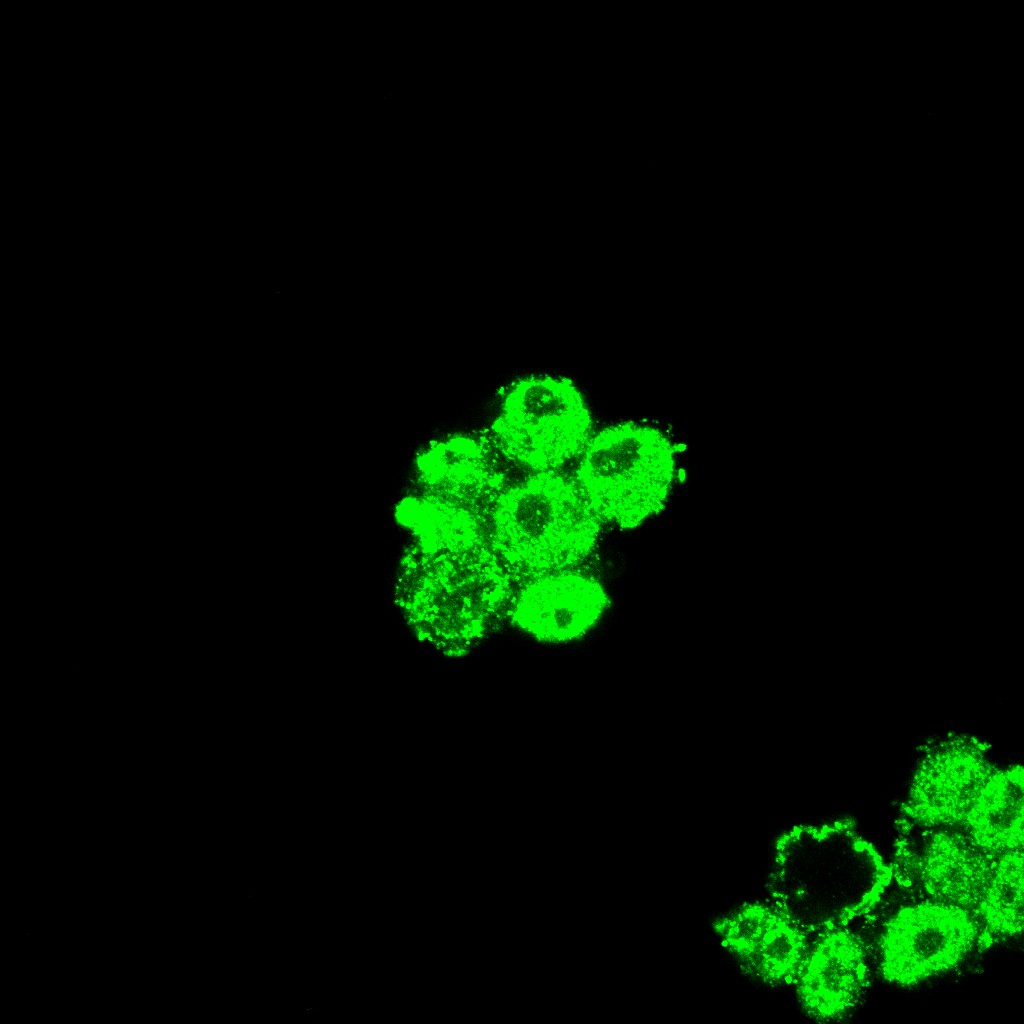

Supplement: Supplementary file 5 [file SupplementaryFile5.zip › 免疫荧光/6.18/BR1-Y_0003.tif.frames/BR1-Y_0003_C002T001.tif]

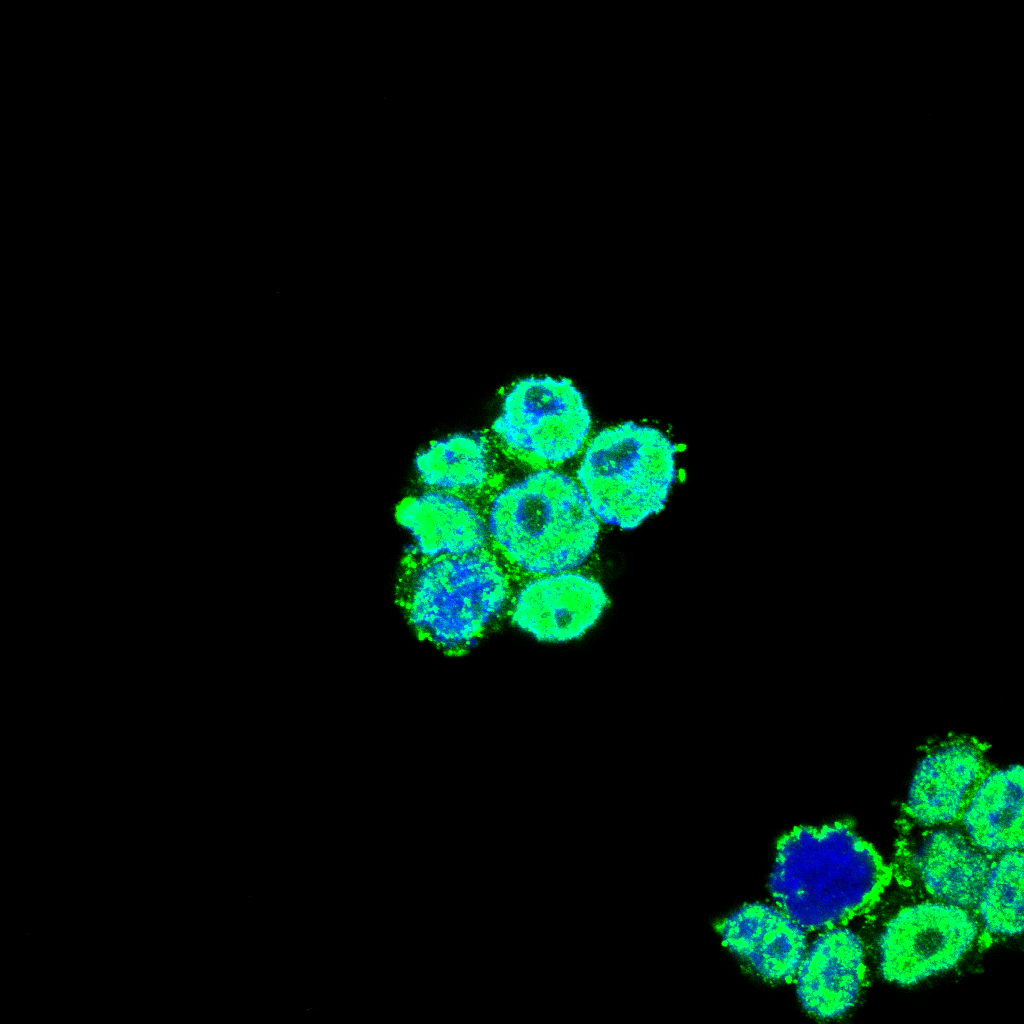

Supplement: Supplementary file 5 [file SupplementaryFile5.zip › 免疫荧光/6.18/BR1-Y_0003.tif.frames/BR1-Y_0003_T001.tif]

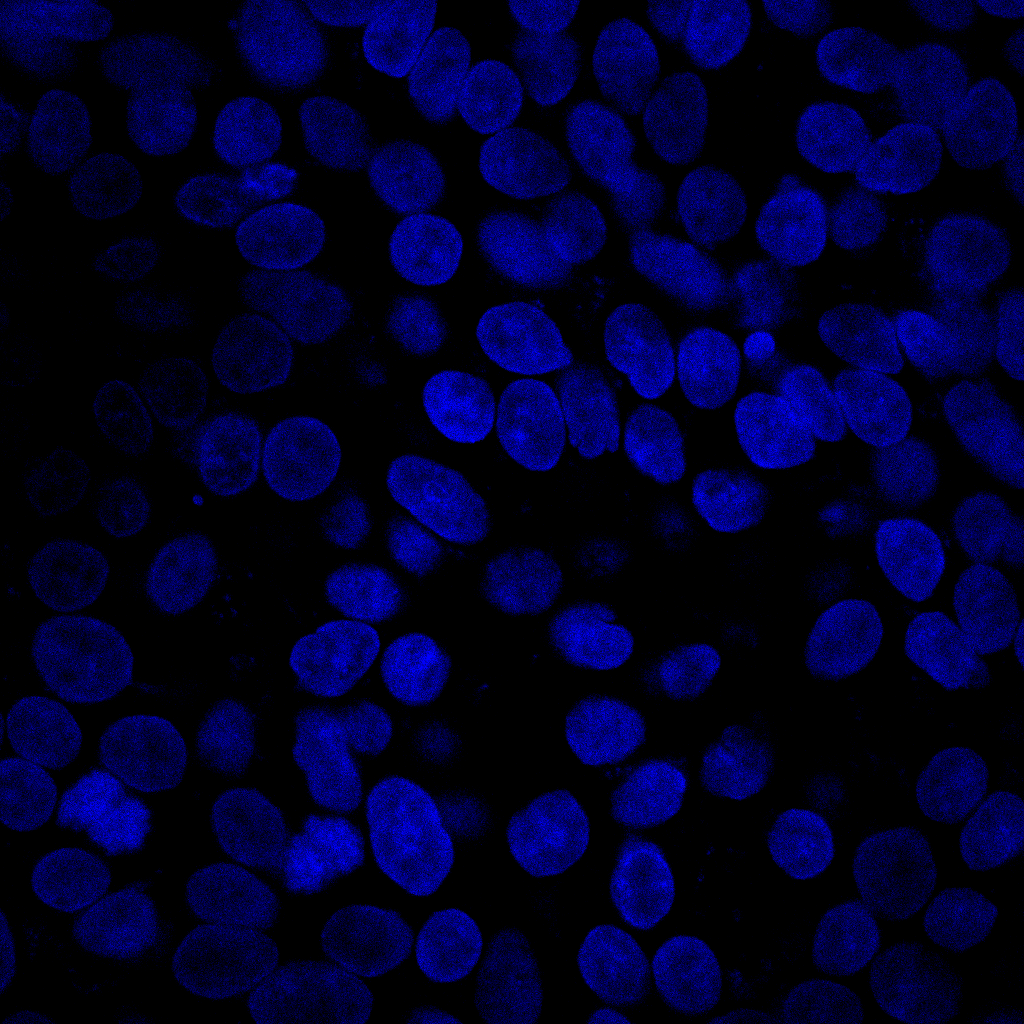

Supplement: Supplementary file 5 [file SupplementaryFile5.zip › 免疫荧光/6.25/E-Cad/488-E-BRE.tif.frames/488-E-BRE_C001T001 - 副本.tif]

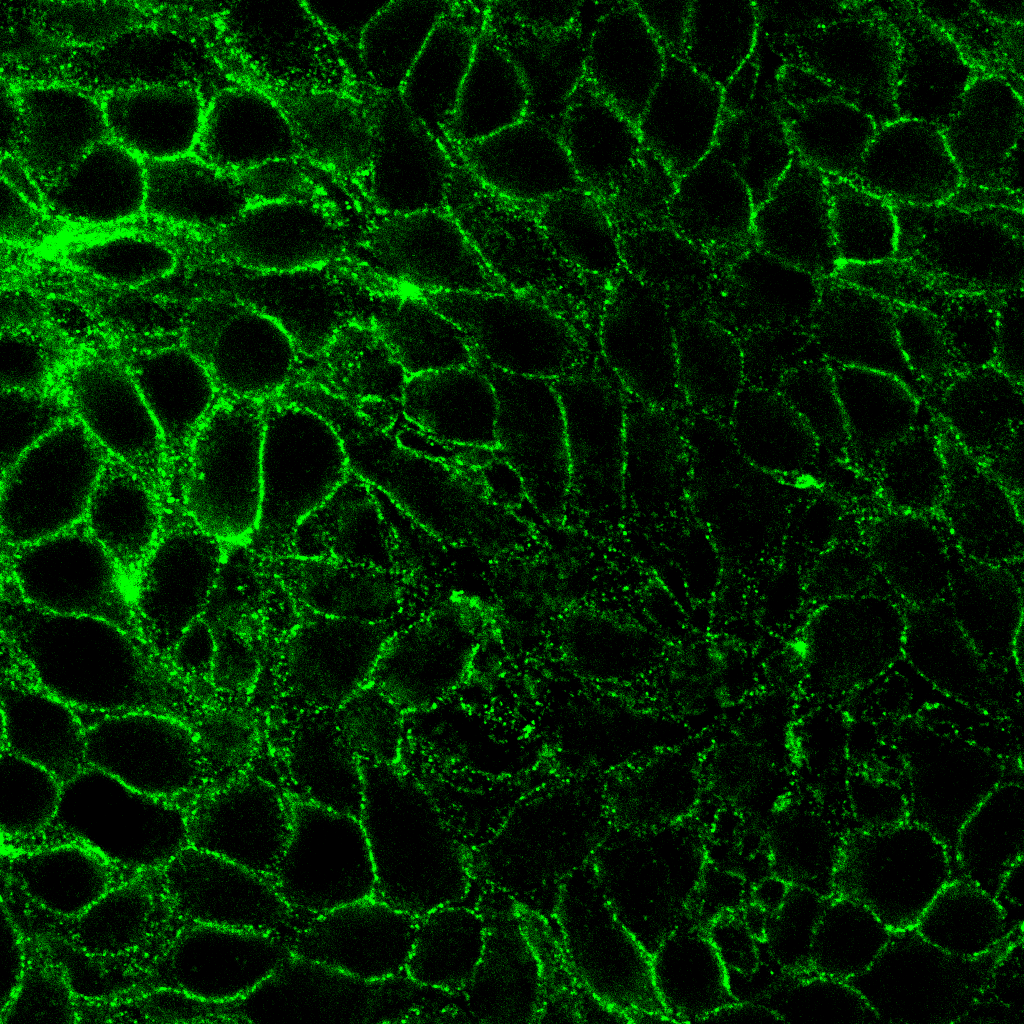

Supplement: Supplementary file 5 [file SupplementaryFile5.zip › 免疫荧光/6.25/E-Cad/488-E-BRE.tif.frames/488-E-BRE_C002T001.tif]

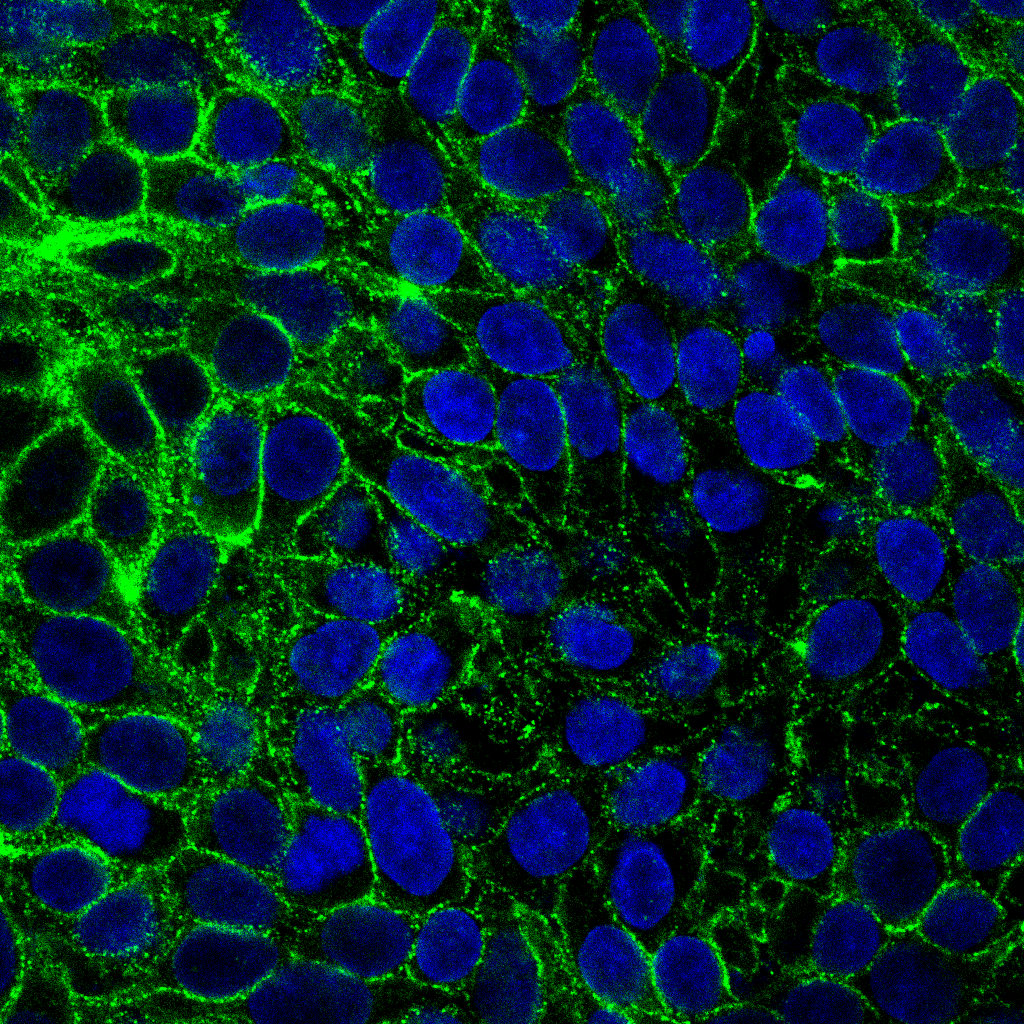

Supplement: Supplementary file 5 [file SupplementaryFile5.zip › 免疫荧光/6.25/E-Cad/488-E-BRE.tif.frames/488-E-BRE_T001.tif]

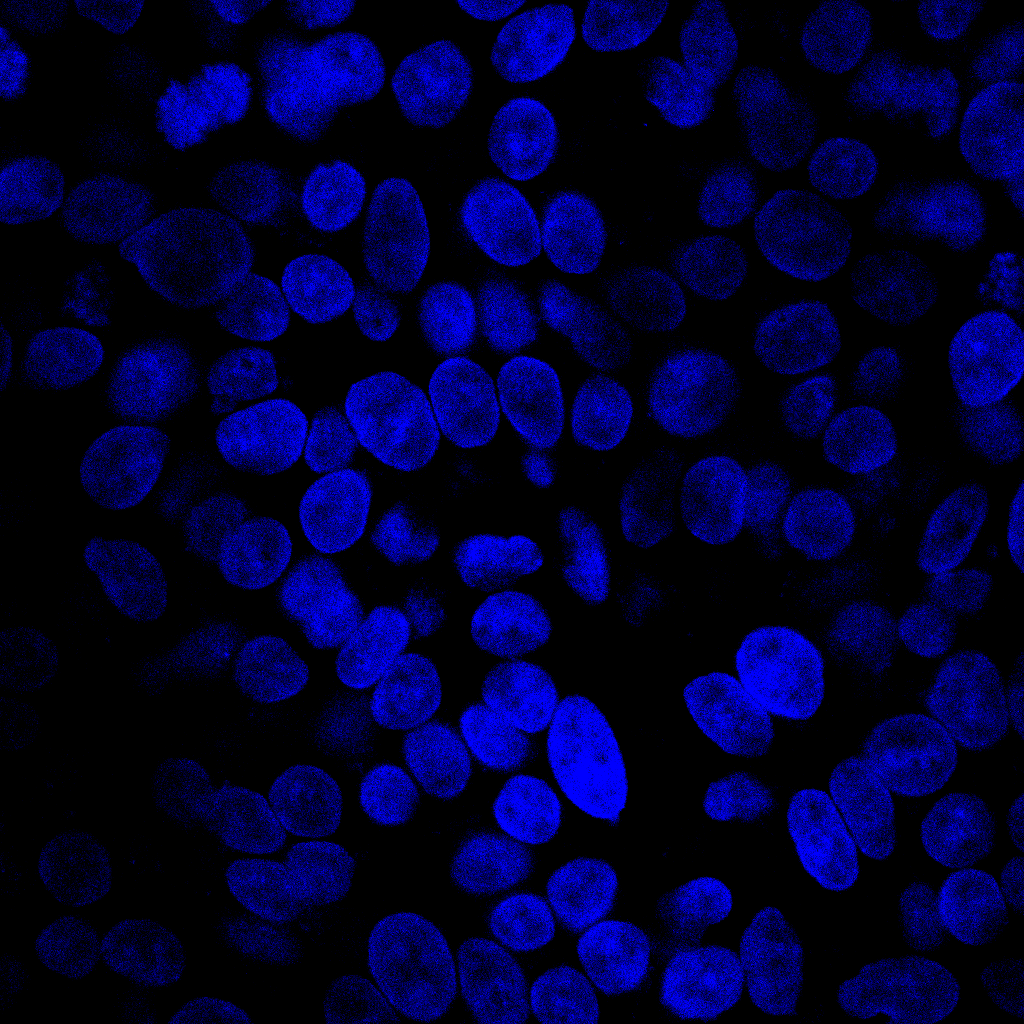

Supplement: Supplementary file 5 [file SupplementaryFile5.zip › 免疫荧光/6.25/E-Cad/488-E-BRE_0001.tif.frames/488-E-BRE_0001_C001T001.tif]

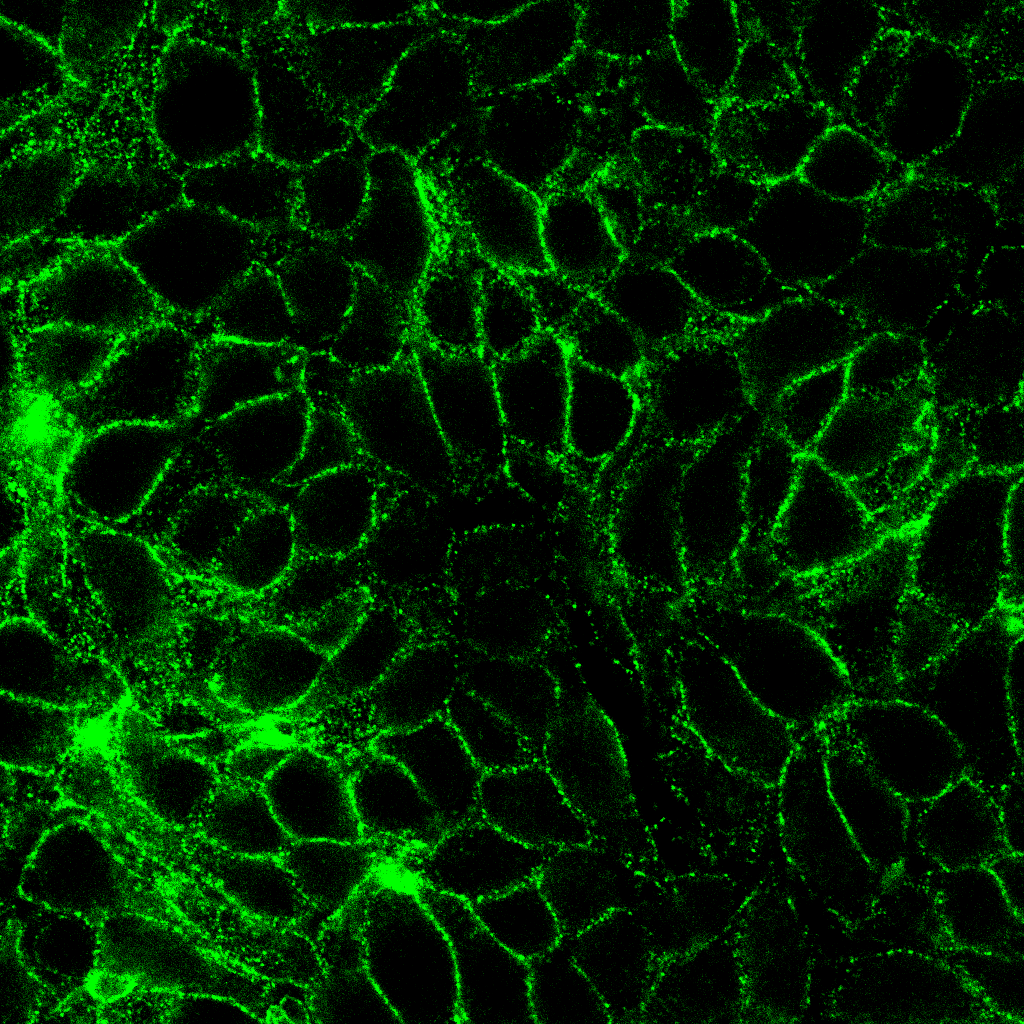

Supplement: Supplementary file 5 [file SupplementaryFile5.zip › 免疫荧光/6.25/E-Cad/488-E-BRE_0001.tif.frames/488-E-BRE_0001_C002T001.tif]

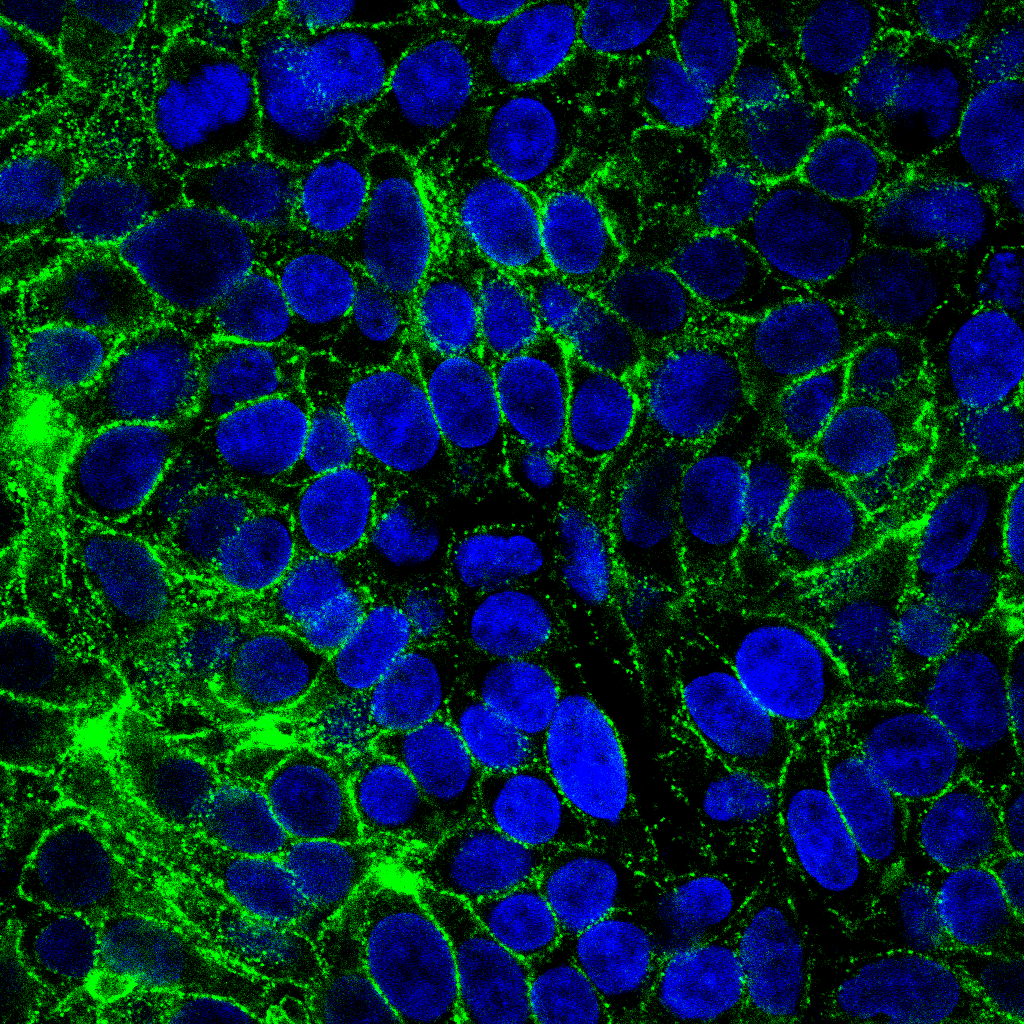

Supplement: Supplementary file 5 [file SupplementaryFile5.zip › 免疫荧光/6.25/E-Cad/488-E-BRE_0001.tif.frames/488-E-BRE_0001_T001.tif]

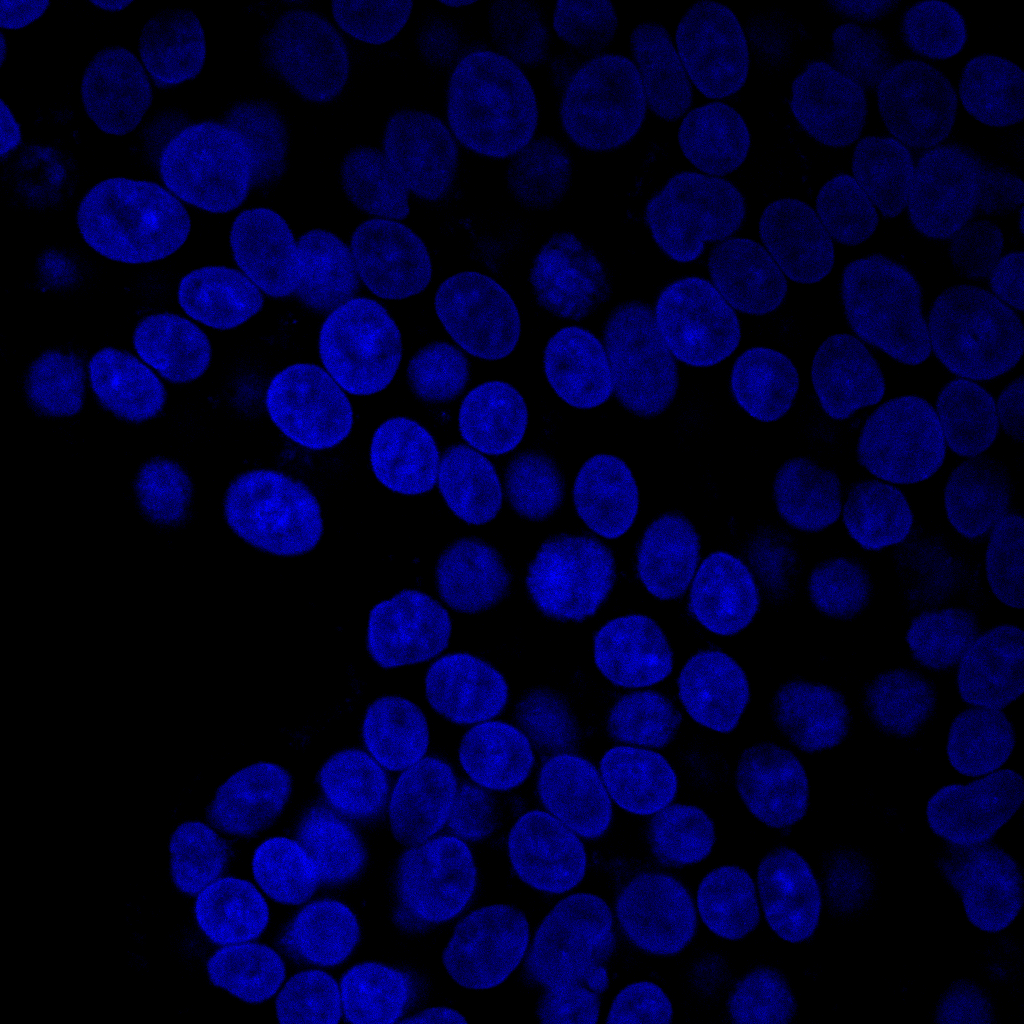

Supplement: Supplementary file 5 [file SupplementaryFile5.zip › 免疫荧光/6.25/E-Cad/488-E-BRE_0005.tif.frames/488-E-BRE_0005_C001T001.tif]

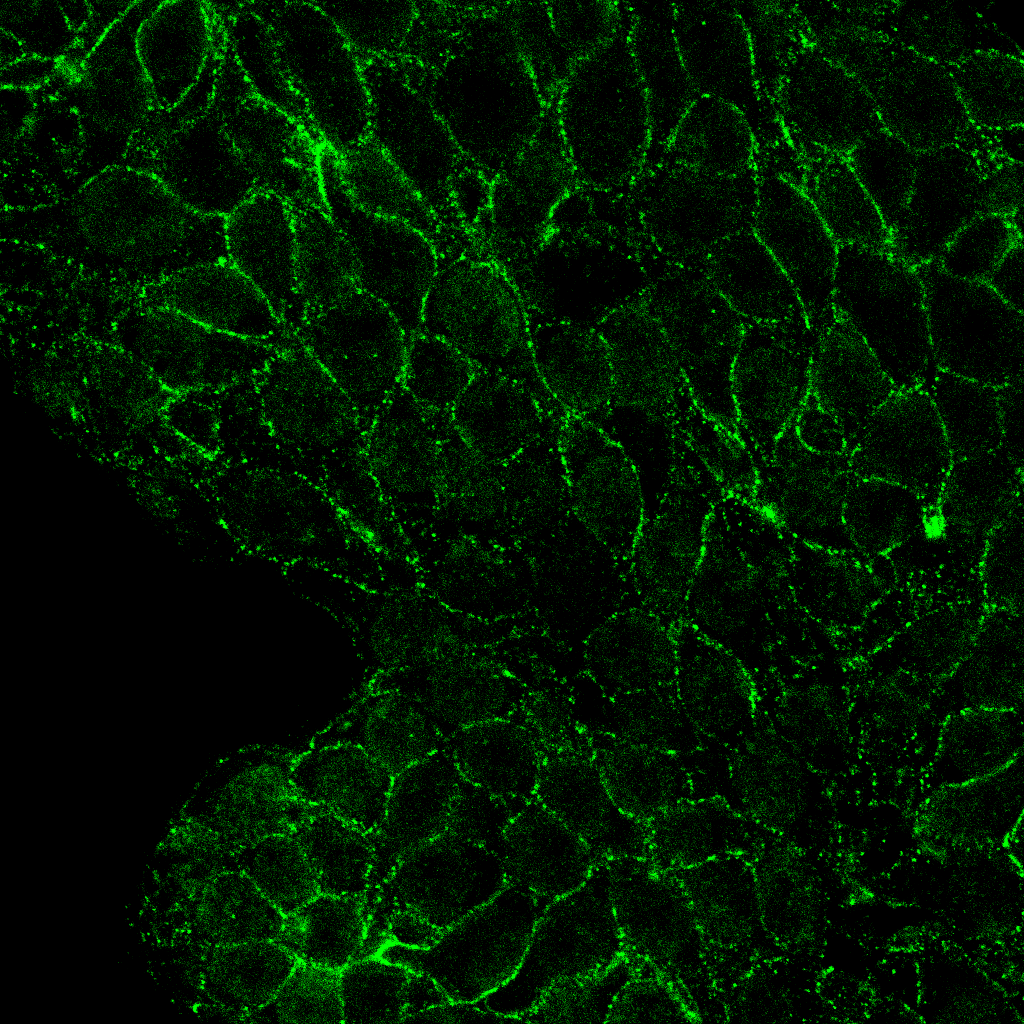

Supplement: Supplementary file 5 [file SupplementaryFile5.zip › 免疫荧光/6.25/E-Cad/488-E-BRE_0005.tif.frames/488-E-BRE_0005_C002T001.tif]

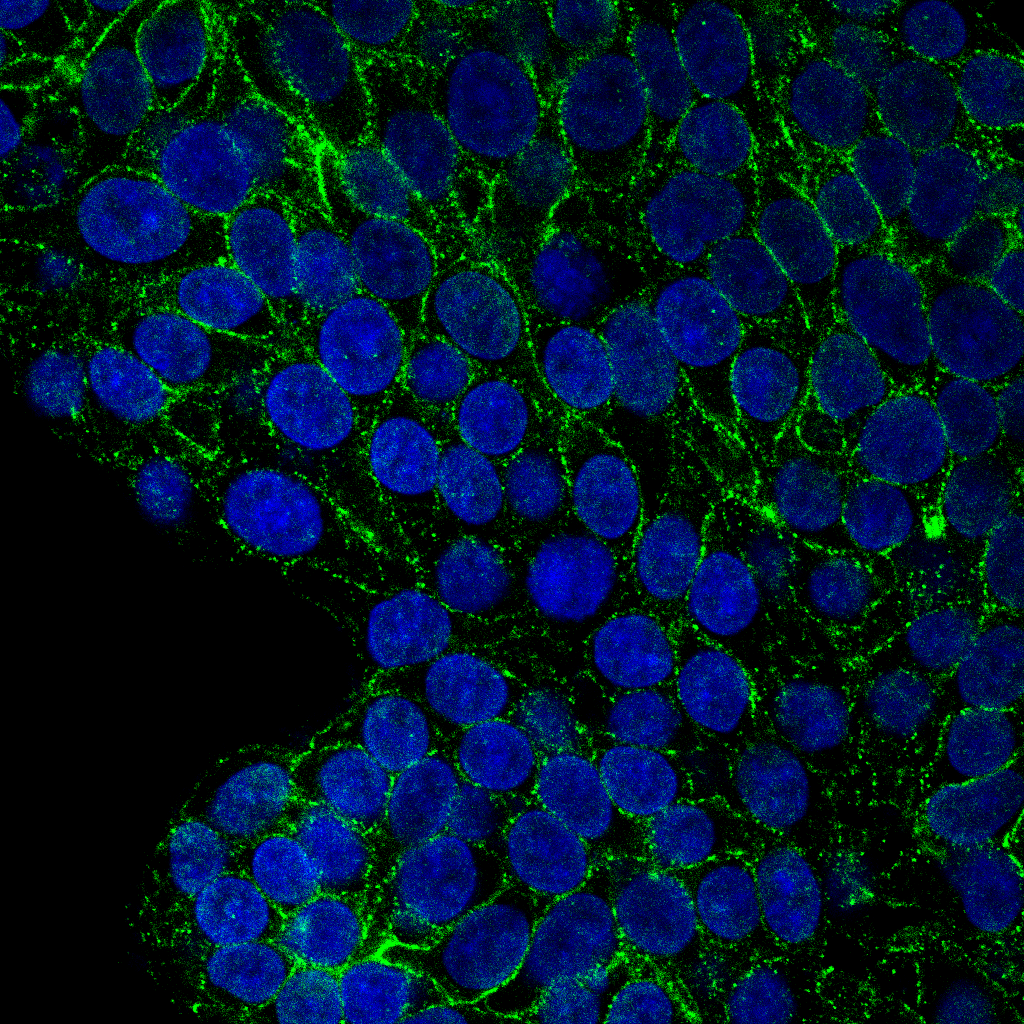

Supplement: Supplementary file 5 [file SupplementaryFile5.zip › 免疫荧光/6.25/E-Cad/488-E-BRE_0005.tif.frames/488-E-BRE_0005_T001.tif]

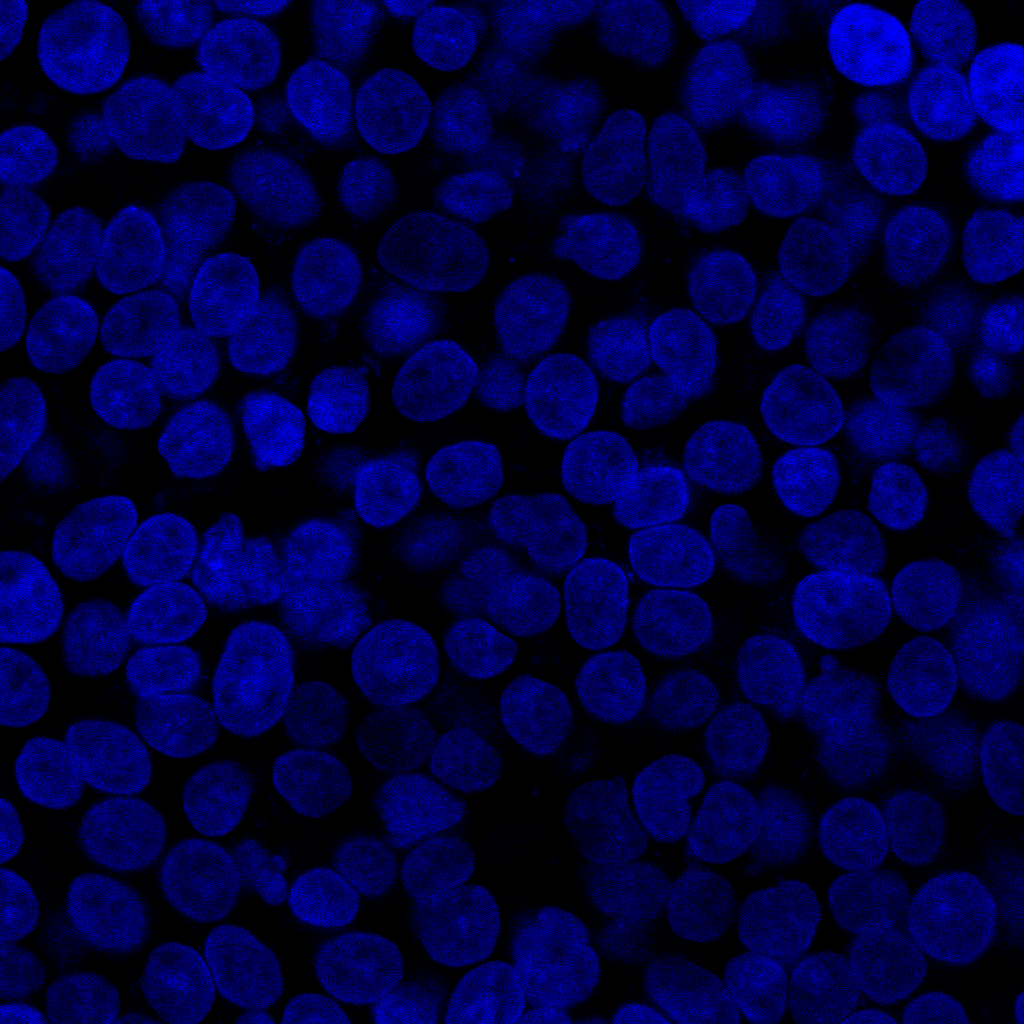

Supplement: Supplementary file 5 [file SupplementaryFile5.zip › 免疫荧光/6.25/E-Cad/488-E-BRE_0006.tif.frames/488-E-BRE_0006_C001T001.tif]

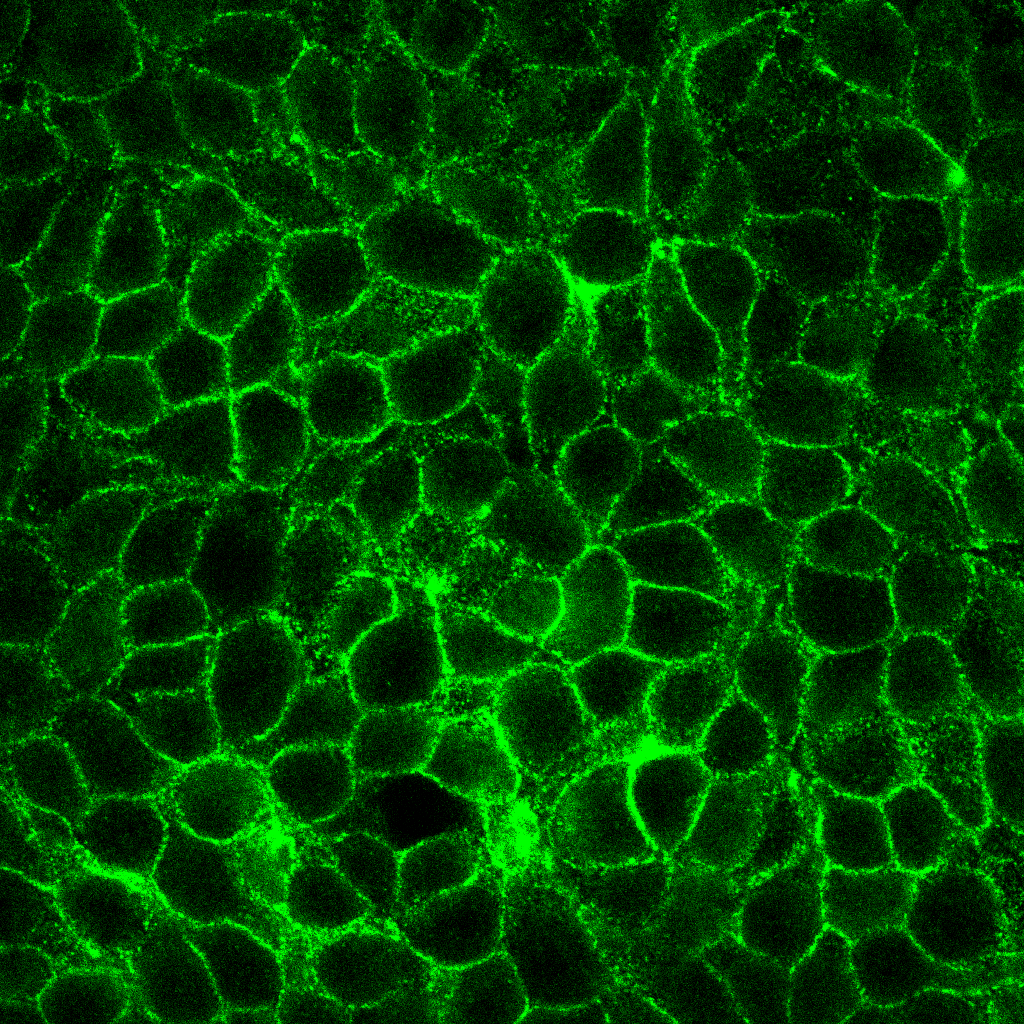

Supplement: Supplementary file 5 [file SupplementaryFile5.zip › 免疫荧光/6.25/E-Cad/488-E-BRE_0006.tif.frames/488-E-BRE_0006_C002T001.tif]

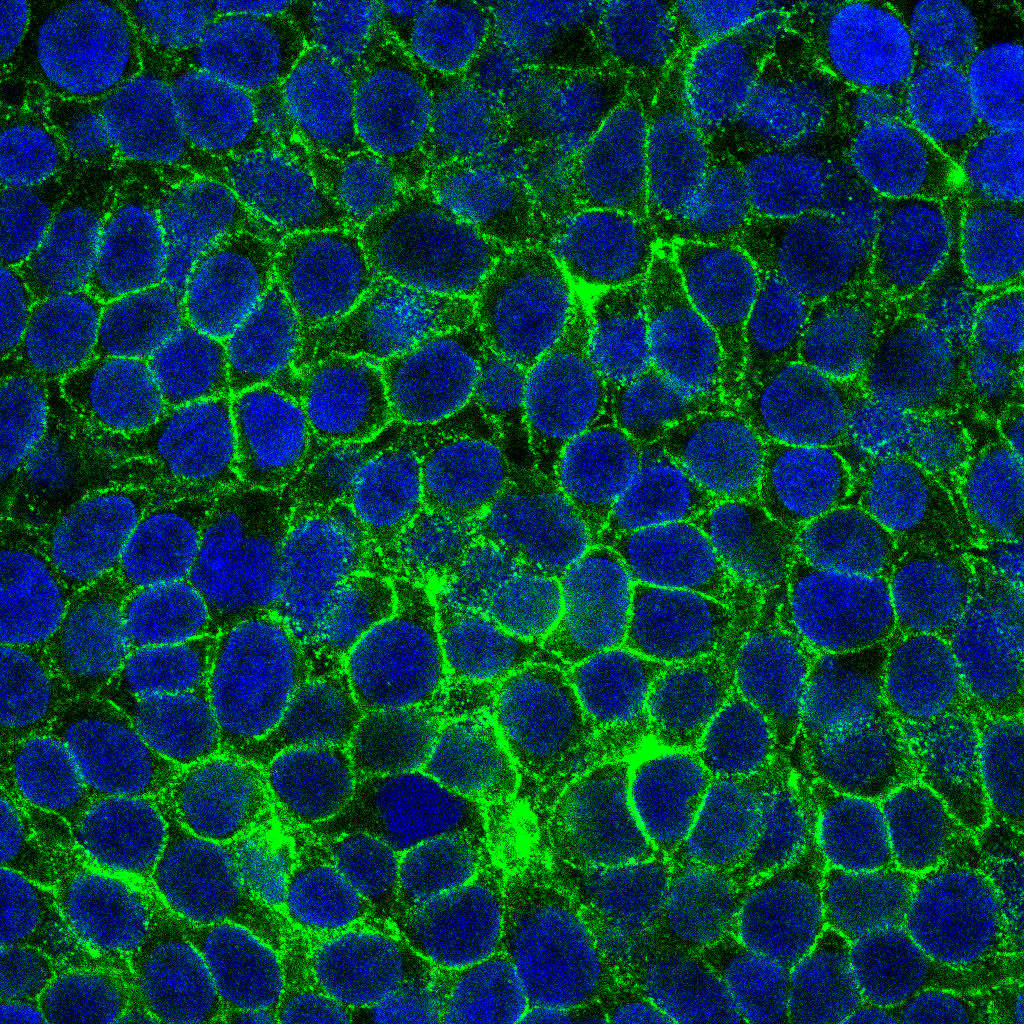

Supplement: Supplementary file 5 [file SupplementaryFile5.zip › 免疫荧光/6.25/E-Cad/488-E-BRE_0006.tif.frames/488-E-BRE_0006_T001.tif]

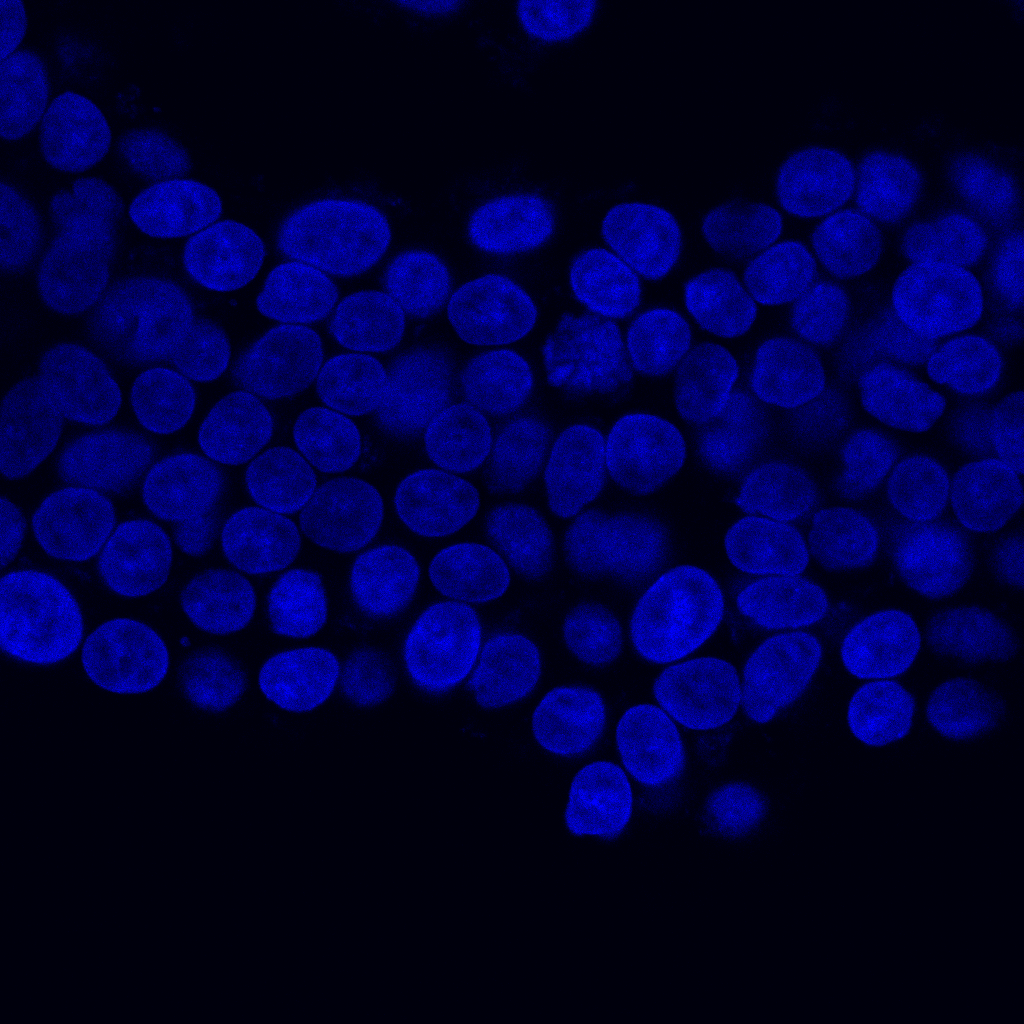

Supplement: Supplementary file 5 [file SupplementaryFile5.zip › 免疫荧光/6.25/E-Cad/488-E-BRE_0007.tif.frames/488-E-BRE_0007_C001T001.tif]

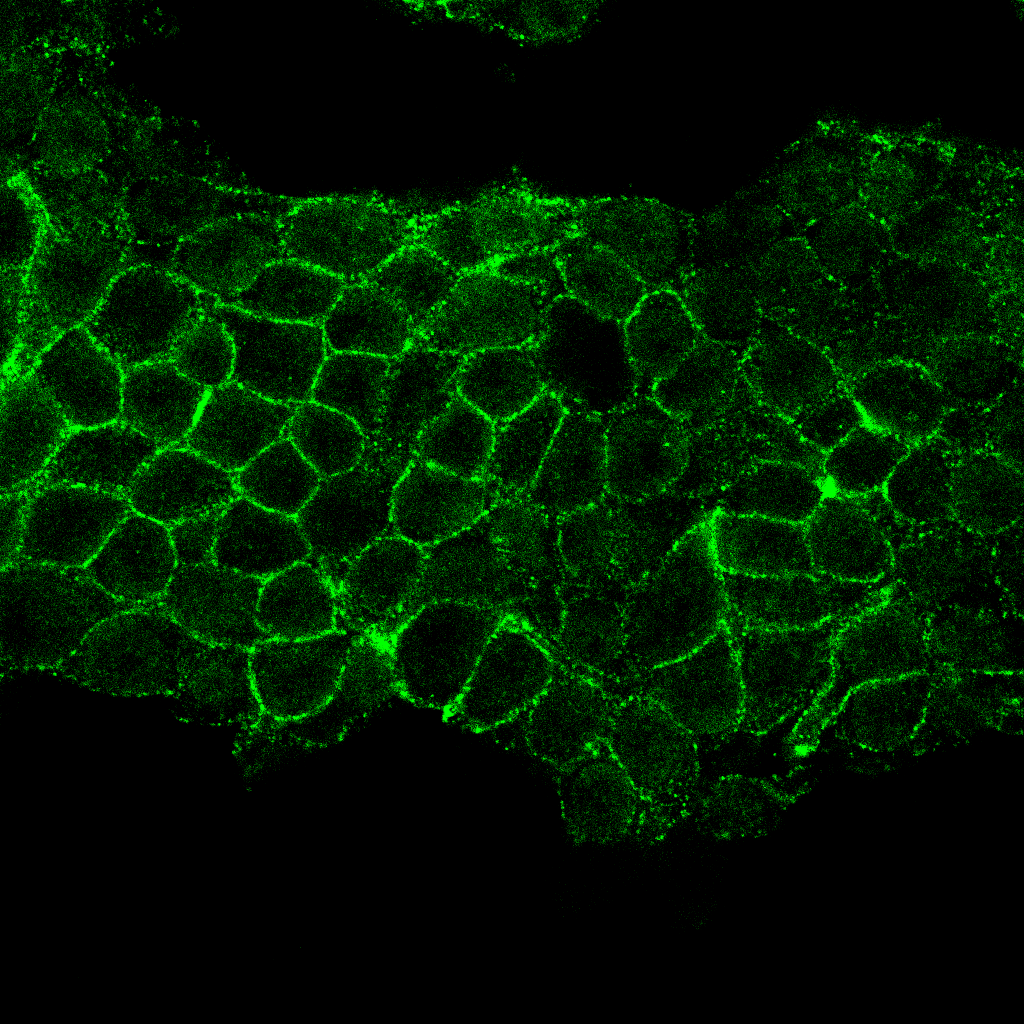

Supplement: Supplementary file 5 [file SupplementaryFile5.zip › 免疫荧光/6.25/E-Cad/488-E-BRE_0007.tif.frames/488-E-BRE_0007_C002T001.tif]

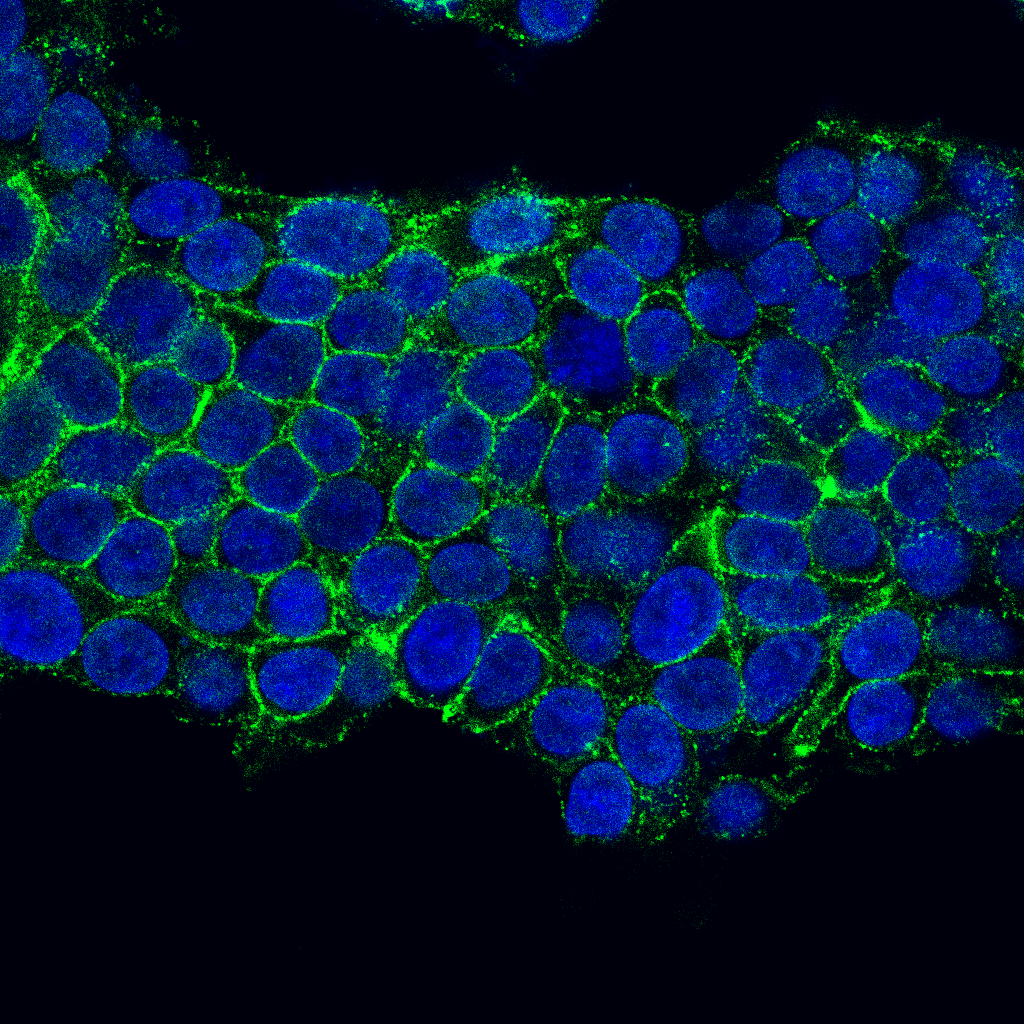

Supplement: Supplementary file 5 [file SupplementaryFile5.zip › 免疫荧光/6.25/E-Cad/488-E-BRE_0007.tif.frames/488-E-BRE_0007_T001.tif]

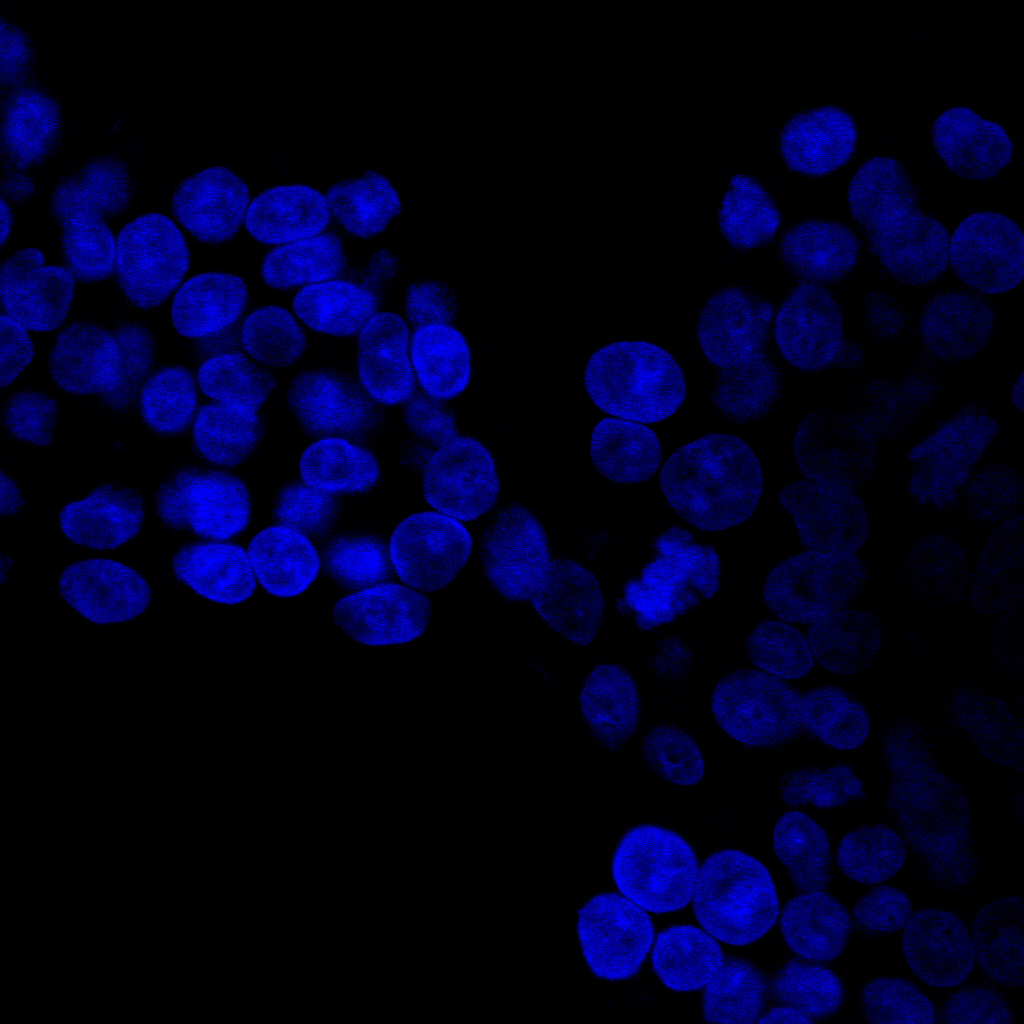

Supplement: Supplementary file 5 [file SupplementaryFile5.zip › 免疫荧光/6.25/E-Cad/488-E-BRE_0008.tif.frames/488-E-BRE_0008_C001T001.tif]

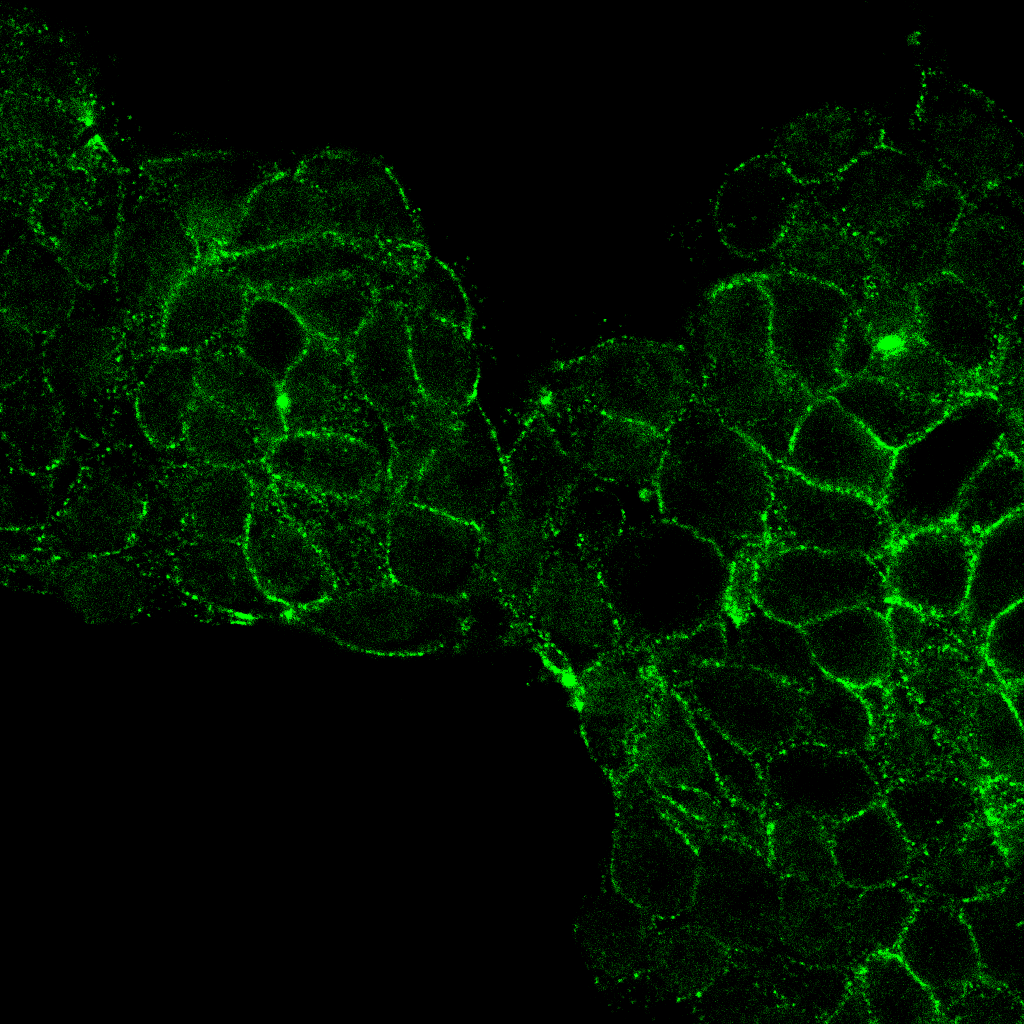

Supplement: Supplementary file 5 [file SupplementaryFile5.zip › 免疫荧光/6.25/E-Cad/488-E-BRE_0008.tif.frames/488-E-BRE_0008_C002T001.tif]

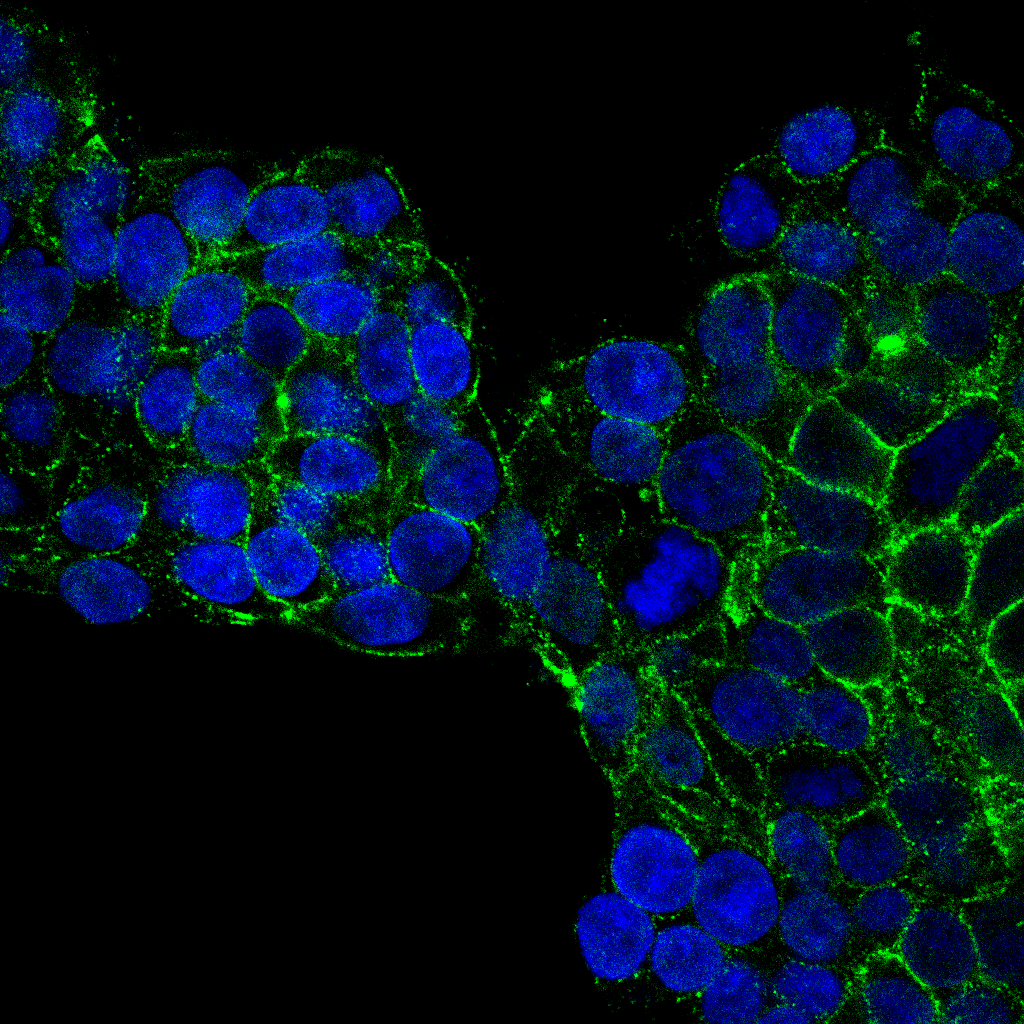

Supplement: Supplementary file 5 [file SupplementaryFile5.zip › 免疫荧光/6.25/E-Cad/488-E-BRE_0008.tif.frames/488-E-BRE_0008_T001.tif]

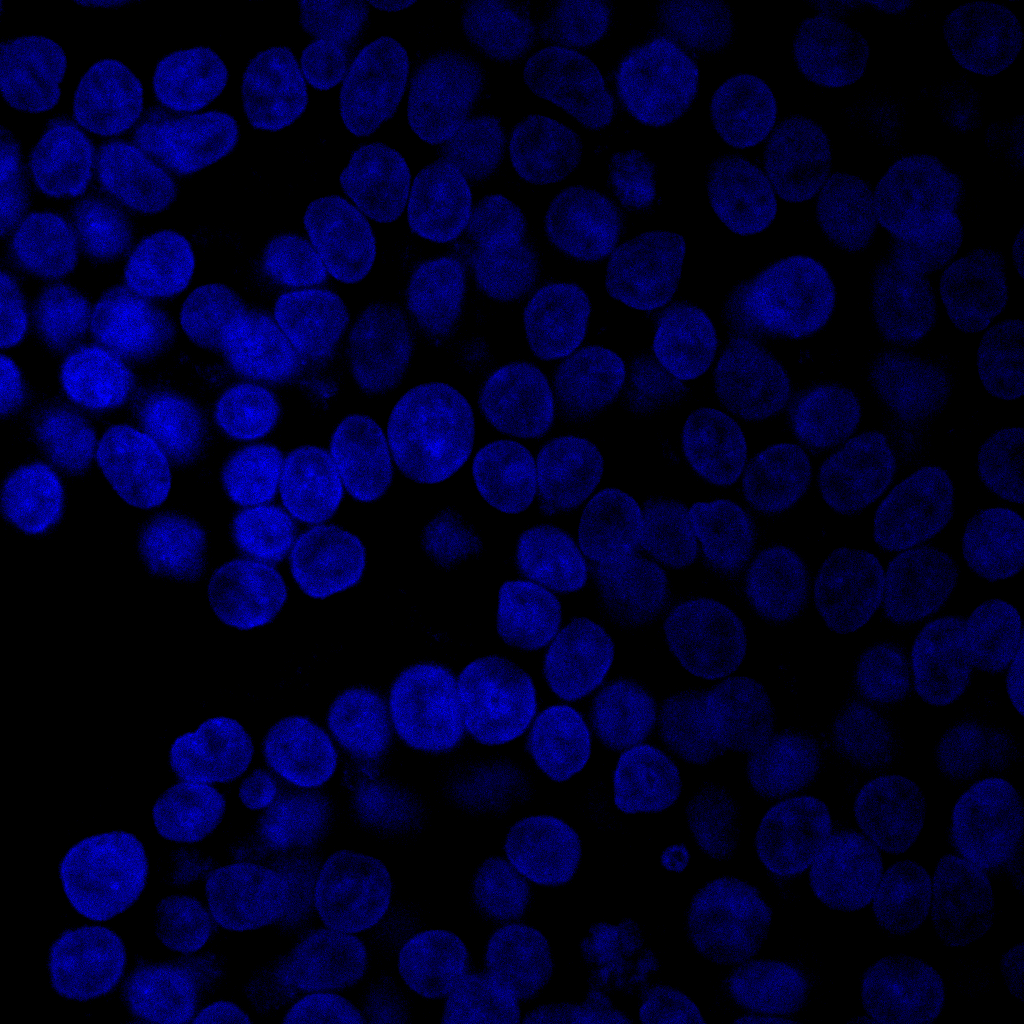

Supplement: Supplementary file 5 [file SupplementaryFile5.zip › 免疫荧光/6.25/E-Cad/488-E-BRE_0009.tif.frames/488-E-BRE_0009_C001T001.tif]

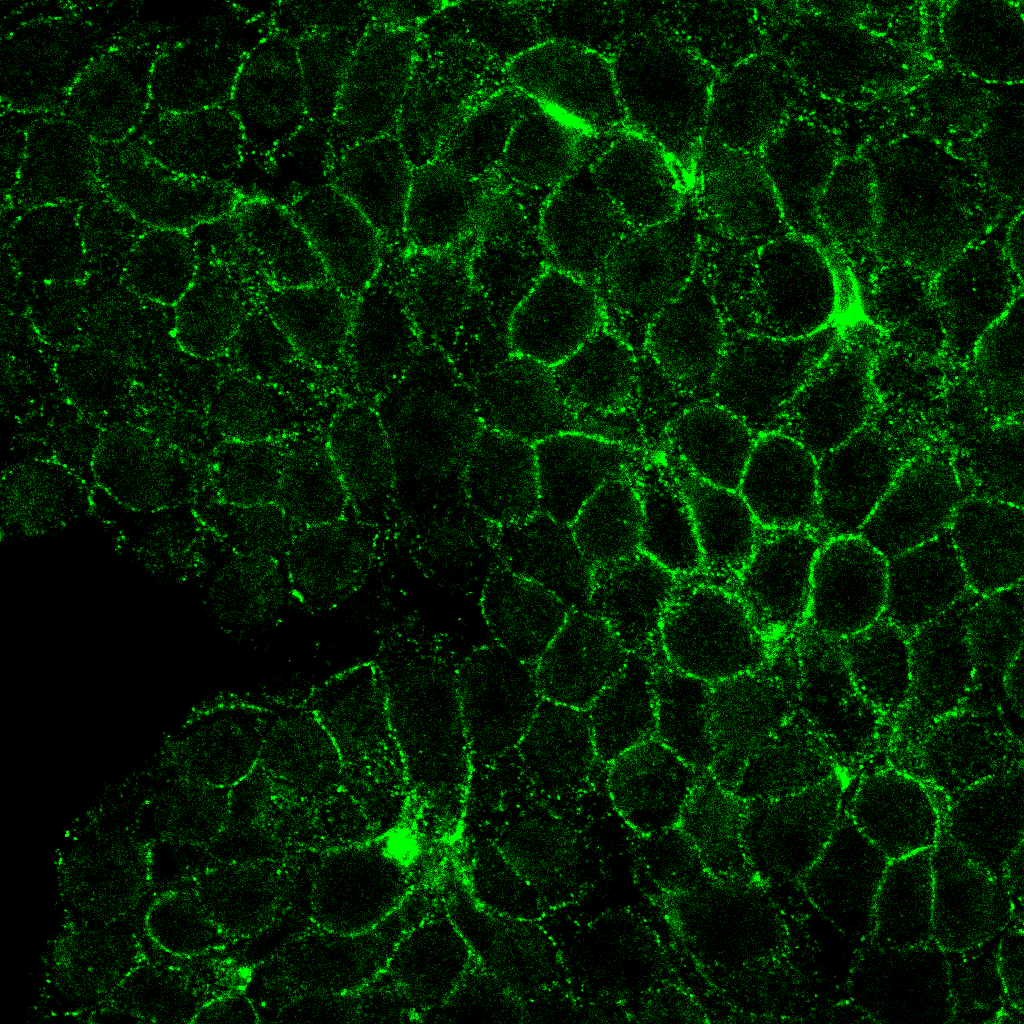

Supplement: Supplementary file 5 [file SupplementaryFile5.zip › 免疫荧光/6.25/E-Cad/488-E-BRE_0009.tif.frames/488-E-BRE_0009_C002T001.tif]

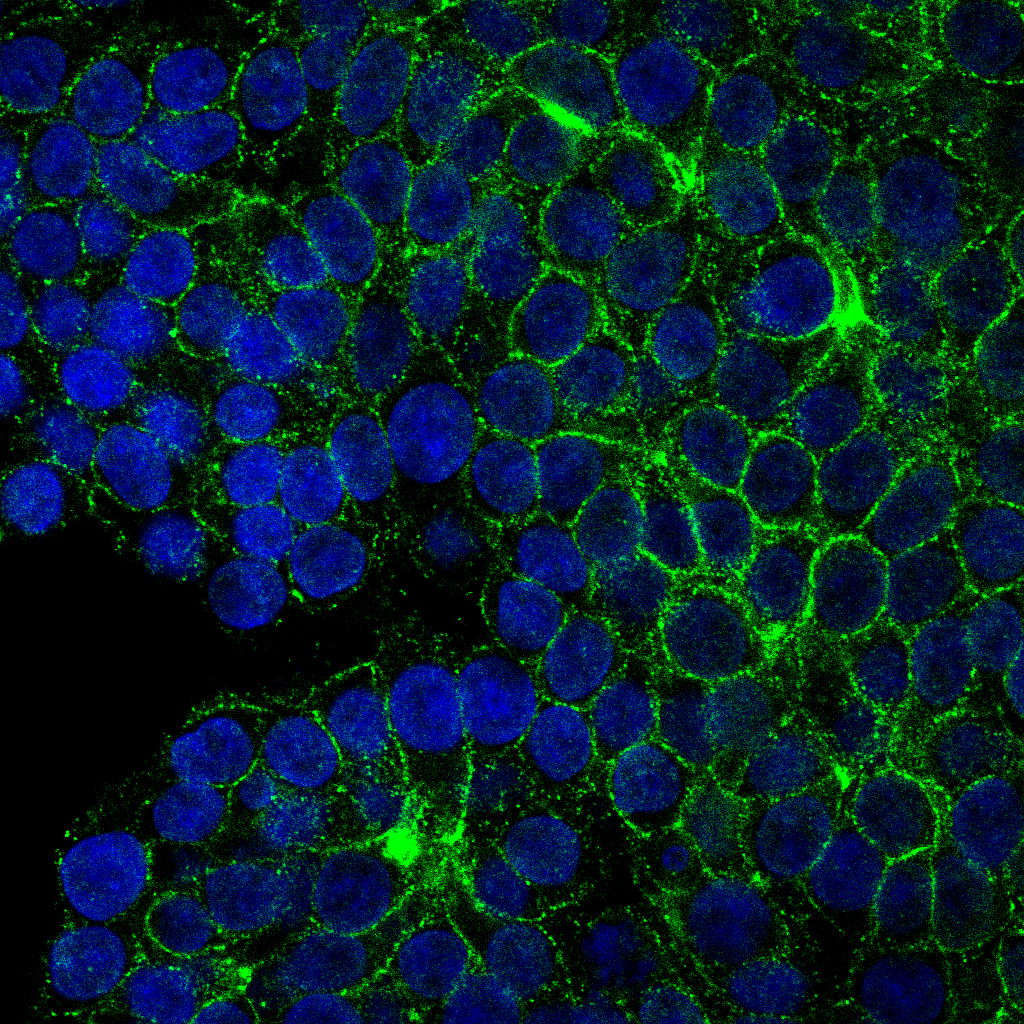

Supplement: Supplementary file 5 [file SupplementaryFile5.zip › 免疫荧光/6.25/E-Cad/488-E-BRE_0009.tif.frames/488-E-BRE_0009_T001.tif]

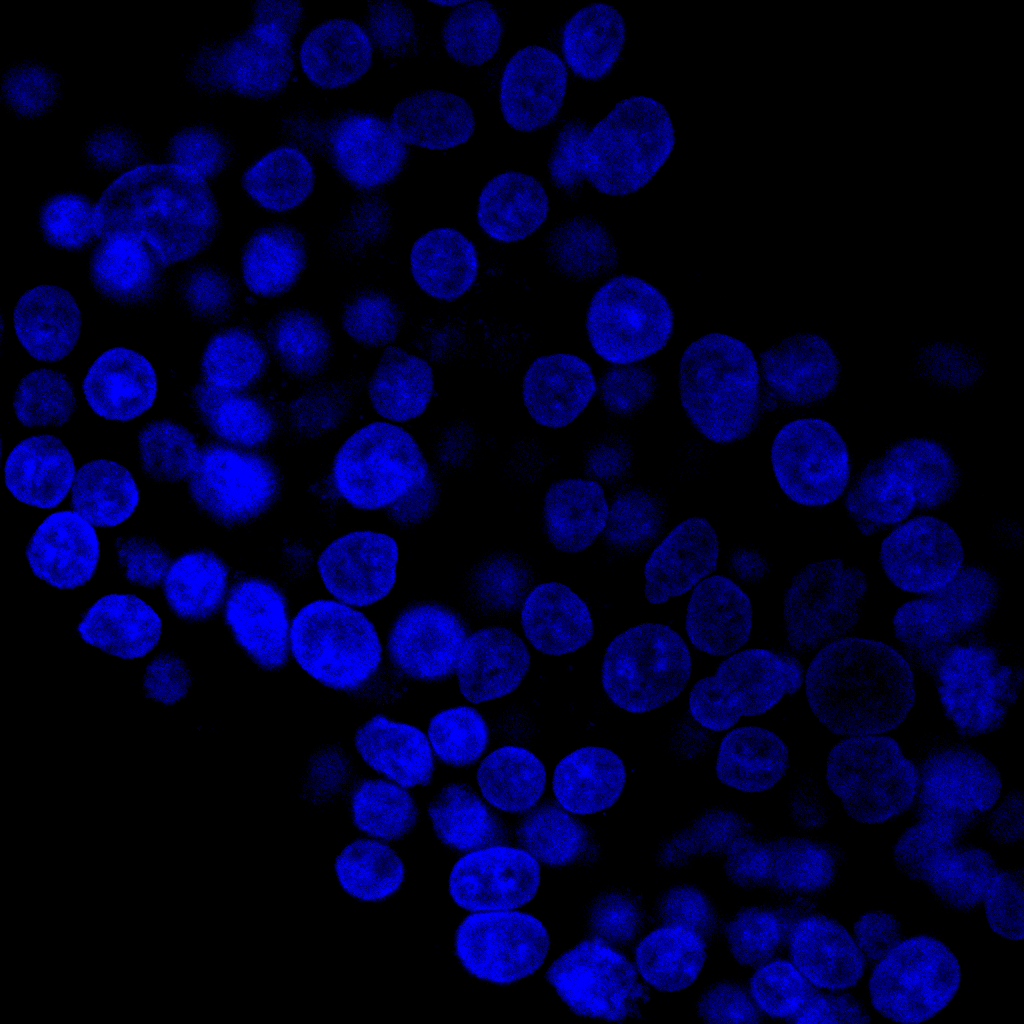

Supplement: Supplementary file 5 [file SupplementaryFile5.zip › 免疫荧光/6.25/E-Cad/488-E-NC.tif.frames/488-E-NC_C001T001.tif]

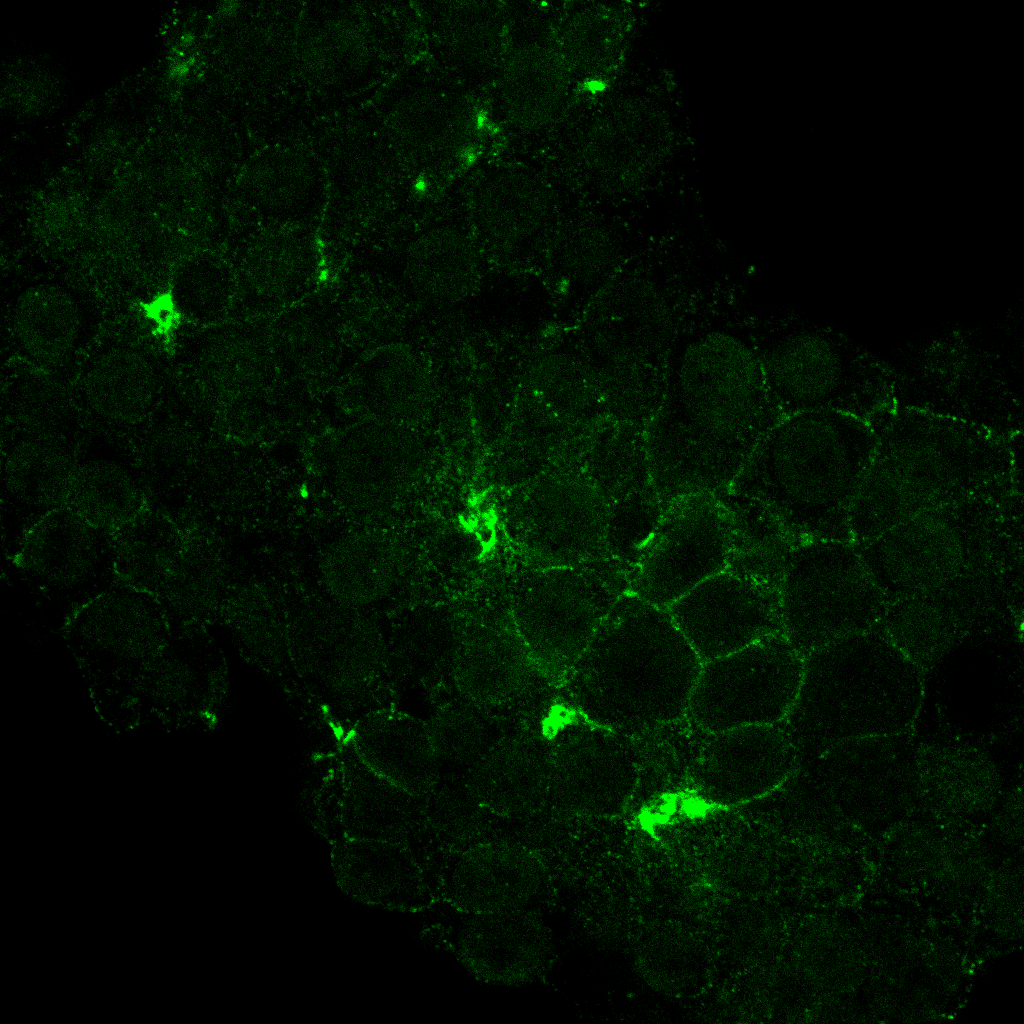

Supplement: Supplementary file 5 [file SupplementaryFile5.zip › 免疫荧光/6.25/E-Cad/488-E-NC.tif.frames/488-E-NC_C002T001.tif]

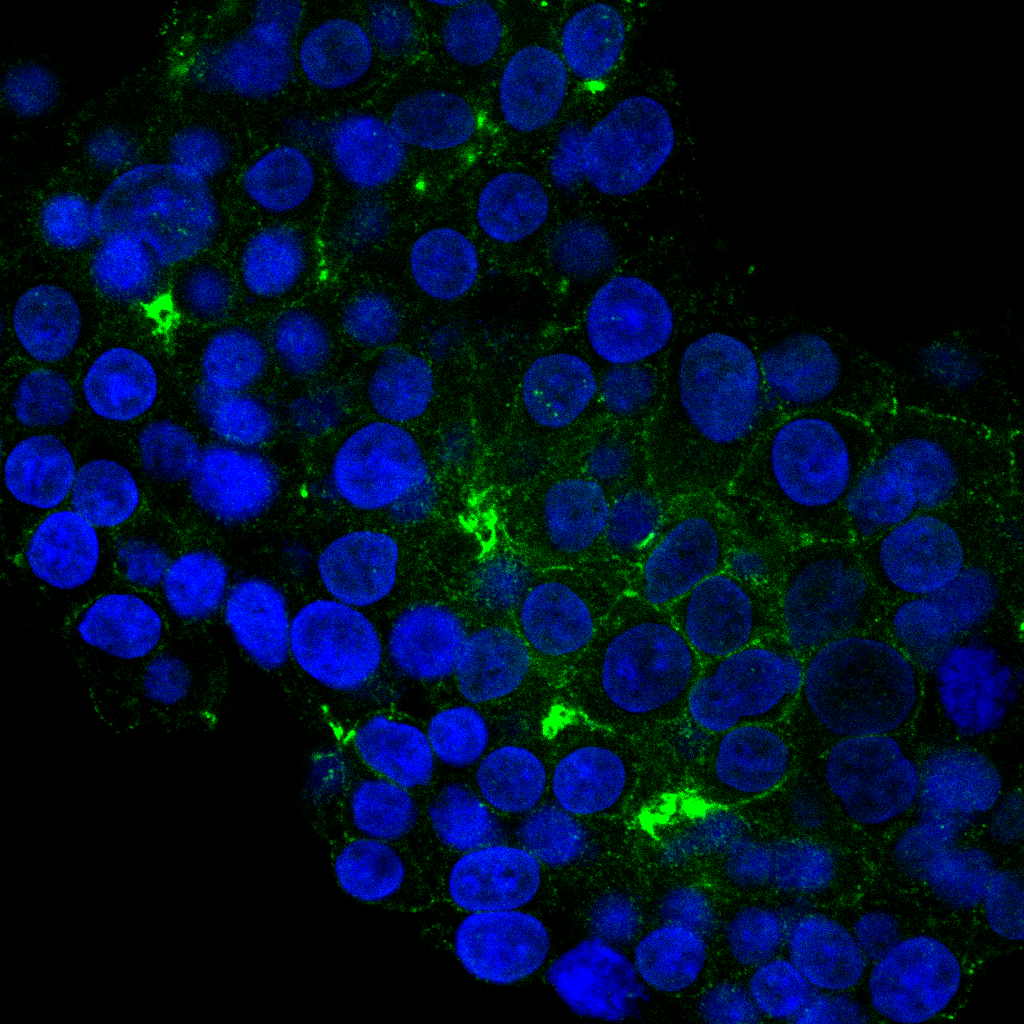

Supplement: Supplementary file 5 [file SupplementaryFile5.zip › 免疫荧光/6.25/E-Cad/488-E-NC.tif.frames/488-E-NC_T001.tif]

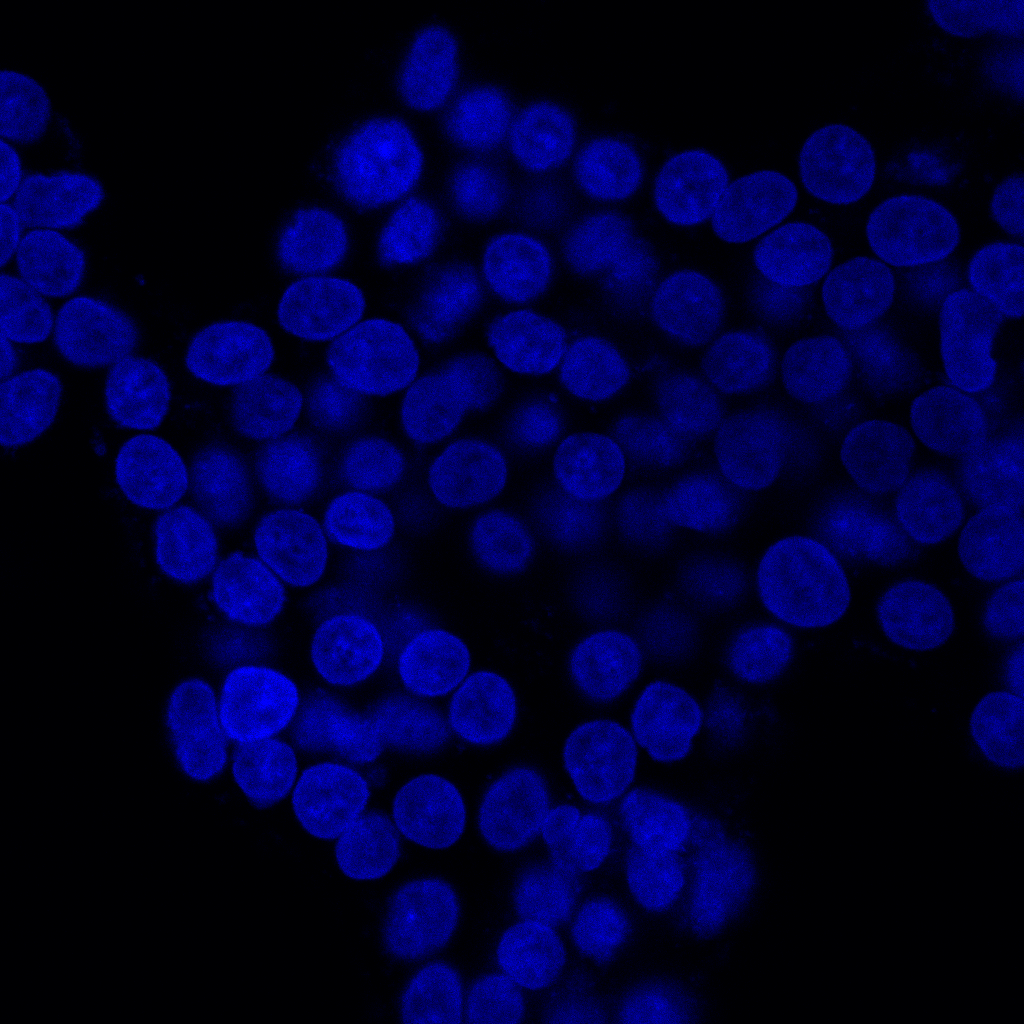

Supplement: Supplementary file 5 [file SupplementaryFile5.zip › 免疫荧光/6.25/E-Cad/488-E-NC_0003.tif.frames/488-E-NC_0003_C001T001.tif]

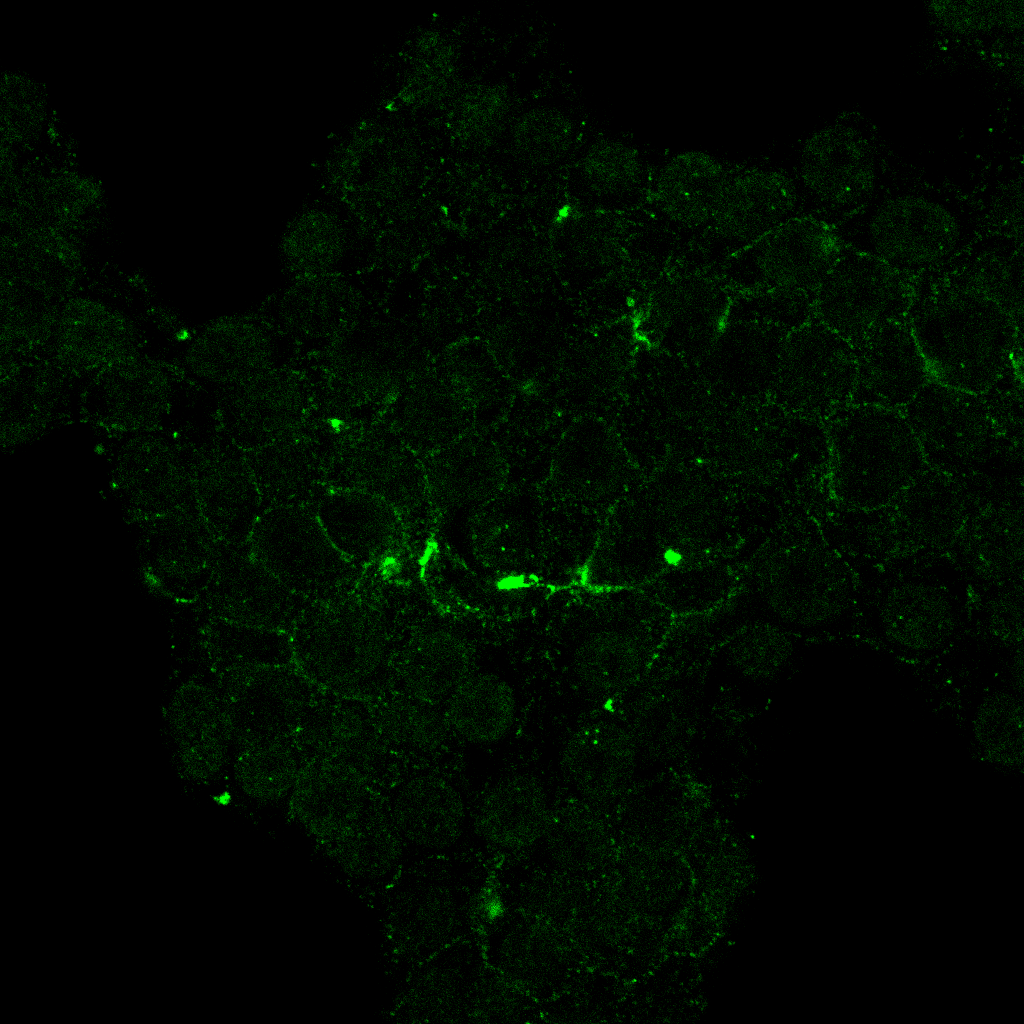

Supplement: Supplementary file 5 [file SupplementaryFile5.zip › 免疫荧光/6.25/E-Cad/488-E-NC_0003.tif.frames/488-E-NC_0003_C002T001.tif]

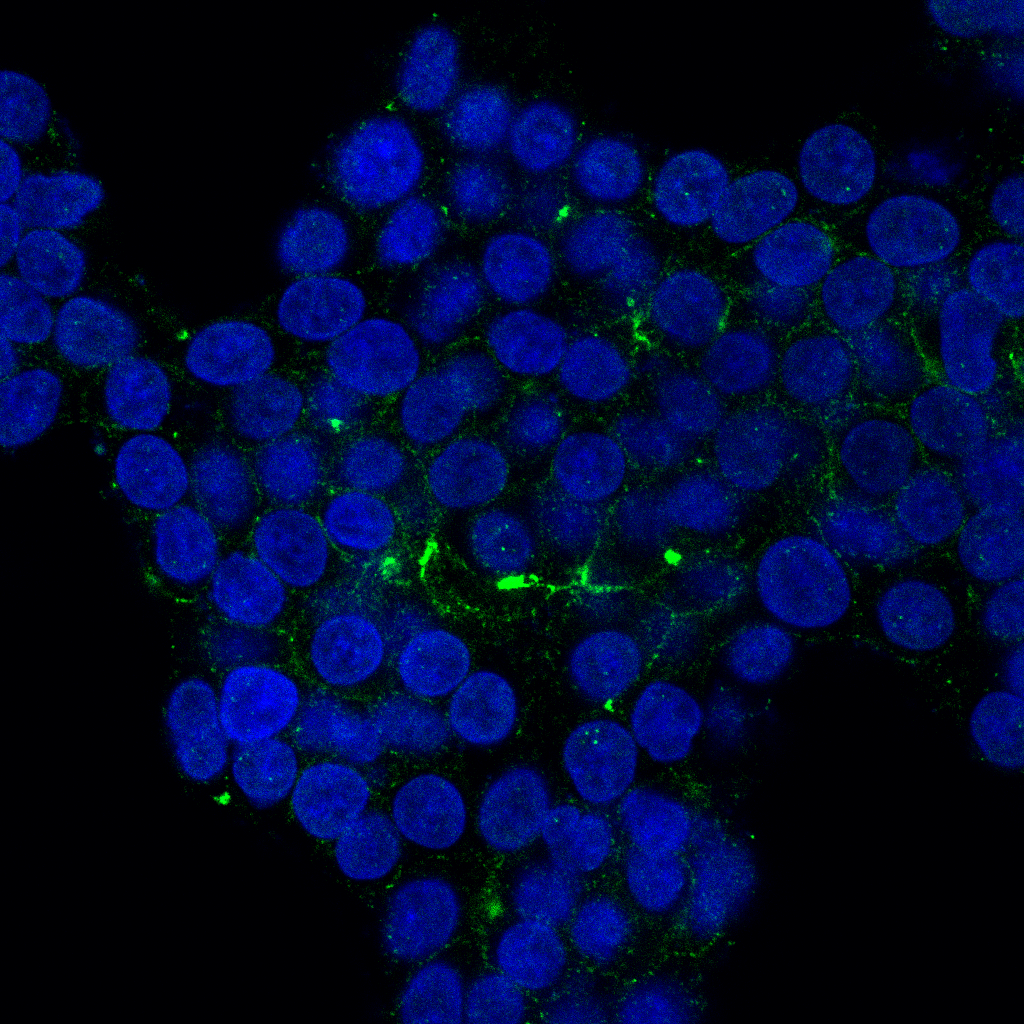

Supplement: Supplementary file 5 [file SupplementaryFile5.zip › 免疫荧光/6.25/E-Cad/488-E-NC_0003.tif.frames/488-E-NC_0003_T001.tif]

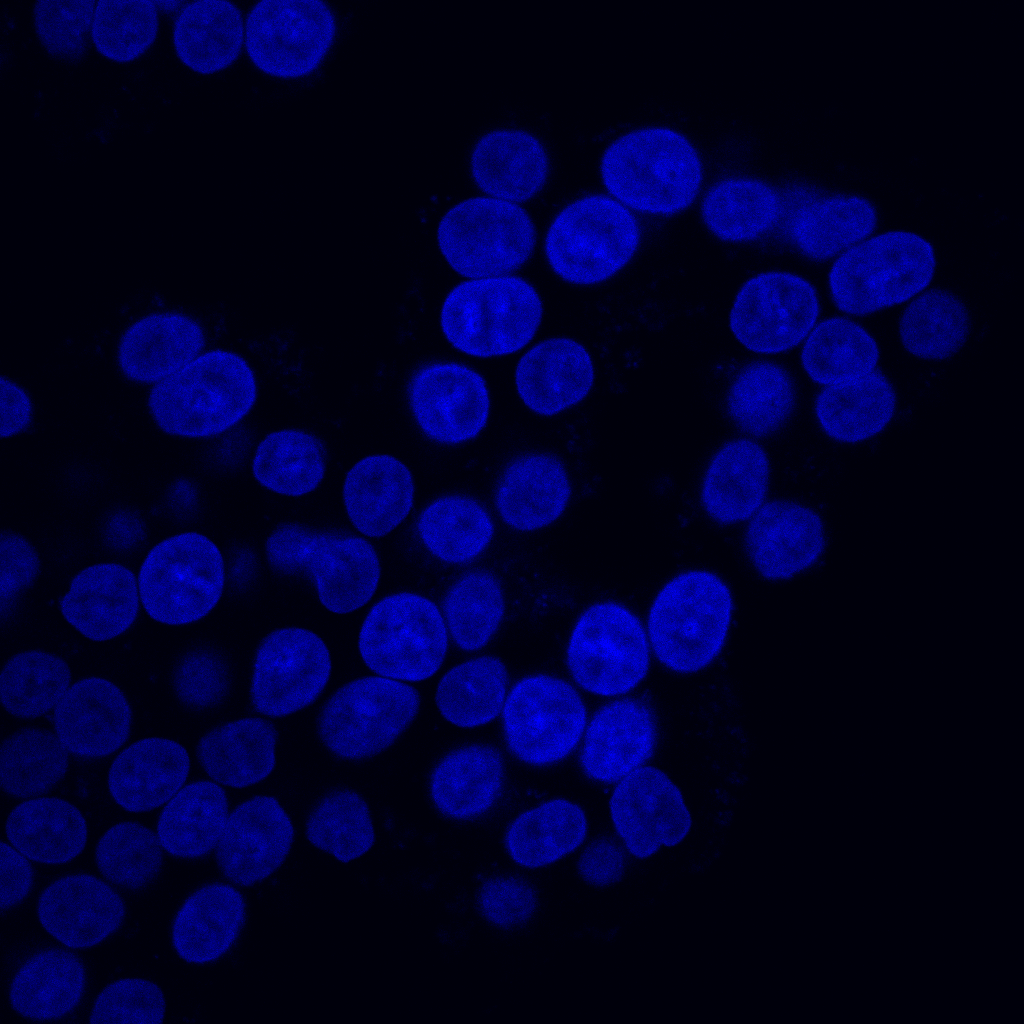

Supplement: Supplementary file 5 [file SupplementaryFile5.zip › 免疫荧光/6.25/E-Cad/488-E-NC_0004.tif.frames/488-E-NC_0004_C001T001.tif]

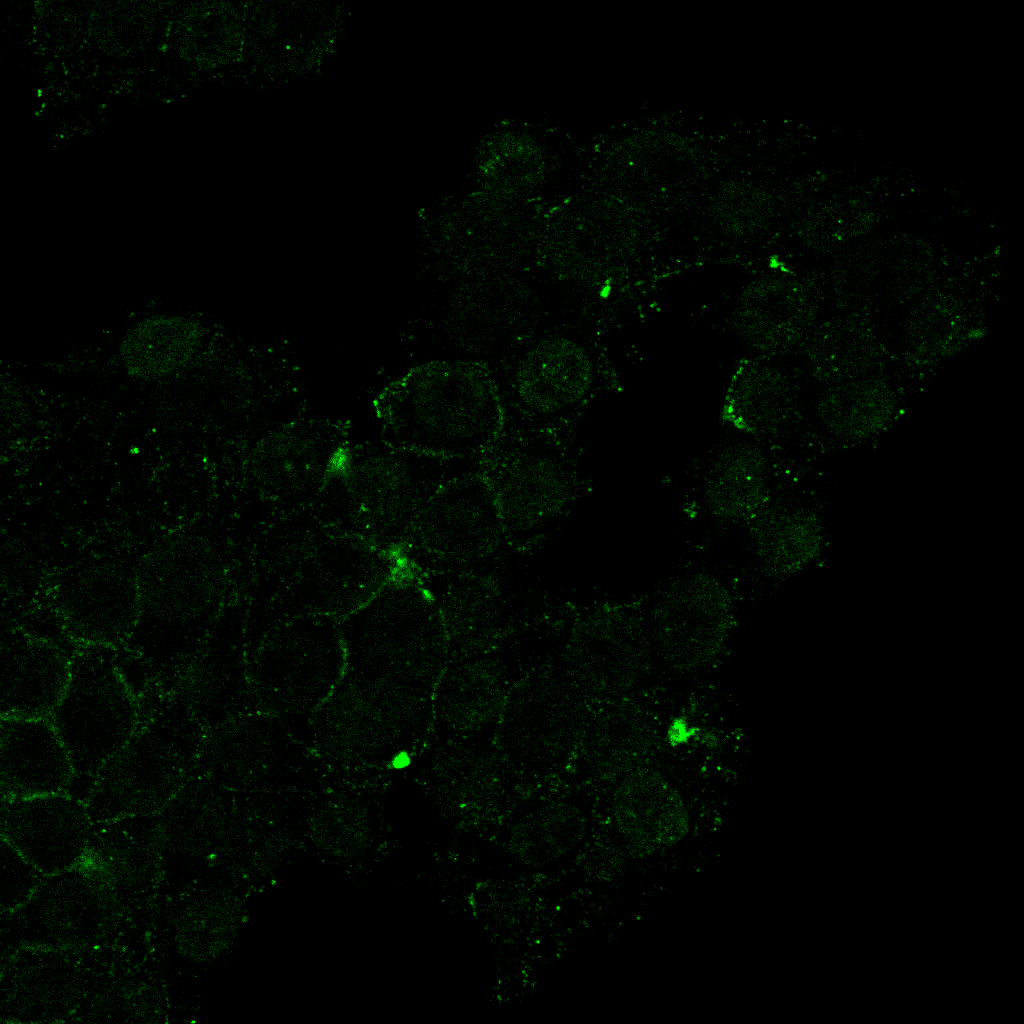

Supplement: Supplementary file 5 [file SupplementaryFile5.zip › 免疫荧光/6.25/E-Cad/488-E-NC_0004.tif.frames/488-E-NC_0004_C002T001.tif]

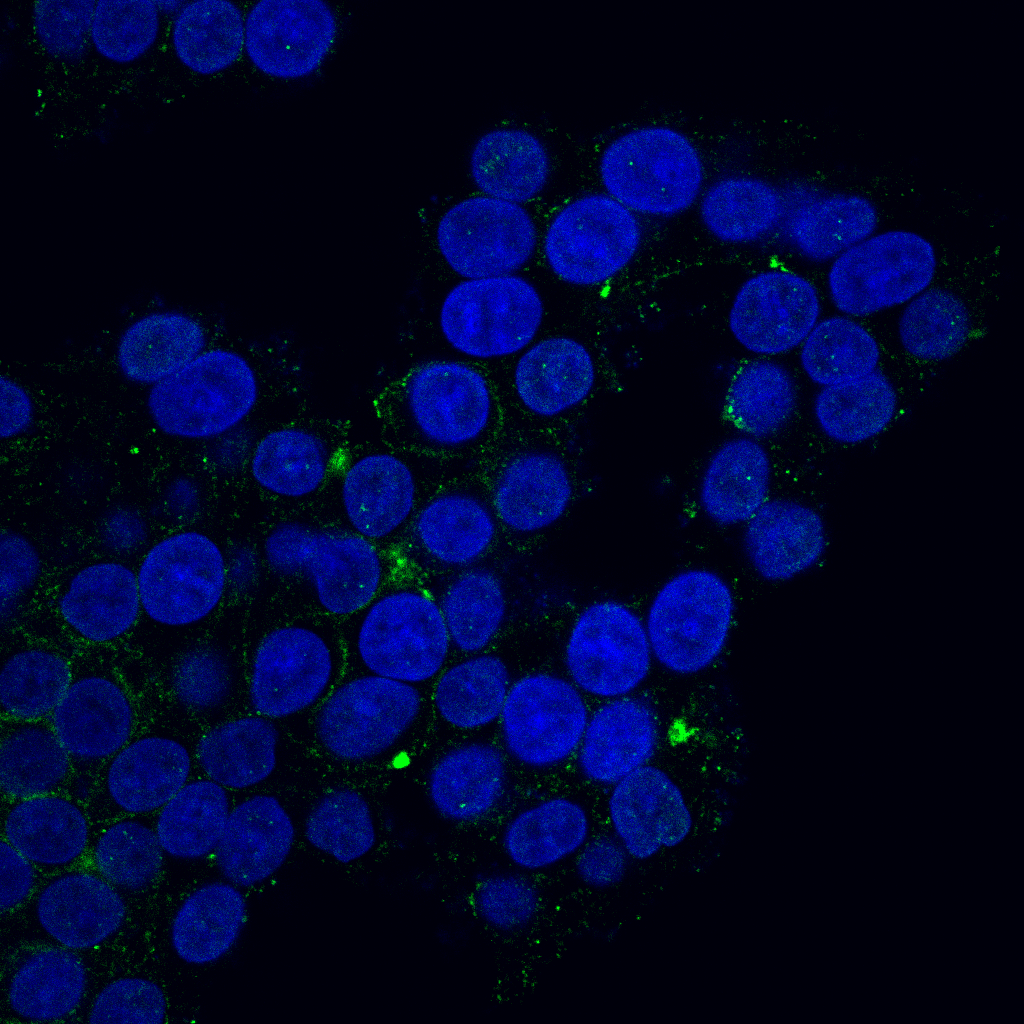

Supplement: Supplementary file 5 [file SupplementaryFile5.zip › 免疫荧光/6.25/E-Cad/488-E-NC_0004.tif.frames/488-E-NC_0004_T001.tif]

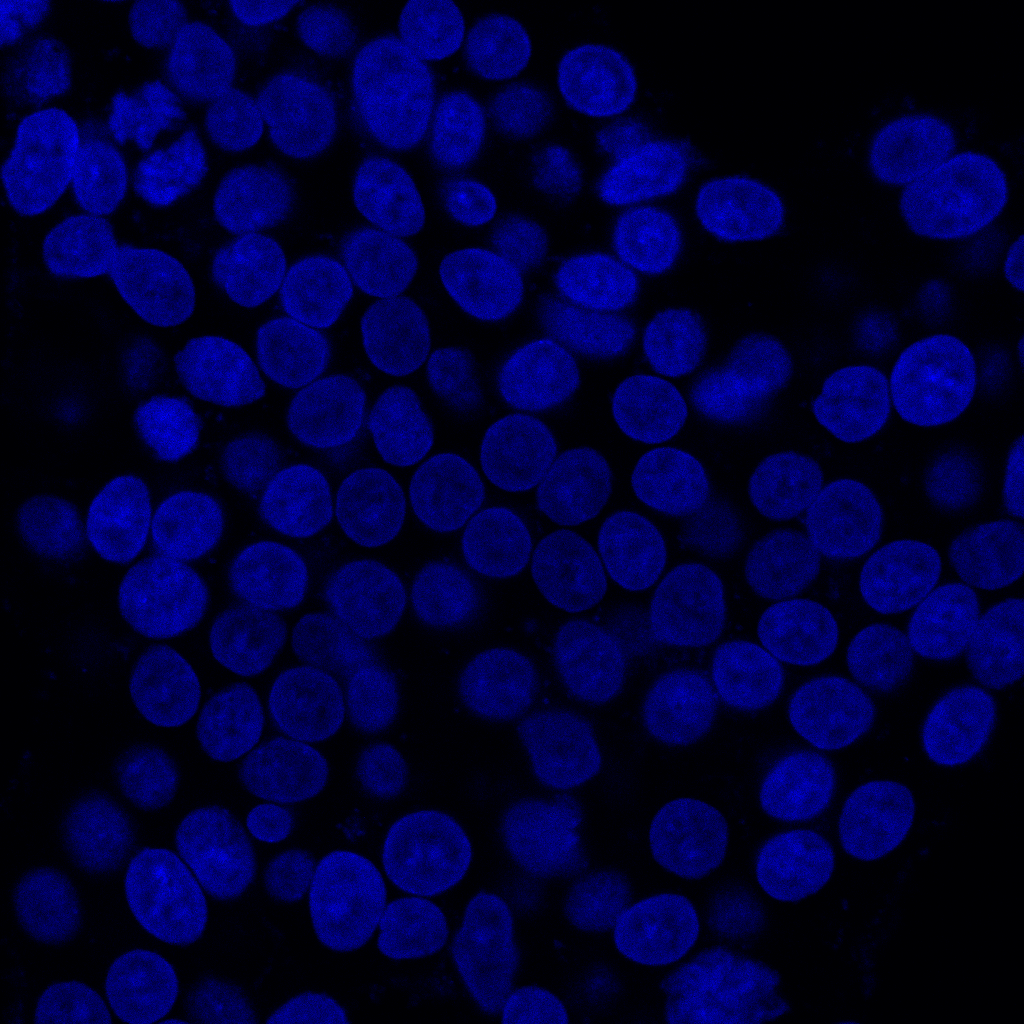

Supplement: Supplementary file 5 [file SupplementaryFile5.zip › 免疫荧光/6.25/E-Cad/488-E-NC_0005.tif.frames/488-E-NC_0005_C001T001.tif]

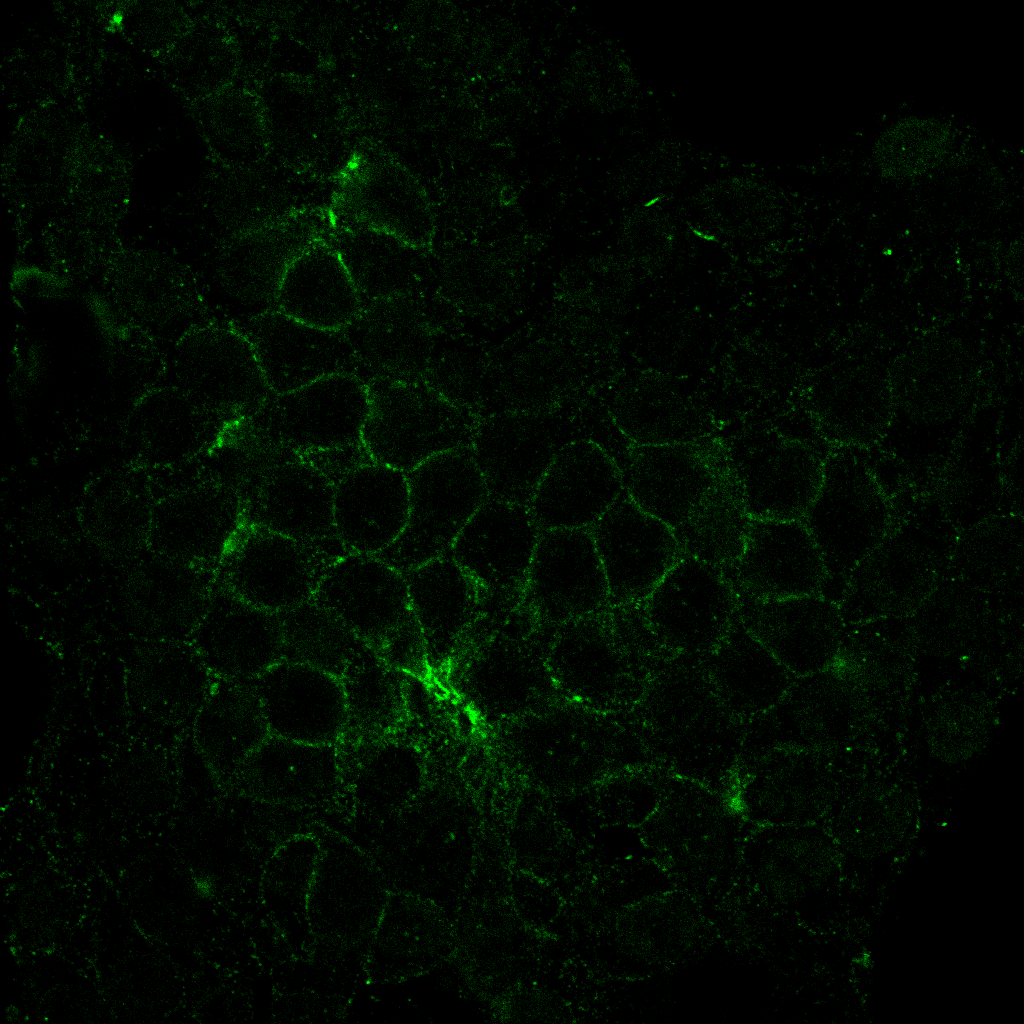

Supplement: Supplementary file 5 [file SupplementaryFile5.zip › 免疫荧光/6.25/E-Cad/488-E-NC_0005.tif.frames/488-E-NC_0005_C002T001.tif]

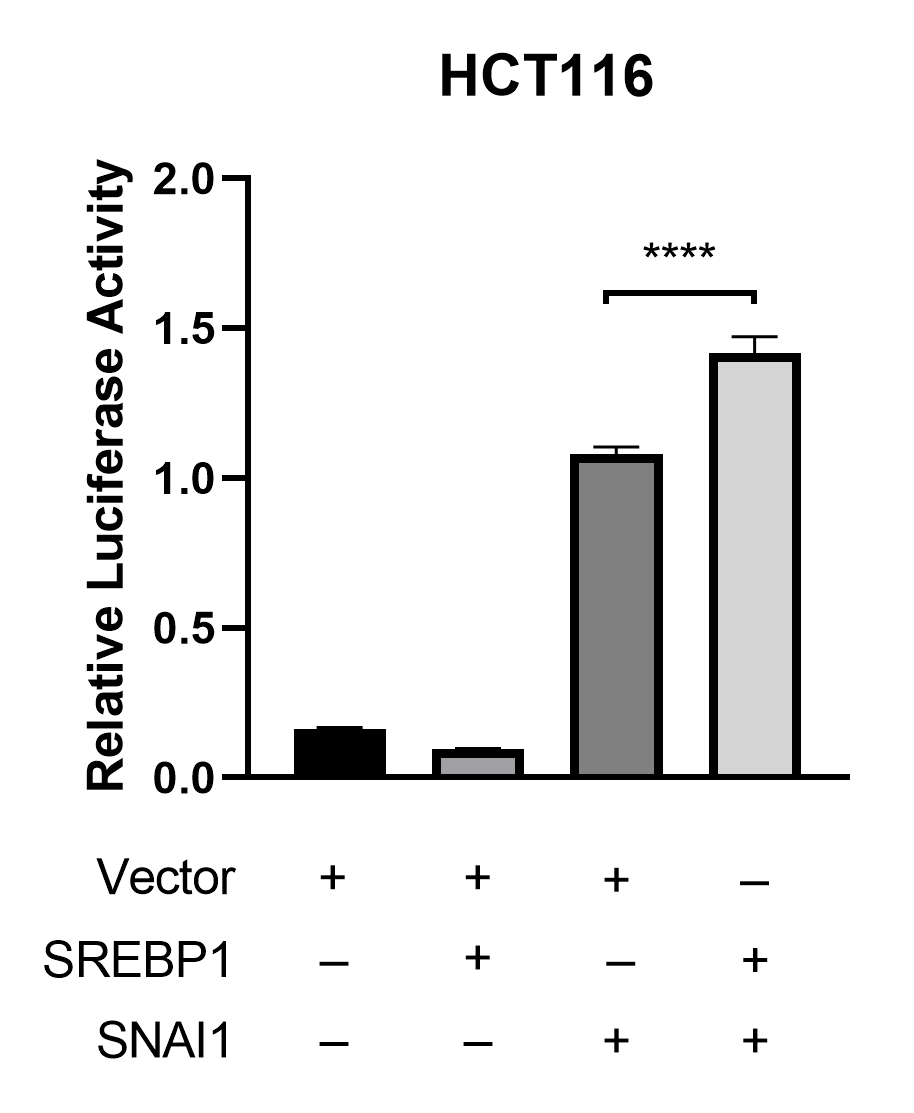

Supplement: Supplementary file 6 [file SupplementaryFile6.zip › 荧光素酶报告基因/hct116.tif]

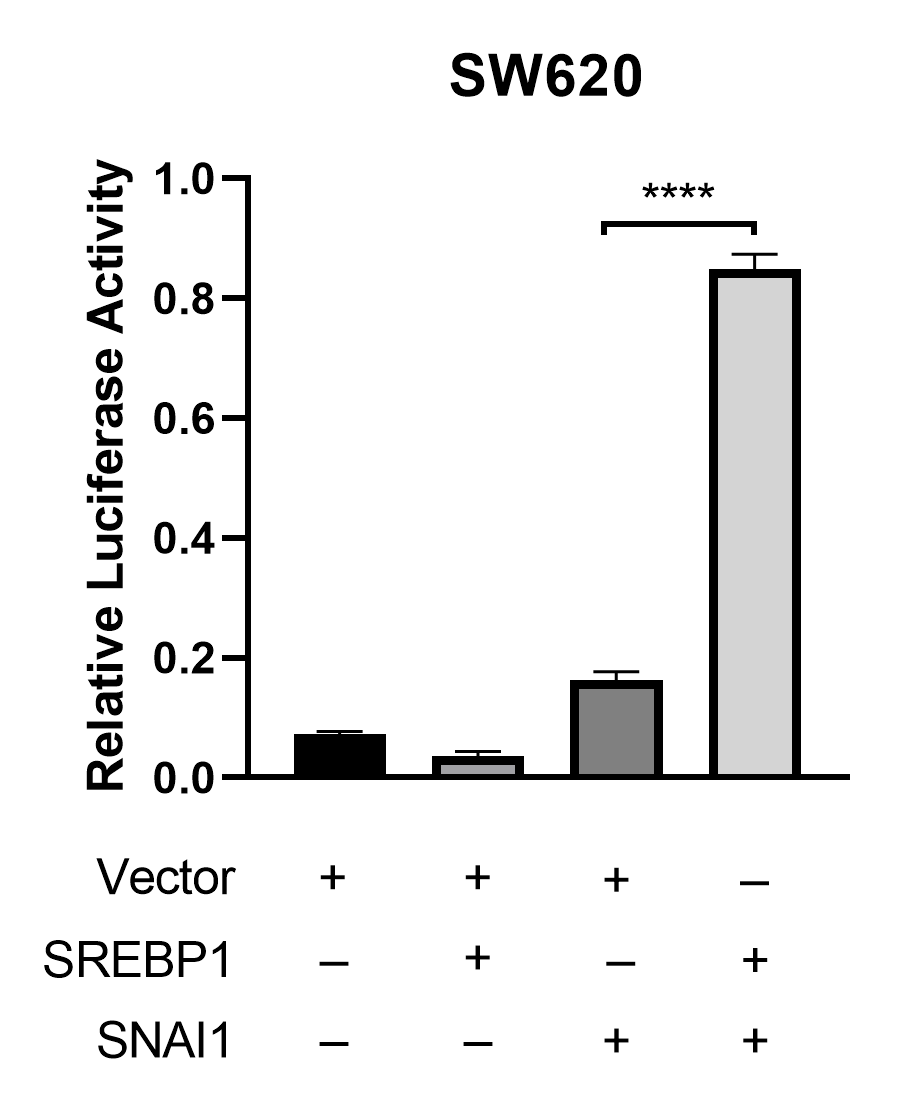

Supplement: Supplementary file 6 [file SupplementaryFile6.zip › 荧光素酶报告基因/SW620.tif]
