# Supplementary figures and images for: Brexpiprazole inhibits EMT and migration of colorectal cancer cells by downregulating the SREBP1/SNAI1 signaling pathway (part 3 of 4)
Source: Front Oncol. 2026 Jan 15;15:1734678. doi: 10.3389/fonc.2025.1734678 (PMC12852020; doi:10.3389/fonc.2025.1734678)

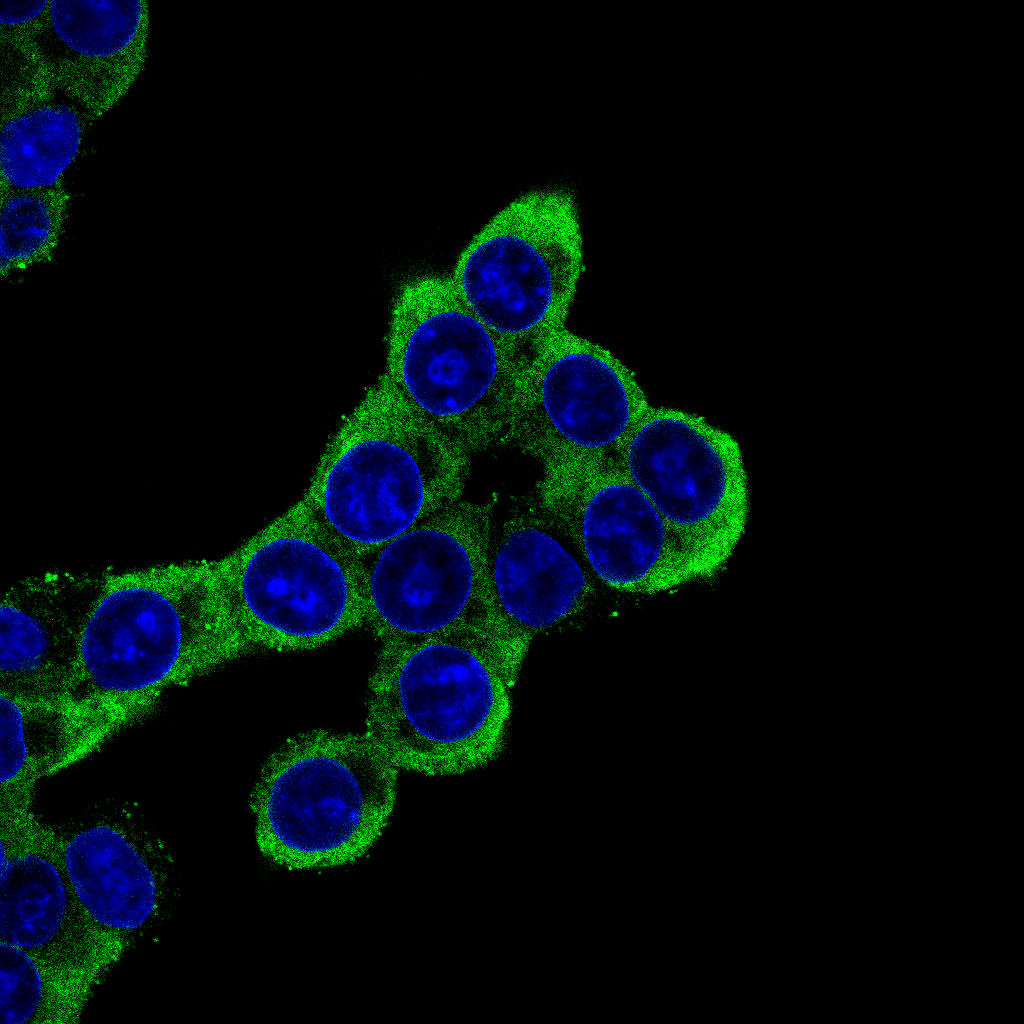

Supplement: Supplementary file 5 [file SupplementaryFile5.zip › 免疫荧光/6.13/E-Cad/lxj-BRE-E_0004.tif.frames/lxj-BRE-E_0004_T001.tif]

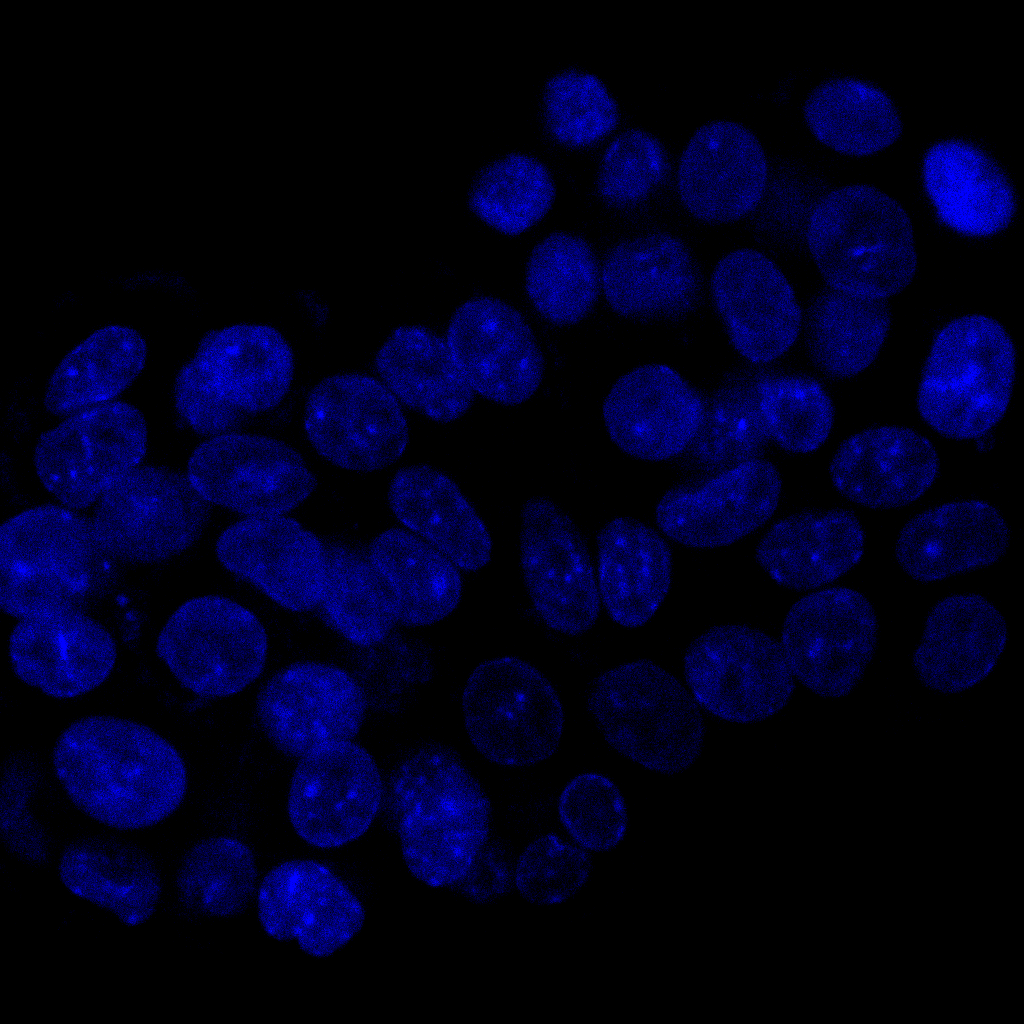

Supplement: Supplementary file 5 [file SupplementaryFile5.zip › 免疫荧光/6.13/E-Cad/lxj-NC-SR1_0003.tif.frames/lxj-NC-SR1_0003_C001T001.tif]

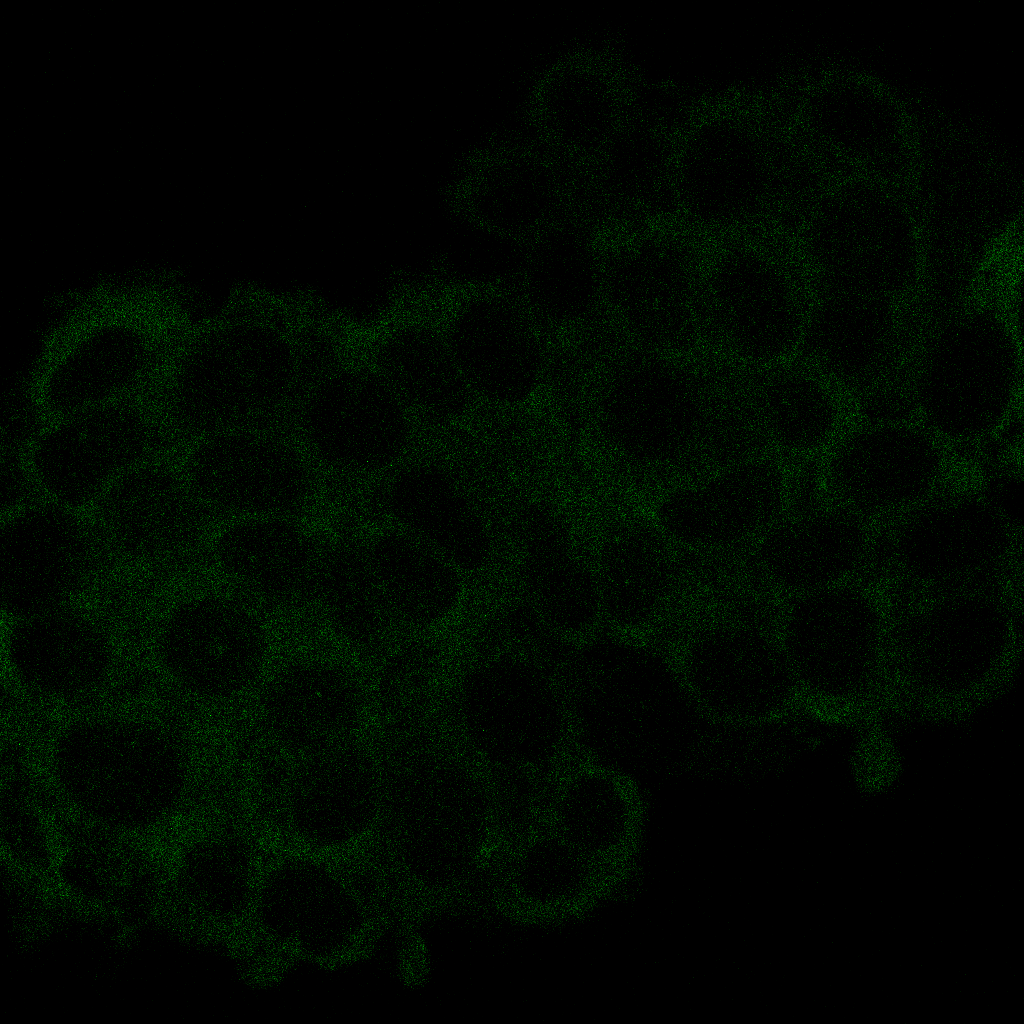

Supplement: Supplementary file 5 [file SupplementaryFile5.zip › 免疫荧光/6.13/E-Cad/lxj-NC-SR1_0003.tif.frames/lxj-NC-SR1_0003_C002T001.tif]

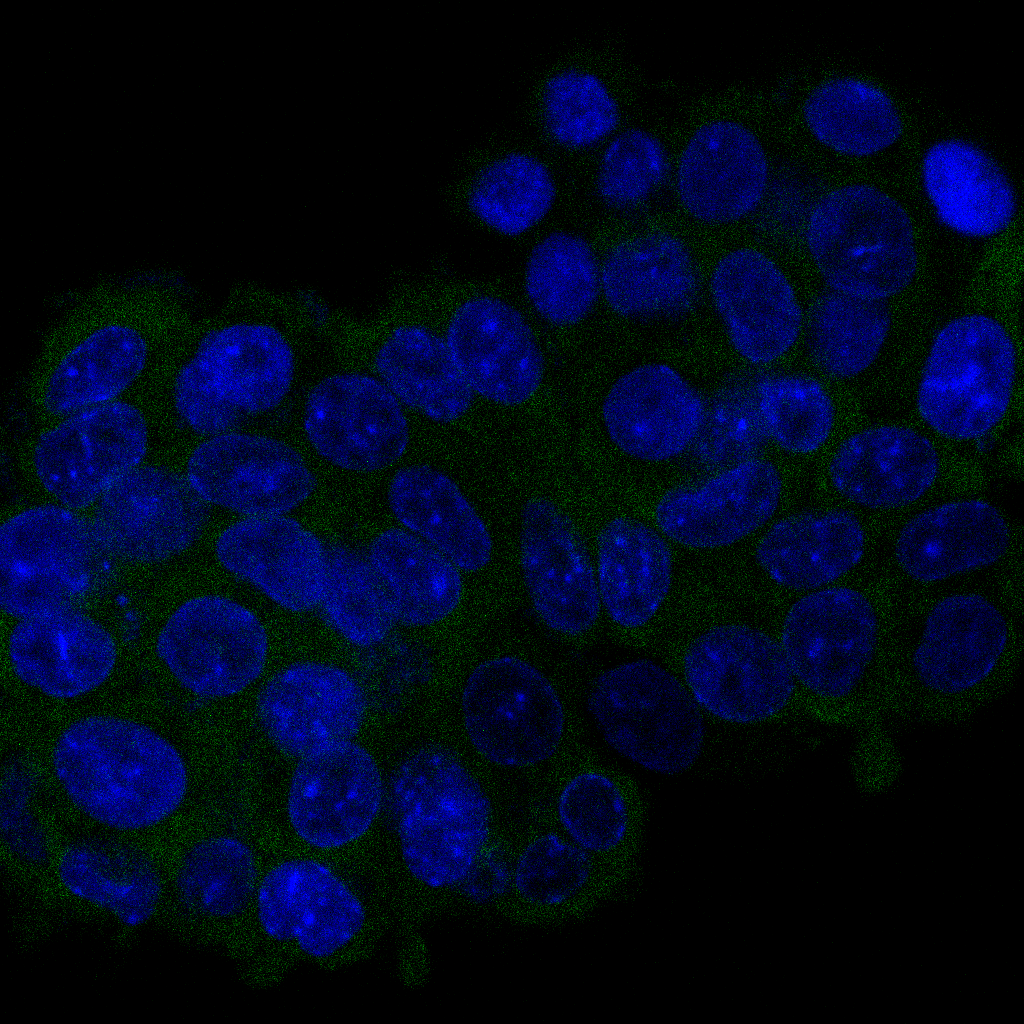

Supplement: Supplementary file 5 [file SupplementaryFile5.zip › 免疫荧光/6.13/E-Cad/lxj-NC-SR1_0003.tif.frames/lxj-NC-SR1_0003_T001.tif]

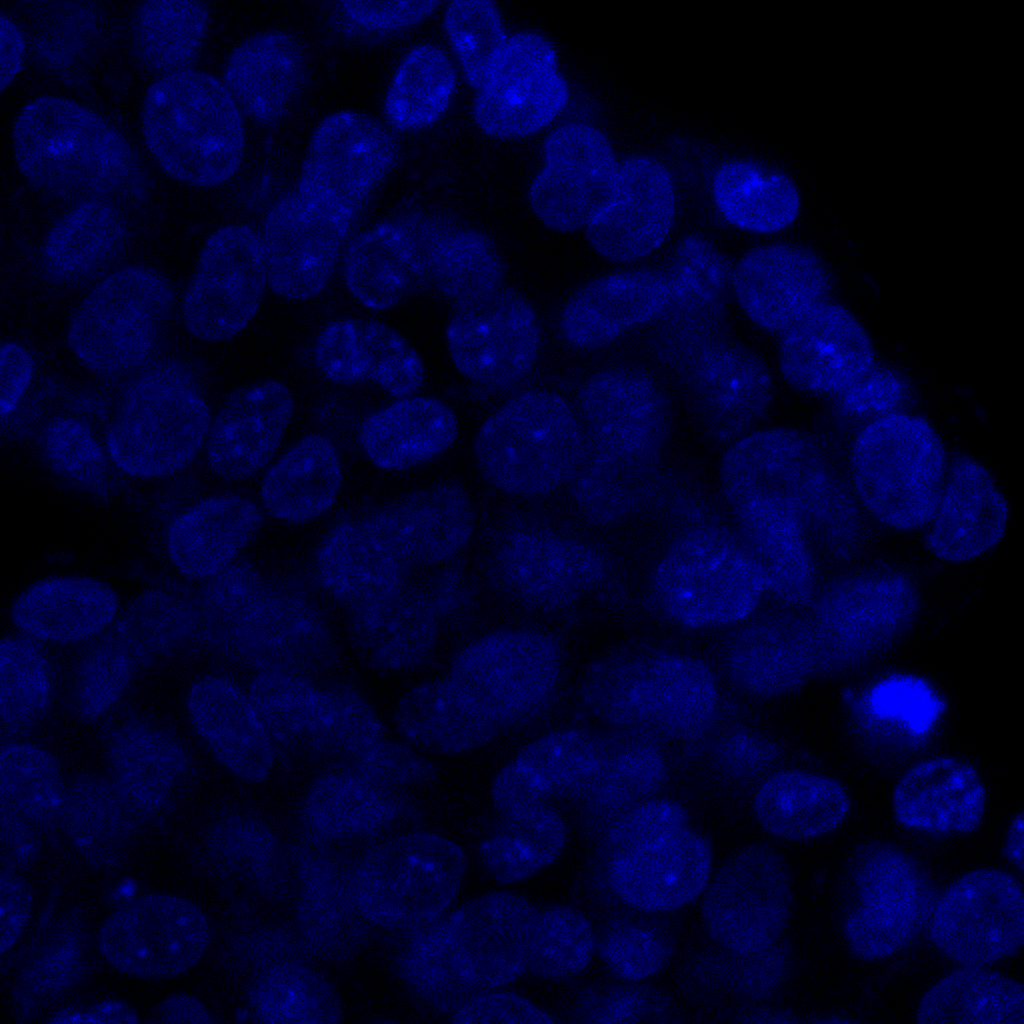

Supplement: Supplementary file 5 [file SupplementaryFile5.zip › 免疫荧光/6.13/E-Cad/lxj-NC-SR1_0006.tif.frames/lxj-NC-SR1_0006_C001T001.tif]

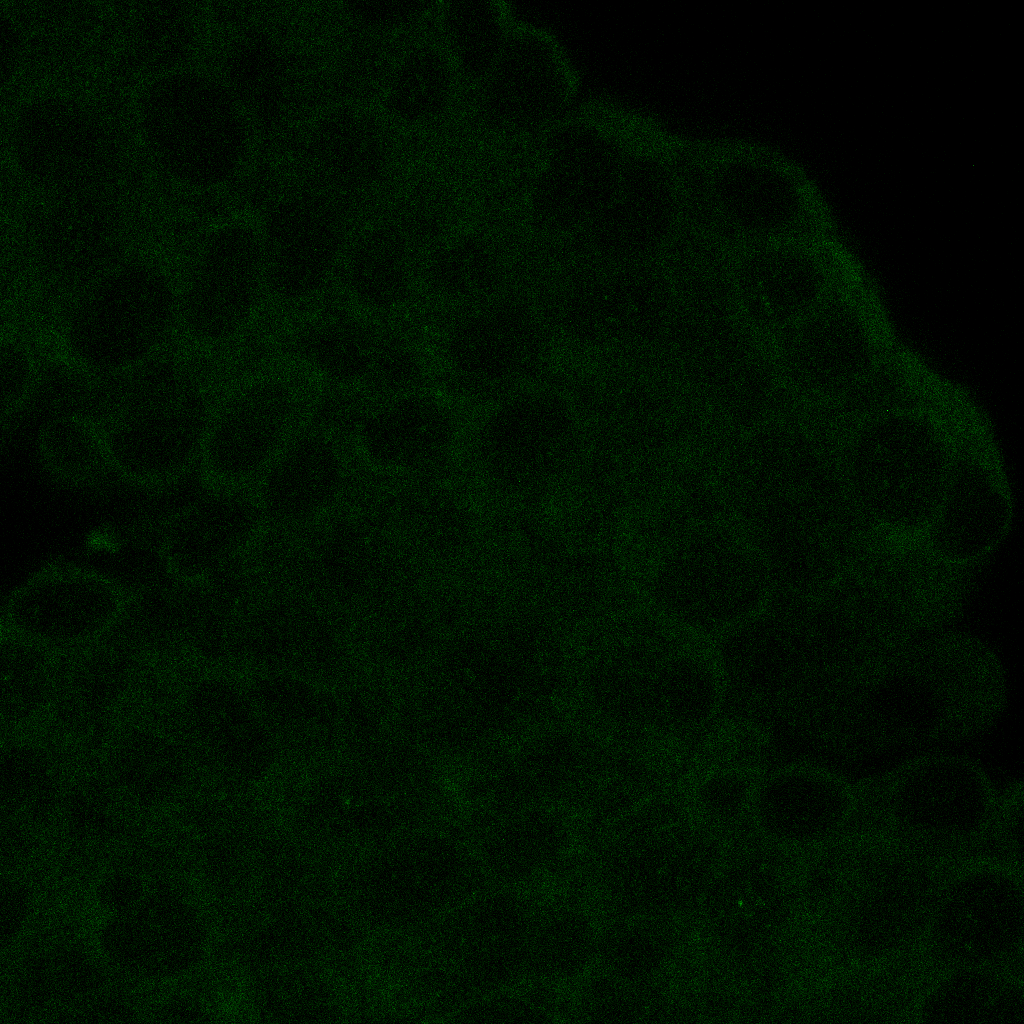

Supplement: Supplementary file 5 [file SupplementaryFile5.zip › 免疫荧光/6.13/E-Cad/lxj-NC-SR1_0006.tif.frames/lxj-NC-SR1_0006_C002T001.tif]

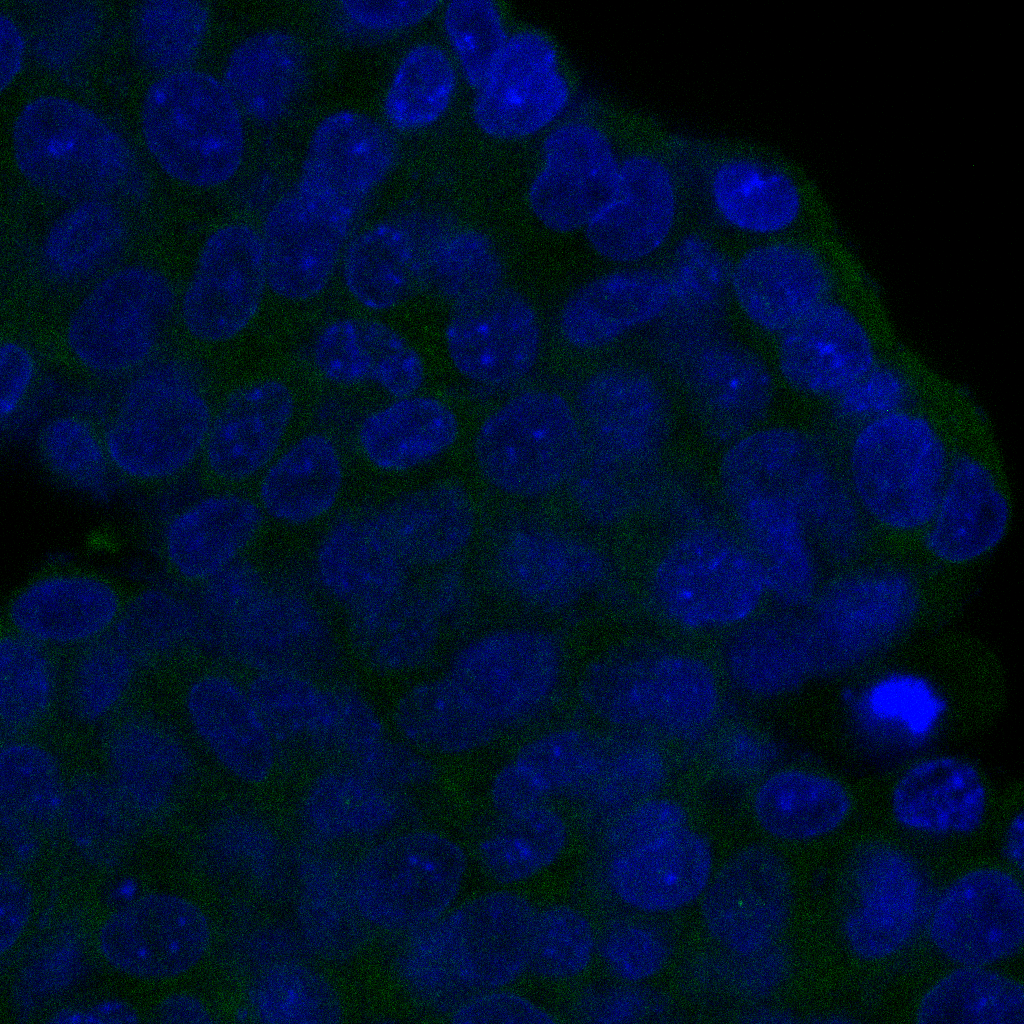

Supplement: Supplementary file 5 [file SupplementaryFile5.zip › 免疫荧光/6.13/E-Cad/lxj-NC-SR1_0006.tif.frames/lxj-NC-SR1_0006_T001.tif]

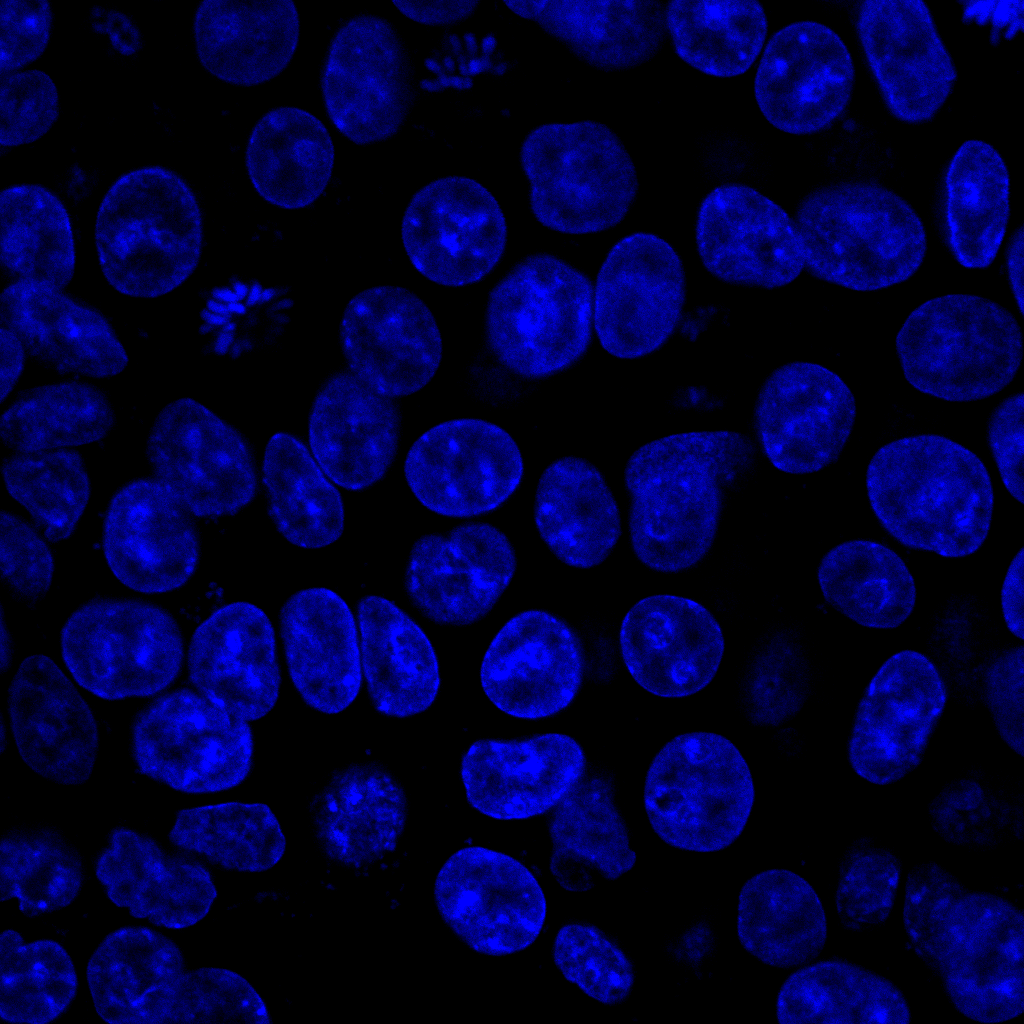

Supplement: Supplementary file 5 [file SupplementaryFile5.zip › 免疫荧光/6.13/E-Cad/lxj-NC-SR1_0007.tif.frames/lxj-NC-SR1_0007_C001T001.tif]

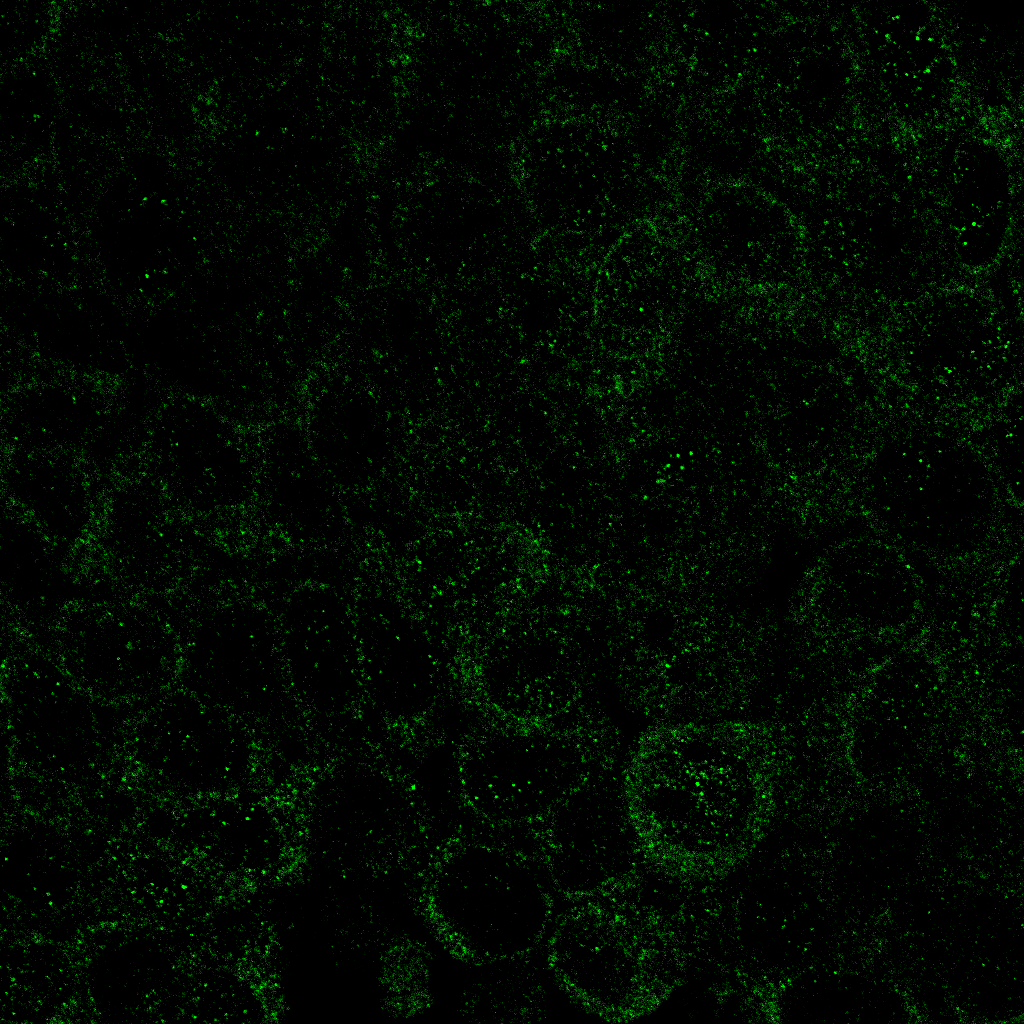

Supplement: Supplementary file 5 [file SupplementaryFile5.zip › 免疫荧光/6.13/E-Cad/lxj-NC-SR1_0007.tif.frames/lxj-NC-SR1_0007_C002T001.tif]

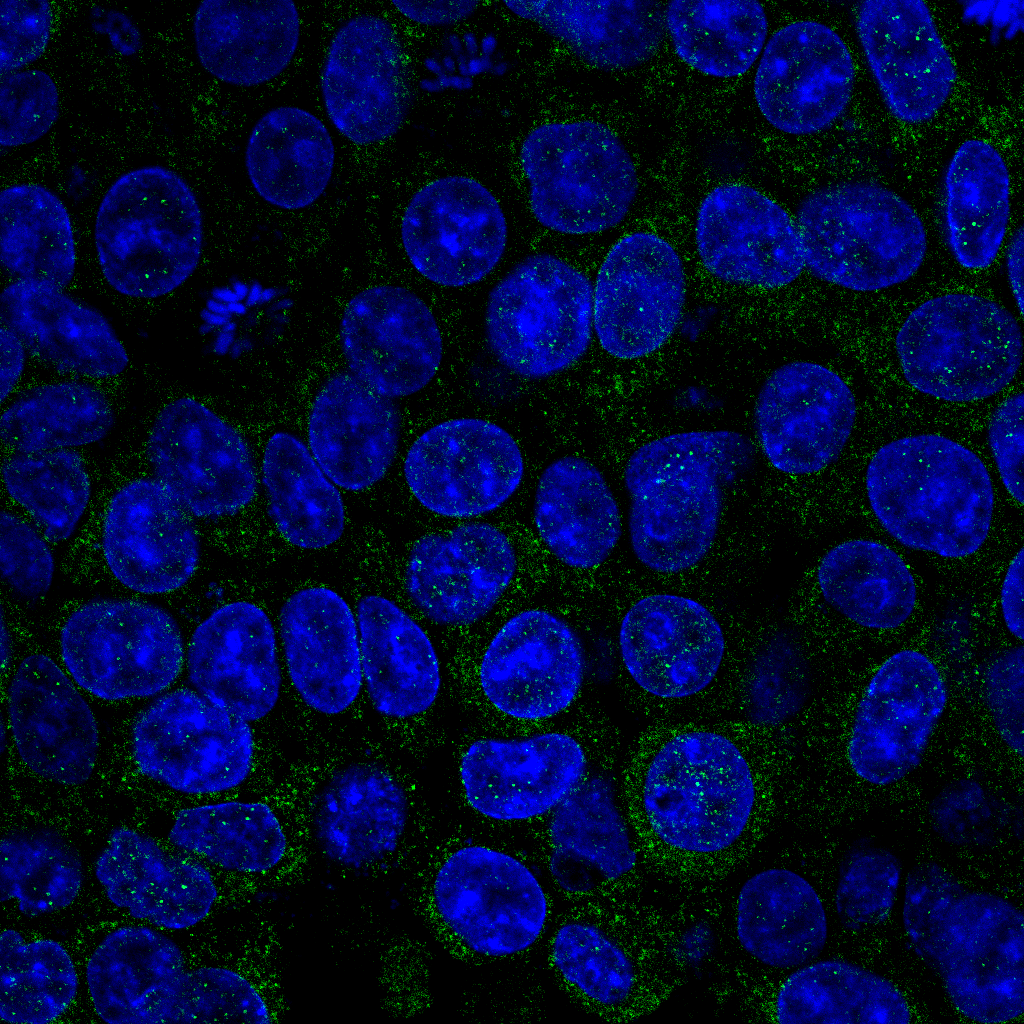

Supplement: Supplementary file 5 [file SupplementaryFile5.zip › 免疫荧光/6.13/E-Cad/lxj-NC-SR1_0007.tif.frames/lxj-NC-SR1_0007_T001.tif]

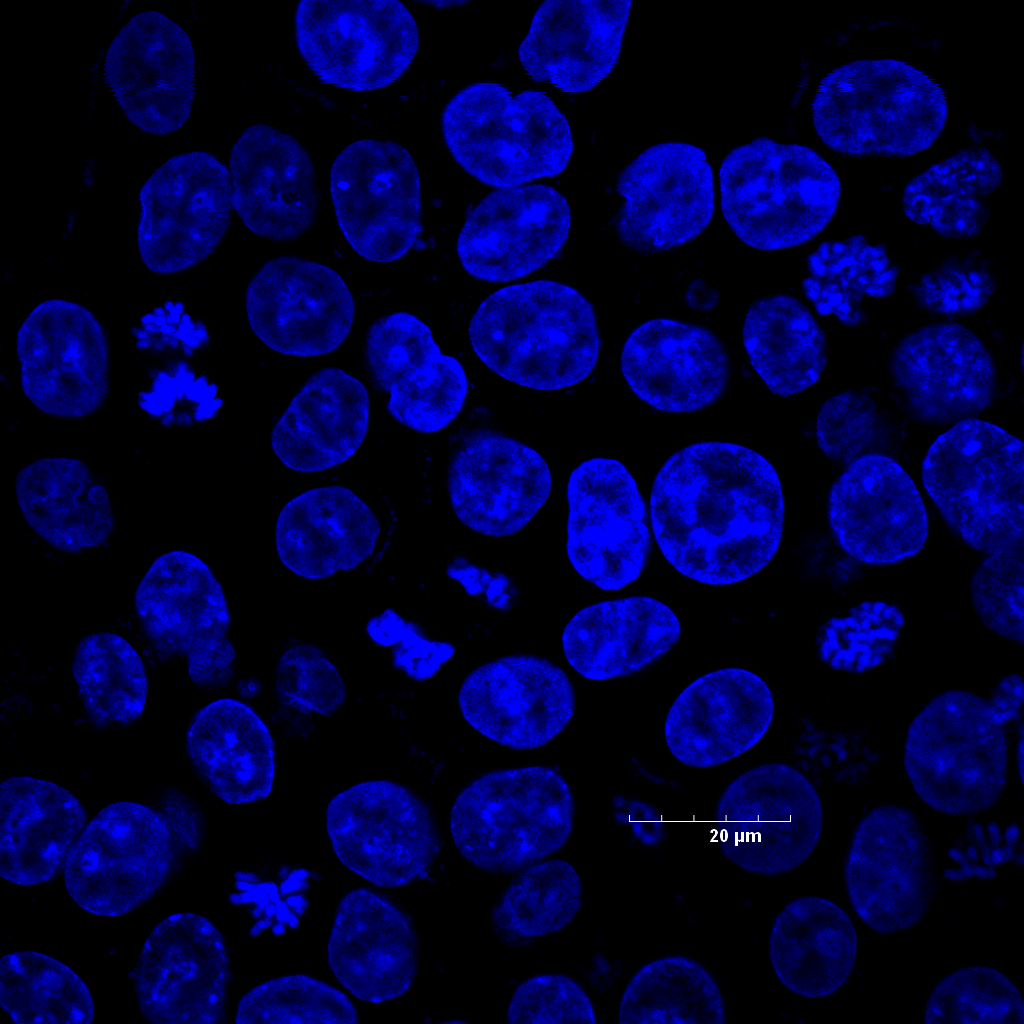

Supplement: Supplementary file 5 [file SupplementaryFile5.zip › 免疫荧光/6.13/E-Cad/lxj-NC-SR1_0008.tif.frames/lxj-NC-SR1_0008_C001T001.tif]

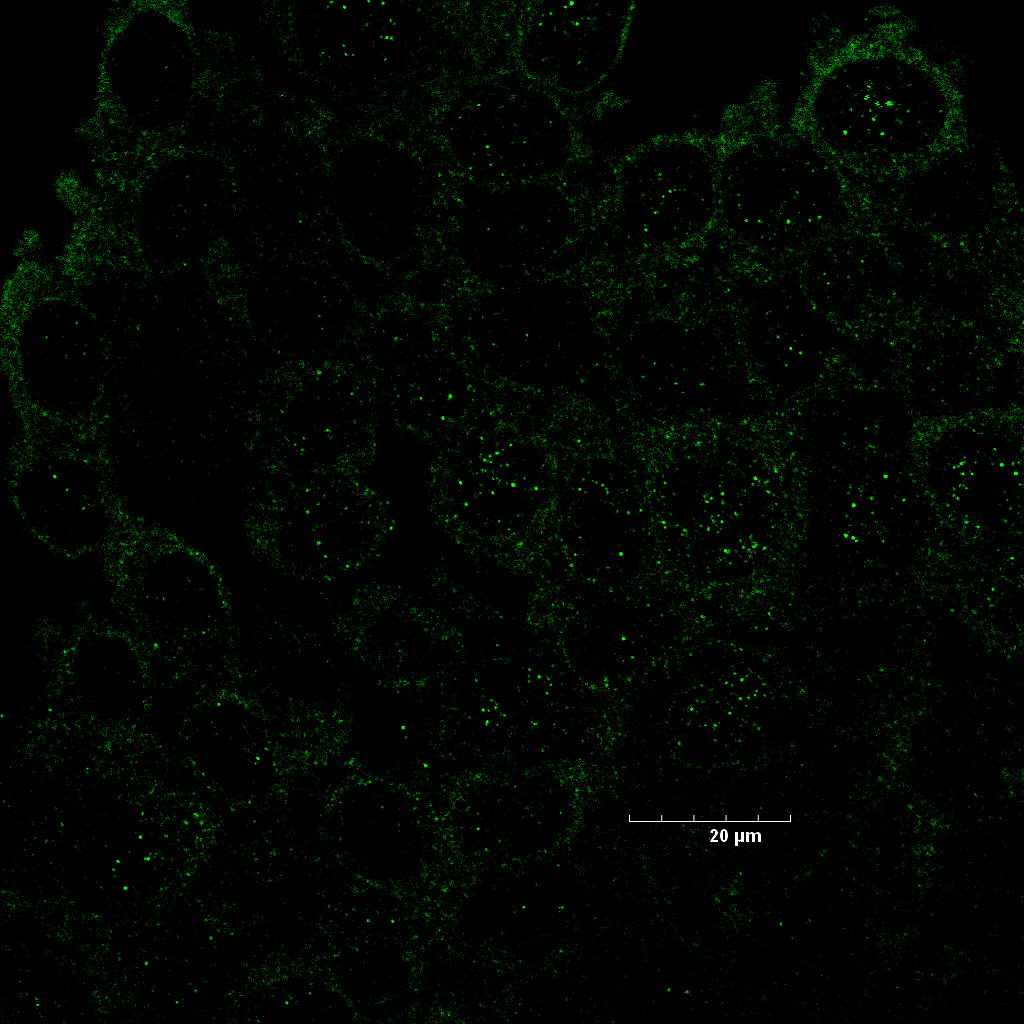

Supplement: Supplementary file 5 [file SupplementaryFile5.zip › 免疫荧光/6.13/E-Cad/lxj-NC-SR1_0008.tif.frames/lxj-NC-SR1_0008_C002T001.tif]

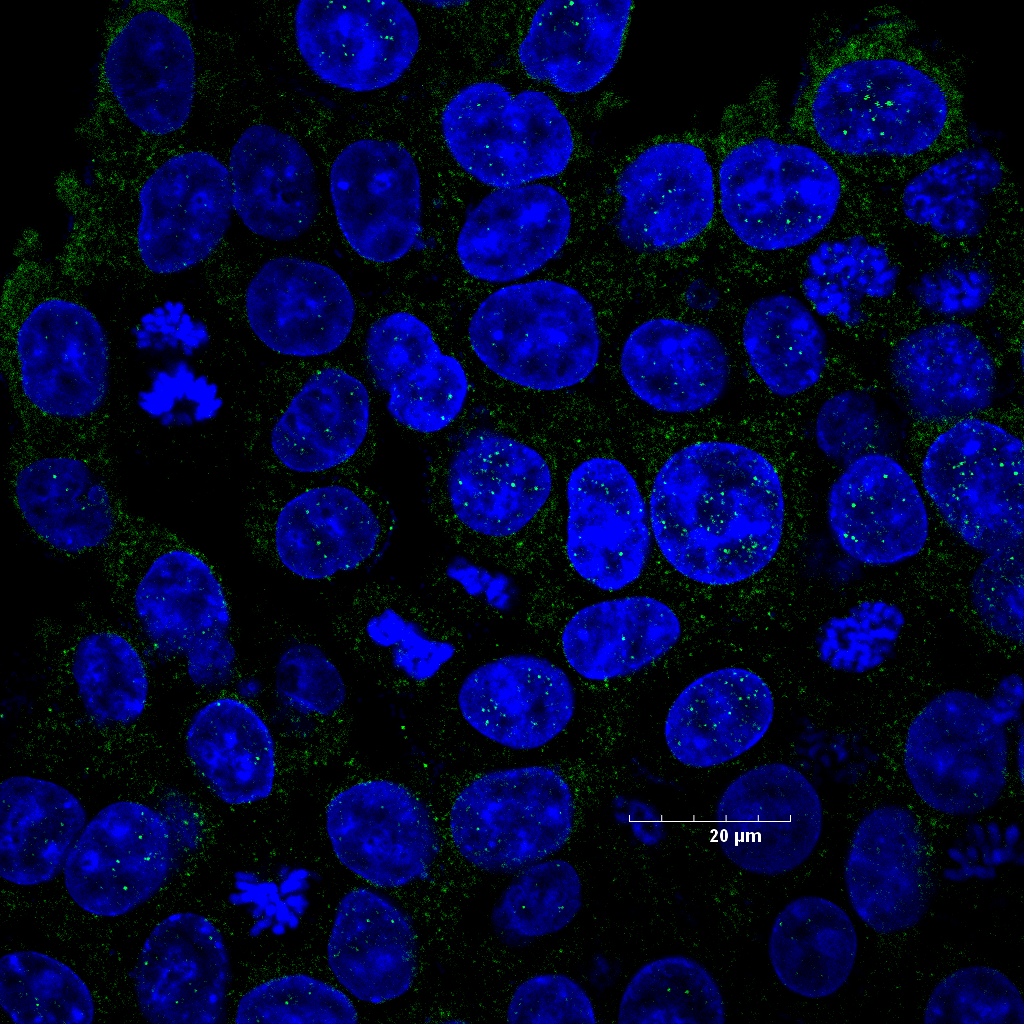

Supplement: Supplementary file 5 [file SupplementaryFile5.zip › 免疫荧光/6.13/E-Cad/lxj-NC-SR1_0008.tif.frames/lxj-NC-SR1_0008_T001.tif]

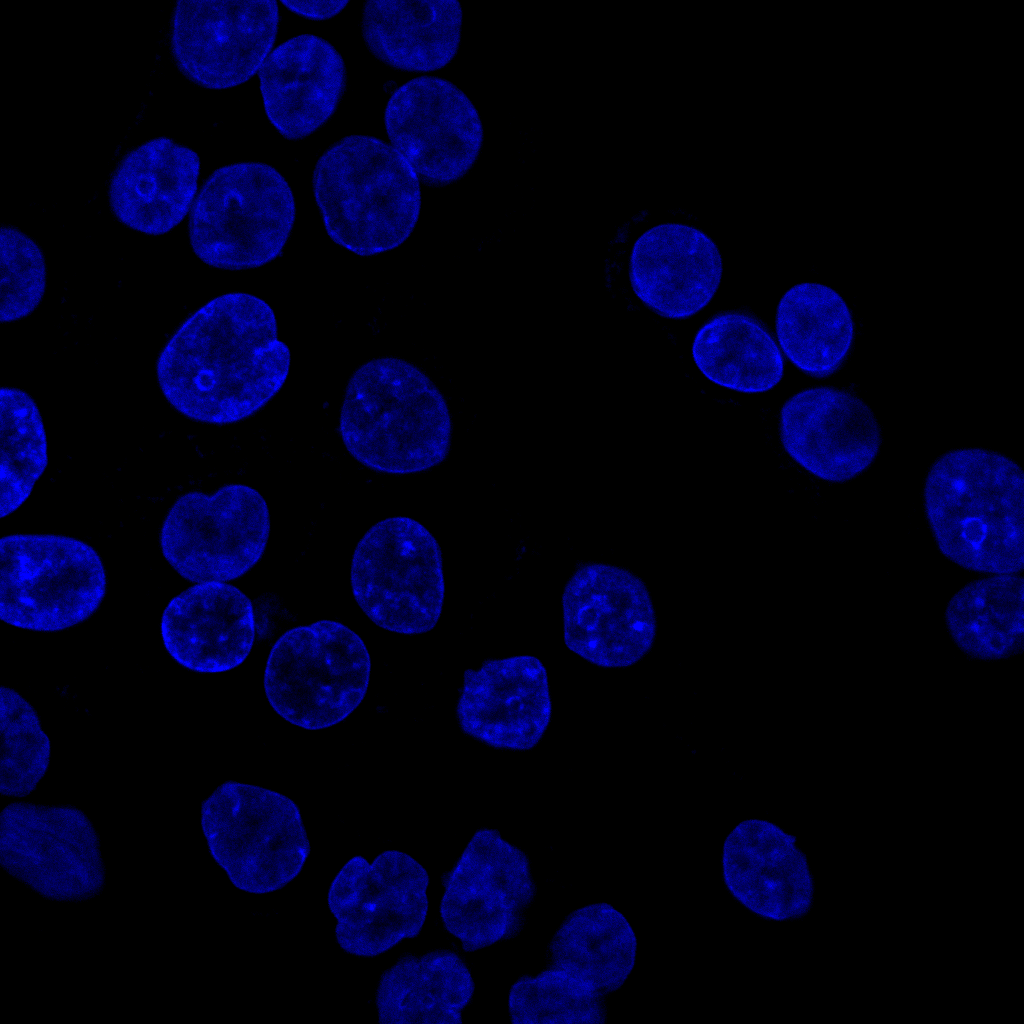

Supplement: Supplementary file 5 [file SupplementaryFile5.zip › 免疫荧光/6.13/E-Cad/lxj-NC-SR1_0013.tif.frames/lxj-NC-SR1_0013_C001T001.tif]

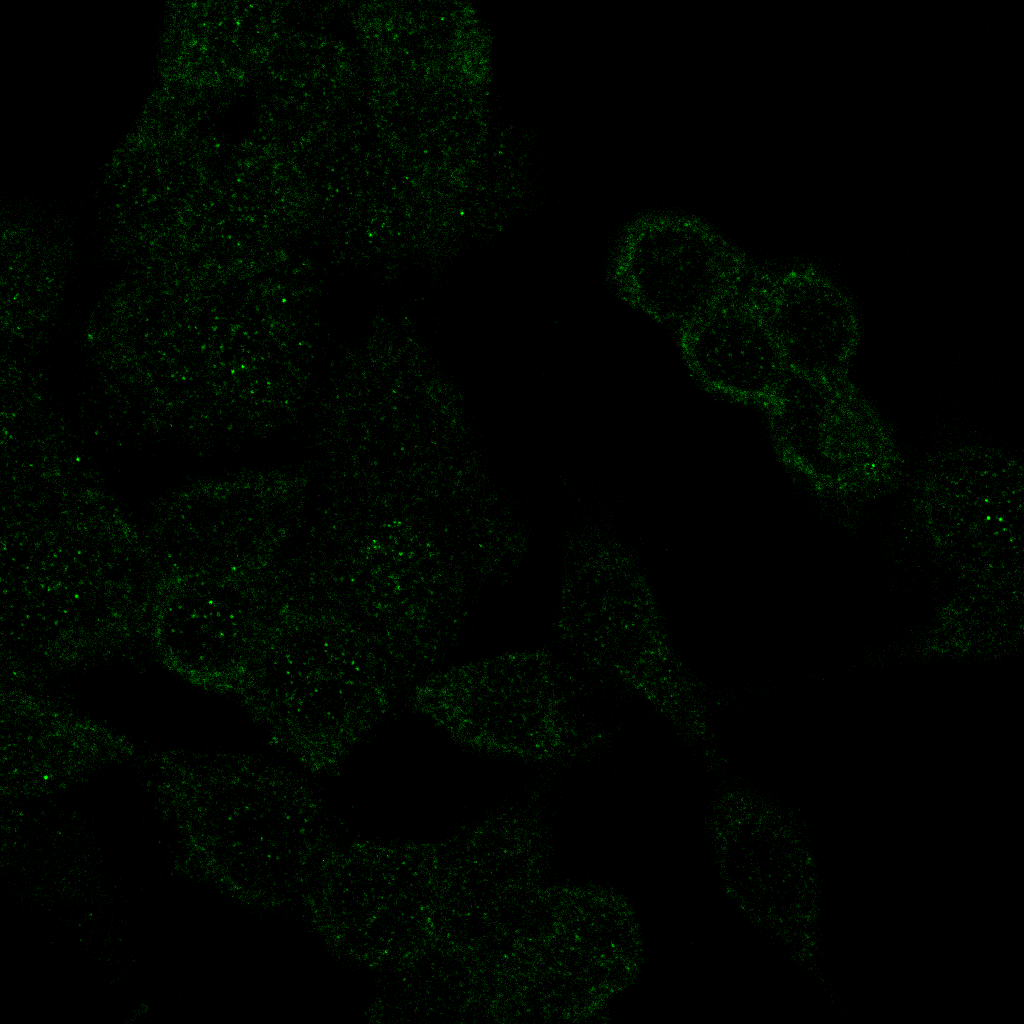

Supplement: Supplementary file 5 [file SupplementaryFile5.zip › 免疫荧光/6.13/E-Cad/lxj-NC-SR1_0013.tif.frames/lxj-NC-SR1_0013_C002T001.tif]

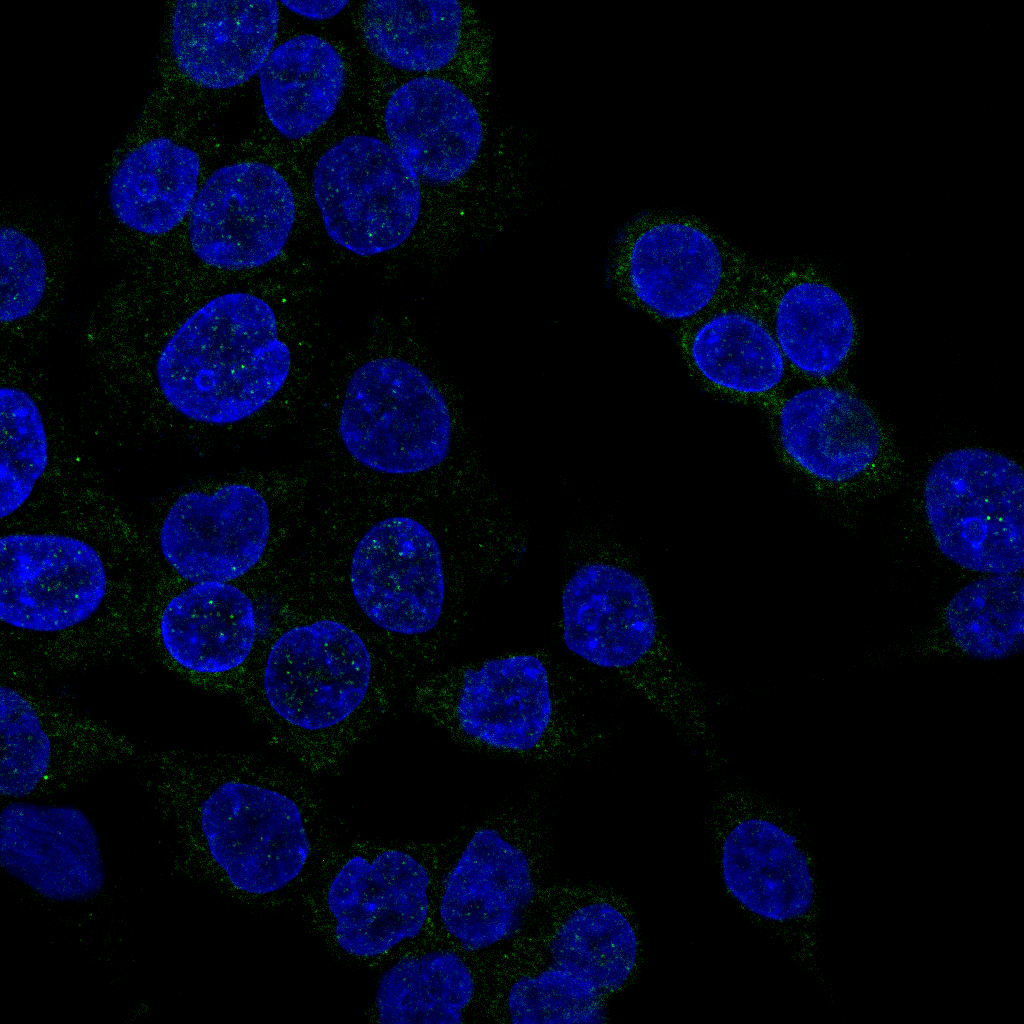

Supplement: Supplementary file 5 [file SupplementaryFile5.zip › 免疫荧光/6.13/E-Cad/lxj-NC-SR1_0013.tif.frames/lxj-NC-SR1_0013_T001.tif]

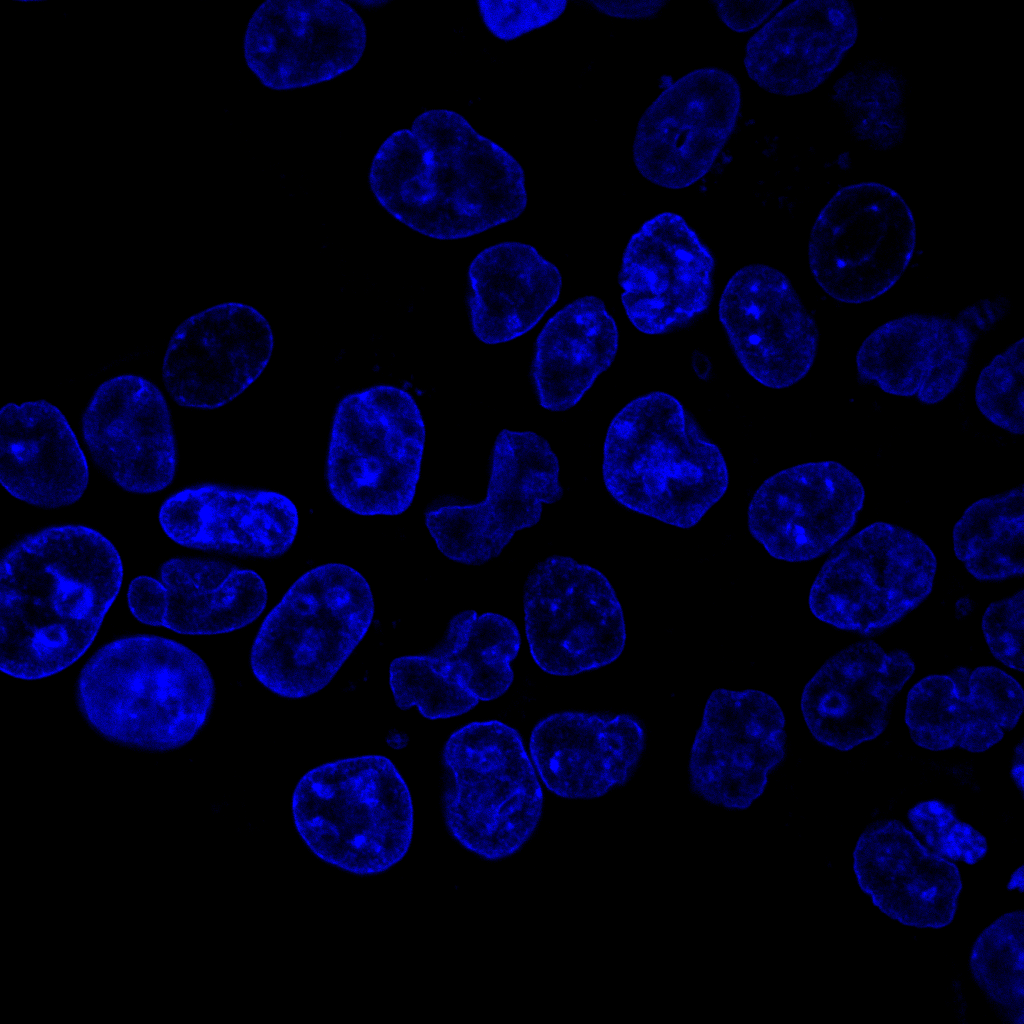

Supplement: Supplementary file 5 [file SupplementaryFile5.zip › 免疫荧光/6.13/SREBP1/lxj-BRE-SR1_0003.tif.frames/lxj-BRE-SR1_0003_C001T001.tif]

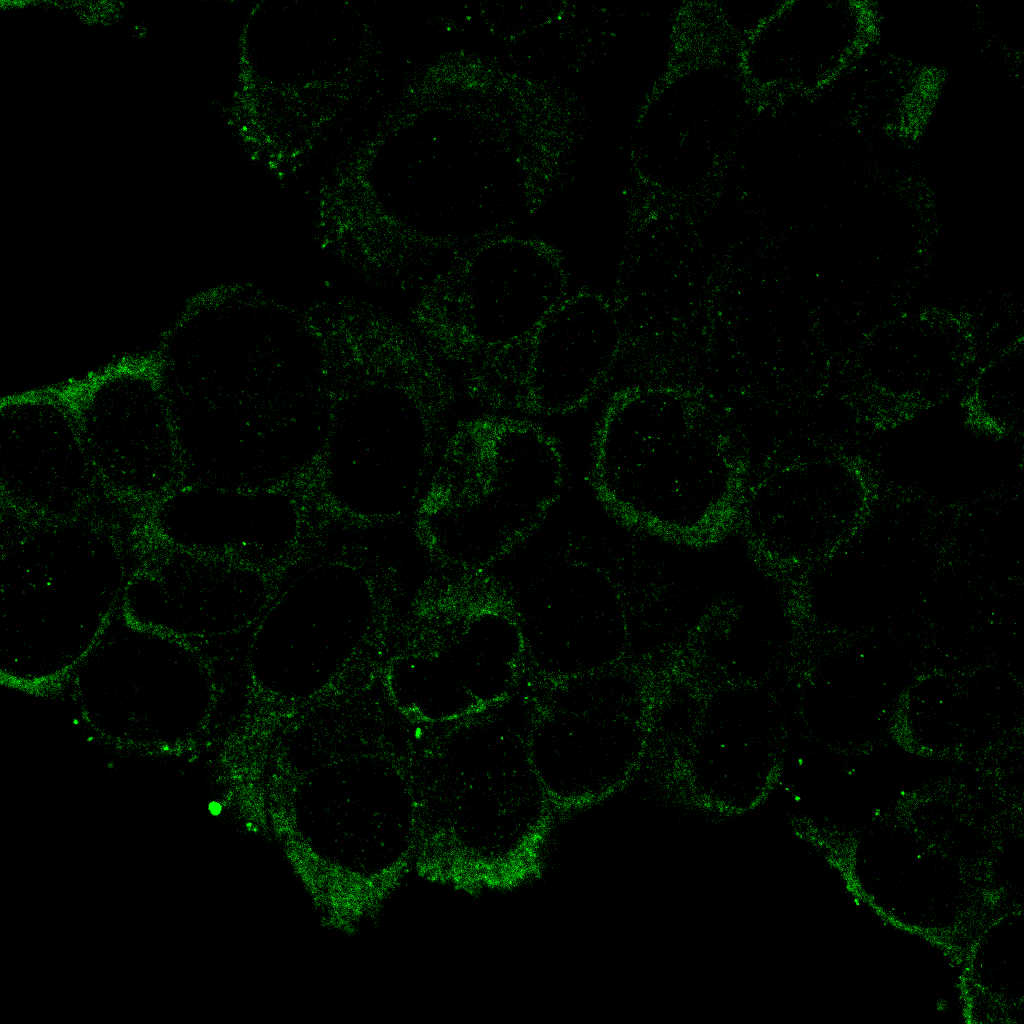

Supplement: Supplementary file 5 [file SupplementaryFile5.zip › 免疫荧光/6.13/SREBP1/lxj-BRE-SR1_0003.tif.frames/lxj-BRE-SR1_0003_C002T001.tif]

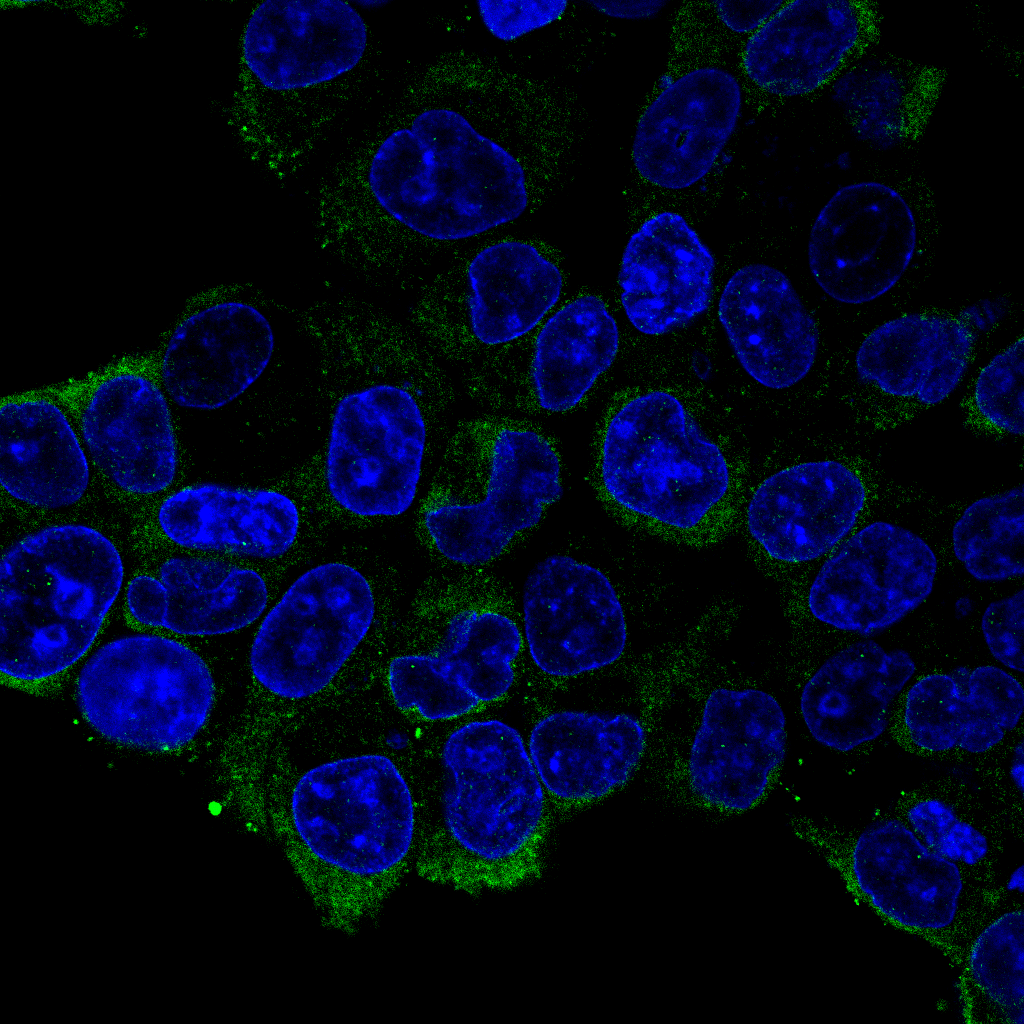

Supplement: Supplementary file 5 [file SupplementaryFile5.zip › 免疫荧光/6.13/SREBP1/lxj-BRE-SR1_0003.tif.frames/lxj-BRE-SR1_0003_T001.tif]

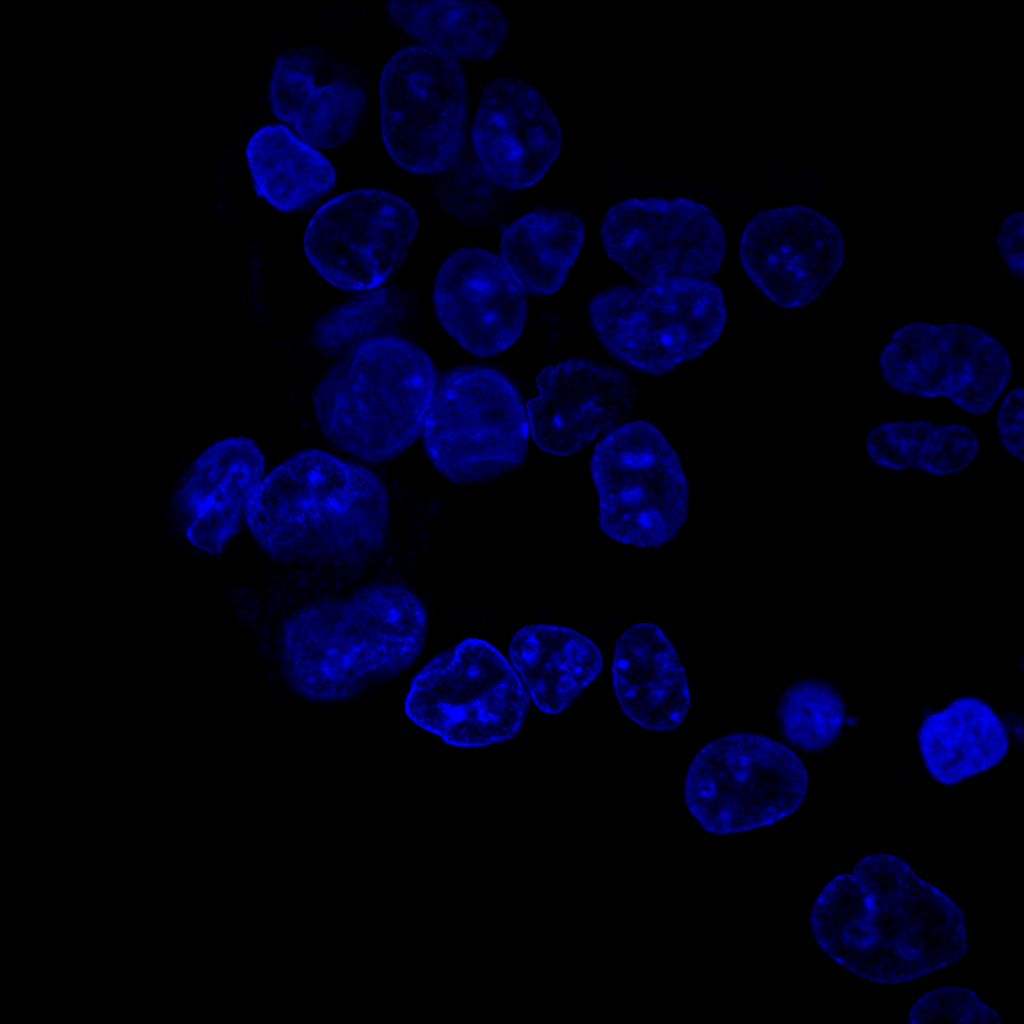

Supplement: Supplementary file 5 [file SupplementaryFile5.zip › 免疫荧光/6.13/SREBP1/lxj-BRE-SR1_0004.tif.frames/lxj-BRE-SR1_0004_C001T001.tif]

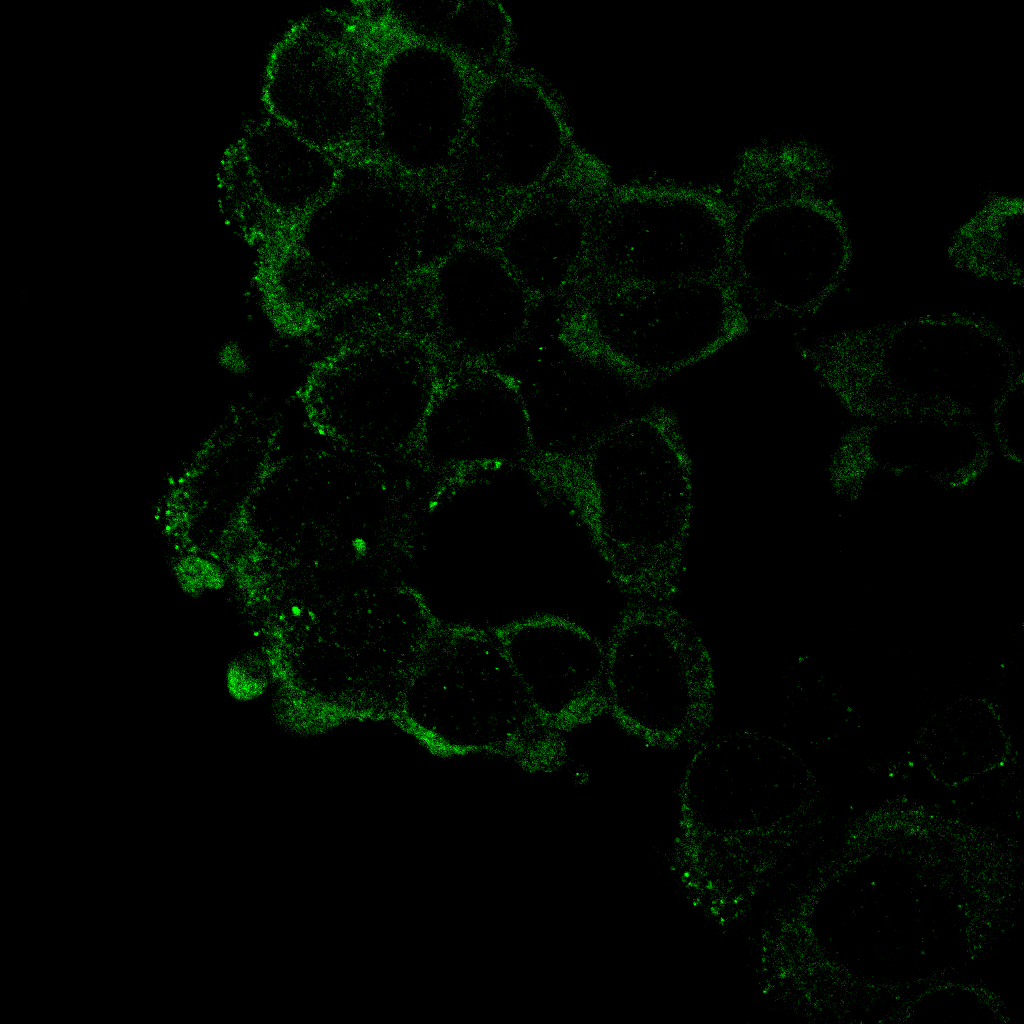

Supplement: Supplementary file 5 [file SupplementaryFile5.zip › 免疫荧光/6.13/SREBP1/lxj-BRE-SR1_0004.tif.frames/lxj-BRE-SR1_0004_C002T001.tif]

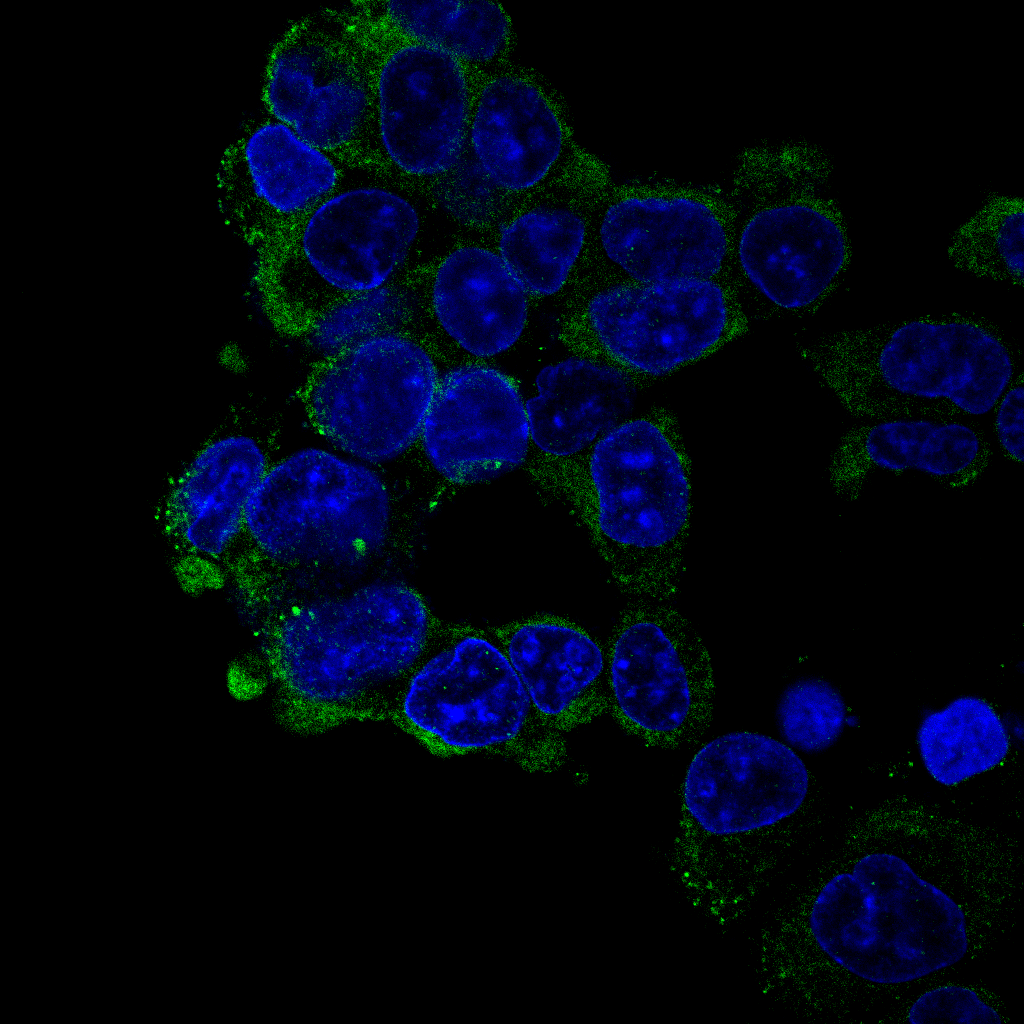

Supplement: Supplementary file 5 [file SupplementaryFile5.zip › 免疫荧光/6.13/SREBP1/lxj-BRE-SR1_0004.tif.frames/lxj-BRE-SR1_0004_T001.tif]

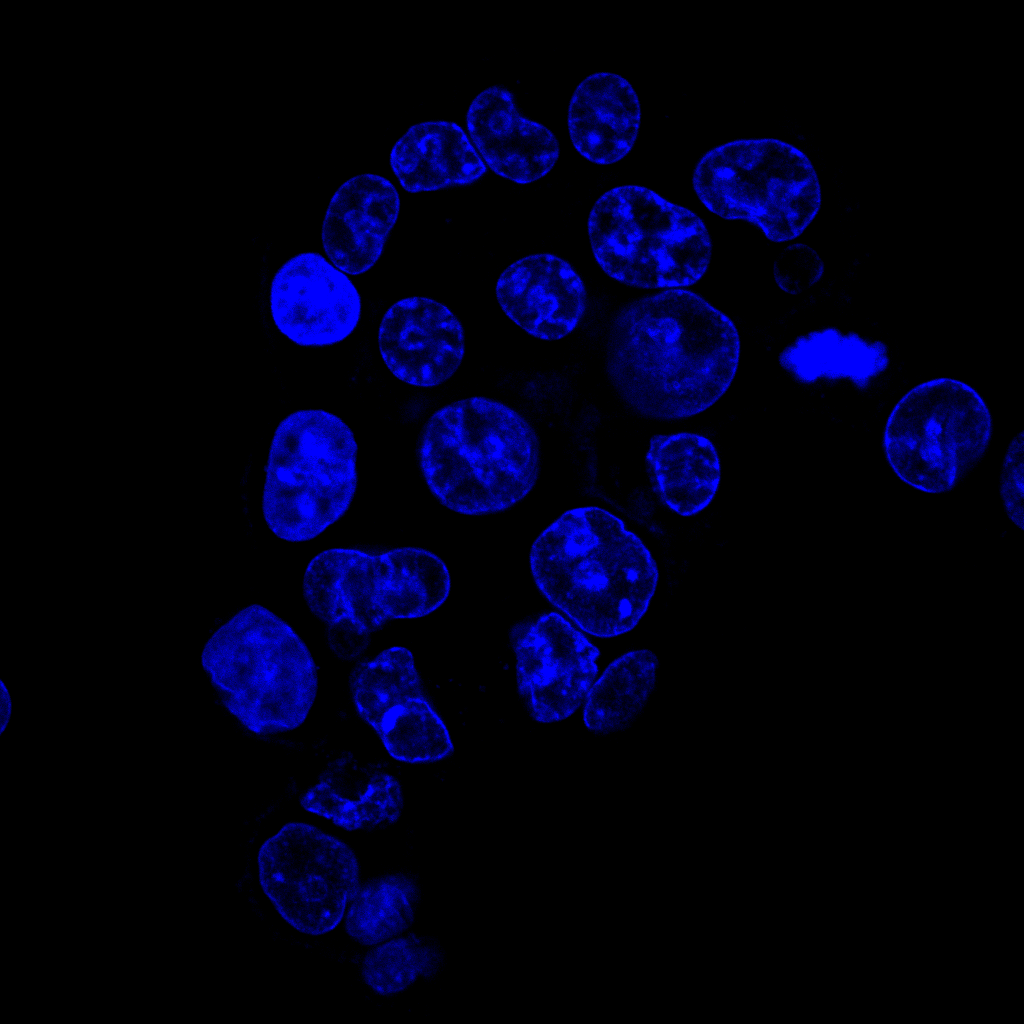

Supplement: Supplementary file 5 [file SupplementaryFile5.zip › 免疫荧光/6.13/SREBP1/lxj-BRE-SR1_0006.tif.frames/lxj-BRE-SR1_0006_C001T001.tif]

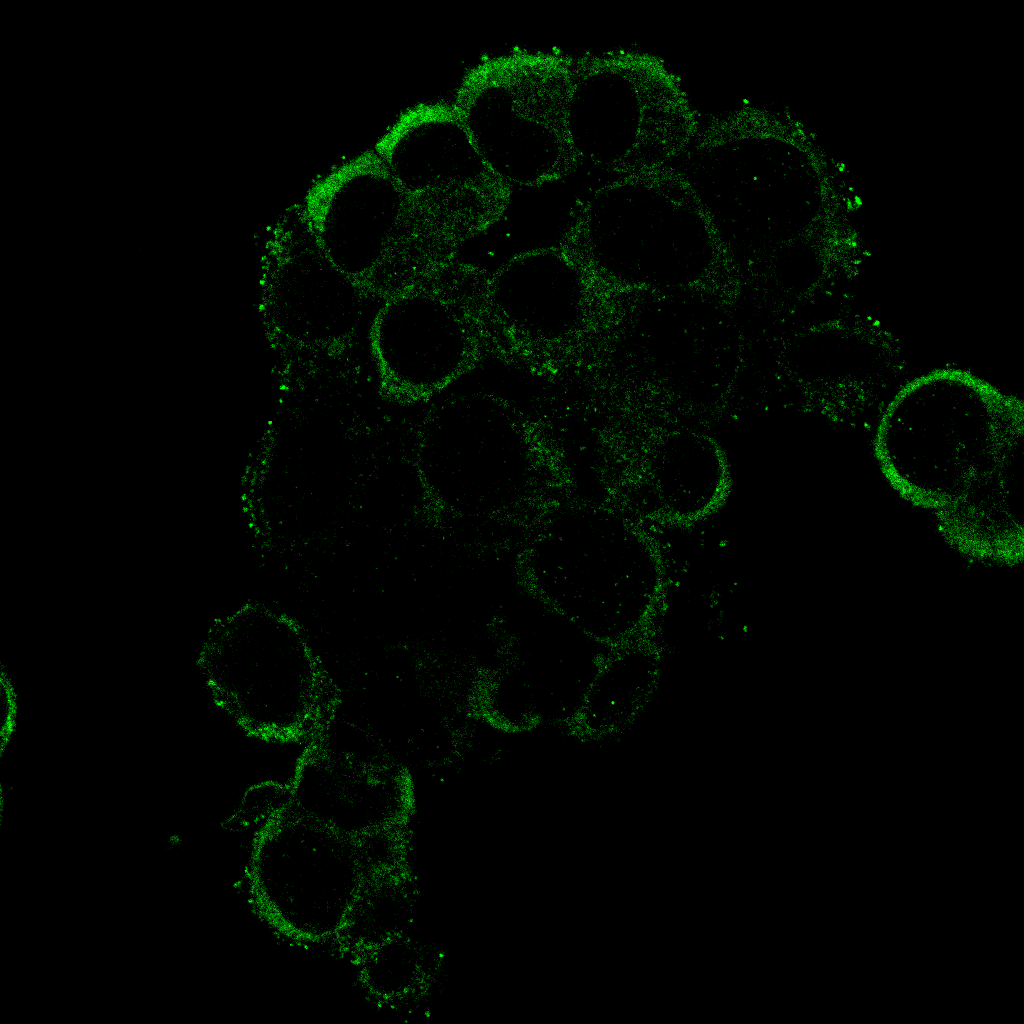

Supplement: Supplementary file 5 [file SupplementaryFile5.zip › 免疫荧光/6.13/SREBP1/lxj-BRE-SR1_0006.tif.frames/lxj-BRE-SR1_0006_C002T001.tif]

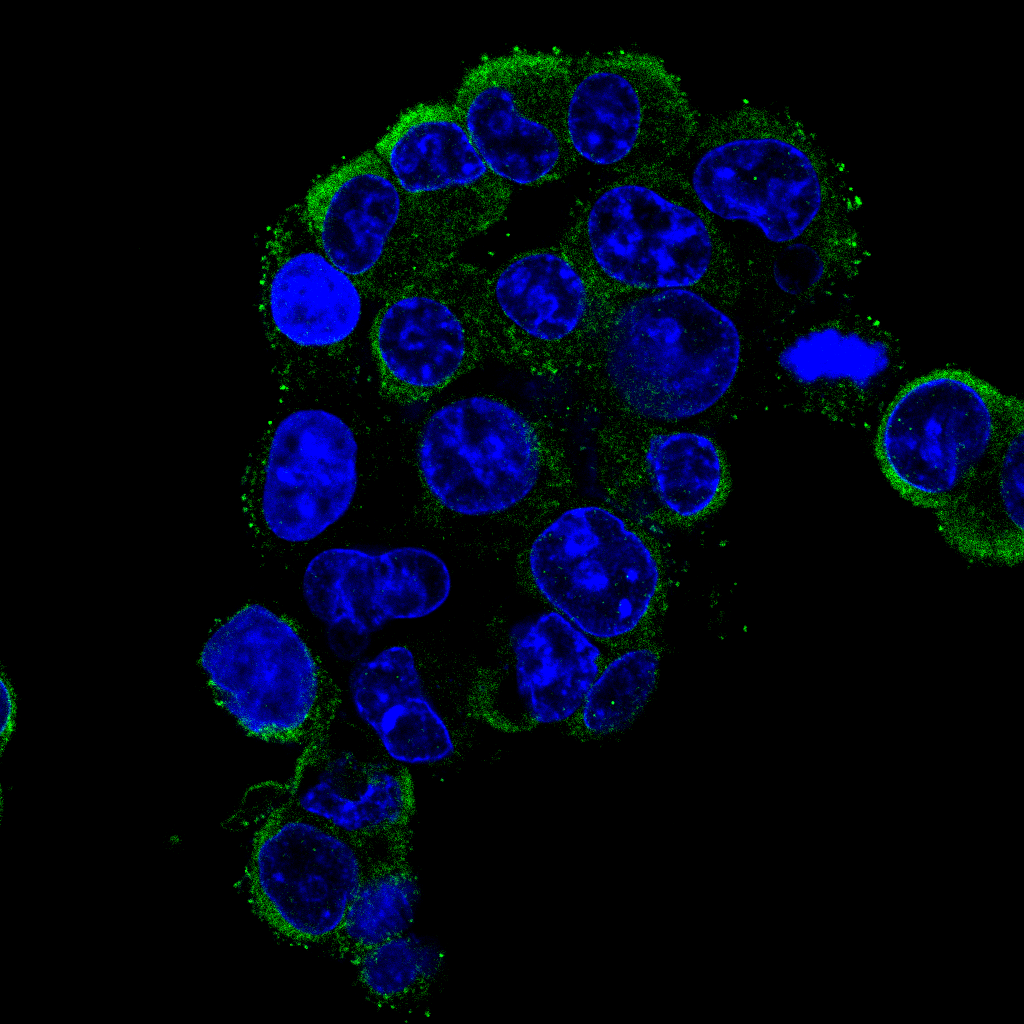

Supplement: Supplementary file 5 [file SupplementaryFile5.zip › 免疫荧光/6.13/SREBP1/lxj-BRE-SR1_0006.tif.frames/lxj-BRE-SR1_0006_T001.tif]

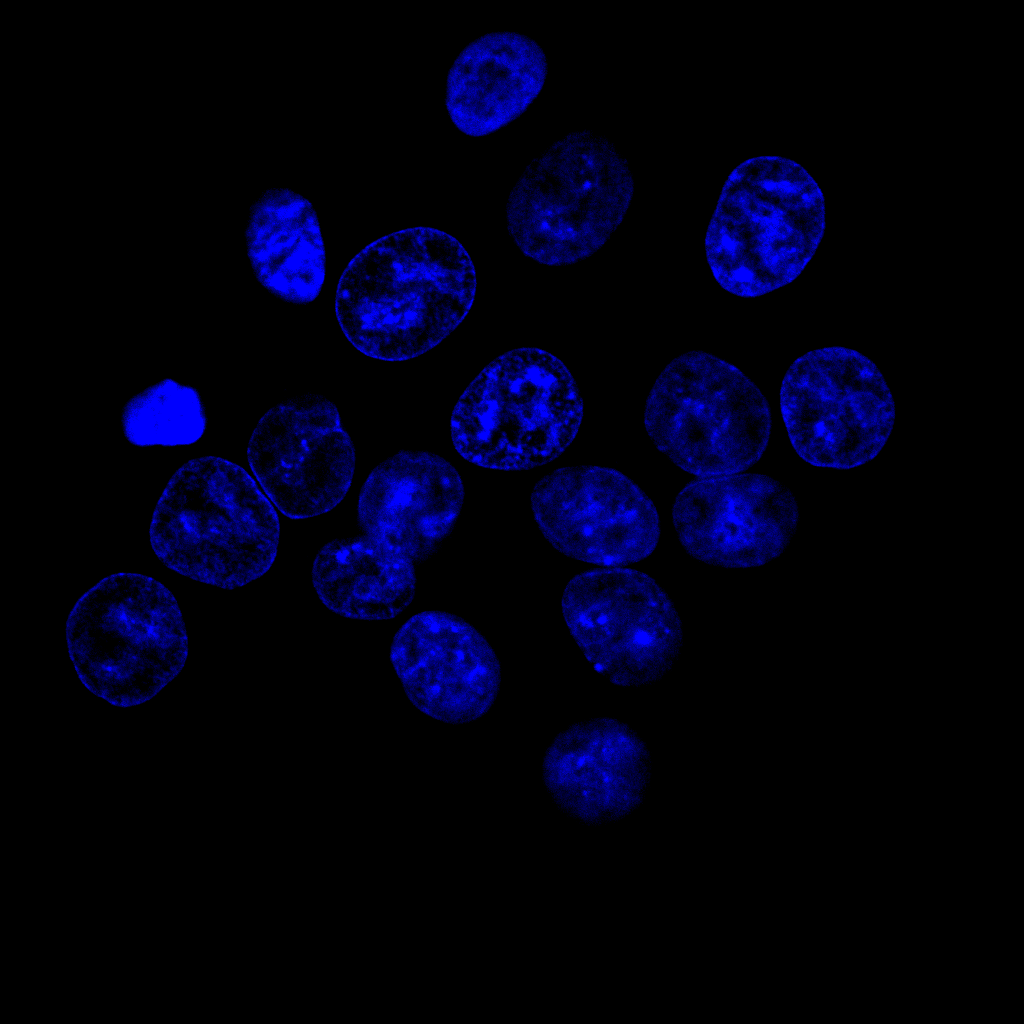

Supplement: Supplementary file 5 [file SupplementaryFile5.zip › 免疫荧光/6.13/SREBP1/lxj-BRE-SR1_0007.tif.frames/lxj-BRE-SR1_0007_C001T001.tif]

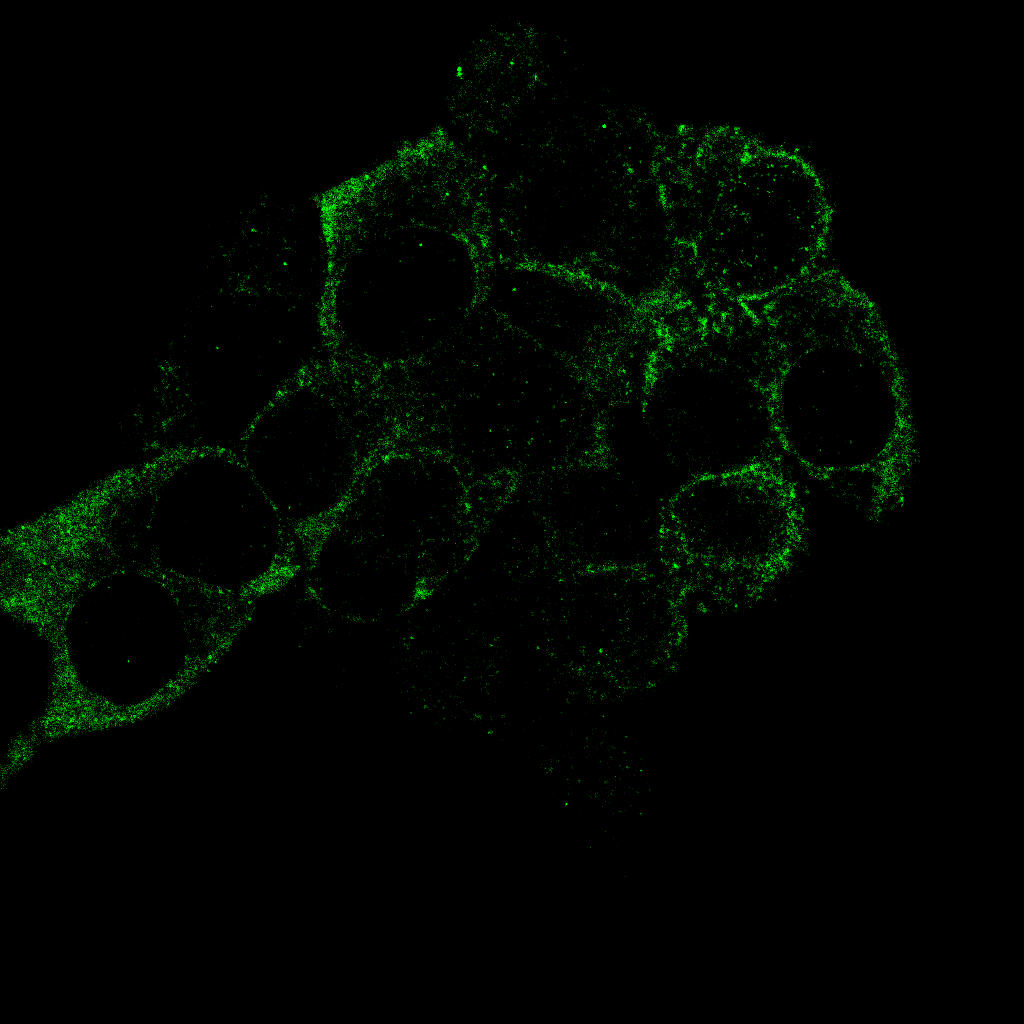

Supplement: Supplementary file 5 [file SupplementaryFile5.zip › 免疫荧光/6.13/SREBP1/lxj-BRE-SR1_0007.tif.frames/lxj-BRE-SR1_0007_C002T001.tif]

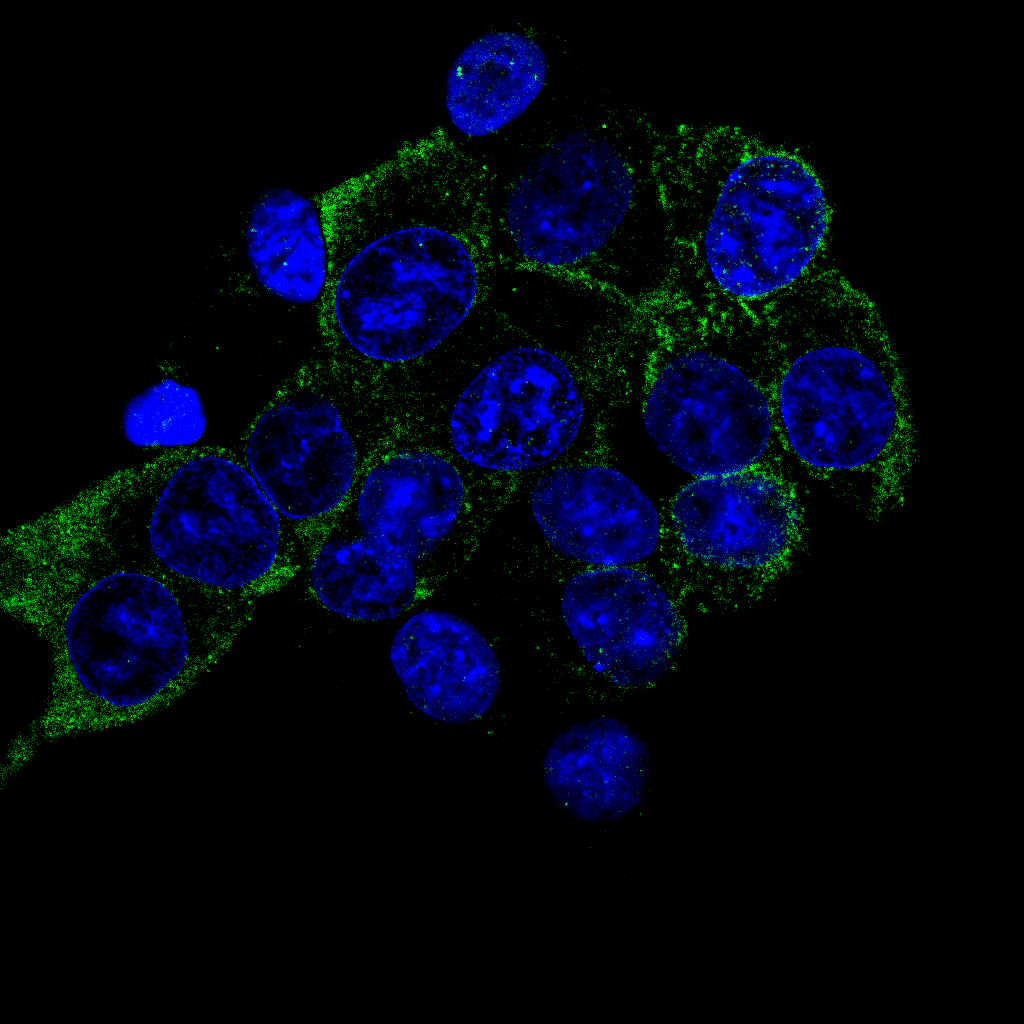

Supplement: Supplementary file 5 [file SupplementaryFile5.zip › 免疫荧光/6.13/SREBP1/lxj-BRE-SR1_0007.tif.frames/lxj-BRE-SR1_0007_T001.tif]

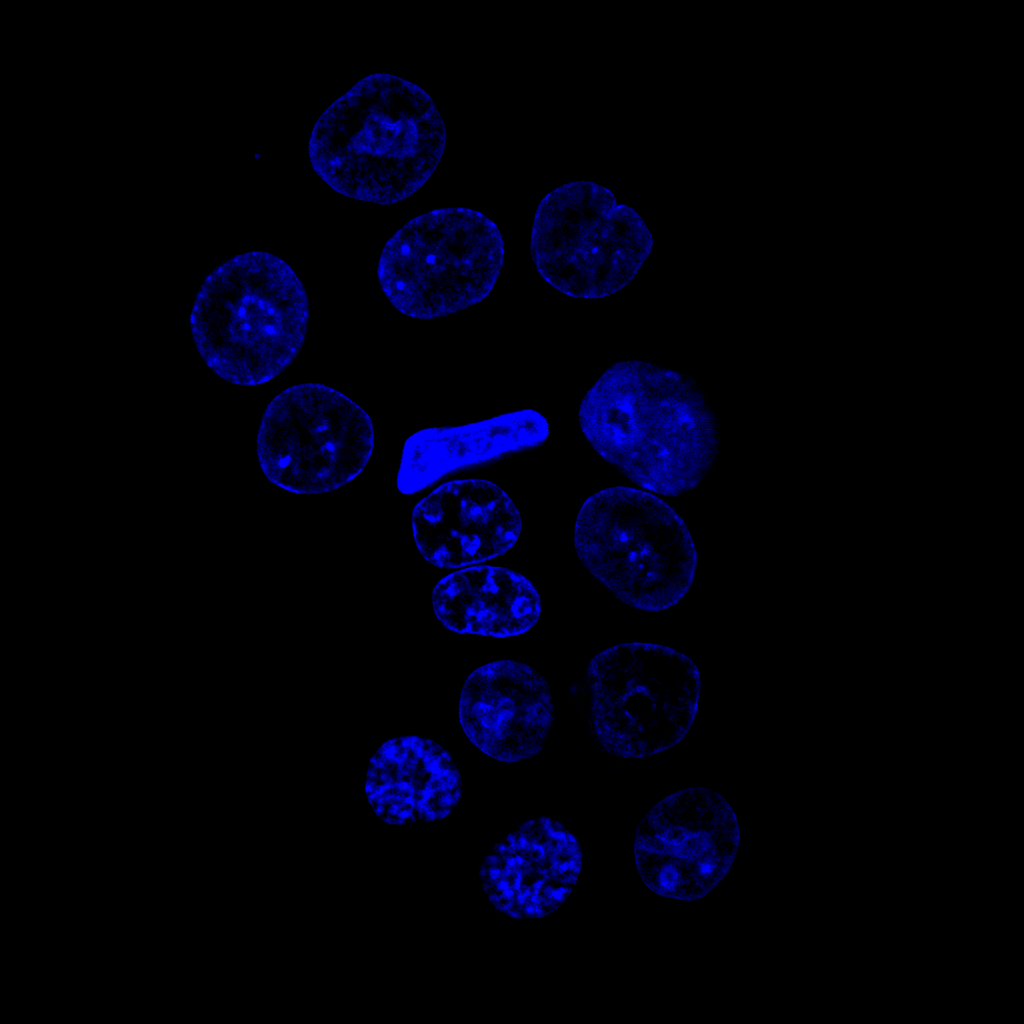

Supplement: Supplementary file 5 [file SupplementaryFile5.zip › 免疫荧光/6.13/SREBP1/lxj-BRE-SR1_0009.tif.frames/lxj-BRE-SR1_0009_C001T001.tif]

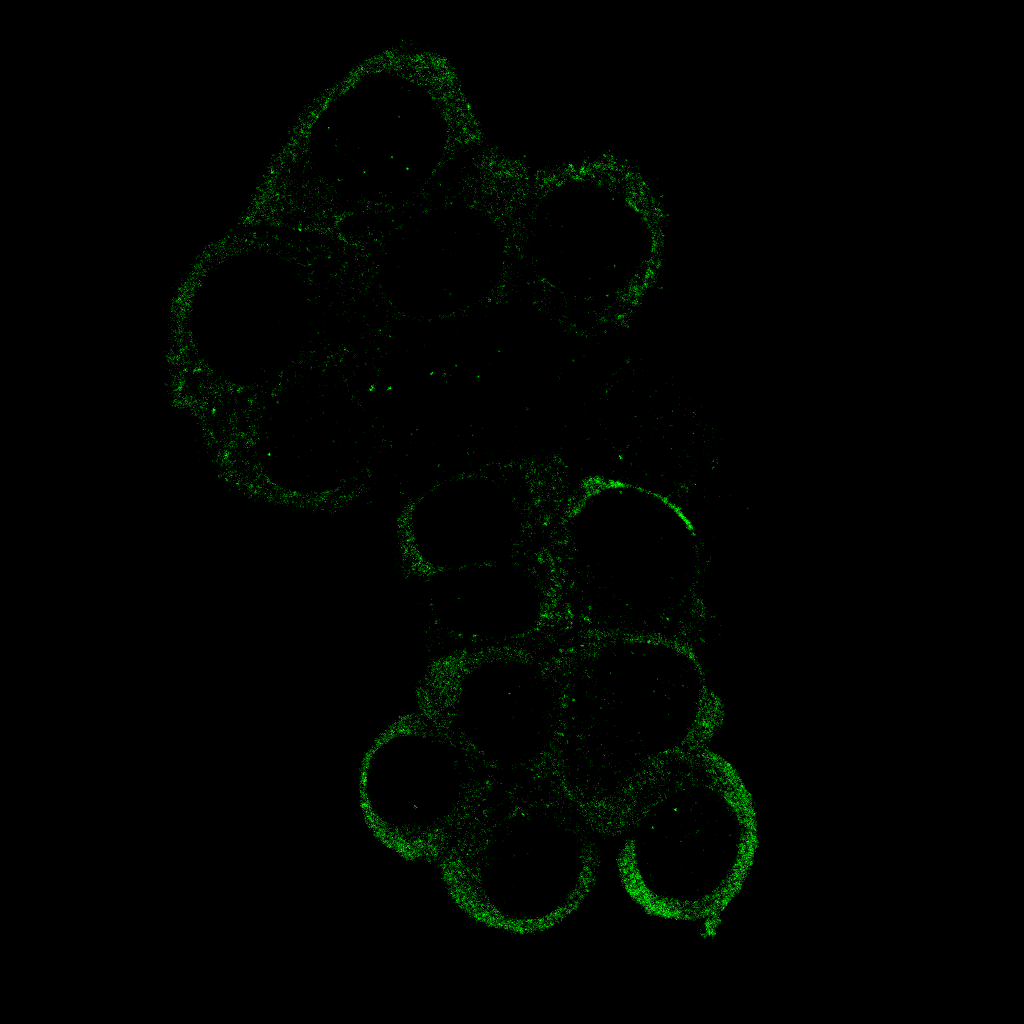

Supplement: Supplementary file 5 [file SupplementaryFile5.zip › 免疫荧光/6.13/SREBP1/lxj-BRE-SR1_0009.tif.frames/lxj-BRE-SR1_0009_C002T001.tif]

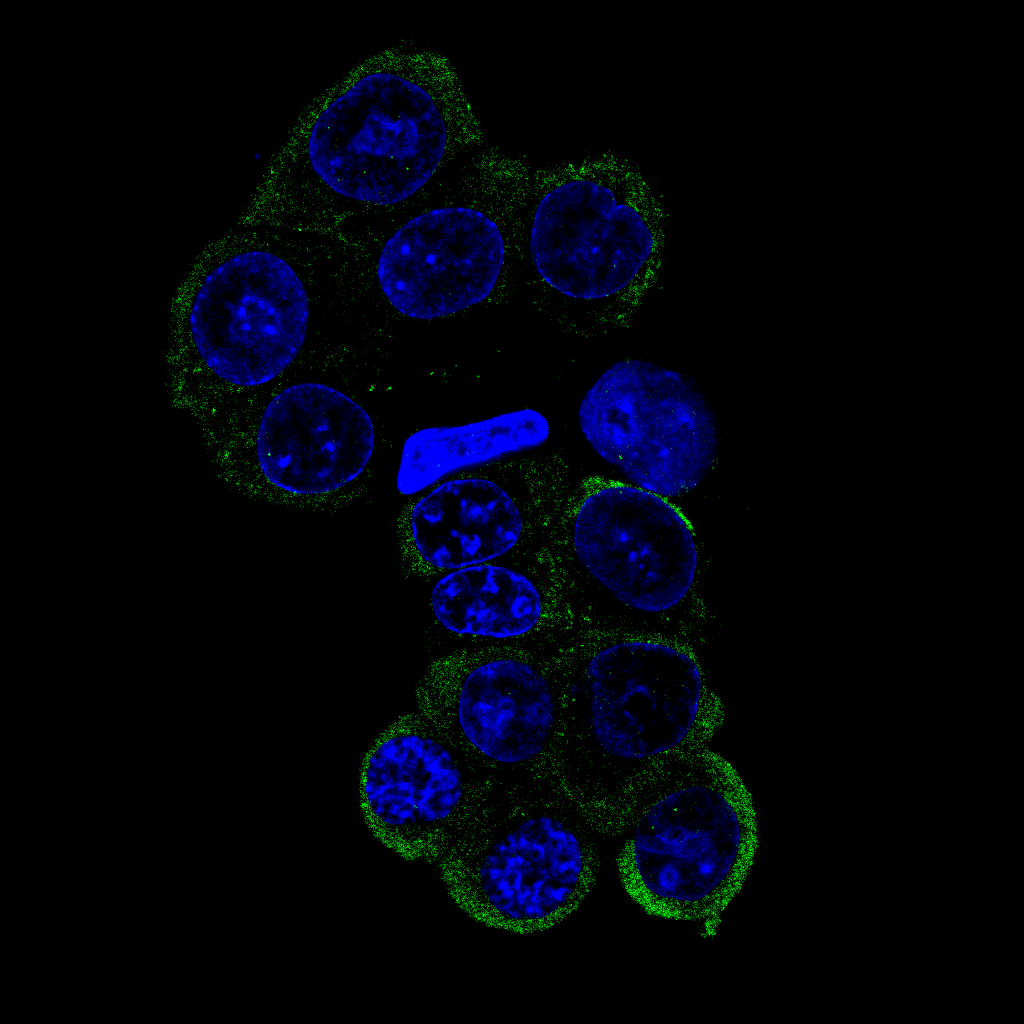

Supplement: Supplementary file 5 [file SupplementaryFile5.zip › 免疫荧光/6.13/SREBP1/lxj-BRE-SR1_0009.tif.frames/lxj-BRE-SR1_0009_T001.tif]

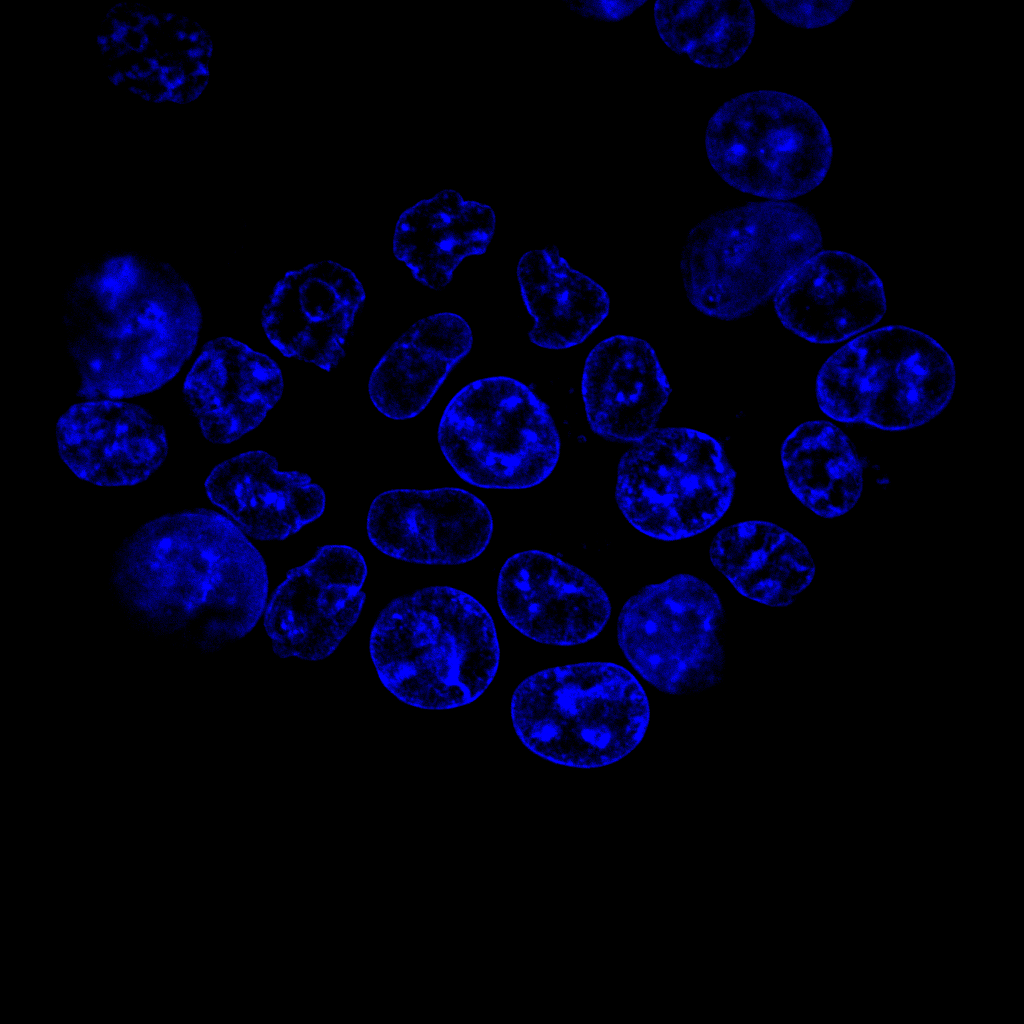

Supplement: Supplementary file 5 [file SupplementaryFile5.zip › 免疫荧光/6.13/SREBP1/lxj-BRE-SR1_0010.tif.frames/lxj-BRE-SR1_0010_C001T001.tif]

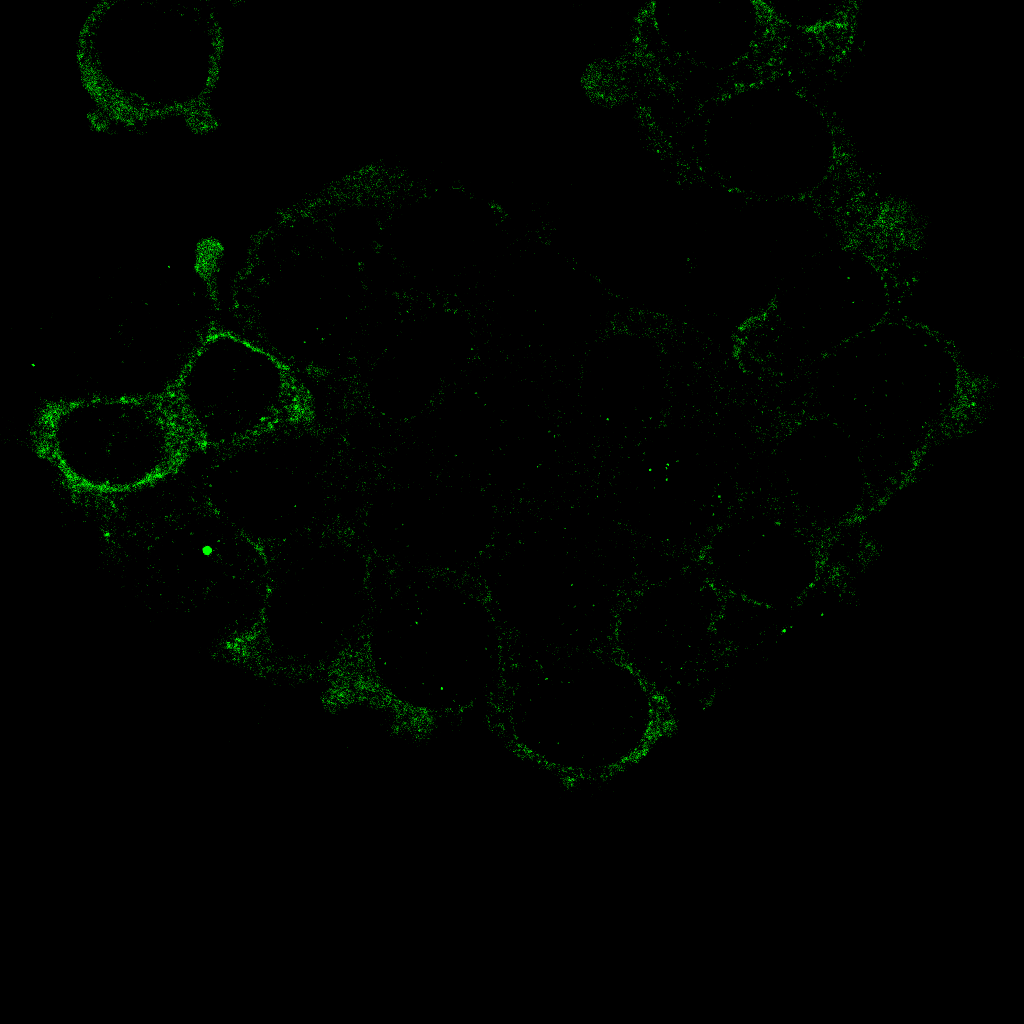

Supplement: Supplementary file 5 [file SupplementaryFile5.zip › 免疫荧光/6.13/SREBP1/lxj-BRE-SR1_0010.tif.frames/lxj-BRE-SR1_0010_C002T001.tif]

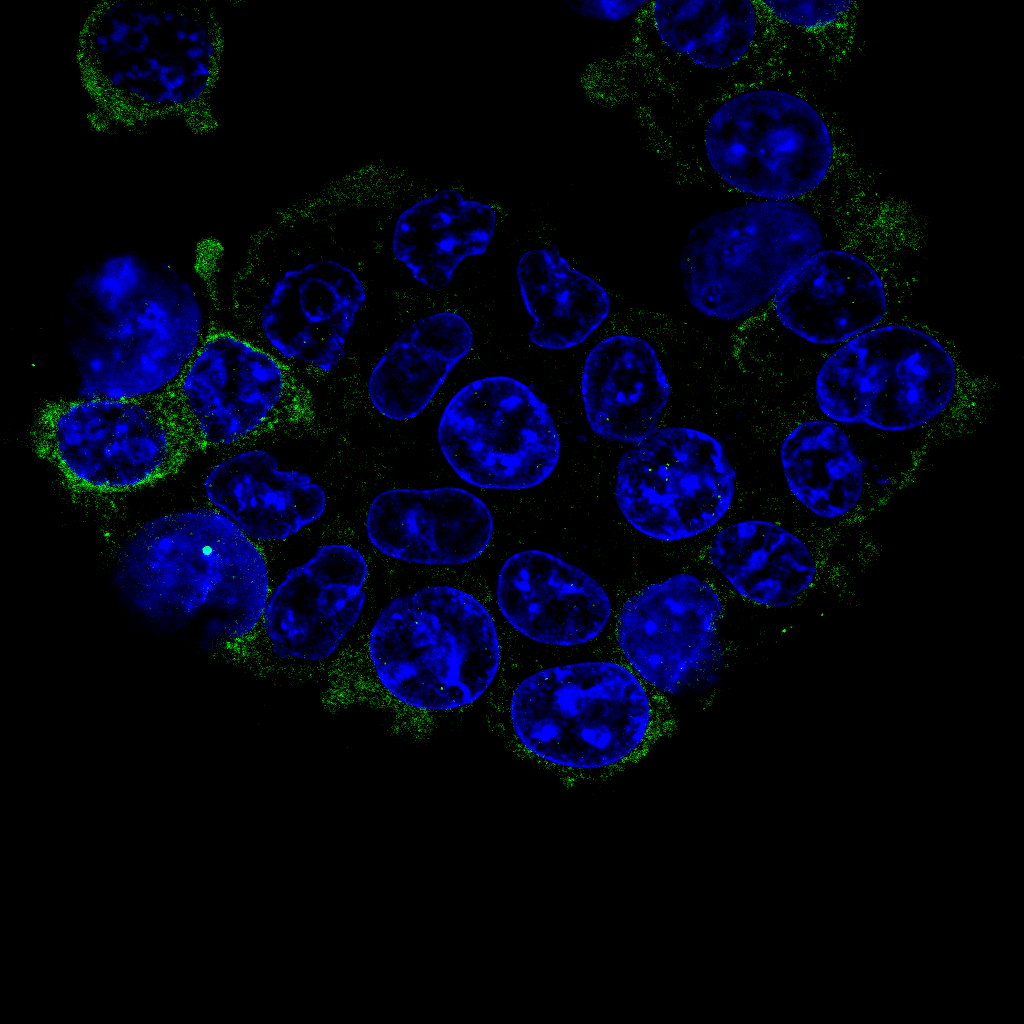

Supplement: Supplementary file 5 [file SupplementaryFile5.zip › 免疫荧光/6.13/SREBP1/lxj-BRE-SR1_0010.tif.frames/lxj-BRE-SR1_0010_T001.tif]

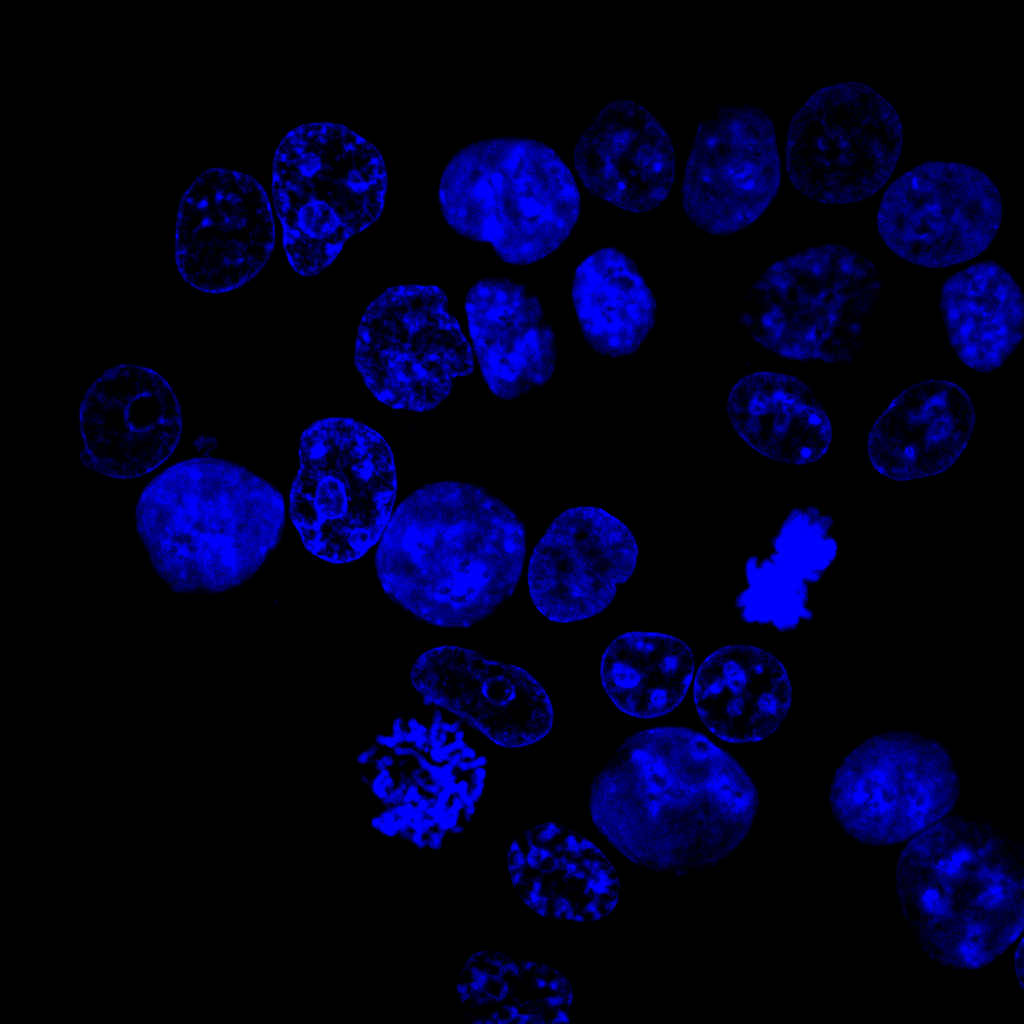

Supplement: Supplementary file 5 [file SupplementaryFile5.zip › 免疫荧光/6.13/SREBP1/lxj-BRE-SR1_0011.tif.frames/lxj-BRE-SR1_0011_C001T001.tif]

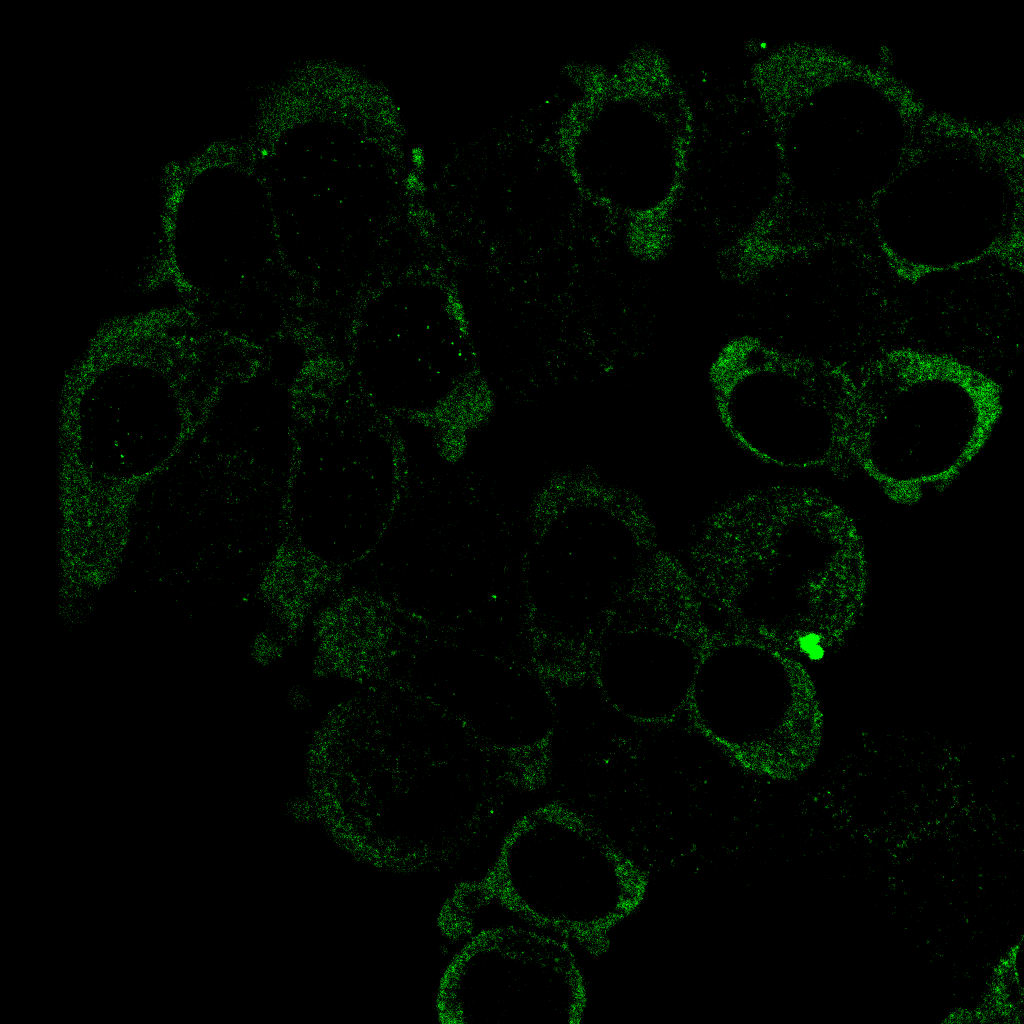

Supplement: Supplementary file 5 [file SupplementaryFile5.zip › 免疫荧光/6.13/SREBP1/lxj-BRE-SR1_0011.tif.frames/lxj-BRE-SR1_0011_C002T001.tif]

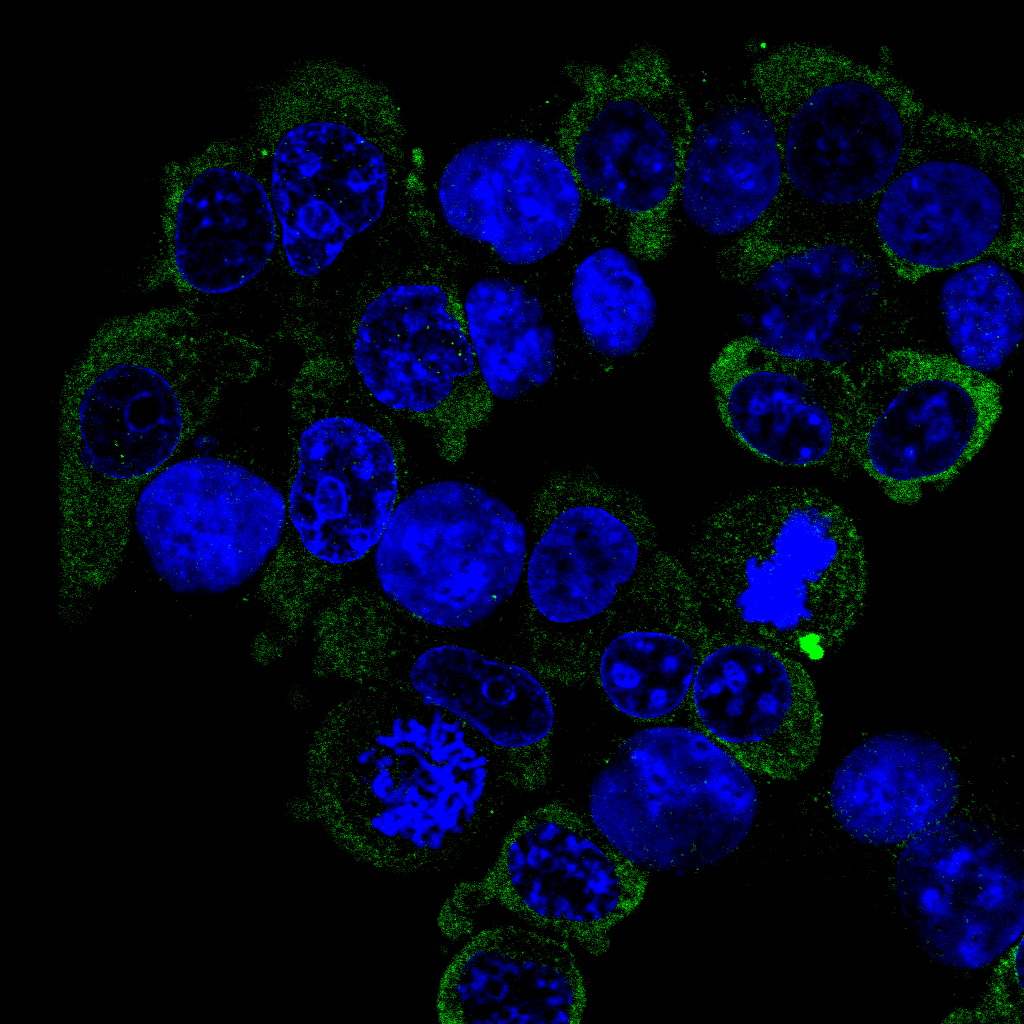

Supplement: Supplementary file 5 [file SupplementaryFile5.zip › 免疫荧光/6.13/SREBP1/lxj-BRE-SR1_0011.tif.frames/lxj-BRE-SR1_0011_T001.tif]

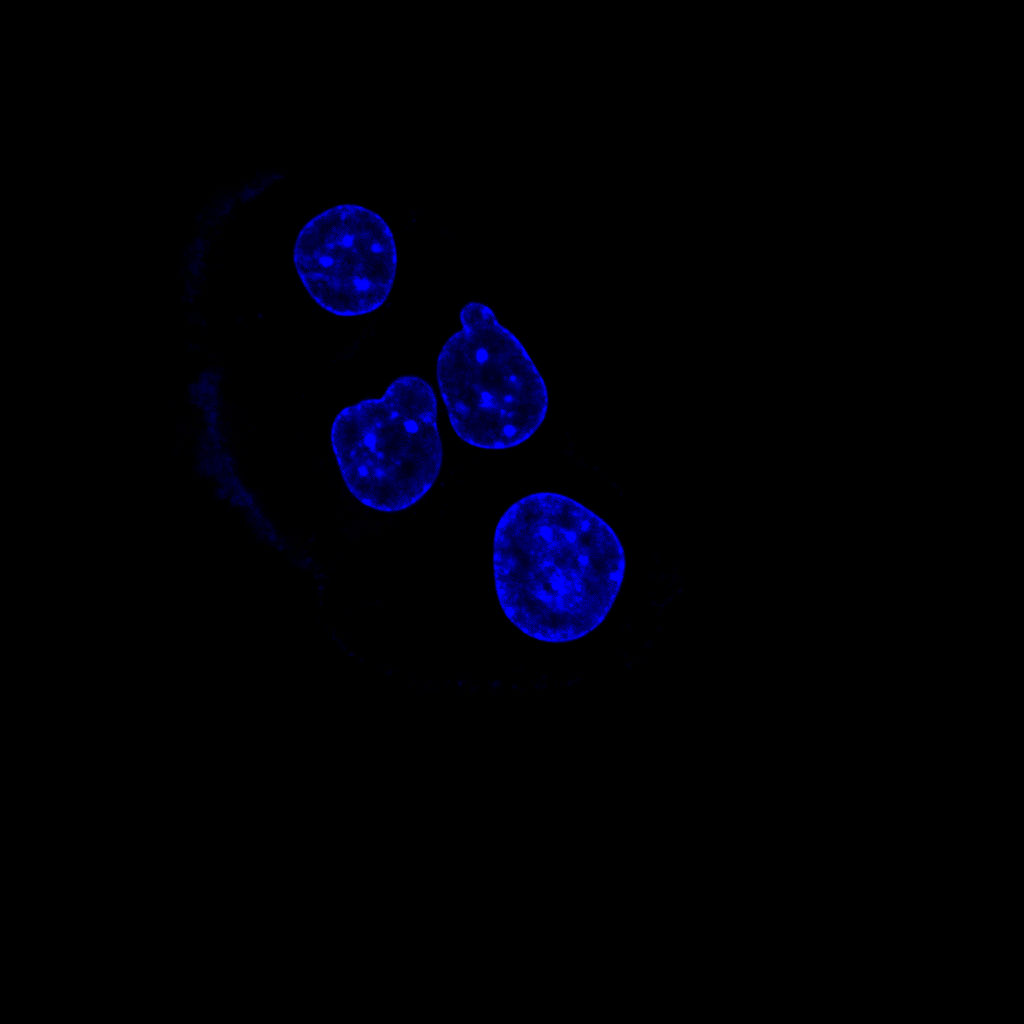

Supplement: Supplementary file 5 [file SupplementaryFile5.zip › 免疫荧光/6.13/SREBP1/lxj-BRE-SR1_0012.tif.frames/lxj-BRE-SR1_0012_C001T001.tif]

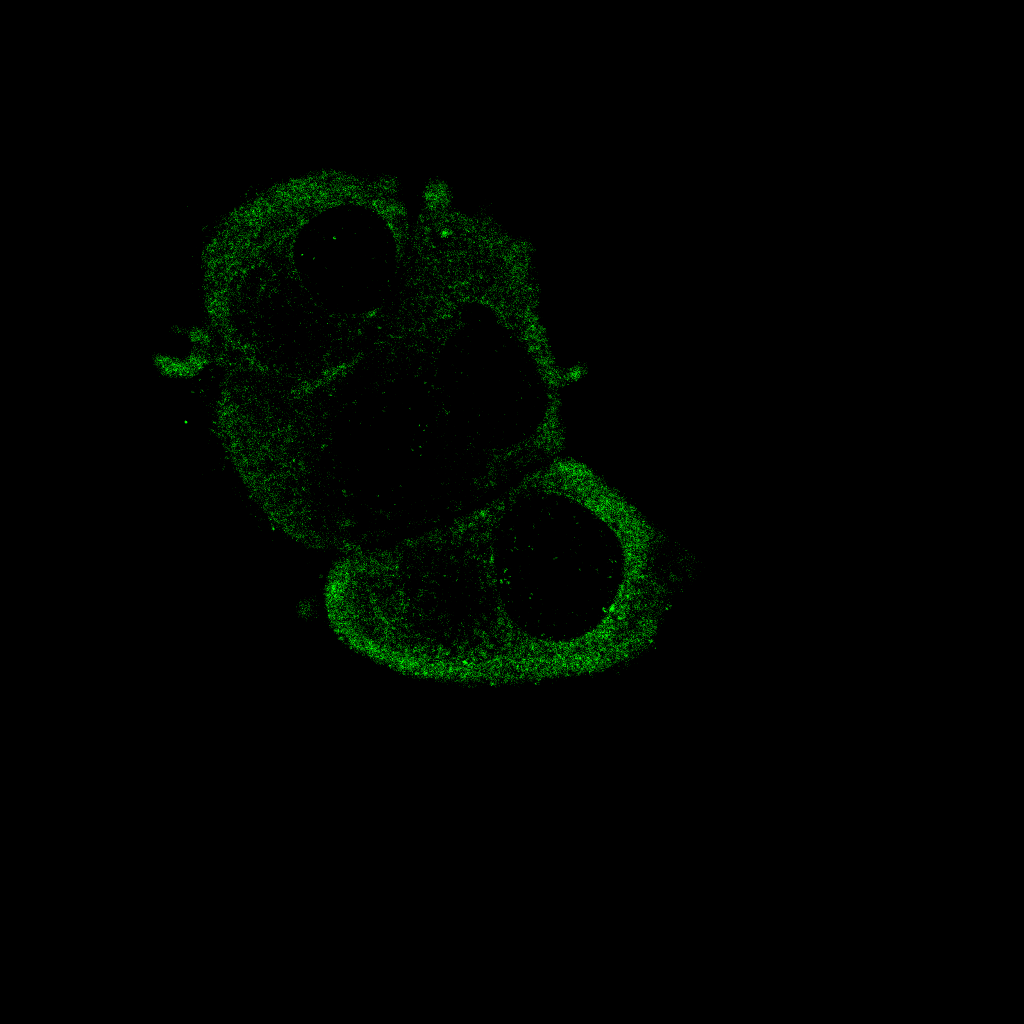

Supplement: Supplementary file 5 [file SupplementaryFile5.zip › 免疫荧光/6.13/SREBP1/lxj-BRE-SR1_0012.tif.frames/lxj-BRE-SR1_0012_C002T001.tif]

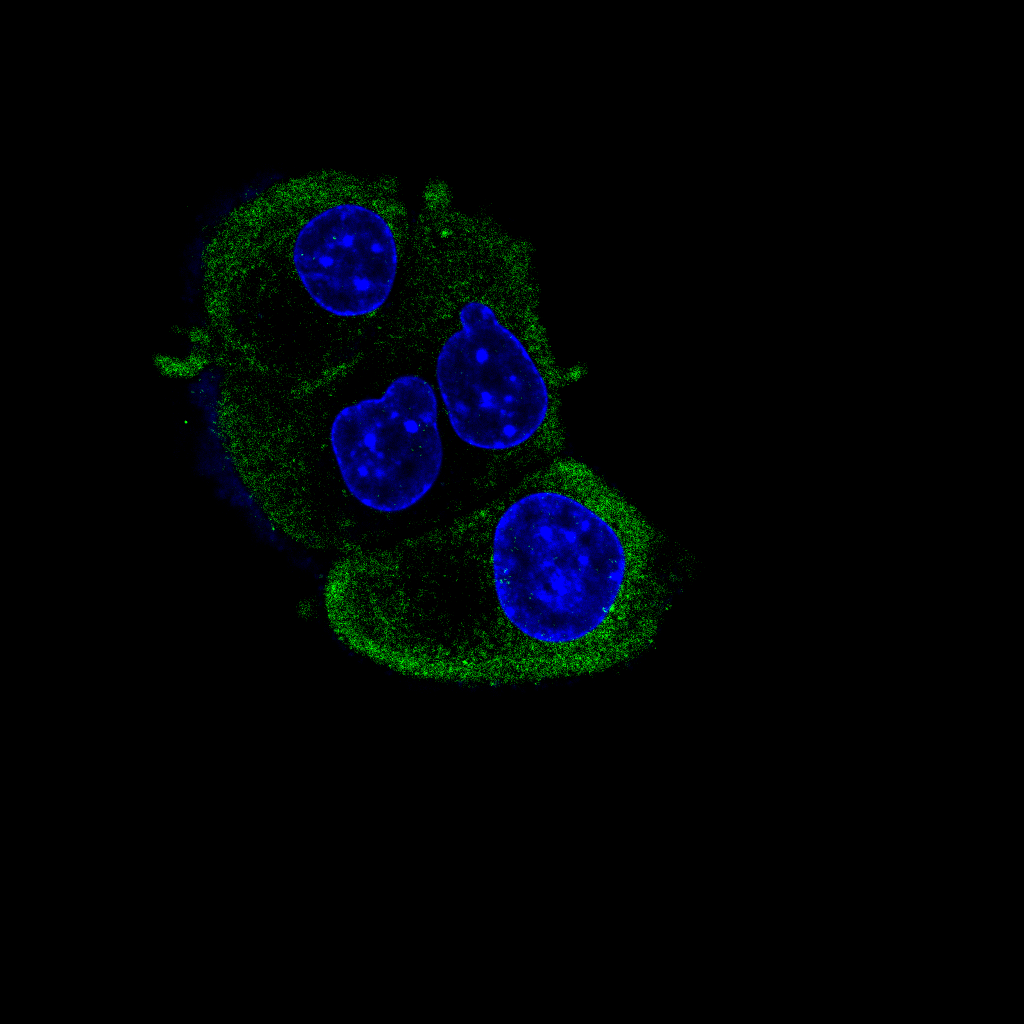

Supplement: Supplementary file 5 [file SupplementaryFile5.zip › 免疫荧光/6.13/SREBP1/lxj-BRE-SR1_0012.tif.frames/lxj-BRE-SR1_0012_T001.tif]

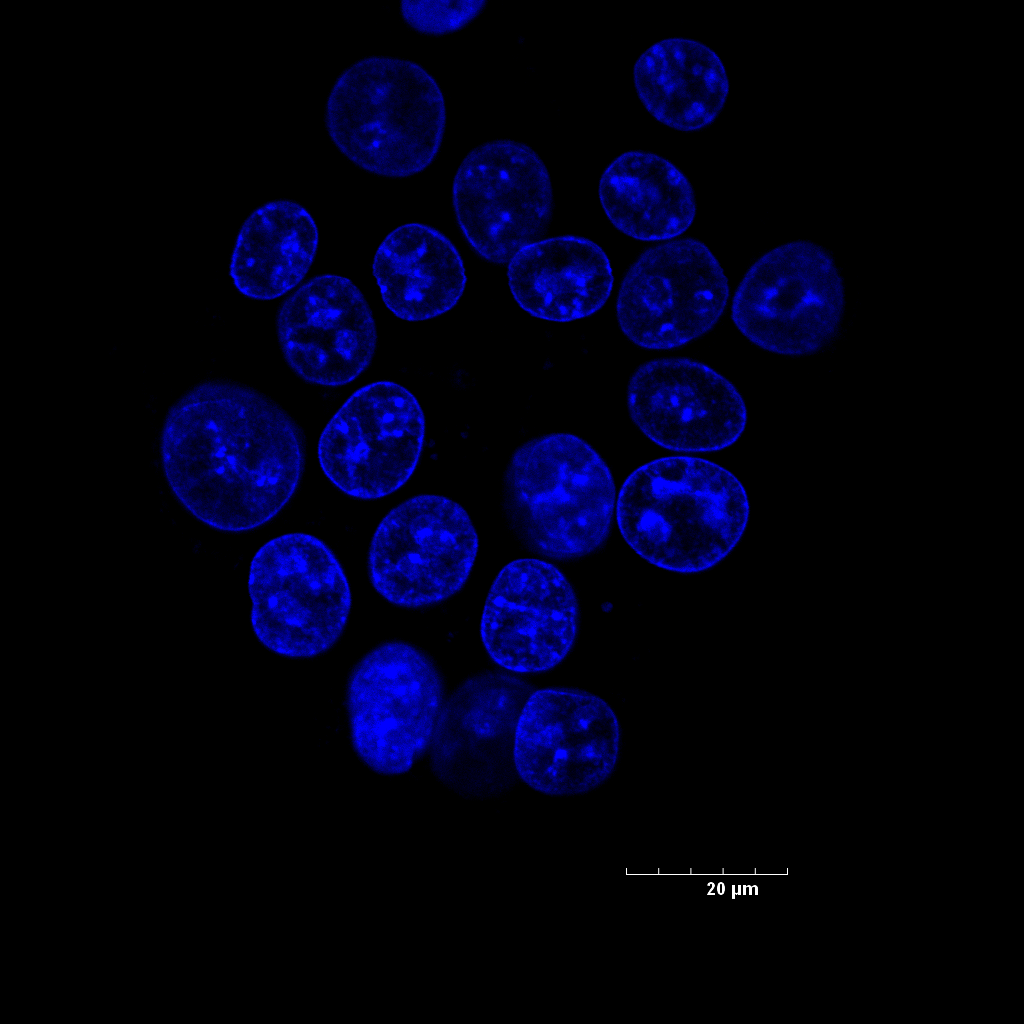

Supplement: Supplementary file 5 [file SupplementaryFile5.zip › 免疫荧光/6.13/SREBP1/lxj-BRE-SR1_0013.tif.frames/lxj-BRE-SR1_0013_C001T001.tif]

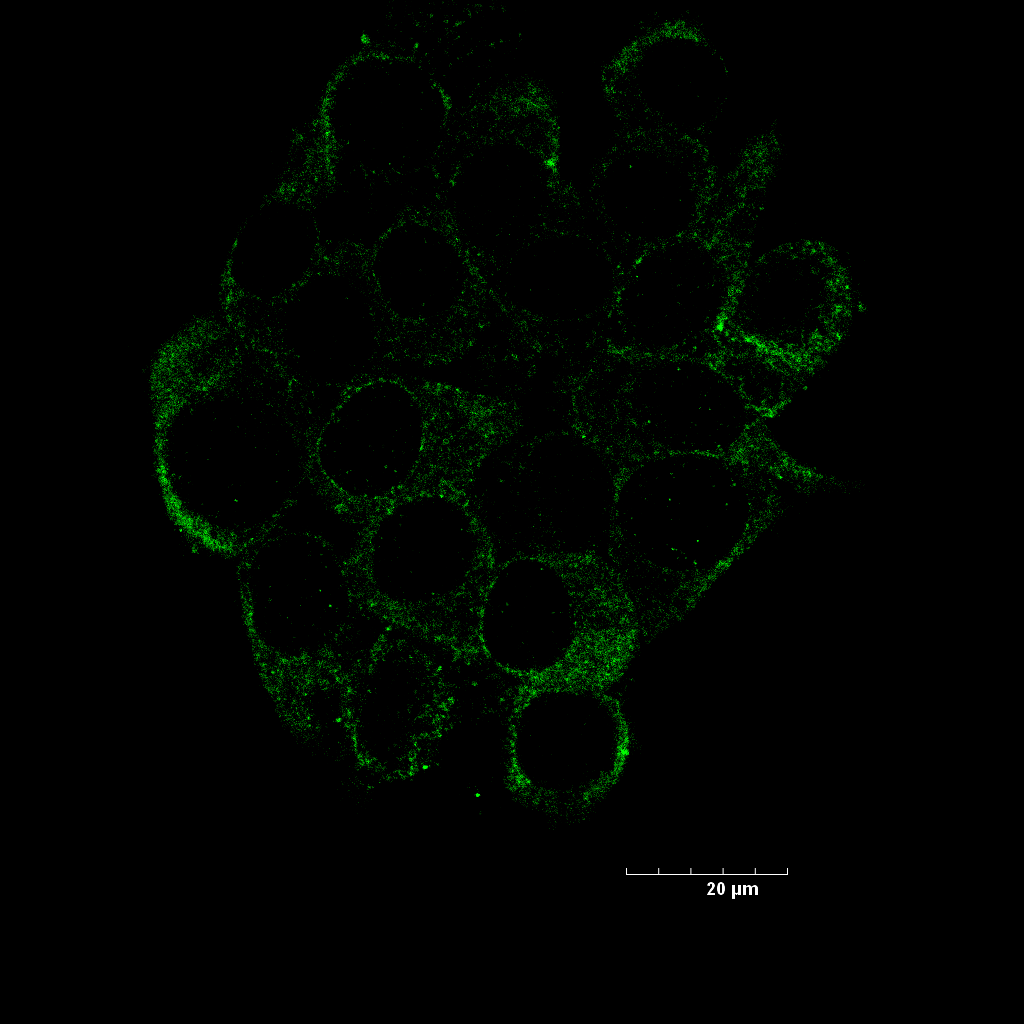

Supplement: Supplementary file 5 [file SupplementaryFile5.zip › 免疫荧光/6.13/SREBP1/lxj-BRE-SR1_0013.tif.frames/lxj-BRE-SR1_0013_C002T001.tif]

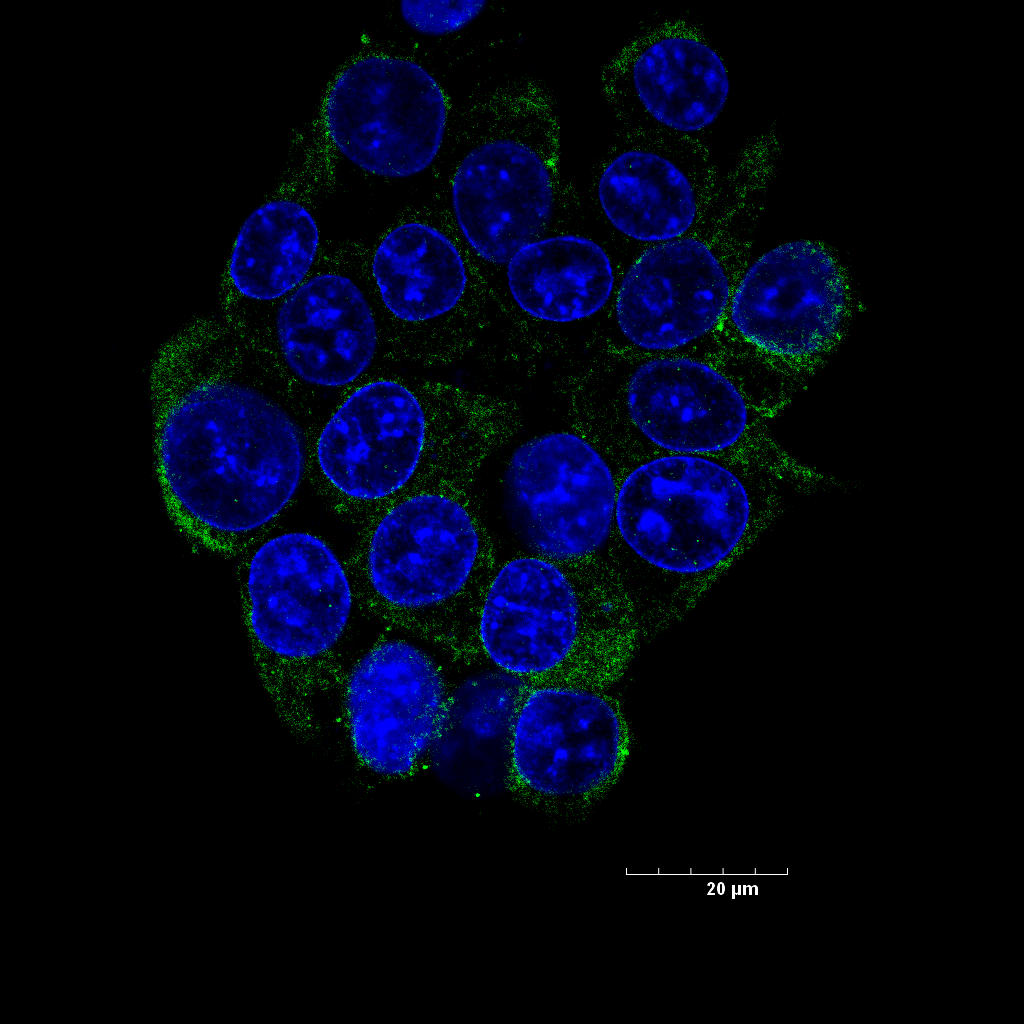

Supplement: Supplementary file 5 [file SupplementaryFile5.zip › 免疫荧光/6.13/SREBP1/lxj-BRE-SR1_0013.tif.frames/lxj-BRE-SR1_0013_T001.tif]

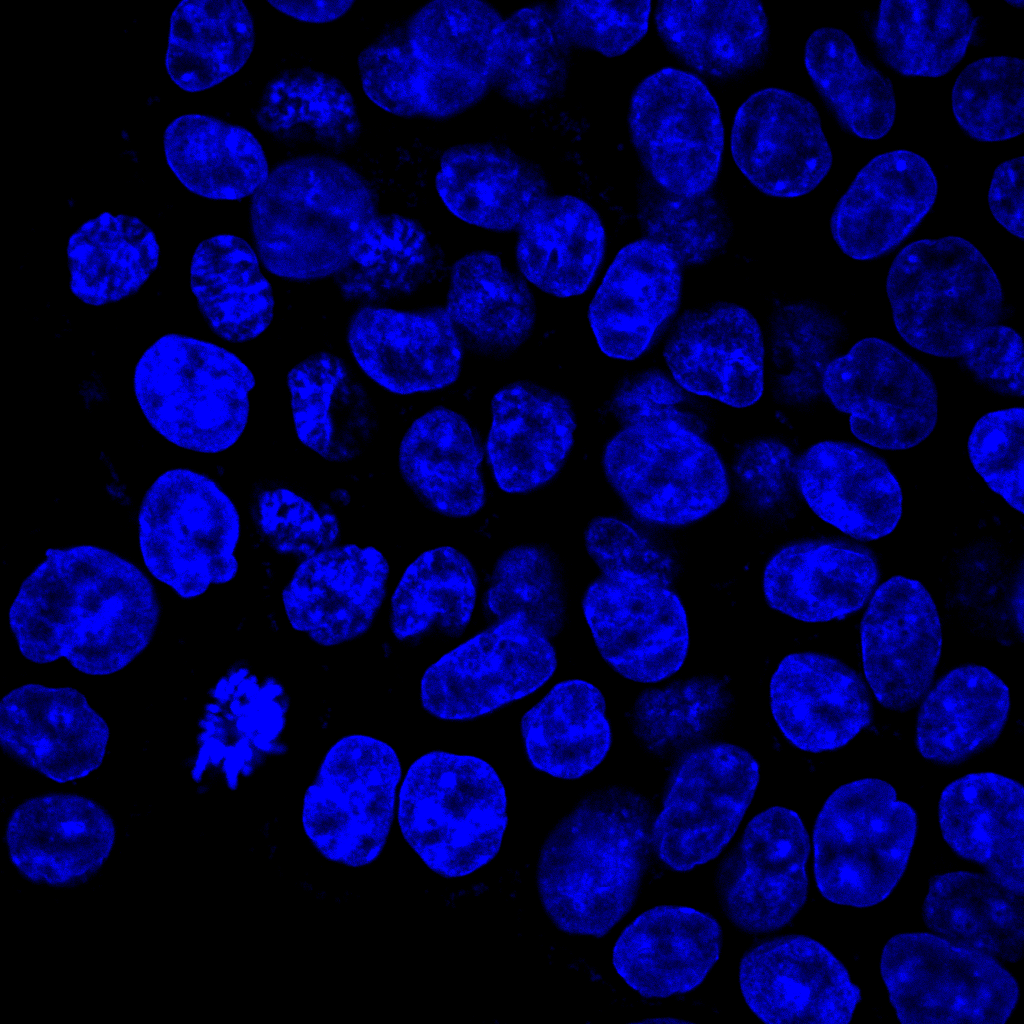

Supplement: Supplementary file 5 [file SupplementaryFile5.zip › 免疫荧光/6.13/SREBP1/lxj-NC-SR1_0009.tif.frames/lxj-NC-SR1_0009_C001T001.tif]

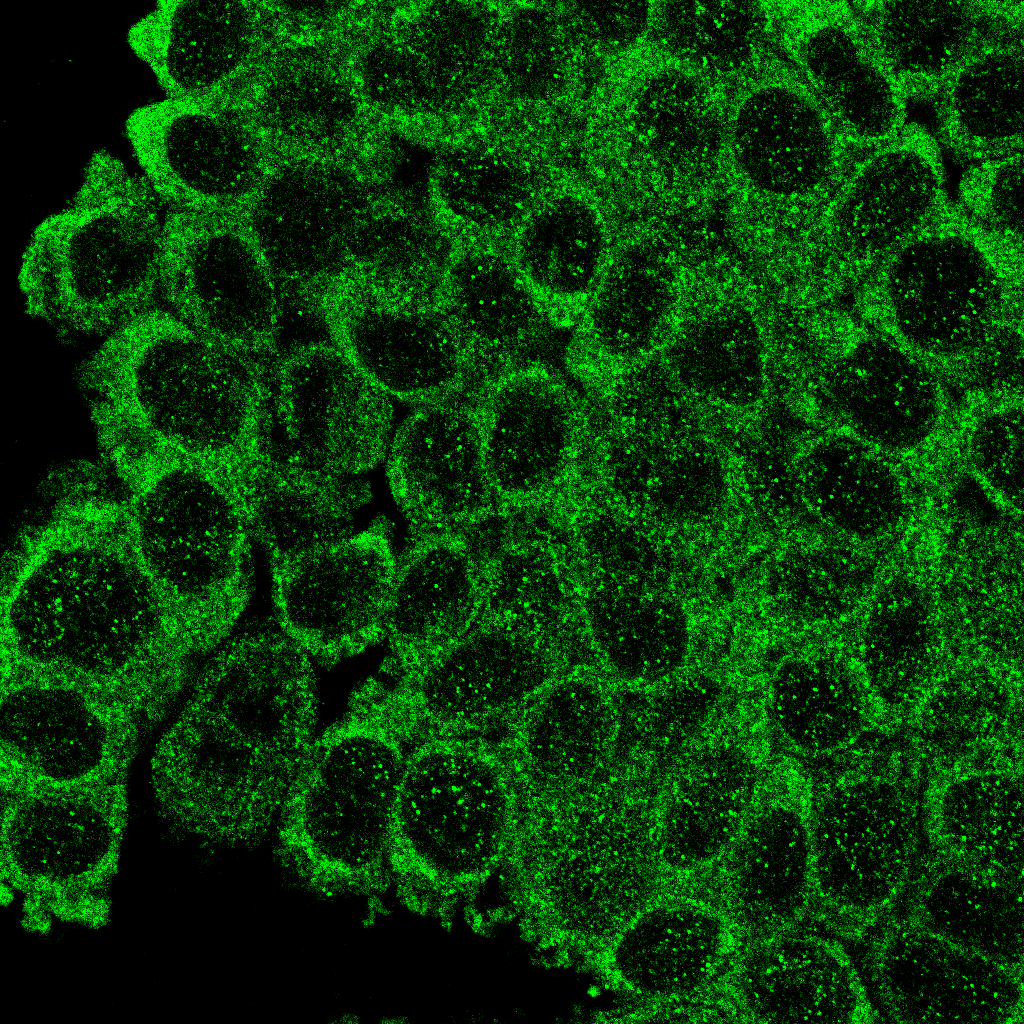

Supplement: Supplementary file 5 [file SupplementaryFile5.zip › 免疫荧光/6.13/SREBP1/lxj-NC-SR1_0009.tif.frames/lxj-NC-SR1_0009_C002T001.tif]

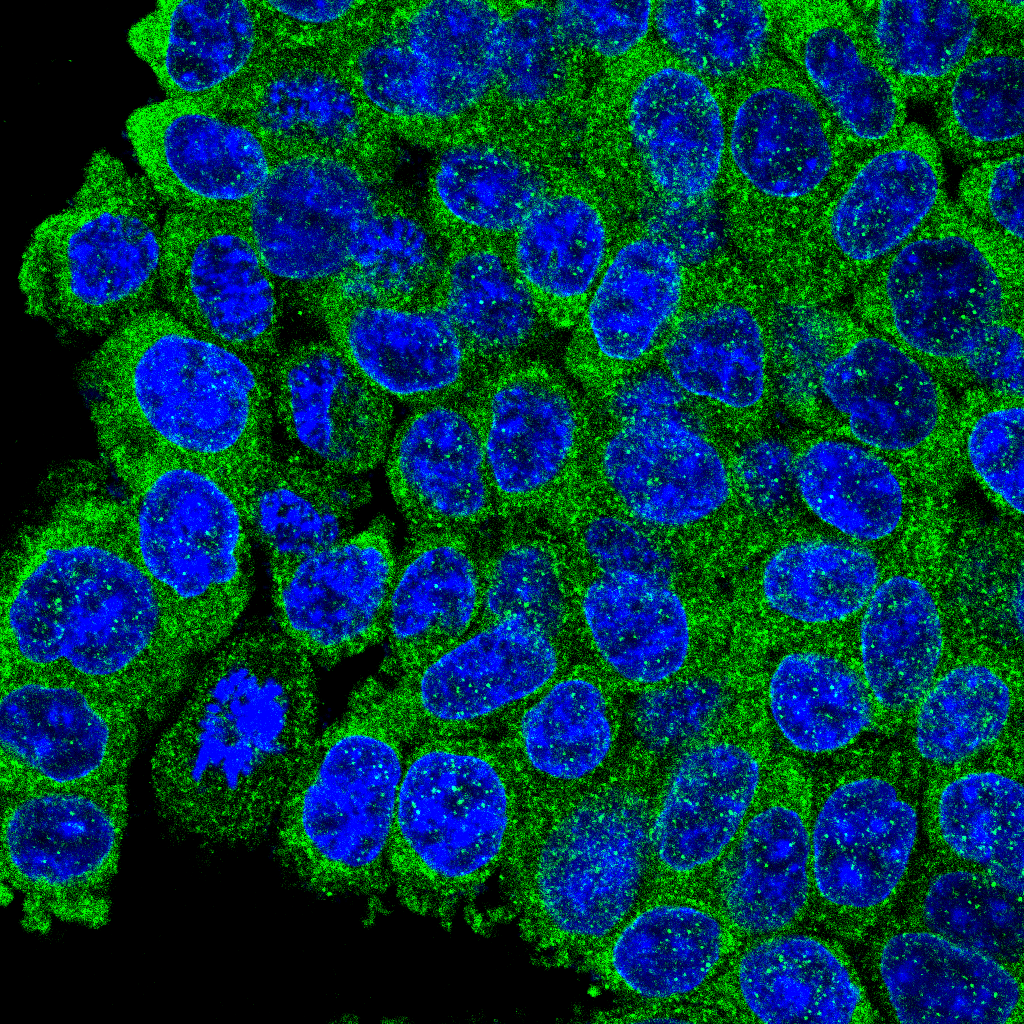

Supplement: Supplementary file 5 [file SupplementaryFile5.zip › 免疫荧光/6.13/SREBP1/lxj-NC-SR1_0009.tif.frames/lxj-NC-SR1_0009_T001.tif]

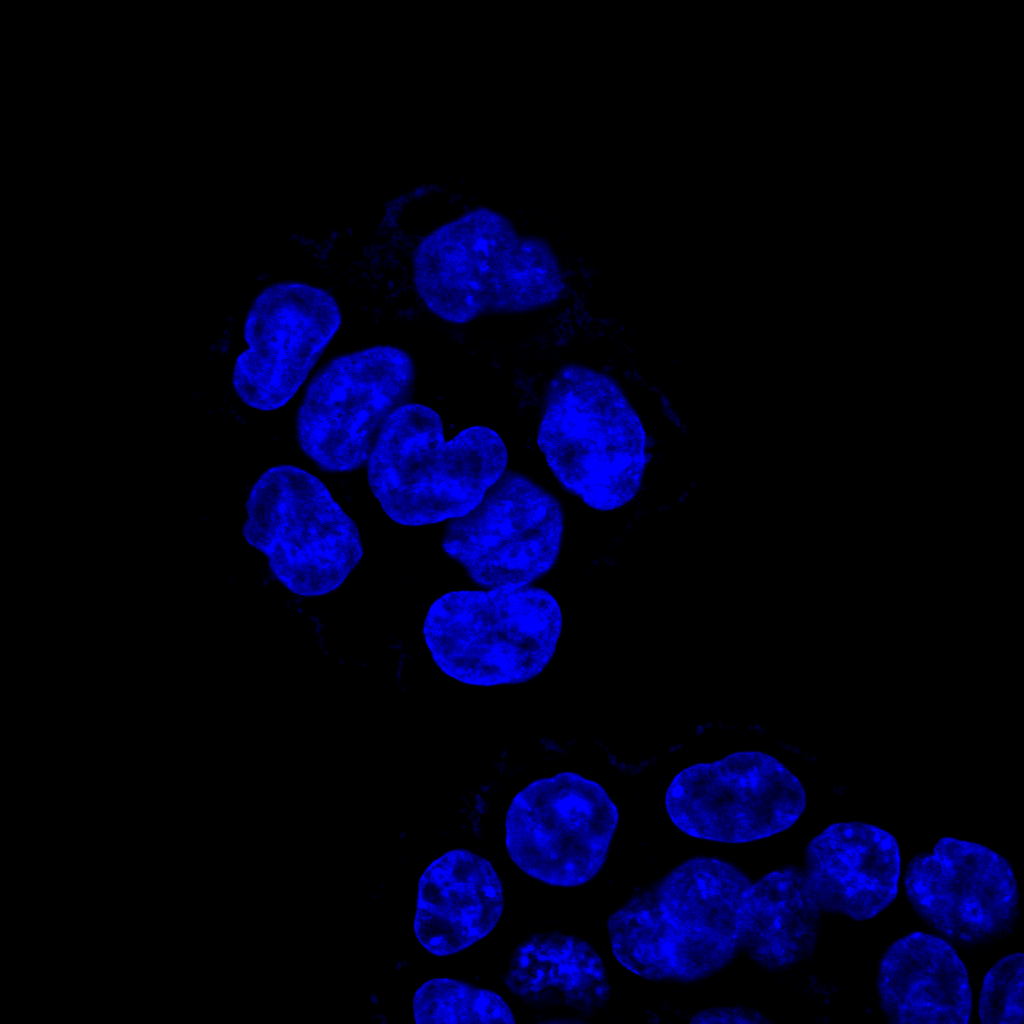

Supplement: Supplementary file 5 [file SupplementaryFile5.zip › 免疫荧光/6.13/SREBP1/lxj-NC-SR1_0010.tif.frames/lxj-NC-SR1_0010_C001T001.tif]

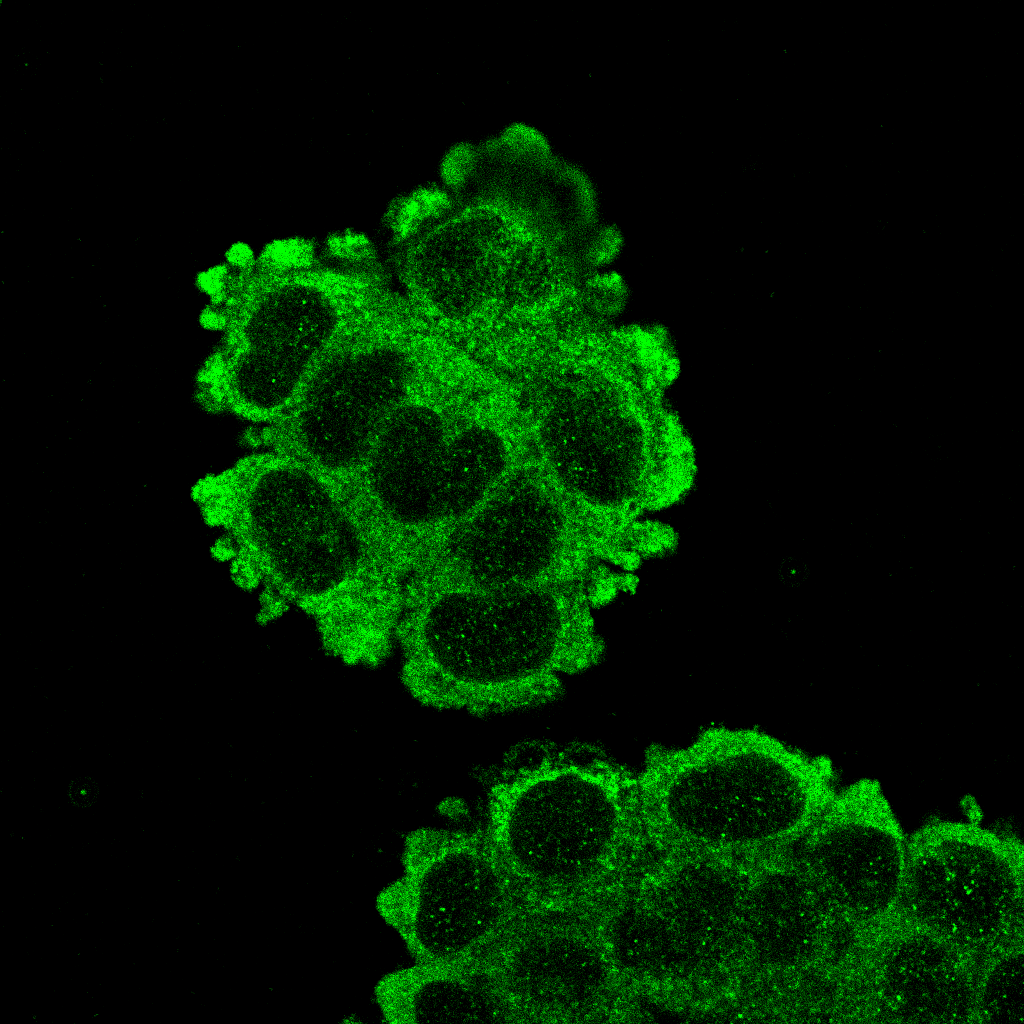

Supplement: Supplementary file 5 [file SupplementaryFile5.zip › 免疫荧光/6.13/SREBP1/lxj-NC-SR1_0010.tif.frames/lxj-NC-SR1_0010_C002T001.tif]

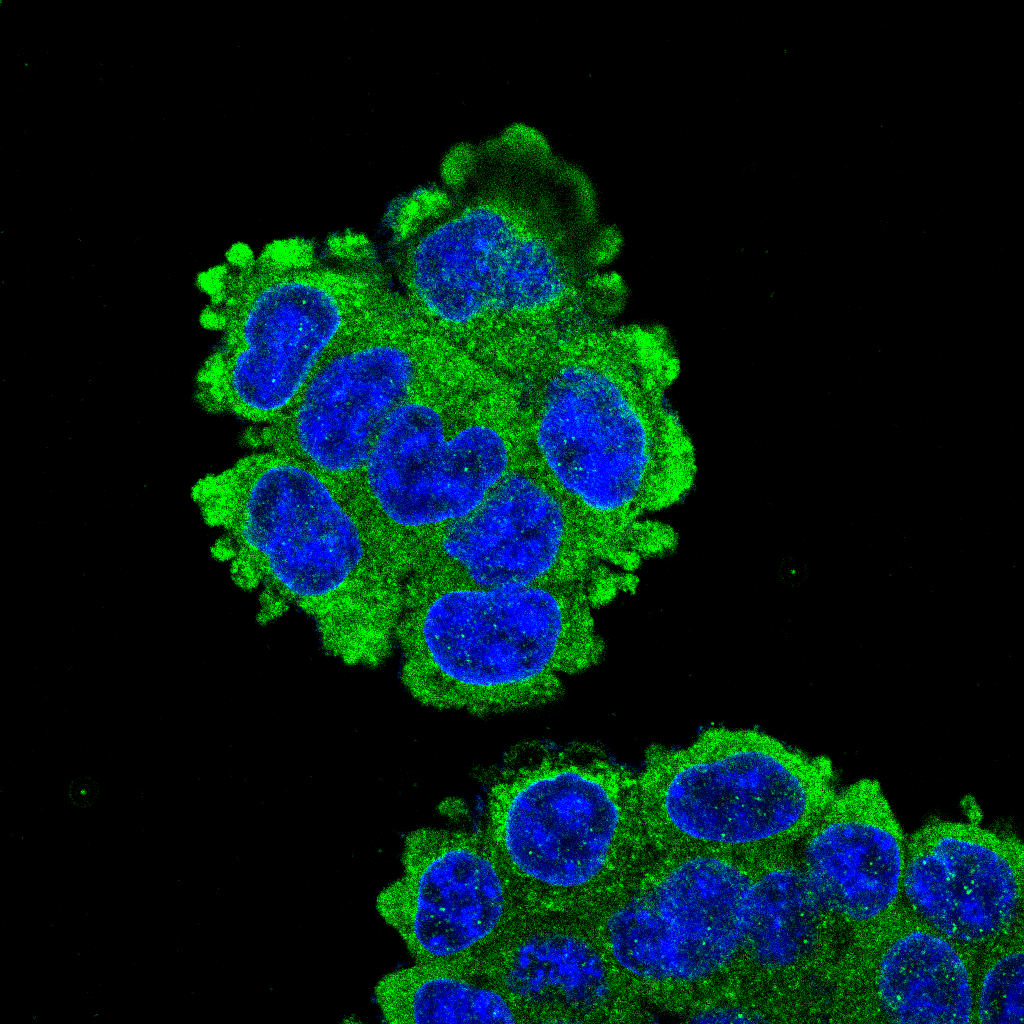

Supplement: Supplementary file 5 [file SupplementaryFile5.zip › 免疫荧光/6.13/SREBP1/lxj-NC-SR1_0010.tif.frames/lxj-NC-SR1_0010_T001.tif]

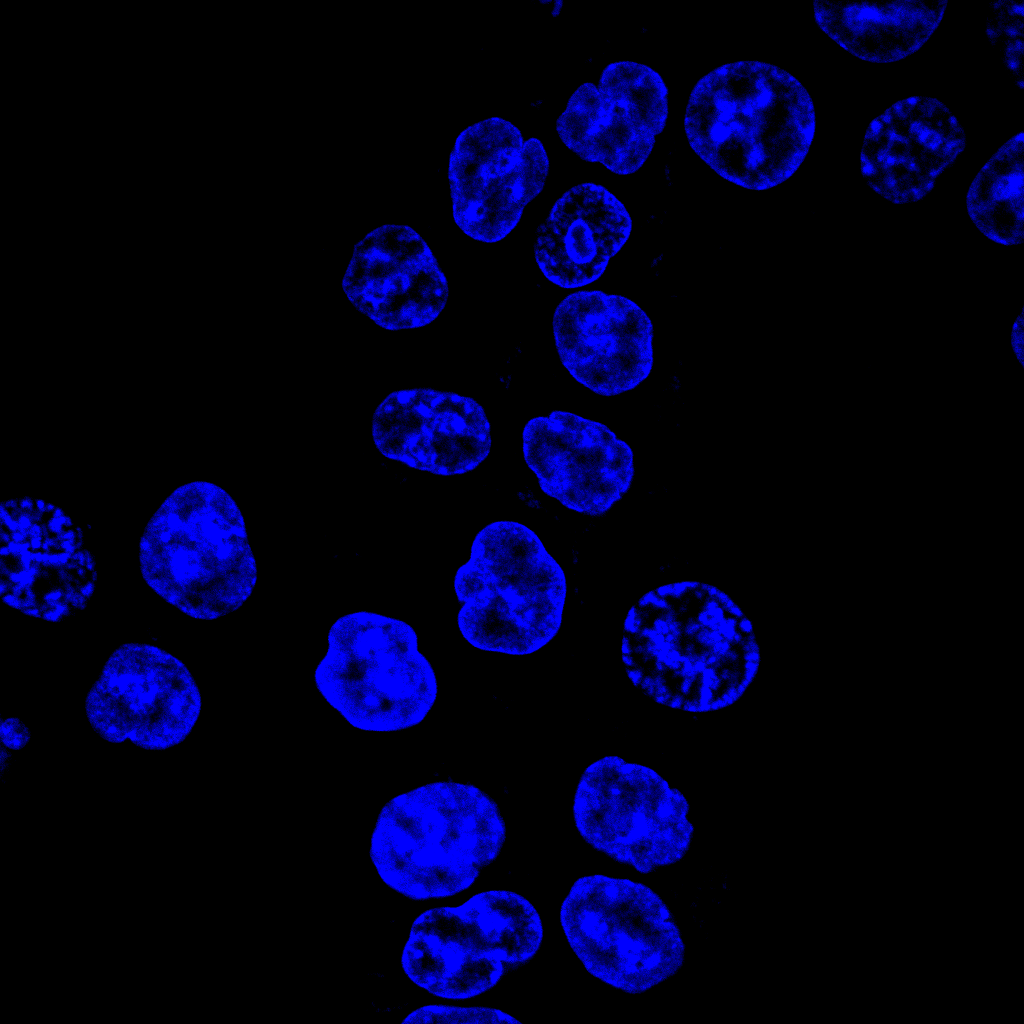

Supplement: Supplementary file 5 [file SupplementaryFile5.zip › 免疫荧光/6.13/SREBP1/lxj-NC-SR1_0011.tif.frames/lxj-NC-SR1_0011_C001T001.tif]

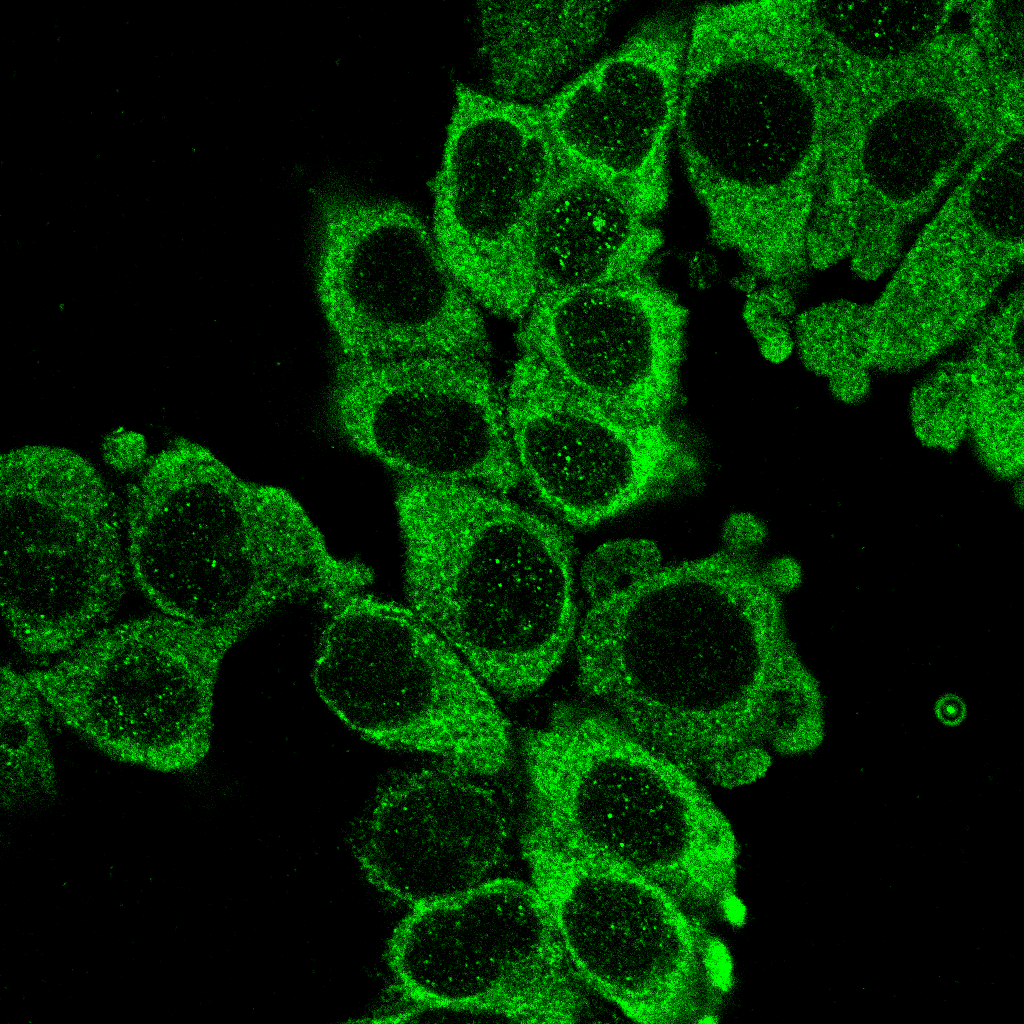

Supplement: Supplementary file 5 [file SupplementaryFile5.zip › 免疫荧光/6.13/SREBP1/lxj-NC-SR1_0011.tif.frames/lxj-NC-SR1_0011_C002T001.tif]

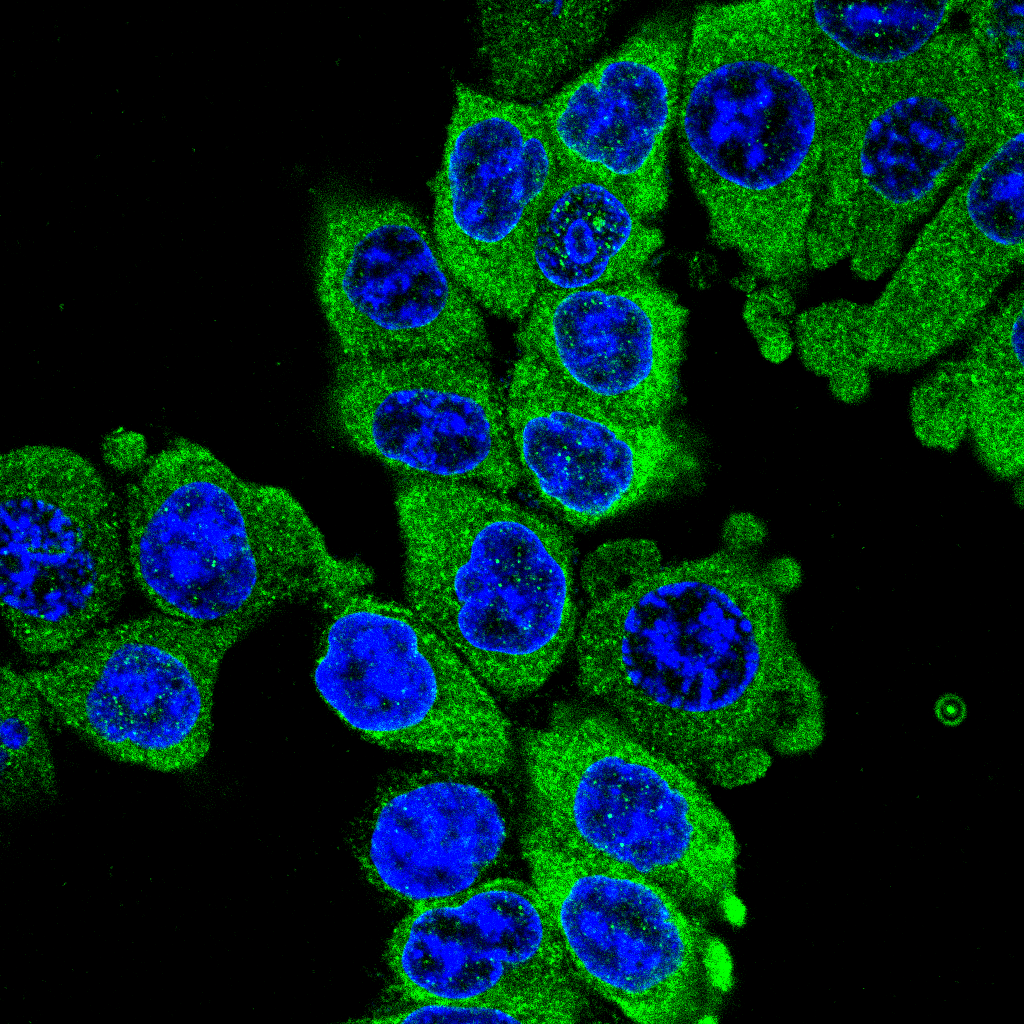

Supplement: Supplementary file 5 [file SupplementaryFile5.zip › 免疫荧光/6.13/SREBP1/lxj-NC-SR1_0011.tif.frames/lxj-NC-SR1_0011_T001.tif]

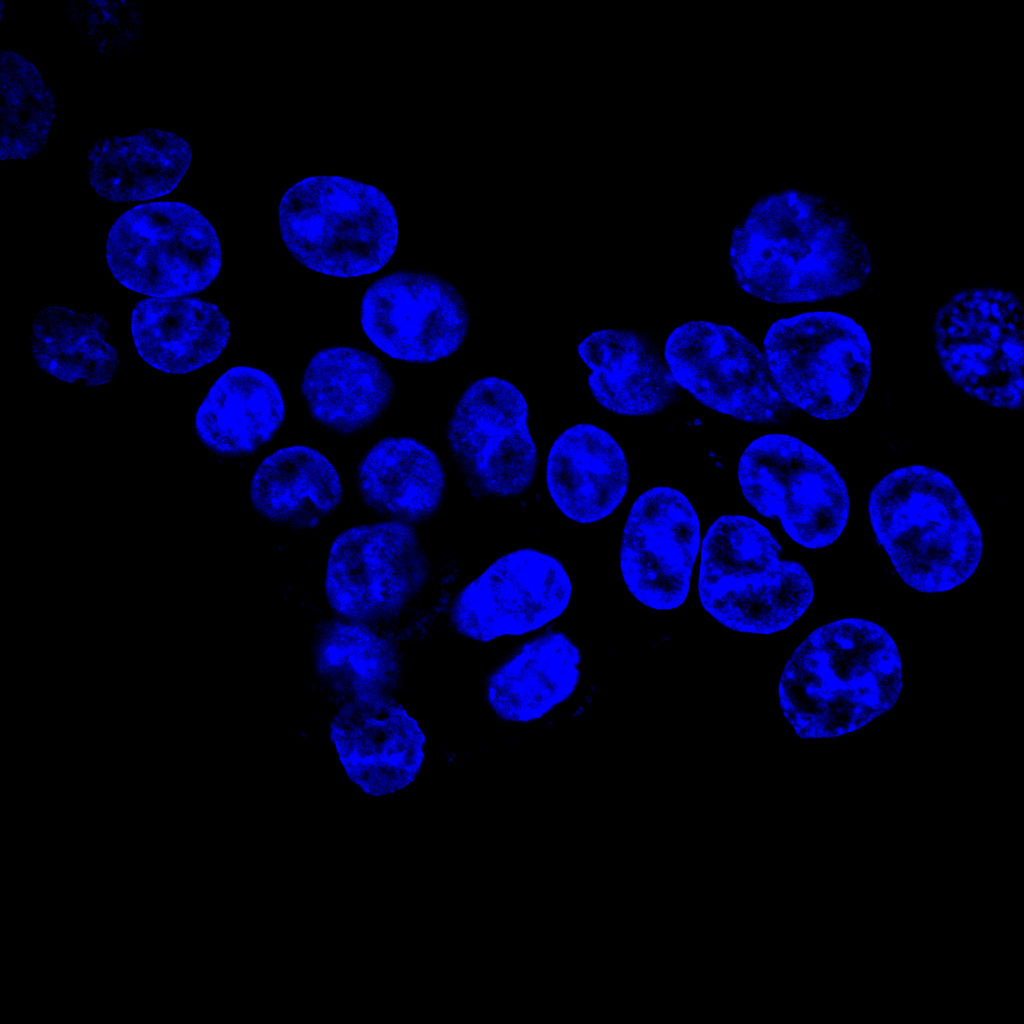

Supplement: Supplementary file 5 [file SupplementaryFile5.zip › 免疫荧光/6.13/SREBP1/lxj-NC-SR1_0012.tif.frames/lxj-NC-SR1_0012_C001T001.tif]

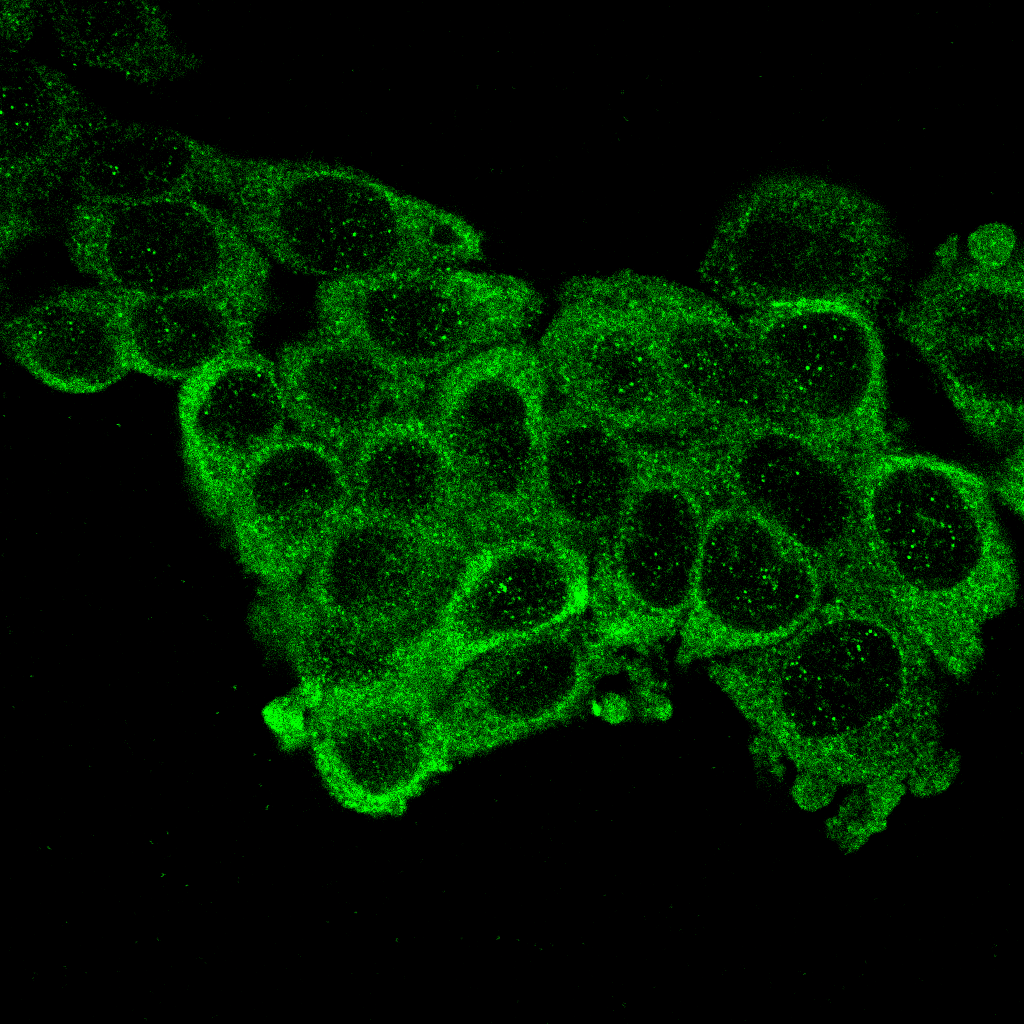

Supplement: Supplementary file 5 [file SupplementaryFile5.zip › 免疫荧光/6.13/SREBP1/lxj-NC-SR1_0012.tif.frames/lxj-NC-SR1_0012_C002T001.tif]

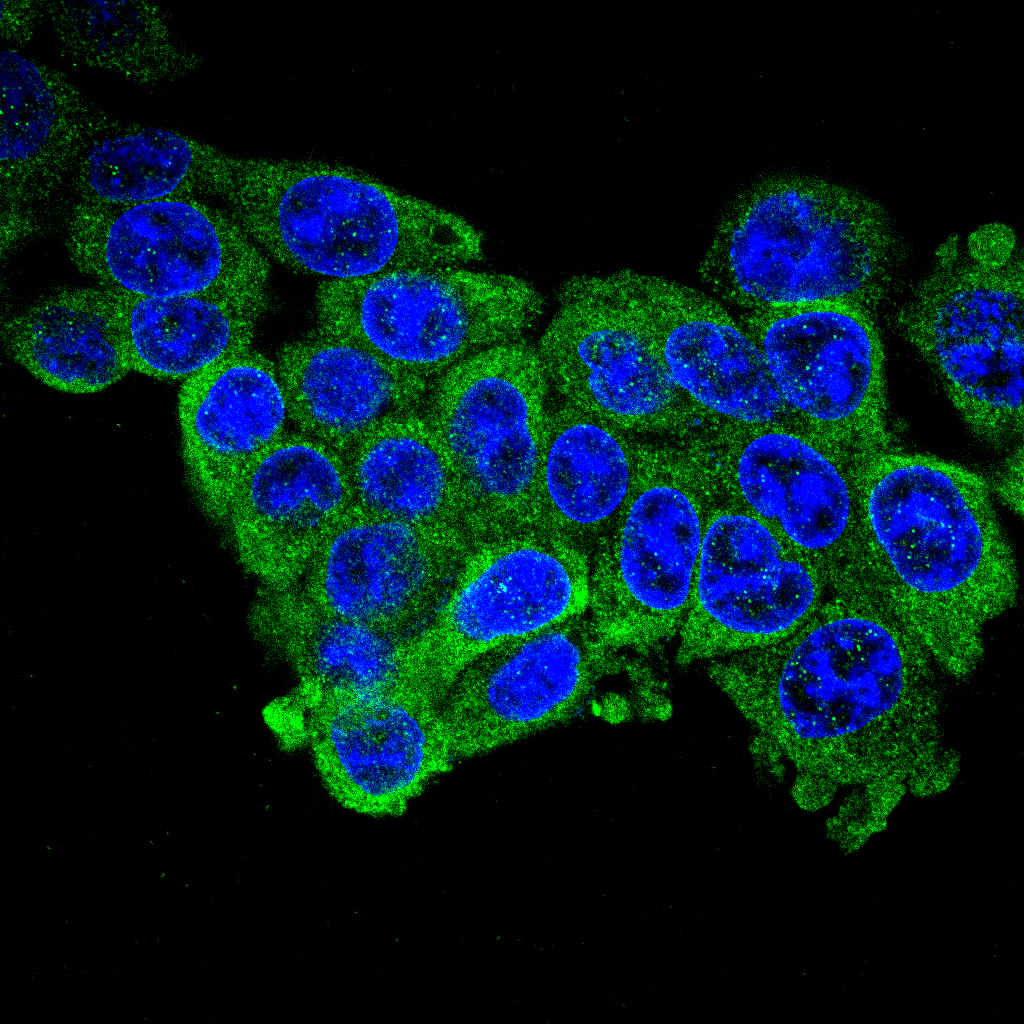

Supplement: Supplementary file 5 [file SupplementaryFile5.zip › 免疫荧光/6.13/SREBP1/lxj-NC-SR1_0012.tif.frames/lxj-NC-SR1_0012_T001.tif]

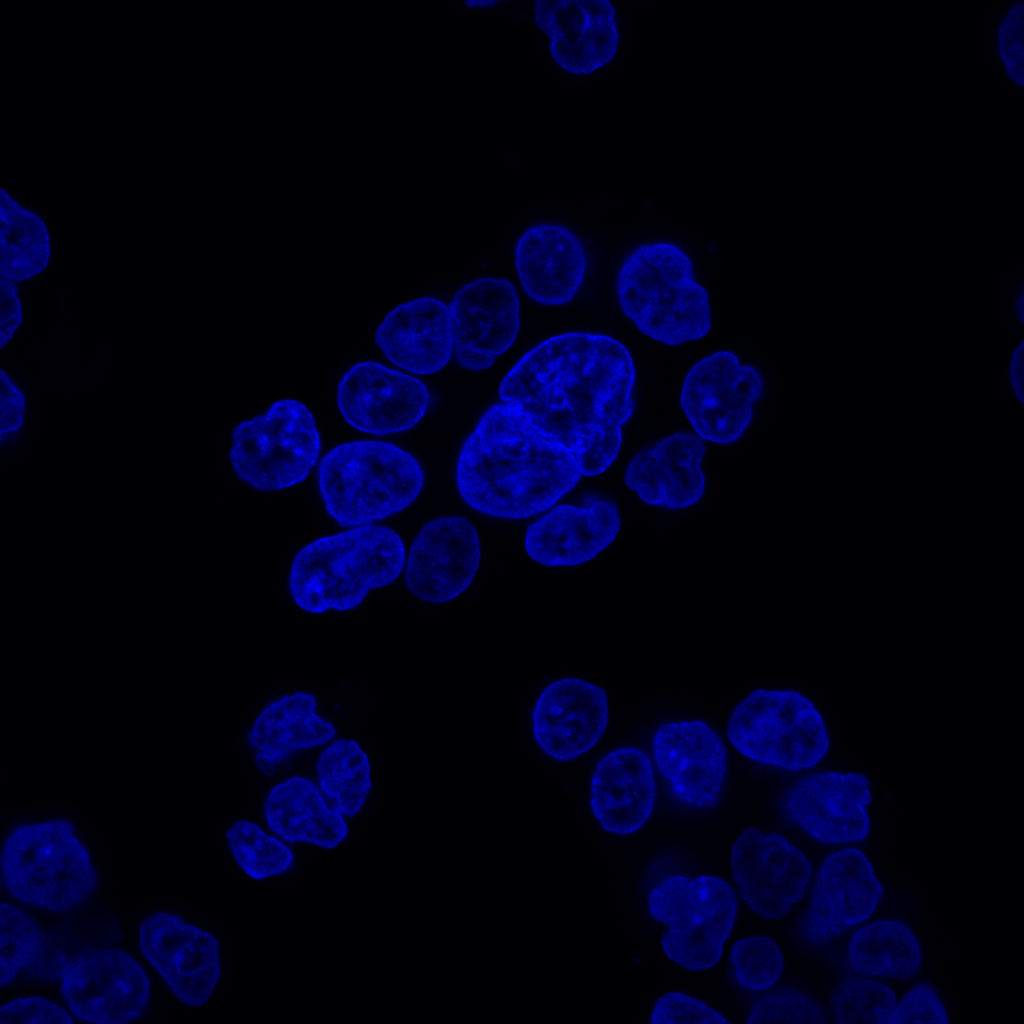

Supplement: Supplementary file 5 [file SupplementaryFile5.zip › 免疫荧光/6.18/BR1-C.tif.frames/BR1-C_C001T001.tif]

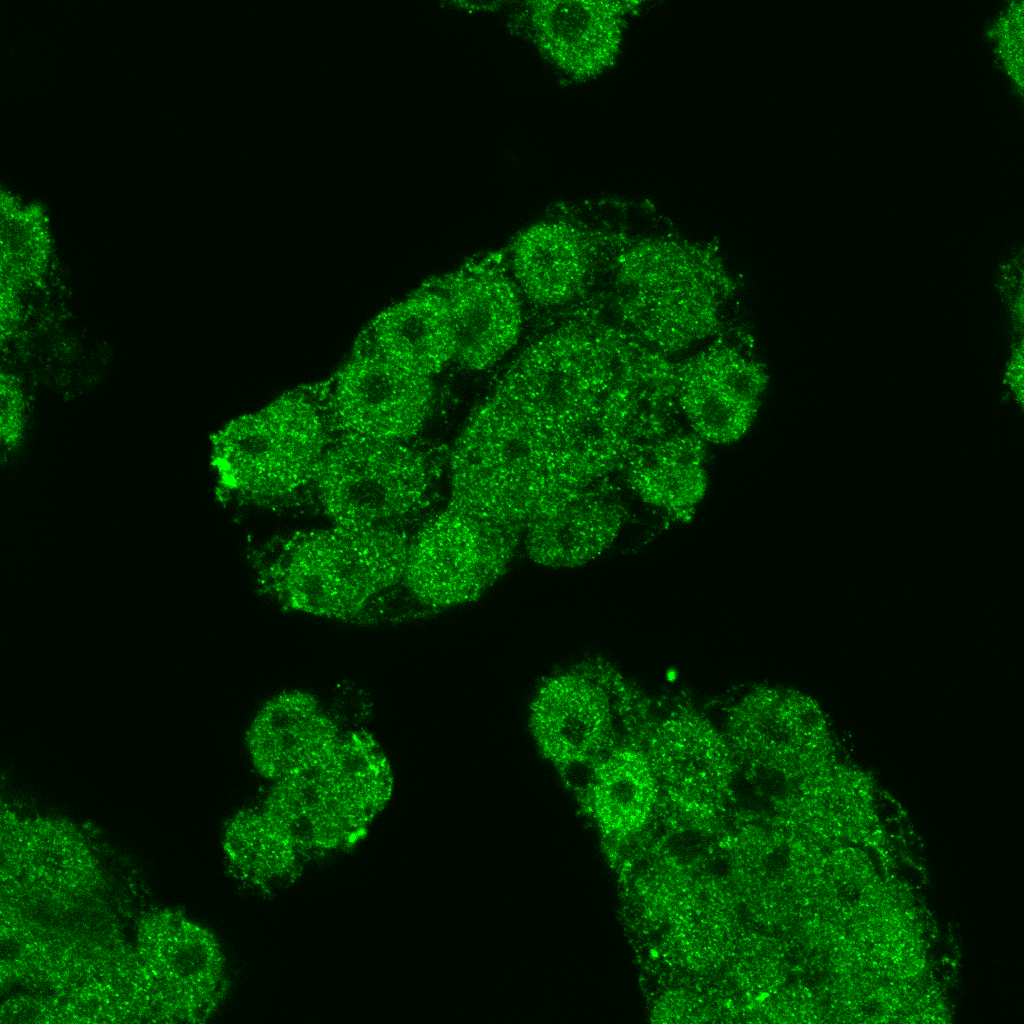

Supplement: Supplementary file 5 [file SupplementaryFile5.zip › 免疫荧光/6.18/BR1-C.tif.frames/BR1-C_C002T001.tif]

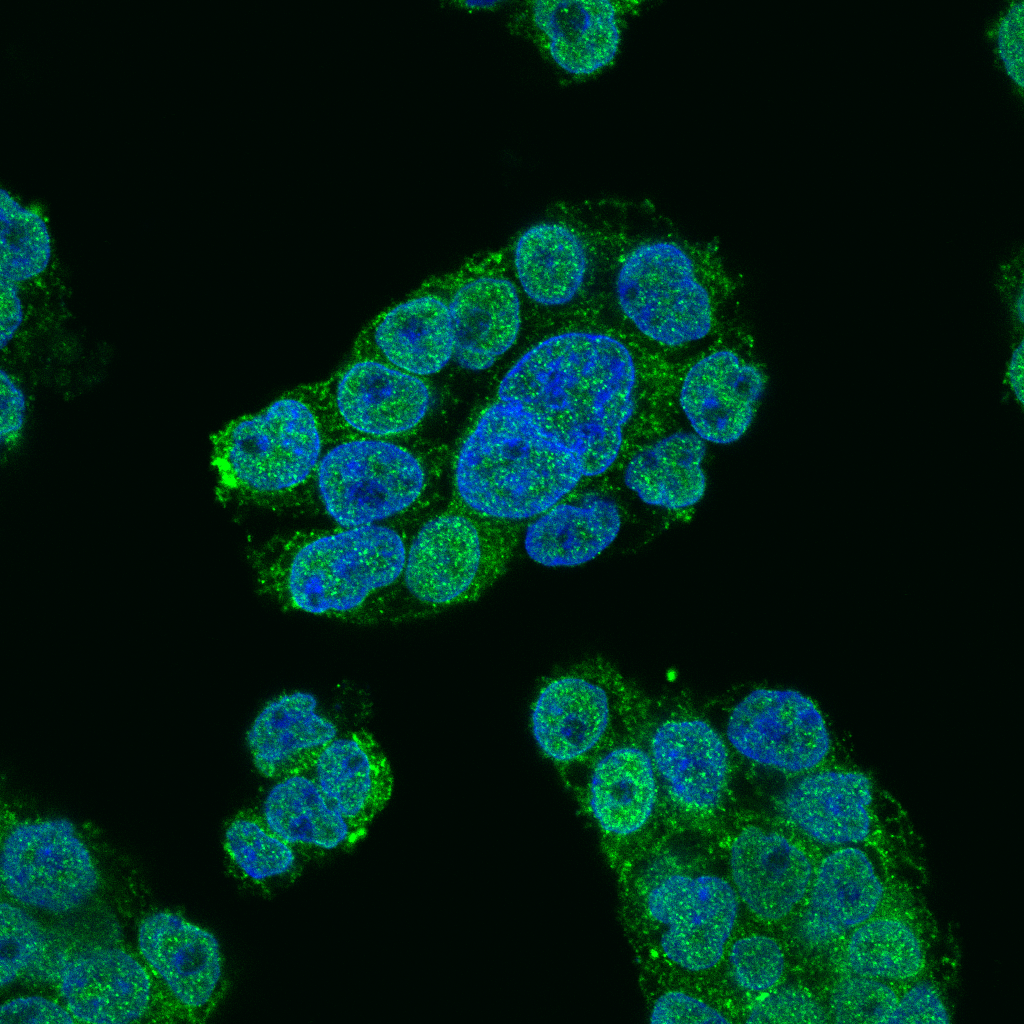

Supplement: Supplementary file 5 [file SupplementaryFile5.zip › 免疫荧光/6.18/BR1-C.tif.frames/BR1-C_T001.tif]

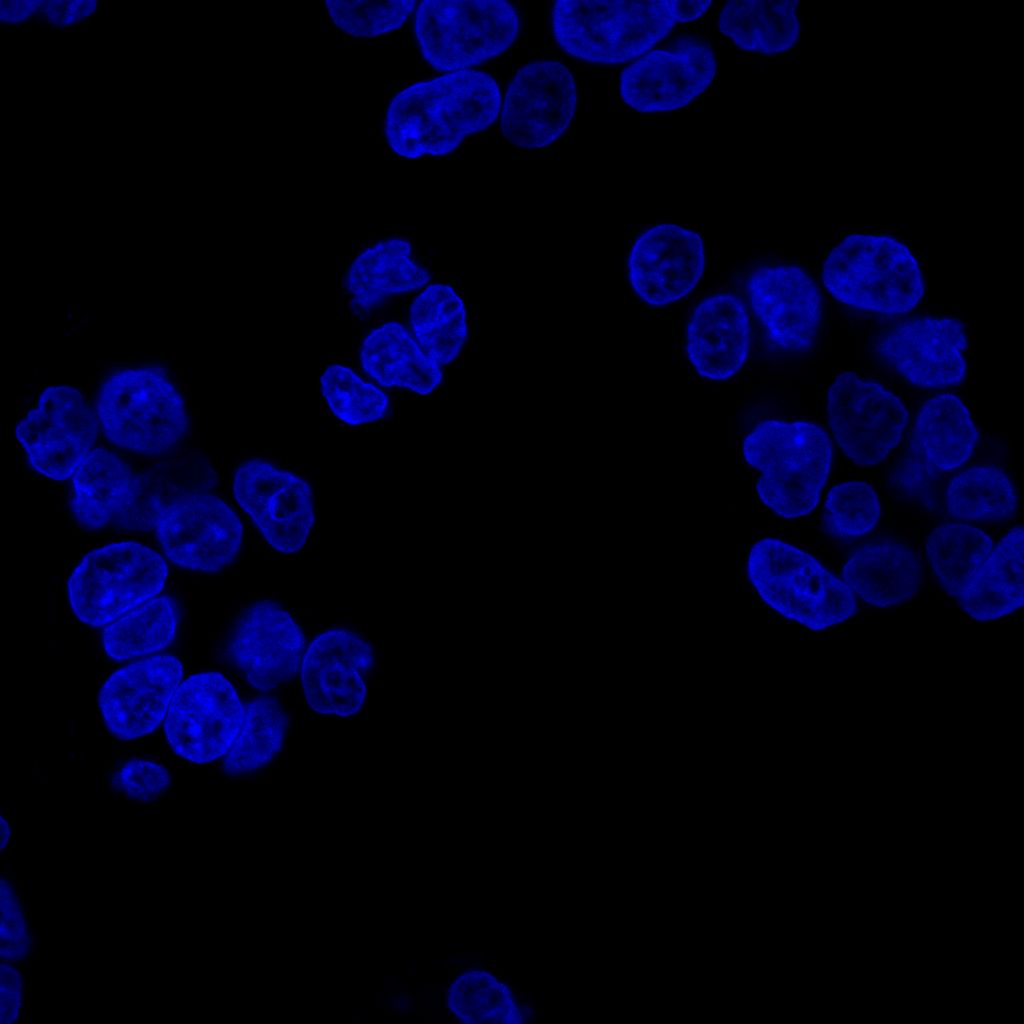

Supplement: Supplementary file 5 [file SupplementaryFile5.zip › 免疫荧光/6.18/BR1-C_0001.tif.frames/BR1-C_0001_C001T001.tif]

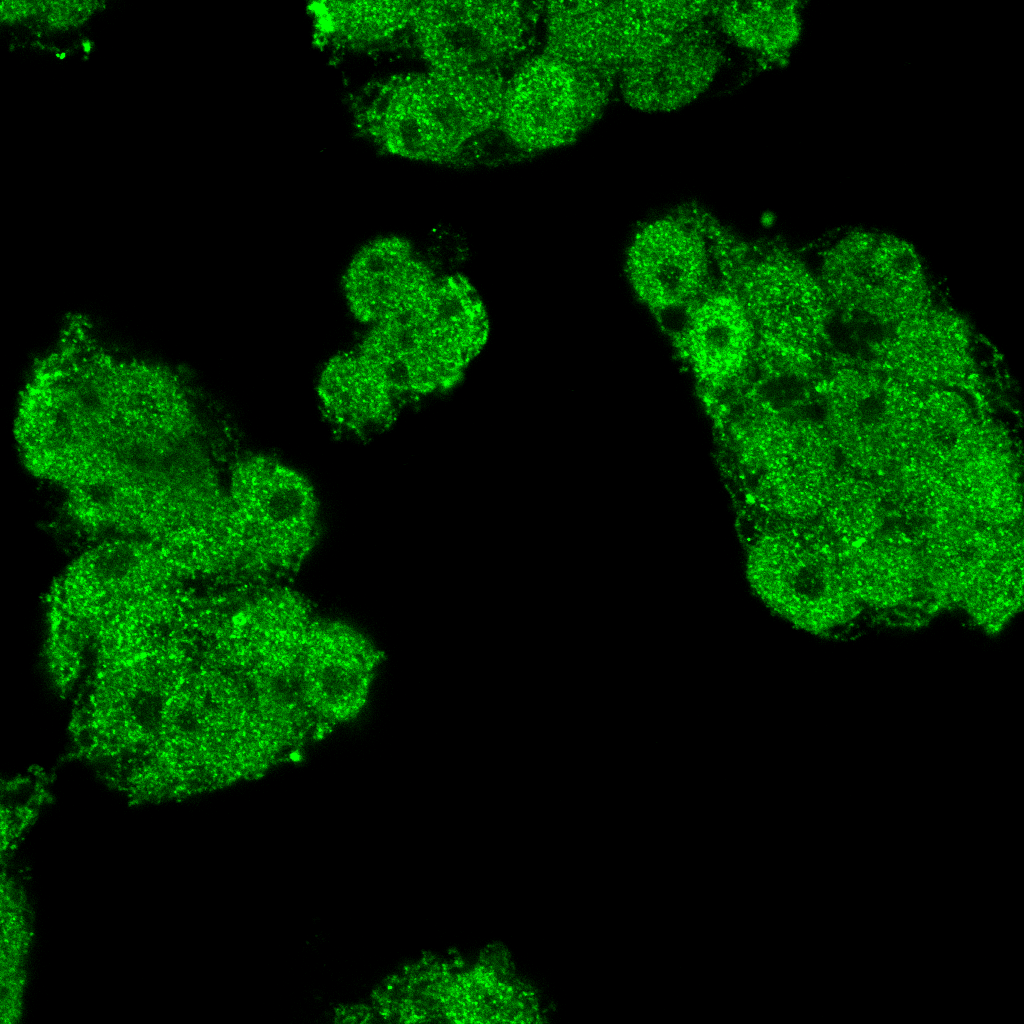

Supplement: Supplementary file 5 [file SupplementaryFile5.zip › 免疫荧光/6.18/BR1-C_0001.tif.frames/BR1-C_0001_C002T001.tif]

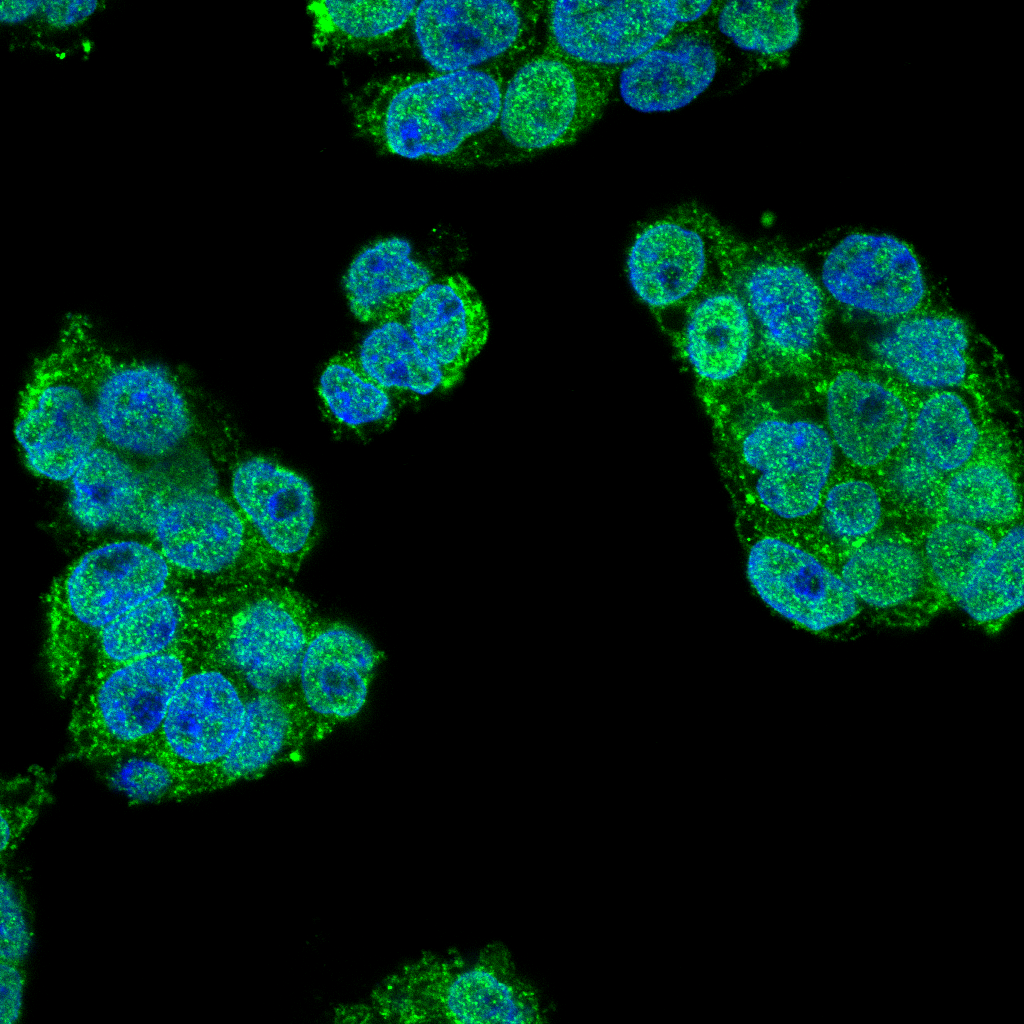

Supplement: Supplementary file 5 [file SupplementaryFile5.zip › 免疫荧光/6.18/BR1-C_0001.tif.frames/BR1-C_0001_T001.tif]

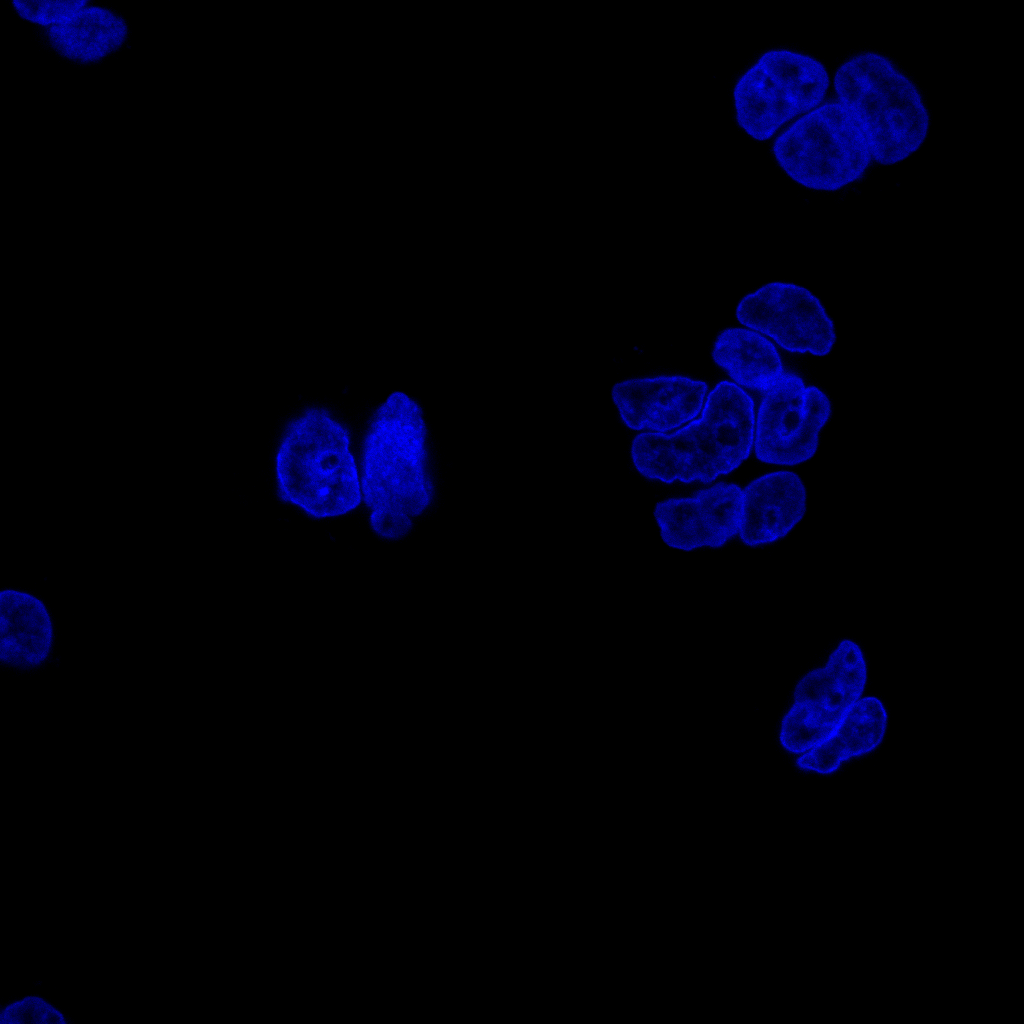

Supplement: Supplementary file 5 [file SupplementaryFile5.zip › 免疫荧光/6.18/BR1-C_0002.tif.frames/BR1-C_0002_C001T001.tif]

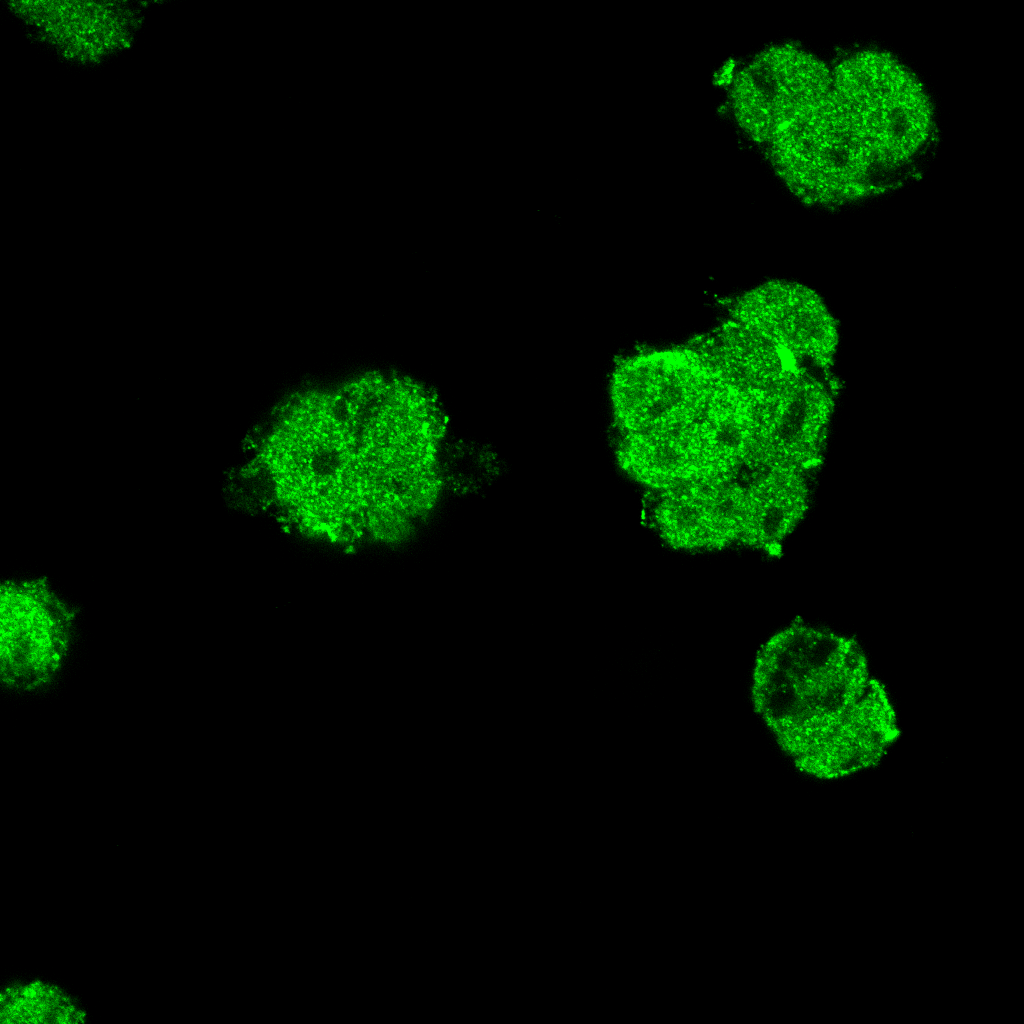

Supplement: Supplementary file 5 [file SupplementaryFile5.zip › 免疫荧光/6.18/BR1-C_0002.tif.frames/BR1-C_0002_C002T001.tif]

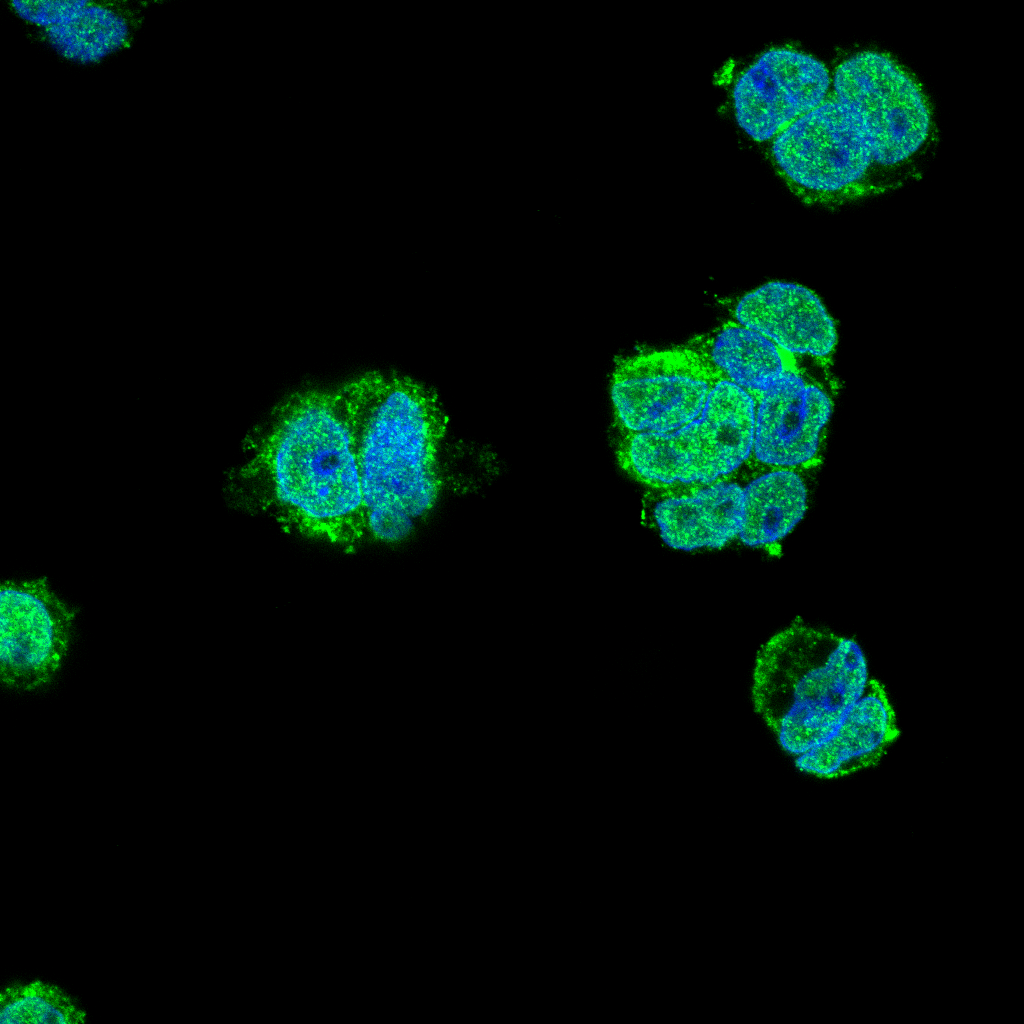

Supplement: Supplementary file 5 [file SupplementaryFile5.zip › 免疫荧光/6.18/BR1-C_0002.tif.frames/BR1-C_0002_T001.tif]

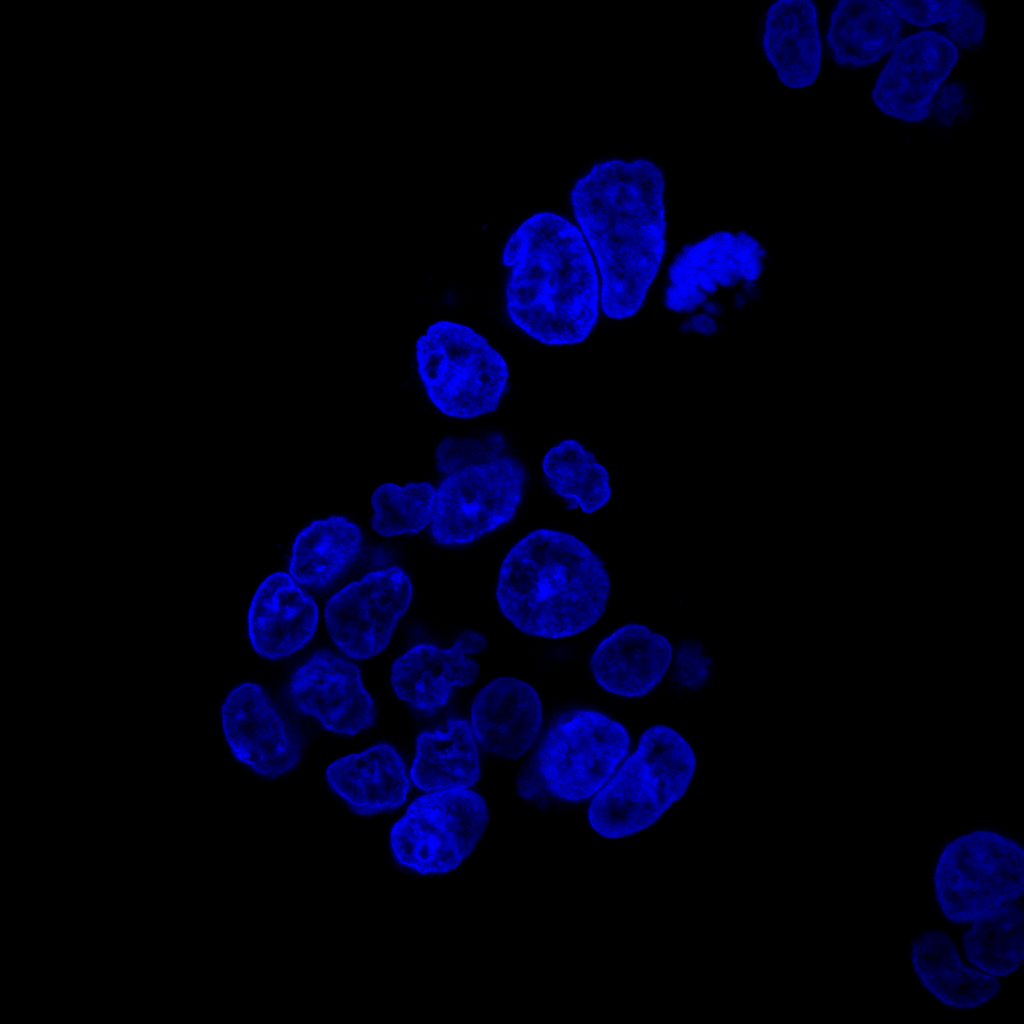

Supplement: Supplementary file 5 [file SupplementaryFile5.zip › 免疫荧光/6.18/BR1-C_0003.tif.frames/BR1-C_0003_C001T001.tif]

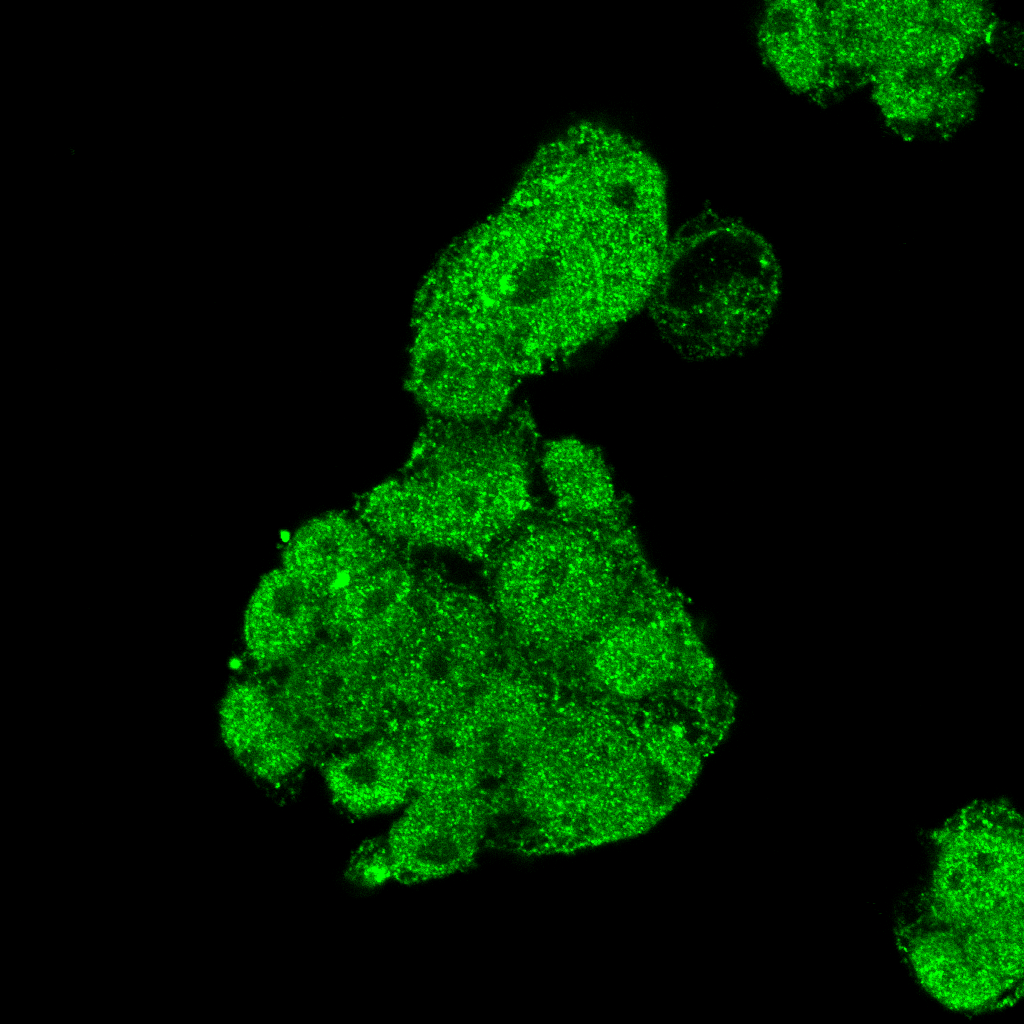

Supplement: Supplementary file 5 [file SupplementaryFile5.zip › 免疫荧光/6.18/BR1-C_0003.tif.frames/BR1-C_0003_C002T001.tif]

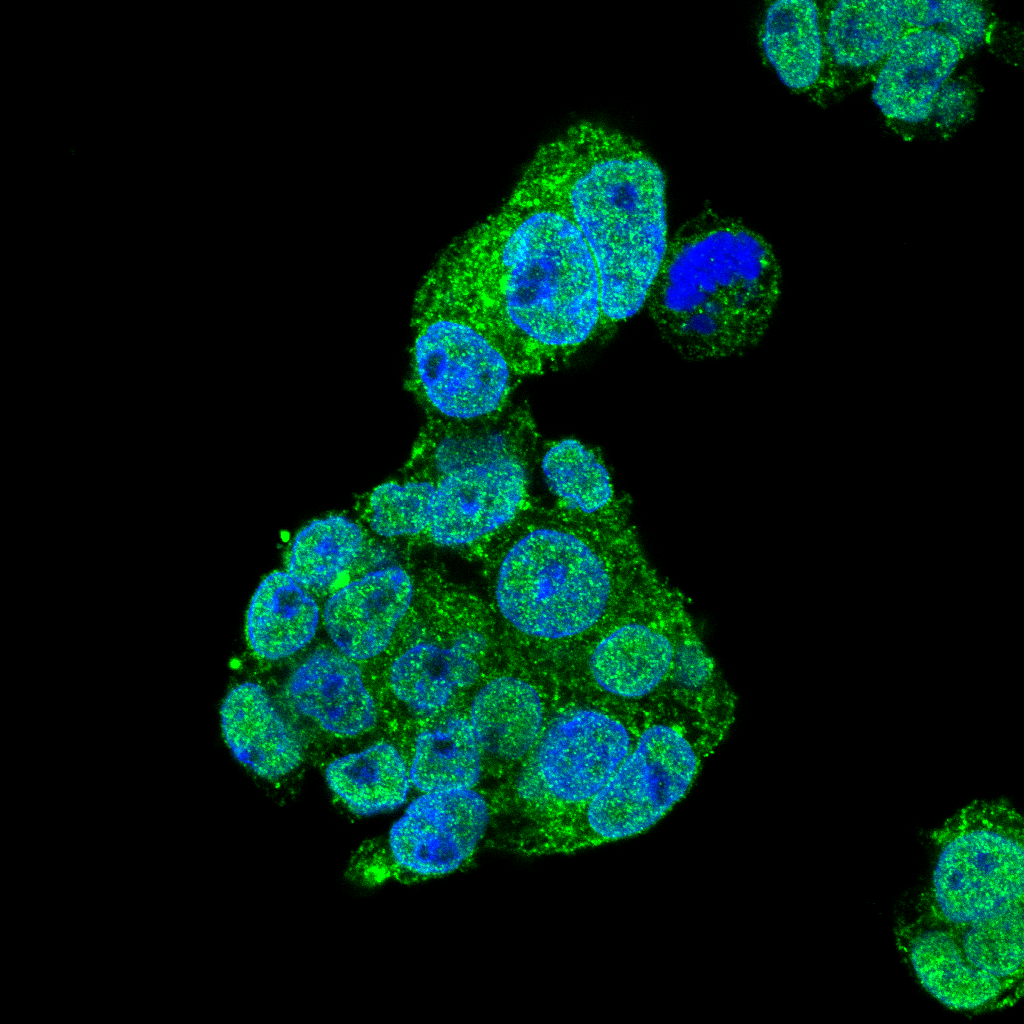

Supplement: Supplementary file 5 [file SupplementaryFile5.zip › 免疫荧光/6.18/BR1-C_0003.tif.frames/BR1-C_0003_T001.tif]

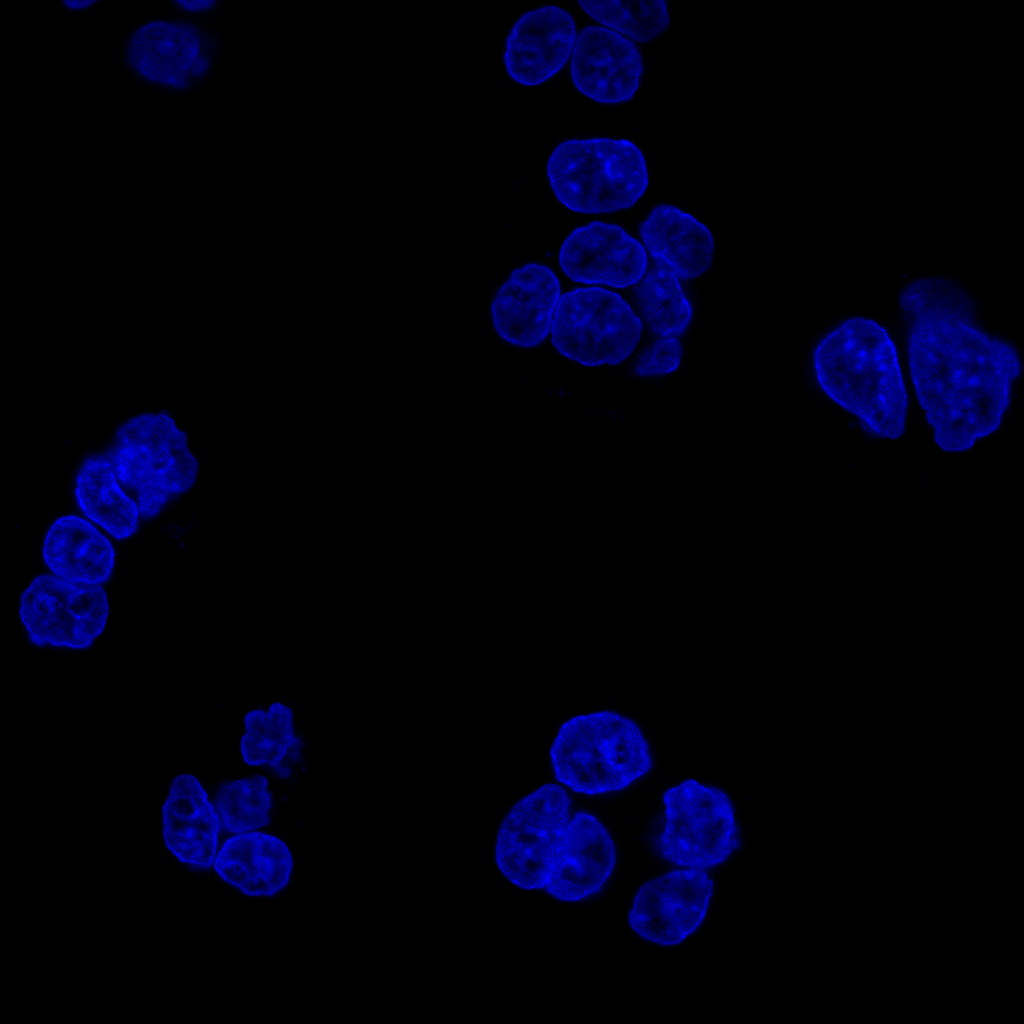

Supplement: Supplementary file 5 [file SupplementaryFile5.zip › 免疫荧光/6.18/BR1-C_0004.tif.frames/BR1-C_0004_C001T001.tif]

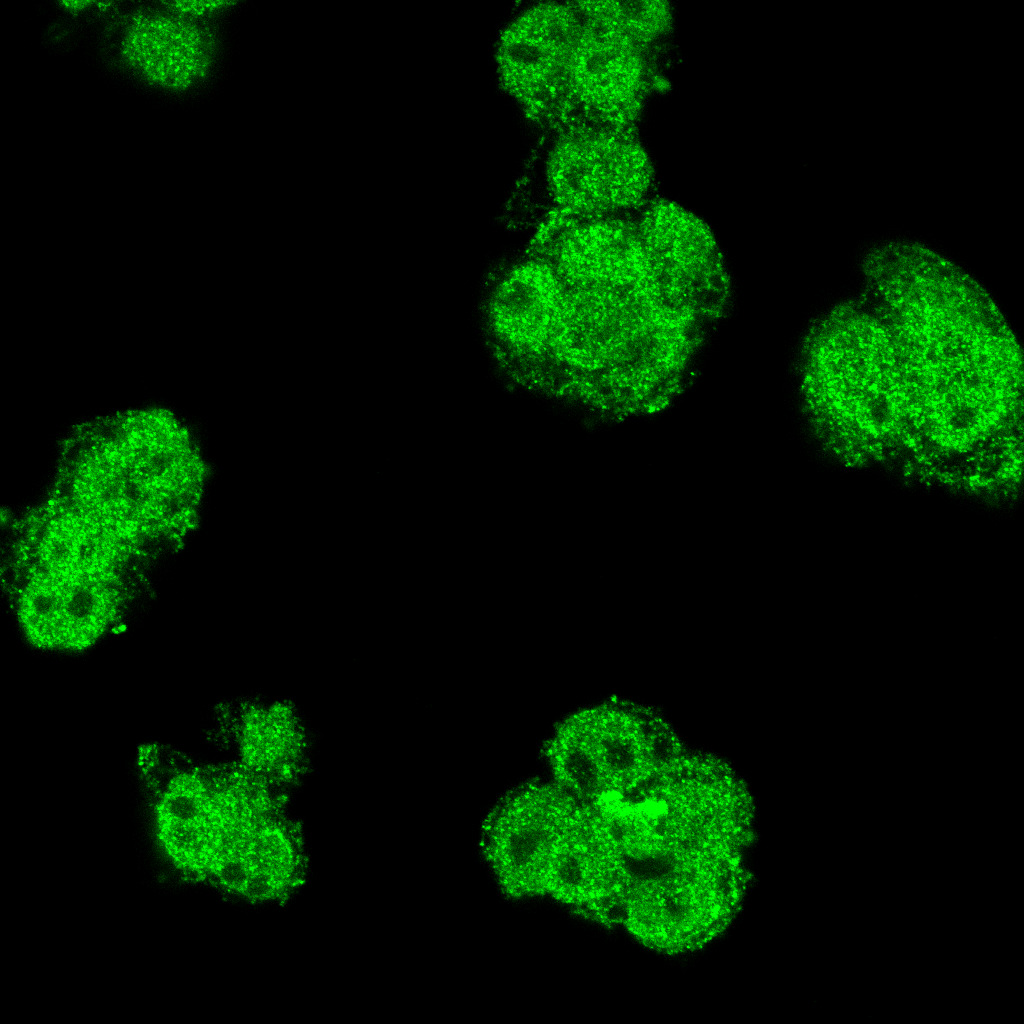

Supplement: Supplementary file 5 [file SupplementaryFile5.zip › 免疫荧光/6.18/BR1-C_0004.tif.frames/BR1-C_0004_C002T001.tif]

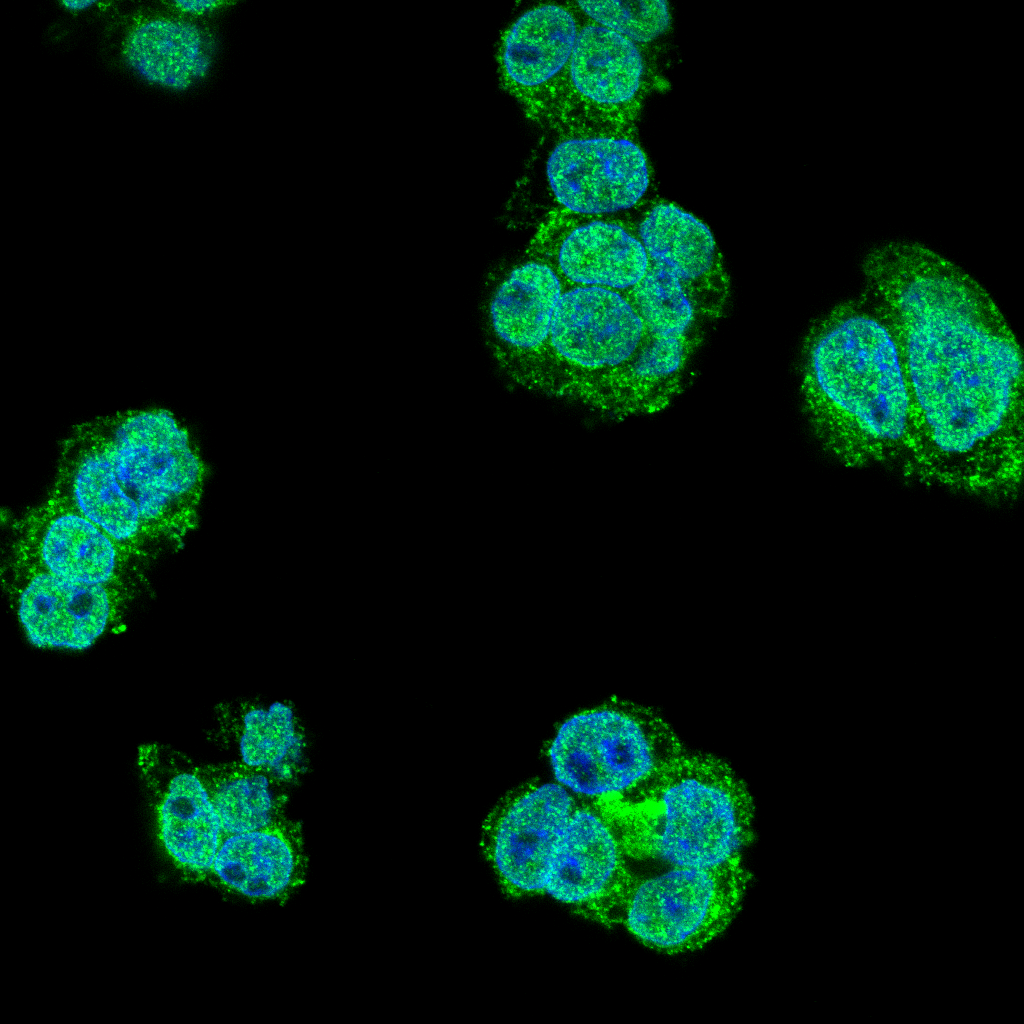

Supplement: Supplementary file 5 [file SupplementaryFile5.zip › 免疫荧光/6.18/BR1-C_0004.tif.frames/BR1-C_0004_T001.tif]

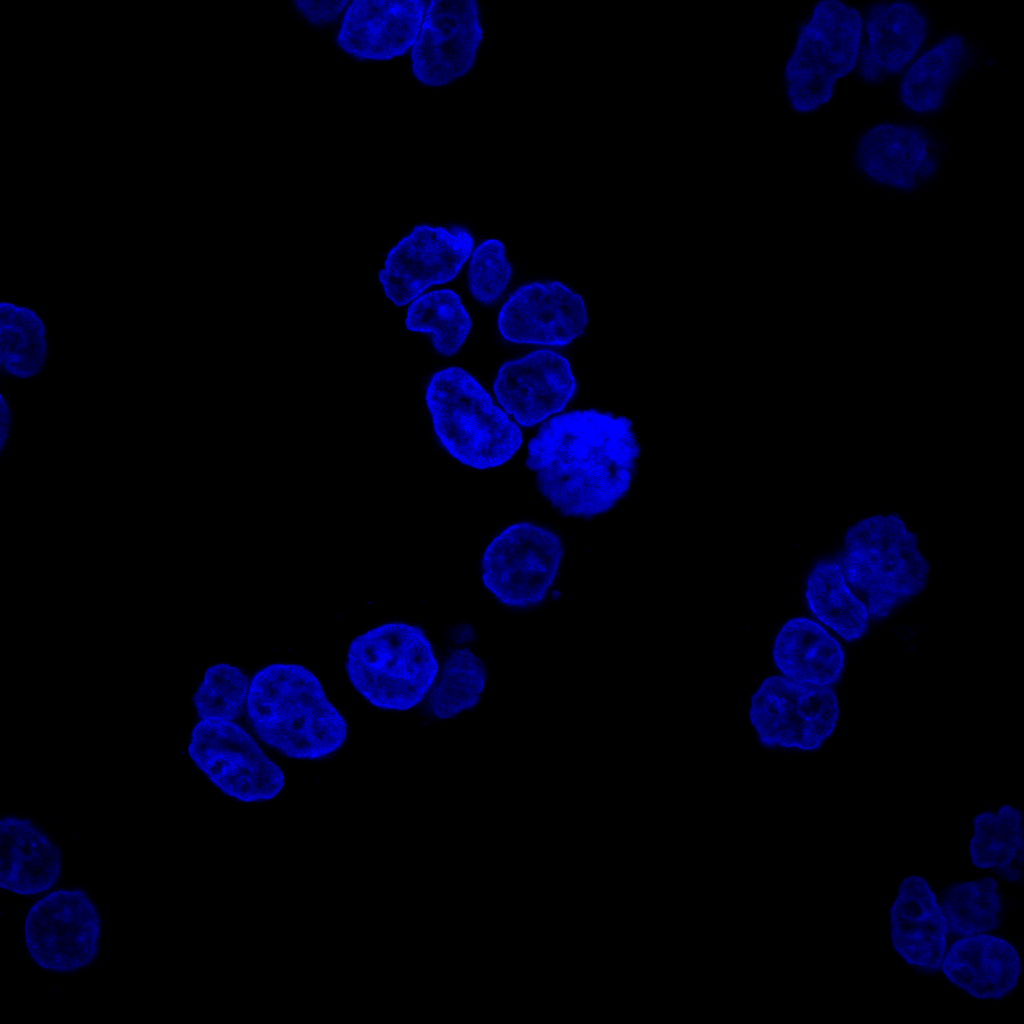

Supplement: Supplementary file 5 [file SupplementaryFile5.zip › 免疫荧光/6.18/BR1-C_0005.tif.frames/BR1-C_0005_C001T001.tif]

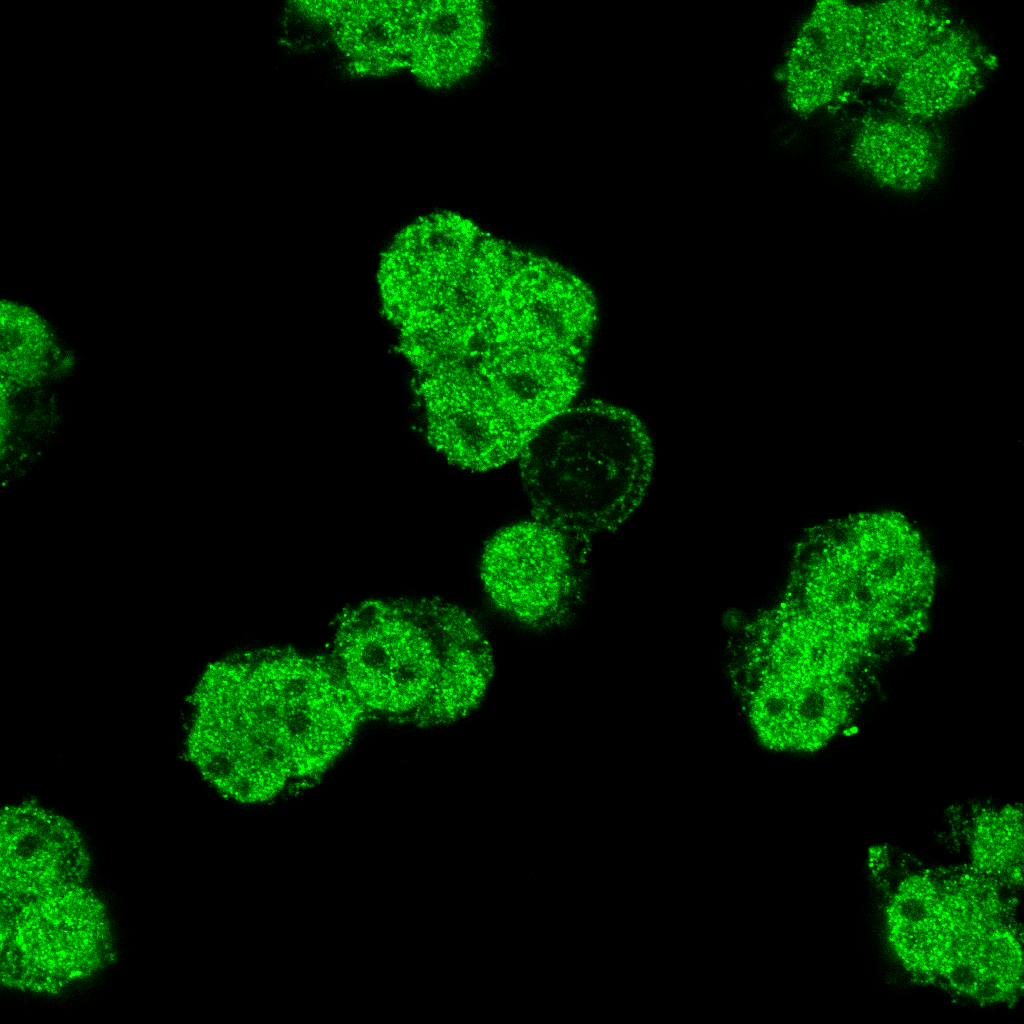

Supplement: Supplementary file 5 [file SupplementaryFile5.zip › 免疫荧光/6.18/BR1-C_0005.tif.frames/BR1-C_0005_C002T001.tif]

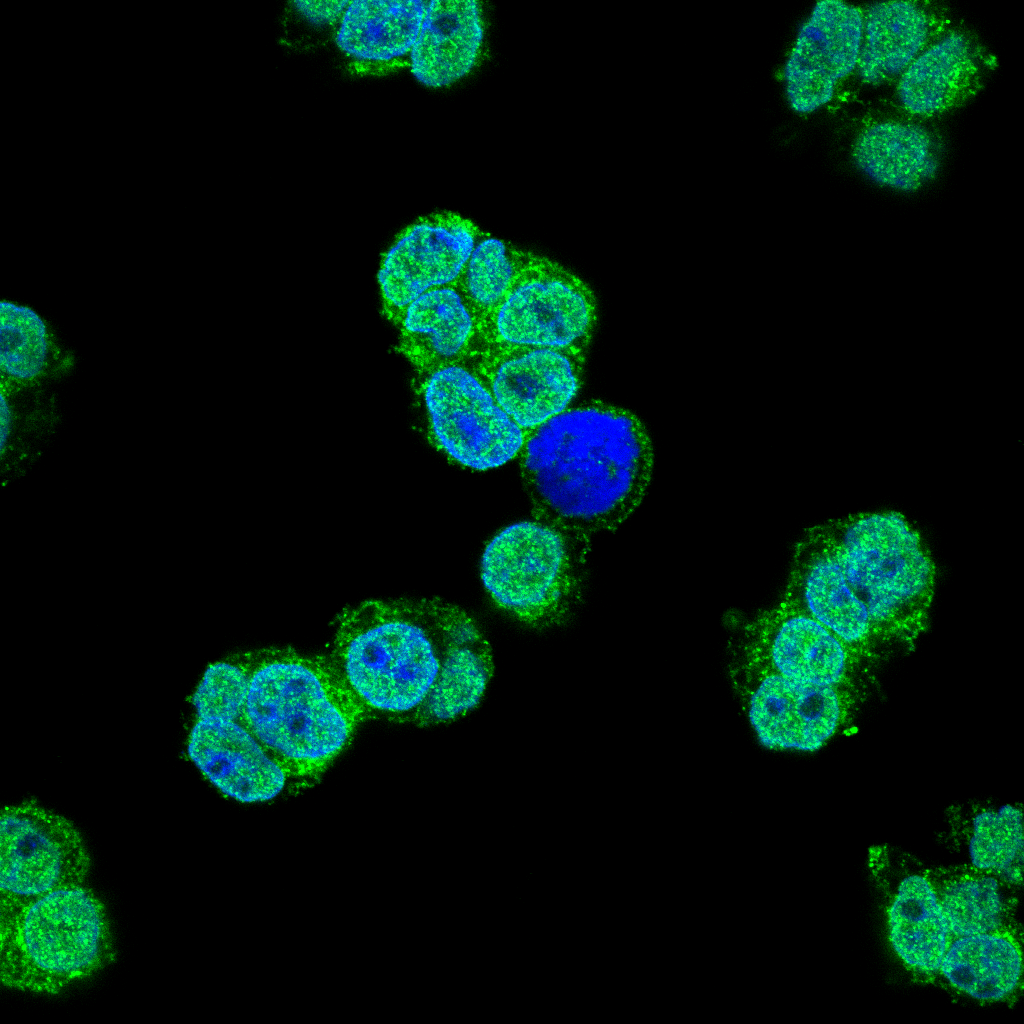

Supplement: Supplementary file 5 [file SupplementaryFile5.zip › 免疫荧光/6.18/BR1-C_0005.tif.frames/BR1-C_0005_T001.tif]

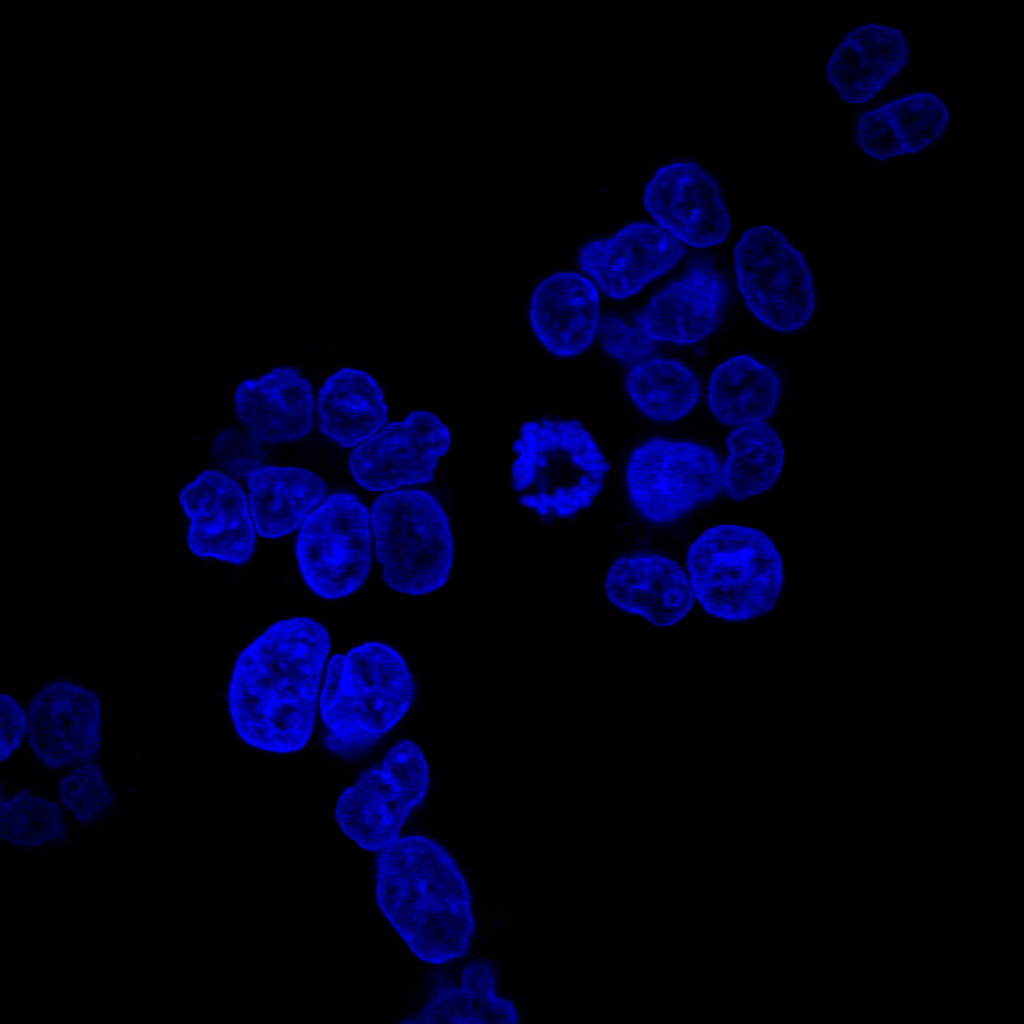

Supplement: Supplementary file 5 [file SupplementaryFile5.zip › 免疫荧光/6.18/BR1-C_0006.tif.frames/BR1-C_0006_C001T001.tif]

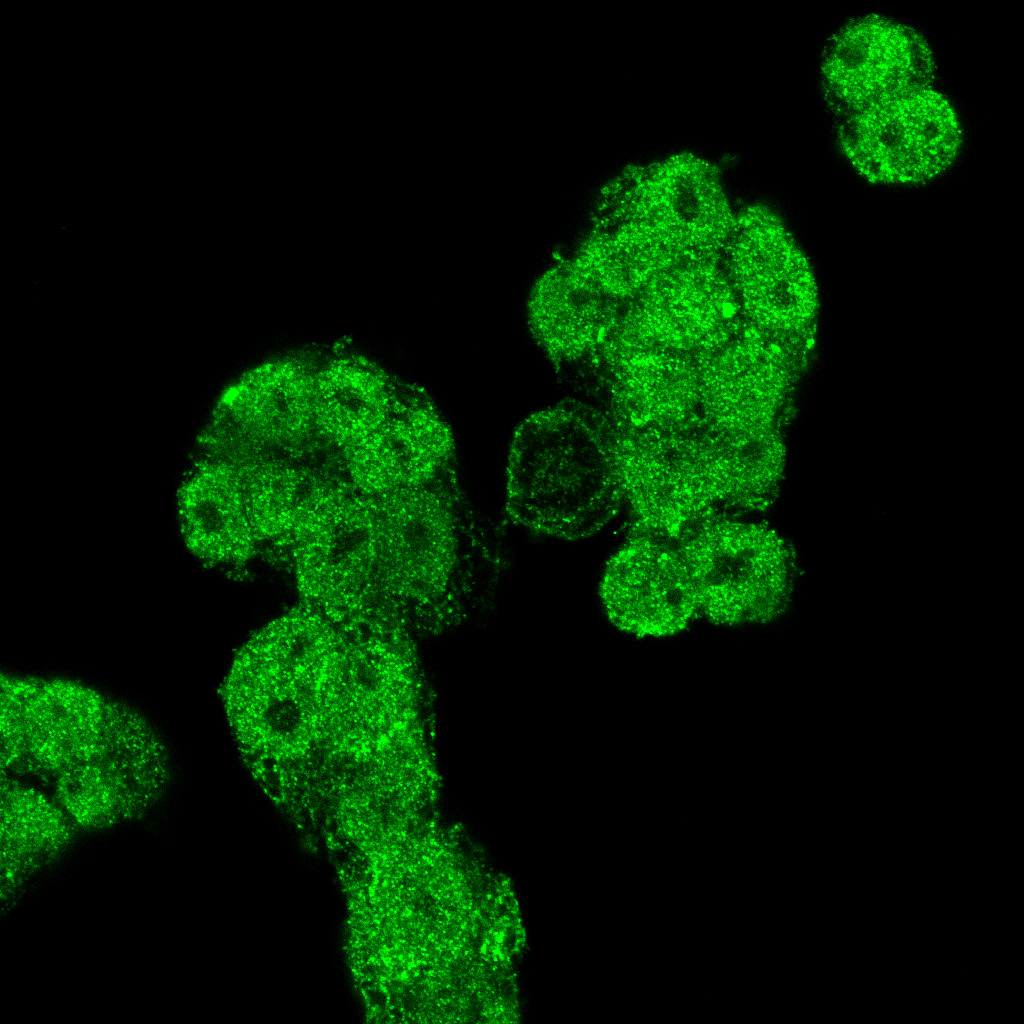

Supplement: Supplementary file 5 [file SupplementaryFile5.zip › 免疫荧光/6.18/BR1-C_0006.tif.frames/BR1-C_0006_C002T001.tif]

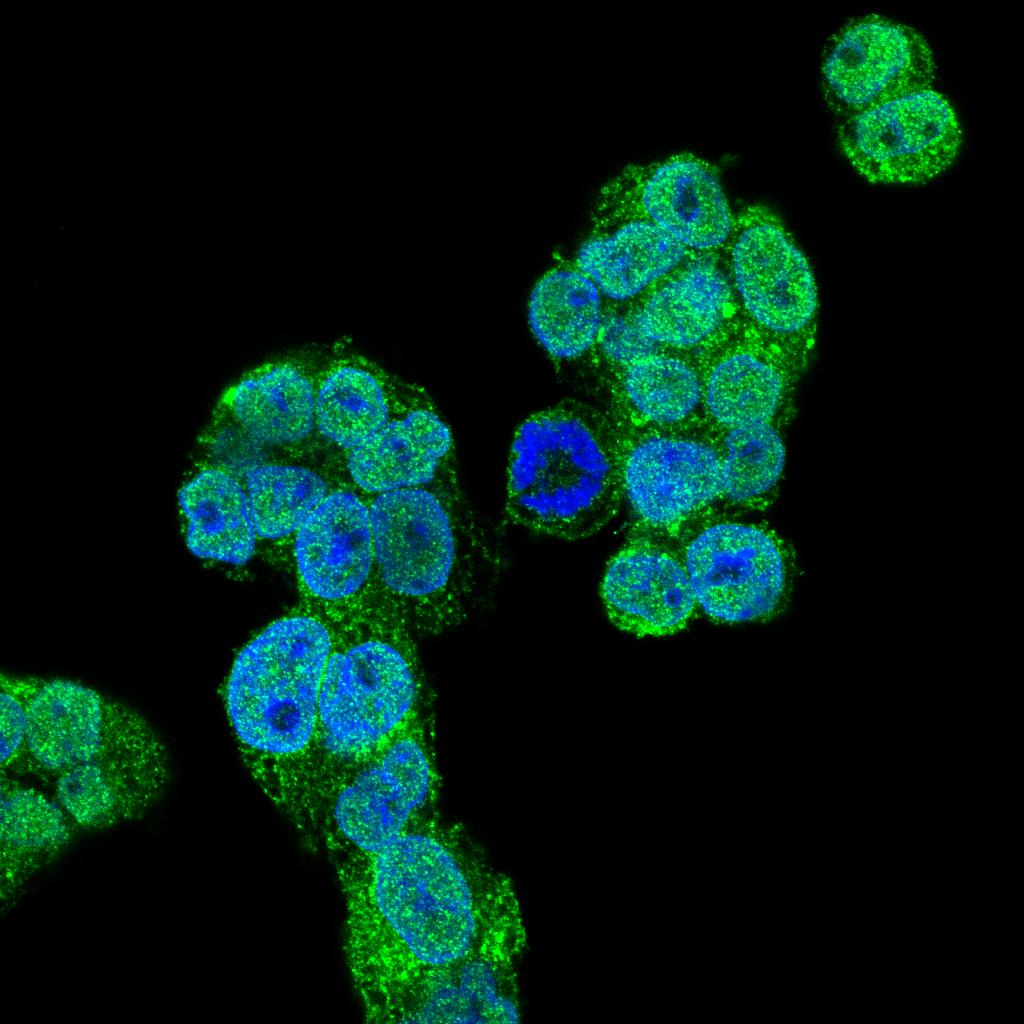

Supplement: Supplementary file 5 [file SupplementaryFile5.zip › 免疫荧光/6.18/BR1-C_0006.tif.frames/BR1-C_0006_T001.tif]

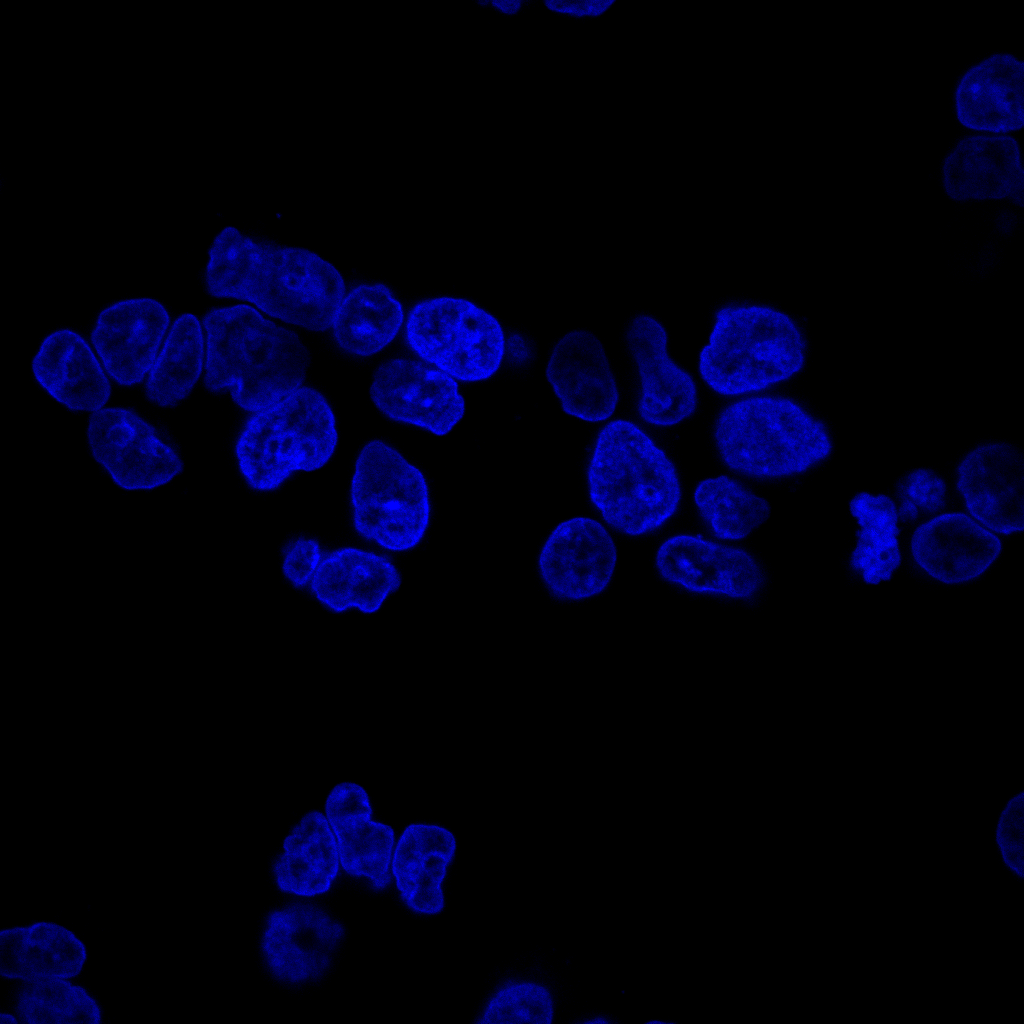

Supplement: Supplementary file 5 [file SupplementaryFile5.zip › 免疫荧光/6.18/BR1-C_0007.tif.frames/BR1-C_0007_C001T001.tif]

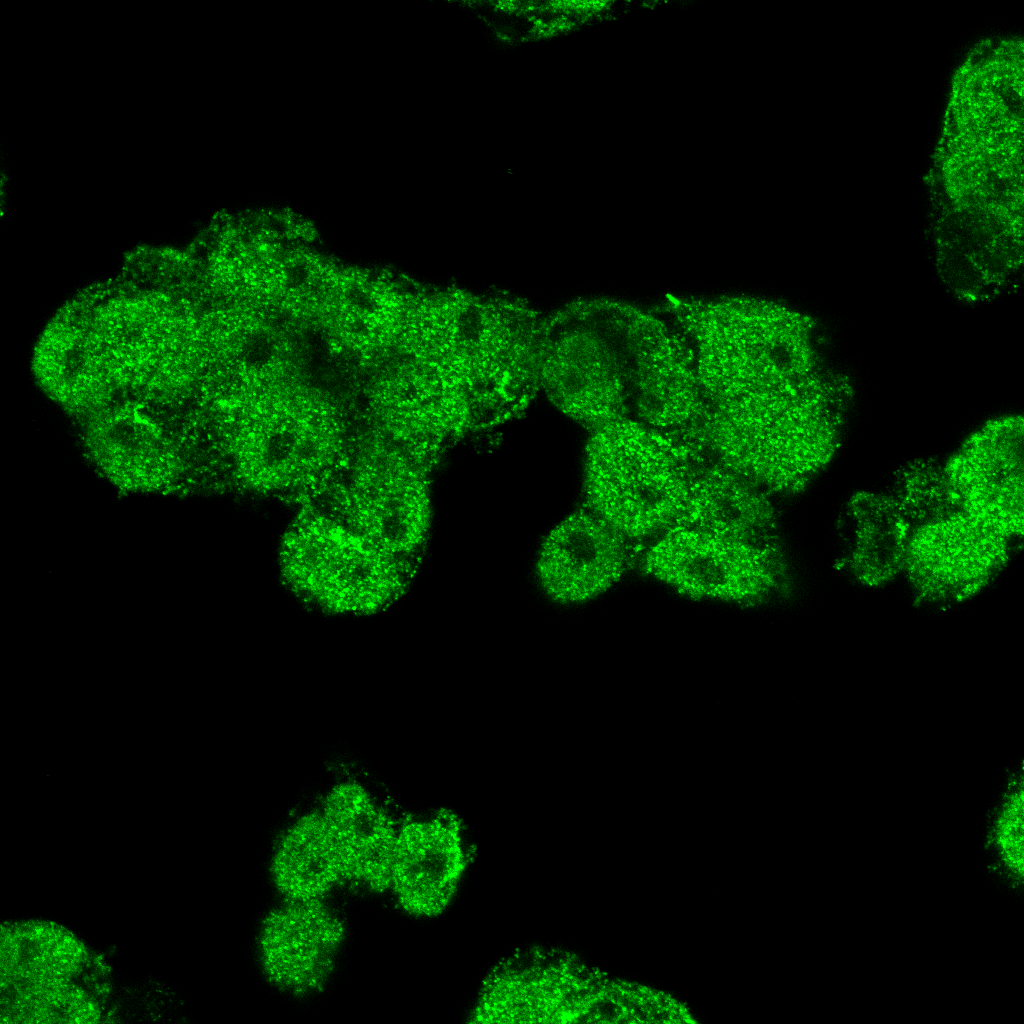

Supplement: Supplementary file 5 [file SupplementaryFile5.zip › 免疫荧光/6.18/BR1-C_0007.tif.frames/BR1-C_0007_C002T001.tif]

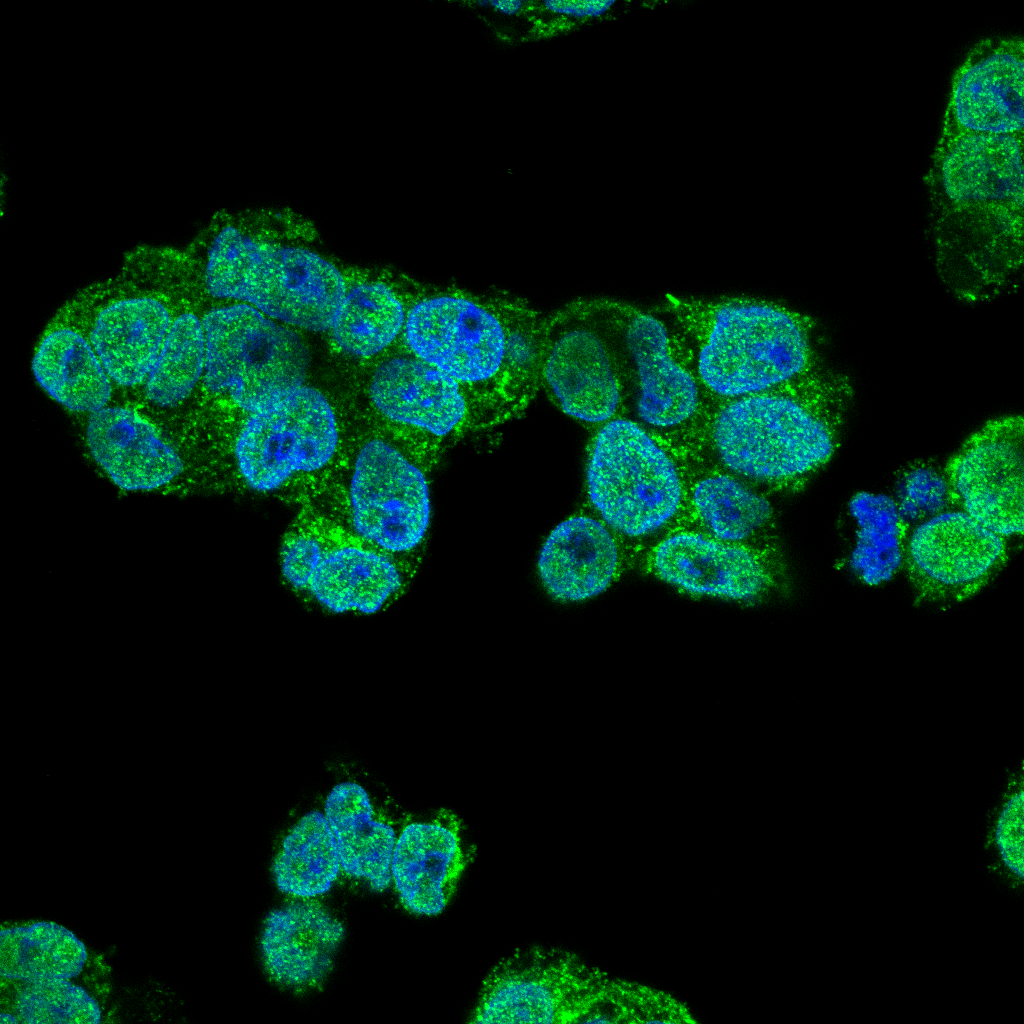

Supplement: Supplementary file 5 [file SupplementaryFile5.zip › 免疫荧光/6.18/BR1-C_0007.tif.frames/BR1-C_0007_T001.tif]

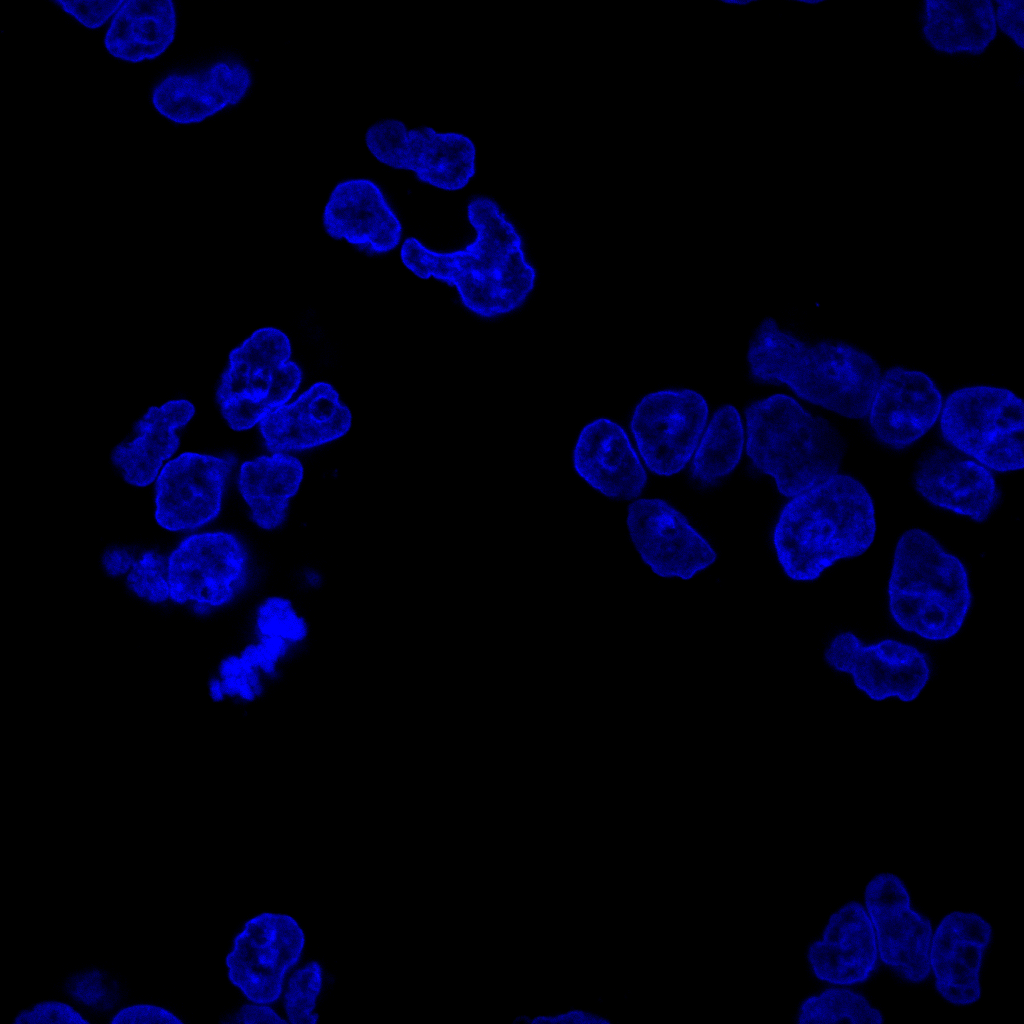

Supplement: Supplementary file 5 [file SupplementaryFile5.zip › 免疫荧光/6.18/BR1-C_0008.tif.frames/BR1-C_0008_C001T001.tif]

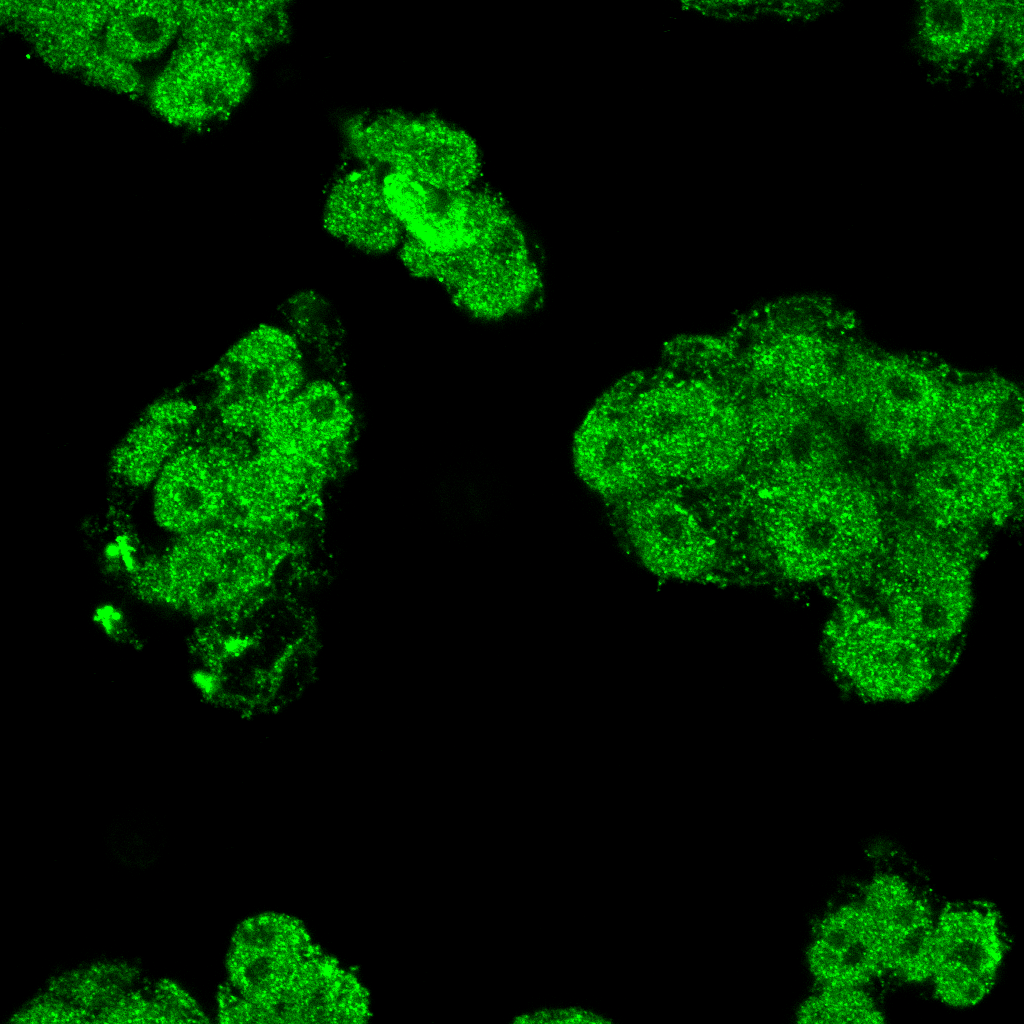

Supplement: Supplementary file 5 [file SupplementaryFile5.zip › 免疫荧光/6.18/BR1-C_0008.tif.frames/BR1-C_0008_C002T001.tif]

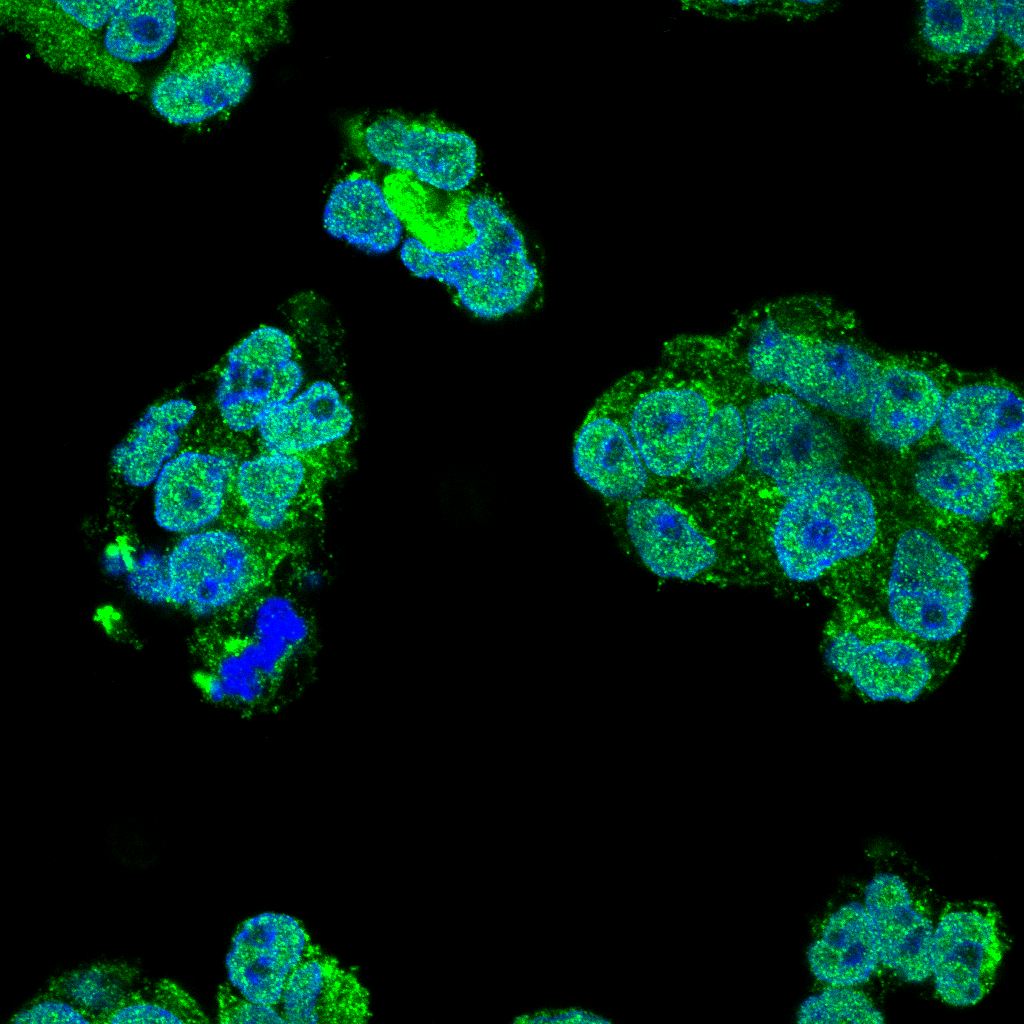

Supplement: Supplementary file 5 [file SupplementaryFile5.zip › 免疫荧光/6.18/BR1-C_0008.tif.frames/BR1-C_0008_T001.tif]

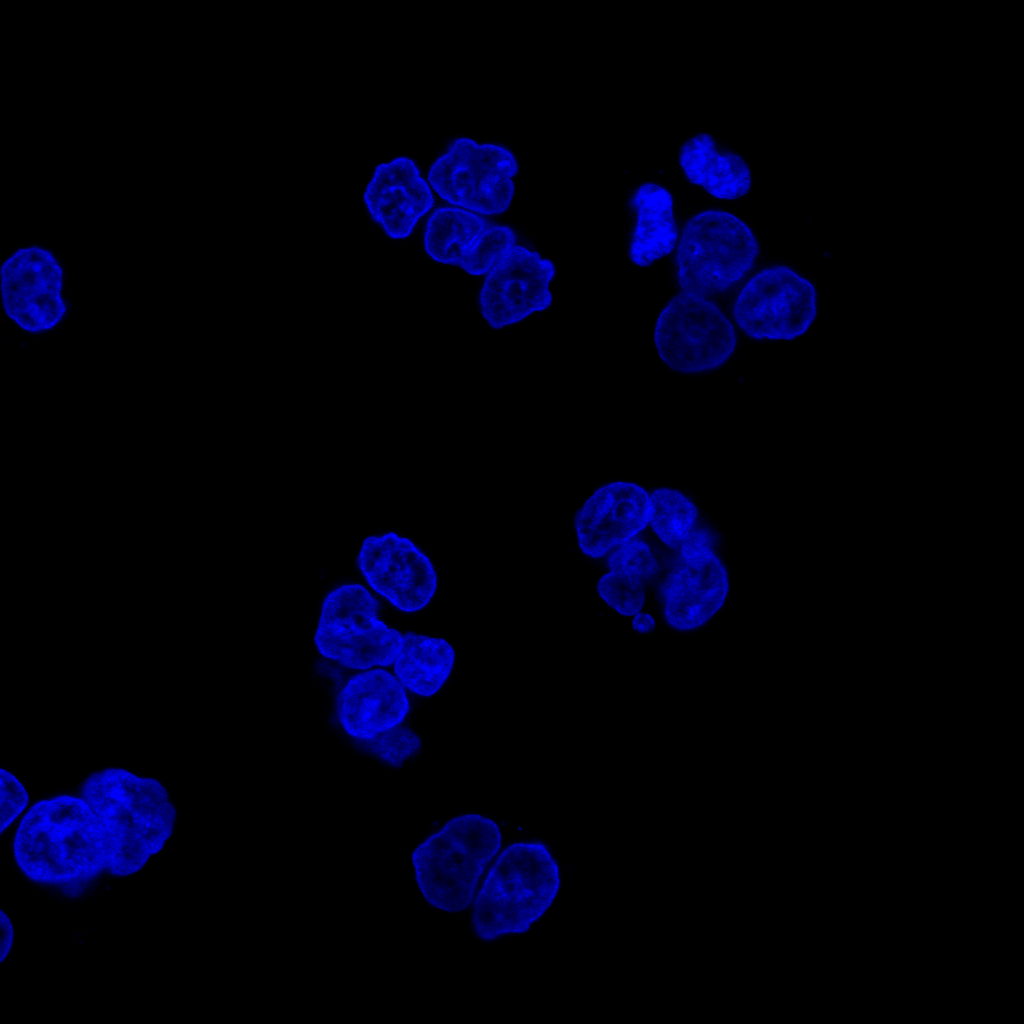

Supplement: Supplementary file 5 [file SupplementaryFile5.zip › 免疫荧光/6.18/BR1-C_0009.tif.frames/BR1-C_0009_C001T001.tif]

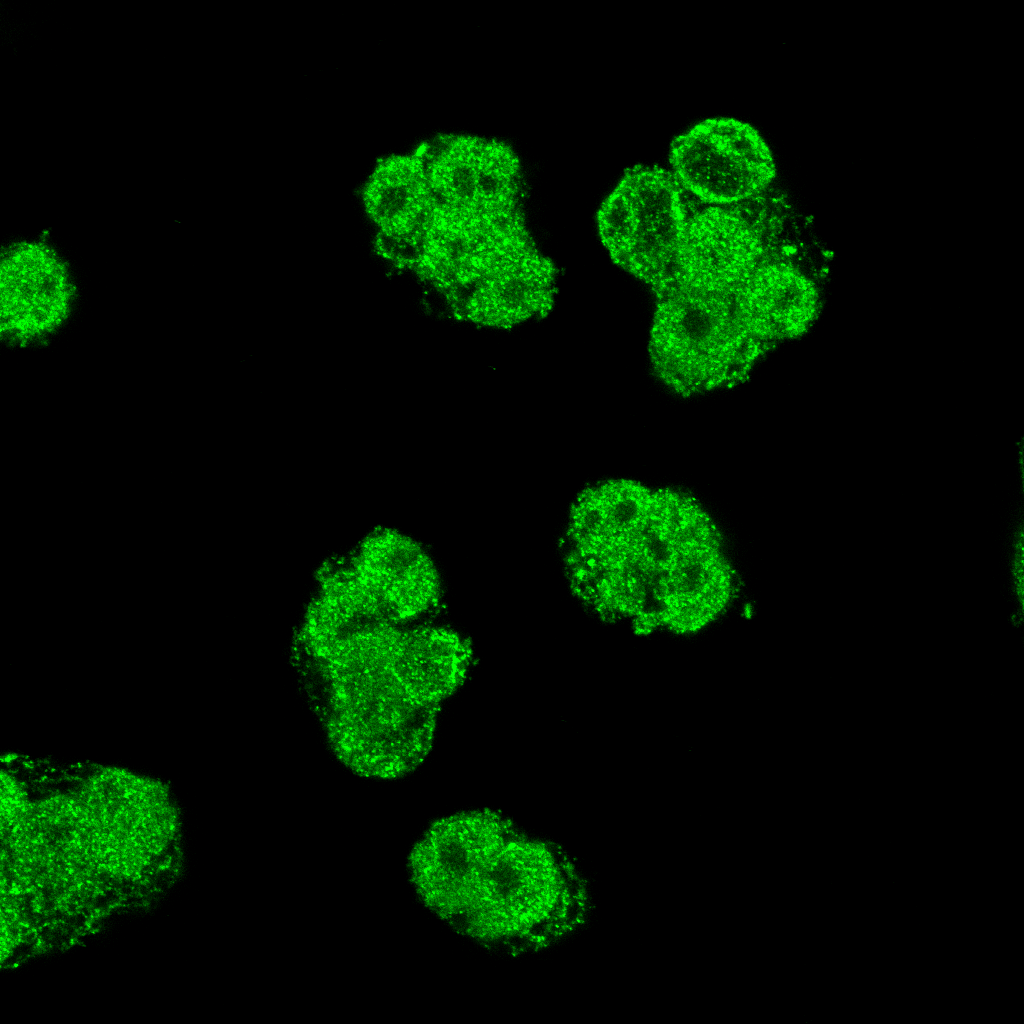

Supplement: Supplementary file 5 [file SupplementaryFile5.zip › 免疫荧光/6.18/BR1-C_0009.tif.frames/BR1-C_0009_C002T001.tif]

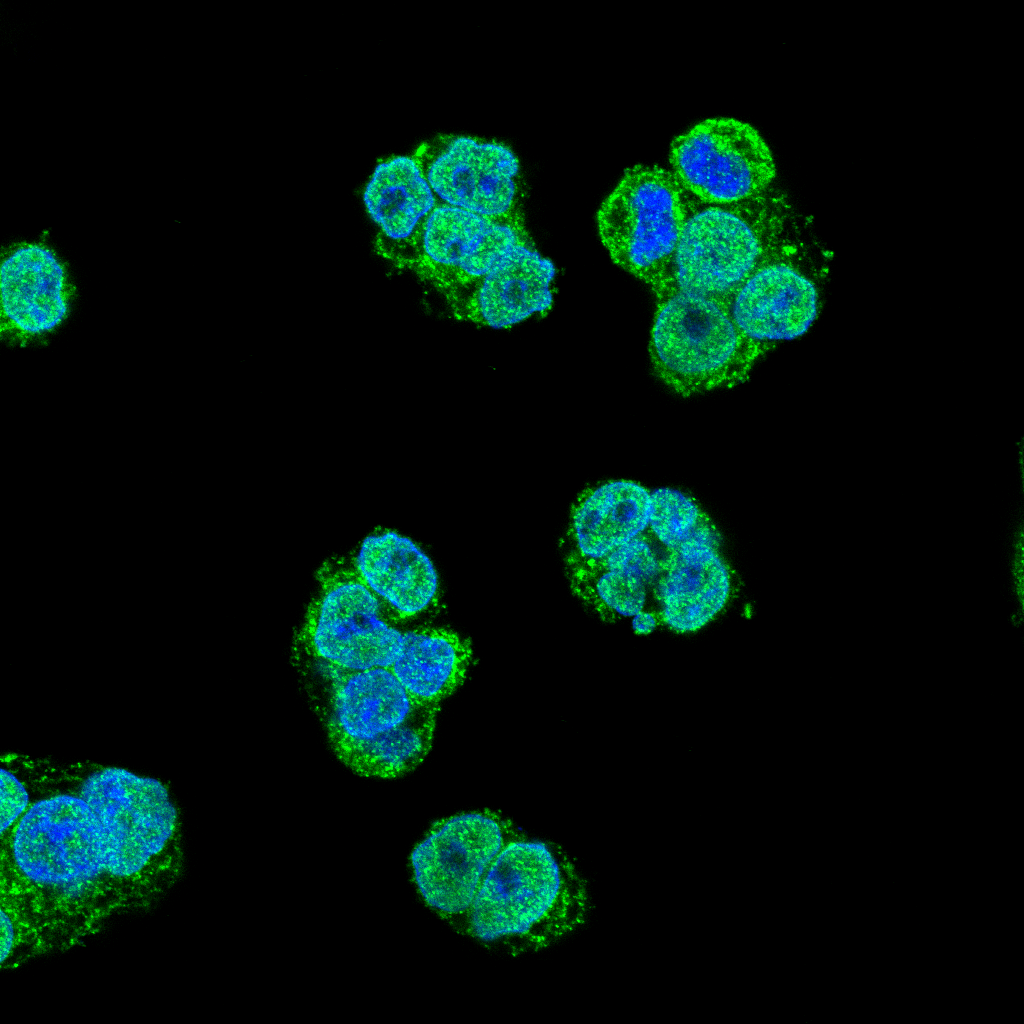

Supplement: Supplementary file 5 [file SupplementaryFile5.zip › 免疫荧光/6.18/BR1-C_0009.tif.frames/BR1-C_0009_T001.tif]

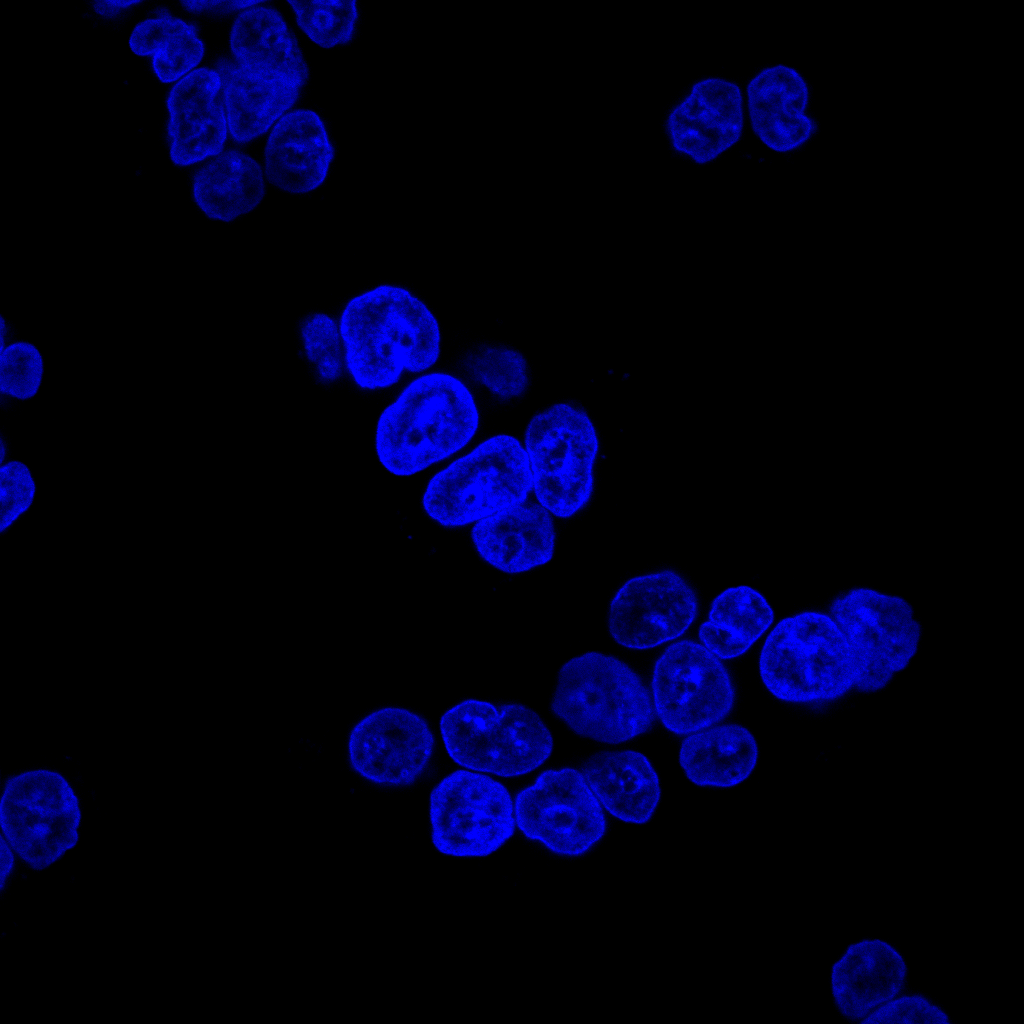

Supplement: Supplementary file 5 [file SupplementaryFile5.zip › 免疫荧光/6.18/BR1-C_0010.tif.frames/BR1-C_0010_C001T001.tif]

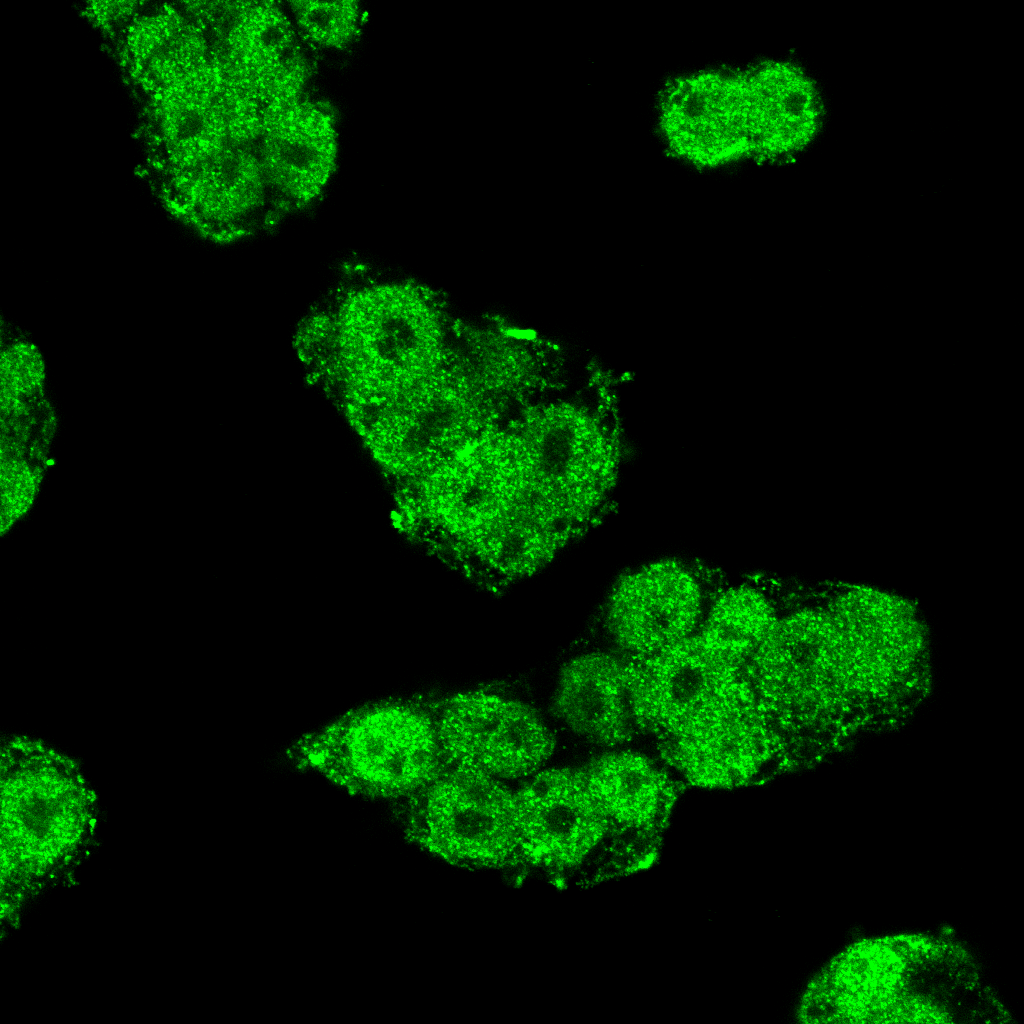

Supplement: Supplementary file 5 [file SupplementaryFile5.zip › 免疫荧光/6.18/BR1-C_0010.tif.frames/BR1-C_0010_C002T001.tif]

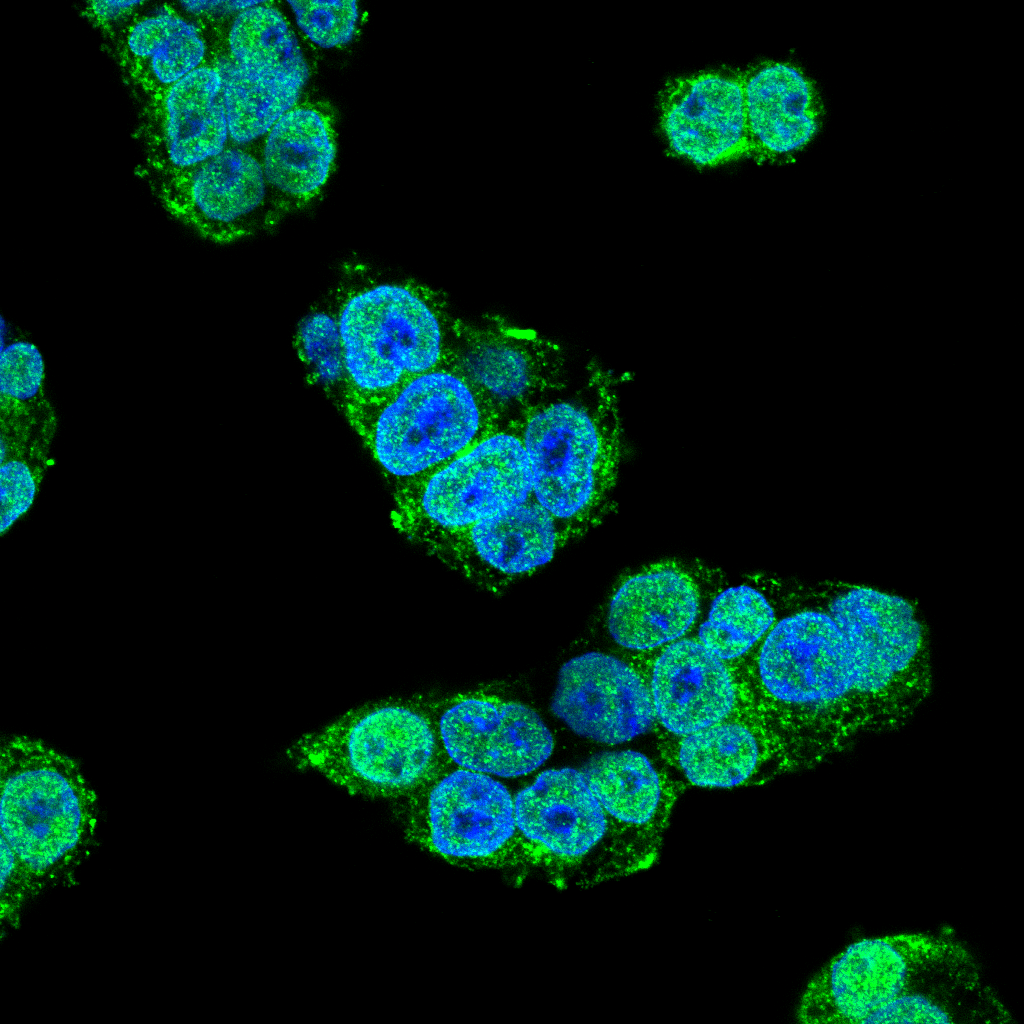

Supplement: Supplementary file 5 [file SupplementaryFile5.zip › 免疫荧光/6.18/BR1-C_0010.tif.frames/BR1-C_0010_T001.tif]

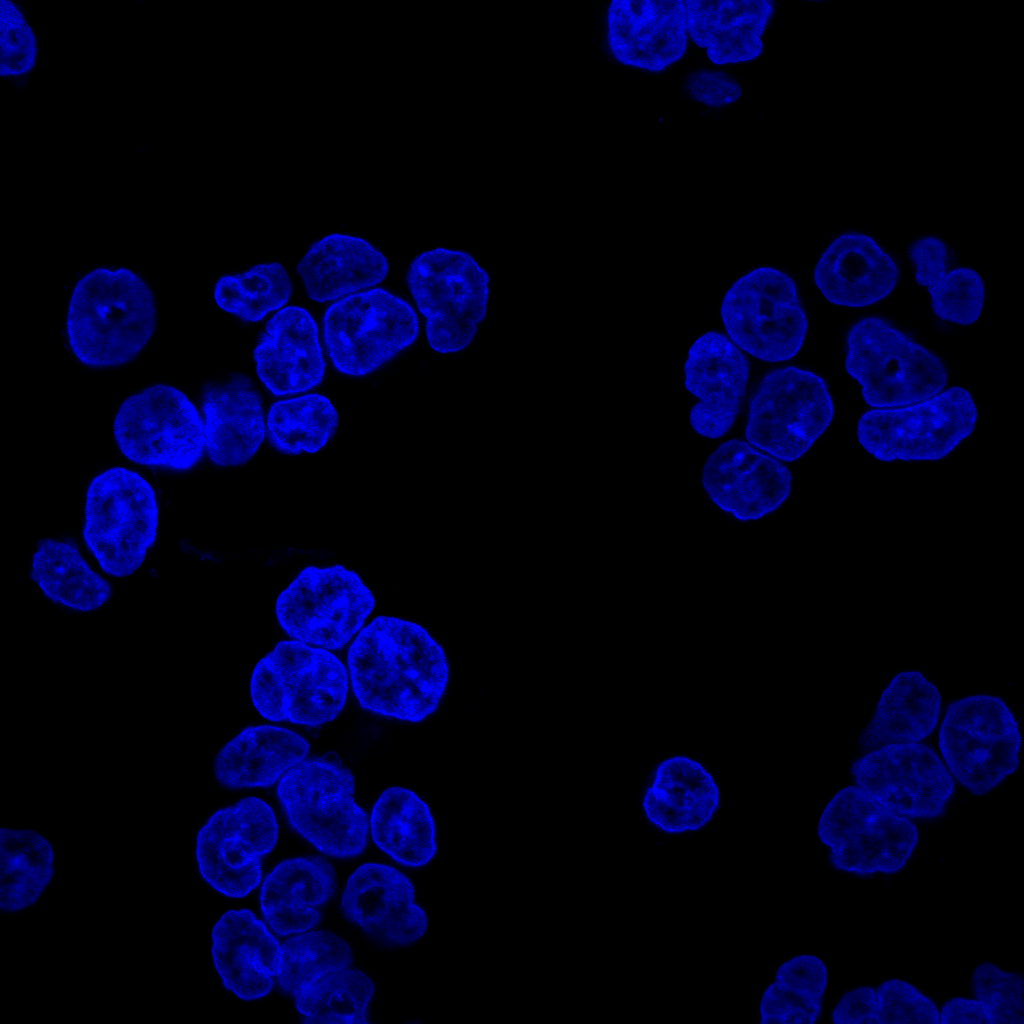

Supplement: Supplementary file 5 [file SupplementaryFile5.zip › 免疫荧光/6.18/BR1-C_0011.tif.frames/BR1-C_0011_C001T001.tif]

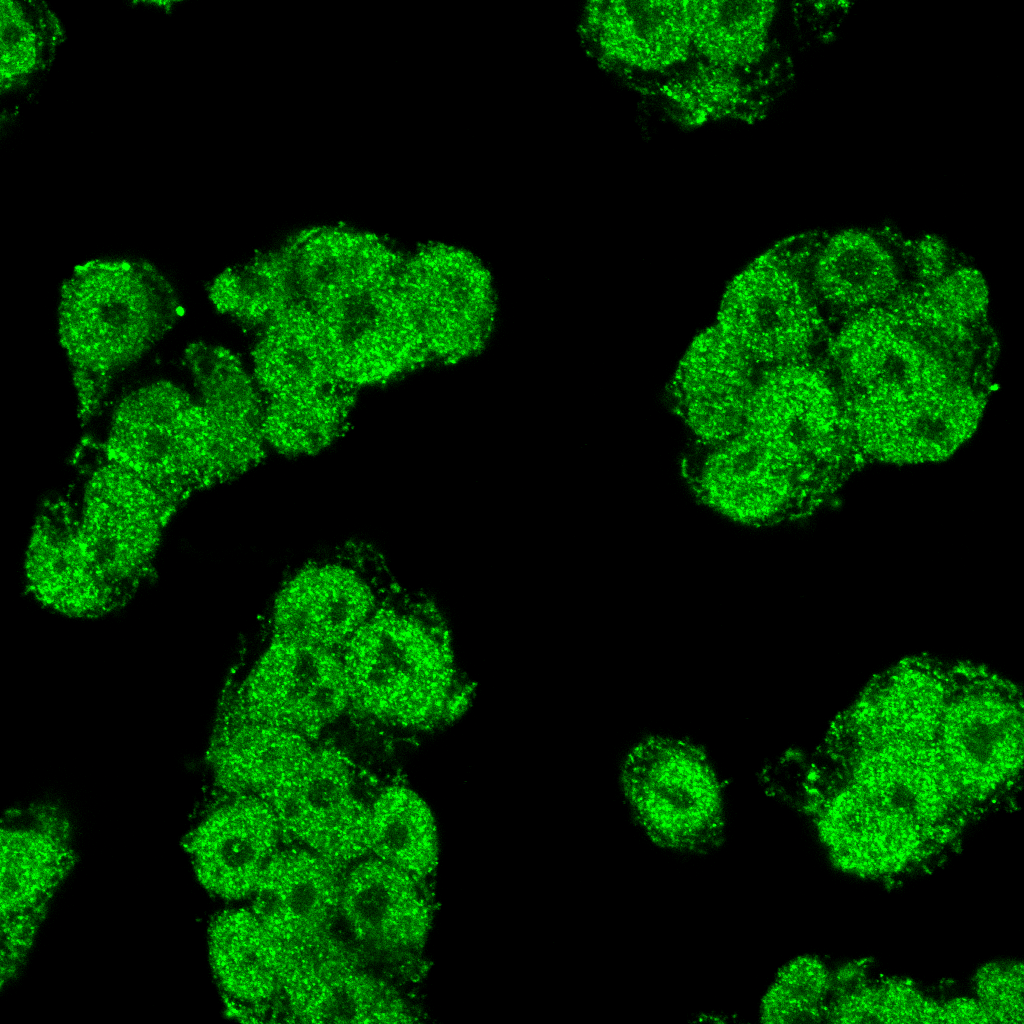

Supplement: Supplementary file 5 [file SupplementaryFile5.zip › 免疫荧光/6.18/BR1-C_0011.tif.frames/BR1-C_0011_C002T001.tif]

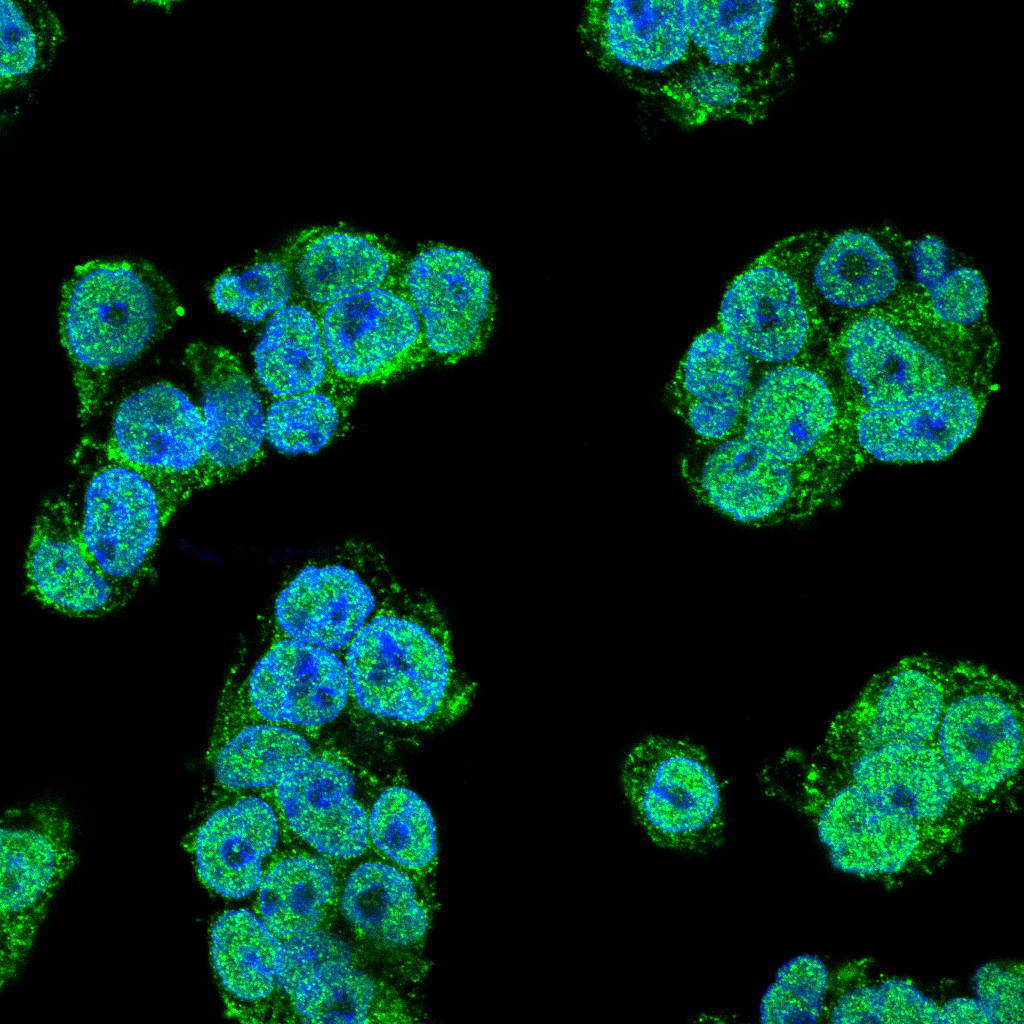

Supplement: Supplementary file 5 [file SupplementaryFile5.zip › 免疫荧光/6.18/BR1-C_0011.tif.frames/BR1-C_0011_T001.tif]

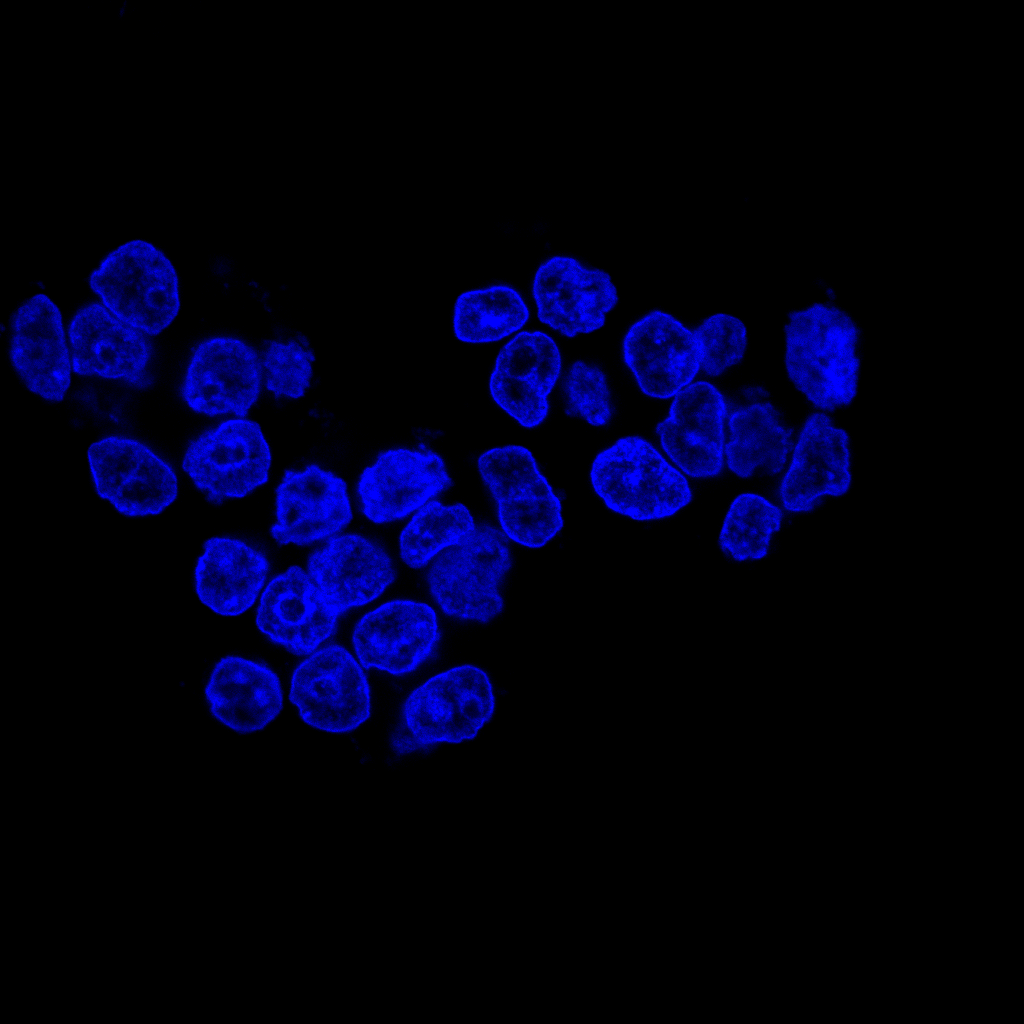

Supplement: Supplementary file 5 [file SupplementaryFile5.zip › 免疫荧光/6.18/BR1-Y.tif.frames/BR1-Y_C001T001.tif]

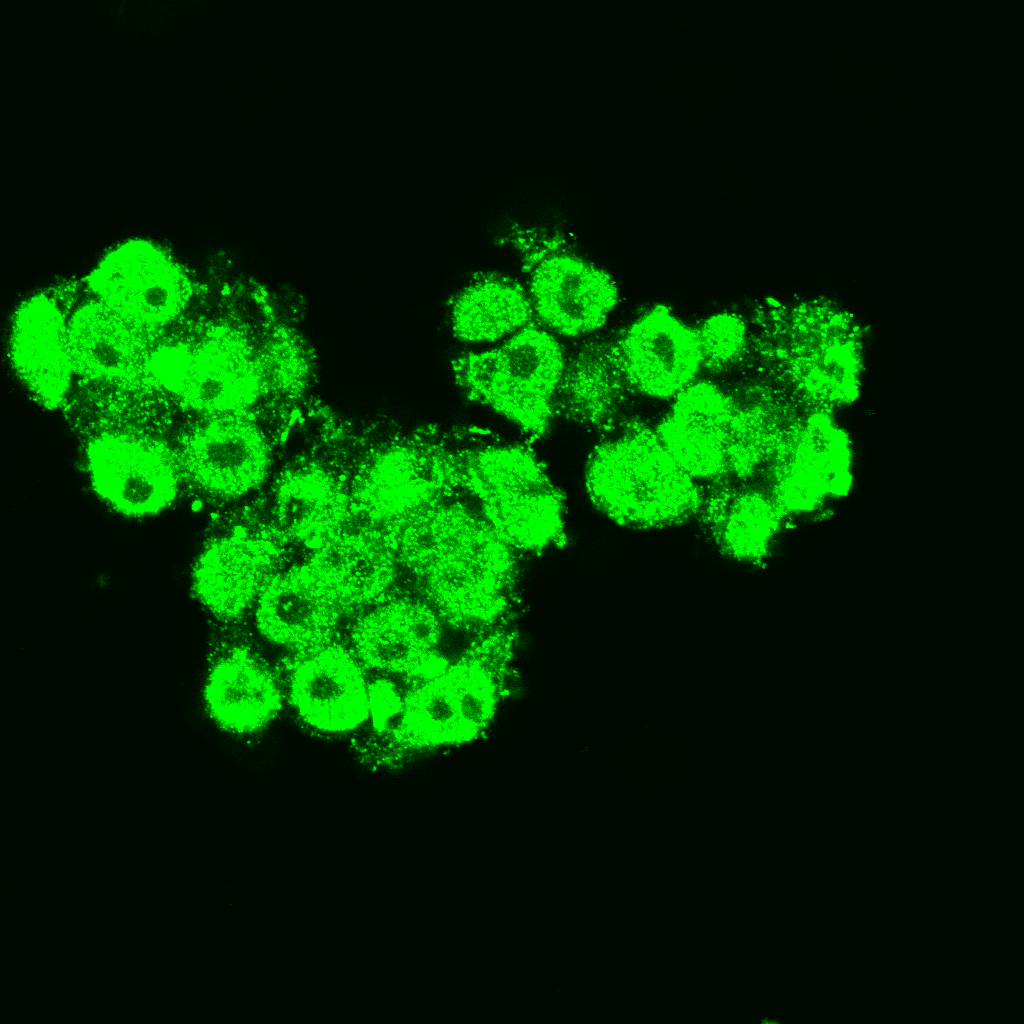

Supplement: Supplementary file 5 [file SupplementaryFile5.zip › 免疫荧光/6.18/BR1-Y.tif.frames/BR1-Y_C002T001.tif]

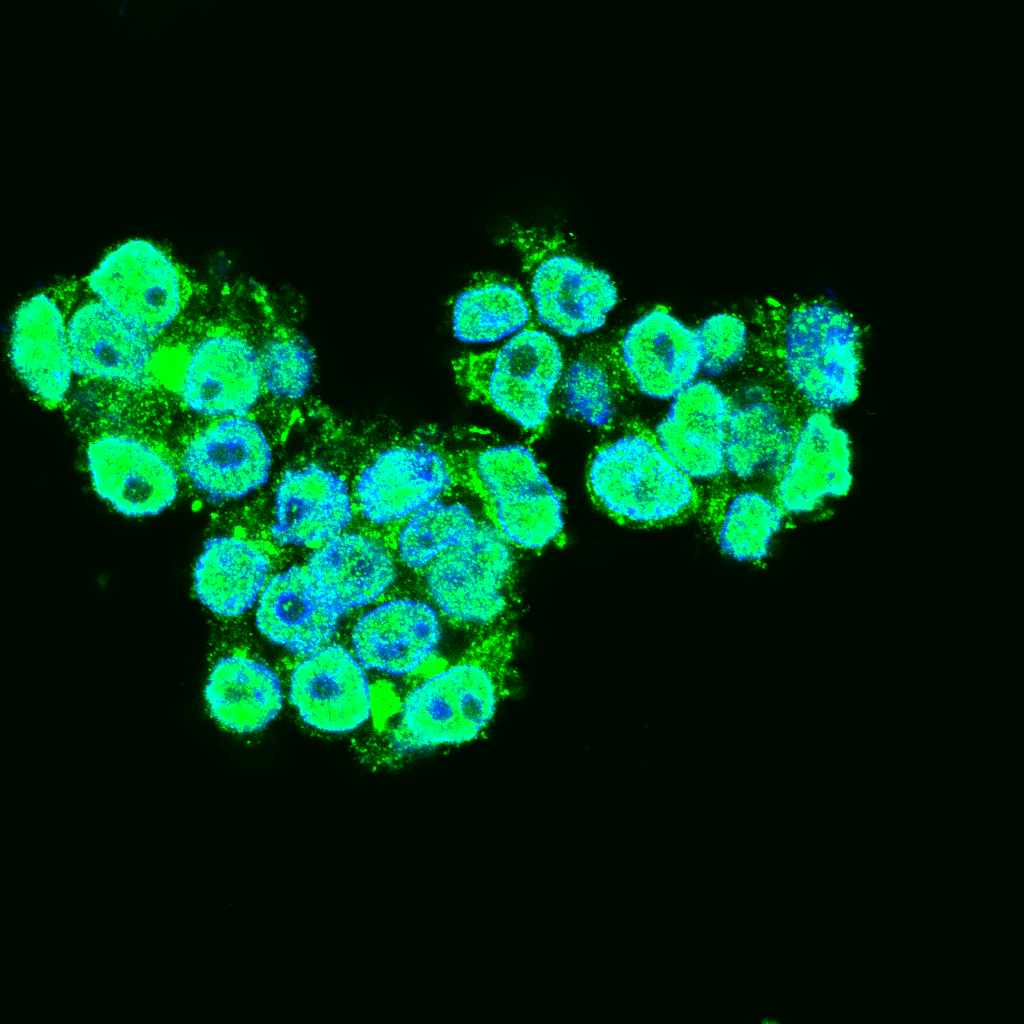

Supplement: Supplementary file 5 [file SupplementaryFile5.zip › 免疫荧光/6.18/BR1-Y.tif.frames/BR1-Y_T001.tif]

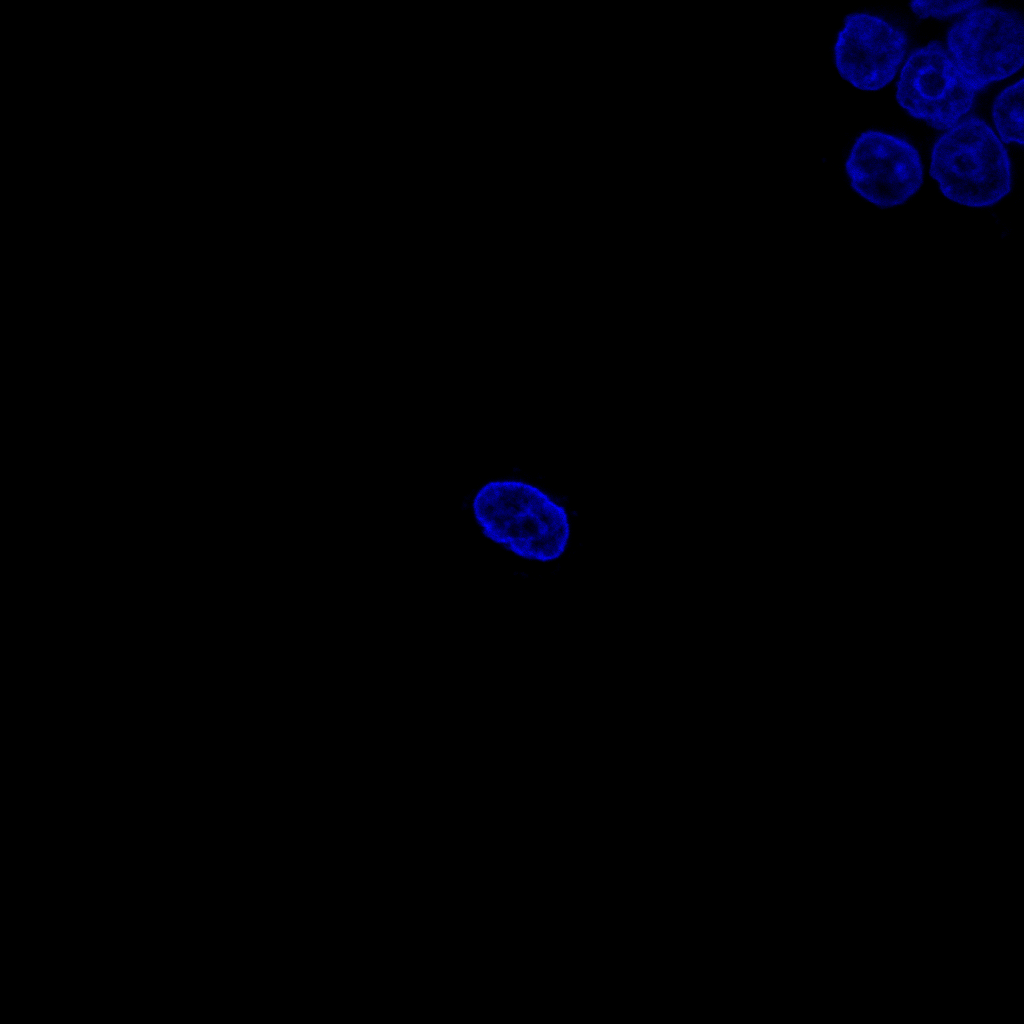

Supplement: Supplementary file 5 [file SupplementaryFile5.zip › 免疫荧光/6.18/BR1-Y_0001.tif.frames/BR1-Y_0001_C001T001.tif]

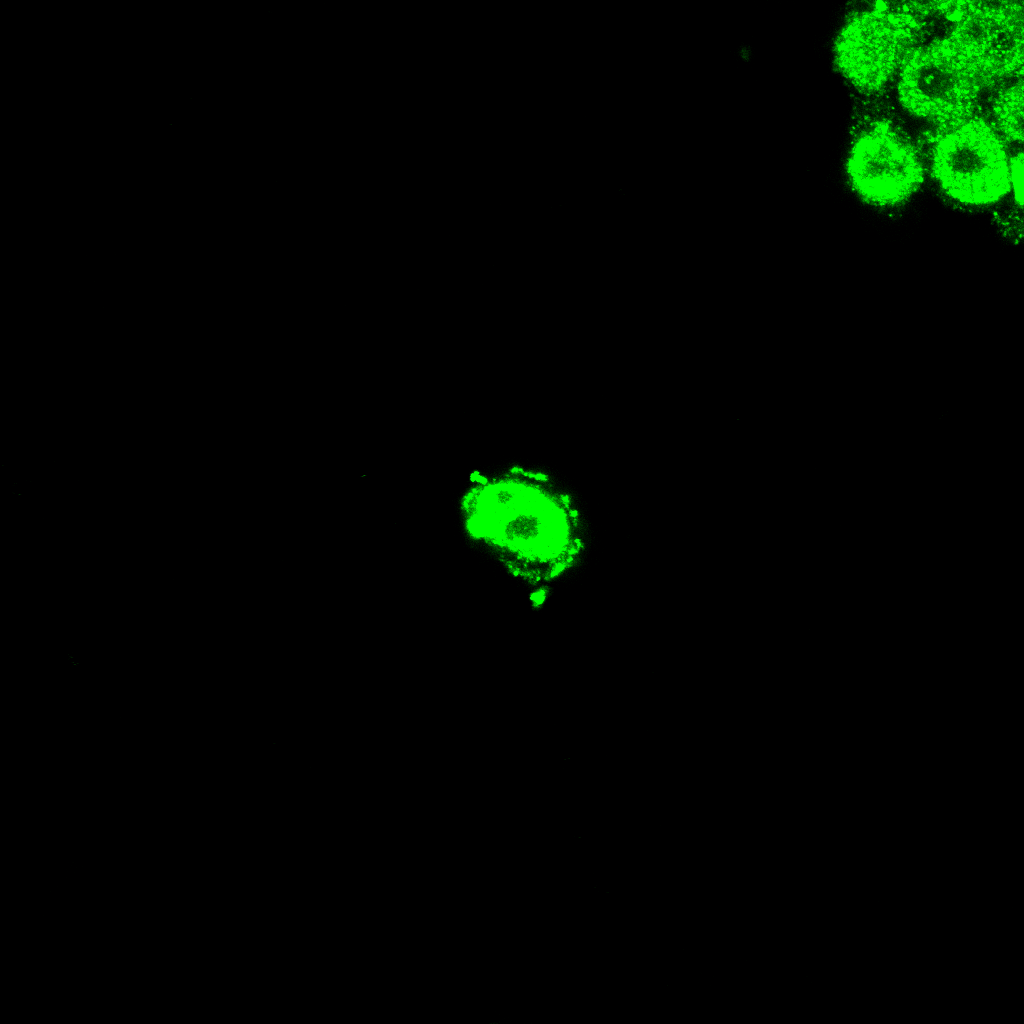

Supplement: Supplementary file 5 [file SupplementaryFile5.zip › 免疫荧光/6.18/BR1-Y_0001.tif.frames/BR1-Y_0001_C002T001.tif]

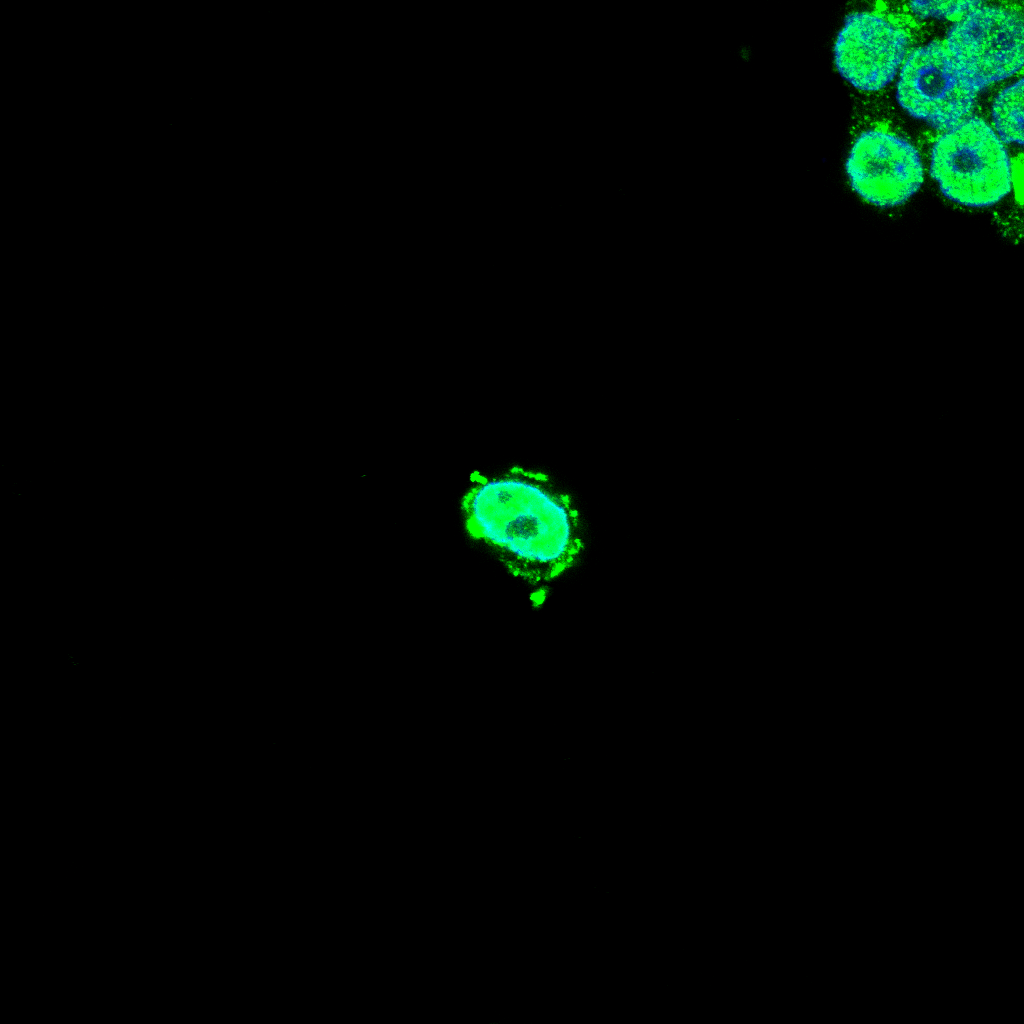

Supplement: Supplementary file 5 [file SupplementaryFile5.zip › 免疫荧光/6.18/BR1-Y_0001.tif.frames/BR1-Y_0001_T001.tif]

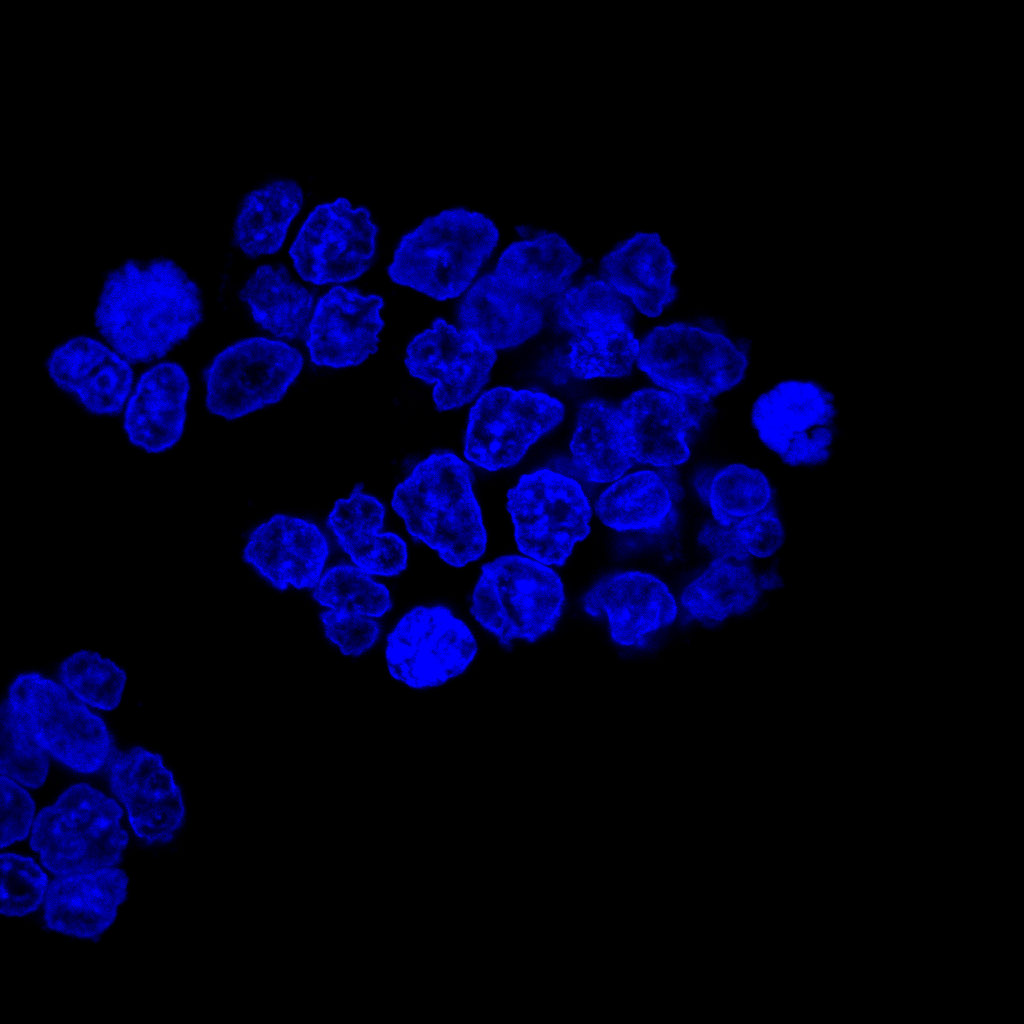

Supplement: Supplementary file 5 [file SupplementaryFile5.zip › 免疫荧光/6.18/BR1-Y_0002.tif.frames/BR1-Y_0002_C001T001.tif]

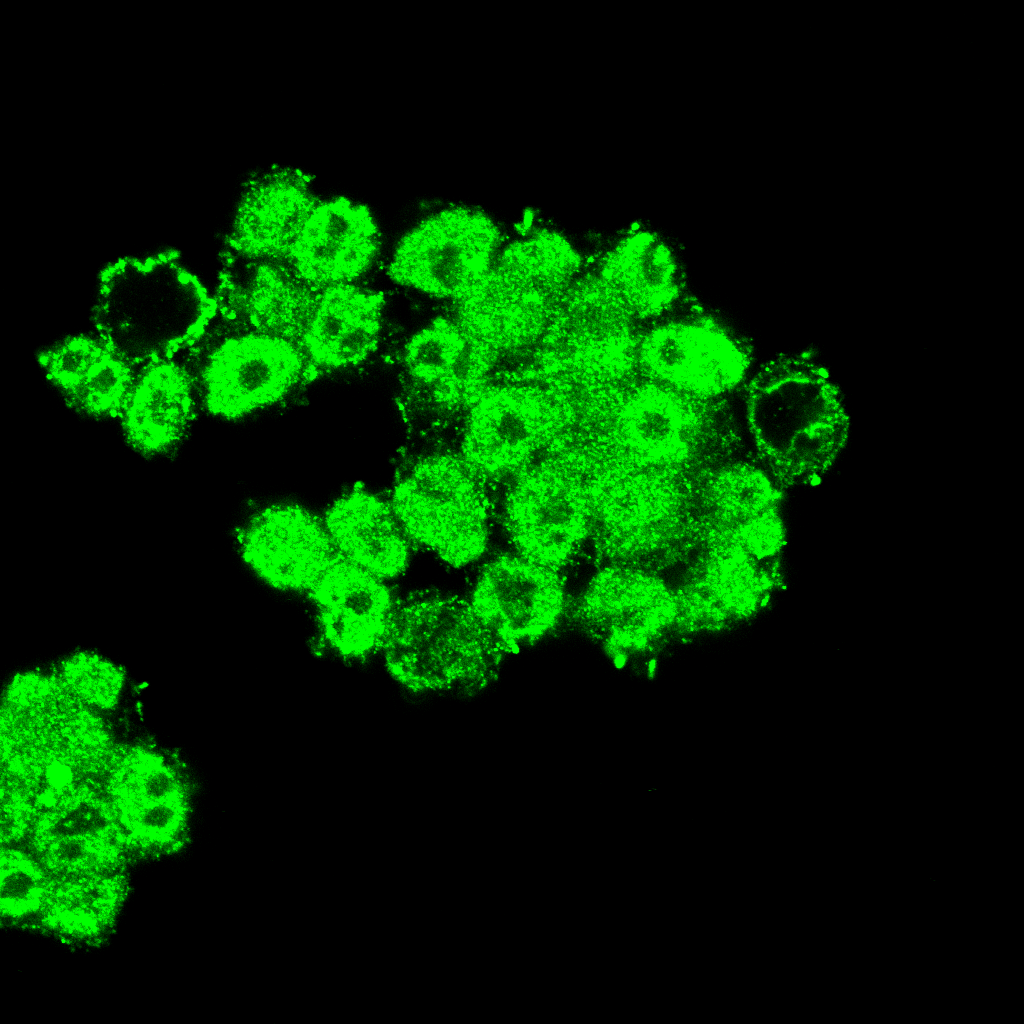

Supplement: Supplementary file 5 [file SupplementaryFile5.zip › 免疫荧光/6.18/BR1-Y_0002.tif.frames/BR1-Y_0002_C002T001.tif]

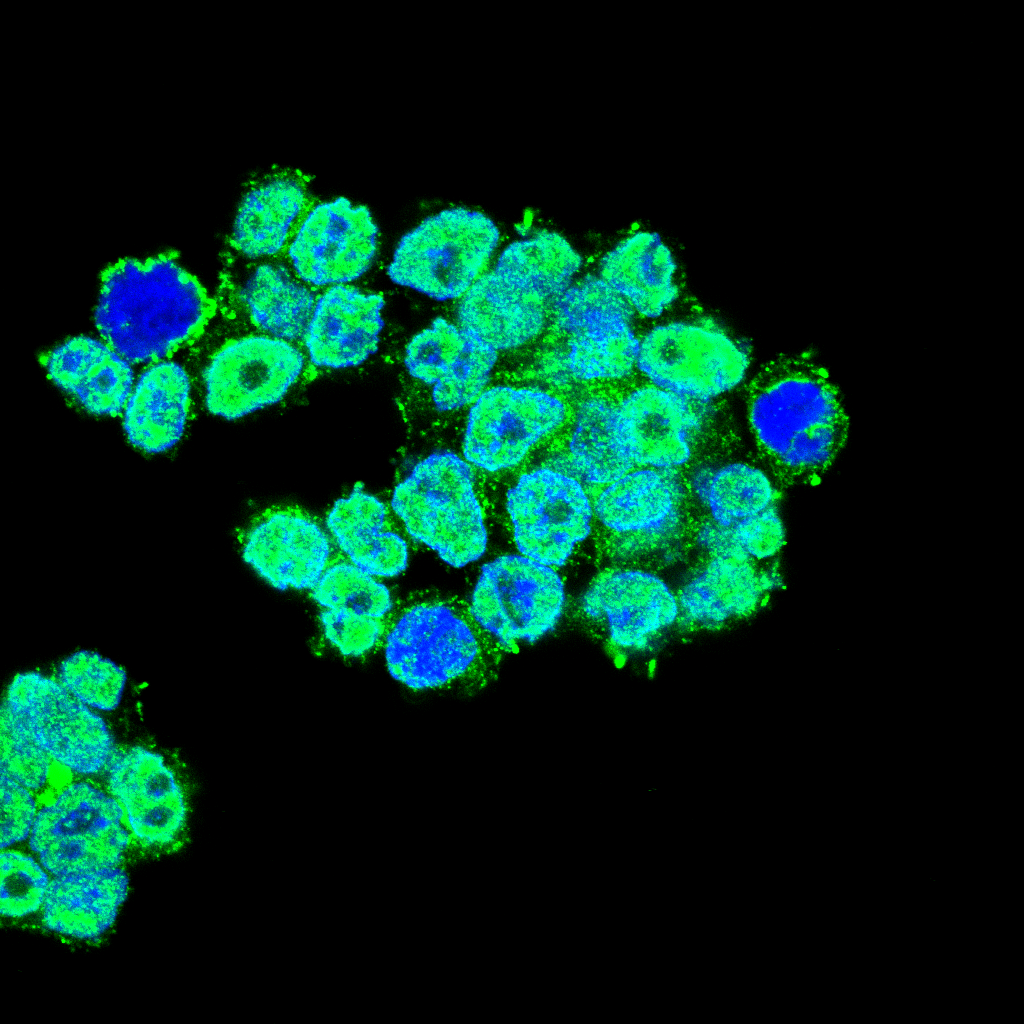

Supplement: Supplementary file 5 [file SupplementaryFile5.zip › 免疫荧光/6.18/BR1-Y_0002.tif.frames/BR1-Y_0002_T001.tif]
